# Robust induction of primordial germ cells of white rhinoceros on the brink of extinction

**DOI:** 10.1126/sciadv.abp9683

**Published:** 2022-12-09

**Authors:** Masafumi Hayashi, Vera Zywitza, Yuki Naitou, Nobuhiko Hamazaki, Frank Goeritz, Robert Hermes, Susanne Holtze, Giovanna Lazzari, Cesare Galli, Jan Stejskal, Sebastian Diecke, Thomas B. Hildebrandt, Katsuhiko Hayashi

**Affiliations:** ^1^ Department of Genome Biology, Graduate School of Medicine, Osaka University, Osaka 565-0871, Japan.; ^2^ Technology Platform Pluripotent Stem Cells, Max Delbrück Center for Molecular Medicine in the Helmholtz Association (MDC), Berlin 13125, Germany.; ^3^ Department of Stem Cell Biology and Medicine, Graduate School of Medical Sciences, Kyushu University, Maidashi 3-1-1, Higashi-ku, Fukuoka 812-8582, Japan.; ^4^ Leibniz Institute for Zoo and Wildlife Research, Berlin 10315, Germany.; ^5^ Avantea, Laboratory of Reproductive Technologies, Cremona 26100, Italy.; ^6^ Fondazione Avantea, Cremona 26100, Italy.; ^7^ ZOO Dvůr Králové, Dvůr Králové nad Labem 54401, Czech Republic.; ^8^ Freie Universitaet Berlin, Berlin D-14195, Germany.

## Abstract

In vitro gametogenesis, the process of generating gametes from pluripotent cells in culture, is a powerful tool for improving our understanding of germ cell development and an alternative source of gametes. Here, we induced primordial germ cell–like cells (PGCLCs) from pluripotent stem cells of the northern white rhinoceros (NWR), a species for which only two females remain, and southern white rhinoceros (SWR), the closest species to the NWR. PGCLC differentiation from SWR embryonic stem cells is highly reliant on bone morphogenetic protein and WNT signals. Genetic analysis revealed that SRY-box transcription factor 17 (SOX17) is essential for SWR-PGCLC induction. Under the defined condition, NWR induced pluripotent stem cells differentiated into PGCLCs. We also identified cell surface markers, CD9 and Integrin subunit alpha 6 (ITGA6), that enabled us to isolate PGCLCs without genetic alteration in pluripotent stem cells. This study provides a first step toward the production of NWR gametes in culture and understanding of the basic mechanism of primordial germ cell specification in a large animal.

## Teaser

Primordial germ cell-like cells are induced from Southern and Northern White Rhinoceros as a first step toward conservation.

## INTRODUCTION

As a result of various human activities such as habitat destruction and poaching, our planet is currently undergoing its sixth mass extinction event (*1*–*3*). Large mammalian species are particularly sensitive to these human impacts because of their low recovery potential, which, in turn, is attributable to the long periods of time required for their sexual maturity and pregnancy and their limited number of progeny. The northern white rhinoceros (*Ceratotherium simum cottoni*; hereinafter NWR) is categorized as “extinct in the wild” on the IUCN Red List of Threatened Species. In the 1960s, the number of NWRs was estimated at 2250 (*4*). Early in this century, the number had dwindled to only four wild animals mainly due to poaching and warfare, and the last confirmation of a live wild NWR was in 2006 (*5*, *6*). The last male NWR, who was given the name Sudan, died in 2018, and there are now thought to be only two females left on Earth, despite a decades-long worldwide effort to conserve this species, both by eradicating rhinoceros poaching and by establishing an adequate reproduction program (*5*, *6*). In light of this severely advanced process of species extinction, which has outpaced traditional breeding programs and habitat conservation modalities, an alternative way of thinking about this problem is vital and extremely urgent.

One option is to adapt advanced assisted reproductive technologies (aARTs) developed for human and livestock animal reproduction for use in NWR (*7*). Various groups have already developed basic aARTs for use in the species closest to NWR—namely, the southern white rhinoceros (*Ceratotherium simum simum*; hereinafter SWR). The adapted technologies include artificial insemination (*8*, *9*), ovum pickup including induction of ovulation (*10*, *11*), and intracytoplasmic sperm injection, followed by in vitro culture to blastocysts (*10*). On the basis of the similar reproductive physiology between SWR and NWR, as evidenced by their ability to breed, it is expected that the development of aARTs will open an avenue to repopulation of NWRs. This development has already allowed the derivation of embryonic stem cells (ESCs) from SWR blastocysts (*10*). SWR-ESCs share features in common with pluripotent stem cells, including indefinite self-renewal potential and the ability to differentiate into three germ layers. Considering the tremendous effort required to establish ESCs from large animal species, i.e., cows and pigs, it was unexpected that SWR-ESC establishment was achieved at the first attempt using only a small number of blastocysts. Nevertheless, SWR-ESCs are irreplaceable materials as a prototype of pluripotent stem cells with less clonal variation in general and, hence, would provide a platform to evaluate induced pluripotent stem cells (iPSCs) from NWR somatic cells (*12*–*14*). Because pluripotent stem cells have the potential to differentiate into the germ cell lineage through either chimera animals or in vitro differentiation, SWR-ESCs and NWR-iPSCs would provide an infinite source of gametes.

Derivation of gametes from pluripotent stem cells, a process known as in vitro gametogenesis (IVG), has rapidly evolved over the past decade (*15*). In mice, both ESCs and iPSCs have been differentiated into primordial germ cell–like cells (PGCLCs) (*16*), which are equivalent to primordial germ cells (PGCs), the origin of eggs and sperm. The resulting mouse PGCLCs were fully functional, developing into fertile eggs or sperm upon transplantation into the ovary and testis, respectively (*16*, *17*). Subsequent studies established culture systems that produce functional eggs and sperm from PGCLCs in culture through reconstitution of the gonadal environment (*18*–*20*). Moreover, PGCLCs can be induced from pluripotent stem cells not only in mice but also in humans, cynomolgus monkeys, and rabbits (*21*–*24*). In all species in which this induction has been attempted, the resulting PGCLCs were highly similar to bona fide PGCs in vivo with respect to gene expression and characteristic epigenetic features, ensuring further application of IVG to a wide range of animals. Therefore, the successful application of IVG to endangered species would be a cutting-edge strategy that could ultimately rescue many animal species from extinction. Here, we report the establishment of PGCLC induction from SWR-ESCs and NWR-iPSCs and provide a critical evaluation of their procedure of differentiation.

## RESULTS

### Reconstitution of PGC specification in SWR-ESCs

**Fig. 1.**
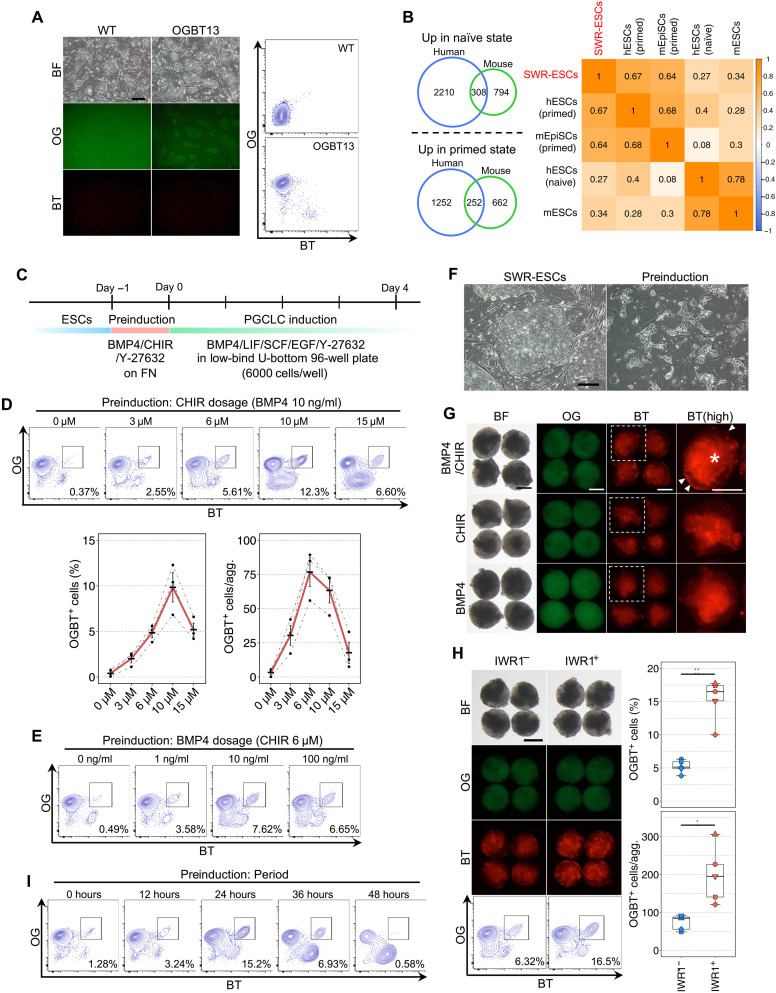
Induction of OGBT-positive cells from SWR-ESCs. (**A**) OGBT reporter SWR-ESCs. Scale bar, 200 μm. BF, bright field; WT, wild type. (**B**) Pluripotent state of SWR-ESCs. The Venn diagrams identify genes up-regulated in the naïve or primed state in human and mouse pluripotent stem cells (*52*, *53*). The heatmap shows correlation coefficients of gene expression representing the pluripotent state. *n* = 2, biologically independent experiments. mEpiSCs, mouse epiblast stem cells. (**C**) Time course of SWR-PGCLC induction. FN, fibronectin. (**D**) Effect of CHIR dosage during preinduction. OGBT SWR-ESC derivatives at day 4 of PGCLC induction are shown. The graphs show a summary of the percentage and number of OGBT-positive cells. *n* = 3, biologically independent experiments shown with SE. (**E**) Effect of BMP4 dosage during preinduction. OGBT SWR-ESC derivatives at day 4 of PGCLC induction are shown. *n* = 2, biologically independent experiments. (**F**) Morphology after preinduction with 6 μM CHIR. Scale bar, 200 μm. (**G**) Formation of BT-positive cells at day 4 of PGCLC induction. BT-positive cells (arrowheads) and an autofluorescence signal presumably derived from dead cells (asterisk) are shown. Scale bars, 200 μm. (**H**) Effect of IWR1 during PGCLC induction. The images and FACS analyses of OGBT SWR-ESC derivatives at day 4 of PGCLC induction. The box-and-whisker plots are a summary of the percentage and number of OGBT-positive cells. *n* = 5, biologically independent experiments. Scale bar, 200 μm. *P < 0.05 and **P < 0.01, Student’s *t* test. (**I**) Effect of the duration of preinduction. FACS plots show OGBT SWR-ESC derivatives at day 4 of PGCLC induction. *n* = 2, biologically independent experiments.

To monitor the process of PGC(LC) specification, we first generated a reporter SWR-ESC line, in which *EGFP* and *tdTomato* with a 2A sequence were inserted into *POU5F1/OCT3/4* and *PRDM1*/*BLIMP1* loci, respectively (fig. S1A). The specific expression of these genes in pluripotent stem cells and PGCs is highly expected, as evident by the conserved expression profile of *POU5F1* and *PRDM1* among a broad range of mammalian species, including mice, rabbits, pigs, and primates (*21*, *22*, *24*–*27*). By successive transfections of gene-targeting vectors with Cas9- and guide RNA (gRNA)–expression vectors for each gene locus, followed by a drug selection (fig. S1B), we successfully isolated six SWR-ESC lines bearing targeted integration of reporter constructs in both gene loci, which we designated the *POU5F1/OCT3/4-EGFP* (OG) and *PRDM1/BLIMP1-tdTomato* (BT) (OGBT) SWR-ESC lines. Inspecting one of the OGBT lines, OGBT13 bearing the reporter genes in a heterozygous fashion (fig. S1A), we found that the OG was detectable under fluorescent microscopy and by fluorescence-activated cell sorting (FACS), while no BT was detectable (Fig. 1A).

Because the ability of ESCs to differentiate into PGCLCs is highly dependent on their pluripotent state (*16*, *21*, *24*), we checked the pluripotent state of the OGBT SWR-ESCs. Referring to a list of genes differentially expressed between the naïve and primed states, we chose 308 and 252 genes that are up-regulated in the naïve and primed states, respectively, in both mouse ESCs and human ESCs/iPSCs (Fig. 1B). A correlation matrix of these gene expressions showed that SWR-ESCs were in the primed state when cultured in Dulbecco’s modified Eagle’s medium (DMEM)/F12 supplemented with 20% knockout serum replacement (KSR) and basic fibroblast growth factor (bFGF; 10 ng/ml). Because human and monkey ESCs in the primed state readily differentiate into PGCLCs (*21*, *23*), we next tried to induce PGCLCs in Glasgow minimal essential medium (GMEM) supplemented with 15% KSR containing bone morphogenetic protein 4 (BMP4), leukemia inhibitory factor (LIF), stem cell factor (SCF), epidermal growth factor (EGF), and Y-27632 (hereinafter GK15 + BLSEY), either directly or via an incipient mesoderm-like cells (iMeLCs) condition containing activin A and CHIR99021 (CHIR), a WNT agonist, as used for human PGCLC induction (*24*). Under these conditions, however, there was no OGBT induction at day 4 of PGCLC induction, irrespective of whether the iMeLC culture condition was used (fig. S2A). This incompetent state was not resolved by culturing OGBT SWR-ESCs under the 4i condition [i.e., in medium containing inhibitors of mitogen-activated protein kinase (MAPK) kinase (MEK)/extracellular signal–regulated kinase, glycogen synthase kinase 3β, c-Jun N-terminal kinase (JNK), and p38, as well as LIF, bFGF, and transforming growth factor–β] that enhanced PGCLC induction from human ESCs/iPSCs. Under the 4i condition, the OGBT SWR-ESCs rapidly lost their OG expression and self-renewal capacities (fig. S2B).

Both BMP and WNT signals are required in mouse and human PGCLC specification (*16*, *21*, *24*, *28*, *29*). Notably, the timing and duration of WNT signaling, before the activation of BMP signaling, are critical for conferring PGC competence to the pluripotent cells in mice and humans: Specifically, a prolonged activation of WNT signaling compromises PGC competence (*28*, *29*). We therefore shortened the preinduction period to 24 hours and added BMP4 at various concentrations for simultaneous stimulation with CHIR (Fig. 1C). Induction of PGCLCs in GK15 + BLSEY for 4 days, followed by FACS analysis, revealed the appearance of OGBT double-positive cells, albeit a relatively small number, in response to BMP4 (fig. S2C), indicating that simultaneous stimulation with BMP4 and CHIR might enhance the PGC competence in OGBT SWR-ESCs. We then examined the optimal concentration of CHIR in the preinduction culture and found that a higher number of OGBT double-positive cells was induced from the cells primed with 6 μM CHIR and BMP4 (10 ng/ml) (Fig. 1D). In addition, we tried again to optimize the concentration of BMP4 for use with 6 μM CHIR in the preinduction culture and found that BMP4 (10 ng/ml) provided the highest proportion of OGBT double-positive cells (Fig. 1E). On the basis of these results, we fixed the concentrations of CHIR and BMP4 at 6 μM and 10 ng/ml, respectively, for the preinduction. During the preinduction culture, ESCs transformed into iMeLC-like cells with a flat morphology and distinct cell-to-cell boundaries (Fig. 1F). Under a fluorescent microscope, the BT-positive cells were preferentially located at the edge of the aggregations (Fig. 1G). This pattern of cell distribution is consistent with that in PGCLC induction in mice and humans (*16*, *21*).

Although OGBT double-positive cells were induced, the number of these cells per aggregate still averaged less than 100 (Fig. 1D), limiting their potential application. We therefore tried to refine the induction medium, GK15 + BLSEY, that was used after the preinduction stage. Because, as described above, excessive WNT signaling reduces the efficiency of PGCLC differentiation, we added IWR1, an inhibitor of WNT signaling, to GK15 + BLSEY. Under this condition, both the percentage and the cell number of OGBT double-positive cells became two to three times higher than in the culture without IWR1 (Fig. 1H). A similar effect was observed in the differentiation of OGBT double-positive cells with other WNT inhibitors, such as XAV939 and IWP2, albeit to a different extent (fig. S2D). Because a more robust induction of OGBT double-positive cells was observed with IWR1 compared to the other WNT inhibitors, we decided to include IWR1 in the induction medium, which we then designated GK15 + BLSEYWi.

Next, we attempted to determine the signals other than BMP4 and WNT that play a role in PGCLC specification in other animals. Activin A is used for preinduction in mouse and human PGCLC induction (*16*, *21*, *24*). In SWR-ESCs, however, addition of activin A to the preinduction medium inhibited the induction of OGBT double-positive cells (fig. S2E). FGF inhibition has been shown to enhance the rate of human PGCLC induction (*24*, *30*), but when PD0399021 (PD), an inhibitor of FGF signaling, was added to the preinduction medium, it had no obvious effect on the induction of OGBT double-positive cells (fig. S2F). When PD was added to the preinduction medium with mouse embryonic fibroblasts (MEFs), it nullified the negative effect of MEFs on the induction of OGBT double-positive cells, suggesting that the FGF signaling provided by MEF had a negative effect, as seen in human PGCLC induction (fig. S2F).

Last, we tested the optimal duration of the preinduction culture using the fixed components determined as described above and found that culture for 24 hours conferred the highest competence for becoming OGBT double-positive cells and that longer duration compromised the competence (Fig. 1I). This temporal acquisition of PGC competence in SWR-ESCs was consistent with that in mouse and human pluripotent stem cells (*16*, *21*, *24*).

### Characterization of OGBT double-positive cells induced from SWR-ESCs

**Fig. 2.**
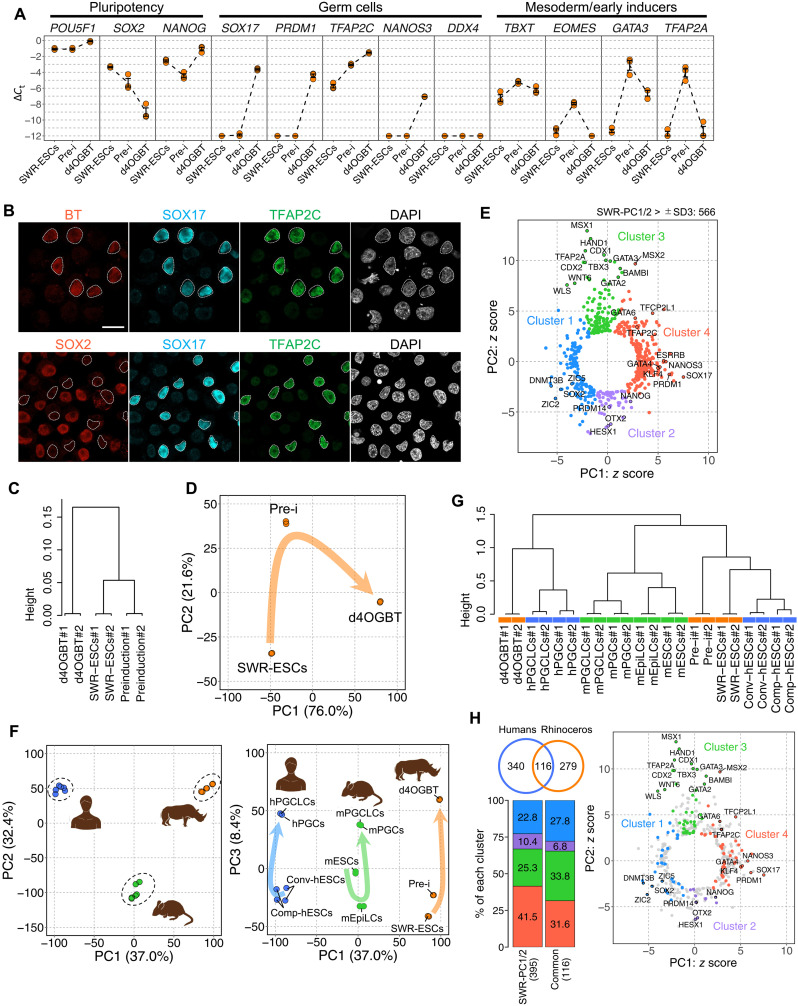
Gene expression profiling of OGBT-positive cells from SWR-ESCs. (**A**) Gene expression dynamics during differentiation. Shown are averaged Δ*C*_t_ values with SE determined by qPCR analysis. *n* = 3, biologically triplicated samples. (**B**) Coexpression of transcription factors in OGBT-positive cells. Immunofluorescent analyses of the transcription factors involved in PGC specification are shown. Scale bar, 20 μm. (**C**) Unsupervised hierarchical clustering of derivatives from SWR-ESCs. (**D**) PCA of transcriptomes of SWR-ESC derivatives. (**E**) Scatterplot of the *z* scores of the genes for the PC1 and PC2. Genes with a radius of >3 SDs (566 genes) are shown. The colors of the plots correspond to the clusters shown in fig. S3B. (**F**) PCA of transcriptomes of ESC derivatives in humans, mice, and SWR. Note that the PCA using PC1 and PC3 showed a similar direction of differentiation. (**G**) Unsupervised hierarchical clustering. The colors correspond to those in (E). (**H**) Scatterplot of the *z* scores of the ortholog gene set involved in PGC specification. In the Venn diagram, the blue and orange circles include 456 human genes involved in PGC specification (*29*) and 395 SWR-PC1/2 genes whose orthologs exist in the gene list of (29), respectively. The bar graphs show the distribution of 395 SWR-PC1/2 genes or 116 genes commonly assigned to the gene clusters in the scatterplot of the *z* scores in (E). The right scatterplot shows the *z* scores of the 395 SWR-PC1/2 genes with the colored plot for the 116 genes. All results are based on biologically duplicated samples.

To characterize OGBT double-positive cells, we first evaluated the gene expression dynamics during the differentiation by quantitative polymerase chain reaction (qPCR) analysis (Fig. 2A). The expression of early PGC marker genes in mice and/or humans—i.e., *SOX17*, *PRDM1*, *TFAP2C*, and *NANOS3*—increased in OGBT double-positive cells at day 4 of the induction culture, whereas the expression of a late germ cell marker gene, *DDX4*, was not detectable. *POU5F1* and *NANOG* expression was constant, while *SOX2* expression was rapidly down-regulated during the differentiation. The expression of *T/TBXT*, *EOMES*, *GATA3*, and *TFAP2A*, genes critical for the nascent mesoderm and PGC competence in mice (*28*) and/or humans (*29*, *31*, *32*), was temporarily increased in preinduced cells. Note that the characteristic gene expression profiles, such as that comprising the expression of *SOX17*, down-regulation of *SOX2*, and temporal expression of *EOMES*, *GATA3*, and *TFAP2A*, were reminiscent of human PGC specification rather than mouse PGC specification. Consistent with this finding, immunofluorescent analysis showed that BT-positive cells expressed SOX17 and Transcription factor AP-2 gamma (TFAP2C), but not SOX2 (Fig. 2B). These results demonstrated that OGBT double-positive cells share key gene expression features with human PGCLCs.

To further characterize OGBT double-positive cells, we carried out RNA sequencing (RNA-seq) analysis on SWR-ESCs, preinduced cells, and day 4 OGBT double-positive cells. Unsupervised hierarchical clustering using all the expressed genes showed that SWR-ESCs and preinduced cells were relatively close to each other and far from the OGBT double-positive cells (Fig. 2C). During the differentiation, 149 and 569 differentially expressed genes (DEGs) [>4 times, false discovery rate (FDR) < 0.001, logarithm of counts per million reads (logCPM) > 4] were detectable between the SWR-ESCs and the preinduced cells and between the preinduced cells and the OGBT double-positive cells, respectively (fig. S3A). As expected, BMP-responsive and/or WNT-responsive mesodermal genes, such as *GATA3*, *HAND1*, and *TBX3*, were up-regulated in the preinduced cells compared to SWR-ESCs, and many of them were down-regulated in OGBT double-positive cells. On the other hand, genes involved in PGC specification, such as *SOX17*, *PRDM1*, and *NANOS3*, were up-regulated in OGBT double-positive cells compared to the preinduced cells (fig. S3A). Principal components analysis (PCA) showed that the difference in gene expression profiles between SWR-ESCs and preinduced cells was projected to PC2 and that between preinduced cells and OGBT double-positive cells was projected to both PC1 and the opposite direction of PC2 (Fig. 2D). Genes highly contributing to each PC (Z scores > ± 3 SDs: 566 genes termed SWR-PC1/2 genes) could be divided into four clusters (Fig. 2E, fig. S3B, and table S1). BMP-responsive and/or WNT-responsive mesodermal genes were enriched in cluster 3, which drives the differentiation to preinduced cells, while pluripotent markers, such as *NANOG* and *PRDM14*, and anterior markers, such as *OTX2* and *HESX1*, were enriched in cluster 2, which is on the side opposite to cluster 3. As expected, genes involved in PGC specification were enriched in cluster 4. *DNMT3B* and *SOX2*, which should be down-regulated during PGC specification (*21*, *24*, *33*), were categorized into cluster 1. These gene expression analyses implied a conserved mode of PGC specification in rhinoceros: Pluripotent cells temporarily acquire an early mesodermal state, and then some of them switch their fate into PGCs with the expression of PGC-specific transcriptional regulators.

We then compared the transcriptome among these SWR-ESC derivatives and related cell types in humans (conventional hESCs, competent hESCs, day 4 hPGCLCs, and PGCs in vivo) and mice [mESCs, mouse epiblast-like cells (mEpiLCs), day 6 mPGCLCs, and PGCs in vivo]. PCA with PC1 and PC2 using all expressed genes showed that mouse, human, and rhinoceros profiles made clusters in each species (Fig. 2F). However, PCA with PC1 and PC3 showed that the cells differentiate in a similar direction along PC3 according to the sequence of the differentiation (Fig. 2F). This result suggests that the differentiation processes from SWR-ESCs to day 4 OGBT double-positive cells were largely similar to those from pluripotent stem cells to PGCLCs in mice and humans. Unsupervised hierarchical clustering using the SWR-PC1/2 genes showed that day 4 OGBT double-positive cells were similar to human PGCs and hPGCLCs rather than mPGCLCs (Fig. 2G). Among the gene set for human PGC specification (*29*), 456 genes were assigned as orthologs of SWR genes. On the other hand, 395 of 573 SWR-PC1/2 genes were found to be human orthologs in the gene list used for transcriptomic analyses of human PGCLC specification (*29*). A total of 116 genes were shared in common between the two gene sets; these were genes involved in differentiation processes from pluripotent stem cells to PGCLCs via the intermediate cell population (Fig. 2H). Consistent with these findings, comparisons of key gene expressions for PGC specification showed that the patterns in rhinoceros were similar overall to those in humans rather than mice (fig. S3C).

### Functional requirement of *SOX17* for OGBT double-positive cell differentiation

**Fig. 3.**
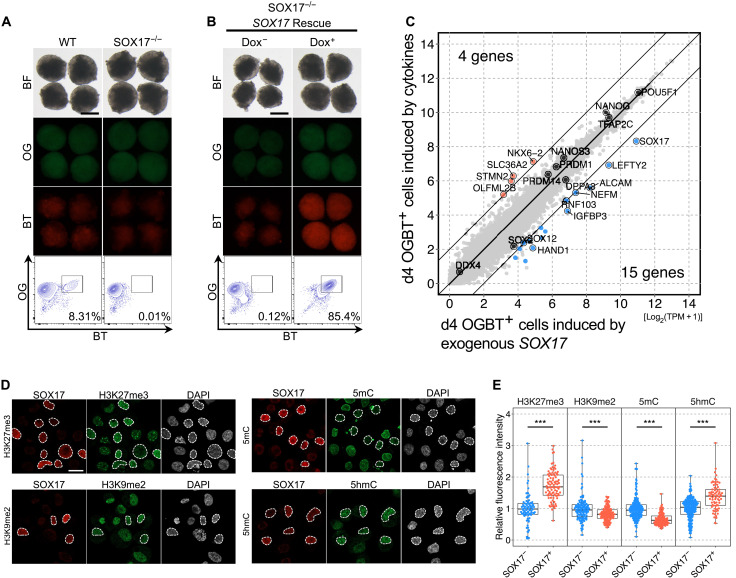
Characterization of OGBT-positive cells from SWR-ESCs. (**A**) *SOX17*-dependent differentiation of OGBT-positive cells. Shown are images and FACS plots of wild-type and *SOX17*^−/−^ OGBT SWR-ESC derivatives at day 4 of PGCLC induction. *n* = 2, biologically independent experiments. Scale bar, 200 μm. (**B**) Restoration of OGBT-positive cell differentiation by exogenous *SOX17* expression. Shown are images and FACS plots of *SOX17*-transgenic OGBT SWR-ESC derivatives at day 4 of PGCLC induction with or without Dox. *n* = 2, biologically independent experiments. Scale bar, 200 μm. (**C**) Comparison of transcriptomes of OGBT-positive cells induced by the cytokines or exogenous *SOX17* expression. Shown are scatter plots of transcriptomes of OGBT-positive cells with DEGs (>4 times, FDR < 0.001, logCPM > 4). (**D**) Epigenetic features in OGBT-positive cells. Shown are the results of immunofluorescence analysis of the epigenetic modification indicated and SOX17 expression in OGBT SWR-ESC derivatives at day 4 of PGCLC induction by cytokines. SOX17-positive cells are enclosed by dashed lines. Scale bar, 20 μm. (**E**) Quantification of the epigenetic modifications in SOX17-positive cells. The box-and-whisker plots show the values relative to the averaged value in SOX17-negative cells. ***P < 0.001, Mann-Whitney *U* test.

Given that the manner of differentiation of OGBT double-positive cells resembles that of human PGCLC differentiation, *SOX17* would play a critical role, as it activates the gene network for PGCLC specification in humans but not in mice (*21*). We then examined the role of *SOX17* in OGBT double-positive cell differentiation from SWR-ESCs with a *SOX17*^−/−^ line generated from OGBT13 SWR-ESCs, in which sequences around the second exon of *SOX17* including the splice acceptor were deleted on both alleles (fig. S4, A and B). From the *SOX17*^−/−^ OGBT13 SWR-ESCs, no OGBT double-positive cells appeared by the preinduction, followed by culture with GK15 + BLSEYWi (Fig. 3A). To exclude the possibility that the defect was caused by off-target genome editing, we rescued *SOX17* expression in *SOX17*^−/−^ OGBT13 SWR-ESCs with a doxycycline (Dox)–inducible *SOX17* expression vector (fig. S4, C and D). In the rescue line, a robust induction (~85%) of OGBT double-positive cells was observed in a Dox-dependent manner (Fig. 3B). Transcriptome analysis revealed that the Dox-induced OGBT double-positive cells were nearly identical to cytokine-induced OGBT double-positive cells: Apart from the expression of *SOX17* including the transgenic transcript, the number of DEGs (>4 times, FDR < 0.001, logCPM > 4) was only 19 genes (Fig. 3C). These results demonstrate that *SOX17* is a key transcription factor essential and sufficient for the differentiation of OGBT double-positive cells.

Because both mouse and human PGCLCs bore characteristic epigenetic properties (*34*–*36*), we evaluated the epigenetic status of cytokine-induced OGBT double-positive cells. Immunofluorescence analyses revealed that day 4 OGBT double-positive cells exhibited a higher level of H3 lysine-27 trimethylation (H3K27me3) and a lower level of histone H3 lysine-9 dimethylation (H3K9me2) compared to neighboring SOX17-negative cells (Fig. 3, D and E). With respect to DNA methylation, OGBT double-positive cells exhibited a lower level of 5-methylcytosine (5mC) and a higher level of 5-hydroxymethylcytosine (5hmC) compared to SOX17-negative cells (Fig. 3, D and E). These results demonstrated that OGBT double-positive cells share the characteristic epigenetic properties of PGCLCs in mice and humans. This finding, together with the cytokines required for the differentiation, the gene expression profile, the SOX17-dependent manner of differentiation, and the characteristic epigenetic properties, led us to conclude that the OGBT double-positive cells could be defined as PGCLCs derived from SWR-ESCs.

### Further refinement for robust induction of SWR-PGCLCs

**Fig. 4.**
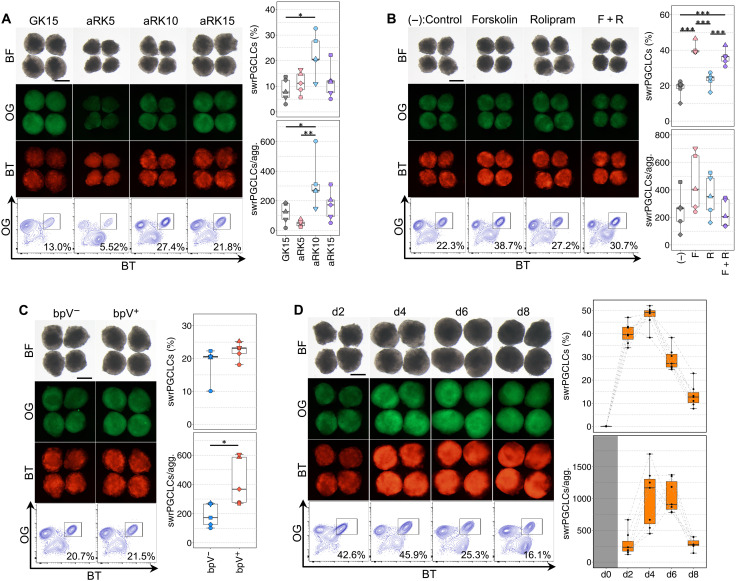
Robust induction of SWR-PGCLCs. (**A**) Refinement of basal medium for SWR-PGCLC induction. Shown are images and FACS plots of PGCLCs at day 4 of induction in the medium indicated. GK15, GMEM containing KSR at 15%; aRK5, aRK10, and aRK15, advanced RPMI 1640 containing KSR at 5, 10, and 15%, respectively. The right box-and-whisker plots summarize the percentage and number of PGCLCs per aggregate. *n* = 5, biologically independent experiments. Scale bar, 200 μm. *P < 0.05 and **P < 0.01, Turkey-Kramer test. (**B**) Effect of cyclic adenosine 5′-monophosphate activation during PGCLC induction. Shown are images and FACS plots of PGCLCs at day 4 of induction with forskolin and/or rolipram. The right box-and-whisker plots summarize the percentage and number of PGCLCs per aggregate. *n* = 5, biologically independent experiments. Scale bar, 200 μm. ***P < 0.001, Turkey-Kramer test. (**C**) Effect of bpV during PGCLC induction. Shown are images and FACS plots of PGCLCs at day 4 of induction with or without bpV. The right box-and-whisker plots summarize the percentage and number of PGCLCs per aggregate. *n* = 5, biologically independent experiments. Scale bar, 200 μm. *P < 0.05, Student’s *t* test. (**D**) Effect of the length of the PGCLC induction period. Shown are images and FACS plots of PGCLCs at the indicated days of induction. The PGCLC induction condition in this experiment was aRK10 + BLSEYF2i. The right box-and-whisker plots summarize the percentage and number of PGCLCs per aggregate. Scale bar, 200 μm. *n* = 7, biologically independent experiments.

During the preparation of this study, there were several updates to the conditions for maintenance of SWR-ESCs and PGCLC induction in mice and primates (*23*, *25*, *37*, *38*). On the basis of these key related studies, we further refined our culture condition, especially with respect to the basal medium and additives for the proliferation of PGCLCs. First, we added activin A for the maintenance of SWR-ESCs, because multiple studies showed the positive effect of activin A on the maintenance of human ESCs/iPSCs (*39*, *40*). Then, we tested the advanced RPMI 1640 with various concentrations of KSR (5, 10, and 15%) and BLSEYWi and found that 10% KSR (aRK10) yielded a higher percentage and number of SWR-PGCLCs (Fig. 4A). We then added forskolin and/or rolipram, both of which have been shown to promote proliferation of mouse and human PGCLCs (*37*, *38*), to aRK10 + BLSEYWi. We found that forskolin, but not rolipram, enhanced the percentage of SWR-PGCLCs (Fig. 4B). In parallel, we tested bpV, an inhibitor of phosphatase and tensin homolog (PTEN) that has a negative impact on proliferation of mouse PGCs (*41*), and found that addition of bpV to aRK10 + BLSEYWi significantly increased the total number of cells in the aggregates with a constant differentiation efficiency, thereby yielding a higher number of SWR-PGCLCs (Fig. 4C). On the basis of these results, we induced SWR-PGCLCs in aRK10 supplemented with forskolin, bpV, and BLSEYWi. Under this condition, nearly half of the cells had become SWR-PGCLCs at 2 and 4 days of induction, and the number of SWR-PGCLCs reached 1019 per aggregation on average at day 4 (Fig. 4D). This series of fine-tuning successfully increased the yield of SWR-PGCLCs by about 15-fold compared with the original condition using GK15 + BLSEY (Fig. 1D).

The transcriptome of SWR-PGCLCs induced under the advanced condition, hereinafter aRK10 + BLSEYF2i, was similar to that of SWR-PGCLCs induced under the condition GK15 + BLSEYWi, as there were only 24 DEGs (>4 times, FDR < 0.001, logCPM > 4) (fig. S5A). In addition, PCA and unsupervised hierarchical clustering using all expressed genes showed that the manner of SWR-PGCLC induction under the aRK10 + BLSEYF2i condition was similar to that under the GK15 + BLSEYWi condition (fig. S5, B and C). Collectively, these results showed that a robust number of SWR-PGCLCs were efficiently induced from SWR-ESCs under the refined condition.

### In vitro expansion of SWR-PGCLCs on feeder cells

**Fig. 5.**
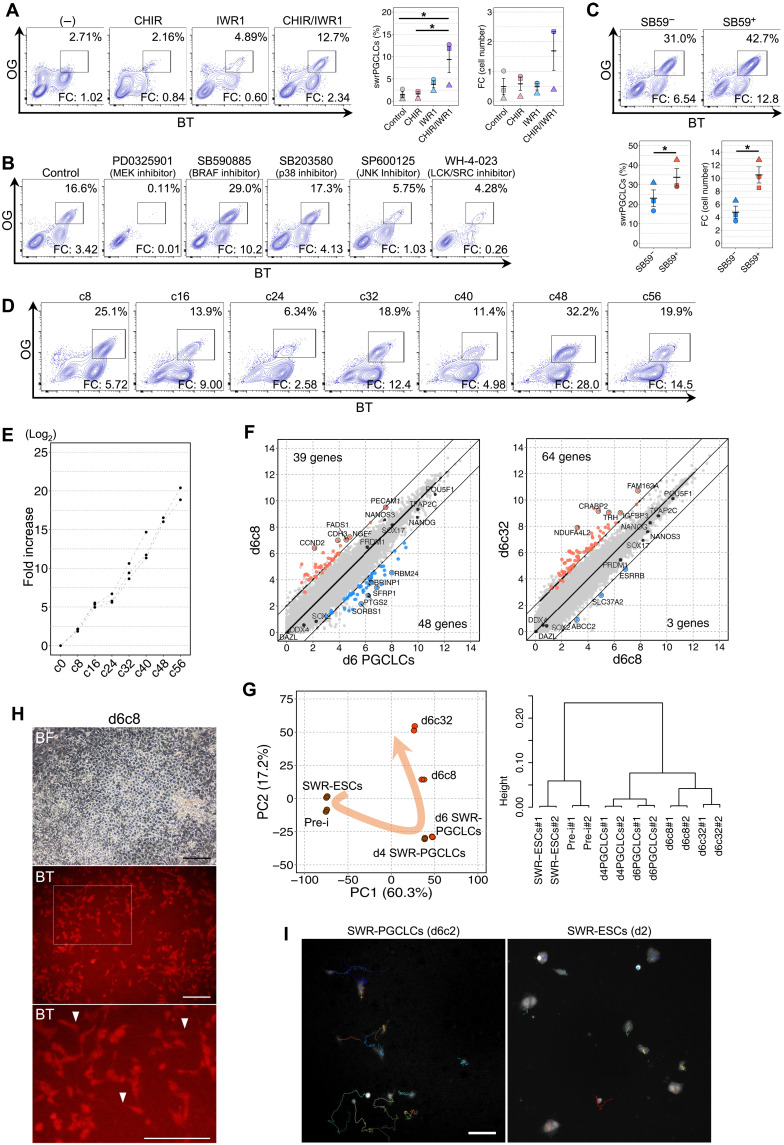
Propagation of SWR-PGCLCs in culture. (**A**) Effect of IWR1 and CHIR on the propagation of SWR-PGCLCs. The fold change (FC) of the SWR-PGCLC number, compared with the initialSWR-PGCLC number spread on the plate, is shown in each FACS plot. The plots at right are a summary of the percentage and FC of SWR-PGCLCs in the aggregates. *n* = 3, biologically independent experiments with SE. *P < 0.05, Tukey-Kramer test. (**B**) Effect of the inhibition of MAPK signaling pathways. (**C**) Effect of SB590885 (SB59). The plots at right are a summary of the percentage and FC of SWR-PGCLCs. *n* = 3, biologically independent experiments. *P < 0.05, Student’s *t* test. (**D**) FACS plot of the cell population during long-term culture. Days of analysis are shown on the top of each FACS plot. (**E**) Biexponential proliferation of SWR-PGCLCs. The plots show the FC increase of SWR-PGCLCs from culture day 0 and the days of culture indicated. (**F**) Comparison of transcriptomes of SWR-PGCLCs in the long-term culture. Shown are the scatter plots of transcriptomes of propagated SWR-PGCLCs. Colored plots show DEGs (>4 times, FDR < 0.001, logCPM > 4). (**G**) PCA and unsupervised hierarchical clustering of derivatives from SWR-ESCs, preinduced cells, and SWR-PGCLCs at the days of culture indicated. (**H**) Images of SWR-PGCLCs at 8 days of long-term culture. The bottom is a high-magnification image of the dotted box in the middle panel. Arrowheads indicate filopodia. Scale bars, 200 μm. (**I**) Tracking of SWR-PGCLC locomotion. Shown are images from the tracking of SWR-PGCLC (left) and SWR-ESC (right) locomotion for 12 hours. Scale bar, 100 μm.

To further characterize SWR-PGCLCs, we assessed whether they have the potential to undergo subsequent developmental processes after PGC specification. Having reported that mouse and human PGCLCs propagate under appropriate conditions (*37*, *38*), we tested propagation of SWR-PGCLCs under various conditions. As both mouse and human PGCLC required feeder cells expressing KIT ligand (KITLG) for their propagation, we generated m220 cells expressing membrane-bound SWR KITLG (hereinafter m246) (fig. S6A). Using m246 as feeder cells, we first cultured SWR-PGCLCs under conditions previously reported to allow human PGCLC expansion (*37*)—namely, GK15 or DMEM containing 15%KSR (DK15) supplemented with fetal bovine serum (FBS), forskolin, and bFGF (see Materials and Methods). However, there were few SWR-PGCLCs after 6 days of culture (fig. S6B). Compared with these media, aRK10 had a slightly more positive effect on the survival of SWR-PGCLCs, but the survival level was still inferior to that of mouse or human PGCLCs in the appropriate medium. This result led us to search for additional reagent(s) required for SWR-PGCLCs propagation. One candidate was WNT signaling, because its effect on PGC proliferation is ambiguous in various species (*42*–*44*), and it has not been intensively investigated in propagation of mouse and human PGCLCs (*37*, *38*, *45*). We therefore added CHIR and/or IWR1, which play roles in WNT signaling, to the culture medium (aRK10 supplemented with FBS, forskolin, bFGF, and SCF). Unexpectedly, these reagents had a synergistic effect on propagation of SWR-PGCLCs, resulting in a significant increase in the percentage of SWR-PGCLCs (Fig. 5A). This synergistic effect was observed irrespective of the dosage of CHIR (fig. S6C). To further improve the condition, we removed individual components from the medium (aRK10 supplemented with FBS, forskolin, bFGF, SCF, CHIR, and IWR1) and found that forskolin appeared to have a negative effect, or at least no positive effect, under this condition (fig. S6D). To simplify, we tested the effect of soluble SCF and concentration of FBS and found that these effects were negligible as well (fig. S6, E and F). Then, using this simplified condition (aRK10 supplemented with FBS, bFGF, CHIR, and IWR1), we further refined the culture condition by testing inhibitors of MAPK-related signaling. As expected, PD0325901, an MEK inhibitor that inhibits FGF signaling, abolished SWR-PGCLC propagation (Fig. 5B). Notably, SB590885, a B-Raf proto-oncogene, serine/threonine kinase (BRAF) inhibitor, was associated with a prominent increase in both the percentage and number of SWR-PGCLCs (Fig. 5B). Other inhibitors for MAPK, such as SB203580 (a p38 inhibitor) and SP600125 (a JNK inhibitor), and upstream kinases such as WH-4-023 (an LCK proto-oncogene, Src family tyrosine kinase (LCK/SRC) inhibitor) did not show such an effect, indicating the specificity of BRAF-mediated signaling. The positive effect of SB590885 was dependent on the presence of m246 feeders (Fig. 5C).

Under the determined condition (aRK10 supplemented with FBS, bFGF, CHIR, IWR1, and SB590885), SWR-PGCLCs continuously proliferated while maintaining their expression of OGBT reporter genes (Fig. 5D). In addition to SWR-PGCLCs, other cell populations negative for the reporter genes or positive for only BT appeared at every cell passaging. Although these cells remain uncharacterized, similar cell populations have been observed during the propagation of human PGCLCs (*37*). Thus, these cell populations could have been derived from dedifferentiation of PGCLCs. Nevertheless, the number of SWR-PGCLCs in culture was biexponentially increased over a month (Fig. 5E and fig. S6G). We noticed that the propagation temporarily slowed down in the middle of the culture period, i.e., at around 24 days, as seen in human PGCLC propagation (*37*). During the long-term culture, SWR-PGCLCs maintained the expression of key PGC genes, such as *POU5F1*, *SOX17*, *PRDM1*, and *NANOS3* (Fig. 5F). PCA and nonhierarchical clustering analyses showed changes in the expression profiles of these genes (Fig. 5G). However, we found no DEGs(>4 times, FDR <0.001, logCPM >4) indicative of late PGCs, such as *DDX4* or *DAZL* (Fig. 5F), suggesting that SWR-PGCLCs in the long-term culture propagate while maintaining the early PGC state, similar to human PGCLCs propagating in culture (*37*). During the long-term culture, SWR-PGCLCs exhibited locomotive activity with filopodium formation (Fig. 5, H and I, and movies S1 and S2), which is a representative characteristic of migratory PGCs after specification. We tried to reaggregate SWR-PGCLCs with mouse embryonic ovarian somatic cells, to see whether further differentiation could be induced as seen in human PGCLCs. However, SWR-PGCLCs never mingled with the mouse somatic cells, perhaps due to the incompatibility of adhesion molecules, consistent with the fact that NWR-iPSCs in mouse embryos hardly mixed (*14*). Collectively, these results showing the potential for long-term proliferation while maintaining the expression of the key PGC genes and the characteristic locomotion activity reinforced our conclusion that SWR-PGCLCs have characteristic phenotypes of PGCs in vivo.

### Induction of PGCLCs from NWR-iPSCs

**Fig. 6.**
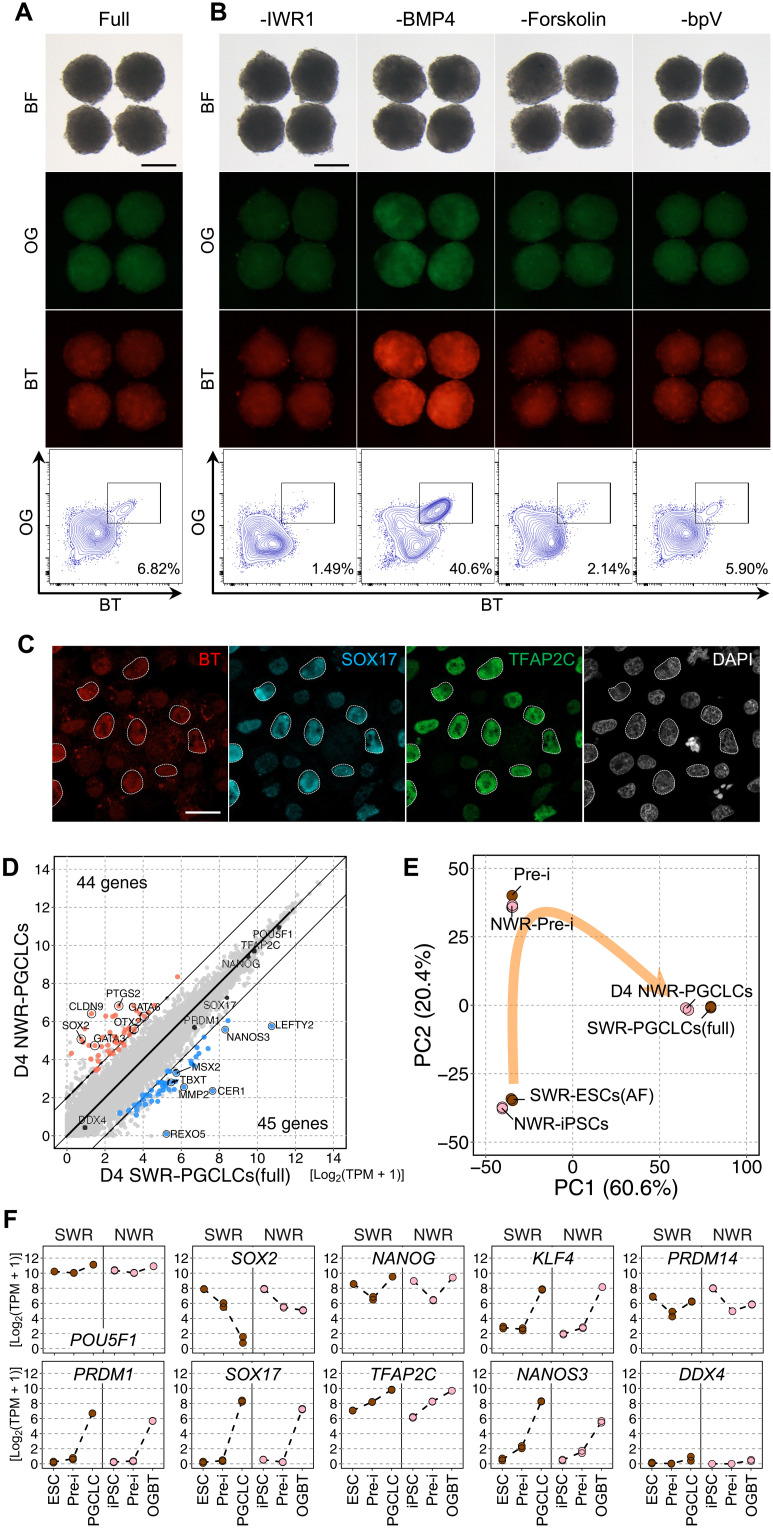
Induction of PGCLCs from OGBT NWR-iPSCs. (**A**) Subtle induction of PGCLCs from OGBT NWR-iPSCs under the aRK10 + BLSEYF2i condition. Shown are images and a FACS plot of PGCLCs at day 4 of induction under condition aRK10 + BLSEYF2i. *n* = 2, biologically independent experiments. Scale bar, 200 μm. (**B**) Optimization of PGCLC induction form NWR-iPSCs. Shown are images and FACS plots of PGCLCs at day 4 of induction without the factor indicated. Note that PGCLCs were efficiently induced under the PGCLC induction condition without BMP4. *n* = 2, biologically independent experiments. Scale bar, 200 μm. (**C**) Coexpression of transcription factors in NWR-PGCLCs. Shown are the results of immunofluorescent analysis of the transcription factors indicated. Note that SOX17 and TFAP2C are coexpressed in BT-positive cells. Scale bar, 20 μm. (**D**) Comparison of transcriptomes of SWR-PGCLCs and NWR-PGCLCs. Shown are scatterplots of the transcriptomes of SWR-PGCLCs and NWR-PGCLCs with DEGs (>4 times, FDR < 0.001, logCPM > 4). (**E**) PCA of the differentiation trajectory from NWR-iPSCs to NWR-PGCLCs. Note that the transcriptomes of derivatives of NWR-iPSCs were similar to the corresponding derivatives of SWR-ESCs. (**F**) Comparison of the expression dynamics of the genes involved in PGC specification between derivatives of NWR-iPSCs and SWR-ESCs. Shown are each value and the averaged values of gene expression based on transcriptome analyses using biologically duplicated samples.

Having established a culture method that efficiently produces SWR-PGCLCs, we next applied the culture condition for PGCLC induction from NWR-iPSCs. The NWR-iPSC line was reprogrammed using skin fibroblasts from Nabire, a female NWR who died in 2015, by transient expression of human *POU5F1*, *SOX2*, *KLF4*, and *C-MYC* encapsulated in the Sendai virus. During the reprogramming, fibroblasts displayed morphological changes characteristic for mesenchymal to epithelial transition within 7 days, and then individual colonies became apparent, which could be isolated from day 15 (fig. S7, A and B). After clearance from Sendai virus–encoded RNA, expression of the pluripotency markers, such as NANOG, SOX2, OCT3/4, TRA-1-60, and SSEA3, and a diploid karyotype (2n = 81 including a marker chromosome as reported previously (*46*)] were confirmed by immunofluorescence and G-banding analyses, respectively (fig. S7, C to E). These iPSCs were capable to differentiate into the three germ layers (fig. S7F). The transcriptomic profile of the NWR-iPSCs was similar, albeit not identical, to that of the SWR-ESCs (fig. S7G), i.e., both cell lines were in a primed state. To closely follow the method established with SWR-ESCs, we inserted the reporter constructs, *OG* and *BT*, into NWR-iPSCs; we named the resulting cells OGBT NWR-iPSCs. However, PGCLC induction from the NWR-iPSCs under the aRK10 + BLSEYF2i condition was much less efficient than that from SWR-ESCs (Fig. 6A). This may have been due to the difference in the pluripotent state and/or propensity for PGC differentiation. On the basis of the refinement for SWR-PGCLC differentiation, the competence was highly dependent on the duration and timing of WNT and BMP signals. Therefore, we removed the component controlling these signals and other components from aRK10 + BLSEYF2i during PGCLC differentiation. Withdrawal of IWR1 and forskolin resulted in a reduction of the OGBT-positive population. Notably, an efficient OGBT-positive cell differentiation, accounting for up to 40% of the aggregated cells, was observed upon withdrawal of BMP4 (Fig. 6B). This effect was supported by the finding that the percentage of OGBT-positive cells was reduced as the concentration of BMP4 was increased (fig. S7H). Withdrawal of bpV did not have a significant impact on OGBT-positive cell differentiation from NWR-iPSCs. These results indicate that the optimal timing and duration of BMP4 signaling is dependent on the iPS clone.

Immunofluorescence analysis revealed that OGBT-positive cells expressed SOX17 and TFAP2C (Fig. 6C). Comparison of key gene expressions in OGBT-positive cells with NWR-iPSCs by qPCR analysis showed constant expression of *POU5F1* and *NANOG*, down-regulation of *SOX2*, up-regulation of early PGC markers, such as *SOX17*, *PRDM1*, *TFAP2C*, and *NANOS3*, and no expression of *DDX4*, and all these features were reminiscent of the gene expression changes during differentiation of PGCLCs from SWR-ESCs (Fig. 2A and fig. S7I). Transcriptome analysis supported the similarity in gene expression profiles between SWR-PGCLCs and NWR-iPSC–derived OGBT-positive cells, as only 89 DEGs (>4 times, FDR < 0.001, logCPM > 4) were detectable (Fig. 6D). On the other hand, the DEGs included *SOX2* and *NANOS3*. The higher expression of *SOX2* and lower expression of *NANOS3* in NWR-iPSC–derived OGBT-positive cells compared to SWR-PGCLCs may have been caused by heterogeneous differentiation and/or slower differentiation of OGBT-positive cells from NWR-iPSCs. Nevertheless, the transcriptome analysis revealed that the trajectory of differentiation from NWR-iPSCs to OGBT-positive cells via the preinduced cells was quite similar to that from SWR-ESCs to SWR-PGCLCs via the preinduced cells (Fig. 6E). The expression dynamics of key genes was almost identical between SWR and NWR derivatives, except in the case of the abovementioned blunt decrease and increase in *SOX2* and *NANOS3* expression, respectively, in NWR-iPSC–derived OGBT-positive cells (Fig. 6F). On the basis of these similarities, we concluded that the NWR-iPSC–derived OGBT-positive cells were NWR-PGCLCs.

### Identification of surface markers for isolation of SWR- and NWR-PGCLCs

**Fig. 7.**
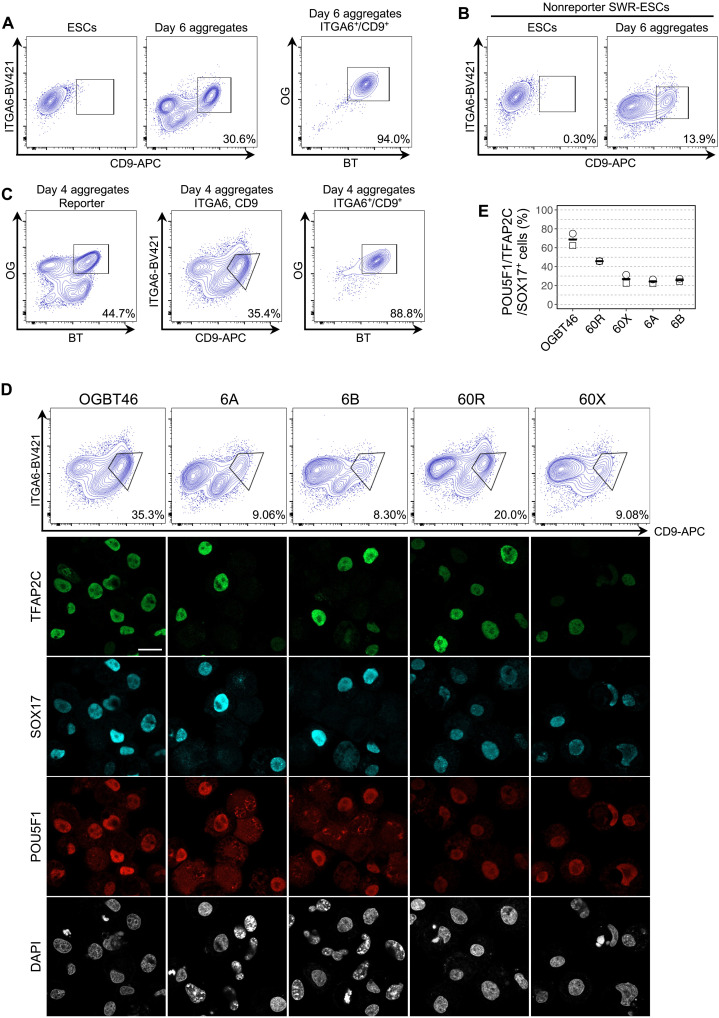
Identification of surface marker proteins for isolation of PGCLCs. (**A**) Specific expression of CD9 and ITGA6 in OGBT SWR-PGCLCs. Shown are FACS plots for CD9 and ITGA6 expression in OGBT SWR-ESCs and their derivatives at day 6 of induction (left). Note that 94% of the gated CD9 and ITGA6 double-positive cells were also OGBT-positive (right). APC;,Allophycocyanin. (**B**) Expression of CD9 and ITGA6 in derivatives from nonreporter SWR-ESCs. Shown are FACS plots for CD9 and ITGA6 expression in nonreporter SWR-ESCs and their derivatives at day 6 of induction. (**C**) Specific expression of CD9 and ITGA6 in OGBT NWR-PGCLCs. Shown are FACS plots of OGBT expression (left) and CD9 and ITGA6 expression (middle) in OGBT NWR-iPSC derivatives at day 4 of induction. Note that 88% of the gated CD9 and ITGA6 double-positive cells were also OGBT-positive (right). (**D**) Induction of CD9 and ITGA6 double-positive cells from nonreporter NWR-iPSCs. Shown are FACS plots and immunofluorescence analysis results for the expression of key transcription factors. 6A, 6B, 60R, and 60X are independent nonreporter NWR-iPSC lines. Note that SOX17-expressing cells also expressed TFAP2C and POU5F1. Scale bar, 20 μm. In addition, note that FACS plots and the percentages of CD9 and ITGA6 double-positive cells were varied among the cell lines. (**E**) Enrichment of NWR-PGCLCs in CD9 and ITGA6 double-positive cells. Shown are the percentages of SOX17, TFAP2C, and POU5F1 triple-positive cells among CD9 and ITGA6 double-positive cells induced from the indicated NWR-iPSCs. All results in this figure are based on experiments using biologically duplicated samples.

With respect to the application of PGCLC derivation to wild animals, especially for species conservation, the requirement of gene editing for the reporter line greatly compromises the value of this technology. To overcome this limitation, we tried to find endogenous surface protein(s) expressed in PGCLCs but not in ESCs and iPSCs. By exploring the transcriptome, we identified candidate proteins that are preferentially expressed in PGCLCs or are used for isolation of PGCLCs in other species (fig. S8A) and then focused on proteins that have homologs for which antibodies are commercially available. Screening of antibodies by immunofluorescence followed by FACS analysis revealed that SWR-ESCs and SWR-PGCLCs could be separated with antibodies against CD9 and ITGA6 (fig. S8B and C). This combination of antibodies successfully isolated PGCLCs: Up to 94% of cells expressing both CD9 and ITGA6 were OGBT-positive SWR-PGCLCs (Fig. 7A). The cell population emerged in nonreporter SWR-ESCs upon PGCLC induction via the preinduction, confirming that these surface proteins can be used as markers for the isolation of PGCLCs (Fig. 7B).

We then tested these surface marker proteins for their ability to isolate NWR-PGCLCs. As seen in SWR-PGCLCs, 88% of NWR cells expressing both CD9 and ITGA6 after PGCLC induction were OGBT-positive cells (Fig. 7C). On the other hand, the separation of the CD9^+^/ ITGA6^+^ double-positive cell population from NWR derivatives was not as clear as that from SWR-ESC derivatives, possibly due to the unexpected differentiation of BT weakly positive cells from OGBT NWR-iPSCs. The differential distribution of the derivatives after PGCLC induction could be attributed to clonal variation in the differentiation propensity of iPSCs. To test the clonal variation, we tested several nonreporter NWR-iPSCs for PGCLC differentiation, followed by cell sorting with CD9 and ITGA6 antibodies. As expected, there was variation in the differentiation of derivatives expressing these surface proteins (Fig. 7D). To evaluate the enrichment of PGCLCs in the CD9- and ITGA6-positive cells, we sorted the double-positive population and then examined the expression of TFAP2C, SOX17, and POU Class 5 Homeobox 1 (POU5F1). The enrichment of cells expressing all these marker proteins was around 70% from the OGBT reporter line and 47% from 60R, a nonreporter line (Fig. 7E). The enrichments from other nonreporter lines, 60X, 6A, and 6B, were around 25% (Fig. 7E). On the basis of these enrichment analyses, 60R was the most suitable NWR-iPSC clone for PGCLC induction among the iPSC clones tested, although the efficiency was less than that in the OGBT reporter line. A possible reason for the lower rate of enrichment in the non-reporter lines was that the culture condition for PGCLCs induction was optimized using the OGBT reporter line. To maximize the efficiency, the condition needs to be fine-tuned for each iPS clone. This clonal variation in the potential of PGCLC differentiation was even observed in humans and monkey pluripotent stem cells (*23*, *30*). Nevertheless, we succeeded in enriching PGCLCs from nonreporter NWR-iPSCs, which provided a sufficient founder population of germ cells for the reconstitution of further differentiation processes toward mature gametes.

## DISCUSSION

In this study, we developed a culture system to reconstitute PGC specification using pluripotent stem cells in large mammals. While there are many large mammals for which embryos are readily available, including domestic animals such as cows and pigs, it was unexpected that the reconstruction could be achieved in white rhinoceroses, a near-threatened species. This accomplishment relied on the establishment of SWR-ESCs (*10*), which are reliable materials for determining the condition of PGCLC differentiation. As in mice and humans, the signals essential for PGCLC differentiation from SWR-ESCs were BMP and WNT, indicating that the signaling cascades are conserved among a broad range of mammalian species. On the other hand, the optimal timing and duration of these signaling cascades were highly dependent on the species and even the cell line. For example, it was critical to interrupt WNT signaling at the beginning of PGCLC induction for a robust induction of SWR-PGCLCs. We revealed that mouse PGCLC induction was enhanced by WNT inhibition upon day 2 of PGCLC induction but was abrogated by WNT inhibition at the beginning of PGCLC induction (fig. S9). This species difference was likely due to a difference in the timing of the lineage commitment to the nascent mesoderm: Mesodermal genes were already up-regulated at the preinduction stage in SWR (Fig. 2A) but were up-regulated at day 2 of PGCLC induction in mice (*16*). Thus, the optimal timing for WNT inhibition is, in principle, at the onset of mesoderm differentiation. This interpretation is consistent with the notion that PGC differentiation requires transient expression of genes downstream of WNT in mice and primates, while mesoderm and endoderm differentiation requires continuous expression of WNT downstream genes (*25*). Similar to the WNT inhibition, interruption of BMP4 signaling had a positive effect on NWR-PGCLC differentiation (Fig. 6B and fig. S7H). This is consistent with the evidence that BMP signaling is required for initial differentiation of PGCs but is no longer activated at a later stage (*47*). These results indicate that the timing and duration of WNT and BMP signaling should be fine-tuned for each individual species and cell line according to the propensity of the pluripotent state to differentiate into mesoderm.

In contrast to the conserved role of the cytokines, the key transcription factors may differ among mammalian species. For example, the transcription factor *SOX17* is important for PGC specification in humans, cynomolgus monkeys, rabbits, and pigs, but not in mice and rats (*21*–*25*, *48*), while *PRDM14* and *SOX2* are critical in rodents but not in humans. This indicates that the common mammalian ancestor might have a *SOX17*-dependent manner of PGC specification, whereas rodents might acquire a unique manner of PGC specification during evolution. Our study demonstrated that *SOX17* plays an essential role in SWR-PGCLC differentiation, thus suggesting that rhinoceros PGCs are specified in a manner that is replicated in the majority of model mammalian species. This distinct gene regulatory network for PGC specification might correlate with the morphology of the postimplantation epiblast, which forms an egg cylinder structure in mice and bilaminar structure in humans and cynomolgus monkeys. Although the precise morphology of the postimplantation epiblast in SWR or NWR is not known, other ungulate species, such as horses, cows, and pigs, show a unique embryogenesis during implantation. In these species, the blastocysts form three layers—a hypoblast, epiblast, and polar trophectoderm—in the manner of mouse and human blastocysts, but, eventually, the polar trophectoderm, known as Rauber’s layer, disappears, and then the remaining two layers form a bilaminar structure lacking the polar trophectoderm and amnion (*49*). With this study, it becomes possible to recapitulate the PGC specification process from pluripotent cells in the three major types of embryogenesis, thus providing a platform to address whether the gene regulatory networks for PGC specification are associated with the structural features of early embryogenesis.

Through repeated refinement, we produced PGCLCs from NWR-iPSCs under a defined condition. Because PGCs are the founder population for gametes, this study paves a way to produce functional gametes from NWR-iPSCs, which will contribute to the effort to rewind the extinction in the NWR. To this end, as shown in the mouse model, the differentiation of mature gametes essentially requires a gonadal somatic cell environment that provides signals for sex-dependent differentiation and functional maturity of the gametes. Such an environment could be reconstituted with either embryonic gonadal somatic cells or their equivalents induced from pluripotent stem cells. Our recent studies in the mouse model showed that fully potent gonadal somatic cells could be induced from mouse ESCs (*20*). In the reconstituted environment, i.e., the ovarian organoid, mouse ESC-derived PGCLCs recapitulated a series of oogenesis processes and lastly differentiated into functional oocytes, which fertilized and gave rise to pups (*20*). These previous findings suggest that, in theory, the differentiation of gonadal somatic cells from NWR-iPSCs would make it feasible to produce mature NWR oocytes suitable for fertilization with banked NWR sperm derived from four deceased males stored in liquid nitrogen. However, even if gonadal somatic cells could be induced from NWR-iPSCs, there would be several obstacles to overcome, such as species differences and the duration of gametogenesis. Recently, production of PGCLCs and gametes in culture have been attempted in numerous mammalian species (*15*). This trend will provide clues to overcome these obstacles in the future.

## MATERIALS AND METHODS

### SWR-ESC and NWR-iPSC culture

SWR-ESCs (051B) and NWR-iPSCs (60R, 60X, 6A, and 6B) were maintained in DK20 [DMEM/F12 (Gibco, #11330033) supplemented with 20% (v/v) KSR (Gibco, #10828028), 1× minimum essential medium (MEM) nonessential amino acid (NEAA) (Gibco, #11140050), 1× GlutaMAX (Gibco, #35050061), 0.1 mM 2-mercaptoethanol (Gibco, #21985023), penicillin/streptomycin (50 U/ml; Gibco, 1570063), recombinant human bFGF (10 ng/ml ; Wako, #064-04541), and activin A (20 ng/ml; PeproTech, #120-14)] except in the case of the experiments shown in Figs. 1 to 3 and figs. S1 to S4 in which SWR-ESCs were cultured without activin A. SWR-ESCs and NWR-iPSCs were passaged around every 4 or 5 days. For passage, cells were dissociated by incubation with TrypLE (Gibco, #12604021) for 4 min, centrifuged, and then plated at a density of 2 × 10^5^ cells on one well of a six-well plate overlaid with 2.0 × 10^5^ mitomycin C–treated(MMC) MEFs. Rho-associated kinase (ROCK) inhibitor (Y-27632, Wako, #03424024) was added to medium at 10 μM, and then the cells were allowed to culture for 24 hours after the passage.

### Generation of NWR-iPSCs

Fibroblasts from the NWR female Nabire were isolated as described (*14*) and maintained in skin medium [DMEM, high glucose (Gibco, #41965039), and GlutaMAX (Gibco, #31966021) supplemented with 10% FBS (Gibco, #16140071), 1× MEM NEAA (Gibco, #11140035), and FGF (10 ng/ml; PeproTech, #AF-100-18B)] on plates coated with attachment factor (Gibco, #S006100). For reprogramming, we used a similar approach as reported in Korody *et al.* (*13*). Briefly, 2 days before transduction (day −2), fibroblasts were detached by incubation with TrypLE Select Enzyme (Gibco, #12563011) for 8 min at 37°C, and 2.4 × 10^4^ cells were plated each in 3 × 12 wells coated with Geltrex (Gibco, #A1569601) in skin medium. On the next day, medium was changed. On the day of transduction (day 0), cells of 1 × 12 wells were detached with TrypLE Select Enzyme and counted (~9.0 × 10^4^ cells). On the basis of the obtained cell number, the amount of Sendai viruses (CytoTune–iPS 2.0 Sendai Reprogramming Kit, Thermo Fisher Scientific, #A16517, lot #L2160042) needed for transduction of cells in 1 × 12 wells with an multiplicity of infection of 10:10:6 (KOS:c-Myc:Klf4) was calculated. The medium of 2 × 12 wells was changed to skin medium containing polybrene (10 μg/ml; Merck, #TR-1003-G). Sendai virus mixture was added to 1 × 12 wells. The other 1 × 12 wells were not transduced and served as negative control. The plate was sealed with parafilm and centrifuged at room temperature and 800*g*. After 20-min centrifugation, the plate was turned and centrifuged for another 10 min. Subsequently, parafilm was removed, and the plate was incubated overnight at 37°C and 5% CO_2_. On the next day (day 1), cells were washed once with skin medium and subsequently fed with skin medium. The skin medium was changed on days 2, 4, and 6. On day 7, transduced cells were carefully detached by incubation with TrypLE Select Enzyme for 2 to 3 min at 37°C, and 5.0 × 10^3^ to 20.0 × 10^3^ cells were plated per six wells [coated with Geltrex and laid with 2.0 × 10^5^ mitomycin C–treated MEFs (tebu-bio, #MEF-MITC)] in skin medium. On the next day (day 8), the medium was changed to KB medium [39% DMEM, high glucose (Gibco, #41965039), 39% complete Fibroblast Growth Medium (FGM) (Lonza, #CC3132), 20% KSR (Gibco, #10828028), 1× NEAA, 1× GlutaMAX (Gibco, #35050061), 0.1 mM 2-mercaptoethanol (Gibco, #21985023), and FGF (12 ng/ml)]. From now on, KB medium was changed daily. On days 15 and 19, single colonies were transferred to individual 24 wells coated with Geltrex and laid with 5.0 × 10^4^ MEFs. KB medium was supplemented with 10 μM ROCK inhibitor (Y-27632, Selleck Chemicals, #SEL-S1049-10MM) at the day of picking and on the day after. Successfully growing clones were transferred to 12 wells coated with Geltrex and laid with 1.0 × 10^5^ MEFs using phosphate-buffered saline (PBS) and 0.5 mM EDTA solution (PBS/EDTA; Gibco, #14190-250, and Thermo Fisher Scientific, #15575-020) for 8 min at 37°C. After split, KB medium was supplemented with 10 μM of Y-27632 for 24 hours. Clones exhibiting nice morphology were further expanded and cryopreserved in KSR, 10% dimethyl sulfoxide (Sigma-Aldrich, #D22660).

To generate feeder-free cultures for clearance of Sendai virus–encoded RNA, iPSCs were kept in normoxia, and the medium was changed to KB:mTeSR1 medium [50% KB medium and 50% mTeSR1 (STEMCELL Technologies, #05850)]. When reaching confluency, cells were split with TrypLE Select Enzyme on Geltrex-coated wells without MEFs. Y-27632 was added at 10 μM for 24 hours. Subsequent splits were performed with PBS/EDTA (~4 min at 37°C) and without adding Y-27632.

### Clearance of Sendai virus–encoded RNA

To generate vector-free NWR-iPSC lines, clones were expanded until passage 7 (clone 6) or passage 5 (clone 60) and then split with PBS/EDTA (incubation 6 min at 37°C) in 1:100 and 1:200 ratios to favor formation of individual colonies. Y-27632 was added at 5 μM for 24 hours after split. When single colonies grew to an appropriate size, they were picked into 24 wells coated with Geltrex and laid with 0.5 × 10^5^ MEFs. Subclones of clone 6 and clone 60 were expanded, and RNA was collected at passage 11 and passage 9, respectively. Reverse transcription PCR (RT-PCR) was performed according to the manufacturer’s instructions (CytoTune–iPS 2.0 Sendai Reprogramming Kit) and using the primer pairs shown in table S2. NWR-iPSC lines 6A and 6B were vector-free at passage 11, and master cell banks were generated. In all subclones of clone 60, Sendai virus–encoded RNA was detected. Subcloning of one subclone was not sufficient to obtain vector-free iPSC lines. Therefore, we shifted the maintenance culture of clone 60 at passage 11 to 38°C for 5 days. Cells were split, and the maintenance culture (passage 12) was incubated for additional 5 days at 39°C. After the next split (passage 13), the maintenance culture was shifted back to 37°C. After RT-PCR of clone 60 at passage 14, weak bands resulting from Sendai virus–encoded RNA were detected. After another round of subcloning of clone 60, we obtained the lines 60R and 60X, which were cleared of Sendai virus RNA at passage 19.

### G banding of NWR-iPSCs

NWR-iPSCs on MEFs were split with PBS/EDTA (>6 min at 37°C), and 7.3 × 10^5^ cells were plated in MEF-conditioned KB medium supplemented with 10 μM of Y-27632 on two Geltrex-coated T25 flasks each. The conditioned medium was generated by incubating MEFs overnight with KB medium. 2-Mercaptoethanol and FGF were added freshly before using the medium on NWR-iPSCs. MEF-conditioned KB medium supplemented with 10 μM Y-27632 was changed daily. When cells reached approximately 50% confluency, they were treated with colcemide (0.1 g/ml) for 2.5 hours at 37°C. Subsequently, cells were detached using trypsin-EDTA (Biochrom AG, #L6221). Enzymatic digestion was stopped by adding medium containing 10% KSR. Cells from both T25 flasks were pooled in one 50-ml Falcon tube. After centrifugation at room temperature for 5 to 10 min at 450*g*, the supernatant was discarded except for ~1 ml, which was used to resuspend the cell pellet and to transfer it into a 15-ml Falcon tube. For hypotonic treatment, 10 ml of 0.075 M KCl solution (Merck, #1049360500) was added, and cells were incubated for 20 min at 37°C. Subsequently, ~1 ml of freshly prepared, ice-cold fixative (96% methanol/acetic acid mix, ratio of 3:1, Merck, #1060092500 and #1000632500, respectively) was added, and cells were carefully mixed by inversion. After centrifugation at room temperature for 5 to 10 min at 450*g*, the supernatant was discarded except for ~1 ml, which was used to resuspend the cell pellet. To wash the cells, 5 to 10 ml of fixative was added carefully in several steps including mixing, and cells were centrifuged at room temperature for 10 min at 450*g*. The supernatant was discarded except for ~1 ml, which was used to resuspend the cell pellet. The washing step was repeated three times. Last, the cells were resuspended in 2 ml of fixative and transferred into a 1.5-ml Eppendorf tube. The tube was sealed with parafilm and sent to the institute for human genetics in Jena, Germany. There, Giemsa-trypsin-Giemsa banding technique was used. Metaphase chromosomes were imaged with the Zeiss Axio Z2 microscope using the scanning system Metafer and the Ikaros V5.1 software (both Metasystems). The average resolution was ~200 bands per haploid chromosome set. In total, 20 metaphase spreads were analyzed per cell line.

### Generation of the OGBT SWR-ESCs and NWR-iPSCs

For construction of the donor vectors to generate *POU5F1-p2A-EGFP* or *BLIMP1-p2A tdTomato* knock-in SWR-ESC lines, the homology arms of *POU5F1* and *BLIMP1* were amplified by PCR using the primer pairs listed in table S2. Amplified PCR products were inserted using an In-Fusion system (TaKaRa, #Z9649N) to each vector linearized by appropriate restriction enzymes. The backbone vectors for the knock-in were provided by Saitou and co-workers (*24*). To construct vectors for the expression of gRNAs, oligonucleotides encoding the gRNAs were inserted into pX330-U6-Chimeric_BB-CBh-hSpCas9 digested by Bbs I [New England Biolabs (NEB), #R3539]. gRNA sequences were chosen using CRISPRdirect (https://crispr.dbcls.jp).

For generation of the OGBT reporter lines, 1.5 μg of the donor vector and 1.5 μg of the gRNA vector were introduced into the 6 × 10^5^ cells of SWR-ESCs (051B) or NWR-iPSCs (60R) with Lipofectamine 2000 (Invitrogen, #11668019). At 24 hours after the lipofection, the culture medium was exchanged with fresh medium. At 48 hours after the lipofection, puromycin (1 μg/ml) or G418 (400 μg/ml) was added to the medium. At 2 to 3 days after the drug selection, the SWR-ESCs or the NWR-iPSCs were passaged to a new culture plate coated with MEFs. The resultant SWR-ESCs and NWR-iPSCs were subjected to subsequent lipofection of *Cre-EGFP* vector to remove the drug resistance gene. At 24 hours after the lipofection, the medium was exchanged with fresh medium. At 48 hours after the lipofection, enhanced green fluorescent protein (EGFP)–positive cells were sorted by FACSAria Fusion (BD Biosciences), and 5000 sorted cells were seeded onto a well of the six-well plate coated with MEFs. After 5 to 6 days of culture, colonies were picked up and cultured in a 96-well plate for genotyping. Cre-mediated deletion of the drug resistance gene was determined by PCR using the primer pairs shown in table S2.

### Generation of SWR-ESCs constitutively expressing mCherry

For the generation of SWR-ESCs constitutively expressing mCherry, 1.5 μg of CAG-mCherry-IRES-Neo flanked by PiggyBac terminal repeat sequences and 1.5 μg of PiggyBac transposase expression vector were transfected into SWR-ESCs (051B) with Lipofectamine 2000, and then the cells were cultured with G418 (400 μg/ml). At 4 days of culture, cells expressing mCherry at a similar level were sorted by FACSAria Fusion. The sorted SWR-ESCs were used as CAG-mCherry SWR-ESCs.

### The preinduction and PGCLC differentiation from SWR-ESCs and NWR-iPSCs

For the preinduction,4.0 × 10^5^ SWR-ESCs were plated on a well of six-well plate coated with fibronectin (Millipore, #FC010) and then cultured for 24 hours in GK15 [GMEM (Gibco, #11710035), 15% (v/v) KSR, 1× NEAA, 1× GlutaMAX, 1 mM sodium pyruvate (Gibco, #11360070), 0.1 mM 2-mercaptoethanol, and penicillin/streptomycin (50 U/ml)] or aRK10 [advanced RPMI 1640 (Gibco, #12633012), 10% (v/v) KSR, 1× NEAA, 1× GlutaMAX, and penicillin/streptomycin (50 U/ml)] medium supplemented with BMP4 (10 ng/ml; R&D Systems, #314-BP), 6 μM CHIR (Wako, #034-23103), and 10 μM of Y-27632 (Wako, #03424024). Then, preinduced cells were dissociated with TrypLE for 4 min, centrifuged, and plated into a well of 96-well U-bottom ultralow attachment microplate (Corning, #7007) at a concentration of 6000 cells per well in GK15 or aRK10 supplemented with LIF (10 ng/ml; Wako, #125-05603), SCF (100 ng/ml; R&D Systems, #455-MC), EGF (50 ng/ml; R&D Systems, #2028-EG), and 10 μM of Y-27632 and with or without BMP4 (200 ng/ml). The images of the aggregates were captured by a stereo microscope (Olympus, SZX16). The following inhibitors and cytokines were added to the culture medium on the appropriate day of culture as indicated in the description of each experiment: 2.5 μM endo-IWR1 (Tocris, #3532), 10 μM forskolin (Merck, #344270), 10 μM bpV(HOpic) (Sigma-Aldrich, #SML0884), 2.5 μM XAV939 (Sigma-Aldrich, #X3004), 2.5 μM IWP-2 (Wako, #034-24301), activin A (50 ng/ml; PeproTech, #120-14), and 1 μM PD0325901 (Stemgent, #04-0006).

### Generation of the SOX17-knockout lines and rescue lines

For construction of the *SOX17*-knockout vector, the t2A-puromycin resistance gene in pX459 was replaced with p2A-*EGFP*, yielding the pX459-p2A-*EGFP* vector. Oligos encoding gRNAs targeting *SOX17* were inserted into the pX459-p2A-*EGFP* vector. Two gRNA vectors were constructed for a large deletion in *SOX17* loci. Transfection was performed in the same way as for the generation of the OGBT reporter lines. At 48 hours after lipofection, EGFP-positive cells were sorted with a FACSAria Fusion, and 5000 sorted cells were seeded onto a well of the six-well plate coated with MEFs. After 4 to 6 days of culture, colonies were picked up and expanded for the genotyping. Genotypes were determined by PCR using the primer pairs in table S2.

For exogenous expression of *SOX17*, the coding sequences (CDSs) of rhinoceros *SOX17* were amplified from cDNA of SWR-PGCLCs using KOD One DNA polymerase (TOYOBO, #KMM-201) with the primer pair listed in table S2. The Kozak sequence, GCCACC, was added within 5′ of the first ATG during the PCR reaction. Rhinoceros *SOX17* amplified with the Kozak sequences was inserted into the PiggyBac-based Dox-inducible expression vector using the In-Fusion system. The rhinoceros *SOX17* expression vector and the rtTA vectors were transfected into cells of the *SOX17*-knockout line along with the PiggyBac transposase expression vector by Lipofectamine 2000. The drug selection by G418, pickup of colonies, and genotyping were performed in the same way as for the generation of OGBT reporter lines.

### FACS sorting of SWR-PGCLCs and NWR-PGCLCs

The floating aggregates containing SWR-PGCLCs or NWR-PGCLCs were incubated in CTK solution (0.1 mg/ml collagenase type IV, 0.25% trypsin, 1 mM CaCl2 and 20% KSR) solution for 30 min at 37°C. After removal of CTK solution, the aggregates were dissociated by incubation in Accutase (Nakarai, #12679-54) for 10 min at 37°C. The cell suspension was filtered using a cell strainer (70 μm) to remove cell clumps. The collected cells were suspended in FACS buffer [PBS containing 0.1% bovine serum albumin (BSA)] and analyzed or sorted with a FACSAria Fusion.

For the analysis/sorting of SWR-PGCLCs or NWR-PGCLCs with the cell surface markers, the floating aggregates were dissociated by incubation in CTK solution, followed by Accutase as described above, and then stained with the fluorescence-conjugated antibodies listed in table S3 for 15 min on ice. After washing the cells with FACS buffer, the cell suspension was filtered by a cell strainer and analyzed or sorted with a FACSAria Fusion.

### Establishment of m246 feeder cells

For construction of the rhinoceros *KITLG* expression vector, the CDSs of rhinoceros *KITLG* were amplified from cDNA of SWR-ESCs using a KOD One DNA polymerase with the primer pair listed in table S2. The Kozak sequence, GCCACC, was appended to the primer and was added within 5′ of the first ATG during the PCR reaction. The rhinoceros *KITLG* amplified with the Kozak sequence was inserted between the CAG promoter and IRES-puromycin resistance gene using the In-Fusion system. A total of 1.5 μg of the CAG-rhinoceros *KITLG-*IRES-puromycin resistance gene flanked by PiggyBac terminal repeat sequences and 1.5 μg of the PiggyBac transposase expression vector were transfected into Sl/Sl4 cells by Lipofectamine 2000, and then the cells were culture with puromycin (1 μg/ml) for 4 days. Then, single cells were sorted into individual wells in a 96-well plate by FACSAria Fusion. After 10 days of culture, individual cell clones were duplicated and then subjected to Mitomycin C (MMC) test following the method described in the previous report (*38*). MMC-resistant clones were subjected to genotyping for quantification of the integration of rhinoceros *KITLG* by qPCR analysis using the primers listed in table S2. Cells of a clone harboring a high copy number of the rhinoceros *KITLG* expression vector were used as m246 feeder cells.

### Expansion culture of SWR-PGCLCs

m246 feeder cells were treated with MMC (5 μg/ml) for 2 hours, washed with PBS, and then cultured with STO [DMEM, high glucose (Nacalai, #08458-45), 10% (v/v) FBS(Gibco, #10437028), 2 mM l-glutamine, and penicillin/streptomycin (50 U/ml)] medium for 5 hours. MMC-treated m246 cells were stored at −80°C. At 6 hours before sorting of SWR-PGCLCs, MMC-treated m246 feeder cells were seeded at 1 × 10^5^ or 2 × 10^5^ cells per well of a 24- or 12-well plate, respectively. SWR-PGCLCs at day 6 of induction were sorted and seeded at 5 × 10^3^ or 1 × 10^4^ cells per well of a 24- or 12-well plate, respectively, with expansion medium [advanced RPMI 1640, 10% (v/v) KSR, 2.5% (v/v) FBS, 1× NEAA, 1× GlutaMAX, and penicillin/streptomycin (50 U/ml)] supplemented with bFGF (20 ng/ml), 6 μM CHIR, 2.5 μM endo-IWR1, and 0.5 μM SB590885 (R&D Systems, #2650). The medium was then supplemented with 10 μM of Y-27632 for the first 48 hours of culture. The medium was changed every 2 days with the addition of 1 × 10^5^ or 2 × 10^5^ of MMC-treated m246 feeder cells to the well of the 24- or 12-well plate, respectively.

### Live-cell imaging analysis of expanded SWR-PGCLCs

To observe cell migration, SWR-PGCLCs expressing OGBT reporter genes and SWR-ESCs expressing mCherry were cultured on MMC-treated m246 and MEF feeder cells, respectively. Live-cell imaging was performed with an ApoTome.2 (Zeiss), taking images at 10-min intervals for 12 hours in total, and the data were analyzed using ImageJ (version: 2.1.0/1.53c) with Fiji. The cell tracing was performed using TrackMate, plugins of Fiji.

### Evaluation of differentiation potential of NWR-iPSCs into three germ layers

Endoderm was differentiated from NWR-iPSCs using the StemMACS Trilineage EndoDiff medium (Miltenyi Biotec, #30-115-659) following the manufacturer’s instructions. Briefly, feeder-free NWR-iPSCs were split with TrypLE select enzyme, and 2.5 × 10^5^ cells were plated per Geltrex-coated 12 wells in KB:mTeSR1 medium containing 5 μM Y-27632 (day 0). On day 1, 1 ml of medium without additional Y-27632 was added. On day 2, medium was changed to EndoDiff medium. On days 3 to 6, EndoDiff medium was changed daily. On day 7, cells were washed twice in PBS^+/+^ (PBS, calcium, and magnesium; Gibco, #1404014) and subsequently fixed for 15 min at room temperature in BD Cytofix solution (BD Biosciences, #554655). After washing twice in PBS^+/+^, fixed cells were stored at 4°C until further use.

To represent the mesoderm, NWR-iPSCs were differentiated into beating cardiomyoctes using two different protocols: In the first approach, feeder-free NWR-iPSCs were split with TrypLE Select Enzyme, and 1.0 × 10^5^ cells were plated per Geltrex-coated 12 wells in KB:mTeSR1 medium containing 10 μM Y-27632 (~day −3). KB:mTeSR1 medium was changed daily until cells reached ~90% confluency. On day 0, mesoderm was induced by changing the medium to RPMI 1640 medium (1 ml per 12 wells; Gibco, #11875093) supplemented with B-27 minus insulin (Gibco, #A1895601) and 6 μM CHIR 99021 (Bio-Techne Sales Corp., #4423/10). On day 1, 2 ml of RPMI B-27 minus insulin medium was added per well (no extra CHIR 99021). On day 3, medium was changed to 2 ml of RPMI B-27 minus insulin supplemented with 5 μM IWR-1 (Biozol, #SEL-S7086). On day 5, 2 ml of RPMI B-27 minus insulin medium was added per well (no extra IWR-1). On day 7, medium was changed to RPMI B-27 medium [1.5 ml per well; RPMI 1640 medium supplemented with B-27 (Gibco, #17504044)]. From now on, medium was changed every other day. To remove noncardiomyocytes, cells were cultivated in starvation medium [RPMI 1640 medium, no glucose (Gibco, # 11879020), recombinant human albumin (500 μg/ml; Sigma-Aldrich, #A0237), l-ascorbic acid 2-phosphate (213 μg/ml; Sigma-Aldrich, #A8960), and 5.3 mM sodium dl-lactate (Sigma-Aldrich, #L7900)] for 2 to 4 days. Beating cardiomyocytes were observed after 12 days of differentiation.

The second approach was modified from Burridge *et al.* (*50*). Briefly, feeder-free NWR-iPSCs were plated as described above. When cells reached ~90% confluency, medium was changed to RPMI CDM3 [RPMI 1640 medium with recombinant human albumin (500 μg/ml) and l-ascorbic acid 2-phosphate (213 μg/ml)] supplemented with 5 μM CHIR (day 0). On day 2, the medium was changed to RPMI CDM3 containing 2 mM Wnt-C59 (Selleck Chemicals, #SEL-S7037-5). On day 3, the medium was changed to RPMI B-27 medium, which was replaced every other day. To remove noncardiomyocytes, cells were cultivated in starvation medium for 2 to 4 days. Beating cardiomyocytes were observed after 15 days of differentiation.

The neural (ectodermal) differentiation of NWR-iPSCs was induced by a modified version of the dual SMAD inhibition protocol published by Chambers *et al.* (*51*). Briefly, NWR-iPSCs on MEFs were split with PBS/EDTA, and 1.25 × 10^5^ cells were seeded without feeder per Geltrex-coated 24-well plate in MEF-conditioned KB medium supplemented with 10 μM Y-27632. On the next day, medium was changed to conditioned KB medium without Y-27632. The day after (day 0), medium was changed to neural induction medium [NIM; DMEM/F12 (Gibco, #12660012), 1× B-27, 1× N-2 (Gibco, #17502048), 10 μM SB431542 (Reagents Direct, #21-A94), and 2 μM dorsomorphin (BioVision, #1686-5)]. NIM was exchanged daily for five consecutive days. On day 6, cells were washed once with PBS and subsequently detached using Accutase (Gibco, #A1110501). Enzymatic reaction was stopped after 8 min by adding neural expansion medium [NEM; 0.5× advanced DMEM/F12 (Gibco, #12634010), 0.5× Neurobasal medium (Gibco, #21103049), and 2× N-2 (Gibco, #17502048)] supplemented with 5 μM Y-27632. Cells were passed through a 70-μm strainer (Miltenyi Biotec, #130-041-407) and subsequently centrifuged at 300*g* for 3 min at room temperature. The supernatant was discarded, cells were resuspended in NEM supplemented with 5 μM Y-27632 and counted, and 1.5 × 10^5^ cells were plated per Geltrex-coated 24 wells. Medium was changed every day with NEM. On day 13, cells were fixed as described previously.

### Immunofluorescence analysis

For immunofluorescence analysis, the day 4 aggregates were dissociated as described above, and then OGBT-positive cells and BT-negative cells were separately sorted by a FACSAria Fusion. The sorted OGBT-positive cells and BT-negative cells were mixed at a ratio of 1:1 and spread onto MAS-coated glass slides (Matsunami, #SMAS-04). The slides were fixed in 4% paraformaldehyde in PBS for 15 min at room temperature, washed three times with PBS, permeabilized with 0.5% Triton X-100 in PBS for 10 min at room temperature, and washed three times with PBS. The slides were then incubated in a blocking solution (1% BSA and 0.2% Tween 20 in PBS) overnight at 4°C. After blocking, the slides were incubated with primary antibodies in blocking solution overnight at 4°C. After washing six times with PBS, the slides were incubated with secondary antibodies in blocking solution containing 4′,6-diamidino-2-phenylindole (DAPI; 1 μg/ml) for 1 hour at room temperature. The slides were then washed six times with PBS and mounted in PermaFluor Aqueous Mounting Medium (Thermo Fisher Scientific, #TA-030-FM). For the immunofluorescence analysis of 5mC and 5hmC, after fixation in 4% paraformaldehyde in PBS for 15 min at room temperature, the slides were treated with 4 and 2 N HCl, respectively, in PBS containing 0.1% triton X-100 for 10 min at room temperature. Then, the slides were incubated in 10 mM tris-HCl (pH 8) for 10 min at room temperature, washed twice with PBS, and then subjected to incubation in the blocking solution, followed by staining procedures with antibodies as described above. All antibodies used in this study are listed in table S3. Images were captured and processed by an LSM 900 confocal microscope (Zeiss).

To stain NWR-iPSCs in pluripotent state or after differentiation into cells of the three germ layers, cells were washed twice in PBS^+/+^ (Gibco, #1404014) and subsequently fixed for 15 min at room temperature in BD Cytofix solution (BD Biosciences, #554655). After washing twice in PBS^+/+^, fixed cells were either stored at 4°C until further use or processed directly. To reduce background signal, cells were blocked for 1 hour shaking at room temperature in 1× PBS^+/+^, 5% normal goat serum (NGS; Abcam, #ab7481) for surface markers, or for intracellular and nuclear markers in blocking buffer [PBS^+/+^, 0.2% BSA (Biomol, #1400100), 0.3% Triton X-100 (Sigma-Aldrich, #T8787), 10× NGS, or normal donkey serum (Abcam, #ab7475)]. Afterward, primary antibodies were diluted as indicated below in 1× PBS^+/+^, 1% BSA for surface markers, or blocking buffer and incubated overnight shaking at 4°C. On the next day, cells were washed three times for 15 min in PBS^+/+^. Secondary antibodies were diluted in PBS^+/+^ containing DAPI (NucBlue Fixed Cell Stain Ready Probe, Invitrogen, #R37606), and cells were incubated in the solution for ~2 hours, shaking at room temperature. Subsequently, cells were washed three times in PBS^+/+^. Images were captured and processed with a Leica DMi8 microscope and the LASX software (including the Thunder computational clearing method).

### qPCR analysis

Total RNA was extracted from 1 × 10^4^ cells of each cell type using the RNeasy Micro Kit (QIAGEN, #74004), and cDNA was synthesized using the PrimeScript First-Strand cDNA Synthesis Kit (TaKaRa, #6110B). The qPCR reaction using Power SYBR Green PCR Master Mix (Life Technologies, #4368708) was performed by a CFX384 real-time qPCR system (Bio-Rad). Primers used for the qPCR reaction are listed in table S2. The gene expression levels were examined by calculating Δ*C*_t_ (in log_2_ scale) normalized to the average Δ*C*_t_ values of *ARBP* and *PPIA*. Error bars are the means ± SE from three independent experiments.

### RNA-seq analysis

Total RNAs were isolated from 1 × 10^4^ cells of each cell type using the RNeasy Micro Kit. Purified RNAs were subjected to library construction using a NEB Next Ultra II Directional RNA Library Prep Kit for Illumina (NEB, #E7760L). cDNAs were amplified by 12 cycles of PCR. Library qualities and concentrations were validated using an Agilent 2100 Bioanalyzer(Agilent) with a high-sensitivity DNA kit (Agilent, #5067-4626). Sequencing of the libraries was performed with NextSeq (Illumina).

### Mapping reads of RNA-seq and conversion to gene expression levels

For mapping of the sequenced fragments, the genome sequence (CerSimSim1.0/cerSim1 for SWR, GRCh38/hg38 for humans, and GRCm38/mm10 for mice) and the transcript annotation (cerSim1.ncbiRefSeq for rhinoceros, hg38.ncbiRefSeq for humans, and mm10.ncbiRefSeq for mice) were obtained from the University of California, Santa Cruz (UCSC). All reads were trimmed with Fastp, and untrimmed and trimmed reads with less than 25 base pairs were eliminated. Treated reads were mapped to the genome of each species with STAR and annotated with RNA-Seq by Expectation-Maximization (RSEM). Paired-end data (*52*, *53*) were analyzed using single reads. Sequence data of NWR cells were mapped to the SWR genome sequence. The mapping rate was comparable to the SWR mapping rate. SWR data were mapped uniquely at 93.92 to 96.54%, and NWR data were mapped uniquely at 94.38 to 94.97%.

Data analysis was performed using R software version 4.0.3. We defined all expressed genes as the genes whose log_2_[Transcripts Per Kilobase Million (TPM) + 1] values were >2 in at least one sample. DEGs were extracted using edgeR version 3.32.0 from the count data of each sample. The DEGs were defined as the genes exhibiting a more than fourfold difference between samples (FDR < 0.001), and the mean of the expression level of the group was >log_2_CPM = 4. Unsupervised hierarchical clustering was performed using the hclust function with Pearson correlation distances and Ward’s method (ward.D2). The PCA was performed using FactMineR version 2.4.

### Comparison of gene expression among SWR, humans, and mice

To compare rhinoceros genes with the genes of humans and mice, we made a one-to-one correspondence table of genes by genomic coordinate comparison as described previously (*54*, *55*). All transcript annotations for rhinoceros were converted to human genome coordinates using LiftOver. The chain files applied for LiftOver (cerSim1ToHg38.over.chain, hg38ToCerSim1.over.chain, hg38ToMm10.over.chain, and mm10ToHg38.overchain) were obtained from the UCSC Genome Browser Gateway (https://genome.ucsc.edu/cgi-bin/hgGateway?redirect=manual&source=genome.ucsc.edu). Then, the rhinoceros gene annotations in human coordinates were compared with cerSim1.ncbiRefSeq, followed by a search for the corresponding cerSim1.ncbiRefSeq transcripts. We performed the same procedure on hg38.ncbiRefSeq, in which transcript annotations in hg38.ncbiRefSeq were converted to rhinoceros coordinates using LiftOver, followed by a search for the corresponding rhinoceros transcripts. The two files, rhinoceros to human and human to rhinoceros, were compared, the corresponding genes were extracted, and a rhinoceros-human correspondence table was generated. We performed the same procedure between humans and mice to obtain a mouse-human correspondence table. By these procedures, the corresponding genes between rhinoceros and humans and between mice and humans were extracted, and a rhinoceros-human-mouse correspondence table was obtained (15,255 genes) (table S4).
