# Supplementary material for: Robust induction of primordial germ cells of white rhinoceros on the brink of extinction
Source: Sci Adv. 2022 Dec 9;8(49):eabp9683. doi: 10.1126/sciadv.abp9683 (PMC9733929; doi:10.1126/sciadv.abp9683)
Supplement: Supplementary file 1 — Figs. S1 to S9 Tables S1 to S4 [file sciadv.abp9683_sm.pdf]

Science Advances
AAAS

Supplementary Materials for

Robust induction of primordial germ cells of white rhinoceros on the brink of extinction

Masafumi Hayashi *et al.*

Corresponding author: Katsuhiko Hayashi, hayashik@gcb.med.osaka-u.ac.jp;
Thomas B. Hildebrandt, hildebrandt@izw-berlin.de; Sebastian Diecke, sebastian.diecke@mdc-berlin.de

Sci. Adv. **8**, eabp9683 (2022)
DOI: 10.1126/sciadv.abp9683

The PDF file includes:

Figs. S1 to S9
Tables S1 to S4
Legends for movies S1 and S2

Other Supplementary Material for this manuscript includes the following:

Movies S1 and S2

Fig. S1

A

Southern white rhinoceros *POU5F1* (*OCT3/4*) locus

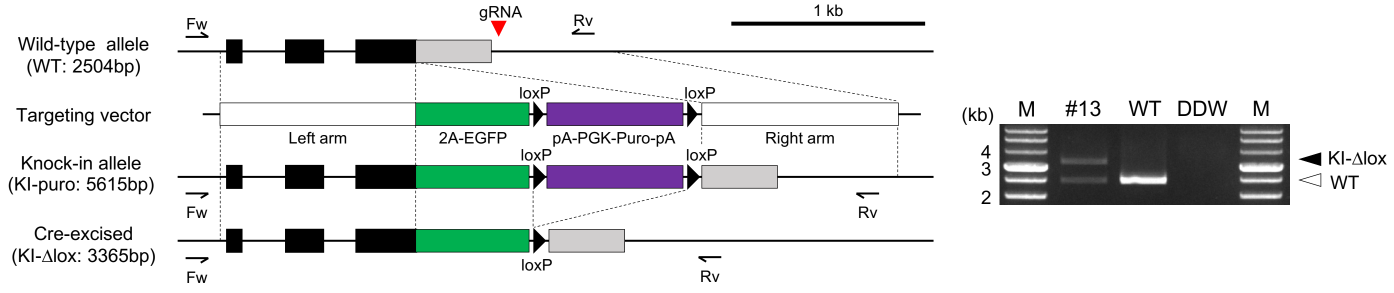

Southern white rhinoceros *PRDM1* (*BLIMP1*) locus

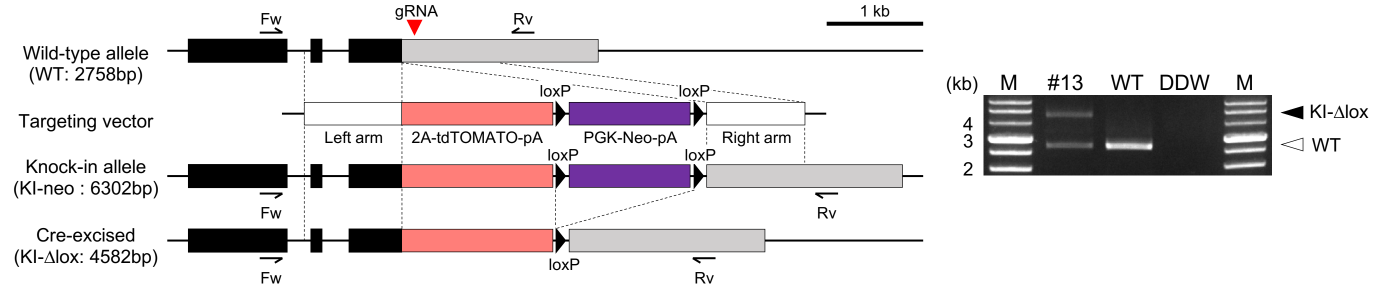

B

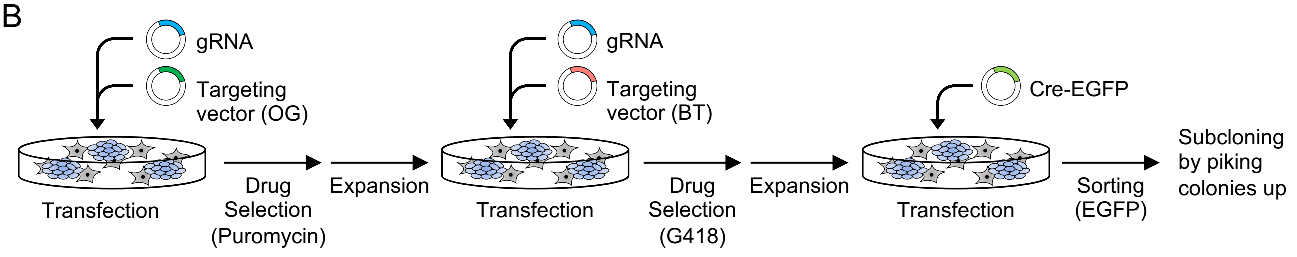

Fig. S1. Establishment of OGBT reporter lines.

(A) Schematic illustration of the targeted insertion of a reporter gene into *POU5F1* and *BLIMP1* loci. Shown are sequences of the targeted insertion of *2A-EGFP* and *2A-tdTOMATO* into *POU5F1* (top) and *BLIMP1* (bottom) loci, respectively. Black, grey, white and purple boxes indicate the exon, 3'UTR region, homologous arm of the targeting vector, and selection cassette containing a drug-resistance gene, respectively. The primers used for screening are indicated by arrows in the direction of amplification. After insertion of the targeting vector in each locus, the selection cassette was excised by Cre-mediated recombination of loxP sequences. The images at right indicate the results of PCR using the primers indicated. The expected size of each amplicon is shown in the bracket at the left. **(B)** Schematic illustration of a serial transfection of targeting vectors for the establishment of OGBT reporter lines. To save the passage number of the resultant reporter lines, OG targeting vector-transfected SWR-ESCs were propagated in the presence of puromycin, and then were immediately—i.e., without subcloning or subsequent screening—subjected to the second transfection using a BT-targeting vector. These cells were propagated in the presence of G418 and subjected to Cre-mediated loxP-excision. After sorting of the cells expressing Cre-EGFP, the cells were cultured, and then individual colonies were picked up. Genomic DNAs from the colonies were subjected to PCR analysis.

Fig. S2

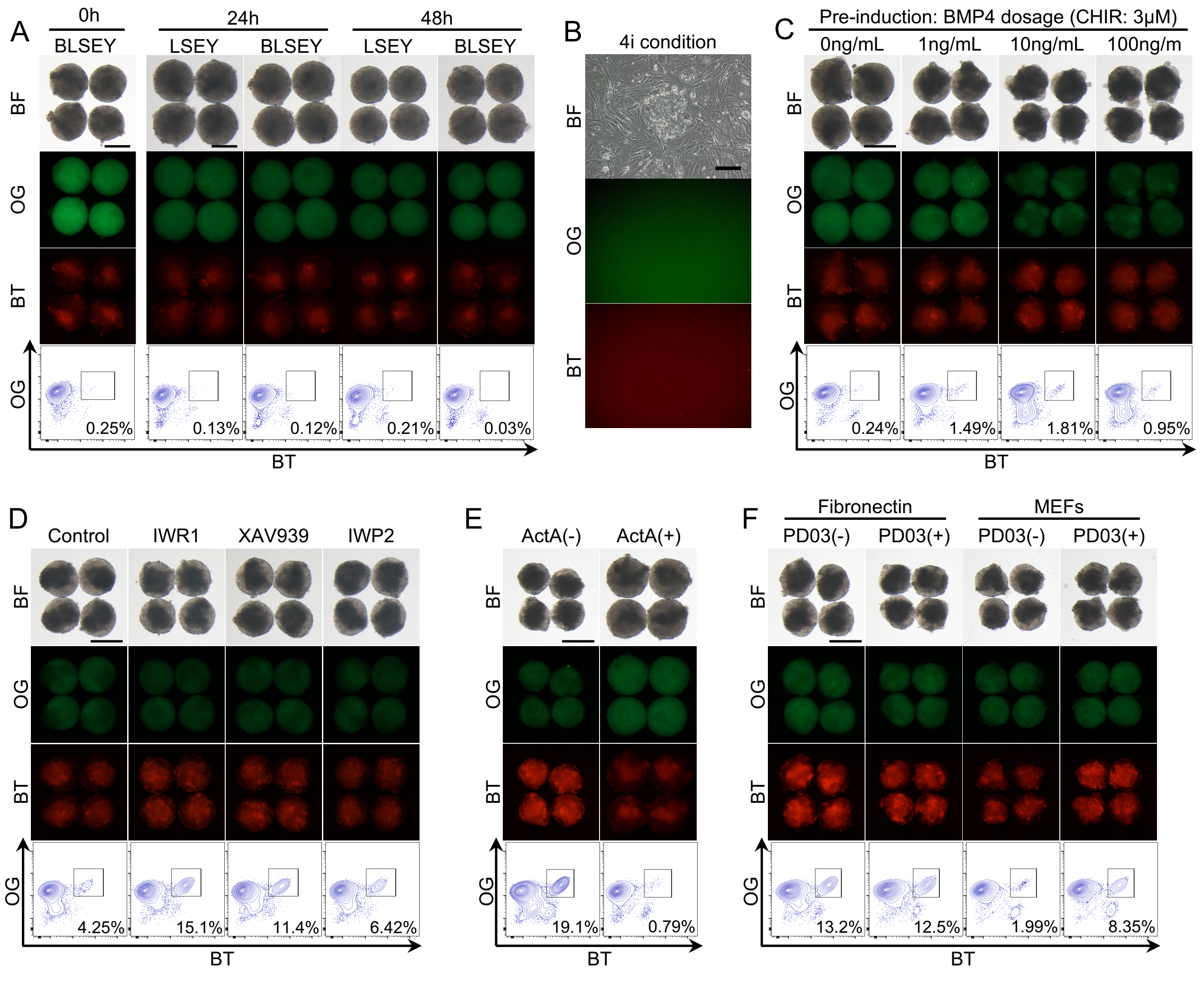

A
0h
BLSEY
24h
LSEY
BLSEY
48h
LSEY
BLSEY
BF
OG
BT
0.25%
0.13%
0.12%
0.21%
0.03%
B
4i condition
C
Pre-induction: BMP4 dosage (CHIR: 3μM)
0ng/mL
1ng/mL
10ng/mL
100ng/m
0.24%
1.49%
1.81%
0.95%
D
Control
IWR1
XAV939
IWP2
4.25%
15.1%
11.4%
6.42%
E
ActA(-)
ActA(+)
19.1%
0.79%
F
Fibronectin
MEFs
PD03(-)
PD03(+)
PD03(-)
PD03(+)
13.2%
12.5%
1.99%
8.35%

Fig. S2. Refinement of culture conditions for induction of OGBT-positive cells.

(A) No OGBT-positive cells were induced from SWR-ESCs cultured under the 4i condition. Shown are images and FACS plots of derivatives at day 4 of PGCLC induction from OGBT SWR-ESCs cultured under the 4i condition. The time on the top indicates the length of the pre-induction. BLSEY and LSEY (LIF, SCF, EGF and Y-27632) are the conditions of PGCLC induction. Scale bar, 200 μm. **(B)** OGBT SWR-ESCs cultured under the 4i condition. Note that OG expression in the cells was not as clear as that in undifferentiated OGBT SWR-ESCs (Fig. 1A). Scale bar, 200 μm. **(C)** Effect of BMP4 signaling during pre-induction. Shown are images and FACS plots of derivatives at day 4 of PGCLC induction from OGBT SWR-ESCs through the pre-induction in a culture containing BMP4 at the concentrations indicated. Scale bar, 200 μm. **(D)** Effect of WNT inhibition during PGCLC induction. Shown are images and FACS plots of OGBT SWR-ESC derivatives at day 4 of PGCLC induction with various WNT inhibitors. These cells were pre-induced with 6 μM CHIR and 10 ng/ml BMP4 for 24 h. Scale bar, 200 μm. **(E)** Effect of activin A signaling during pre-induction. Shown are images and FACS plots of derivatives at day 4 of PGCLC induction from OGBT SWR-ESCs through the pre-induction with or without activin A. PGCLC induction was performed in GK15+BLSEYWi. Scale bar, 200 μm. **(F)** Effect of FGF inhibition during pre-induction. Shown are images and FACS plots of derivatives at day 4 of PGCLC induction from OGBT SWR-ESCs through the pre-induction with or without PD0399021. PGCLC induction was performed in GK15+BLSEYWi. Scale bar, 200 μm.

Fig. S3

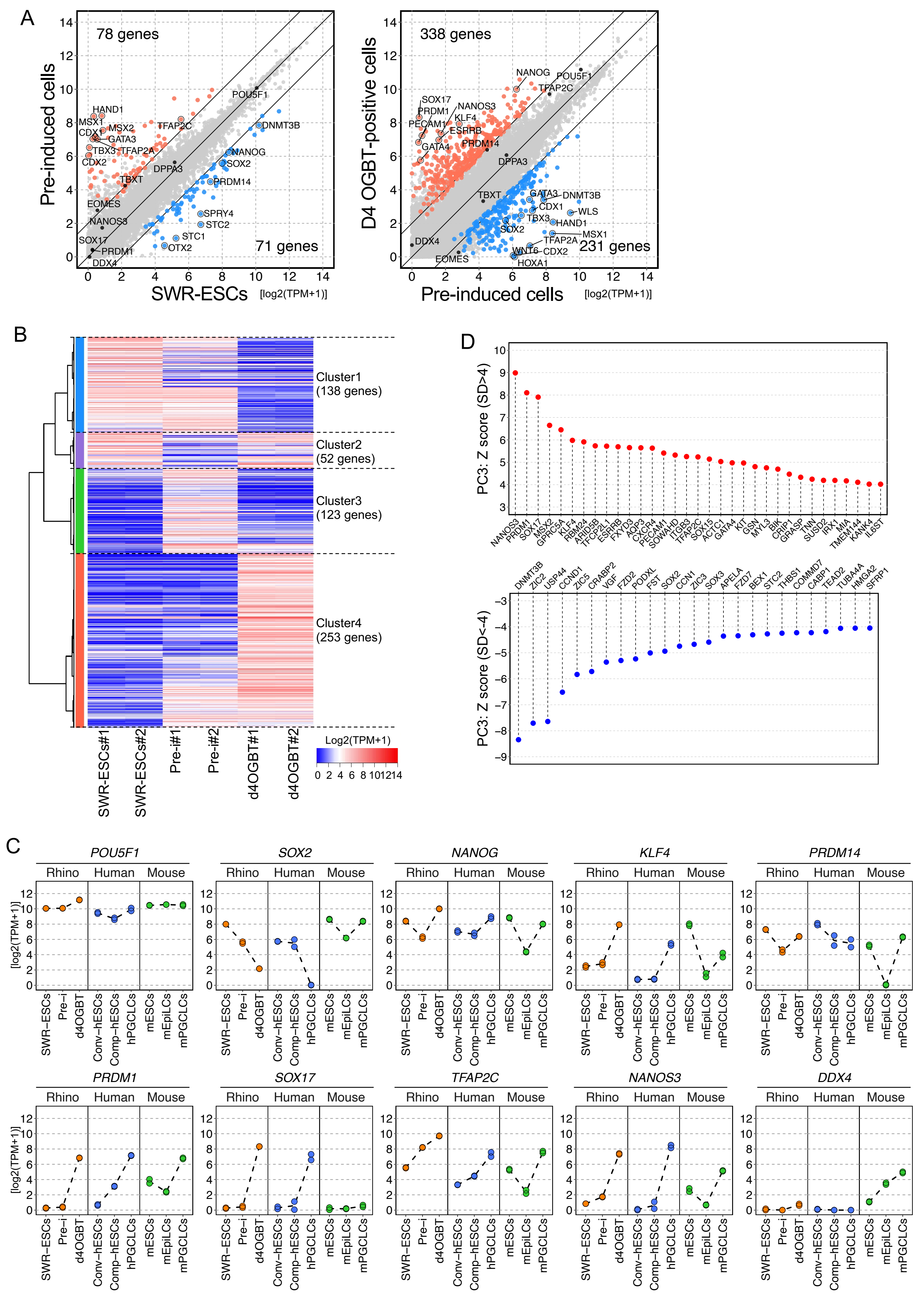

A
78 genes
71 genes
Pre-induced cells
SWR-ESCs [log2(TPM+1)]
338 genes
231 genes
D4 OGBT-positive cells
Pre-induced cells [log2(TPM+1)]
B
Cluster1 (138 genes)
Cluster2 (52 genes)
Cluster3 (123 genes)
Cluster4 (253 genes)
SWR-ESCs#1
SWR-ESCs#2
Pre-i#1
Pre-i#2
d4OGBT#1
d4OGBT#2
Log2(TPM+1)
C
POU5F1
SOX2
NANOG
KLF4
PRDM14
PRDM1
SOX17
TFAP2C
NANOS3
DDX4
Rhino
Human
Mouse
[log2(TPM+1)]
SWR-ESCs
Pre-i
d4OGBT
Conv-hESCs
Comp-hESCs
hPGCLCs
mESCs
mEpiLCs
mPGCLCs
D
PC3: Z score (SD>4)
PC3: Z score (SD<-4)

Fig. S3. Gene expression dynamics during differentiation of SWR-ESC into OGBT-positive cells

(A) Comparison of the transcriptomes of SWR-ESC derivatives. Shown are scatter plots comparing the transcriptomes of the cell types indicated. The colored dots are DEGs (>4 times, FDR <0.001, logCPM >4). The numbers of DEGs are shown in the plot. **(B)** Heatmap representation of the expression dynamics of the 566 genes (sd >3, Fig. 2E) highly contributing to PC1 or PC2. Unsupervised hierarchical clustering analysis showed that the expression dynamics can be subdivided into four clusters. Genes in each cluster are shown in Table S1. **(C)** Comparison of the expression dynamics of genes involved in PGC specification between humans, mice and SWR. Shown are the averaged values with SE of gene expression based on transcriptome analyses using biologically duplicated samples. **(D)** Genes contributing to PC3 in Figure 2F. Genes with >4 (top) or <-4 SDs (bottom) of the Z scores are shown.

Fig. S4

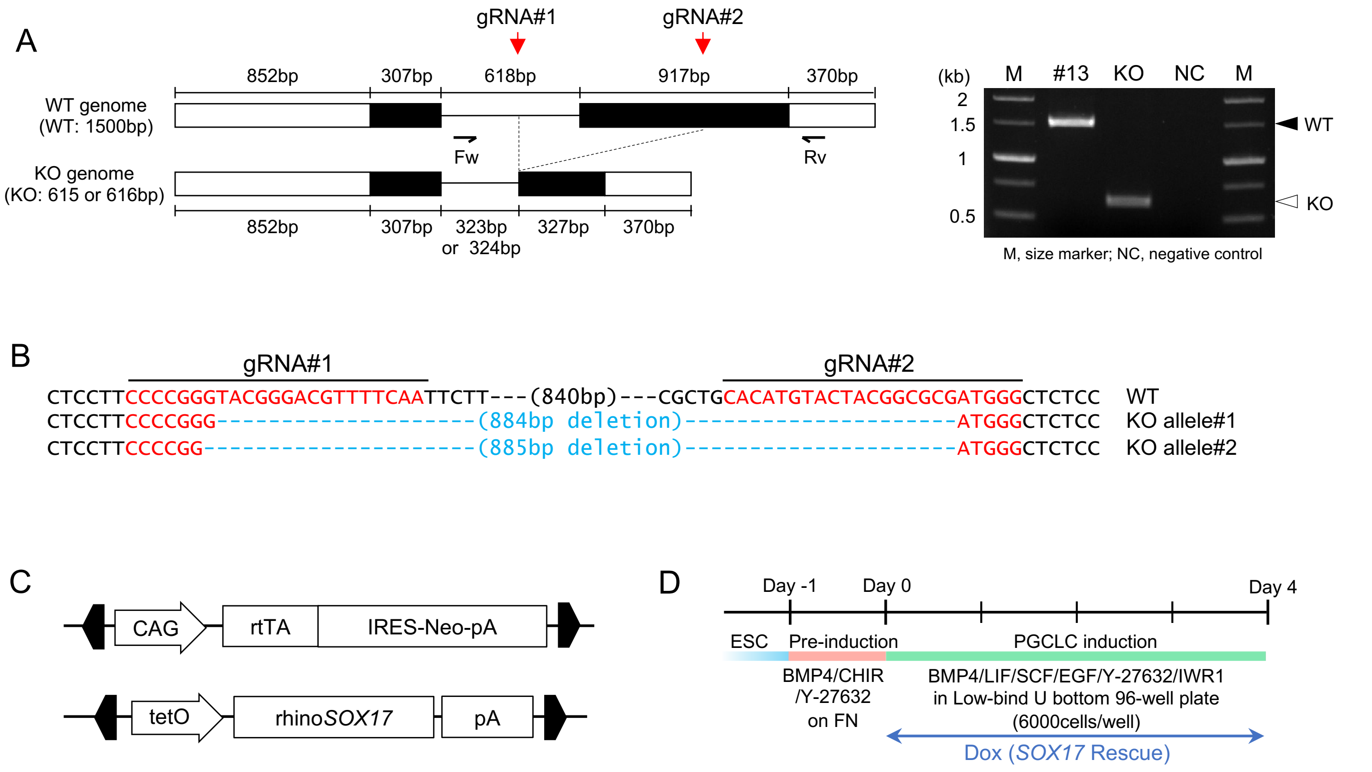
A
gRNA#1
gRNA#2
852bp
307bp
618bp
917bp
370bp
WT genome
(WT: 1500bp)
Fw
Rv
KO genome
(KO: 615 or 616bp)
852bp
307bp
323bp
or 324bp
327bp
370bp
(kb)
M
#13
KO
NC
M
2
1.5
1
0.5
WT
KO
M, size marker; NC, negative control
B
gRNA#1
gRNA#2
CTCCTTCCCCGGGTACGGGACGTTTTCAATTCTT---(840bp)---CGCTGCACATGTACTACGGCGCGATGGGCTCTCC WT
CTCCTTCCCCGGG--------------------(884bp deletion)--------------------ATGGGCTCTCC KO allele#1
CTCCTTCCCCGG--------------------(885bp deletion)--------------------ATGGGCTCTCC KO allele#2
C
CAG
rtTA
IRES-Neo-pA
tetO
rhinoSOX17
pA
D
Day -1
Day 0
Day 4
ESC
Pre-induction
PGCLC induction
BMP4/CHIR
/Y-27632
on FN
BMP4/LIF/SCF/EGF/Y-27632/IWR1
in Low-bind U bottom 96-well plate
(6000cells/well)
Dox (SOX17 Rescue)

Fig. S4. Evaluation of the function of *SOX17* in the differentiation of OGBT-positive cells.

(A) Disruption of the *SOX17* gene in SWR-ESCs. Shown are a schematic illustration of the *SOX17* gene disruption (left) and PCR analysis of the disrupted SER-ESC clone (right). Black and white boxes indicate exons and UTR regions, respectively. The gRNA and primers used are indicated by red and black arrows, respectively. The deletion is expected in both alleles as a 884-bp or 885-bp deletion. **(B)** Sequence of the disrupted genomic region. The actual deleted sequences and gRNA are shown in blue and red font, respectively. **(C)** Vectors for exogenous *SOX17* expression. The illustrations show a vector constitutively expressing rtTA with the neomycin-resistance gene driven by the CAG promoter (top) and a vector expressing the *SOX17* gene driven by the tetO promoter (bottom). Black pentagons show piggyBac-responsive sequences. **(D)** Time course of the induction of OGBT-positive cells by exogenous *SOX17* expression.

Fig. S5

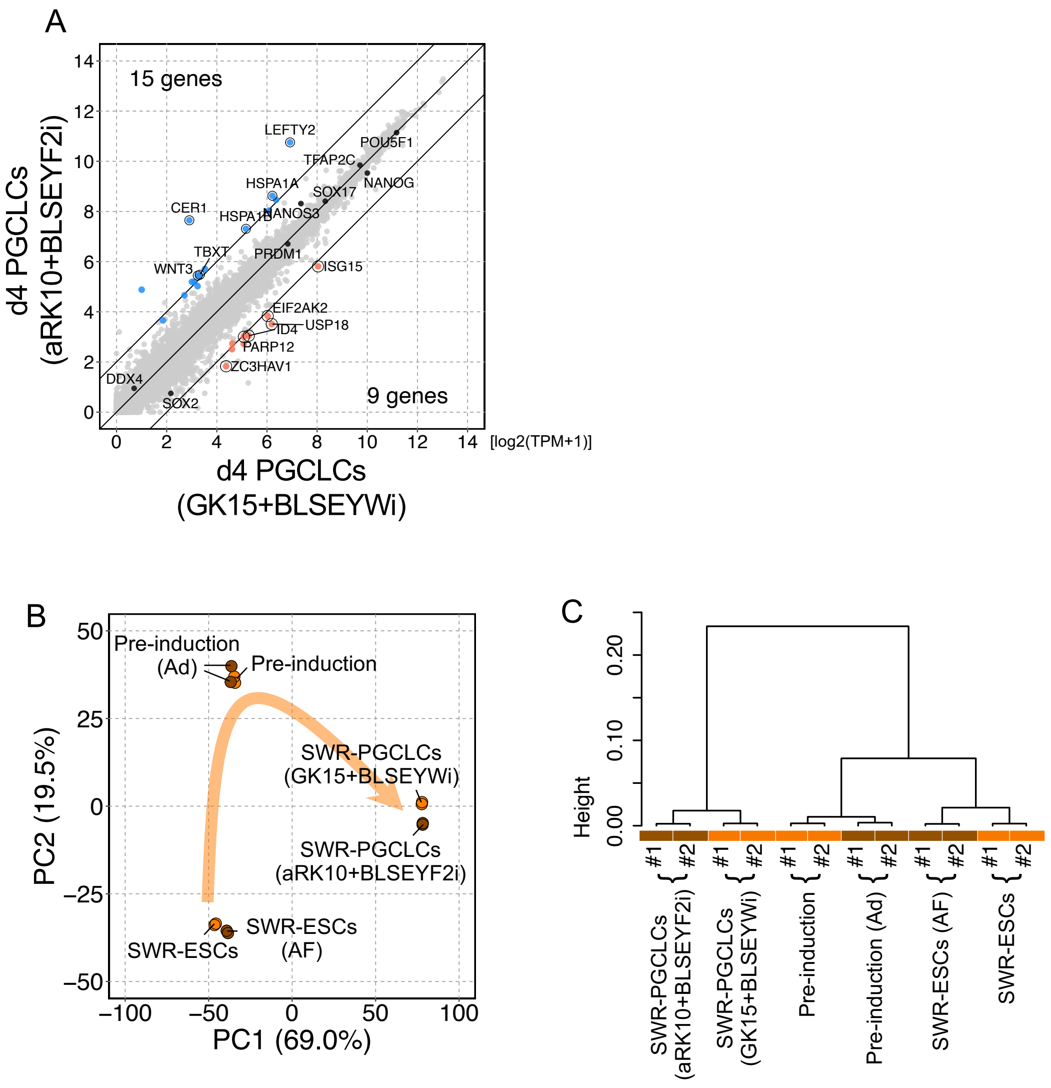
A
15 genes
9 genes
LEFTY2
POU5F1
TFAP2C
NANOG
HSPA1A
SOX17
CER1
HSPA1B
NANOS3
TBXT
WNT3
PRDM1
ISG15
EIF2AK2
USP18
ID4
PARP12
ZC3HAV1
DDX4
SOX2
d4 PGCLCs
(aRK10+BLSEYF2i)
d4 PGCLCs
(GK15+BLSEYWi)
[log2(TPM+1)]
B
Pre-induction
(Ad)
Pre-induction
SWR-PGCLCs
(GK15+BLSEYWi)
SWR-PGCLCs
(aRK10+BLSEYF2i)
SWR-ESCs
SWR-ESCs
(AF)
PC2 (19.5%)
PC1 (69.0%)
C
Height
SWR-PGCLCs
(aRK10+BLSEYF2i)
SWR-PGCLCs
(GK15+BLSEYWi)
Pre-induction
Pre-induction (Ad)
SWR-ESCs (AF)
SWR-ESCs

Fig. S5. Transcriptome analysis of SWR-PGCLCs under the advanced condition.

(A) Comparison of the transcriptomes of SWR-PGCLCs induced under the advanced condition. Shown is a scatter plot of the transcriptomes of PGCLCs induced under the GK15+BLSEYWi (original) or aRK10+BLSEYF2i (advanced) condition. The colored dots are DEGs (>4 times, FDR <0.001, logCPM >4). The numbers of DEGs are shown in the plot. **(B)** PCA of the differentiation trajectory under the advanced condition. Note that the transcriptomes of derivatives of SWR-ESCs under the advanced condition, such as SWR-ESCs(AF), Pre-induction (Ad) and SWR-PGCLCs (aRK10+BLSEYF2i), were similar to their counterparts under the original condition. **(C)** Unsupervised hierarchical clustering analysis of SWR ESC-derivatives under the original and advanced conditions.

Fig. S6

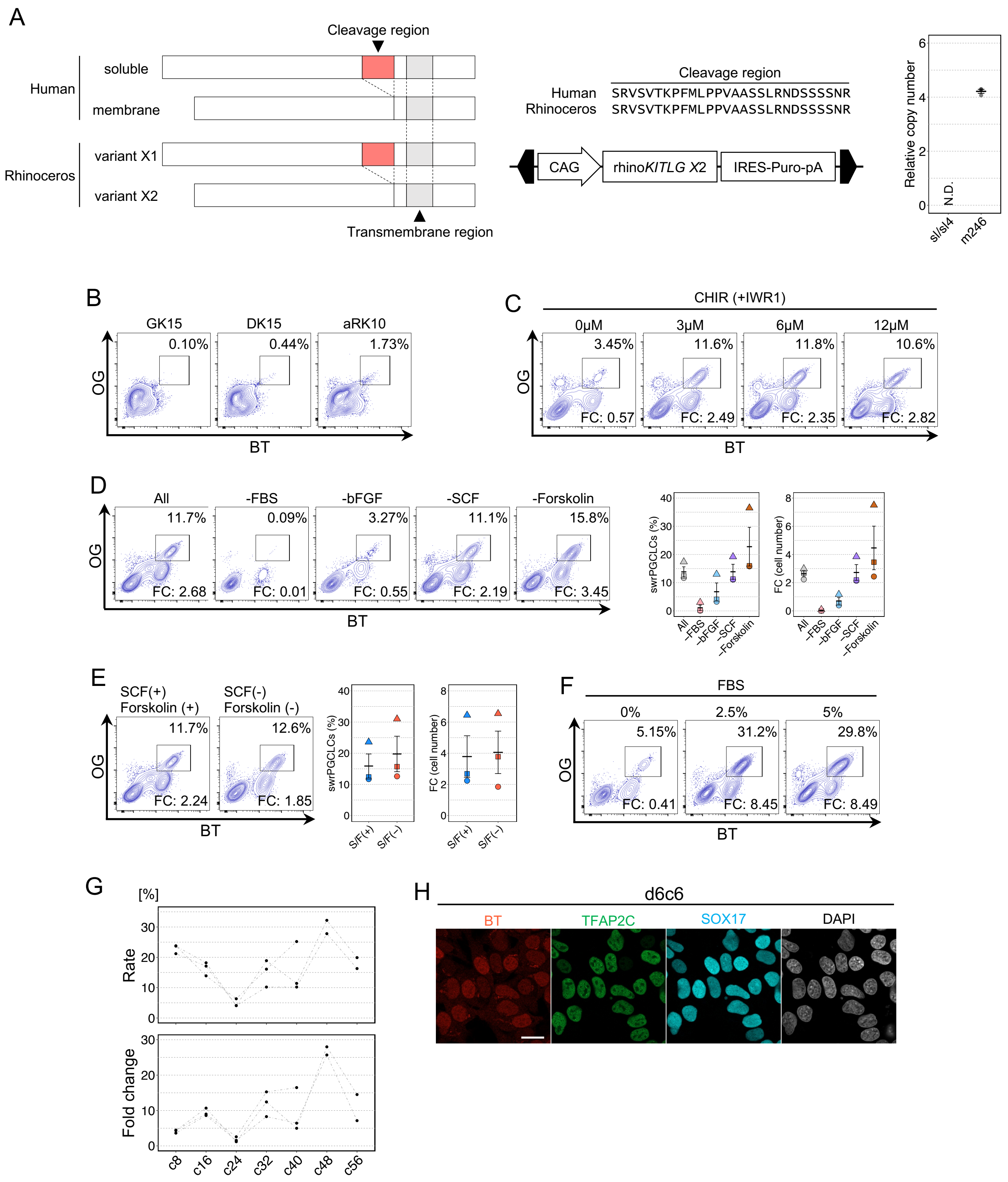
A
Cleavage region
Human
soluble
membrane
Rhinoceros
variant X1
variant X2
Transmembrane region
Cleavage region
Human SRVSVTKPFMLPPVAASSLRNDSSSSNR
Rhinoceros SRVSVTKPFMLPPVAASSLRNDSSSSNR
CAG
rhinoKITLG X2
IRES-Puro-pA
Relative copy number
N.D.
sl/sl4
m246
B
GK15
DK15
aRK10
0.10%
0.44%
1.73%
OG
BT
C
CHIR (+IWR1)
0μM
3μM
6μM
12μM
3.45%
11.6%
11.8%
10.6%
FC: 0.57
FC: 2.49
FC: 2.35
FC: 2.82
OG
BT
D
All
-FBS
-bFGF
-SCF
-Forskolin
11.7%
0.09%
3.27%
11.1%
15.8%
FC: 2.68
FC: 0.01
FC: 0.55
FC: 2.19
FC: 3.45
OG
BT
swrPGCLCs (%)
FC (cell number)
E
SCF(+)
Forskolin (+)
SCF(-)
Forskolin (-)
11.7%
12.6%
FC: 2.24
FC: 1.85
OG
BT
swrPGCLCs (%)
FC (cell number)
S/F(+)
S/F(-)
F
FBS
0%
2.5%
5%
5.15%
31.2%
29.8%
FC: 0.41
FC: 8.45
FC: 8.49
OG
BT
G
[%]
Rate
Fold change
c8
c16
c24
c32
c40
c48
c56
H
d6c6
BT
TFAP2C
SOX17
DAPI

Fig. S6. Refinement of conditions for a long-term culture of SWR-PGCLCs

(A) Generation of m246 feeder cells. Structures of soluble and membrane-bound KITLG in humans and rhinoceros are depicted (left). The amino acid sequences of the cleavage sites are well-conserved between humans and rhinoceros (middle, upper), and rhinoceros *KITLG X2*, which does not encode the cleave site, is inserted between the CAG promoter and *IRES* sequences. Integration of the construct in Sl/SL4 was confirmed by Q-PCR analysis using the primers listed in Table S2. **(B)** Effect of basal media on the propagation of SWR-PGCLCs. FACS plots show the expression of OG and BT in cells cultured with the basal medium shown on the top. **(C)** Effect of CHIR dosage. The percentage of SWR-PGCLCs and FCs of each cell number, compared with the initial number of cells spread on the plate, is shown under each condition. **(D)** Effect of the removal of each factor. The plots at right are a summary of the percentage (left) and FC (right) of SWR-PGCLCs in the aggregates. Results are shown for n=3 biologically independent experiments. **(E)** Effect of the removal of forskolin. The plots at right are a summary of the percentage (left) and FC (right) of SWR-PGCLCs in the aggregates. Results are shown for n=3 biologically independent experiments. **(F)** Effect of FBS concentration. **(G)** Percentage and fold change of SWR-ESCs during long-term culture. FC values were calculated by dividing OGBT-positive cells by the initial number of OGBT-positive cells spread on the plate. **(H)** Co-expression of key transcription factors in SWR-PGCLCs at day 6 of the long-term culture. The results of immunofluorescent analysis of the indicated transcription factors are shown. Scale bar, 20 μm.

Fig. S7

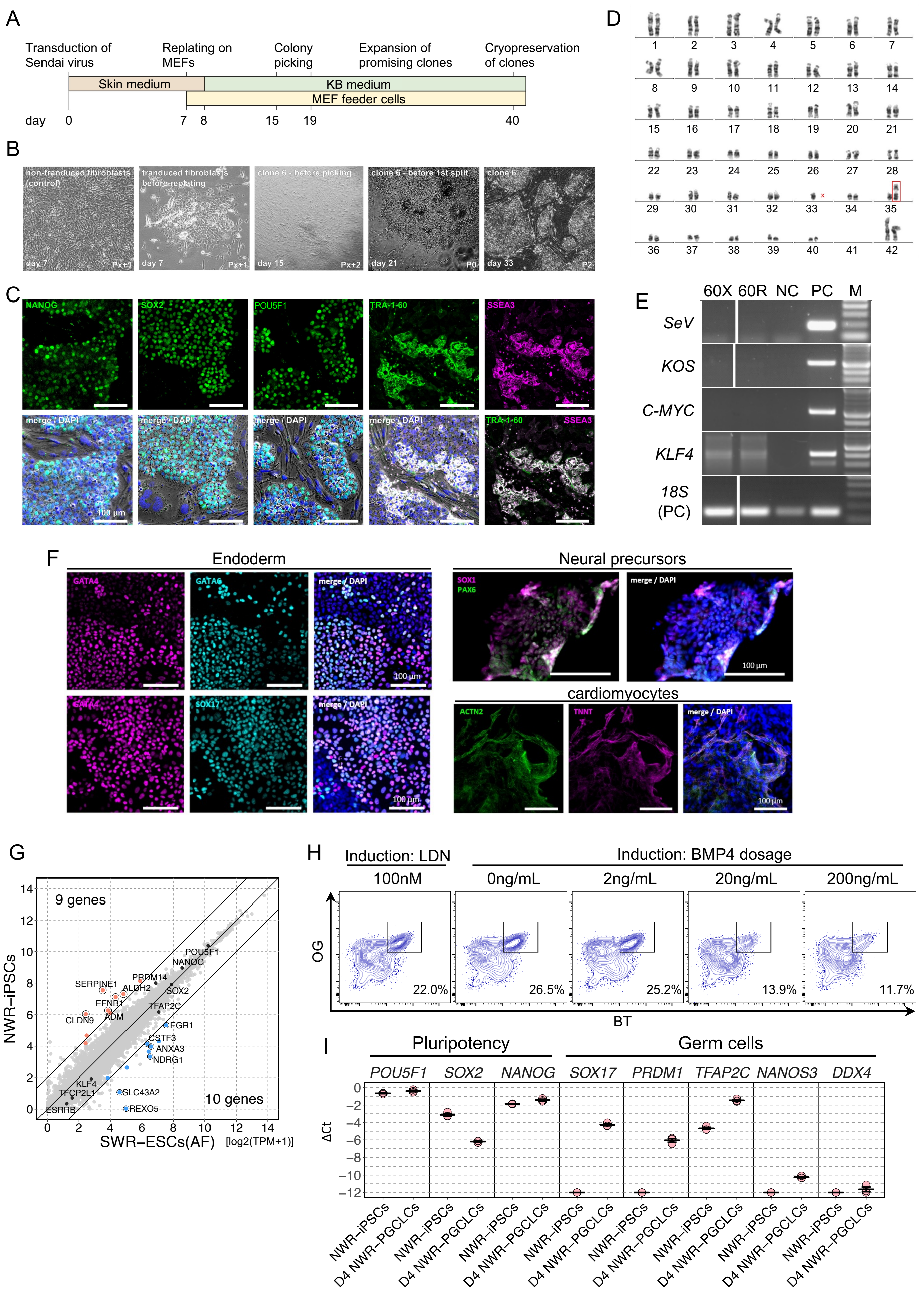

A
Transduction of Sendai virus
Replating on MEFs
Colony picking
Expansion of promising clones
Cryopreservation of clones
Skin medium
KB medium
MEF feeder cells
day 0 7 8 15 19 40
B
non-tranduced fibroblasts (control)
tranduced fibroblasts before replating
clone 6 - before picking
clone 6 - before 1st split
clone 6
day 7 Px+1
day 7 Px+1
day 15 Px+2
day 21 P0
day 33 P2
C
NANOG
SOX2
POU5F1
TRA-1-60
SSEA3
merge / DAPI
100 µm
D
E
60X 60R NC PC M
SeV
KOS
C-MYC
KLF4
18S (PC)
F
Endoderm
GATA4
GATA6
SOX17
merge / DAPI
Neural precursors
SOX1
PAX6
cardiomyocytes
ACTN2
TNNT
G
9 genes
10 genes
NWR–iPSCs
SWR–ESCs(AF)
[log2(TPM+1)]
POU5F1
NANOG
PRDM14
SERPINE1
ALDH2
SOX2
EFNB1
TFAP2C
CLDN9
ADM
EGR1
CSTF3
ANXA3
NDRG1
KLF4
TFCP2L1
ESRRB
SLC43A2
REXO5
H
Induction: LDN
100nM
Induction: BMP4 dosage
0ng/mL
2ng/mL
20ng/mL
200ng/mL
OG
BT
22.0%
26.5%
25.2%
13.9%
11.7%
I
Pluripotency
Germ cells
POU5F1
SOX2
NANOG
SOX17
PRDM1
TFAP2C
NANOS3
DDX4
ΔCt
NWR–iPSCs
D4 NWR–PGCLCs

Fig. S7. Gene expression profiles of NWR-iPSCs and their derivatives.

(A) Time course of reprogramming for generation of NWR-iPSCs. **(B)** Changing cell appearance during reprogramming. Shown are brightfield images of cells at days indicated. **(C)** Expression of pluripotency markers in the NWR-iPSCs. Shown are representative images of immunofluorescent analysis of NANOG, SOX2, POU5F1, TRA-1-60 and SSEA3 with their merged images with brightfield and DAPI. Scale bars: 100 μm. **(D)** Representative karyotype of NWR- iPSCs with 2n=81, XX, mar. The marker chromosome (mar, labeled in red) is probably the result of an Robertsonian translocation of chromosomes 33 and 35. **(E)** PCR analysis of Sendai virus-encoded RNA in NWR-iPSCs. Shown are the results of RT-PCR of total RNAs isolated from NWR-iPSC lines, 60R and 60X. PC: positive control; NC: negative control; M: marker. **(F)** Differentiation of NWR-iPSCs into three germ layers. Shown are representative results from immunofluorescent analysis of markers upon differentiation towards endoderm, cardiomyocytes (mesoderm), and neural precursors (ectoderm). Scale bars: 100 μm. **(G)** Comparison of the transcriptomes of NWR-iPSCs and SWR-ESCs used in this study. Shown is a scatter plot of the transcriptomes of NWR-iPSCs and SWR-ESCs with DEGs (>4 times, FDR <0.001, logCPM >4). **(H)** Effect of BMP4 signaling during PGCLC induction. FACS plots show the expression of OG and BT in OGBT NWR-iPSC derivatives at day 4 of PGCLC induction with a BMP inhibitor (LDN) or various concentrations of BMP4. n = 2, biologically independent experiments. **(I)** Expression of key genes between NWR-iPSCs and NWR-PGCLCs. Shown are averaged ΔCt values of gene expression determined by Q-PCR analysis using biologically triplicated samples. Bars indicate the mean values. Each cell type was isolated by FACS either to remove MEFs from ESCs or to isolate PGCLCs.

Fig. S8

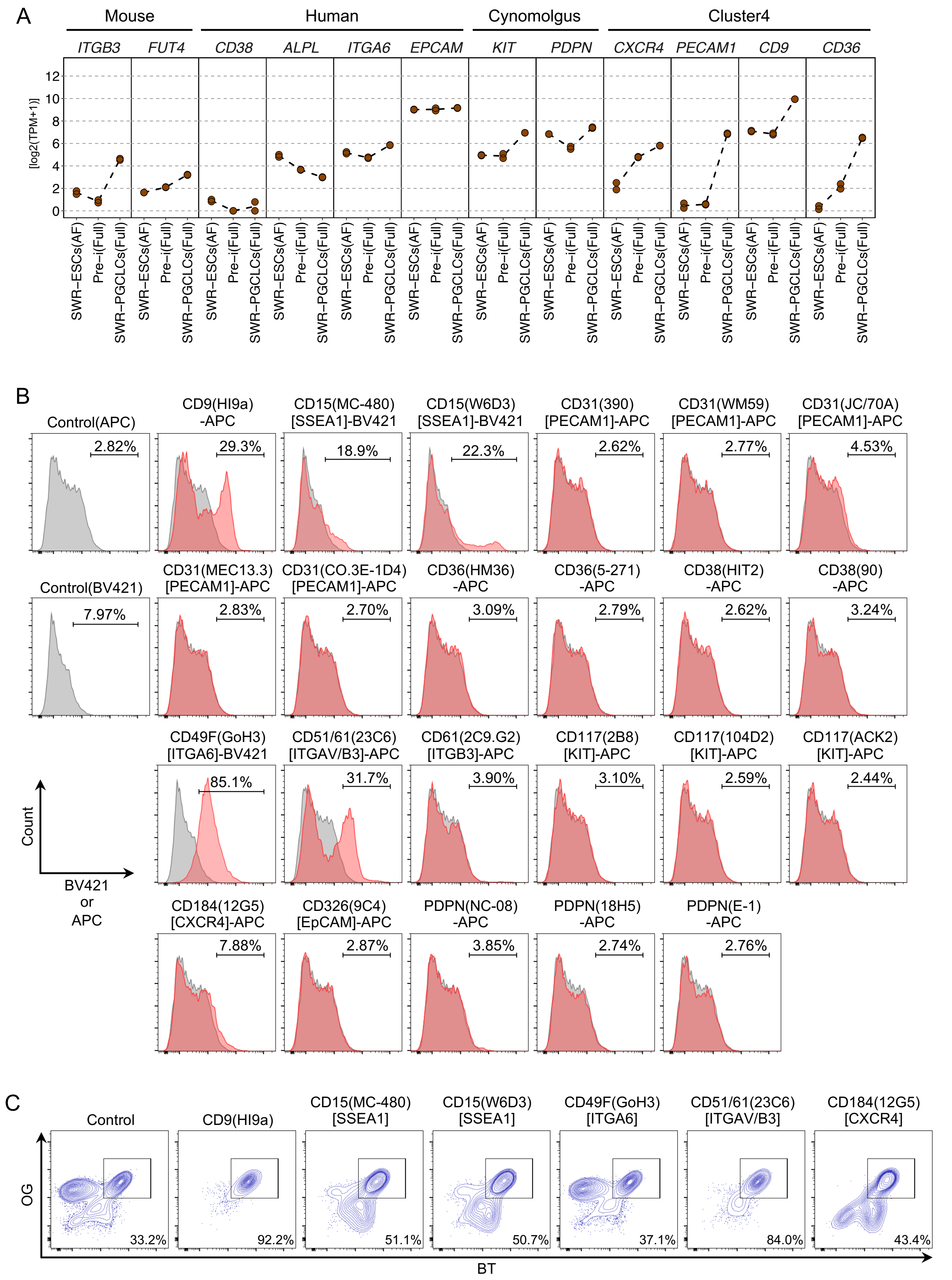

A
Mouse
Human
Cynomolgus
Cluster4
ITGB3
FUT4
CD38
ALPL
ITGA6
EPCAM
KIT
PDPN
CXCR4
PECAM1
CD9
CD36
[log2(TPM+1)]
SWR−ESCs(AF)
Pre−i(Full)
SWR−PGCLCs(Full)
B
Control(APC) 2.82%
CD9(HI9a) -APC 29.3%
CD15(MC-480) [SSEA1]-BV421 18.9%
CD15(W6D3) [SSEA1]-BV421 22.3%
CD31(390) [PECAM1]-APC 2.62%
CD31(WM59) [PECAM1]-APC 2.77%
CD31(JC/70A) [PECAM1]-APC 4.53%
Control(BV421) 7.97%
CD31(MEC13.3) [PECAM1]-APC 2.83%
CD31(CO.3E-1D4) [PECAM1]-APC 2.70%
CD36(HM36) -APC 3.09%
CD36(5-271) -APC 2.79%
CD38(HIT2) -APC 2.62%
CD38(90) -APC 3.24%
CD49F(GoH3) [ITGA6]-BV421 85.1%
CD51/61(23C6) [ITGAV/B3]-APC 31.7%
CD61(2C9.G2) [ITGB3]-APC 3.90%
CD117(2B8) [KIT]-APC 3.10%
CD117(104D2) [KIT]-APC 2.59%
CD117(ACK2) [KIT]-APC 2.44%
Count
BV421 or APC
CD184(12G5) [CXCR4]-APC 7.88%
CD326(9C4) [EpCAM]-APC 2.87%
PDPN(NC-08) -APC 3.85%
PDPN(18H5) -APC 2.74%
PDPN(E-1) -APC 2.76%
C
Control 33.2%
CD9(HI9a) 92.2%
CD15(MC-480) [SSEA1] 51.1%
CD15(W6D3) [SSEA1] 50.7%
CD49F(GoH3) [ITGA6] 37.1%
CD51/61(23C6) [ITGAV/B3] 84.0%
CD184(12G5) [CXCR4] 43.4%
OG
BT

Fig. S8. Exploring surface marker proteins for use in the isolation of PGCLCs.

(A) Expression of candidate genes for surface marker proteins between NWR-iPSCs and NWR-PGCLCs. Shown are the averaged values with SE of gene expression based on transcriptome analyses using biologically duplicated samples. Genes encoding surface marker proteins of PGCLCs in mice, humans and cynomolgus monkeys are shown. CXCR4, PECAM1, CD9 and CD36 are chosen from genes in the cluster 4 shown in fig. S3B. **(B)** FACS analyses of expression of surface marker proteins. Histograms show detectable levels of surface marker proteins (red histograms) merged with background without the antibody (grey histograms) in aggregates at day 6 of PGCLC induction. The bracket and scare bracket show the ID of the monoclonal antibody and an alias of the surface protein, respectively. The percentages show the cell population stained with the antibody. **(C)** BT and OG expression in cell population stained with the antibody. Shown are FACS plots for BT and OG expression in the gated cells in the histogram shown in B.

Fig. S9

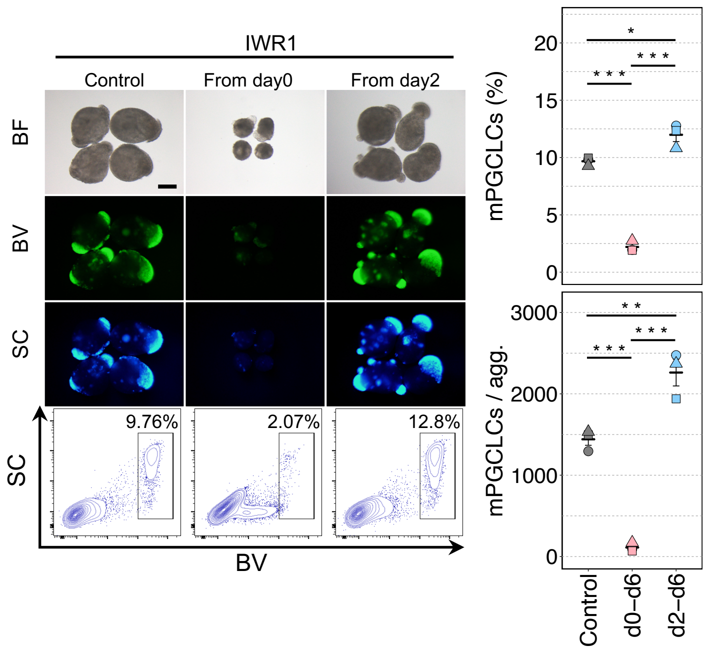
IWR1
Control
From day0
From day2
BF
BV
SC
SC
BV
9.76%
2.07%
12.8%
mPGCLCs (%)
mPGCLCs / agg.
Control
d0–d6
d2–d6

Fig. S9. Conserved effect of WNT inhibition on mouse PGCLC induction.

Effect of WNT inhibition during mouse PGCLC induction. Shown are images and FACS plots of PGCLCs at day 6 of induction from *Blimp1-mVenus* (BV) and *stella-ECFP* (SC) mouse ESCs in the presence of IWR1 from day 0 or 2 of induction. In the control, no IWR1 was added. The right box plots summarize the percentage and number of OGBT-positive cells per aggregate. Scale bar, 200 μm. n = 3, biologically independent experiments shown with SE. *P<0.05, **P<0.01, ***P<0.001, Tukey–Kramer test.

Table S1: Genes enriched in each cluster shown in Figure S3B

Cluster 1
ACSL4, ADAMTS1, ADGRL2, ADM, ANGPTL4, APELA, ARL6IP5, ASPHD1, BAAT, BCAM, BEX1, BFSP1, BTBD17, CABP1, CADM3, CAV1, CCN1, CCND1, CDH3, CDH6, COL1A1, COL5A2, COMMD7, CRABP2, CRYAB, DACT1, DDAH1, DNALI1, DNMT3B, DUSP4, DUSP6, EFNB1, FAM162A, FAM43A, FCGRT, FILIP1, FKBP10, FOXB2, FST, FZD10, FZD2, FZD7, GALNT11, GDPD2, GLS2, GZMM, HAPLN3, HK2, HMGA2, ICAM1, IGDCC3, IGF1R, IGFBP4, JADE1, KLHDC7A, LDB2, LDHA, LOXL1, LOXL2, LTBP4, LZTS1, MICAL2, MSI1, MYCN, MYF5, NDUFA4L2, NINL, NLGN3, NODAL, NPAS1, PCLAF, PDE4B, PDGFB, PFKFB3, PGLYRP2, PIDD1, PKP2, PLAUR, PLEK, PODXL, PPP1R17, PREX2, PRKAG3, PTH2, PTX3, RAB7B, RARG, RBP1, RCN3, RND3, SEMA6A, SERPINE1, SFRP2, SLC20A1, SLC27A3, SLC2A1, SLC37A1, SLC39A14, SLIT2, SLITRK5, SMAD6, SMPDL3B, SOX12, SOX2, SP8, SPOCK3, SPTSSB, SQOR, SST, STC1, STON2, STXBP2, SULF1, SUSD4, SV2A, SYT10, TACC1, TAPBP, TEAD2, THBS1, TMEM255A, TMEM98, TNFSF11, TPBG, TRABD2B, TXNIP, UNC5B, UPK2, USH1G, USP44, VGF, WIF1, ZBTB12, ZIC2, ZIC3, ZIC5, ZNF445, ZSCAN10
Cluster 2
ABCC4, AMN, APLN, ARHGAP42, BHLHE41, BNC2, CA2, CAV2, CD247, CNGB1, CYP1A1, DPPA4, ENC1, ETV4, ETV5, FGFR4, FZD5, GPC4, HESX1, IL13RA1, KRT7, LIPK, LRAT, MAFF, NANOG, NAV1, NEDD9, NPHS2, NUAK2, OPRK1, OSMR, OSTF1, OTX2, PGBD5, PKDCC, PLAT, PLCG2, PLPP3, PNMA2, PRDM14, RNF168, SLC16A3, SLC2A3, SNAI1, SPRY4, SSH1, STARD8, STC2, TCF15, TMEM179, TRIB1, USP43
Cluster 3
ADAMTS17, ARSB, ATOH8, AXIN2, B4GALT3, BAHCC1, BAMBI, BMP4, C1GALT1, CAPN6, CDX1, CDX2, CHRDL2, CITED2, CLDN1, CLEC7A, CXCL12, CXXC5, CYP11A1, DAB2, DCX, DKK1, DLX2, DLX3, DLX4, DLX5, DLX6, DPP6, DUSP2, DYNC1I1, DYRK3, EFNA1, EHHADH, EOMES, EPN3, FBN2, FIBIN, FOS, GABRP, GATA2, GATA3, GATA5, GNA14, GREB1L, GRHL3, GRIN3A, HAND1, HCN4, HNF4G, HOXA1, HSD17B14, HSD17B6, HSD3B2, ID1, ID2, ID3, IGFBP5, IL6, ISLR2, ITGB4, ITPKA, KALRN, LAYN, LEF1, LIX1, LRP2, LRRTM1, LYN, MAFB, MSX1, NECTIN4, NOTUM, NPNT, NRP2, NSG1, OAF, OVOL1, P3H2, PDGFRB, PRR15L, PRTG, PTGS2, RAP1GAP2, RARRES1, RBBP8NL, RGS4, RGS5, RNF43, S1PR3, SAMD11, SCN2B, SCN4B, SERPINA5, SH3RF1, SIX2, SLC34A2, SLC6A8, SLC7A8, SLITRK6, SMAD9, SOX14, SP5, SP6, STMN3, TBX3, TBXT, TCF7, TFAP2A, THEMIS2, TMEM132C, TMPRSS2, TNFRSF19, TP63, TSC22D3, TSPAN12, TTC29, UPK3A, VWCE, WLS, WNT3, WNT6, ZC2HC1C, ZFPM1,
Cluster 4
ABCG2, ACTA2, ACTC1, ADAM8, ADAMTSL4, ADCY4, AFAP1L2, AGPAT2, AKR1A1, ALDH3A1, AMPH, ANPEP, ANXA1, APCDD1, APOA1, AQP3, ARHGAP24, ARID5B, ATF3, ATP1B1, ATP6V0A4, ATP6V1B1, ATP6V1C2, AVIL, AVP, BHLHE40, BHMT, BIK, BMF, BRWD3, C1orf162, CAMK2D, CARD6, CASP14, CCDC85A, CCL3, CCL5, CCNA1, CCR1, CD36, CD9, CDC42EP3, CDH2, CDKN1C, CDKN2D, CEBPA, CFC1B, CHRD, CISD3, CITED1, CNTFR, CNTN3, COL14A1, COL15A1, COL1A2, COL6A1, COTL1, CPS1, CR2, CRPPA, CRYBG3, CSGALNACT1, CSPG4, CSTB, CTSH, CXCL14, CXCR4, DEPTOR, DIAPH2, DNER, DOK6, DPPA2, DTL, DYSF, ECM1, EGFL6, EIF2AK2, ERAS, ESRRB, ESYT3, FAAH, FADS3, FAM20C, FAM221A, FAR2, FAXC, FBXO32, FGD5, FN3K, FOXI3, FOXO6, FRMD7, FRMPD3, FXYD3, FXYD4, GABRA3, GABRD, GATA4, GATA6, GCNT2, GJB2, GNG11, GPNMB, GPR162, GPRC5A, GRASP, GSC, GSN, HAAO, HACD4, HAPLN1, HBEGF, HEBP2, HENMT1, HLA-A, HLA-DQA1, HMOX1, HR, IFIH1, IGFBP6, IRF1, IRF7, IRF8, IRX1, ISG15, ISM2, ITPK1, KANK4, KAZN, KCNE5, KCNH3, KCNN1, KCTD1, KLF13, KLF4, KLF5, KLHDC8A, LCP1, LFNG, LGALS3, LIMCH1, LRRC31, LRRIQ3, MAN1A1, MAP3K5, MEGF11, MFSD4A, MGST1, MIA, MORC4, MS4A3, MSX2, MTMR7, MUC1, MVP, MYH6, MYL3, NANOS3, NDRG2, NEUROG1, NGEF, NIPAL3, NKX1-2, NKX6-2, NPL, NR4A1, NTNG1, NTS, OAS3, OLFML1, OLFML2B, ORM1, OXT, PAPSS2, PARP12, PCSK1N, PCSK5, PECAM1, PIK3AP1, PKIB, PLAC8, PLCL1, PLD1, PLPP4, PNCK, PPFIBP2, PPP1R14C, PRDM1, PRIMA1, PTGES, PTH1R, QPRT, RAB11FIP5, RAB20, RAMP3, RASSF9, RBM24, RHOBTB3, RNASE1, S100A14, SCN1B, SEL1L3, SFRP4, SGPP2, SHROOM4, SLC22A3, SLC36A2, SLC37A2, SLC40A1, SLC6A15, SLC6A4, SLC7A7, SMPD3, SOWAHD, SOX17, SPHK1, SPOCK2, SRD5A2, ST3GAL1, ST6GAL2, STK31, STK32A, STMN2, STYK1, SUSD2, SUSD3, SYBU, TESC, TEX13A, TEX14, TFAP2C, TFCP2L1, TFPI, TGM3, THBD, TMBIM1, TMEM116, TMEM144, TMEM171, TMEM37, TMEM61, TNFAIP6, TNN, TOX, TPH2, TRIB3, TRIM38, TTC36, TUB, UTRN, WNT11, WNT2B, XAF1,

Table S2: Primers used in this study

**Q-PCR for rhinoceros**		
**Gene**	**Forward primer**	**Reverse primer**
PPIA	GTCCAGGAATGGCAAGACCA	TCAAGCAGACGGGGGTAAAG
ARBP	CATGCTGAACATCTCGCCCT	AGCGAGAATGCAGAGCTTCC
POU5F1	ACTACGCTGTACTCCTCGGT	TGCATAGGAGAGCCCAGAGT
SOX2	CATGAAGGAGCACCCGGATTA	CAGGCAGCGTGTACTTATCCT
NANOG	TCCAGCAGATGCAAGAACTTT	GCAAGTCTTTGGCCAGTTGT
BLIMP1	ACCAAAGCATCACACGCTGA	CCGTGCAGGTCTTGGTAAGAA
TFAP2C	GTCCCAGAGGAGGCTCTGTA	AAGTCATAAGAAGTGCACCCGT
SOX17	CTCAGCCTCGTGTTGTGTTG	CACACCCTCACATTTTTCTCCC
NANOS3	GAACGCCTGTGCTCTTTCTG	GGCACACATAGTCCCGAAGG
DDX4	GCCCAGTTCTTGTTGCTACG	TTCCACAACGACCAGTACGC
DAZL	CGCTGGGGAACAAAGGAGTTA	TGGGCCATTTCCAGAGGATG
T	CGCTGTGACGGCTTATCAGA	GTACCCAGATTGCTGGCTGT
EOMES	AAATCCTTGCCCCTCCAGAC	AACACAGTGGGGCTTGTTCT
GATA3	TGCGGGCTCTACCACAAAAT	GACAGTTCGCACAGGACGTA
TFAP2A	GCCAAAAGCAGCGACAAAGA	GGTGGTCTTAGGTGCAGGAC
**Cloning of homology arms**		
POU5F1 Left arm Forward	AATTGGGCCCTCTAGAGAAGGTGTTCAGCCAAACGA	
POU5F1 Left arm Reverse	AGTTAGTGGCGGCGCTAGCGTTTGAGTGCATAGGAGAGC	
POU5F1 Right arm Forward	TATTAGGTCCCTCGAAGAGGGGTAGCCTGCCCTTCTCAGG	
POU5F1 Right arm Reverse	CACTGCTCGACCAGGAGCTCCTGGTACACCTGCCAGAGC	
POU5F1 PAM mutation#1	CTATTGGGTACTTTGTATCCCCCC	
POU5F1 PAM mutation#2	GGGGGGATACAAAGTACCCAATAG	
BLIMP1 Left arm Forward	AATTGGGCCCTCTAGAACTGAGAGCAGAGCTAACGC	
BLIMP1 Left arm Reverse	AGTTAGTGGCGCTAGCAGGATCCATTGGTTCGACTG	
BLIMP1 Right arm Forward	TATTAGGTCCCTCGAAGAGGGATTTTCAGAAAATA	
BLIMP1 Right arm Reverse	CACTGCTCGACCAGGAGCTCTTGGTCGTTTTTAAAG	
BLIMP1 PAM mutation#1	GAGCGCACGACCCCATGCATCGAT	
BLIMP1 PAM mutation#2	GTTTGCTATAGGCACTTTGACTCGCC	
BLIMP1 PAM mutation#3	GGCGAGTCAAAGTGCCTATAGCAAAC	
**Oligos eocoding gRNA**	**Forward primer**	**Reverse primr**
POU5F1	CACCCTCGAGGTACTATCTACTATTGGGTAC	AAACGTACCCAATAGTAGATAGTACCTCGAG
BLIMP1	CACCCTCGAGGAACTTATGACTTGGCGAGTC	AAACGACTCGCCAAGTCATAAGTTCCTCGAG
SOX17#1	CACCCTCGAGGTTGAAAACGTCCCGTACCCG	AAACCGGGTACGGGACGTTTTCAACCTCGAG
SOX17#2	CACCCTCGAGGCACATGTACTACGGCGCGAT	AAACATCGCGCCGTAGTACATGTGCCTCGAG
**Genotyping**	**Forward primer**	**Reverse primr**
POU5F1 GT	AGGCAGGAGCTCCCTTCATA	AAGGGCCCCGATTAGGTAGT
BLIMP1 GT	CCCTACCCCTTGAAGAAGCA	TCATTACCCGCTTTGACCCT
SOX17 GT	CCCTCTGGCTCTTCGCTCTC	AAACAACACAACACGAGGCTG
KITLG GT	GGATCTGCGAAAACCGTGTG	CGGGGACATATTTGAGGGCT
mouse OMP (genome)	GCCCACTTGATTCCCTGA	GCATCTGCTGGGTCAGGTCC
**Clearance of Sendai virus**	**Forward primer**	**Reverse primr**
SeV	GGA TCA CTA GGT GAT ATC GAG C	ACC AGA CAA GAG TTT AAG AGA TAT GTA TC
KOS	ATG CAC CGC TAC GAC GTG AGC GC	ACC TTG ACA ATC CTG ATG TGG
KLF4	TTC CTG CAT GCC AGA GGA GCC C	AAT GTA TCG AAG GTG CTC AA
C-MYC	TAA CTG ACT AGC AGG CTT GTC G	TCC ACA TAC AGT CCT GGA TGA TGA TG
**Cloning**	**Forward primer**	**Reverse primr**
TRE-SOX17	CCCTCGTAAAGTCGACGCCACCATGAGCAGCCCGGATGCGGG	ATCCCTGCAGGCTAGCTCAGACGTCAGGATAGCTGC
CAG-KITLG	CACTAGTGATCTCGAGGCCACCATGAGAGGCGCTCGCGGCGC	GTTGTGCTAGCGGCCGCTCAGACATCTTCATGCACCAGC

Table S3: Antibodies used in this study

For Immunofluorescence

Antibody	Supplier	Catalogue#
Rabbit anti-RFP	MBL life science	PM005
Mouse anti-AP2γ	SantaCruz Biotechnology	sc-12762
Goat anti-SOX17	R&D systems	AF1924
Rabbit anti-H3K9me2	Merck	07-441
Rabbit anti-H3K27me3	Merck	07-449
Mouse anti-5mC	Merck	NA81
Rabbit anti-5hmC	Active Motif	39769
Rabbit anti-SOX2	BioLegend	630802
Rabbit anti-POU5F1	abcam	ab19857
Rabbit anti-NANOG	Thermofisher Scientific	PA1-097
Rat anti-SSEA3	Thermofisher Scientific	MA1-020X
Mouse anti-TRA-1-60	Cell Signaling	4746
Mouse anti-ACTN2	Sigma Aldrich	A7811
Rabbit anti-TNNT2	abcam	ab45932
Mouse anti-GATA4	Santa Cruz	sc-25310
Rabbit anti-GATA4	Cell Signaling	36966
Goat anti-GATA6	R&D systems	AF1700
Rabbit anti-PAX6	Thermofisher Scientific	A24354
Mouse anti-NESTIN	Thermofisher Scientific	A24354
Goat anti-SOX17	Thermofisher Scientific	A24354
Rabbit anti-SOX2	Thermofisher Scientific	A24354
AlexaFluor 647 conjugated donkey anti-goat IgG	Thermofisher Scientific	A21447
AlexaFluor 568 conjugated donkey anti-rabbit IgG	Thermofisher Scientific	A10042
AlexaFluor 555 conjugated donkey anti-rabbit IgG	Thermofisher Scientific	A31572
AlexaFluor 555 conjugated goat anti-rat IgG	Thermofisher Scientific	A21434
AlexaFluor 488 conjugated donkey anti-rabbit IgG	Thermofisher Scientific	A21206
AlexaFluor 488 conjugated goat anti-mouse IgG	Thermofisher Scientific	A21042
AlexaFluor 488 conjugated donkey anti-mouse IgG	Thermofisher Scientific	A21202

For FACS

Antibody	Format	Clone	Supplier	Catalogue#
anti-CD9	APC	HI9a	BioLegend	312107
anti-CD15 (SSEA-1)	Brilliant Violet 421	MC-480	BioLegend	125613
anti-CD15 (SSEA-1)	Brilliant Violet 421	W6D3	BioLegend	323039
anti-CD31 (PECAM-1)	APC	390	BioLegend	102409
anti-CD31 (PECAM-1)	APC	WM59	BioLegend	303115
anti-CD31 (PECAM-1)	APC	MEC13.3	BD Biosciences	551262
anti-CD31 (PECAM-1)	APC	JC/70A	Novus Biologicals	NB600-562APC
anti-CD31 (PECAM-1)	unconjugated	CO.3E-1D4	Novus Biologicals	NB100-65900-0.025mg
anti-CD36	APC	HM36	BioLegend	102611
anti-CD36	APC	5-271	BioLegend	336207
anti-CD38	APC	90	BioLegend	102711
anti-CD38	APC	HIT2	eBioscience	17-0389-41
anti-CD49f (ITGA6)	Brilliant Violet 421	GoH3	BioLegend	313623
anti-CD51/61 (ITGAV/B3)	APC	23C6	BioLegend	304415
anti-CD61 (ITGB3)	APC	2C9.G2(HMβ3-1)	BioLegend	104315
anti-CD117 (c-kit)	APC	ACK2	BioLegend	135107
anti-CD117 (c-kit)	APC	2B8	BioLegend	105811
anti-CD117 (c-kit)	APC	104D2	BioLegend	313205
anti-CD184 (CXCR4)	APC	12G5	BioLegend	306509
anti-CD326 (EpCAM)	Alexa Fluor 647	9C4	BioLegend	324212
anti-Podoplanin	APC	NC-08	BioLegend	337022
anti-Podoplanin	unconjugated	18H5	Santa Cruz Biotechnology	sc-59347
anti-Podoplanin	unconjugated	E-1	Santa Cruz Biotechnology	sc-376695

Table S4: Correspondence of gene IDs

human gene	mouse gene	rhinoceros gene	human gene	mouse gene	rhinoceros gene	human gene	mouse gene	rhinoceros gene
A1CF	A1cf	LOC101401001	GDF11	Gdf11	LOC101405503	PPP1CA	Ppp1ca	LOC101405274
A2M	A2m	LOC101408849	GDF2	Gdf2	LOC101388254	PPP1CB	Ppp1cb	LOC101396313
A3GALT2	A3galt2	LOC101406381	GDF3	Gdf3	LOC101389918	PPP1CC	Ppp1cc	LOC101397581
A4GALT	A4galt	LOC101395098	GDF5	Gdf5	LOC101394396	PPP1R10	Ppp1r10	LOC101387369
A4GNT	A4gnt	LOC101406242	GDF6	Gdf6	LOC101402023	PPP1R11	Ppp1r11	LOC101402840
AAAS	Aaas	LOC101387892	GDF7	Gdf7	LOC101400025	PPP1R12A	Ppp1r12a	LOC101394590
AACS	Aacs	LOC101397504	GDF9	Gdf9	LOC101399253	PPP1R12B	Ppp1r12b	LOC101397359
AADACL2	Aadacl2	LOC101406078	GDI1	Gdi1	LOC101395218	PPP1R12C	Ppp1r12c	LOC101389485
AADACL3	Aadacl3	LOC101397316	GDI2	Gdi2	LOC101403215	PPP1R13B	Ppp1r13b	LOC101400184
AADAT	Aadat	LOC101388761	GDNF	Gdnf	LOC101407223	PPP1R13L	Ppp1r13l	LOC101396494
AAGAB	Aagab	LOC101397635	GDPD1	Gdpd1	LOC101388861	PPP1R14A	Ppp1r14a	LOC101401714
AAK1	Aak1	LOC101402034	GDPD2	Gdpd2	LOC101408682	PPP1R14B	Ppp1r14b	LOC101403433
AAMDC	Aamdc	LOC101408833	GDPD3	Gdpd3	LOC101388361	PPP1R14C	Ppp1r14c	LOC101389933
AAMP	Aamp	LOC101406974	GDPD4	Gdpd4	LOC101405431	PPP1R14D	Ppp1r14d	LOC101394114
AANAT	Aanat	LOC101404984	GDPD5	Gdpd5	LOC101402369	PPP1R16A	Ppp1r16a	LOC101387523
AAR2	Aar2	LOC101388248	GEM	Gem	LOC101398604	PPP1R16B	Ppp1r16b	LOC101399909
AARD	Aard	LOC101391583	GEMIN2	Gemin2	LOC101406663	PPP1R17	Ppp1r17	LOC101405368
AARS2	Aars2	LOC101394625	GEMIN4	Gemin4	LOC101406653	PPP1R18	Ppp1r18	LOC101407486
AARSD1	Aarsd1	LOC101389035	GEMIN5	Gemin5	LOC101390322	PPP1R1A	Ppp1r1a	LOC101399906
AASDH	Aasdh	LOC101392651	GEMIN6	Gemin6	LOC101408669	PPP1R1B	Ppp1r1b	LOC101404476
AASDHPPT	Aasdhppt	LOC101404711	GEMIN7	Gemin7	LOC101395024	PPP1R1C	Ppp1r1c	LOC101389971
AASS	Aass	LOC101405550	GEMIN8	Gemin8	LOC101391676	PPP1R2	Ppp1r2	LOC101392421
AATF	Aatf	LOC101397941	GEN1	Gen1	LOC101403090	PPP1R21	Ppp1r21	LOC101392816
AATK	Aatk	LOC101401498	GET1	Wrb	LOC101394390	PPP1R27	Ppp1r27	LOC101398029
ABAT	Abat	LOC101388607	GET3	Asna1	LOC101403465	PPP1R32	Ppp1r32	LOC101392980
ABCA1	Abca1	LOC101404539	GET4	Get4	LOC101399696	PPP1R35	Ppp1r35	LOC101393517
ABCA12	Abca12	LOC101400436	GFAP	Gfap	LOC101397936	PPP1R36	Ppp1r36	LOC101402087
ABCA13	Abca13	LOC101403172	GFER	Gfer	LOC101389826	PPP1R37	Ppp1r37	LOC101395283
ABCA2	Abca2	LOC101391991	GFI1	Gfi1	LOC101408782	PPP1R3A	Ppp1r3a	LOC101393413
ABCA3	Abca3	LOC101408062	GFI1B	Gfi1b	LOC101394369	PPP1R3B	Ppp1r3b	LOC101401055
ABCA4	Abca4	LOC101402929	GFM1	Gfm1	LOC101393688	PPP1R3C	Ppp1r3c	LOC101393453
ABCA5	Abca5	LOC101403589	GFM2	Gfm2	LOC101391789	PPP1R3E	Ppp1r3e	LOC101405127
ABCA6	Abca6	LOC101403848	GFOD1	Gfod1	LOC101405343	PPP1R3F	Ppp1r3f	LOC101401856
ABCA7	Abca7	LOC101388982	GFOD2	Gfod2	LOC101394067	PPP1R3G	Ppp1r3g	LOC101392181
ABCA8	Abca8b	LOC101390923	GFPT1	Gfpt1	LOC101402815	PPP1R42	Ppp1r42	LOC101392977
ABCA9	Abca9	LOC101403326	GFPT2	Gfpt2	LOC101391319	PPP1R7	Ppp1r7	LOC101388928
ABCB1	Abcb1a	LOC101397422	GFRA1	Gfra1	LOC101391405	PPP1R8	Ppp1r8	LOC101405063
ABCB10	Abcb10	LOC101394902	GFRA2	Gfra2	LOC101388300	PPP1R9A	Ppp1r9a	LOC101404372
ABCB11	Abcb11	LOC101404021	GFRA3	Gfra3	LOC101405459	PPP1R9B	Ppp1r9b	LOC101407532
ABCB4	Abcb4	LOC101390666	GFRA4	Gfra4	LOC101404302	PPP2CA	Ppp2ca	LOC101396247
ABCB5	Abcb5	LOC101404328	GFRAL	Gfral	LOC101393119	PPP2CB	Ppp2cb	LOC101401405
ABCB6	Abcb6	LOC101392876	GFY	Gfy	LOC101403179	PPP2R1A	Ppp2r1a	LOC101396135
ABCB7	Abcb7	LOC101397380	GGA1	Gga1	LOC101401698	PPP2R1B	Ppp2r1b	LOC101396442
ABCB8	Abcb8	LOC101387986	GGA2	Gga2	LOC101388042	PPP2R2A	Ppp2r2a	LOC101406217
ABCB9	Abcb9	LOC101389707	GGA3	Gga3	LOC101392965	PPP2R2B	Ppp2r2b	LOC101396941
ABCC1	Abcc1	LOC101400042	GGCT	Ggct	LOC101409025	PPP2R2C	Ppp2r2c	LOC101404375
ABCC10	Abcc10	LOC101402858	GGCX	Ggcx	LOC101388692	PPP2R2D	Ppp2r2d	LOC101389552
ABCC12	Abcc12	LOC101396086	GGH	Ggh	LOC101396564	PPP2R3A	Ppp2r3a	LOC101387085
ABCC2	Abcc2	LOC101398426	GGN	Ggn	LOC101402491	PPP2R3C	Ppp2r3c	LOC101391095
ABCC3	Abcc3	LOC101393736	GGNBP2	Ggnbp2	LOC101396737	PPP2R5A	Ppp2r5a	LOC101389344
ABCC4	Abcc4	LOC101395222	GGPS1	Ggps1	LOC101392545	PPP2R5B	Ppp2r5b	LOC101398547
ABCC5	Abcc5	LOC101397048	GGT1	Ggt1	LOC101390038	PPP2R5C	Ppp2r5c	LOC101391335

ABCC6	Abcc6	LOC101394111	GGT5	Ggt5	LOC101390299	PPP2R5D	Ppp2r5d	LOC101408199
ABCC8	Abcc8	LOC101406674	GGT6	Ggt6	LOC101408565	PPP2R5E	Ppp2r5e	LOC101399198
ABCC9	Abcc9	LOC101408666	GGT7	Ggt7	LOC101398521	PPP3CA	Ppp3ca	LOC101396543
ABCD1	Abcd1	LOC101408363	GHDC	Ghdc	LOC101399922	PPP3CB	Ppp3cb	LOC101391729
ABCD2	Abcd2	LOC101400835	GHITM	Ghitm	LOC101395076	PPP3CC	Ppp3cc	LOC101404524
ABCD3	Abcd3	LOC101402061	GHR	Ghr	LOC101391145	PPP3R1	Ppp3r1	LOC101406326
ABCD4	Abcd4	LOC101404960	GHRH	Ghrh	LOC101397768	PPP3R2	Ppp3r2	LOC106800745
ABCE1	Abce1	LOC101394781	GHRHR	Ghrhr	LOC101406934	PPP4C	Ppp4c	LOC101389310
ABCF1	Abcf1	LOC101387790	GHRL	Ghrl	LOC101393192	PPP4R1	Ppp4r1	LOC101397467
ABCF2	Abcf2	LOC101406798	GHSR	Ghsr	LOC101389780	PPP4R2	Ppp4r2	LOC101388206
ABCF3	Abcf3	LOC101391725	GID4	Gid4	LOC101391846	PPP4R3A	Smek1	LOC101398292
ABCG1	Abcg1	LOC101397669	GID8	Gid8	LOC101396987	PPP4R3B	Smek2	LOC101399669
ABCG2	Abcg2	LOC101406036	GIGYF1	Gigyf1	LOC101397037	PPP4R4	Ppp4r4	LOC101406141
ABCG4	Abcg4	LOC101389103	GIGYF2	Gigyf2	LOC101395923	PPP5C	Ppp5c	LOC101403098
ABCG5	Abcg5	LOC101402548	GIMAP1	Gimap1	LOC106800074	PPP6C	Ppp6c	LOC101401650
ABCG8	Abcg8	LOC101402299	GIMAP5	Gimap5	LOC101408738	PPP6R1	Ppp6r1	LOC101390777
ABHD10	Abhd10	LOC101394244	GIMAP6	Gimap6	LOC101390147	PPP6R2	Ppp6r2	LOC101402383
ABHD11	Abhd11	LOC101400667	GIMD1	Gimd1	LOC101405153	PPP6R3	Ppp6r3	LOC101390348
ABHD12	Abhd12	LOC101394958	GIN1	Gin1	LOC101408602	PPRC1	Pprc1	LOC101388666
ABHD12B	Abhd12b	LOC101397352	GINM1	Ginm1	LOC101395460	PPT1	Ppt1	LOC101406709
ABHD14A	Abhd14a	LOC101397882	GINS1	Gins1	LOC101394698	PPT2	Ppt2	LOC101387443
ABHD14B	Abhd14b	LOC101397623	GINS2	Gins2	LOC101391603	PPTC7	Pptc7	LOC101399300
ABHD15	Abhd15	LOC101389382	GINS3	Gins3	LOC101398279	PPWD1	Ppwd1	LOC101392245
ABHD16A	Abhd16a	LOC101396884	GINS4	Gins4	LOC101389663	PPY	Ppy	LOC101389470
ABHD16B	Abhd16b	LOC101391410	GIP	Gip	LOC101400011	PQBP1	Pqbp1	LOC101387148
ABHD17A	Abhd17a	LOC101408603	GIPC1	Gipc1	LOC101391135	PRADC1	Pradc1	LOC101390168
ABHD17B	Abhd17b	LOC101406245	GIPC2	Gipc2	LOC101403936	PRAF2	Praf2	LOC101406324
ABHD17C	Abhd17c	LOC101395941	GIPC3	Gipc3	LOC101401449	PRAM1	Pram1	LOC101404272
ABHD18	Abhd18	LOC101404283	GIPR	Gipr	LOC101398717	PRAMEF12	Pramef12	LOC101407056
ABHD2	Abhd2	LOC101401152	GIT1	Git1	LOC101389129	PRC1	Prc1	LOC101407259
ABHD3	Abhd3	LOC101388213	GIT2	Git2	LOC101398272	PRCC	Prcc	LOC101389044
ABHD4	Abhd4	LOC101390879	GJA1	Gja1	LOC101389768	PRCP	Prcp	LOC101389032
ABHD5	Abhd5	LOC101401884	GJA10	Gja10	LOC101398839	PRDM1	Prdm1	LOC101408711
ABHD6	Abhd6	LOC101395980	GJA4	Gja4	LOC101398857	PRDM10	Prdm10	LOC101406228
ABHD8	Abhd8	LOC101395907	GJA8	Gja8	LOC101389665	PRDM11	Prdm11	LOC101392560
ABI1	Abi1	LOC101397912	GJB1	Gjb1	LOC101404596	PRDM12	Prdm12	LOC101405058
ABI2	Abi2	LOC101395236	GJB2	Gjb2	LOC101399501	PRDM13	Prdm13	LOC101404937
ABI3	Abi3	LOC101401248	GJB3	Gjb3	LOC106800073	PRDM14	Prdm14	LOC101395868
ABI3BP	Abi3bp	LOC101394506	GJB5	Gjb5	LOC101408113	PRDM15	Prdm15	LOC101396723
ABITRAM	Fam206a	LOC101405927	GJC1	Gjc1	LOC101396208	PRDM16	Prdm16	LOC101397154
ABL1	Abl1	LOC101388054	GJD2	Gjd2	LOC101405834	PRDM2	Prdm2	LOC101397568
ABL2	Abl2	LOC101389450	GK	Gk	LOC101394411	PRDM4	Prdm4	LOC101390094
ABLIM1	Ablim1	LOC101390403	GK2	Gk2	LOC101389983	PRDM5	Prdm5	LOC101390314
ABLIM2	Ablim2	LOC101402975	GK5	Gk5	LOC101390531	PRDM6	Prdm6	LOC101391206
ABLIM3	Ablim3	LOC101405073	GKAP1	Gkap1	LOC101387503	PRDM8	Prdm8	LOC101401062
ABO	Abo	LOC101407140	GKN1	Gkn1	LOC101403516	PRDM9	Prdm9	LOC101392897
ABR	Abr	LOC101408223	GKN2	Gkn2	LOC101403780	PRDX1	Prdx1	LOC101396151
ABRA	Abra	LOC101396288	GLA	Gla	LOC101399087	PRDX2	Prdx2	LOC101404528
ABRACL	Abracl	LOC101395640	GLB1	Glb1	LOC101393256	PRDX3	Prdx3	LOC101396717
ABT1	Abt1	LOC101400677	GLB1L	Glb1l	LOC101393969	PRDX4	Prdx4	LOC101407430
ABTB1	Abtb1	LOC101397729	GLB1L2	Glb1l2	LOC101390268	PRDX5	Prdx5	LOC101393402
ABTB2	Abtb2	LOC101404484	GLB1L3	Glb1l3	LOC101397792	PRDX6	Prdx6	LOC101400428
ACAA1	Acaa1b	LOC101401882	GLCCI1	Glcci1	LOC101401330	PREB	Preb	LOC101407281
ACAA2	Acaa2	LOC101392241	GLCE	Glce	LOC101401548	PRELID1	Prelid1	LOC101402963

ACACA	Acaca	LOC101398204	GLDC	Gldc	LOC101392114	PRELID2	Prelid2	LOC101402059
ACACB	Acacb	LOC101393718	GLDN	Gldn	LOC101407215	PRELP	Prelp	LOC101406968
ACAD10	Acad10	LOC101399652	GLE1	Gle1	LOC101398494	PREP	Prep	LOC101408450
ACAD11	Acad11	LOC101391958	GLG1	Glg1	LOC101408846	PREPL	Prepl	LOC101400190
ACAD8	Acad8	LOC101390006	GLI1	Gli1	LOC101393891	PREX1	Prex1	LOC101389282
ACAD9	Acad9	LOC101390958	GLI2	Gli2	LOC101389962	PREX2	Prex2	LOC101394577
ACADL	Acadl	LOC101396277	GLI3	Gli3	LOC101394871	PRF1	Prf1	LOC101402008
ACADM	Acadm	LOC101397586	GLIPR1	Glipr1	LOC101390271	PRG2	Prg2	LOC101395706
ACADS	Acads	LOC101401225	GLIPR1L2	Glipr1l2	LOC101390009	PRG3	Prg3	LOC106800019
ACADSB	Acadsb	LOC101404919	GLIPR2	Glipr2	LOC101396085	PRG4	Prg4	LOC101396267
ACADVL	Acadvl	LOC101389723	GLIS1	Glis1	LOC101390520	PRICKLE1	Prickle1	LOC101405674
ACAN	Acan	LOC101400358	GLIS2	Glis2	LOC101396925	PRICKLE2	Prickle2	LOC101403452
ACAP1	Acap1	LOC101406467	GLIS3	Glis3	LOC101396853	PRICKLE3	Prickle3	LOC101404117
ACAP2	Acap2	LOC101392672	GLMN	Glmn	LOC101387513	PRICKLE4	Prickle4	LOC101393616
ACAP3	Acap3	LOC101389869	GLMP	Glmp	LOC101405441	PRIM1	Prim1	LOC101408732
ACAT1	Acat1	LOC101402175	GLO1	Glo1	LOC101399281	PRIMA1	Prima1	LOC101404036
ACAT2	Acat2	LOC101408076	GLOD4	Glod4	LOC101407425	PRIMPOL	Primpol	LOC101406028
ACBD3	Acbd3	LOC101408505	GLOD5	Glod5	LOC101389136	PRKAA1	Prkaa1	LOC101389261
ACBD4	Acbd4	LOC101399573	GLP1R	Glp1r	LOC101404542	PRKAA2	Prkaa2	LOC101398137
ACBD5	Acbd5	LOC101397655	GLP2R	Glp2r	LOC101408742	PRKAB1	Prkab1	LOC101406899
ACBD6	Acbd6	LOC101408203	GLRA1	Glra1	LOC101394385	PRKAB2	Prkab2	LOC101391886
ACBD7	Acbd7	LOC101390646	GLRA2	Glra2	LOC101392193	PRKACA	Prkaca	LOC101389673
ACCS	Accs	LOC101395181	GLRA3	Glra3	LOC101392186	PRKACB	Prkacb	LOC101406209
ACCSL	Accsl	LOC106802906	GLRB	Glrb	LOC101390289	PRKAG1	Prkag1	LOC101400250
ACD	Acd	LOC101401938	GLRX	Glrx	LOC101391376	PRKAG2	Prkag2	LOC101404641
ACE	Ace	LOC101387731	GLRX2	Glrx2	LOC101387493	PRKAG3	Prkag3	LOC101389356
ACE2	Ace2	LOC101395957	GLRX3	Glrx3	LOC101397009	PRKAR1A	Prkar1a	LOC101402286
ACER1	Acer1	LOC101406498	GLS	Gls	LOC101408026	PRKAR1B	Prkar1b	LOC101399954
ACER2	Acer2	LOC101404122	GLS2	Gls2	LOC101405945	PRKAR2A	Prkar2a	LOC101394349
ACER3	Acer3	LOC101406562	GLT1D1	Glt1d1	LOC101405420	PRKAR2B	Prkar2b	LOC101406072
ACHE	Ache	LOC101399446	GLT6D1	Glt6d1	LOC101389689	PRKCA	Prkca	LOC101399048
ACIN1	Acin1	LOC101406690	GLT8D1	Glt8d1	LOC101405292	PRKCB	Prkcb	LOC101392856
ACKR1	Ackr1	LOC101401643	GLT8D2	Glt8d2	LOC101405362	PRKCD	Prkcd	LOC101407822
ACLY	Acly	LOC101403241	GLTP	Gltp	LOC101394472	PRKCE	Prkce	LOC101398699
ACMSD	Acmsd	LOC101389194	GLTPD2	Gltpd2	LOC101395164	PRKCG	Prkcg	LOC101404588
ACO1	Aco1	LOC101396015	GLUD1	Glud1	LOC101389991	PRKCH	Prkch	LOC101396708
ACO2	Aco2	LOC101405627	GLUL	Glul	LOC101405747	PRKCI	Prkci	LOC101394457
ACOD1	Acod1	LOC101388649	GLYAT	Glyat	LOC101403786	PRKCQ	Prkcq	LOC101401310
ACOT1	Acot2	LOC101400162	GLYATL3	Glyatl3	LOC101397238	PRKCSH	Prkcsh	LOC101395899
ACOT11	Acot11	LOC101395791	GLYCTK	Glyctk	LOC101401537	PRKCZ	Prkcz	LOC101395319
ACOT12	Acot12	LOC101402234	GLYR1	Glyr1	LOC101391519	PRKD1	Prkd1	LOC101407738
ACOT13	Acot13	LOC101394481	GM2A	Gm2a	LOC101396802	PRKD2	Prkd2	LOC101404061
ACOT4	Acot4	LOC101400867	GMCL1	Gmcl1	LOC101401513	PRKD3	Prkd3	LOC101406159
ACOT6	Acot6	LOC101390313	GMDS	Gmds	LOC101404601	PRKDC	Prkdc	LOC101393218
ACOT7	Acot7	LOC101401576	GMEB1	Gmeb1	LOC101388405	PRKG1	Prkg1	LOC101401443
ACOT8	Act8	LOC101405507	GMEB2	Gmeb2	LOC101393142	PRKG2	Prkg2	LOC101391746
ACOT9	Acot9	LOC101407707	GMFB	Gmfb	LOC101406970	PRKN	Park2	LOC101393677
ACOX1	Acox1	LOC101399574	GMFG	Gmfg	LOC101387095	PRKRA	Prkra	LOC101406193
ACOX2	Acox2	LOC101398152	GMIP	Gmip	LOC101400762	PRKRIP1	Prkrip1	LOC101408609
ACOX3	Acox3	LOC101401333	GMNC	Gmnc	LOC101407325	PRL	Prl	LOC101397071
ACOXL	Acoxl	LOC101398376	GMNN	Gmnn	LOC101393985	PRLHR	Prlhr	LOC101395667
ACP1	Acp1	LOC101399323	GMPPA	Gmppa	LOC101397409	PRLR	Prlr	LOC101400759
ACP2	Acp2	LOC101402209	GMPPB	Gmppb	LOC101405197	PRMT1	Prmt1	LOC101407653
ACP5	Acp5	LOC101398162	GMPR	Gmpr	LOC101401846	PRMT2	Prmt2	LOC101389608

ACP6	Acp6	LOC101390197	GMPR2	Gmpr2	LOC101394025	PRMT3	Prmt3	LOC101394682
ACP7	Acp7	LOC101407996	GMPS	Gmps	LOC101404522	PRMT5	Prmt5	LOC101409036
ACPP	Acpp	LOC101392997	GNA11	Gna11	LOC101401977	PRMT6	Prmt6	LOC101388460
ACR	Acr	LOC101403435	GNA13	Gna13	LOC101400886	PRMT7	Prmt7	LOC101399820
ACRBP	Acrbp	LOC101403864	GNA14	Gna14	LOC101406418	PRMT8	Prmt8	LOC101390690
ACRV1	Acrv1	LOC101396486	GNA15	Gna15	LOC101401711	PRMT9	Prmt9	LOC101399698
ACSBG1	Acsbg1	LOC101407216	GNAI1	Gnai1	LOC101392831	PROB1	Prob1	LOC101388210
ACSBG2	Acsbg2	LOC101406756	GNAI2	Gnai2	LOC101388115	PROC	Proc	LOC101400684
ACSF2	Acsf2	LOC101387480	GNAI3	Gnai3	LOC101402405	PROCA1	Proca1	LOC101394921
ACSF3	Acsf3	LOC101407187	GNAL	Gnal	LOC101390374	PROCR	Procr	LOC101396200
ACSL1	Acsl1	LOC101406294	GNAO1	Gnao1	LOC101387727	PRODH2	Prodh2	LOC101399334
ACSL3	Acsl3	LOC101402695	GNAQ	Gnaq	LOC101406162	PROK1	Prok1	LOC101395293
ACSL4	Acsl4	LOC101395028	GNAS	Gnas	LOC101403929	PROK2	Prok2	LOC101387255
ACSL5	Acsl5	LOC101409155	GNAT1	Gnat1	LOC101387590	PROKR1	Prokr1	LOC101404994
ACSL6	Acsl6	LOC101404408	GNAT2	Gnat2	LOC101401983	PROKR2	Prokr2	LOC101402978
ACSM1	Acsm1	LOC106800136	GNAT3	Gnat3	LOC101393244	PROM1	Prom1	LOC101406652
ACSM3	Acsm3	LOC101394033	GNAZ	Gnaz	LOC101387868	PROM2	Prom2	LOC101400729
ACSM5	Acsm5	LOC101401552	GNB1	Gnb1	LOC101394810	PROP1	Prop1	LOC101396370
ACSS1	Acss1	LOC101401984	GNB1L	Gnb1l	LOC101394027	PRORP	1110008L16R	LOC101390683
ACSS2	Acss2	LOC101397926	GNB2	Gnb2	LOC101396779	PROS1	Pros1	LOC101400827
ACSS3	Acss3	LOC101396664	GNB3	Gnb3	LOC101399419	PROSER2	Proser2	LOC101397656
ACTA1	Acta1	LOC101394053	GNB4	Gnb4	LOC101406628	PROSER3	Proser3	LOC101393091
ACTA2	Acta2	LOC101395975	GNB5	Gnb5	LOC101388216	PROX1	Prox1	LOC101392940
ACTB	Actb	LOC101391786	GNE	Gne	LOC101394888	PROX2	Prox2	LOC101407329
ACTBL2	Actbl2	LOC101406620	GNG10	Gng10	LOC106800723	PRPF18	Prpf18	LOC101393880
ACTC1	Actc1	LOC101406096	GNG11	Gng11	LOC101402101	PRPF19	Prpf19	LOC101387575
ACTG1	Actg1	LOC101394920	GNG12	Gng12	LOC101392364	PRPF3	Prpf3	LOC101391260
ACTG2	Actg2	LOC101408837	GNG13	Gng13	LOC101399416	PRPF31	Prpf31	LOC101406838
ACTL10	Actl10	LOC106803880	GNG3	Gng3	LOC101401613	PRPF38A	Prpf38a	LOC101402907
ACTL6A	Actl6a	LOC101406187	GNG4	Gng4	LOC101393315	PRPF38B	Prpf38b	LOC101408781
ACTL6B	Actl6b	LOC101396514	GNG5	Gng5	LOC101408741	PRPF39	Prpf39	LOC101403249
ACTL7A	Actl7a	LOC101407935	GNG8	Gng8	LOC101394012	PRPF4	Prpf4	LOC101395309
ACTL7B	Actl7b	LOC101407399	GNGT1	Gngt1	LOC101401843	PRPF40A	Prpf40a	LOC101398175
ACTN1	Actn1	LOC101388479	GNGT2	Gngt2	LOC101400983	PRPF40B	Prpf40b	LOC101404968
ACTN2	Actn2	LOC101395684	GNL1	Gnl1	LOC101388227	PRPF4B	Prpf4b	LOC101393467
ACTN3	Actn3	LOC101398046	GNL2	Gnl2	LOC101396271	PRPF6	Prpf6	LOC101389371
ACTN4	Actn4	LOC101404250	GNL3	Gnl3	LOC101405025	PRPF8	Prpf8	LOC101402618
ACTR10	Actr10	LOC101390817	GNL3L	Gnl3l	LOC101399452	PRPH	Prph	LOC101402012
ACTR1A	Actr1a	LOC101392085	GNMT	Gnmt	LOC101388056	PRPH2	Prph2	LOC101389964
ACTR1B	Actr1b	LOC101401252	GNPAT	Gnpat	LOC101388420	PRPS1	Prps1	LOC101388977
ACTR2	Actr2	LOC101408838	GNPDA1	Gnpda1	LOC101404861	PRPS1L1	Prps1l1	LOC101407453
ACTR3	Actr3	LOC101390473	GNPDA2	Gnpda2	LOC101402914	PRPS2	Prps2	LOC101388003
ACTR3B	Actr3b	LOC101403407	GNPNAT1	Gnpnat1	LOC101403914	PRPSAP1	Prpsap1	LOC101404210
ACTR5	Actr5	LOC101400355	GNPTAB	Gnptab	LOC101400822	PRPSAP2	Prpsap2	LOC101394311
ACTR6	Actr6	LOC101397448	GNPTG	Gnptg	LOC101395966	PRR11	Prr11	LOC101388345
ACTR8	Actr8	LOC101388287	GNRH1	Gnrh1	LOC101407792	PRR12	Prr12	LOC101402311
ACTRT1	Actrt1	LOC101393915	GNRHR	Gnrhr	LOC101400209	PRR14	Prr14	LOC101399076
ACTRT2	Actrt2	LOC101396887	GNS	Gns	LOC101408036	PRR14L	Prr14l	LOC101387721
ACTRT3	Actrt3	LOC101396625	GOLGA1	Golga1	LOC101401123	PRR15L	Prr15l	LOC101392972
ACVR1	Acvr1	LOC101398948	GOLGA2	Golga2	LOC101394973	PRR16	Prr16	LOC101394021
ACVR1B	Acvr1b	LOC101397500	GOLGA3	Golga3	LOC101408038	PRR19	Prr19	LOC101403357
ACVR1C	Acvr1c	LOC101399206	GOLGA4	Golga4	LOC101399770	PRR22	Prr22	LOC101409114
ACVR2A	Acvr2a	LOC101403742	GOLGA5	Golga5	LOC101401507	PRR29	Prr29	LOC101388587
ACVR2B	Acvr2b	LOC101404242	GOLGA7	Golga7	LOC101389245	PRR3	Prr3	LOC101388475

ACVRL1	Acvrl1	LOC101397252	GOLGA7B	Golga7b	LOC101408382	PRR30	Prr30	LOC101397204
ACY1	Acy1	LOC101398150	GOLGB1	Golgb1	LOC101399588	PRR32	Prr32	LOC101394165
ACY3	Acy3	LOC101387576	GOLIM4	Golim4	LOC101398179	PRR36	Prr36	LOC101390963
ADA	Ada	LOC101391497	GOLM1	Golm1	LOC101391698	PRR5	Prr5	LOC106799949
ADAD1	Adad1	LOC101408288	GOLPH3	Golph3	LOC101395822	PRR5L	Prr5l	LOC101387663
ADAD2	Adad2	LOC101394245	GOLPH3L	Golph3l	LOC101393831	PRR7	Prr7	LOC101400168
ADAL	Adal	LOC101406611	GOLT1A	Golt1a	LOC101403746	PRR9	Prr9	LOC101389636
ADAM10	Adam10	LOC101396156	GOLT1B	Golt1b	LOC101407179	PRRC1	Prrc1	LOC101408949
ADAM11	Adam11	LOC101395944	GON4L	Gon4l	LOC101397523	PRRC2A	Prrc2a	LOC101399112
ADAM12	Adam12	LOC101390602	GON7	AK010878	LOC101402810	PRRC2B	Prrc2b	LOC101390305
ADAM15	Adam15	LOC101387483	GOPC	Gopc	LOC101408530	PRRC2C	Prrc2c	LOC101403655
ADAM17	Adam17	LOC101404999	GORAB	Gorab	LOC101396966	PRRG1	Prrg1	LOC101390341
ADAM18	Adam18	LOC101398725	GORASP1	Gorasp1	LOC101407116	PRRG2	Prrg2	LOC106803519
ADAM19	Adam19	LOC101408984	GORASP2	Gorasp2	LOC101399294	PRRG3	Prrg3	LOC101405740
ADAM2	Adam2	LOC101387766	GOSR1	Gosr1	LOC101408997	PRRG4	Prrg4	LOC101404058
ADAM22	Adam22	LOC101392363	GOSR2	Gosr2	LOC101402107	PRRT1	Prrt1	LOC101387966
ADAM23	Adam23	LOC101400512	GOT1	Got1	LOC101396978	PRRT2	Prrt2	LOC101395275
ADAM28	Adam28	LOC101395168	GOT1L1	Got1l1	LOC101389605	PRRT3	Prrt3	LOC101395562
ADAM32	Adam32	LOC101402699	GOT2	Got2	LOC101400176	PRRT4	Prrt4	LOC101399689
ADAM33	Adam33	LOC101404035	GP5	Gp5	LOC101406528	PRRX1	Prrx1	LOC101404881
ADAM7	Adam7	LOC101394660	GP6	Gp6	LOC101388967	PRRX2	Prrx2	LOC101404541
ADAM8	Adam8	LOC101388014	GPA33	Gpa33	LOC101400598	PRSS12	Prss12	LOC101391911
ADAM9	Adam9	LOC101395148	GPAA1	Gpaa1	LOC101391897	PRSS2	Prss2	LOC101404642
ADAMDEC1	Adamdec1	LOC101394924	GPALPP1	Gpalpp1	LOC101406811	PRSS22	Prss22	LOC101393745
ADAMTS1	Adamts1	LOC101394726	GPAM	Gpam	LOC101408472	PRSS27	Prss27	LOC101405628
ADAMTS10	Adamts10	LOC101401385	GPANK1	Gpank1	LOC101398088	PRSS36	Prss36	LOC101405805
ADAMTS12	Adamts12	LOC101397560	GPAT2	Gpat2	LOC101396741	PRSS37	Prss37	LOC101406391
ADAMTS13	Adamts13	LOC101408461	GPAT3	Agpat9	LOC101397675	PRSS38	Prss38	LOC101406857
ADAMTS14	Adamts14	LOC101401737	GPAT4	Gpat4	LOC101389935	PRSS50	Prss50	LOC101403979
ADAMTS15	Adamts15	LOC101408237	GPATCH1	Gpatch1	LOC101405807	PRSS53	Prss53	LOC101401779
ADAMTS16	Adamts16	LOC101392312	GPATCH11	Gpatch11	LOC101407447	PRSS54	Prss54	LOC101399042
ADAMTS17	Adamts17	LOC101391500	GPATCH2	Gpatch2	LOC101394862	PRSS55	Prss55	LOC101396214
ADAMTS18	Adamts18	LOC101402465	GPATCH2L	Gpatch2l	LOC101390735	PRSS56	Prss56	LOC101404631
ADAMTS19	Adamts19	LOC101407660	GPATCH3	Gpatch3	LOC101398348	PRSS57	Prss57	LOC101391537
ADAMTS2	Adamts2	LOC101387981	GPATCH4	Gpatch4	LOC101407974	PRSS58	Prss58	LOC101405682
ADAMTS20	Adamts20	LOC101406979	GPATCH8	Gpatch8	LOC101394919	PRSS8	Prss8	LOC101402046
ADAMTS3	Adamts3	LOC101391616	GPBP1	Gpbp1	LOC101405845	PRTG	Prtg	LOC101391545
ADAMTS4	Adamts4	LOC101397042	GPBP1L1	Gpbp1l1	LOC101398075	PRTN3	Prtn3	LOC101390534
ADAMTS5	Adamts5	LOC101394989	GPC1	Gpc1	LOC101408808	PRUNE1	Prune	LOC101396566
ADAMTS6	Adamts6	LOC101391713	GPC2	Gpc2	LOC101391708	PRUNE2	Prune2	LOC101390943
ADAMTS7	Adamts7	LOC101408527	GPC3	Gpc3	LOC101404660	PRX	Prx	LOC101389071
ADAMTS8	Adamts8	LOC101397530	GPC4	Gpc4	LOC101405534	PSAP	Psap	LOC101398859
ADAMTS9	Adamts9	LOC101403716	GPC6	Gpc6	LOC101394203	PSAPL1	Psapl1	LOC106802148
ADAMTSL1	Adamtsl1	LOC101405893	GPCPD1	Gpcpd1	LOC101402029	PSAT1	Psat1	LOC101405448
ADAMTSL2	Adamtsl2	LOC101398495	GPD1	Gpd1	LOC101408813	PSCA	Psca	LOC101406549
ADAMTSL3	Adamtsl3	LOC101393222	GPD1L	Gpd1l	LOC101391121	PSD	Psd	LOC101390565
ADAMTSL4	Adamtsl4	LOC101392710	GPD2	Gpd2	LOC101400784	PSD2	Psd2	LOC101397292
ADAMTSL5	Adamtsl5	LOC101387690	GPHA2	Gpha2	LOC101398805	PSD3	Psd3	LOC101397626
ADAP1	Adap1	LOC101399436	GPHB5	Gphb5	LOC101398938	PSD4	Psd4	LOC101387316
ADAP2	Adap2	LOC101393267	GPHN	Gphn	LOC101405664	PSEN1	Psen1	LOC101398350
ADAR	Adar	LOC101405962	GPI	Gpi1	LOC101408071	PSEN2	Psen2	LOC101387938
ADARB1	Adarb1	LOC101408476	GPIHBP1	Gpihbp1	LOC101407471	PSENEN	Psenen	LOC101396932
ADAT1	Adat1	LOC101404313	GPKOW	Gpkow	LOC101405434	PSIP1	Psip1	LOC101408156
ADAT2	Adat2	LOC101394948	GPLD1	Gpld1	LOC101396024	PSKH1	Pskh1	LOC101395857

ADAT3	Adat3	LOC101408346	GPM6A	Gpm6a	LOC101399296	PSMA1	Psma1	LOC101402303
ADCK1	Adck1	LOC101397406	GPM6B	Gpm6b	LOC101390763	PSMA2	Psma2	LOC101407274
ADCK2	Adck2	LOC101408737	GPN1	Gpn1	LOC101399331	PSMA3	Psma3	LOC101391061
ADCK5	Adck5	LOC101389766	GPN2	Gpn2	LOC101398096	PSMA4	Psma4	LOC101405133
ADCY1	Adcy1	LOC101402124	GPN3	Gpn3	LOC101399818	PSMA5	Psma5	LOC101404069
ADCY10	Adcy10	LOC101399023	GPNMB	Gpnmb	LOC101408858	PSMA6	Psma6	LOC101390258
ADCY2	Adcy2	LOC101396586	GPR101	Gpr101	LOC101408867	PSMA7	Psma7	LOC101405421
ADCY3	Adcy3	LOC101394679	GPR107	Gpr107	LOC101408722	PSMA8	Psma8	LOC101396413
ADCY4	Adcy4	LOC101391706	GPR108	Gpr108	LOC101403270	PSMB1	Psmb1	LOC101404425
ADCY5	Adcy5	LOC101403390	GPR119	Gpr119	LOC101388270	PSMB10	Psmb10	LOC101396113
ADCY6	Adcy6	LOC101396722	GPR12	Gpr12	LOC101407970	PSMB11	Psmb11	LOC101407572
ADCY7	Adcy7	LOC101402708	GPR137	Gpr137	LOC101392460	PSMB2	Psmb2	LOC101389349
ADCY8	Adcy8	LOC101391747	GPR137B	Gpr137b	LOC101394068	PSMB3	Psmb3	LOC101387479
ADCY9	Adcy9	LOC101397702	GPR137C	Gpr137c	LOC101404627	PSMB4	Psmb4	LOC101403339
ADCYAP1	Adcyap1	LOC101406429	GPR139	Gpr139	LOC101389669	PSMB5	Psmb5	LOC101407835
ADCYAP1R1	Adcyap1r1	LOC101406163	GPR142	Gpr142	LOC101393468	PSMB6	Psmb6	LOC101395426
ADD1	Add1	LOC101398197	GPR143	Gpr143	LOC101404506	PSMB7	Psmb7	LOC101399108
ADD2	Add2	LOC101398040	GPR149	Gpr149	LOC101405993	PSMB8	Psmb8	LOC101403564
ADD3	Add3	LOC101404189	GPR15	Gpr15	LOC101404852	PSMB9	Psmb9	LOC101403041
ADGB	Adgb	LOC101390193	GPR151	Gpr151	LOC101398398	PSMC1	Psmc1	LOC101396216
ADGRA1	Adgra1	LOC101388519	GPR153	Gpr153	LOC101401046	PSMC2	Psmc2	LOC101389406
ADGRA2	Adgra2	LOC101388668	GPR155	Gpr155	LOC101397573	PSMC3	Psmc3	LOC101405700
ADGRA3	Adgra3	LOC101395509	GPR156	Gpr156	LOC101402629	PSMC3IP	Psmc3ip	LOC101395862
ADGRB1	Adgrb1	LOC101408042	GPR157	Gpr157	LOC101403135	PSMC4	Psmc4	LOC101391792
ADGRB2	Adgrb2	LOC101395493	GPR158	Gpr158	LOC101401309	PSMC5	Psmc5	LOC101392445
ADGRB3	Adgrb3	LOC101396700	GPR161	Gpr161	LOC101388476	PSMC6	Psmc6	LOC101403220
ADGRD1	Adgrd1	LOC101399301	GPR162	Gpr162	LOC101399935	PSMD1	Psmd1	LOC101391066
ADGRE1	Adgre1	LOC101394692	GPR173	Gpr173	LOC101405317	PSMD10	Psmd10	LOC101391959
ADGRE5	Adgre5	LOC101399788	GPR176	Gpr176	LOC101387953	PSMD11	Psmd11	LOC101407570
ADGRF1	Adgrf1	LOC101389963	GPR179	Gpr179	LOC101389388	PSMD12	Psmd12	LOC101397592
ADGRF2	Adgrf2	LOC101397749	GPR18	Gpr18	LOC101400502	PSMD13	Psmd13	LOC101387594
ADGRF3	Adgrf3	LOC101397451	GPR180	Gpr180	LOC101394971	PSMD14	Psmd14	LOC101393377
ADGRF4	Adgrf4	LOC101389196	GPR183	Gpr183	LOC101400765	PSMD2	Psmd2	LOC101392673
ADGRF5	Adgrf5	LOC101390222	GPR20	Gpr20	LOC101407960	PSMD3	Psmd3	LOC101398794
ADGRG1	Adgrg1	LOC101398280	GPR21	Gpr21	LOC101397650	PSMD4	Psmd4	LOC101400989
ADGRG2	Adgrg2	LOC101401337	GPR22	Gpr22	LOC101405634	PSMD5	Psmd5	LOC101403036
ADGRG3	Adgrg3	LOC101398525	GPR26	Gpr26	LOC101403702	PSMD6	Psmd6	LOC101402656
ADGRG4	Adgrg4	LOC101408349	GPR3	Gpr3	LOC101402004	PSMD7	Psmd7	LOC101406136
ADGRG5	Adgrg5	LOC101398025	GPR33	Gpr33	LOC101404608	PSMD8	Psmd8	LOC101402237
ADGRG6	Adgrg6	LOC101394026	GPR37	Gpr37	LOC101401269	PSMD9	Psmd9	LOC101406796
ADGRG7	Adgrg7	LOC101394254	GPR37L1	Gpr37l1	LOC101398630	PSME1	Psme1	LOC101397126
ADGRL1	Adgrl1	LOC101390201	GPR39	Gpr39	LOC101390220	PSME2	Psme2	LOC101396862
ADGRL2	Adgrl2	LOC101405168	GPR45	Gpr45	LOC101390169	PSME3	Psme3	LOC101390586
ADGRL3	Adgrl3	LOC101397287	GPR50	Gpr50	LOC101395215	PSME4	Psme4	LOC101387238
ADGRL4	Adgrl4	LOC101404899	GPR52	Gpr52	LOC101397566	PSMF1	Psmf1	LOC101396555
ADGRV1	Adgrv1	LOC101395033	GPR6	Gpr6	LOC101396162	PSMG1	Psmg1	LOC101393808
ADH1C	Adh1	LOC101394066	GPR62	Gpr62	LOC101388628	PSMG2	Psmg2	LOC101397815
ADH4	Adh4	LOC101393311	GPR65	Gpr65	LOC101401213	PSMG3	Psmg3	LOC101397374
ADH5	Adh5	LOC101403672	GPR75	Gpr75	LOC106799943	PSMG4	Psmg4	LOC101394482
ADHFE1	Adhfe1	LOC101400099	GPR82	Gpr82	LOC101404991	PSORS1C2	Psors1c2	LOC101403389
ADI1	Adi1	LOC101401164	GPR83	Gpr83	LOC101396798	PSPC1	Pspc1	LOC101397601
ADIG	Adig	LOC101401146	GPR84	Gpr84	LOC101397667	PSPH	Psph	LOC101389486
ADIPOQ	Adipoq	LOC101402589	GPR85	Gpr85	LOC101393672	PSPN	Pspn	LOC101405719
ADIPOR1	Adipor1	LOC101395969	GPR87	Gpr87	LOC101407204	PSRC1	Psrc1	LOC101404516

ADIPOR2	Adipor2	LOC101397278	GPR88	Gpr88	LOC101392847	PSTK	Pstk	LOC101405447
ADK	Adk	LOC101407241	GPR89B	Gpr89	LOC101388717	PSTPIP1	Pstpip1	LOC101392665
ADM	Adm	LOC101394335	GPRC5A	Gprc5a	LOC101395176	PSTPIP2	Pstpip2	LOC101405215
ADNP	Adnp	LOC101391923	GPRC5B	Gprc5b	LOC101389940	PTAR1	Ptar1	LOC101390103
ADNP2	Adnp2	LOC101400757	GPRC5C	Gprc5c	LOC101407962	PTBP1	Ptbp1	LOC101392138
ADORA1	Adora1	LOC101394501	GPRC5D	Gprc5d	LOC101395439	PTBP2	Ptbp2	LOC101398472
ADORA2A	Adora2a	LOC101388813	GPRC6A	Gprc6a	LOC101402069	PTBP3	Ptbp3	LOC101392251
ADORA2B	Adora2b	LOC101399575	GPRIN3	Gprin3	LOC101408648	PTCD1	Ptcd1	LOC101404142
ADORA3	Adora3	LOC101391035	GPS1	Gps1	LOC101401755	PTCD2	Ptcd2	LOC101401107
ADPGK	Adpgk	LOC101408704	GPS2	Gps2	LOC101407423	PTCD3	Ptcd3	LOC101401082
ADPRH	Adprh	LOC101405443	GPSM1	Gpsm1	LOC101404173	PTCH1	Ptch1	LOC101393759
ADPRHL1	Adprhl1	LOC101390468	GPSM2	Gpsm2	LOC101407997	PTCH2	Ptch2	LOC101393007
ADPRHL2	Adprhl2	LOC101391157	GPSM3	Gpsm3	LOC101408016	PTCHD1	Ptchd1	LOC101407176
ADPRM	Adprm	LOC101406558	GPT	Gpt	LOC101387273	PTCHD3	Ptchd3	LOC101394898
ADRA1B	Adra1b	LOC101405673	GPT2	Gpt2	LOC101403118	PTCHD4	Ptchd4	LOC101388656
ADRA1D	Adra1d	LOC101403508	GPX1	Gpx1	LOC101401536	PTCRA	Ptcra	LOC101388561
ADRA2A	Adra2a	LOC101408211	GPX2	Gpx2	LOC101403300	PTDSS1	Ptdss1	LOC101402798
ADRA2B	Adra2b	LOC106802741	GPX3	Gpx3	LOC101397578	PTDSS2	Ptdss2	LOC101406754
ADRB2	Adrb2	LOC101405603	GPX4	Gpx4	LOC101389422	PTEN	Pten	LOC101399337
ADRM1	Adrm1	LOC101399038	GPX5	Gpx5	LOC101389073	PTER	Pter	LOC101389451
ADSL	Adsl	LOC101400282	GPX6	Gpx6	LOC101389500	PTF1A	Ptf1a	LOC101403214
ADSS1	Adssl1	LOC101402033	GPX7	Gpx7	LOC101409013	PTGDR	Ptgdr	LOC101395087
ADSS2	Adss	LOC101402224	GPX8	Gpx8	LOC101400499	PTGDS	Ptgds	LOC101387279
ADTRP	Adtrp	LOC101406727	GRAMD1A	Gramd1a	LOC101398143	PTGER1	Ptger1	LOC101400049
AEBP1	Aebp1	LOC101400819	GRAMD1B	Gramd1b	LOC101405498	PTGER2	Ptger2	LOC101394844
AEBP2	Aebp2	LOC101404656	GRAMD1C	Gramd1c	LOC101389140	PTGER3	Ptger3	LOC101396462
AEN	Aen	LOC101400884	GRAMD2A	Gramd2	LOC101390633	PTGER4	Ptger4	LOC101388729
AFAP1	Afap1	LOC101401848	GRAMD2B	Gramd3	LOC101389497	PTGES	Ptges	LOC101406275
AFAP1L1	Afap1l1	LOC101404634	GRAMD4	Gramd4	LOC101404050	PTGES2	Ptges2	LOC101393030
AFAP1L2	Afap1l2	LOC101390908	GRAP2	Grap2	LOC101399151	PTGES3	Ptges3	LOC101407510
AFDN	Mllt4	LOC101392131	GRAPL	Grap	LOC101395947	PTGES3L	Ptges3l	LOC106802519
AFF1	Aff1	LOC101401589	GRASP	Grasp	LOC101388671	PTGFR	Ptgfr	LOC101404205
AFF2	Aff2	LOC101398651	GRB10	Grb10	LOC101398304	PTGFRN	Ptgfrn	LOC101403370
AFF3	Aff3	LOC101406915	GRB14	Grb14	LOC101389601	PTGIS	Ptgis	LOC101395154
AFF4	Aff4	LOC101398321	GRB2	Grb2	LOC101394151	PTGR1	Ptgr1	LOC101390811
AFG1L	Lace1	LOC101391633	GRB7	Grb7	LOC101401765	PTGR2	Ptgr2	LOC101401918
AFG3L2	Afg3l2	LOC101391704	GREB1	Greb1	LOC101388603	PTGS1	Ptgs1	LOC101387194
AFM	Afm	LOC101403007	GREB1L	Greb1l	LOC101387430	PTGS2	Ptgs2	LOC101394896
AFMID	Afmid	LOC101388422	GREM1	Grem1	LOC101404523	PTH	Pth	LOC101400646
AFP	Afp	LOC101392580	GRHL1	Grhl1	LOC101405698	PTH1R	Pth1r	LOC101406082
AFTPH	Aftph	LOC101388264	GRHL2	Grhl2	LOC101388677	PTH2	Pth2	LOC101402916
AGA	Aga	LOC101401480	GRHL3	Grhl3	LOC101407943	PTH2R	Pth2r	LOC101404354
AGAP1	Agap1	LOC101401476	GRHPR	Grhpr	LOC101408459	PTHLH	Pthlh	LOC101395702
AGAP2	Agap2	LOC101400704	GRIA1	Gria1	LOC101393548	PTK2	Ptk2	LOC101408820
AGAP3	Agap3	LOC101407870	GRIA2	Gria2	LOC101390538	PTK2B	Ptk2b	LOC101402977
AGBL1	Agbl1	LOC101397588	GRIA3	Gria3	LOC101397003	PTK6	Ptk6	LOC101393893
AGBL2	Agbl2	LOC101396745	GRIA4	Gria4	LOC101405497	PTK7	Ptk7	LOC101405059
AGBL3	Agbl3	LOC101398867	GRID1	Grid1	LOC101393383	PTMA	Ptma	LOC101392168
AGBL4	Agbl4	LOC101402125	GRID2	Grid2	LOC101387725	PTMS	Ptms	LOC101400384
AGBL5	Agbl5	LOC101409099	GRID2IP	Grid2ip	LOC101389841	PTN	Ptn	LOC101392886
AGER	Ager	LOC101408538	GRIFIN	Grifin	LOC101405716	PTOV1	Ptov1	LOC101403360
AGFG1	Agfg1	LOC101408209	GRIK1	Grik1	LOC101397842	PTP4A1	Ptp4a1	LOC101397563
AGFG2	Agfg2	LOC101394273	GRIK2	Grik2	LOC101406261	PTP4A2	Ptp4a2	LOC101395751
AGGF1	Aggf1	LOC101398728	GRIK3	Grik3	LOC101394811	PTP4A3	Ptp4a3	LOC101407517

AGK	Agk	LOC101407418	GRIK4	Grik4	LOC101406534	PTPA	Ptpa	LOC101404434
AGL	Agl	LOC101395466	GRIK5	Grik5	LOC106803497	PTPDC1	Ptpdc1	LOC101398577
AGMAT	Agmat	LOC101398592	GRIN1	Grin1	LOC101388821	PTPMT1	Ptpmt1	LOC101406828
AGMO	Agmo	LOC101392694	GRIN2A	Grin2a	LOC101387577	PTPN1	Ptpn1	LOC101392176
AGO1	Ago1	LOC101390227	GRIN2B	Grin2b	LOC101396827	PTPN11	Ptpn11	LOC101394219
AGO2	Ago2	LOC101408739	GRIN2C	Grin2c	LOC101387732	PTPN12	Ptpn12	LOC101391357
AGO3	Ago3	LOC101390479	GRIN2D	Grin2d	LOC101398905	PTPN13	Ptpn13	LOC101401061
AGO4	Ago4	LOC101400430	GRIN3A	Grin3a	LOC101397743	PTPN14	Ptpn14	LOC101393203
AGPAT1	Agpat1	LOC101387707	GRIN3B	Grin3b	LOC101389498	PTPN18	Ptpn18	LOC101405399
AGPAT2	Agpat2	LOC101401569	GRINA	Grina	LOC101392666	PTPN2	Ptpn2	LOC101393346
AGPAT3	Agpat3	LOC101402792	GRIP1	Grip1	LOC101387894	PTPN21	Ptpn21	LOC101402516
AGPAT4	Agpat4	LOC101388889	GRIP2	Grip2	LOC101393275	PTPN22	Ptpn22	LOC101406501
AGPAT5	Agpat5	LOC101398466	GRIPAP1	Gripap1	LOC101407632	PTPN23	Ptpn23	LOC101408341
AGPS	Agps	LOC101403395	GRK1	Grk1	LOC101391716	PTPN3	Ptpn3	LOC101387116
AGR2	Agr2	LOC101394652	GRK2	Adrbk1	LOC101403696	PTPN4	Ptpn4	LOC101394623
AGR3	Agr3	LOC101402883	GRK3	Adrbk2	LOC101392175	PTPN5	Ptpn5	LOC101391615
AGRN	Agrn	LOC101392529	GRK4	Grk4	LOC101399661	PTPN6	Ptpn6	LOC101396232
AGRP	Agrp	LOC101391416	GRK5	Grk5	LOC101397161	PTPN7	Ptpn7	LOC101398139
AGT	Agt	LOC101390483	GRK6	Grk6	LOC101400786	PTPN9	Ptpn9	LOC101398915
AGTPBP1	Agtpbp1	LOC101390872	GRM1	Grm1	LOC101390450	PTPRA	Ptpra	LOC101389292
AGTR2	Agtr2	LOC101392494	GRM2	Grm2	LOC101395888	PTPRB	Ptprb	LOC101395930
AGTRAP	Agtrap	LOC101394978	GRM3	Grm3	LOC101389374	PTPRC	Ptprc	LOC101392571
AGXT	Agxt	LOC101387889	GRM4	Grm4	LOC101393535	PTPRCAP	Ptprcap	LOC106799924
AGXT2	Agxt2	LOC101400497	GRM5	Grm5	LOC101400457	PTPRD	Ptprd	LOC101390848
AHCTF1	Ahctf1	LOC101389408	GRM6	Grm6	LOC101394552	PTPRE	Ptpre	LOC101398138
AHCYL1	Ahcyl1	LOC101397550	GRM7	Grm7	LOC101403279	PTPRF	Ptprf	LOC101407665
AHCYL2	Ahcyl2	LOC101391776	GRM8	Grm8	LOC101400291	PTPRG	Ptprg	LOC101399170
AHDC1	Ahdc1	LOC101402691	GRN	Grn	LOC101394402	PTPRH	Ptprh	LOC101390010
AHI1	Ahi1	LOC101409130	GRP	Grp	LOC101405843	PTPRJ	Ptprj	LOC101397001
AHNAK	Ahnak	LOC101398451	GRPEL1	Grpel1	LOC101402284	PTPRK	Ptprk	LOC101397899
AHR	Ahr	LOC101408249	GRPEL2	Grpel2	LOC101404359	PTPRM	Ptprm	LOC101399265
AHRR	Ahrr	LOC101387422	GRPR	Grpr	LOC101397267	PTPRN	Ptprn	LOC101395760
AHSA1	Ahsa1	LOC101395756	GRSF1	Grsf1	LOC101389750	PTPRN2	Ptprn2	LOC101404560
AHSG	Ahsg	LOC101404702	GRTP1	Grtp1	LOC101403123	PTPRO	Ptpro	LOC101400273
AICDA	Aicda	LOC101407004	GRWD1	Grwd1	LOC101391530	PTPRQ	Ptprq	LOC101395624
AIDA	Aida	LOC101399163	GRXCR1	Grxcr1	LOC101401878	PTPRR	Ptprr	LOC101396194
AIF1	Aif1	LOC101399373	GRXCR2	Grxcr2	LOC101401794	PTPRS	Ptprs	LOC101390024
AIF1L	Aif1l	LOC101389265	GSAP	Gsap	LOC101391109	PTPRT	Ptprt	LOC101395937
AIFM1	Aifm1	LOC101389227	GSC	Gsc	LOC101407886	PTPRU	Ptpru	LOC101390977
AIFM2	Aifm2	LOC101403828	GSC2	Gsc2	LOC101407128	PTPRZ1	Ptprz1	LOC101405977
AIFM3	Aifm3	LOC101400670	GSDMA	Gsdma	LOC101399225	PTRH1	Ptrh1	LOC101389264
AIG1	Aig1	LOC101397543	GSDMC	Gsdmc	LOC101388755	PTRH2	Ptrh2	LOC101390340
AIMP1	Aimp1	LOC101405406	GSDMD	Gsdmd	LOC101406435	PTRHD1	Ptrhd1	LOC101395368
AIMP2	Aimp2	LOC101388116	GSDME	Dfna5	LOC101397802	PTS	Pts	LOC101391316
AIP	Aip	LOC101407721	GSE1	Gse1	LOC101391865	PTTG1	Pttg1	LOC101403051
AIPL1	Aipl1	LOC101404569	GSG1	Gsg1	LOC101395959	PTTG1IP	Pttg1ip	LOC101407415
AIRE	Aire	LOC101405336	GSG1L	Gsg1l	LOC101403259	PTX3	Ptx3	LOC106803737
AJAP1	Ajap1	LOC101399551	GSG1L2	Gsg1l2	LOC106802287	PTX4	Ptx4	LOC101397610
AJUBA	Ajuba	LOC101408354	GSK3A	Gsk3a	LOC101404598	PUF60	Puf60	LOC101394451
AK1	Ak1	LOC101390217	GSK3B	Gsk3b	LOC101402897	PUM1	Pum1	LOC101392346
AK2	Ak2	LOC101404803	GSKIP	Gskip	LOC101387566	PUM2	Pum2	LOC101399941
AK3	Ak3	LOC101398149	GSN	Gsn	LOC101405223	PUM3	Pum3	LOC101395458
AK4	Ak4	LOC101408561	GSPT1	Gspt1	LOC101405186	PURB	Purb	LOC101392304
AK5	Ak5	LOC101401232	GSPT2	Gspt2	LOC101407137	PURG	Purg	LOC101404816

AK6	Ak6	AK6	GSR	Gsr	LOC101404024	PUS1	Pus1	LOC101400255
AK7	Ak7	LOC106802479	GSS	Gss	LOC101397671	PUS10	Pus10	LOC101396653
AK8	Ak8	LOC101393700	GSTA5	Gm10639	LOC101394374	PUS3	Pus3	LOC101398807
AK9	Ak9	LOC101398658	GSTCD	Gstcd	LOC101406286	PUS7	Pus7	LOC101408764
AKAIN1	Akain1	LOC101395470	GSTK1	Gstk1	LOC101401935	PUS7L	Pus7l	LOC101407247
AKAP1	Akap1	LOC101402982	GSTM1	Gstm6	LOC101400038	PUSL1	Pusl1	LOC101394292
AKAP10	Akap10	LOC101400452	GSTM3	Gstm5	LOC101399092	PVALB	Pvalb	LOC101397011
AKAP11	Akap11	LOC101403851	GSTM4	Gstm4	LOC101400928	PWP1	Pwp1	LOC101389831
AKAP13	Akap13	LOC101397070	GSTO1	Gsto1	LOC101400956	PWP2	Pwp2	LOC101404295
AKAP14	Akap14	LOC106803851	GSTO2	Gsto2	LOC101401403	PWWP2A	Pwwp2a	LOC101404814
AKAP3	Akap3	LOC101388193	GSTP1	Gstp1	LOC101407897	PWWP2B	Pwwp2b	LOC101395709
AKAP4	Akap4	LOC101400984	GSTT2B	Gstt2	LOC101391804	PWWP3A	Mum1	LOC101388455
AKAP6	Akap6	LOC101403544	GSTZ1	Gstz1	LOC101393884	PXDC1	Pxdc1	LOC101393986
AKAP7	Akap7	LOC101401020	GSX1	Gsx1	LOC101387395	PXDN	Pxdn	LOC101392558
AKAP8	Akap8	LOC101395044	GSX2	Gsx2	LOC101390946	PXK	Pxk	LOC101396674
AKAP8L	Akap8l	LOC101395304	GTDC1	Gtdc1	LOC101404699	PXMP2	Pxmp2	LOC101401326
AKAP9	Akap9	LOC101396728	GTF2A1	Gtf2a1	LOC101399554	PXMP4	Pxmp4	LOC101405837
AKIP1	Akip1	LOC101390942	GTF2A1L	Gtf2a1l	LOC101391861	PXN	Pxn	LOC101395504
AKIRIN1	Akirin1	LOC101401917	GTF2A2	Gtf2a2	LOC101398837	PXYLP1	Pxylp1	LOC101399690
AKIRIN2	Akirin2	LOC101393952	GTF2B	Gtf2b	LOC101404074	PYCARD	Pycard	LOC101406486
AKNA	Akna	LOC101399107	GTF2E1	Gtf2e1	LOC101401076	PYCR1	Pycr1	LOC101400003
AKNAD1	Aknad1	LOC101408090	GTF2E2	Gtf2e2	LOC101403309	PYCR2	Pycr2	LOC101398815
AKR1A1	Akr1a1	LOC101396414	GTF2F1	Gtf2f1	LOC101405461	PYCR3	Pycrl	LOC101398920
AKR1B1	Akr1b3	LOC101401231	GTF2F2	Gtf2f2	LOC101407080	PYGB	Pygb	LOC101395206
AKR1B10	Akr1b8	LOC101400084	GTF2H1	Gtf2h1	LOC101387753	PYGL	Pygl	LOC101396743
AKR1B15	Akr1b7	LOC101399393	GTF2H3	Gtf2h3	LOC101395070	PYGM	Pygm	LOC101395526
AKR1C1	Akr1c12	LOC101405151	GTF2H4	Gtf2h4	LOC101404438	PYGO1	Pygo1	LOC101391295
AKR1D1	Akr1d1	LOC101392007	GTF2H5	Gtf2h5	LOC101388626	PYGO2	Pygo2	LOC101407182
AKR1E2	Akr1e1	LOC101408467	GTF2I	Gtf2i	LOC101397976	PYM1	Pym1	LOC101395068
AKR7A2	Akr7a5	LOC101387282	GTF2IRD1	Gtf2ird1	LOC101398912	PYROXD1	Pyroxd1	LOC101406402
AKT1	Akt1	LOC101402979	GTF2IRD2	Gtf2ird2	LOC101397468	PYROXD2	Pyroxd2	LOC101395845
AKT1S1	Akt1s1	LOC101403620	GTF3A	Gtf3a	LOC101406912	PYURF	Pyurf	LOC101406985
AKT2	Akt2	LOC101392744	GTF3C1	Gtf3c1	LOC101396841	QDPR	Qdpr	LOC101407167
AKT3	Akt3	LOC101394761	GTF3C2	Gtf3c2	LOC101404324	QKI	Qk	LOC101389417
AKTIP	Aktip	LOC101405252	GTF3C3	Gtf3c3	LOC101403222	QPCT	Qpct	LOC101405904
ALAD	Alad	LOC101396620	GTF3C4	Gtf3c4	LOC101393289	QPCTL	Qpctl	LOC101399430
ALAS1	Alas1	LOC101399864	GTF3C5	Gtf3c5	LOC101394804	QPRT	Qprt	LOC101396051
ALAS2	Alas2	LOC101401119	GTF3C6	Gtf3c6	LOC101398337	QRFPR	Qrfpr	LOC101388482
ALB	Alb	LOC101392314	GTPBP1	Gtpbp1	LOC101395097	QRICH1	Qrich1	LOC101397806
ALCAM	Alcam	LOC101408233	GTPBP10	Gtpbp10	LOC101397677	QRICH2	Qrich2	LOC101397077
ALDH16A1	Aldh16a1	LOC101403973	GTPBP2	Gtpbp2	LOC101399193	QRSL1	Qrsl1	LOC101388219
ALDH18A1	Aldh18a1	LOC101406789	GTPBP3	Gtpbp3	LOC101395221	QSER1	Qser1	LOC101403793
ALDH1A1	Aldh1a1	LOC101388018	GTPBP4	Gtpbp4	LOC101390226	QSOX1	Qsox1	LOC101408727
ALDH1A2	Aldh1a2	LOC101394786	GTPBP6	Gtpbp6	LOC101408689	QSOX2	Qsox2	LOC101388399
ALDH1A3	Aldh1a3	LOC101390499	GTPBP8	Gtpbp8	LOC101391599	QTRT1	Qtrt1	LOC101390955
ALDH1B1	Aldh1b1	LOC101405318	GTSE1	Gtse1	LOC101403344	QTRT2	Qtrtd1	LOC101388434
ALDH1L2	Aldh1l2	LOC101389237	GTSF1	Gtsf1	LOC101398771	R3HCC1	R3hcc1	LOC101387474
ALDH2	Aldh2	LOC101396538	GTSF1L	Gtsf1l	LOC101395155	R3HCC1L	R3hcc1l	LOC101395328
ALDH3A1	Aldh3a1	LOC101405689	GUCA1A	Guca1a	LOC101401127	R3HDM1	R3hdm1	LOC101408537
ALDH3A2	Aldh3a2	LOC101401157	GUCA1B	Guca1b	LOC101390894	R3HDM2	R3hdm2	LOC101391739
ALDH3B1	Aldh3b1	LOC101388517	GUCA2A	Guca2a	LOC101407393	R3HDM4	R3hdm4	LOC101390026
ALDH3B2	Aldh3b3	LOC101387829	GUCA2B	Guca2b	LOC101403027	R3HDML	R3hdml	LOC101393309
ALDH4A1	Aldh4a1	LOC101407763	GUCD1	Gucd1	LOC101389335	RAB10	Rab10	LOC101392204
ALDH5A1	Aldh5a1	LOC101395603	GUCY1A1	Gucy1a3	LOC101388212	RAB11A	Rab11a	LOC101394115

ALDH6A1	Aldh6a1	LOC101403753	GUCY1A2	Gucy1a2	LOC101404448	RAB11B	Rab11b	LOC101403034
ALDH7A1	Aldh7a1	LOC101389069	GUCY1B1	Gucy1b3	LOC101388801	RAB11FIP1	Rab11fip1	LOC101389204
ALDH8A1	Aldh8a1	LOC101406868	GUCY2C	Gucy2c	LOC101403515	RAB11FIP2	Rab11fip2	LOC101394987
ALDH9A1	Aldh9a1	LOC101401038	GUCY2D	Gucy2e	LOC101396553	RAB11FIP3	Rab11fip3	LOC101400469
ALDOA	Aldoa	LOC101389557	GUCY2F	Gucy2f	LOC101394258	RAB11FIP4	Rab11fip4	LOC101393509
ALDOB	Aldob	LOC101396961	GUF1	Guf1	LOC101402648	RAB11FIP5	Rab11fip5	LOC101390932
ALDOC	Aldoc	LOC101396469	GUK1	Guk1	LOC101401713	RAB12	Rab12	LOC101399614
ALG1	Alg1	LOC101390088	GULP1	Gulp1	LOC101394052	RAB13	Rab13	LOC101398206
ALG11	Alg11	LOC101398290	GUSB	Gusb	LOC101390866	RAB14	Rab14	LOC101404950
ALG12	Alg12	LOC101405276	GXYLT1	Gxylt1	LOC101399035	RAB15	Rab15	LOC101403567
ALG13	Alg13	LOC101399083	GXYLT2	Gxylt2	LOC101387946	RAB17	Rab17	LOC101404020
ALG14	Alg14	LOC101400306	GYG1	Gyg	LOC101389243	RAB18	Rab18	LOC101396004
ALG2	Alg2	LOC101391810	GYS1	Gys1	LOC101398464	RAB19	Rab19	LOC101387383
ALG3	Alg3	LOC101393795	GYS2	Gys2	LOC101407710	RAB1A	Rab1a	LOC101387155
ALG5	Alg5	LOC101399319	GZF1	Gzf1	LOC101398475	RAB1B	Rab1b	LOC101393328
ALG6	Alg6	LOC101406551	GZMA	Gzma	LOC101399971	RAB20	Rab20	LOC101387439
ALG8	Alg8	LOC101387740	GZMB	Gzmb	LOC101388989	RAB21	Rab21	LOC101387409
ALG9	Alg9	LOC101396010	GZMK	Gzmk	LOC101399455	RAB22A	Rab22a	LOC101407516
ALK	Alk	LOC101394859	GZMM	Gzmm	LOC101393424	RAB23	Rab23	LOC101399110
ALKBH1	Alkbh1	LOC101396273	H1-0	H1f0	LOC101388605	RAB24	Rab24	LOC101403401
ALKBH2	Alkbh2	LOC101394913	H1-1	Hist1h1a	LOC101390071	RAB25	Rab25	LOC101401517
ALKBH3	Alkbh3	LOC101390595	H1-2	Hist1h1c	LOC101388759	RAB26	Rab26	LOC101387831
ALKBH4	Alkbh4	LOC101408093	H1-3	Hist1h1d	LOC101406804	RAB27A	Rab27a	LOC101389428
ALKBH5	Alkbh5	LOC101392369	H1-4	Hist1h1e	LOC106800113	RAB27B	Rab27b	LOC101398842
ALKBH6	Alkbh6	LOC101400290	H1-5	Hist1h1b	LOC101393181	RAB28	Rab28	LOC101409169
ALKBH7	Alkbh7	LOC101405988	H1-6	Hist1h1t	LOC106802122	RAB29	Rab29	LOC101397315
ALKBH8	Alkbh8	LOC101403916	H1-8	H1foo	LOC101401376	RAB2A	Rab2a	LOC101395779
ALLC	Allc	LOC101401688	H2AC15	Hist1h2ao	LOC101396946	RAB2B	Rab2b	LOC101397127
ALMS1	Alms1	LOC101403424	H2AC16	Hist1h2ak	LOC101395467	RAB30	Rab30	LOC101389725
ALOX12	Alox12	LOC101392370	H2AC17	Hist1h2ai	LOC101392408	RAB31	Rab31	LOC101399093
ALOX12B	Alox12b	LOC101395512	H2AC18	Hist2h2aa1	LOC106802770	RAB32	Rab32	LOC101396764
ALOX15	Alox15	LOC101392629	H2AC4	Hist1h2ab	LOC101408823	RAB33A	Rab33a	LOC101388958
ALOX5	Alox5	LOC101403323	H2AJ	H2afj	LOC101398038	RAB33B	Rab33b	LOC101398262
ALOX5AP	Alox5ap	LOC101392290	H2AW	Hist3h2a	LOC101400926	RAB34	Rab34	LOC101394657
ALOXE3	Aloxe3	LOC101395253	H2AX	H2afx	LOC101390484	RAB35	Rab35	LOC101395765
ALPI	Akp3	LOC101404356	H2AZ1	H2afz	LOC101396020	RAB36	Rab36	LOC101396956
ALPK1	Alpk1	LOC101397054	H2AZ2	H2afv	LOC101391869	RAB37	Rab37	LOC101408657
ALPK2	Alpk2	LOC101404267	H2BC1	Hist1h2ba	LOC101391843	RAB38	Rab38	LOC101399582
ALPK3	Alpk3	LOC101402450	H2BC14	Hist1h2bm	LOC101392142	RAB39A	Rab39	LOC101402434
ALPL	Alpl	LOC101395320	H2BC15	Hist1h2bq	LOC101396682	RAB39B	Rab39b	LOC101404429
ALS2	Als2	LOC101391647	H2BC17	Hist1h2bl	LOC101395204	RAB3A	Rab3a	LOC106800668
ALS2CL	Als2cl	LOC101405023	H2BC4	Hist1h2bc	LOC101387465	RAB3B	Rab3b	LOC101406229
ALX1	Alx1	LOC101399682	H3-3A	H3f3a	LOC101407986	RAB3C	Rab3c	LOC101407749
ALX3	Alx3	LOC101397028	H3-3B	H3f3b	LOC101397682	RAB3D	Rab3d	LOC101393352
ALX4	Alx4	LOC101391343	H3-4	Gm12260	LOC101405300	RAB3GAP1	Rab3gap1	LOC101387706
ALYREF	Alyref	LOC101403590	H3C1	Hist1h3a	LOC101389804	RAB3GAP2	Rab3gap2	LOC101396842
AMACR	Amacr	LOC101407224	H3C12	Hist1h3h	LOC106803747	RAB3IL1	Rab3il1	LOC101395357
AMBN	Ambn	LOC101387757	H3C13	Hist2h3b	LOC101404310	RAB3IP	Rab3ip	LOC101394823
AMBP	Ambp	LOC106800725	H3C4	Hist1h3d	LOC106800173	RAB40B	Rab40b	LOC101405085
AMBRA1	Ambra1	LOC101397192	H4-16	Hist4h4	LOC101397779	RAB40C	Rab40c	LOC101403607
AMD1	Amd1	LOC101397733	H4C1	Hist1h4a	LOC101389533	RAB42	Rab42	LOC101387537
AMDHD1	Amdhd1	LOC101389920	H4C11	Hist1h4n	LOC106803750	RAB43	Rab43	LOC101387867
AMDHD2	Amdhd2	LOC101406583	H4C4	Hist1h4d	LOC106802099	RAB44	Rab44	LOC101406378
AMELX	Amelx	LOC101408148	H6PD	H6pd	LOC101407407	RAB4A	Rab4a	LOC101393629

AMER1	Amer1	LOC101395974	HAAO	Haao	LOC101404312	RAB4B	Rab4b	LOC101397971
AMER2	Amer2	LOC101405529	HABP2	Habp2	LOC101387980	RAB5A	Rab5a	LOC101402918
AMER3	Amer3	LOC101405148	HABP4	Habp4	LOC101403623	RAB5B	Rab5b	LOC101396634
AMFR	Amfr	LOC101388167	HACD1	Hacd1	LOC101399285	RAB5C	Rab5c	LOC101400721
AMH	Amh	LOC101406168	HACD2	Hacd2	LOC101400948	RAB5IF	1110008F13R	LOC101408040
AMHR2	Amhr2	LOC101389364	HACD3	Hacd3	LOC101392056	RAB6A	Rab6a	LOC101394662
AMIGO1	Amigo1	LOC101402928	HACD4	Hacd4	LOC101402898	RAB6B	Rab6b	LOC101389562
AMIGO2	Amigo2	LOC101389017	HACE1	Hace1	LOC101406866	RAB7A	Rab7	LOC101400578
AMIGO3	Amigo3	LOC101405456	HACL1	Hacl1	LOC101398225	RAB7B	Rab7b	LOC101396525
AMMECR1	Ammecr1	LOC101395886	HADH	Hadh	LOC101404087	RAB8A	Rab8a	LOC101402770
AMMECR1L	Ammecr1l	LOC101402425	HADHA	Hadha	LOC101391774	RAB8B	Rab8b	LOC101404153
AMN	Amn	LOC101394664	HADHB	Hadhb	LOC101391356	RABAC1	Rabac1	LOC101406594
AMN1	Amn1	LOC101404768	HAGH	Hagh	LOC101392031	RABEP1	Rabep1	LOC101401237
AMOT	Amot	LOC101400296	HAGHL	Haghl	LOC101400732	RABEP2	Rabep2	LOC101405106
AMOTL1	Amotl1	LOC101395062	HAL	Hal	LOC101390353	RABEPK	Rabepk	LOC101402256
AMOTL2	Amotl2	LOC101389062	HAMP	Hamp	LOC101402132	RABGAP1	Rabgap1	LOC101397399
AMPD1	Ampd1	LOC101403627	HAND1	Hand1	LOC101391489	RABGAP1L	Rabgap1l	LOC101396268
AMPD2	Ampd2	LOC101401370	HAND2	Hand2	LOC101390504	RABGGTA	Rabggta	LOC101393511
AMPD3	Ampd3	LOC101394592	HAO1	Hao1	LOC101399747	RABGGTB	Rabggtb	LOC101399913
AMPH	Amph	LOC101399257	HAO2	Hao2	LOC101394607	RABIF	Rabif	LOC106803255
AMT	Amt	LOC101402835	HAP1	Hap1	LOC101406568	RABL3	Rabl3	LOC101401341
AMTN	Amtn	LOC101402482	HAPLN1	Hapln1	LOC101399256	RABL6	Rabl6	LOC101407593
AMY2B	Amy1	LOC101388985	HAPLN2	Hapln2	LOC101408415	RAC1	Rac1	LOC101402315
AMZ1	Amz1	LOC101395289	HAPLN3	Hapln3	LOC101400617	RAC2	Rac2	LOC101399513
AMZ2	Amz2	LOC101401156	HAPLN4	Hapln4	LOC101398249	RAC3	Rac3	LOC101400711
ANAPC1	Anapc1	LOC101392196	HARBI1	Harbi1	LOC101397607	RACGAP1	Racgap1	LOC101407689
ANAPC10	Anapc10	LOC101395036	HARS2	Hars2	LOC101390532	RACK1	Rack1	LOC101387875
ANAPC11	Anapc11	LOC101398964	HAS1	Has1	LOC101395623	RAD1	Rad1	LOC101399711
ANAPC13	Anapc13	LOC101388789	HAS2	Has2	LOC101401751	RAD17	Rad17	LOC101398157
ANAPC15	Anapc15	LOC101388951	HAS3	Has3	LOC101401753	RAD18	Rad18	LOC101401546
ANAPC16	Anapc16	LOC101397158	HAT1	Hat1	LOC101396187	RAD21	Rad21	LOC101405768
ANAPC2	Anapc2	LOC101390218	HAUS1	Haus1	LOC101407040	RAD23A	Rad23a	LOC101407032
ANAPC4	Anapc4	LOC101407961	HAUS2	Haus2	LOC101404525	RAD23B	Rad23b	LOC101406523
ANAPC5	Anapc5	LOC101403500	HAUS3	Haus3	LOC101396379	RAD50	Rad50	LOC101401278
ANAPC7	Anapc7	LOC101400875	HAUS4	Haus4	LOC101408608	RAD51	Rad51	LOC101392411
ANG	Ang	LOC101404780	HAUS5	Haus5	LOC101390014	RAD51AP1	Rad51ap1	LOC101388698
ANGEL1	Angel1	LOC101392161	HAUS6	Haus6	LOC101405353	RAD51AP2	Rad51ap2	LOC101400824
ANGEL2	Angel2	LOC101391563	HAUS7	Haus7	LOC101398244	RAD51B	Rad51b	LOC101387798
ANGPT1	Angpt1	LOC101396545	HAUS8	Haus8	LOC101403736	RAD51C	Rad51c	LOC101387392
ANGPT2	Angpt2	LOC101398987	HAVCR1	Havcr1	LOC101389363	RAD51D	Rad51d	LOC101402240
ANGPT4	Angpt4	LOC101396818	HAVCR2	Havcr2	LOC101389112	RAD52	Rad52	LOC101395876
ANGPTL1	Angptl1	LOC101391819	HAX1	Hax1	LOC101404220	RAD54L	Rad54l	LOC101400574
ANGPTL2	Angptl2	LOC101406701	HBB	Hbb-bt	LOC101391184	RAD54L2	Rad54l2	LOC101395030
ANGPTL3	Angptl3	LOC101405610	HBD	Hbb-bs	LOC101390926	RAD9A	Rad9a	LOC101405006
ANGPTL4	Angptl4	LOC101403293	HBE1	Hbb-y	LOC101390420	RAD9B	Rad9b	LOC101398357
ANGPTL6	Angptl6	LOC101402573	HBEGF	Hbegf	LOC101396245	RADIL	Radil	LOC101393680
ANGPTL7	Angptl7	LOC101391824	HBP1	Hbp1	LOC101405015	RADX	D330045A20I	LOC101408775
ANGPTL8	Angptl8	LOC101392854	HBQ1	Hbq1a	LOC101401775	RAE1	Rae1	LOC101406299
ANK1	Ank1	LOC101390195	HBS1L	Hbs1l	LOC101407473	RAF1	Raf1	LOC101387524
ANK2	Ank2	LOC101395920	HCAR1	Hcar1	LOC101402018	RAG1	Rag1	LOC101393157
ANK3	Ank3	LOC101399384	HCAR2	Hcar2	LOC101401746	RAI1	Rai1	LOC101390758
ANKAR	Ankar	LOC101396531	HCCS	Hccs	LOC101407429	RAI14	Rai14	LOC101399104
ANKDD1A	Ankdd1a	LOC101387780	HCFC1	Hcfc1	LOC101390637	RAI2	Rai2	LOC101399925
ANKDD1B	Ankdd1b	LOC101399258	HCFC1R1	Hcfc1r1	LOC101403436	RALA	Rala	LOC101396503

ANKEF1	Ankef1	LOC101397341	HCFC2	Hcfc2	LOC101405631	RALB	Ralb	LOC101395745
ANKFN1	Ankfn1	LOC101401161	HCK	Hck	LOC101389855	RALBP1	Ralbp1	LOC101397724
ANKFY1	Ankfy1	LOC101387472	HCLS1	Hcls1	LOC101400015	RALGAPA1	Ralgapa1	LOC101388774
ANKH	Ank	LOC101402522	HCN1	Hcn1	LOC101395481	RALGAPA2	Ralgapa2	LOC101399830
ANKHD1	Ankhd1	LOC101394262	HCN2	Hcn2	LOC101392844	RALGAPB	Ralgapb	LOC101401410
ANKIB1	Ankib1	LOC101398113	HCN3	Hcn3	LOC101393997	RALGDS	Ralgds	LOC101395486
ANKK1	Ankk1	LOC101390485	HCN4	Hcn4	LOC101408958	RALGPS1	Ralgps1	LOC101406962
ANKLE1	Ankle1	LOC101403207	HCRT	Hcrt	LOC101400185	RALGPS2	Ralgps2	LOC101391397
ANKLE2	Ankle2	LOC101407779	HCRTR1	Hcrtr1	LOC101394716	RALY	Raly	LOC101404527
ANKMY1	Ankmy1	LOC101387207	HCRTR2	Hcrtr2	LOC101401731	RALYL	Ralyl	LOC101396246
ANKMY2	Ankmy2	LOC101393726	HCST	Hcst	LOC101397452	RAMAC	Fam103a1	LOC101391501
ANKRA2	Ankra2	LOC101393503	HDAC1	Hdac1	LOC101400339	RAMP2	Ramp2	LOC101392452
ANKRD1	Ankrd1	LOC101392267	HDAC10	Hdac10	LOC101401616	RAMP3	Ramp3	LOC101393664
ANKRD10	Ankrd10	LOC101387963	HDAC11	Hdac11	LOC101388383	RAN	Ran	LOC101399037
ANKRD11	Ankrd11	LOC101406666	HDAC2	Hdac2	LOC101402940	RANBP1	Ranbp1	LOC101405574
ANKRD12	Ankrd12	LOC101398235	HDAC3	Hdac3	LOC101407119	RANBP10	Ranbp10	LOC101394306
ANKRD13A	Ankrd13a	LOC101399564	HDAC4	Hdac4	LOC101408809	RANBP17	Ranbp17	LOC101394820
ANKRD13B	Ankrd13b	LOC101388855	HDAC5	Hdac5	LOC101390999	RANBP2	Ranbp2	LOC101393996
ANKRD13C	Ankrd13c	LOC101394733	HDAC6	Hdac6	LOC101397187	RANBP3	Ranbp3	LOC101407459
ANKRD13D	Ankrd13d	LOC101403957	HDAC7	Hdac7	LOC101390409	RANBP3L	Ranbp3l	LOC101405142
ANKRD16	Ankrd16	LOC101402952	HDAC8	Hdac8	LOC101398714	RANBP6	Ranbp6	LOC101392899
ANKRD17	Ankrd17	LOC101391877	HDAC9	Hdac9	LOC101406164	RANBP9	Ranbp9	LOC101404465
ANKRD2	Ankrd2	LOC101391916	HDC	Hdc	LOC101403990	RANGAP1	Rangap1	LOC101403959
ANKRD22	Ankrd22	LOC101396845	HDDC2	Hddc2	LOC101393187	RANGRF	Rangrf	LOC101392282
ANKRD23	Ankrd23	LOC101400188	HDDC3	Hddc3	LOC101408399	RAP1A	Rap1a	LOC101390622
ANKRD24	Ankrd24	LOC101399610	HDGF	Hdgf	LOC101388182	RAP1B	Rap1b	LOC101390066
ANKRD26	Ankrd26	LOC101401040	HDHD2	Hdhd2	LOC101388814	RAP1GAP	Rap1gap	LOC101395918
ANKRD27	Ankrd27	LOC101407110	HDHD5	Cecr5	LOC101391105	RAP1GAP2	Rap1gap2	LOC101397180
ANKRD28	Ankrd28	LOC101398906	HDLBP	Hdlbp	LOC101389598	RAP1GDS1	Rap1gds1	LOC101403409
ANKRD29	Ankrd29	LOC101389848	HDX	Hdx	LOC101395008	RAP2A	Rap2a	LOC101397742
ANKRD31	Ankrd31	LOC101399529	HEATR1	Heatr1	LOC101395424	RAP2B	Rap2b	LOC101406256
ANKRD33	Ankrd33	LOC101396982	HEATR3	Heatr3	LOC101402447	RAP2C	Rap2c	LOC101407095
ANKRD33B	Ankrd33b	LOC101404017	HEATR4	Heatr4	LOC101389787	RAPGEF1	Rapgef1	LOC101405589
ANKRD34C	Ankrd34c	LOC101388127	HEATR5A	Heatr5a	LOC101405126	RAPGEF2	Rapgef2	LOC101394442
ANKRD35	Ankrd35	LOC101387261	HEATR5B	Heatr5b	LOC101407724	RAPGEF3	Rapgef3	LOC101390144
ANKRD37	Ankrd37	LOC101408300	HEATR6	Heatr6	LOC101401162	RAPGEF4	Rapgef4	LOC101394633
ANKRD39	Ankrd39	LOC101403080	HEATR9	Heatr9	LOC101396825	RAPGEF5	Rapgef5	LOC101402560
ANKRD40	Ankrd40	LOC101394236	HEBP1	Hebp1	LOC101395701	RAPGEF6	Rapgef6	LOC101405460
ANKRD42	Ankrd42	LOC101390583	HEBP2	Hebp2	LOC101388553	RAPGEFL1	Rapgefl1	LOC101396561
ANKRD44	Ankrd44	LOC101403755	HECA	Heca	LOC101396947	RAPH1	Raph1	LOC101394814
ANKRD46	Ankrd46	LOC101408649	HECTD1	Hectd1	LOC101405380	RAPSN	Rapsn	LOC101405964
ANKRD49	Ankrd49	LOC101396275	HECTD2	Hectd2	LOC101392946	RARA	Rara	LOC101395083
ANKRD50	Ankrd50	LOC101405597	HECTD3	Hectd3	LOC101393268	RARB	Rarb	LOC101406681
ANKRD52	Ankrd52	LOC101401223	HECTD4	Gm15800	LOC101399127	RARG	Rarg	LOC101407690
ANKRD53	Ankrd53	LOC101403951	HECW1	Hecw1	LOC101407803	RARRES1	Rarres1	LOC101397628
ANKRD54	Ankrd54	LOC101388696	HECW2	Hecw2	LOC101402957	RARRES2	Rarres2	LOC101391172
ANKRD55	Ankrd55	LOC101404079	HEG1	Heg1	LOC101403909	RARS2	Rars2	LOC101393276
ANKRD6	Ankrd6	LOC101396954	HELB	Helb	LOC101387304	RASA1	Rasa1	LOC101398323
ANKRD60	Ankrd60	LOC101408041	HELLS	Hells	LOC101401924	RASA2	Rasa2	LOC101398985
ANKRD61	Ankrd61	LOC106803618	HELQ	Helq	LOC101397170	RASA3	Rasa3	LOC101392249
ANKRD66	Ankrd66	LOC101398754	HELT	Helt	LOC101406541	RASA4B	Rasa4	LOC101406949
ANKRD7	Ankrd7	LOC101402561	HELZ	Helz	LOC101398028	RASAL1	Rasal1	LOC101392882
ANKS1A	Anks1	LOC101389778	HELZ2	Helz2	LOC101402528	RASAL2	Rasal2	LOC101392527
ANKS1B	Anks1b	LOC101395103	HEMGN	Hemgn	LOC101398341	RASAL3	Rasal3	LOC101396168

ANKS3	Anks3	LOC101392714	HEMK1	Hemk1	LOC101393340	RASD1	Rasd1	LOC101389897
ANKS4B	Anks4b	LOC101396344	HENMT1	Henmt1	LOC101408352	RASD2	Rasd2	LOC101393584
ANKS6	Anks6	LOC101391054	HEPACAM	Hepacam	LOC101395363	RASEF	Rasef	LOC101403794
ANKUB1	Ankub1	LOC101388624	HEPACAM2	Hepacam2	LOC101400085	RASGEF1A	Rasgef1a	LOC101401155
ANKZF1	Ankzf1	LOC101393544	HEPH	Heph	LOC101392727	RASGEF1B	Rasgef1b	LOC101392009
ANLN	Anln	LOC101398903	HEPHL1	Hephl1	LOC101397574	RASGEF1C	Rasgef1c	LOC101392002
ANO1	Ano1	LOC101391373	HERC1	Herc1	LOC101405575	RASGRF1	Rasgrf1	LOC101387864
ANO10	Ano10	LOC101396591	HERC2	Herc2	LOC101392594	RASGRF2	Rasgrf2	LOC101403188
ANO2	Ano2	LOC101408758	HERC3	Herc3	LOC101407256	RASGRP1	Rasgrp1	LOC101409122
ANO3	Ano3	LOC101396756	HERC4	Herc4	LOC101391730	RASGRP2	Rasgrp2	LOC101396130
ANO4	Ano4	LOC101399159	HERC6	Herc6	LOC101402608	RASGRP3	Rasgrp3	LOC101387495
ANO5	Ano5	LOC101395797	HERPUD1	Herpud1	LOC101391661	RASGRP4	Rasgrp4	LOC101403014
ANO6	Ano6	LOC101387555	HERPUD2	Herpud2	LOC101400118	RASIP1	Rasip1	LOC101399951
ANO7	Ano7	LOC101389357	HES1	Hes1	LOC101394627	RASL10A	Rasl10a	LOC101405077
ANO8	Ano8	LOC101402943	HES2	Hes2	LOC101402003	RASL10B	Rasl10b	LOC101403776
ANO9	Ano9	LOC101406496	HES3	Hes3	LOC101401311	RASL11A	Rasl11a	LOC101408489
ANP32A	Anp32a	LOC101400671	HES5	Hes5	LOC101398673	RASL11B	Rasl11b	LOC101389162
ANP32B	Anp32b	LOC101398586	HES7	Hes7	LOC101394834	RASL12	Rasl12	LOC101389255
ANP32E	Anp32e	LOC101389042	HESX1	Hesx1	LOC101392220	RASSF1	Rassf1	LOC101390702
ANPEP	Anpep	LOC101403675	HEXA	Hexa	LOC101407925	RASSF10	Rassf10	LOC101398556
ANTKMT	Fam173a	LOC101399417	HEXB	Hexb	LOC101400305	RASSF2	Rassf2	LOC101403331
ANTXR1	Antxr1	LOC101403247	HEXD	Hexdc	LOC101403679	RASSF3	Rassf3	LOC101407777
ANTXR2	Antxr2	LOC101390241	HEXIM1	Hexim1	LOC101400263	RASSF4	Rassf4	LOC101398116
ANXA1	Anxa1	LOC101387754	HEXIM2	Hexim2	LOC101407622	RASSF5	Rassf5	LOC101394545
ANXA10	Anxa10	LOC101407526	HEY1	Hey1	LOC101407410	RASSF6	Rassf6	LOC101392837
ANXA11	Anxa11	LOC101396712	HEY2	Hey2	LOC101393434	RASSF7	Rassf7	LOC101394776
ANXA13	Anxa13	LOC101397584	HEYL	Heyl	LOC101404013	RASSF8	Rassf8	LOC101391339
ANXA2	Anxa2	LOC101399619	HFE	Hfe	LOC101407879	RASSF9	Rassf9	LOC101399938
ANXA3	Anxa3	LOC101388941	HFM1	Hfm1	LOC101389667	RAVER1	Raver1	LOC101388041
ANXA4	Anxa4	LOC101401767	HGD	Hgd	LOC101401610	RAVER2	Raver2	LOC101393983
ANXA5	Anxa5	LOC101388235	HGF	Hgf	LOC101387216	RAX	Rax	LOC101406698
ANXA6	Anxa6	LOC101397060	HGFAC	Hgfac	LOC101399915	RB1	Rb1	LOC101390253
ANXA7	Anxa7	LOC101392164	HGH1	Hgh1	LOC101403554	RB1CC1	Rb1cc1	LOC101387659
ANXA8L1	Anxa8	LOC101388500	HGS	Hgs	LOC101396551	RBBP4	Rbbp4	LOC101402692
ANXA9	Anxa9	LOC101396036	HGSNAT	Hgsnat	LOC101400836	RBBP5	Rbbp5	LOC101400158
AOAH	Aoah	LOC101398460	HHAT	Hhat	LOC101406379	RBBP6	Rbbp6	LOC101406767
AOC1	Aoc1	LOC101389210	HHATL	Hhatl	LOC101394015	RBBP7	Rbbp7	LOC101398880
AOC2	Aoc2	LOC101390162	HHEX	Hhex	LOC101396103	RBBP8	Rbbp8	LOC101388720
AOPEP	2010111I01R	LOC101393259	HHIP	Hhip	LOC101394519	RBBP8NL	Rbbp8nl	LOC101404640
AOX1	Aox1	LOC106800147	HHIPL1	Hhipl1	LOC101387653	RBBP9	Rbbp9	LOC101387476
AP1AR	Ap1ar	LOC101397572	HHIPL2	Hhipl2	LOC101403970	RBCK1	Rbck1	LOC101398440
AP1B1	Ap1b1	LOC101390916	HHLA1	Hhla1	LOC101391078	RBFA	Rbfa	LOC101391390
AP1G1	Ap1g1	LOC101387219	HIBADH	Hibadh	LOC101391444	RBFOX1	Rbfox1	LOC101389147
AP1G2	Ap1g2	LOC101401454	HIBCH	Hibch	LOC101398423	RBFOX2	Rbfox2	LOC101394093
AP1M1	Ap1m1	LOC101400587	HIC1	Hic1	LOC101400004	RBFOX3	Rbfox3	LOC101390757
AP1M2	Ap1m2	LOC101390202	HID1	Hid1	LOC101390153	RBIS	1810022K09F	LOC101395548
AP1S1	Ap1s1	LOC101389325	HIF1A	Hif1a	LOC101396970	RBKS	Rbks	LOC101397956
AP1S2	Ap1s2	LOC101396997	HIF1AN	Hif1an	LOC101401832	RBL1	Rbl1	LOC101404973
AP1S3	Ap1s3	LOC101403830	HIF3A	Hif3a	LOC101402650	RBL2	Rbl2	LOC101404980
AP2A1	Ap2a1	LOC101408941	HIGD1A	Higd1a	LOC101394511	RBM10	Rbm10	LOC101393824
AP2A2	Ap2a2	LOC101408344	HIGD1B	Higd1b	LOC101397074	RBM11	Rbm11	LOC101398065
AP2B1	Ap2b1	LOC101404039	HIGD2A	Higd2a	LOC101408297	RBM14	Rbm14	LOC106799922
AP2M1	Ap2m1	LOC101393201	HILPDA	Hilpda	LOC101399431	RBM15	Rbm15	LOC101396070
AP2S1	Ap2s1	LOC101404599	HINFP	Hinfp	LOC101389354	RBM15B	Rbm15b	LOC101394350

AP3B1	Ap3b1	LOC101408950	HINT1	Hint1	LOC101407120	RBM17	Rbm17	LOC101402002
AP3B2	Ap3b2	LOC101390576	HINT2	Hint2	LOC101394707	RBM18	Rbm18	LOC101408278
AP3D1	Ap3d1	LOC101406604	HINT3	Hint3	LOC101403375	RBM19	Rbm19	LOC101390492
AP3M1	Ap3m1	LOC101408027	HIP1	Hip1	LOC101403630	RBM20	Rbm20	LOC101406637
AP3M2	Ap3m2	LOC101391622	HIP1R	Hip1r	LOC101402527	RBM22	Rbm22	LOC101398510
AP3S1	Ap3s1	LOC101397461	HIPK1	Hipk1	LOC101405568	RBM24	Rbm24	LOC101401594
AP3S2	Ap3s2	LOC106799937	HIPK2	Hipk2	LOC101388161	RBM25	Rbm25	LOC101398098
AP4B1	Ap4b1	LOC101405827	HIPK3	Hipk3	LOC101402726	RBM26	Rbm26	LOC101391471
AP4E1	Ap4e1	LOC101406692	HIPK4	Hipk4	LOC101394694	RBM27	Rbm27	LOC101399697
AP4M1	Ap4m1	LOC101390460	HIRA	Hira	LOC101395555	RBM28	Rbm28	LOC101397709
AP4S1	Ap4s1	LOC101406094	HIRIP3	Hirip3	LOC101391111	RBM3	Rbm3	LOC101390589
AP5B1	Ap5b1	LOC101408847	HIVEP1	Hivep1	LOC101406462	RBM33	Rbm33	LOC101405871
AP5M1	Ap5m1	LOC101389784	HIVEP2	Hivep2	LOC101393597	RBM34	Rbm34	LOC101391664
AP5S1	Ap5s1	LOC101405781	HIVEP3	Hivep3	LOC101402575	RBM38	Rbm38	LOC101406033
AP5Z1	Ap5z1	LOC101393936	HJURP	Hjurp	LOC101406197	RBM39	Rbm39	LOC101389612
APAF1	Apaf1	LOC101394006	HJV	Hfe2	LOC101407385	RBM4	Rbm4	LOC101399413
APBA1	Apba1	LOC101390365	HK1	Hk1	LOC101406635	RBM41	Rbm41	LOC101388449
APBA2	Apba2	LOC101387989	HK2	Hk2	LOC101403784	RBM42	Rbm42	LOC101398902
APBA3	Apba3	LOC101400658	HK3	Hk3	LOC101405072	RBM44	Rbm44	LOC101404887
APBB1	Apbb1	LOC101396577	HKDC1	Hkdc1	LOC101406888	RBM45	Rbm45	LOC101404961
APBB1IP	Apbb1ip	LOC101400336	HLA-A	2410137M14	LOC101398669	RBM46	Rbm46	LOC101387266
APBB2	Apbb2	LOC101397961	HLA-C	H2-Q10	LOC101402588	RBM47	Rbm47	LOC101397112
APBB3	Apbb3	LOC101393261	HLA-DMA	H2-DMa	LOC101402777	RBM48	Rbm48	LOC101398870
APC	Apc	LOC101402660	HLA-DOA	H2-Oa	LOC101401908	RBM4B	Rbm4b	LOC101401083
APC2	Apc2	LOC101409031	HLA-DOB	H2-Ob	LOC101404085	RBM5	Rbm5	LOC101409029
APCDD1	Apcdd1	LOC101396774	HLA-DQA1	H2-Aa	LOC101406278	RBM6	Rbm6	LOC101408777
APCS	Apcs	LOC101407391	HLA-DQB1	H2-Ab1	LOC101405854	RBM7	Rbm7	LOC101409150
APEH	Apeh	LOC101404244	HLA-DRA	H2-Ea-ps	LOC101406780	RBM8A	Rbm8a	LOC101408441
APELA	Apela	LOC106802309	HLA-DRB5	H2-Eb2	LOC101406527	RBMS1	Rbms1	LOC101394059
APEX1	Apex1	LOC101406255	HLCS	Hlcs	LOC101389978	RBMS2	Rbms2	LOC101406204
APEX2	Apex2	LOC101400678	HLF	Hlf	LOC101399668	RBMS3	Rbms3	LOC101388113
APH1A	Aph1a	LOC101389818	HLTF	Hltf	LOC101391368	RBMX	Rbmx	LOC101403273
APH1B	Aph1c	LOC101404417	HLX	Hlx	LOC101398315	RBMX2	Rbmx2	LOC101388011
API5	Api5	LOC101389141	HM13	H13	LOC101393107	RBP1	Rbp1	LOC101392046
APIP	Apip	LOC101406920	HMBOX1	Hmbox1	LOC101397182	RBP2	Rbp2	LOC101392319
APLF	Aplf	LOC101405266	HMBS	Hmbs	LOC101390738	RBP3	Rbp3	LOC101405951
APLN	Apln	LOC101392299	HMCES	Hmces	LOC101394864	RBP4	Rbp4	LOC101398945
APLNR	Aplnr	LOC101387162	HMCN1	Hmcn1	LOC101396524	RBP7	Rbp7	LOC101403913
APLP1	Aplp1	LOC101398638	HMCN2	Hmcn2	LOC101387117	RBPJ	Rbpj	LOC101387180
APLP2	Aplp2	LOC101406836	HMG20A	Hmg20a	LOC101401285	RBPJL	Rbpjl	LOC101388493
APMAP	Apmap	LOC101395469	HMG20B	Hmg20b	LOC101399089	RBPMS	Rbpms	LOC101402874
APOA1	Apoa1	LOC101405070	HMGA1	Hmga1	LOC101393118	RBPMS2	Rbpms2	LOC101387518
APOA2	Apoa2	LOC101397990	HMGA2	Hmga2	LOC101408986	RBSN	Rbsn	LOC101395556
APOA4	Apoa4	LOC101405601	HMGB1	Hmgb1	LOC101391431	RBX1	Rbx1	LOC101402902
APOA5	Apoa5	LOC101405863	HMGB2	Hmgb2	LOC101389809	RC3H1	Rc3h1	LOC101398092
APOB	Apob	LOC101399516	HMGB3	Hmgb3	LOC101395736	RC3H2	Rc3h2	LOC101396881
APOBEC1	Apobec1	LOC101389643	HMGCL	Hmgcl	LOC101405493	RCAN1	Rcan1	LOC101387381
APOBEC2	Apobec2	LOC101396433	HMGCLL1	Hmgcll1	LOC101401305	RCAN2	Rcan2	LOC101391905
APOBEC4	Apobec4	LOC101399979	HMGCR	Hmgcr	LOC101391127	RCAN3	Rcan3	LOC101408468
APOBR	Apobr	LOC101398141	HMGCS1	Hmgcs1	LOC101393020	RCBTB1	Rcbtb1	LOC101392892
APOC3	Apoc3	LOC101405331	HMGCS2	Hmgcs2	LOC101393425	RCBTB2	Rcbtb2	LOC101390761
APOC4	Apoc4	LOC101393414	HMGN2	Hmgn2	LOC101395838	RCC1	Rcc1	LOC101408469
APOD	Apod	LOC101392154	HMGN3	Hmgn3	LOC101403553	RCC2	Rcc2	LOC101388565
APOE	Apoe	LOC101406349	HMGN5	Hmgn5	LOC101393103	RCCD1	Rccd1	LOC101407878

APOF	Apof	LOC101404893	HMGXB3	Hmgxb3	LOC101401741	RCE1	Rce1	LOC101401614
APOH	Apoh	LOC101399487	HMGXB4	Hmgxb4	LOC101392033	RCHY1	Rchy1	LOC101403317
APOLD1	Apold1	LOC101394490	HMMR	Hmmr	LOC101395147	RCL1	Rcl1	LOC101398565
APOM	Apom	LOC101398852	HMOX1	Hmox1	LOC101392907	RCN1	Rcn1	LOC101402827
APOO	Apoo	LOC101408488	HMOX2	Hmox2	LOC101394419	RCN2	Rcn2	LOC101402415
APOOL	Apool	LOC101400276	HMX2	Hmx2	LOC101404393	RCN3	Rcn3	LOC101406241
APP	App	LOC101392359	HMX3	Hmx3	LOC101404663	RCOR2	Rcor2	LOC101409092
APPBP2	Appbp2	LOC101395004	HNF1A	Hnf1a	LOC101400353	RCOR3	Rcor3	LOC101408017
APPL1	Appl1	LOC101392483	HNF1B	Hnf1b	LOC101400723	RCSD1	Rcsd1	LOC101399284
APPL2	Appl2	LOC101408162	HNF4A	Hnf4a	LOC101392689	RCVRN	Rcvrn	LOC101408485
APRT	Aprt	LOC101408670	HNF4G	Hnf4g	LOC101405068	RD3	Rd3	LOC101387124
APTX	Aptx	LOC101393714	HNMT	Hnmt	LOC101406624	RD3L	Rd3l	LOC101400981
AQP1	Aqp1	LOC101407201	HNRNPA0	Hnrnpa0	LOC101387765	RDH10	Rdh10	LOC101399836
AQP11	Aqp11	LOC101408052	HNRNPA1	Hnrnpa1	LOC101395847	RDH11	Rdh11	LOC101408801
AQP2	Aqp2	LOC101406893	HNRNPA2B1	Hnrnpa2b1	LOC101395801	RDH12	Rdh12	LOC101387289
AQP3	Aqp3	LOC101391576	HNRNPA3	Hnrnpa3	LOC101402518	RDH13	Rdh13	LOC101389403
AQP4	Aqp4	LOC101395295	HNRNPAB	Hnrnpab	LOC101396894	RDH14	Rdh14	LOC106799923
AQP5	Aqp5	LOC101407155	HNRNPC	Hnrnpc	LOC101398076	RDH5	Rdh5	LOC101392794
AQP6	Aqp6	LOC101407412	HNRNPD	Hnrnpd	LOC101392278	RDH8	Rdh8	LOC101406510
AQP7	Aqp7	LOC101391834	HNRNPDL	Hnrnpdl	LOC101393057	RDX	Rdx	LOC101399292
AQP8	Aqp8	LOC101401700	HNRNPF	Hnrnpf	LOC106799932	REC114	Rec114	LOC101387519
AQP9	Aqp9	LOC101395385	HNRNPH1	Hnrnph1	LOC101394060	REC8	Rec8	LOC101399360
AQR	Aqr	LOC101406508	HNRNPH2	Hnrnph2	LOC106799945	RECK	Reck	LOC101395737
AR	Ar	LOC101392211	HNRNPH3	Hnrnph3	LOC101389875	RECQL	Recql	LOC101406913
ARAF	Araf	LOC101398539	HNRNPK	Hnrnpk	LOC101388205	RECQL4	Recql4	LOC101408710
ARAP1	Arap1	LOC101391084	HNRNPL	Hnrnpl	LOC101405825	RECQL5	Recql5	LOC101395946
ARAP2	Arap2	LOC101389058	HNRNPLL	Hnrnpll	LOC101403441	REEP1	Reep1	LOC101392642
ARAP3	Arap3	LOC101406356	HNRNPM	Hnrnpm	LOC101402159	REEP2	Reep2	LOC101403801
ARC	Arc	LOC101406800	HNRNPR	Hnrnpr	LOC101400689	REEP3	Reep3	LOC101394549
ARCN1	Arcn1	LOC101397160	HNRNPU	Hnrnpu	LOC101402742	REEP4	Reep4	LOC101407306
AREG	Areg	LOC101396142	HNRNPUL1	Hnrnpul1	LOC101400660	REEP5	Reep5	LOC101401796
AREL1	Arel1	LOC101406533	HNRNPUL2	Hnrnpul2	LOC101400899	REG3A	Reg3b	LOC101406478
ARF1	Arf1	LOC101402236	HOGA1	Hoga1	LOC101392169	REG3G	Reg3g	LOC101405965
ARF3	Arf3	LOC101398951	HOMER1	Homer1	LOC101406425	REL	Rel	LOC101396915
ARF4	Arf4	LOC101393260	HOMER2	Homer2	LOC101391250	RELA	Rela	LOC101406064
ARF5	Arf5	LOC101399335	HOMER3	Homer3	LOC101405739	RELB	Relb	LOC101406596
ARF6	Arf6	LOC101399673	HOMEZ	Homez	LOC101405381	RELL1	Rell1	LOC101390099
ARFGAP1	Arfgap1	LOC101395851	HOOK1	Hook1	LOC101401087	RELL2	Rell2	LOC101406854
ARFGAP2	Arfgap2	LOC101400191	HOOK2	Hook2	LOC101403992	RELN	Reln	LOC101388525
ARFGAP3	Arfgap3	LOC101395360	HOOK3	Hook3	LOC101396064	RELT	Relt	LOC101393733
ARFGEF1	Arfgef1	LOC101394083	HOPX	Hopx	LOC101395023	REM1	Rem1	LOC101393515
ARFGEF2	Arfgef2	LOC101396898	HORMAD1	Hormad1	LOC101394242	REM2	Rem2	LOC101388298
ARFGEF3	Arfgef3	LOC101388906	HORMAD2	Hormad2	LOC101399390	REN	Ren1	LOC101403299
ARFIP1	Arfip1	LOC101404777	HOXA1	Hoxa1	LOC101394599	RENBP	Renbp	LOC101390387
ARFIP2	Arfip2	LOC101399425	HOXA10	Hoxa10	LOC101392475	REP15	Rep15	LOC101394666
ARFRP1	Arfrp1	LOC101392361	HOXA11	Hoxa11	LOC101392212	REPIN1	Repin1	LOC101390917
ARG1	Arg1	LOC101401645	HOXA13	Hoxa13	LOC101407555	REPS1	Reps1	LOC101390124
ARG2	Arg2	LOC101408286	HOXA2	Hoxa2	LOC101394343	REPS2	Reps2	LOC101399500
ARGLU1	Arglu1	LOC101407397	HOXA3	Hoxa3	LOC101393752	RER1	Rer1	LOC101395836
ARHGAP1	Arhgap1	LOC101398971	HOXA4	Hoxa4	LOC101407817	RERE	Rere	LOC101405491
ARHGAP10	Arhgap10	LOC101400133	HOXA5	Hoxa5	LOC101393492	RERG	Rerg	LOC101399833
ARHGAP11A	Arhgap11a	LOC101405383	HOXA6	Hoxa6	LOC101393250	RERGL	Rergl	LOC101402814
ARHGAP12	Arhgap12	LOC101392781	HOXA7	Hoxa7	LOC101392992	RESP18	Resp18	LOC101396183
ARHGAP15	Arhgap15	LOC101405147	HOXA9	Hoxa9	LOC101392728	REST	Rest	LOC101395631

ARHGAP17	Arhgap17	LOC101392595	HOXB1	Hoxb1	LOC101395434	RET	Ret	LOC101401849
ARHGAP18	Arhgap18	LOC101398921	HOXB13	Hoxb13	LOC101397940	RETN	Retn	LOC101391981
ARHGAP19	Arhgap19	LOC106799940	HOXB2	Hoxb2	LOC101395697	RETSAT	Retsat	LOC101387161
ARHGAP20	Arhgap20	LOC101398677	HOXB3	Hoxb3	LOC101395955	REV1	Rev1	LOC101406660
ARHGAP21	Arhgap21	LOC101402341	HOXB4	Hoxb4	LOC101396383	REV3L	Rev3l	LOC101399622
ARHGAP22	Arhgap22	LOC101408562	HOXB5	Hoxb5	LOC101396649	REX1BD	2810428I15R	LOC101409042
ARHGAP23	Arhgap23	LOC101390424	HOXB6	Hoxb6	LOC101396913	REXO1	Rexo1	LOC101408865
ARHGAP24	Arhgap24	LOC101399130	HOXB7	Hoxb7	LOC101397185	REXO2	Rexo2	LOC101408893
ARHGAP25	Arhgap25	LOC101404576	HOXB8	Hoxb8	LOC101397433	REXO4	Rexo4	LOC101408197
ARHGAP26	Arhgap26	LOC101403455	HOXB9	Hoxb9	LOC101397688	REXO5	2610020H08I	LOC101394527
ARHGAP27	Arhgap27	LOC101408659	HOXC10	Hoxc10	LOC101393551	RFC1	Rfc1	LOC101393252
ARHGAP28	Arhgap28	LOC101400043	HOXC11	Hoxc11	LOC101393302	RFC2	Rfc2	LOC101399352
ARHGAP29	Arhgap29	LOC101402665	HOXC12	Hoxc12	LOC101393049	RFC4	Rfc4	LOC101403130
ARHGAP30	Arhgap30	LOC101391903	HOXC13	Hoxc13	LOC101392793	RFC5	Rfc5	LOC101409161
ARHGAP31	Arhgap31	LOC101406576	HOXC4	Hoxc4	LOC106799953	RFESD	Rfesd	LOC101392137
ARHGAP32	Arhgap32	LOC101404843	HOXC5	Hoxc5	LOC101394388	RFFL	Rffl	LOC101402493
ARHGAP33	Arhgap33	LOC101389488	HOXC6	Hoxc6	LOC101393806	RFK	Rfk	LOC101391198
ARHGAP35	Arhgap35	LOC101405020	HOXC8	Hoxc8	LOC106800011	RFNG	Rfng	LOC101404902
ARHGAP36	Arhgap36	LOC101408928	HOXC9	Hoxc9	LOC101391494	RFT1	Rft1	LOC101407559
ARHGAP39	Arhgap39	LOC101407582	HOXD1	Hoxd1	LOC101402007	RFTN1	Rftn1	LOC101395114
ARHGAP4	Arhgap4	LOC101389510	HOXD10	Hoxd10	LOC101400691	RFTN2	Rftn2	LOC101405860
ARHGAP40	Arhgap40	LOC101400876	HOXD11	Hoxd11	LOC101405667	RFWD3	Rfwd3	LOC101408580
ARHGAP42	Arhgap42	LOC101391317	HOXD12	Hoxd12	LOC101399808	RFX1	Rfx1	LOC101398478
ARHGAP44	Arhgap44	LOC101404829	HOXD13	Hoxd13	LOC101399555	RFX2	Rfx2	LOC101407019
ARHGAP5	Arhgap5	LOC101403807	HOXD3	Hoxd3	LOC101401736	RFX3	Rfx3	LOC101396406
ARHGAP6	Arhgap6	LOC101407706	HOXD8	Hoxd8	LOC101400951	RFX4	Rfx4	LOC101387580
ARHGAP8	Arhgap8	LOC101399237	HOXD9	Hoxd9	LOC101400432	RFX5	Rfx5	LOC101402627
ARHGAP9	Arhgap9	LOC101393304	HP	Hp	LOC101404297	RFX6	Rfx6	LOC101407745
ARHGDIA	Arhgdia	LOC101398531	HP1BP3	Hp1bp3	LOC101393292	RFX7	Rfx7	LOC101392235
ARHGDIB	Arhgdib	LOC101399320	HPCA	Hpca	LOC101404281	RFX8	Rfx8	LOC101387398
ARHGDIG	Arhgdig	LOC101401261	HPCAL4	Hpcal4	LOC101404547	RFXANK	Rfxank	LOC101403558
ARHGEF1	Arhgef1	LOC101406847	HPD	Hpd	LOC101406544	RFXAP	Rfxap	LOC101409005
ARHGEF10	Arhgef10	LOC101404672	HPDL	Hpdl	LOC101394520	RGCC	Rgcc	LOC101402889
ARHGEF10L	Arhgef10l	LOC101406283	HPF1	Hpf1	LOC101388085	RGL1	Rgl1	LOC101399195
ARHGEF11	Arhgef11	LOC101391094	HPGD	Hpgd	LOC101391253	RGL2	Rgl2	LOC101398177
ARHGEF12	Arhgef12	LOC101406788	HPGDS	Hpgds	LOC101388676	RGL3	Rgl3	LOC101395212
ARHGEF15	Arhgef15	LOC101391847	HPN	Hpn	LOC101391021	RGMA	Rgma	LOC101400088
ARHGEF16	Arhgef16	LOC101397567	HPS1	Hps1	LOC101396104	RGMB	Rgmb	LOC101396411
ARHGEF17	Arhgef17	LOC101393470	HPS3	Hps3	LOC101390445	RGN	Rgn	LOC101396122
ARHGEF18	Arhgef18	LOC101389681	HPS4	Hps4	LOC101389207	RGP1	Rgp1	LOC101389434
ARHGEF19	Arhgef19	LOC101402514	HPS5	Hps5	LOC101387497	RGR	Rgr	LOC101394069
ARHGEF2	Arhgef2	LOC101399757	HPS6	Hps6	LOC101387979	RGS1	Rgs1	LOC101388965
ARHGEF25	Arhgef25	LOC101398014	HPSE	Hpse	LOC101396286	RGS10	Rgs10	LOC101397664
ARHGEF26	Arhgef26	LOC101408182	HPSE2	Hpse2	LOC101396369	RGS11	Rgs11	LOC101401524
ARHGEF28	Arhgef28	LOC101392843	HPX	Hpx	LOC101398554	RGS12	Rgs12	LOC101401416
ARHGEF3	Arhgef3	LOC101391028	HR	Hr	LOC101407575	RGS13	Rgs13	LOC101388699
ARHGEF33	Arhgef33	LOC101407636	HRAS	Hras	LOC101395198	RGS14	Rgs14	LOC101402180
ARHGEF37	Arhgef37	LOC101398768	HRC	Hrc	LOC101401004	RGS16	Rgs16	LOC101404442
ARHGEF39	Arhgef39	LOC101387355	HRG	Hrg	LOC101404177	RGS17	Rgs17	LOC101406422
ARHGEF4	Arhgef4	LOC101404700	HRH3	Hrh3	LOC101399737	RGS18	Rgs18	LOC101389233
ARHGEF40	Arhgef40	LOC101400311	HRH4	Hrh4	LOC101398326	RGS19	Rgs19	LOC101388938
ARHGEF5	Arhgef5	LOC101395335	HS1BP3	Hs1bp3	LOC101399161	RGS2	Rgs2	LOC101388194
ARHGEF6	Arhgef6	LOC101403015	HS2ST1	Hs2st1	LOC101405998	RGS20	Rgs20	LOC101389040
ARHGEF7	Arhgef7	LOC101388222	HS3ST1	Hs3st1	LOC101408916	RGS21	Rgs21	LOC101395102

ARHGEF9	Arhgef9	LOC101396404	HS3ST2	Hs3st2	LOC101389329	RGS22	Rgs22	LOC101405251
ARID1A	Arid1a	LOC101396526	HS3ST3A1	Hs3st3a1	LOC101404568	RGS3	Rgs3	LOC101397485
ARID1B	Arid1b	LOC101391450	HS3ST3B1	Hs3st3b1	LOC101404033	RGS4	Rgs4	LOC101398091
ARID2	Arid2	LOC101387810	HS3ST4	Hs3st4	LOC101396052	RGS5	Rgs5	LOC101398498
ARID3A	Arid3a	LOC101391207	HS3ST6	Hs3st6	LOC106803074	RGS6	Rgs6	LOC101395755
ARID3B	Arid3b	LOC101392754	HS6ST1	Hs6st1	LOC101403387	RGS7	Rgs7	LOC101391525
ARID3C	Arid3c	LOC101407333	HS6ST2	Hs6st2	LOC101406063	RGS7BP	Rgs7bp	LOC101391051
ARID4A	Arid4a	LOC101391483	HS6ST3	Hs6st3	LOC101398664	RGS8	Rgs8	LOC101403824
ARID4B	Arid4b	LOC101391929	HSBP1	Hsbp1	LOC101396306	RGS9	Rgs9	LOC101400621
ARID5A	Arid5a	LOC101402297	HSBP1L1	Hsbp1l1	LOC101391144	RGS9BP	Rgs9bp	LOC101406844
ARID5B	Arid5b	LOC101397245	HSCB	Hscb	LOC101408816	RGSL1	Rgsl1	LOC101404705
ARIH1	Arih1	LOC101408184	HSD11B1	Hsd11b1	LOC101403823	RHAG	Rhag	LOC101396703
ARIH2	Arih2	LOC101395459	HSD11B2	Hsd11b2	LOC101390919	RHBDD1	Rhbdd1	LOC101400696
ARL1	Arl1	LOC101400114	HSD17B1	Hsd17b1	LOC101396560	RHBDD2	Rhbdd2	LOC101404337
ARL10	Arl10	LOC101408810	HSD17B10	Hsd17b10	LOC101396782	RHBDD3	Rhbdd3	LOC101406898
ARL11	Arl11	LOC101393149	HSD17B11	Hsd17b11	LOC101402707	RHBDF1	Rhbdf1	LOC101408425
ARL13A	Arl13a	LOC101407208	HSD17B12	Hsd17b12	LOC101390173	RHBDF2	Rhbdf2	LOC101405425
ARL13B	Arl13b	LOC101401093	HSD17B13	Hsd17b13	LOC101402281	RHBDL1	Rhbdl1	LOC101402301
ARL14	Arl14	LOC101397377	HSD17B14	Hsd17b14	LOC101400744	RHBDL2	Rhbdl2	LOC101401315
ARL14EP	Arl14ep	LOC101400739	HSD17B2	Hsd17b2	LOC101397440	RHBDL3	Rhbdl3	LOC101409034
ARL14EPL	Arl14epl	LOC101396943	HSD17B3	Hsd17b3	LOC101402836	RHBG	Rhbg	LOC101406476
ARL15	Arl15	LOC101397737	HSD17B4	Hsd17b4	LOC101394516	RHCE	Rhd	LOC101389097
ARL16	Arl16	LOC101403327	HSD17B6	Hsd17b6	LOC101408985	RHCG	Rhcg	LOC101402191
ARL2	Arl2	LOC101399069	HSD17B7	Hsd17b7	LOC101387199	RHEB	Rheb	LOC101407696
ARL2BP	Arl2bp	LOC106802023	HSD17B8	H2-Ke6	LOC101400332	RHEBL1	Rhebl1	LOC101400872
ARL3	Arl3	LOC101393210	HSD3B2	Hsd3b1	LOC101394356	RHNO1	Rhno1	LOC101388778
ARL4A	Arl4a	LOC101390574	HSD3B7	Hsd3b7	LOC101403350	RHO	Rho	LOC101394785
ARL4C	Arl4c	LOC101400782	HSDL1	Hsdl1	LOC101395010	RHOA	Rhoa	LOC101401790
ARL4D	Arl4d	LOC101408563	HSDL2	Hsdl2	LOC101392862	RHOB	Rhob	LOC101399424
ARL5A	Arl5a	LOC101399632	HSF1	Hsf1	LOC101390548	RHOBTB1	Rhobtb1	LOC101398183
ARL5B	Arl5b	LOC101406445	HSF2	Hsf2	LOC101390205	RHOBTB2	Rhobtb2	LOC101401718
ARL5C	Arl5c	LOC101407268	HSF2BP	Hsf2bp	LOC101401483	RHOBTB3	Rhobtb3	LOC101391623
ARL6	Arl6	LOC101402307	HSF4	Hsf4	LOC101408651	RHOC	Rhoc	LOC101387597
ARL6IP1	Arl6ip1	LOC101393428	HSF5	Hsf5	LOC101393995	RHOD	Rhod	LOC101402992
ARL6IP4	Arl6ip4	LOC101390748	HSH2D	Hsh2d	LOC101401901	RHOF	Rhof	LOC101405678
ARL6IP5	Arl6ip5	LOC101406752	HSP90AA1	Hsp90aa1	LOC101392015	RHOG	Rhog	LOC101407968
ARL6IP6	Arl6ip6	LOC101397910	HSP90AB1	Hsp90ab1	LOC101395658	RHOJ	Rhoj	LOC101398674
ARL8A	Arl8a	LOC101398384	HSP90B1	Hsp90b1	LOC101404589	RHOQ	Rhoq	LOC101397525
ARL8B	Arl8b	LOC101403726	HSPA12A	Hspa12a	LOC101392351	RHOT1	Rhot1	LOC101387344
ARL9	Arl9	LOC101394766	HSPA12B	Hspa12b	LOC101407624	RHOT2	Rhot2	LOC101402551
ARMC1	Armc1	LOC101397602	HSPA13	Hspa13	LOC101394433	RHOU	Rhou	LOC101393372
ARMC10	Armc10	LOC101398811	HSPA14	Hspa14	LOC101392675	RHOV	Rhov	LOC101394611
ARMC12	Armc12	LOC101407940	HSPA1A	Hspa1a	LOC101392936	RHPN1	Rhpn1	LOC101406953
ARMC2	Armc2	LOC101392415	HSPA1B	Hspa1b	LOC101392671	RHPN2	Rhpn2	LOC101406075
ARMC3	Armc3	LOC101403481	HSPA1L	Hspa1l	LOC101393199	RIBC1	Ribc1	LOC101396351
ARMC4	Armc4	LOC101395749	HSPA2	Hspa2	LOC106801192	RIBC2	Ribc2	LOC101401259
ARMC5	Armc5	LOC101407726	HSPA4	Hspa4	LOC101397809	RIC1	Ric1	LOC101394413
ARMC6	Armc6	LOC101405052	HSPA4L	Hspa4l	LOC101404806	RIC3	Ric3	LOC101388701
ARMC7	Armc7	LOC101391586	HSPA5	Hspa5	LOC101402682	RIC8A	Ric8a	LOC101387339
ARMC8	Armc8	LOC101404857	HSPA8	Hspa8	LOC101403829	RIC8B	Ric8b	LOC101388196
ARMC9	Armc9	LOC101403050	HSPA9	Hspa9	LOC101402838	RICTOR	Rictor	LOC101409133
ARMCX3	Armcx3	LOC101398228	HSPB1	Hspb1	LOC101405645	RIDA	Rida	LOC101405250
ARMCX6	Armcx6	LOC101398825	HSPB11	Hspb11	LOC101392201	RIF1	Rif1	LOC101400152
ARMT1	Armt1	LOC101403879	HSPB2	Hspb2	LOC101394984	RIIAD1	Riiad1	LOC101406329

ARNT	Arnt	LOC101405351	HSPB3	Hspb3	LOC101397988	RILP	Rilp	LOC101396994
ARNT2	Arnt2	LOC101396205	HSPB6	Hspb6	LOC101395715	RILPL1	Rilpl1	LOC101393717
ARNTL	Arntl	LOC101399077	HSPB7	Hspb7	LOC101401577	RILPL2	Rilpl2	LOC101393054
ARNTL2	Arntl2	LOC101406659	HSPB8	Hspb8	LOC101407417	RIMBP2	Rimbp2	LOC101398775
ARPC1A	Arpc1a	LOC101406353	HSPB9	Hspb9	LOC101406057	RIMKLA	Rimkla	LOC101403555
ARPC1B	Arpc1b	LOC101406084	HSPBAP1	Hspbap1	LOC101399893	RIMKLB	Rimklb	LOC101388878
ARPC2	Arpc2	LOC101406536	HSPBP1	Hspbp1	LOC101391017	RIMS1	Rims1	LOC101393615
ARPC3	Arpc3	LOC101400612	HSPD1	Hspd1	LOC101404710	RIMS2	Rims2	LOC101392955
ARPC4	Arpc4	LOC101391554	HSPG2	Hspg2	LOC101396628	RIMS3	Rims3	LOC101389350
ARPC5	Arpc5	LOC101400239	HSPH1	Hsph1	LOC101392810	RIMS4	Rims4	LOC101390749
ARPC5L	Arpc5l	LOC101400682	HTATIP2	Htatip2	LOC101394428	RIN1	Rin1	LOC101394583
ARPIN	Arpin	LOC101403938	HTATSF1	Htatsf1	LOC101402239	RIN2	Rin2	LOC101400366
ARPP19	Arpp19	LOC101388634	HTR1A	Htr1a	LOC101390551	RIN3	Rin3	LOC101400980
ARPP21	Arpp21	LOC101395544	HTR1B	Htr1b	LOC101403025	RING1	Ring1	LOC101400061
ARR3	Arr3	LOC101387683	HTR2A	Htr2a	LOC101389030	RINL	Rinl	LOC101406358
ARRB1	Arrb1	LOC101401505	HTR2B	Htr2b	LOC101391649	RINT1	Rint1	LOC101408243
ARRB2	Arrb2	LOC101393316	HTR2C	Htr2c	LOC101401098	RIOK1	Riok1	LOC101389805
ARRDC1	Arrdc1	LOC101395574	HTR3A	Htr3a	LOC101388240	RIOK2	Riok2	LOC101388629
ARRDC2	Arrdc2	LOC101391468	HTR3B	Htr3b	LOC101388486	RIOK3	Riok3	LOC101396776
ARRDC3	Arrdc3	LOC101394778	HTR4	Htr4	LOC101405866	RIOX2	Mina	LOC101402559
ARRDC4	Arrdc4	LOC101394480	HTR5A	Htr5a	LOC101402529	RIPK1	Ripk1	LOC101395422
ARRDC5	Arrdc5	LOC101397547	HTR6	Htr6	LOC101389096	RIPK2	Ripk2	LOC101393640
ARSA	Arsa	LOC101388441	HTR7	Htr7	LOC101391733	RIPK3	Ripk3	LOC101399099
ARSB	Arsb	LOC101398470	HTRA2	Htra2	LOC101402039	RIPK4	Ripk4	LOC101396454
ARSG	Arsg	LOC101390154	HTRA3	Htra3	LOC101402712	RIPPLY1	Ripply1	LOC101402919
ARSI	Arsi	LOC101399988	HTRA4	Htra4	LOC101402439	RIPPLY2	Ripply2	LOC101394035
ARSJ	Arsj	LOC101393708	HTT	Htt	LOC101400885	RIPPLY3	Ripply3	LOC101399563
ARSK	Arsk	LOC101392404	HUNK	Hunk	LOC101394644	RIT1	Rit1	LOC101398621
ART1	Art1	LOC101387145	HUS1	Hus1	LOC101394854	RIT2	Rit2	LOC101404688
ART3	Art3	LOC101406393	HUWE1	Huwe1	LOC101397231	RITA1	Rita1	LOC101392174
ART4	Art4	LOC101404042	HVCN1	Hvcn1	LOC101397844	RLBP1	Rlbp1	LOC101401414
ART5	Art5	LOC101387652	HYAL3	Hyal3	LOC101389064	RLF	Rlf	LOC101407148
ARTN	Artn	LOC101405038	HYAL4	Hyal4	LOC101401531	RLIM	Rlim	LOC101396600
ARV1	Arv1	LOC101387387	HYDIN	Hydin	LOC101389467	RLN3	Rln3	LOC101389178
ARVCF	Arvcf	LOC101393271	HYKK	Hykk	LOC101405918	RMC1	3110002H16I	LOC101389250
ARX	Arx	LOC101393825	HYOU1	Hyou1	LOC101391732	RMDN1	Rmdn1	LOC101397968
AS3MT	As3mt	LOC101394817	HYPK	Hypk	LOC101389328	RMDN2	Rmdn2	LOC101405105
ASAH1	Asah1	LOC101399561	IAH1	Iah1	LOC101404741	RMDN3	Rmdn3	LOC101392851
ASAH2	Asah2	LOC101400558	IAPP	Iapp	LOC101406144	RMI1	Rmi1	LOC101389840
ASAP1	Asap1	LOC101392010	IARS2	Iars2	LOC101396581	RMND1	Rmnd1	LOC101403449
ASAP2	Asap2	LOC101394322	IBSP	Ibsp	LOC101403932	RMND5A	Rmnd5a	LOC101394164
ASAP3	Asap3	LOC101402781	IBTK	Ibtk	LOC101406103	RMND5B	Rmnd5b	LOC101397577
ASB1	Asb1	LOC101408550	ICA1	Ica1	LOC101388165	RNASE1	Rnase1	LOC101403987
ASB10	Asb10	LOC101407068	ICA1L	Ica1l	LOC101393371	RNASE10	Rnase10	LOC101405572
ASB11	Asb11	LOC101394160	ICAM1	Icam1	LOC101408875	RNASE11	Rnase11	LOC101405305
ASB13	Asb13	LOC101403911	ICAM2	Icam2	LOC101388852	RNASE12	Rnase12	LOC101405840
ASB14	Asb14	LOC101392737	ICAM4	Icam4	LOC101409126	RNASE13	Rnase13	LOC101400845
ASB15	Asb15	LOC101402306	ICAM5	Icam5	LOC101387270	RNASE4	Rnase4	LOC101404521
ASB16	Asb16	LOC101391419	ICE1	Ice1	LOC101392579	RNASE6	Rnase6	LOC101404255
ASB17	Asb17	LOC101400445	ICE2	Ice2	LOC101399874	RNASEH1	Rnaseh1	RNASEH1
ASB18	Asb18	LOC101406716	ICK	Ick	LOC101404174	RNASEH2A	Rnaseh2a	LOC101404785
ASB2	Asb2	LOC101404732	ICMT	Icmt	LOC101400777	RNASEH2B	Rnaseh2b	LOC101395080
ASB3	Asb3	LOC101389638	ICOS	Icos	LOC101391830	RNASEH2C	Rnaseh2c	LOC101408582
ASB4	Asb4	LOC101405339	ID1	Id1	LOC101392852	RNASEK	Rnasek	LOC101392100

ASB5	Asb5	LOC101400248	ID2	Id2	LOC101402989	RNASEL	Rnasel	LOC101392868
ASB6	Asb6	LOC101405657	ID3	Id3	LOC101403485	RND1	Rnd1	LOC101398188
ASB7	Asb7	LOC101390754	ID4	Id4	LOC101402366	RND2	Rnd2	LOC101387742
ASB8	Asb8	LOC101392792	IDE	Ide	LOC101395145	RND3	Rnd3	LOC101401570
ASB9	Asb9	LOC101393739	IDH1	Idh1	LOC101403827	RNF10	Rnf10	LOC101402703
ASCC1	Ascc1	LOC101397756	IDH2	Idh2	LOC101404824	RNF103	Rnf103	LOC106802961
ASCC2	Ascc2	LOC101400254	IDH3A	Idh3a	LOC101407468	RNF11	Rnf11	LOC101403863
ASCC3	Ascc3	LOC101405730	IDH3B	Idh3b	LOC101392702	RNF111	Rnf111	LOC101396866
ASCL1	Ascl1	LOC101404054	IDH3G	Idh3g	LOC101387190	RNF112	Rnf112	LOC101397426
ASF1A	Asf1a	LOC101388385	IDI1	Idi1	LOC101389969	RNF114	Rnf114	LOC101393976
ASF1B	Asf1b	LOC101389943	IDO1	Ido1	LOC101388028	RNF115	Rnf115	LOC101387768
ASGR1	Asgr1	LOC101390155	IDO2	Ido2	LOC101388454	RNF121	Rnf121	LOC101387911
ASGR2	Asgr2	LOC101390671	IDS	Ids	LOC101399359	RNF122	Rnf122	LOC101408641
ASH1L	Ash1l	LOC101396478	IDUA	Idua	LOC101391585	RNF123	Rnf123	LOC101404764
ASH2L	Ash2l	LOC101390408	IER2	Ier2	LOC101387603	RNF125	Rnf125	LOC101392240
ASIC1	Asic1	LOC101407949	IER3	Ier3	LOC101405323	RNF128	Rnf128	LOC101387086
ASIC2	Asic2	LOC101405991	IER3IP1	Ier3ip1	LOC101389259	RNF13	Rnf13	LOC101388367
ASIC3	Asic3	LOC101387558	IFFO1	Iffo1	LOC101405101	RNF130	Rnf130	LOC101392270
ASIC4	Asic4	LOC101397838	IFFO2	Iffo2	LOC101389098	RNF133	Rnf133	LOC101404595
ASIC5	Asic5	LOC101389249	IFI16	LOC1026395	LOC101400852	RNF135	Rnf135	LOC101388122
ASL	Asl	LOC101391360	IFI30	Ifi30	LOC101390808	RNF138	Rnf138	LOC101392504
ASNS	Asns	LOC101408307	IFI35	Ifi35	LOC101388261	RNF139	Rnf139	LOC101395855
ASNSD1	Asnsd1	LOC101396274	IFI44	Ifi44	LOC101398362	RNF14	Rnf14	LOC101405294
ASPA	Aspa	LOC101391934	IFIH1	Ifih1	LOC101390654	RNF141	Rnf141	LOC101395188
ASPDH	Aspdh	LOC101392315	IFIT1B	Ifit1	LOC101402871	RNF144A	Rnf144a	LOC101402724
ASPG	Aspg	LOC101390762	IFITM3	Ifitm3	LOC101388793	RNF144B	Rnf144b	LOC101398962
ASPH	Asph	LOC101406061	IFITM5	Ifitm5	LOC101388288	RNF145	Rnf145	LOC101406455
ASPHD1	Asphd1	LOC101392832	IFNA1	Ifna1	LOC101389110	RNF148	Rnf148	LOC101404851
ASPHD2	Asphd2	LOC101389459	IFNA21	Ifna15	LOC101402379	RNF149	Rnf149	LOC101409006
ASPM	Aspm	LOC101407544	IFNA7	Ifna9	LOC101397694	RNF150	Rnf150	LOC101395406
ASPN	Aspn	LOC101396688	IFNA8	Ifna5	LOC101389602	RNF151	Rnf151	LOC101396572
ASPRV1	Asprv1	LOC101404479	IFNAR1	Ifnar1	LOC101406205	RNF152	Rnf152	LOC101408274
ASPSCR1	Aspscr1	LOC101404378	IFNAR2	Ifnar2	LOC101396197	RNF157	Rnf157	LOC101396552
ASRGL1	Asrgl1	LOC101397195	IFNB1	Ifnb1	LOC101402630	RNF165	Rnf165	LOC101408102
ASS1	Ass1	LOC101387364	IFNE	Ifne	LOC101401322	RNF166	Rnf166	LOC101409179
ASTE1	Aste1	LOC101395885	IFNG	Ifng	LOC101388839	RNF167	Rnf167	LOC101398031
ASTL	Astl	LOC101397348	IFNGR2	Ifngr2	LOC101396455	RNF168	Rnf168	LOC101408885
ASTN1	Astn1	LOC101393619	IFNL1	Gm6518	LOC101408951	RNF169	Rnf169	LOC101399491
ASTN2	Astn2	LOC101400944	IFNLR1	Ifnlr1	LOC101407493	RNF17	Rnf17	LOC101397090
ASXL1	Asxl1	LOC101388125	IFRD1	Ifrd1	LOC101395019	RNF170	Rnf170	LOC101395636
ASXL2	Asxl2	LOC101392720	IFRD2	Ifrd2	LOC101388790	RNF180	Rnf180	LOC101390807
ASXL3	Asxl3	LOC101393695	IFT122	Ift122	LOC101394194	RNF181	Rnf181	LOC101389395
ASZ1	Asz1	LOC101387177	IFT140	Ift140	LOC101394499	RNF185	Rnf185	LOC101391839
ATAD1	Atad1	LOC101399602	IFT172	Ift172	LOC101401437	RNF186	Rnf186	LOC101389591
ATAD2	Atad2	LOC101399131	IFT20	Ift20	LOC101400264	RNF187	Rnf187	LOC101405034
ATAD2B	Atad2b	LOC101398218	IFT22	Ift22	LOC101387774	RNF19A	Rnf19a	LOC101408394
ATAD3A	Atad3a	LOC101391566	IFT27	Ift27	LOC101396753	RNF19B	Rnf19b	LOC101398349
ATAD5	Atad5	LOC101388802	IFT43	Ift43	LOC101390481	RNF2	Rnf2	LOC101397750
ATAT1	Atat1	LOC101408625	IFT46	Ift46	LOC101396532	RNF20	Rnf20	LOC101397232
ATCAY	Atcay	LOC101397546	IFT52	Ift52	LOC101395247	RNF207	Rnf207	LOC101401042
ATE1	Ate1	LOC101399986	IFT57	Ift57	LOC101396917	RNF208	Rnf208	LOC101391721
ATF1	Atf1	LOC101388930	IFT74	Ift74	LOC101398266	RNF212	Rnf212	LOC101399045
ATF2	Atf2	LOC101398762	IFT80	Ift80	LOC101396334	RNF212B	Rnf212b	LOC106800452
ATF3	Atf3	LOC101389867	IFT81	Ift81	LOC101401586	RNF213	Rnf213	LOC101401236

ATF4	Atf4	LOC101398452	IFT88	Ift88	LOC101400014	RNF214	Rnf214	LOC101403571
ATF5	Atf5	LOC101389242	IGBP1	Igbp1	LOC101388201	RNF215	Rnf215	LOC101397843
ATF6	Atf6	LOC101394976	IGDCC3	Igdcc3	LOC101388551	RNF216	Rnf216	LOC101390949
ATF6B	Atf6b	LOC101388736	IGDCC4	Igdcc4	LOC101388809	RNF217	Rnf217	LOC101403113
ATF7	Atf7	LOC101391492	IGF1	Igf1	LOC101402642	RNF220	Rnf220	LOC101390376
ATF7IP	Atf7ip	LOC101397092	IGF1R	Igf1r	LOC101394229	RNF222	Rnf222	LOC101391588
ATF7IP2	Atf7ip2	LOC101409182	IGF2	Igf2	LOC101398066	RNF223	Rnf223	LOC101393039
ATG10	Atg10	LOC101397970	IGF2BP1	Igf2bp1	LOC101400270	RNF24	Rnf24	LOC101405258
ATG101	Atg101	LOC101398513	IGF2BP2	Igf2bp2	LOC101407757	RNF25	Rnf25	LOC101388571
ATG12	Atg12	LOC101392845	IGF2BP3	Igf2bp3	LOC101400207	RNF26	Rnf26	LOC101388066
ATG13	Atg13	LOC101397866	IGF2R	Igf2r	LOC101393172	RNF31	Rnf31	LOC101396337
ATG14	Atg14	LOC101387541	IGFALS	Igfals	LOC101392462	RNF32	Rnf32	LOC101401489
ATG16L1	Atg16l1	LOC101397662	IGFBP1	Igfbp1	LOC101393920	RNF34	Rnf34	LOC101403927
ATG16L2	Atg16l2	LOC101392104	IGFBP2	Igfbp2	LOC101397323	RNF38	Rnf38	LOC101395482
ATG2A	Atg2a	LOC101398302	IGFBP3	Igfbp3	LOC101394330	RNF39	Rnf39	LOC101400405
ATG2B	Atg2b	LOC101387314	IGFBP4	Igfbp4	LOC101394318	RNF4	Rnf4	LOC101396904
ATG3	Atg3	LOC101392559	IGFBP5	Igfbp5	LOC101404810	RNF40	Rnf40	LOC101400996
ATG4A	Atg4a	LOC101392398	IGFBP6	Igfbp6	LOC101406894	RNF41	Rnf41	LOC101400703
ATG4B	Atg4b	LOC101391163	IGFBP7	Igfbp7	LOC101396759	RNF43	Rnf43	LOC101407630
ATG4C	Atg4c	LOC101405874	IGFBPL1	Igfbpl1	LOC101396265	RNF44	Rnf44	LOC101407154
ATG4D	Atg4d	LOC101403372	IGFLR1	Igflr1	LOC101400556	RNF5	Rnf5	LOC101409138
ATG5	Atg5	LOC101409129	IGFN1	Igfn1	LOC101391521	RNF6	Rnf6	LOC101406738
ATG7	Atg7	LOC101391301	IGHMBP2	Ighmbp2	LOC101392656	RNF7	Rnf7	LOC101398563
ATG9A	Atg9a	LOC101393135	IGIP	Igip	LOC101397022	RNF8	Rnf8	LOC101400595
ATG9B	Atg9b	LOC101388250	IGLON5	Iglon5	LOC106804051	RNFT1	Rnft1	LOC101392107
ATIC	Atic	LOC101400695	IGSF1	Igsf1	LOC101408671	RNFT2	Rnft2	LOC101389369
ATL1	Atl1	LOC101398444	IGSF10	Igsf10	LOC101406348	RNGTT	Rngtt	LOC101394882
ATL2	Atl2	LOC101404129	IGSF11	Igsf11	LOC101407362	RNH1	Rnh1	LOC101389566
ATL3	Atl3	LOC101402122	IGSF21	Igsf21	LOC101388827	RNLS	Rnls	LOC101399080
ATM	Atm	LOC101401401	IGSF3	Igsf3	LOC101398233	RNMT	Rnmt	LOC101395468
ATMIN	Atmin	LOC101399839	IGSF6	Igsf6	LOC101390383	RNPC3	Rnpc3	LOC101389425
ATN1	Atn1	LOC101397102	IGSF8	Igsf8	LOC101389081	RNPEP	Rnpep	LOC101399156
ATOH1	Atoh1	LOC101387987	IGSF9	Igsf9	LOC101387605	RNPEPL1	Rnpepl1	LOC101387717
ATOH7	Atoh7	LOC101390906	IGSF9B	Igsf9b	LOC101388359	RNPS1	Rnps1	LOC101408327
ATOH8	Atoh8	LOC101390684	IHH	Ihh	LOC101390981	RO60	Trove2	LOC101409015
ATOX1	Atox1	LOC101395241	IK	Ik	LOC101391283	ROBO1	Robo1	LOC101392481
ATP10A	Atp10a	LOC101394449	IKBIP	Ikbip	LOC101393583	ROBO2	Robo2	LOC101393258
ATP10B	Atp10b	LOC101402790	IKBKB	Ikbkb	LOC101392135	ROBO3	Robo3	LOC101395101
ATP11A	Atp11a	LOC101402078	IKBKE	Ikbke	LOC101393962	ROBO4	Robo4	LOC101395967
ATP11B	Atp11b	LOC101400597	IKBKG	Ikbkg	LOC101397305	ROCK1	Rock1	LOC101387185
ATP11C	Atp11c	LOC101406759	IKZF1	Ikzf1	LOC101395621	ROCK2	Rock2	LOC101387662
ATP12A	Atp12a	LOC101403245	IKZF2	Ikzf2	LOC101399202	ROGDI	Rogdi	LOC101391767
ATP13A1	Atp13a1	LOC101400500	IKZF3	Ikzf3	LOC101401510	ROM1	Rom1	LOC101398888
ATP13A2	Atp13a2	LOC101404279	IKZF4	Ikzf4	LOC101397501	ROMO1	Romo1	LOC101391171
ATP13A3	Atp13a3	LOC101393703	IKZF5	Ikzf5	LOC101405188	ROPN1	Ropn1	LOC101401910
ATP13A4	Atp13a4	LOC101395137	IL10	Il10	LOC101392604	ROPN1L	Ropn1l	LOC101404284
ATP13A5	Atp13a5	LOC101406781	IL10RA	Il10ra	LOC101401215	ROR1	Ror1	LOC101393727
ATP1A1	Atp1a1	LOC101398647	IL10RB	Il10rb	LOC101405946	ROR2	Ror2	LOC101405159
ATP1A2	Atp1a2	LOC101389330	IL11	Il11	LOC101391771	RORA	Rora	LOC101400315
ATP1A3	Atp1a3	LOC101398718	IL12A	Il12a	LOC101396071	RORB	Rorb	LOC101387498
ATP1A4	Atp1a4	LOC101389578	IL12B	Il12b	LOC101405942	RORC	Rorc	LOC101407713
ATP1B1	Atp1b1	LOC101408628	IL12RB1	Il12rb1	LOC101400854	ROS1	Ros1	LOC101408272
ATP1B2	Atp1b2	LOC101399827	IL12RB2	Il12rb2	LOC101390243	RP1	Rp1	LOC101391007
ATP1B3	Atp1b3	LOC101390787	IL13	Il13	LOC101401011	RP1L1	Rp1l1	LOC101395950

ATP1B4	Atp1b4	LOC101399068	IL13RA1	Il13ra1	LOC101394702	RP2	Rp2	LOC101396384
ATP23	Xrcc6bp1	LOC101403578	IL13RA2	Il13ra2	LOC101401362	RP9	Rp9	LOC101405711
ATP2A1	Atp2a1	LOC101405545	IL15	Il15	LOC101394719	RPA1	Rpa1	LOC101400974
ATP2A2	Atp2a2	LOC101401145	IL15RA	Il15ra	LOC101403216	RPA2	Rpa2	LOC101396528
ATP2A3	Atp2a3	LOC101388255	IL16	Il16	LOC101394918	RPA3	Rpa3	LOC101387645
ATP2B1	Atp2b1	LOC101403866	IL17A	Il17a	LOC101407681	RPAIN	Rpain	LOC101402196
ATP2B2	Atp2b2	LOC101391052	IL17B	Il17b	LOC101403832	RPAP1	Rpap1	LOC101398237
ATP2B3	Atp2b3	LOC101406106	IL17C	Il17c	LOC101389226	RPAP2	Rpap2	LOC101387265
ATP2B4	Atp2b4	LOC101405931	IL17RA	Il17ra	LOC101390601	RPAP3	Rpap3	LOC101389456
ATP2C1	Atp2c1	LOC101394945	IL17RB	Il17rb	LOC101388024	RPE	Rpe	LOC101397837
ATP2C2	Atp2c2	LOC101393742	IL17RC	Il17rc	LOC101389949	RPE65	Rpe65	LOC101393312
ATP4A	Atp4a	LOC101388703	IL17RD	Il17rd	LOC101391964	RPF1	Rpf1	LOC101408483
ATP4B	Atp4b	LOC101391473	IL17RE	Il17re	LOC101395821	RPF2	Rpf2	LOC101398579
ATP5F1A	Atp5a1	LOC101406264	IL18	Il18	LOC101392264	RPGR	Rpgr	LOC101387393
ATP5F1B	Atp5b	LOC101407249	IL18BP	Il18bp	LOC101388175	RPGRIP1	Rpgrip1	LOC101404263
ATP5F1C	Atp5c1	LOC101399639	IL18R1	Il18r1	LOC101388600	RPGRIP1L	Rpgrip1l	LOC101405513
ATP5F1D	Atp5d	LOC101388894	IL18RAP	Il18rap	LOC101388866	RPH3A	Rph3a	LOC101393809
ATP5F1E	Atp5e	LOC101402878	IL19	Il19	LOC101392341	RPH3AL	Rph3al	LOC106802278
ATP5IF1	Atpif1	LOC101407235	IL1A	Il1a	LOC101388771	RPIA	Rpia	LOC101396393
ATP5MC1	Atp5g1	LOC101399058	IL1B	Il1b	LOC101396565	RPL10	Rpl10	LOC101394276
ATP5MD	Usmg5	LOC101397324	IL1F10	Il1f10	LOC101388005	RPL10A	Rpl10a	LOC101387368
ATP5ME	Atp5k	LOC101390578	IL1R1	Il1r1	LOC101405182	RPL10L	Rpl10l	LOC101402461
ATP5MF	Atp5j2	LOC106799917	IL1R2	Il1r2	LOC101387660	RPL11	Rpl11	LOC101404280
ATP5MG	Atp5l	LOC101398009	IL1RAP	Il1rap	LOC101397564	RPL12	Rpl12	LOC101408721
ATP5PB	Atp5f1	LOC101391455	IL1RAPL1	Il1rapl1	LOC101389221	RPL13	Rpl13	LOC101405271
ATP5PD	Atp5h	LOC101390922	IL1RL1	Il1rl1	LOC101388350	RPL13A	Rpl13a	LOC101405554
ATP5PF	Atp5j	LOC101391407	IL1RL2	Il1rl2	LOC101388092	RPL14	Rpl14	LOC101388708
ATP5PO	Atp5o	LOC101407952	IL1RN	Il1rn	LOC101387571	RPL15	Rpl15	LOC101405372
ATP6AP1	Atp6ap1	LOC101394968	IL2	Il2	LOC101408023	RPL17	Rpl17	LOC101391141
ATP6AP1L	Atp6ap1l	LOC101400568	IL20	Il20	LOC101392074	RPL18	Rpl18	LOC101393253
ATP6AP2	Atp6ap2	LOC101407088	IL20RA	Il20ra	LOC101388046	RPL18A	Rpl18a	LOC101392417
ATP6V0A1	Atp6v0a1	LOC101397086	IL20RB	Il20rb	LOC101393341	RPL19	Rpl19	LOC101406567
ATP6V0A2	Atp6v0a2	LOC101395934	IL21	Il21	LOC101407598	RPL21	Rpl21	LOC101408229
ATP6V0A4	Atp6v0a4	LOC101390751	IL21R	Il21r	LOC101403000	RPL22	Rpl22	LOC101400775
ATP6V0B	Atp6v0b	LOC101388988	IL22	Il22	LOC101389366	RPL22L1	Rpl22l1	LOC101392674
ATP6V0C	Atp6v0c	LOC101407001	IL22RA1	Il22ra1	LOC101392943	RPL23	Rpl23	LOC101408318
ATP6V0D1	Atp6v0d1	LOC101391174	IL22RA2	Il22ra2	LOC101387870	RPL23A	Rpl23a	LOC101400093
ATP6V0D2	Atp6v0d2	LOC101394263	IL23A	Il23a	LOC101404194	RPL24	Rpl24	LOC101388884
ATP6V0E1	Atp6v0e	LOC101391735	IL23R	Il23r	LOC101389985	RPL26	Rpl26	LOC101391331
ATP6V0E2	Atp6v0e2	LOC101391656	IL24	Il24	LOC101402429	RPL27	Rpl27	LOC101388507
ATP6V1A	Atp6v1a	LOC101389543	IL25	Il25	LOC101400140	RPL27A	Rpl27a	LOC101389922
ATP6V1B1	Atp6v1b1	LOC101396999	IL27	Il27	LOC101387414	RPL28	Rpl28	LOC101392465
ATP6V1B2	Atp6v1b2	LOC101388991	IL27RA	Il27ra	LOC101398737	RPL29	Rpl29	LOC101399086
ATP6V1C1	Atp6v1c1	LOC101391659	IL2RA	Il2ra	LOC101402262	RPL3	Rpl3	LOC101397005
ATP6V1C2	Atp6v1c2	LOC101408320	IL2RB	Il2rb	LOC101392833	RPL30	Rpl30	LOC101404978
ATP6V1D	Atp6v1d	LOC101406532	IL2RG	Il2rg	LOC101406420	RPL31	Rpl31	LOC101408231
ATP6V1E1	Atp6v1e1	LOC101401431	IL3	Il3	LOC106803226	RPL32	Rpl32	LOC101408965
ATP6V1F	Atp6v1f	LOC101394339	IL31	Il31	LOC101401226	RPL34	Rpl34	LOC101402865
ATP6V1G1	Atp6v1g1	LOC101399628	IL31RA	Il31ra	LOC101390809	RPL35	Rpl35	LOC101400423
ATP6V1G2	Atp6v1g2	LOC101401126	IL33	Il33	LOC101401343	RPL35A	Rpl35a	LOC101405224
ATP6V1G3	Atp6v1g3	LOC101405102	IL34	Il34	LOC101390498	RPL36	Rpl36	LOC101387853
ATP6V1H	Atp6v1h	LOC101388432	IL4	Il4	LOC101389247	RPL36A	Rpl36a	LOC101399344
ATP7A	Atp7a	LOC101387346	IL4I1	Il4i1	LOC101403877	RPL36AL	Rpl36al	LOC101401340
ATP7B	Atp7b	LOC101397340	IL5	Il5	LOC101401710	RPL37	Rpl37	LOC101389950

ATP8A1	Atp8a1	LOC101401444	IL5RA	Il5ra	LOC101393012	RPL37A	Rpl37a	LOC101404553
ATP8A2	Atp8a2	LOC101406221	IL6	Il6	LOC101402308	RPL38	Rpl38	LOC101392967
ATP8B1	Atp8b1	LOC101403116	IL6ST	Il6st	LOC101403644	RPL39	Rpl39	LOC101401456
ATP8B2	Atp8b2	LOC101404740	IL7	Il7	LOC101406889	RPL3L	Rpl3l	LOC101390855
ATP8B3	Atp8b3	LOC101407209	IL7R	Il7r	LOC101401646	RPL4	Rpl4	LOC101395816
ATP8B4	Atp8b4	LOC101405471	IL9	Il9	LOC101390874	RPL5	Rpl5	LOC101408089
ATP9A	Atp9a	LOC101389980	ILDR1	Ildr1	LOC101397865	RPL6	Rpl6	LOC101394471
ATP9B	Atp9b	LOC101399103	ILDR2	Ildr2	LOC101400863	RPL7	Rpl7	LOC101399587
ATPAF1	Atpaf1	LOC101407666	ILF2	Ilf2	LOC101395522	RPL7A	Rpl7a	LOC101396882
ATPAF2	Atpaf2	LOC101391587	ILF3	Ilf3	LOC101390710	RPL7L1	Rpl7l1	LOC101389091
ATR	Atr	LOC101396242	ILK	Ilk	LOC101400737	RPL8	Rpl8	LOC101407037
ATRAID	Atraid	LOC101406233	ILKAP	Ilkap	LOC101407770	RPL9	Rpl9	LOC101393931
ATRIP	Atrip	LOC101390786	ILRUN	D17Wsu92e	LOC101391149	RPLP0	Rplp0	LOC101405606
ATRN	Atrn	LOC101408139	ILVBL	Ilvbl	LOC101394279	RPLP1	Rplp1	LOC101402320
ATRNL1	Atrnl1	LOC101391406	IMMP1L	Immp1l	LOC101401267	RPLP2	Rplp2	LOC101392401
ATRX	Atrx	LOC101401282	IMMP2L	Immp2l	LOC101396583	RPN1	Rpn1	LOC101399786
ATXN1	Atxn1	LOC101403677	IMMT	Immt	LOC101391761	RPN2	Rpn2	LOC101405246
ATXN10	Atxn10	LOC101401960	IMP3	Imp3	LOC101399789	RPP14	Rpp14	LOC101396408
ATXN1L	Atxn1l	LOC101409070	IMP4	Imp4	LOC101405852	RPP21	Rpp21	LOC106799925
ATXN2	Atxn2	LOC101397063	IMPA1	Impa1	LOC101388065	RPP25	Rpp25	LOC101396605
ATXN2L	Atxn2l	LOC101406841	IMPA2	Impa2	LOC101391209	RPP25L	Rpp25l	LOC101408212
ATXN3	Atxn3	LOC101399749	IMPACT	Impact	LOC101398731	RPP30	Rpp30	LOC101392000
ATXN7	Atxn7	LOC101402057	IMPAD1	Impad1	LOC101393829	RPP40	Rpp40	LOC101392444
ATXN7L1	Atxn7l1	LOC101407448	IMPDH1	Impdh1	LOC101396933	RPRD1A	Rprd1a	LOC101399880
ATXN7L2	Atxn7l2	LOC101407302	IMPDH2	Impdh2	LOC101397210	RPRD1B	Rprd1b	LOC101402356
ATXN7L3	Atxn7l3	LOC101391930	IMPG1	Impg1	LOC101402764	RPRD2	Rprd2	LOC101391858
ATXN7L3B	Atxn7l3b	LOC101388780	IMPG2	Impg2	LOC101395021	RPRM	Rprm	LOC101397651
AUH	Auh	LOC101404633	INA	Ina	LOC101395924	RPRML	Rprml	LOC101401850
AUNIP	Aunip	LOC101394717	INCA1	Inca1	LOC101390075	RPS10	Rps10	LOC101392072
AUP1	Aup1	LOC101401772	INCENP	Incenp	LOC101396659	RPS11	Rps11	LOC101405982
AURKA	Aurka	LOC101408039	INF2	Inf2	LOC101402454	RPS12	Rps12	LOC101404160
AURKAIP1	Aurkaip1	LOC101390816	ING1	Ing1	LOC101401819	RPS13	Rps13	LOC101405635
AURKB	Aurkb	LOC101393062	ING2	Ing2	LOC101403833	RPS14	Rps14	LOC101399731
AURKC	Aurkc	LOC101399849	ING3	Ing3	LOC101407551	RPS15	Rps15	LOC101388208
AUTS2	Auts2	LOC101394860	ING4	Ing4	LOC101403255	RPS15A	Rps15a	LOC101393690
AVEN	Aven	LOC101399445	ING5	Ing5	LOC101392266	RPS16	Rps16	LOC101389423
AVIL	Avil	LOC101403054	INHA	Inha	LOC101399385	RPS17	Rps17	LOC101389892
AVL9	Avl9	LOC101404239	INHBA	Inhba	LOC101395292	RPS18	Rps18	LOC101399799
AVP	Avp	LOC101404570	INHBB	Inhbb	LOC106800813	RPS19	Rps19	LOC101407908
AVPI1	Avpi1	LOC101393137	INHBC	Inhbc	LOC101392795	RPS19BP1	Rps19bp1	LOC101398890
AVPR1A	Avpr1a	LOC101406295	INHBE	Inhbe	LOC101393051	RPS2	Rps2	LOC101390350
AVPR1B	Avpr1b	LOC101395490	INIP	Inip	LOC101393530	RPS20	Rps20	LOC101392195
AWAT1	Awat1	LOC101387942	INKA1	Fam212a	LOC101406850	RPS21	Rps21	LOC101398519
AWAT2	Awat2	LOC101388706	INKA2	Fam212b	LOC101390373	RPS23	Rps23	LOC101400838
AXDND1	Axdnd1	LOC101393620	INO80	Ino80	LOC101395814	RPS24	Rps24	LOC101397494
AXIN1	Axin1	LOC101405100	INO80B	Ino80b	LOC101398210	RPS25	Rps25	LOC101393296
AXIN2	Axin2	LOC101400179	INO80C	Ino80c	LOC101398742	RPS26	Rps26	LOC101397924
AXL	Axl	LOC101400404	INO80D	Ino80d	LOC101399121	RPS27	Rps27	LOC101399063
AZI2	Azi2	LOC101387587	INO80E	Ino80e	LOC101390865	RPS27A	Rps27a	LOC101401859
AZIN1	Azin1	AZIN1	INPP1	Inpp1	LOC101398675	RPS27L	Rps27l	LOC101403888
AZIN2	Azin2	AZIN2	INPP4A	Inpp4a	LOC101402205	RPS29	Rps29	LOC101401609
B2M	B2m	LOC101392055	INPP4B	Inpp4b	LOC101393004	RPS3	Rps3	LOC101401946
B3GALNT2	B3galnt2	LOC101393061	INPP5A	Inpp5a	LOC101395446	RPS4X	Rps4x	LOC101399945
B3GALT2	B3galt2	LOC101408759	INPP5B	Inpp5b	LOC101398936	RPS5	Rps5	LOC101405542

B3GALT4	B3galt4	LOC101399111	INPP5D	Inpp5d	LOC101397247	RPS6	Rps6	LOC101404388
B3GAT1	B3gat1	LOC101390519	INPP5E	Inpp5e	LOC101403125	RPS6KA1	Rps6ka1	LOC101396099
B3GAT2	B3gat2	LOC101395053	INPP5F	Inpp5f	LOC101393211	RPS6KA2	Rps6ka2	LOC101390616
B3GAT3	B3gat3	LOC101399149	INPP5J	Inpp5j	LOC101392540	RPS6KA3	Rps6ka3	LOC101403858
B3GLCT	B3glct	LOC101393569	INPP5K	Inpp5k	LOC101404034	RPS6KA4	Rps6ka4	LOC101393917
B3GNT2	B3gnt2	LOC101392110	INPPL1	Inppl1	LOC101402542	RPS6KA5	Rps6ka5	LOC101397516
B3GNT3	B3gnt3	LOC101401120	INS	Ins2	LOC101397808	RPS6KA6	Rps6ka6	LOC101395265
B3GNT6	B3gnt6	LOC101406812	INSC	Insc	LOC101404497	RPS6KB1	Rps6kb1	LOC101391507
B3GNT7	B3gnt7	LOC106801428	INSIG1	Insig1	LOC101402280	RPS6KB2	Rps6kb2	LOC101405968
B3GNT8	B3gnt8	LOC101402927	INSIG2	Insig2	LOC101392071	RPS6KC1	Rps6kc1	LOC101391818
B3GNT9	B3gnt9	LOC101407876	INSL5	Insl5	LOC101388757	RPS6KL1	Rps6kl1	LOC101407858
B3GNTL1	B3gntl1	LOC101407524	INSL6	Insl6	LOC101399249	RPS7	Rps7	LOC101392814
B4GALNT1	B4galnt1	LOC101398429	INSRR	Insrr	LOC101389912	RPS8	Rps8	LOC101391973
B4GALNT2	B4galnt2	LOC101400722	INTS1	Ints1	LOC101398153	RPS9	Rps9	LOC101387169
B4GALNT3	B4galnt3	LOC101389828	INTS10	Ints10	LOC101396858	RPSA	Rpsa	LOC101387087
B4GALNT4	B4galnt4	LOC101406247	INTS12	Ints12	LOC101406712	RPTOR	Rptor	LOC101393226
B4GALT1	B4galt1	LOC101392789	INTS2	Ints2	LOC101392635	RPUSD1	Rpusd1	LOC101399933
B4GALT2	B4galt2	LOC101388721	INTS3	Ints3	LOC101396037	RPUSD2	Rpusd2	LOC101391892
B4GALT3	B4galt3	LOC101396616	INTS4	Ints4	LOC101409081	RPUSD3	Rpusd3	LOC106804067
B4GALT4	B4galt4	LOC101406826	INTS5	Ints5	LOC101399841	RPUSD4	Rpusd4	LOC101400110
B4GALT5	B4galt5	LOC101388753	INTS6	Ints6	LOC101395860	RRAD	Rrad	LOC101405683
B4GALT6	B4galt6	LOC101391388	INTS7	Ints7	LOC101388316	RRAGA	Rraga	LOC101405623
B4GALT7	B4galt7	LOC101398106	INTS8	Ints8	LOC101400525	RRAGB	Rragb	LOC101399273
B4GAT1	B4gat1	LOC101395095	INTS9	Ints9	LOC101397430	RRAGC	Rragc	LOC101400340
B9D1	B9d1	LOC101396643	INTU	Intu	LOC101388483	RRAGD	Rragd	LOC101396421
B9D2	B9d2	LOC101393943	INVS	Invs	LOC101393027	RRAS	Rras	LOC101406936
BAAT	Baat	LOC101395829	IP6K1	Ip6k1	LOC101405714	RRAS2	Rras2	LOC101401440
BABAM1	Babam1	LOC101396615	IP6K2	Ip6k2	LOC101394104	RRBP1	Rrbp1	LOC101390419
BABAM2	Bre	LOC101397360	IP6K3	Ip6k3	LOC101393960	RREB1	Rreb1	LOC101390500
BACE1	Bace1	LOC101402272	IPCEF1	Ipcef1	LOC101406684	RRH	Rrh	LOC101399982
BACE2	Bace2	LOC101395932	IPMK	Ipmk	LOC101402271	RRM1	Rrm1	LOC101407266
BACH1	Bach1	LOC101397580	IPO11	Ipo11	LOC101391556	RRM2	Rrm2	LOC101406575
BACH2	Bach2	LOC101399100	IPO13	Ipo13	LOC101388214	RRM2B	Rrm2b	LOC101389713
BAD	Bad	LOC101392198	IPO4	Ipo4	LOC101395471	RRN3	Rrn3	LOC101397378
BAG1	Bag1	LOC101407866	IPO5	Ipo5	LOC101398340	RRNAD1	Rrnad1	LOC101387920
BAG2	Bag2	LOC101399371	IPO7	Ipo7	LOC101392721	RRP1	Rrp1	LOC101403766
BAG3	Bag3	LOC101398681	IPO8	Ipo8	LOC101405821	RRP12	Rrp12	LOC101389358
BAG4	Bag4	LOC101390912	IPO9	Ipo9	LOC101399937	RRP15	Rrp15	LOC101402391
BAG5	Bag5	LOC101397183	IPP	Ipp	LOC101398573	RRP1B	Rrp1b	LOC101401743
BAG6	Bag6	LOC101398590	IPPK	Ippk	LOC101397385	RRP36	Rrp36	LOC101407939
BAHCC1	Bahcc1	LOC101402536	IQCA1L	4931409K22F	LOC101408219	RRP8	Rrp8	LOC101400476
BAHD1	Bahd1	LOC101391134	IQCB1	Iqcb1	LOC101399147	RRP9	Rrp9	LOC101396673
BAIAP2	Baiap2	LOC101394152	IQCC	Iqcc	LOC101398504	RRS1	Rrs1	LOC101399835
BAIAP2L1	Baiap2l1	LOC101407913	IQCD	Iqcd	LOC101397845	RS1	Rs1	LOC101392553
BAIAP2L2	Baiap2l2	LOC101391606	IQCE	Iqce	LOC101405198	RSAD1	Rsad1	LOC101388179
BAIAP3	Baiap3	LOC101396485	IQCF5	Iqcf5	LOC101388118	RSAD2	Rsad2	LOC101402462
BAK1	Bak1	LOC101394975	IQCF6	Iqcf6	LOC101396146	RSBN1	Rsbn1	LOC101407123
BAMBI	Bambi	LOC106801065	IQCG	Iqcg	LOC101404178	RSBN1L	Rsbn1l	LOC101399597
BANF1	Banf1	LOC101391100	IQCH	Iqch	LOC101389332	RSF1	Rsf1	LOC101408570
BANF2	Banf2	LOC101390156	IQCJ	Iqcj	LOC106803740	RSKR	BC030499	LOC101395948
BANK1	Bank1	LOC101396989	IQCK	Iqck	LOC101399001	RSL1D1	Rsl1d1	LOC101405796
BANP	Banp	LOC101388269	IQGAP1	Iqgap1	LOC101388081	RSL24D1	Rsl24d1	LOC101389176
BAP1	Bap1	LOC101401974	IQGAP2	Iqgap2	LOC101389569	RSPH1	Rsph1	LOC101398865
BARD1	Bard1	LOC101400165	IQGAP3	Iqgap3	LOC101407183	RSPH3	Rsph3b	LOC101392912

BARHL1	Barhl1	LOC101393031	IQSEC1	Iqsec1	LOC101408099	RSPH4A	Rsph4a	LOC101406370
BARHL2	Barhl2	LOC101390537	IQSEC2	Iqsec2	LOC101406270	RSPH6A	Rsph6a	LOC101400829
BARX1	Barx1	LOC101402762	IQSEC3	Iqsec3	LOC101393333	RSPH9	Rsph9	LOC101398667
BARX2	Barx2	LOC101405277	IQUB	Iqub	LOC101403004	RSPO1	Rspo1	LOC101396527
BASP1	Basp1	LOC101393609	IRAK1	Irak1	LOC101398486	RSPO2	Rspo2	LOC101396809
BATF	Batf	LOC101388742	IRAK1BP1	Irak1bp1	LOC101403284	RSPO3	Rspo3	LOC101395130
BATF2	Batf2	LOC101405007	IRAK2	Irak2	LOC101392511	RSPO4	Rspo4	LOC101408487
BATF3	Batf3	LOC101390815	IRAK3	Irak3	LOC101387636	RSPRY1	Rspry1	LOC101397262
BAX	Bax	LOC101397619	IRAK4	Irak4	LOC101407508	RSRC1	Rsrc1	LOC101392661
BAZ1A	Baz1a	LOC101398801	IREB2	Ireb2	LOC101406173	RSRC2	Rsrc2	LOC101388160
BAZ1B	Baz1b	LOC101402842	IRF1	Irf1	LOC101402232	RSRP1	Rsrp1	LOC101394211
BAZ2A	Baz2a	LOC101406641	IRF2	Irf2	LOC101405334	RSU1	Rsu1	LOC101388923
BAZ2B	Baz2b	LOC101396447	IRF2BP1	Irf2bp1	LOC101401627	RTBDN	Rtbdn	LOC101405045
BBIP1	Bbip1	LOC101407503	IRF2BP2	Irf2bp2	LOC101400178	RTCA	Rtca	LOC101393505
BBOF1	Bbof1	LOC101404185	IRF2BPL	Irf2bpl	LOC101392428	RTCB	Rtcb	LOC101390355
BBOX1	Bbox1	LOC101397958	IRF3	Irf3	LOC101407203	RTEL1	Rtel1	LOC101392621
BBS1	Bbs1	LOC101397527	IRF4	Irf4	LOC101397879	RTF1	Rtf1	LOC101397728
BBS10	Bbs10	LOC101392125	IRF5	Irf5	LOC101393490	RTKN	Rtkn	LOC101397608
BBS2	Bbs2	LOC101388944	IRF6	Irf6	LOC101404954	RTKN2	Rtkn2	LOC101396973
BBS4	Bbs4	LOC101408447	IRF7	Irf7	LOC101394514	RTL6	Ldoc1l	LOC101398975
BBS5	Bbs5	LOC101403225	IRF8	Irf8	LOC101390851	RTL8A	Cxx1b	LOC101408623
BBS7	Bbs7	LOC101387290	IRF9	Irf9	LOC101396599	RTL8C	Cxx1c	LOC101408368
BBS9	Bbs9	LOC101402050	IRGQ	Irgq	LOC101402917	RTL9	Rgag1	LOC101404762
BBX	Bbx	LOC101397353	IRS1	Irs1	LOC101406452	RTN1	Rtn1	LOC101393624
BCAM	Bcam	LOC101401302	IRS4	Irs4	LOC101394013	RTN2	Rtn2	LOC101397455
BCAN	Bcan	LOC101408843	IRX1	Irx1	LOC101392040	RTN3	Rtn3	LOC101407365
BCAP29	Bcap29	LOC101404496	IRX2	Irx2	LOC101391781	RTN4	Rtn4	LOC101408492
BCAP31	Bcap31	LOC101408103	IRX3	Irx3	LOC101406041	RTN4IP1	Rtn4ip1	LOC101387958
BCAR1	Bcar1	LOC101405895	IRX4	Irx4	LOC101391529	RTN4R	Rtn4r	LOC101391798
BCAR3	Bcar3	LOC101403721	IRX5	Irx5	LOC101406307	RTN4RL1	Rtn4rl1	LOC101400714
BCAS1	Bcas1	LOC101388077	IRX6	Irx6	LOC101406552	RTN4RL2	Rtn4rl2	LOC101388438
BCAS2	Bcas2	LOC101404515	ISCA1	Isca1	LOC101391963	RTP1	Rtp1	LOC101401825
BCAS3	Bcas3	LOC101394158	ISCA2	Isca2	LOC101406021	RTP2	Rtp2	LOC101400506
BCAT1	Bcat1	LOC101389909	ISCU	Iscu	LOC101393053	RTRAF	2700060E02F	LOC101395351
BCAT2	Bcat2	LOC101395717	ISG15	Isg15	LOC101392260	RTTN	Rttn	LOC101394277
BCCIP	Bccip	LOC101400195	ISG20	Isg20	LOC101400087	RUBCN	Rubcn	LOC101405930
BCDIN3D	Bcdin3d	LOC101406387	ISG20L2	Isg20l2	LOC101387484	RUFY1	Rufy1	LOC101394299
BCHE	Bche	LOC101400064	ISL1	Isl1	LOC101396167	RUFY2	Rufy2	LOC101389102
BCKDHA	Bckdha	LOC101402404	ISL2	Isl2	LOC101402935	RUFY3	Rufy3	LOC101388787
BCKDHB	Bckdhb	LOC101405209	ISLR	Islr	LOC101390381	RUFY4	Rufy4	LOC101398353
BCKDK	Bckdk	LOC101405107	ISLR2	Islr2	LOC101390119	RUNDC1	Rundc1	LOC101388768
BCL10	Bcl10	LOC101389616	ISM1	Ism1	LOC101395000	RUNDC3A	Rundc3a	LOC101393225
BCL11A	Bcl11a	LOC101397436	ISM2	Ism2	LOC101393128	RUNDC3B	Rundc3b	LOC101391245
BCL11B	Bcl11b	LOC101387146	ISOC1	Isoc1	LOC101407915	RUNX1	Runx1	LOC101387812
BCL2	Bcl2	LOC101387356	IST1	Ist1	LOC101407698	RUNX1T1	Runx1t1	LOC101396727
BCL2L1	Bcl2l1	LOC101391800	ISX	Isx	LOC101390274	RUNX2	Runx2	LOC101393442
BCL2L10	Bcl2l10	LOC101406768	ISY1	Isy1	LOC101387608	RUNX3	Runx3	LOC101388153
BCL2L11	Bcl2l11	LOC101392458	ISYNA1	Isyna1	LOC101388730	RUSC1	Rusc1	LOC101394930
BCL2L12	Bcl2l12	LOC101402833	ITCH	Itch	LOC101400521	RUSC2	Rusc2	LOC101408713
BCL2L13	Bcl2l13	LOC101402045	ITFG1	Itfg1	LOC101402328	RUVBL1	Ruvbl1	LOC101399096
BCL2L14	Bcl2l14	LOC101392108	ITFG2	Itfg2	LOC101392570	RUVBL2	Ruvbl2	LOC101399081
BCL2L15	Bcl2l15	LOC101406252	ITGA1	Itga1	LOC101396427	RWDD1	Rwdd1	LOC101405731
BCL2L2	Bcl2l2	LOC106799941	ITGA10	Itga10	LOC101409116	RWDD2A	Rwdd2a	LOC101408004
BCL3	Bcl3	LOC106800130	ITGA11	Itga11	LOC101389858	RWDD2B	Rwdd2b	LOC101396195

BCL6	Bcl6	LOC101400063	ITGA2	Itga2	LOC101396958	RWDD3	Rwdd3	LOC101399261
BCL6B	Bcl6b	LOC101391422	ITGA2B	Itga2b	LOC101394655	RWDD4	Rwdd4a	LOC101404093
BCL7A	Bcl7a	LOC101407513	ITGA3	Itga3	LOC101406400	RXFP1	Rxfp1	LOC101391969
BCL7B	Bcl7b	LOC101402409	ITGA4	Itga4	LOC101388237	RXFP2	Rxfp2	LOC101393826
BCL7C	Bcl7c	LOC101401702	ITGA5	Itga5	LOC101398514	RXFP3	Rxfp3	LOC101397987
BCL9	Bcl9	LOC101390454	ITGA6	Itga6	LOC101395146	RXFP4	Rxfp4	LOC101399504
BCL9L	Bcl9l	LOC101394463	ITGA7	Itga7	LOC101392356	RXRA	Rxra	LOC101399977
BCLAF1	Bclaf1	LOC101408615	ITGA8	Itga8	LOC101390133	RXRB	Rxrb	LOC101400770
BCO1	Bco1	LOC101398702	ITGA9	Itga9	LOC101400213	RXRG	Rxrg	LOC101400065
BCO2	Bco2	LOC101391574	ITGAD	Itgad	LOC101402556	RYBP	Rybp	LOC101392485
BCOR	Bcor	LOC101407533	ITGAE	Itgae	LOC101392371	RYK	Ryk	LOC101393594
BCORL1	Bcorl1	LOC101390086	ITGAL	Itgal	LOC101400477	RYR1	Ryr1	LOC101403272
BCR	Bcr	LOC101397226	ITGAM	Itgam	LOC101402304	RYR2	Ryr2	LOC101400449
BCS1L	Bcs1l	LOC101387550	ITGAV	Itgav	LOC101392611	RYR3	Ryr3	LOC101403808
BDH1	Bdh1	LOC101391906	ITGAX	Itgax	LOC101407198	S100A1	S100a1	LOC101394576
BDH2	Bdh2	LOC101398690	ITGB1	Itgb1	LOC101391310	S100A10	S100a10	LOC101408926
BDKRB2	Bdkrb2	LOC101408745	ITGB1BP1	Itgb1bp1	LOC101404483	S100A11	S100a11	LOC101409178
BDNF	Bdnf	LOC101398458	ITGB1BP2	Itgb1bp2	LOC101403264	S100A13	S100a13	LOC101394161
BDP1	Bdp1	LOC101401634	ITGB2	Itgb2	LOC101407692	S100A14	S100a14	LOC101393910
BECN1	Becn1	LOC101391005	ITGB3	Itgb3	LOC101406992	S100A16	S100a16	LOC101393655
BECN2	Becn2	LOC101392989	ITGB3BP	Itgb3bp	LOC101406802	S100A2	S100a2	LOC101393396
BEGAIN	Begain	LOC101388686	ITGB4	Itgb4	LOC101397075	S100A3	S100a3	LOC101393154
BEND3	Bend3	LOC101389334	ITGB5	Itgb5	LOC101403479	S100A4	S100a4	LOC101392896
BEND4	Bend4	LOC101400916	ITGB6	Itgb6	LOC101395065	S100A5	S100a5	LOC101392637
BEND5	Bend5	LOC101401263	ITGB7	Itgb7	LOC101390984	S100A6	S100a6	LOC101392381
BEND6	Bend6	LOC101392603	ITGB8	Itgb8	LOC101404597	S100A8	S100a8	LOC101391859
BEND7	Bend7	LOC101394131	ITGBL1	Itgbl1	LOC101404168	S100B	S100b	LOC101389368
BEST1	Best1	LOC101395787	ITIH1	Itih1	LOC101405985	S100G	S100g	LOC101398127
BEST2	Best2	LOC101403729	ITIH2	Itih2	LOC101400509	S100P	Gm40318	LOC101403770
BEST3	Best3	LOC101394555	ITIH3	Itih3	LOC101406423	S100PBP	S100pbp	LOC101403752
BET1	Bet1	LOC101402360	ITIH4	Itih4	LOC101389420	S1PR1	S1pr1	LOC101391037
BET1L	Bet1l	LOC101387092	ITIH5	Itih5	LOC101400774	S1PR2	S1pr2	LOC101408357
BEX1	Bex2	LOC106800209	ITIH6	Itih5l-ps	LOC101401810	S1PR3	S1pr3	LOC101402179
BEX2	Bex1	LOC101403559	ITK	Itk	LOC101388328	S1PR4	S1pr4	LOC101401279
BEX4	Bex4	LOC101404536	ITM2A	Itm2a	LOC101389851	SAA1	Saa2	LOC101408937
BFAR	Bfar	LOC101397629	ITM2B	Itm2b	LOC101389811	SAAL1	Saal1	LOC101408165
BFSP1	Bfsp1	LOC101391254	ITM2C	Itm2c	LOC101390232	SAC3D1	Sac3d1	LOC101399932
BFSP2	Bfsp2	LOC101391278	ITPA	Itpa	LOC101387143	SACM1L	Sacm1l	LOC101400920
BGN	Bgn	LOC101405654	ITPK1	Itpk1	LOC101402032	SACS	Sacs	LOC101402984
BHLHE40	Bhlhe40	LOC101392495	ITPKA	Itpka	LOC101397979	SAE1	Sae1	LOC101406077
BHLHE41	Bhlhe41	LOC101406403	ITPKB	Itpkb	LOC101387678	SAFB	Safb	LOC101388796
BHMG1	Gm4969	LOC101408942	ITPKC	Itpkc	LOC101397216	SAFB2	Safb2	LOC101389568
BHMT	Bhmt	LOC101407296	ITPR1	Itpr1	LOC101391893	SAG	Sag	LOC101398264
BHMT2	Bhmt2	LOC101407918	ITPR2	Itpr2	LOC101391855	SALL1	Sall1	LOC101403937
BICC1	Bicc1	LOC101401049	ITPR3	Itpr3	LOC101394456	SALL2	Sall2	LOC101395992
BICD1	Bicd1	LOC101403883	ITPRIP	Itprip	LOC101401833	SALL3	Sall3	LOC101390388
BICD2	Bicd2	LOC101397638	ITPRIPL1	Itpripl1	LOC101398618	SALL4	Sall4	LOC101389708
BICDL1	Ccdc64	LOC101396017	ITPRIPL2	Itpripl2	LOC101392490	SAMD1	Samd1	LOC101399536
BICDL2	Ccdc64b	LOC101392199	ITSN1	Itsn1	LOC101407512	SAMD10	Samd10	LOC101389611
BICRA	Gltscr1	LOC101408773	ITSN2	Itsn2	LOC101396235	SAMD11	Samd11	LOC101387793
BICRAL	Gltscr1l	LOC101388822	IVD	Ivd	LOC101390709	SAMD12	Samd12	LOC101404722
BID	Bid	LOC101391353	IVNS1ABP	Ivns1abp	LOC101396789	SAMD14	Samd14	LOC101407269
BIK	Bik	LOC106803058	IWS1	Iws1	LOC101400946	SAMD15	Samd15	LOC101392873
BIN1	Bin1	LOC101398086	IYD	Iyd	LOC101402400	SAMD3	Samd3	LOC101399623

BIN2	Bin2	LOC101393550	IZUMO1	Izumo1	LOC101400212	SAMD4A	Samd4	LOC101407495
BIN3	Bin3	LOC101402843	IZUMO1R	Izumo1r	LOC101397057	SAMD4B	Samd4b	LOC101387341
BIRC2	Birc2	LOC101388067	IZUMO2	Izumo2	LOC101404925	SAMD7	Samd7	LOC101395661
BIRC3	Birc3	LOC101388322	IZUMO3	Izumo3	LOC101399295	SAMD8	Samd8	LOC101404630
BIRC5	Birc5	LOC101388682	IZUMO4	Izumo4	LOC101406856	SAMHD1	Samhd1	LOC101406131
BIRC6	Birc6	LOC101389054	JADE1	Jade1	LOC101403046	SAMM50	Samm50	LOC101397790
BIVM	Bivm	LOC101406271	JADE2	Jade2	LOC101395199	SAMSN1	Samsn1	LOC101394687
BLCAP	Blcap	LOC101403838	JADE3	Jade3	LOC101395172	SAP130	Sap130	LOC101402685
BLK	Blk	LOC101393905	JAG1	Jag1	LOC101396118	SAP18	Sap18	LOC101401249
BLM	Blm	LOC101387817	JAG2	Jag2	LOC101392016	SAP25	Sap25	LOC106803961
BLMH	Blmh	LOC101398791	JAGN1	Jagn1	LOC101390888	SAP30	Sap30	LOC101392102
BLNK	Blnk	LOC101408897	JAK1	Jak1	LOC101408308	SAP30BP	Sap30bp	LOC101396468
BLOC1S1	Bloc1s1	LOC101393050	JAK2	Jak2	LOC101398822	SAP30L	Sap30l	LOC101391736
BLOC1S2	Bloc1s2	LOC101400346	JAK3	Jak3	LOC101392668	SAPCD1	Sapcd1	LOC101394806
BLOC1S4	Bloc1s4	LOC101403322	JAKMIP1	Jakmip1	LOC101404900	SAPCD2	Sapcd2	LOC106800741
BLOC1S5	Bloc1s5	LOC101406043	JAKMIP2	Jakmip2	LOC101394949	SAR1A	Sar1a	LOC101403570
BLOC1S6	Bloc1s6	LOC101396256	JAKMIP3	Jakmip3	LOC101396487	SAR1B	Sar1b	LOC101394950
BLVRA	Blvra	LOC101408328	JAM2	Jam2	LOC101390746	SARAF	Saraf	LOC101401835
BLVRB	Blvrb	LOC101395726	JAM3	Jam3	LOC101388611	SARDH	Sardh	LOC101399279
BLZF1	Blzf1	LOC101408373	JAML	Amica1	LOC101400164	SARM1	Sarm1	LOC101400365
BMF	Bmf	LOC101388806	JARID2	Jarid2	LOC101403413	SARNP	Sarnp	LOC101393715
BMI1	Bmi1	LOC101404443	JAZF1	Jazf1	LOC101390612	SARS2	Sars2	LOC101407565
BMP1	Bmp1	LOC101405994	JCHAIN	Jchain	LOC101388284	SART1	Sart1	LOC101390264
BMP10	Bmp10	LOC101404309	JDP2	Jdp2	LOC101388480	SART3	Sart3	LOC101392436
BMP15	Bmp15	LOC101399924	JHY	Jhy	LOC101404551	SASH1	Sash1	LOC101389495
BMP2	Bmp2	LOC101400007	JKAMP	Jkamp	LOC101393367	SASH3	Sash3	LOC101391762
BMP2K	Bmp2k	LOC101389212	JMJD1C	Jmjd1c	LOC101394815	SASS6	Sass6	LOC101394518
BMP3	Bmp3	LOC101391499	JMJD4	Jmjd4	LOC101407122	SAT1	Sat1	LOC101408149
BMP4	Bmp4	LOC101405065	JMJD6	Jmjd6	LOC101406213	SAT2	Sat2	LOC101400092
BMP5	Bmp5	LOC101400860	JMJD7	Jmjd7	LOC106799926	SATB1	Satb1	LOC101401787
BMP6	Bmp6	LOC101406309	JMJD8	Jmjd8	LOC101399678	SATB2	Satb2	LOC101407150
BMP7	Bmp7	LOC101406983	JMY	Jmy	LOC101407021	SATL1	Satl1	LOC101400016
BMPER	Bmper	LOC101401625	JOSD1	Josd1	LOC101394851	SAV1	Sav1	LOC101398207
BMPR1A	Bmpr1a	LOC101390998	JOSD2	Josd2	LOC101391692	SAXO1	Saxo1	LOC101398970
BMPR1B	Bmpr1b	LOC101391077	JPH1	Jph1	LOC101402547	SAXO2	Saxo2	LOC101393466
BMPR2	Bmpr2	LOC101391315	JPH3	Jph3	LOC101389546	SAYSD1	Saysd1	LOC101398589
BMS1	Bms1	LOC101403324	JPH4	Jph4	LOC101400932	SBF1	Sbf1	LOC101402637
BMT2	Bmt2	LOC101394100	JPT1	Hn1	LOC101391845	SBF2	Sbf2	LOC101394096
BMX	Bmx	LOC101395699	JPT2	Hn1l	LOC101394001	SBK2	Sbk2	LOC101393667
BNC1	Bnc1	LOC101403236	JRKL	Jrkl	LOC101391999	SBK3	Sbk3	LOC101393924
BNC2	Bnc2	LOC101407439	JSRP1	Jsrp1	LOC101405823	SBNO1	Sbno1	LOC101392541
BNIP1	Bnip1	LOC101391068	JTB	Jtb	LOC101397944	SBSN	Sbsn	LOC101392834
BNIP2	Bnip2	LOC101399097	JUN	Jun	LOC101400112	SBSPON	Sbspon	LOC101399064
BNIP3	Bnip3	LOC101389305	JUNB	Junb	LOC101404261	SC5D	Sc5d	LOC101405600
BNIP3L	Bnip3l	LOC101405779	JUP	Jup	LOC101405178	SCAF1	Scaf1	LOC101402562
BNIP5	4930539E08F	LOC101406627	KALRN	Kalrn	LOC101402167	SCAF11	Scaf11	LOC101388074
BNIPL	Bnipl	LOC101396830	KANK1	Kank1	LOC101393592	SCAF4	Scaf4	LOC101403312
BOC	Boc	LOC101402990	KANK2	Kank2	LOC101392412	SCAF8	Scaf8	LOC101408516
BOD1	Bod1	LOC101389795	KANK3	Kank3	LOC101404538	SCAI	Scai	LOC101401388
BOK	Bok	LOC101390741	KANK4	Kank4	LOC101404104	SCAMP1	Scamp1	LOC101408440
BOLA2B	Bola2	LOC101398900	KANSL1	Kansl1	LOC101405685	SCAMP2	Scamp2	LOC101395387
BOLA3	Bola3	LOC101407711	KANSL1L	Kansl1l	LOC101398101	SCAMP3	Scamp3	LOC101393573
BOLL	Boll	LOC101406382	KANSL2	Kansl2	LOC101395929	SCAMP4	Scamp4	LOC101406686
BOP1	Bop1	LOC101403026	KANSL3	Kansl3	LOC101399062	SCAMP5	Scamp5	LOC101392147

BORA	Bora	LOC101407320	KAT14	Csrp2bp	LOC101388856	SCAP	Scap	LOC101408776
BORCS5	Borcs5	LOC101393071	KAT2A	Kat2a	LOC101400982	SCAPER	Scaper	LOC101402672
BORCS6	Borcs6	LOC101393647	KAT2B	Kat2b	LOC101395633	SCARA3	Scara3	LOC101402027
BORCS7	Borcs7	LOC101394384	KAT5	Kat5	LOC101407639	SCARA5	Scara5	LOC101400715
BORCS8	Borcs8	LOC101404270	KAT6A	Kat6a	LOC101391125	SCARB1	Scarb1	LOC101396725
BPGM	Bpgm	LOC101399128	KAT6B	Kat6b	LOC101406634	SCARB2	Scarb2	LOC101406902
BPHL	Bphl	LOC101407620	KAT7	Kat7	LOC101404305	SCARF1	Scarf1	LOC101402888
BPI	Bpi	LOC101397258	KAT8	Kat8	LOC101405547	SCARF2	Scarf2	LOC101408446
BPIFA1	Bpifa1	LOC106803881	KATNA1	Katna1	LOC101388117	SCCPDH	Sccpdh	LOC101389650
BPIFA2	Bpifa2	LOC106800127	KATNAL1	Katnal1	LOC101390930	SCD	Scd3	LOC101401051
BPIFA3	Bpifa3	LOC106803885	KATNAL2	Katnal2	LOC101388554	SCEL	Scel	LOC101389864
BPIFB1	Bpifb1	LOC101395557	KATNB1	Katnb1	LOC101394995	SCFD1	Scfd1	LOC101406608
BPIFB2	Bpifb2	LOC101408266	KATNBL1	Katnbl1	LOC101403021	SCFD2	Scfd2	LOC101389412
BPIFB3	Bpifb3	LOC101407576	KAZALD1	Kazald1	LOC101405158	SCG2	Scg2	LOC101403572
BPIFB4	Bpifb4	LOC101407307	KAZN	Kazn	LOC101408114	SCG3	Scg3	LOC101407924
BPIFB6	Bpifb6	LOC101407839	KBTBD12	Kbtbd12	LOC101398574	SCG5	Scg5	LOC101405129
BPIFC	Bpifc	LOC101390604	KBTBD13	Kbtbd13	LOC101389505	SCGB1A1	Scgb1a1	LOC101397443
BPNT1	Bpnt1	LOC101396320	KBTBD2	Kbtbd2	LOC101403972	SCGB1C1	Scgb1c1	LOC101408518
BPTF	Bptf	LOC101396467	KBTBD3	Kbtbd3	LOC101404962	SCGB2A2	LOC1026391	LOC101398213
BRAF	Braf	LOC101408218	KBTBD4	Kbtbd4	LOC101407094	SCGB3A1	Scgb3a1	LOC101408811
BRAP	Brap	LOC101396804	KBTBD6	Kbtbd6	LOC101401502	SCGB3A2	Scgb3a2	LOC101394435
BRAT1	Brat1	LOC101395547	KBTBD7	Kbtbd7	LOC101401759	SCGN	Scgn	LOC101392096
BRCA1	Brca1	LOC101387147	KBTBD8	Kbtbd8	LOC101404859	SCHIP1	Schip1	LOC101394956
BRCA2	Brca2	LOC101408230	KCMF1	Kcmf1	LOC101399235	SCIMP	Scimp	LOC101391082
BRCC3	Brcc3	LOC101403899	KCNA1	Kcna1	LOC101387408	SCIN	Scin	LOC101390329
BRD1	Brd1	LOC101404584	KCNA10	Kcna10	LOC101395034	SCLT1	Sclt1	LOC101402784
BRD2	Brd2	LOC101402164	KCNA4	Kcna4	LOC101405976	SCLY	Scly	LOC101405602
BRD3	Brd3	LOC106800773	KCNA5	Kcna5	LOC101387167	SCMH1	Scmh1	LOC101390397
BRD4	Brd4	LOC101399626	KCNA7	Kcna7	LOC101399603	SCML4	Scml4	LOC101390122
BRD7	Brd7	LOC101395160	KCNAB1	Kcnab1	LOC101389072	SCN10A	Scn10a	LOC101406081
BRD8	Brd8	LOC101406602	KCNAB2	Kcnab2	LOC101399804	SCN11A	Scn11a	LOC101400030
BRD9	Brd9	LOC101389059	KCNAB3	Kcnab3	LOC101397339	SCN1A	Scn1a	LOC101407865
BRDT	Brdt	LOC101389850	KCNB1	Kcnb1	LOC101395416	SCN1B	Scn1b	LOC101391022
BRF1	Brf1	LOC101404473	KCNC1	Kcnc1	LOC101407649	SCN2A	Scn2a1	LOC101387300
BRF2	Brf2	LOC101388929	KCNC2	Kcnc2	LOC101389052	SCN2B	Scn2b	LOC101400435
BRI3	Bri3	LOC101408173	KCNC3	Kcnc3	LOC106800140	SCN3A	Scn3a	LOC101388071
BRI3BP	Bri3bp	LOC101397256	KCNC4	Kcnc4	LOC101396505	SCN3B	Scn3b	LOC101403396
BRICD5	Bricd5	LOC101395013	KCND1	Kcnd1	LOC101407891	SCN4A	Scn4a	LOC101394070
BRINP1	Brinp1	LOC101401822	KCND2	Kcnd2	LOC101408247	SCN4B	Scn4b	LOC101400694
BRINP2	Brinp2	LOC101393363	KCND3	Kcnd3	LOC101389666	SCN5A	Scn5a	LOC101405117
BRIP1	Brip1	LOC101394748	KCNE2	Kcne2	LOC101408475	SCN7A	Scn7a	LOC101406978
BRIX1	Brix1	LOC101399969	KCNE4	Kcne4	LOC101403305	SCN8A	Scn8a	LOC101395672
BRMS1	Brms1	LOC101394849	KCNE5	Kcne1l	LOC101394771	SCN9A	Scn9a	LOC101407245
BRMS1L	Brms1l	LOC101388352	KCNF1	Kcnf1	LOC101409007	SCNM1	Scnm1	LOC101399407
BROX	Brox	LOC101399427	KCNG1	Kcng1	LOC101391170	SCNN1A	Scnn1a	LOC101407541
BRPF1	Brpf1	LOC101392507	KCNG2	Kcng2	LOC101390889	SCNN1B	Scnn1b	LOC101388807
BRPF3	Brpf3	LOC101406014	KCNG3	Kcng3	LOC101404578	SCNN1G	Scnn1g	LOC101389080
BRS3	Brs3	LOC101401982	KCNG4	Kcng4	LOC101393999	SCO1	Sco1	LOC101406808
BRSK1	Brsk1	LOC101391269	KCNH1	Kcnh1	LOC101407326	SCO2	Sco2	LOC101408423
BRSK2	Brsk2	LOC101390452	KCNH2	Kcnh2	LOC101388754	SCOC	Scoc	LOC101396710
BRWD1	Brwd1	LOC101400082	KCNH3	Kcnh3	LOC101404293	SCP2	Scp2	LOC101388192
BRWD3	Brwd3	LOC101390796	KCNH4	Kcnh4	LOC101400460	SCP2D1	Scp2d1	LOC101408406
BSCL2	Bscl2	LOC101401169	KCNH5	Kcnh5	LOC101398261	SCPEP1	Scpep1	LOC101402718
BSDC1	Bsdc1	LOC101401578	KCNH6	Kcnh6	LOC101388421	SCRG1	Scrg1	LOC101390252

BSG	Bsg	LOC101393003	KCNH7	Kcnh7	LOC101390142	SCRIB	Scrib	LOC101395214
BSN	Bsn	LOC101403976	KCNH8	Kcnh8	LOC101402399	SCRN1	Scrn1	LOC101388528
BSND	Bsnd	LOC101397358	KCNIP1	Kcnip1	LOC101395589	SCRN2	Scrn2	LOC101391754
BSPRY	Bspry	LOC101396091	KCNIP2	Kcnip2	LOC101408471	SCRN3	Scrn3	LOC101397320
BST1	Bst1	LOC101389468	KCNIP3	Kcnip3	LOC101396034	SCRT1	Scrt1	LOC101402765
BSX	Bsx	LOC101404285	KCNIP4	Kcnip4	LOC101394564	SCTR	Sctr	LOC101393875
BTAF1	Btaf1	LOC101394215	KCNJ10	Kcnj10	LOC101388550	SCUBE1	Scube1	LOC101397197
BTBD1	Btbd1	LOC101392013	KCNJ11	Kcnj11	LOC101406415	SCUBE2	Scube2	LOC101398389
BTBD10	Btbd10	LOC101400202	KCNJ12	Kcnj12	LOC101387733	SCUBE3	Scube3	LOC101389092
BTBD11	Btbd11	LOC101389404	KCNJ13	Kcnj13	LOC101396444	SCYL1	Scyl1	LOC101405445
BTBD16	Btbd16	LOC101401667	KCNJ14	Kcnj14	LOC101391783	SCYL2	Scyl2	LOC101397872
BTBD17	Btbd17	LOC101407700	KCNJ15	Kcnj15	LOC101392172	SCYL3	Scyl3	LOC101406017
BTBD18	Btbd18	LOC101391866	KCNJ2	Kcnj2	LOC101404827	SDAD1	Sdad1	LOC101405167
BTBD2	Btbd2	LOC101407661	KCNJ4	Kcnj4	LOC101392388	SDC1	Sdc1	LOC101400288
BTBD3	Btbd3	LOC101395513	KCNJ8	Kcnj8	LOC101408411	SDC2	Sdc2	LOC101403060
BTBD6	Btbd6	LOC101405089	KCNJ9	Kcnj9	LOC101388808	SDC3	Sdc3	LOC101392080
BTBD7	Btbd7	LOC101403335	KCNK1	Kcnk1	LOC101390668	SDC4	Sdc4	LOC101409065
BTBD8	Btbd8	LOC101388296	KCNK10	Kcnk10	LOC101401472	SDCBP	Sdcbp	LOC101394841
BTBD9	Btbd9	LOC101399547	KCNK12	Kcnk12	LOC101394323	SDCBP2	Sdcbp2	LOC101395774
BTC	Btc	LOC101396587	KCNK13	Kcnk13	LOC101395952	SDCCAG8	Sdccag8	LOC101394504
BTD	Btd	LOC101398642	KCNK15	Kcnk15	LOC101390989	SDE2	Sde2	LOC101407727
BTF3	Btf3	LOC101393764	KCNK18	Kcnk18	LOC101393803	SDF2	Sdf2	LOC101395427
BTF3L4	Btf3l4	LOC101407193	KCNK2	Kcnk2	LOC101393705	SDF2L1	Sdf2l1	LOC101403989
BTG2	Btg2	LOC101407490	KCNK3	Kcnk3	LOC101389308	SDF4	Sdf4	LOC101389346
BTG3	Btg3	LOC101396854	KCNK4	Kcnk4	LOC101392901	SDHA	Sdha	LOC101408856
BTG4	Btg4	LOC101397495	KCNK5	Kcnk5	LOC101398342	SDHAF1	Sdhaf1	LOC101397205
BTK	Btk	LOC101400127	KCNK7	Kcnk7	LOC101405897	SDHAF2	Sdhaf2	LOC101392713
BTLA	Btla	LOC101392815	KCNMA1	Kcnma1	LOC101398941	SDHAF3	Sdhaf3	LOC101407258
BTN1A1	Btn1a1	LOC101404344	KCNMB1	Kcnmb1	LOC101396536	SDHAF4	Sdhaf4	LOC101390307
BTN2A1	Btn2a2	LOC101404616	KCNMB2	Kcnmb2	LOC101407854	SDHB	Sdhb	LOC101404545
BTNL2	Btnl2	LOC106800895	KCNMB3	Kcnmb3	LOC101406880	SDHC	Sdhc	LOC101399715
BTRC	Btrc	LOC101405670	KCNMB4	Kcnmb4	LOC101395673	SDHD	Sdhd	LOC101392682
BUB1	Bub1	LOC101398619	KCNN1	Kcnn1	LOC101391221	SDK1	Sdk1	LOC101404927
BUB1B	Bub1b	LOC101389078	KCNN2	Kcnn2	LOC101399956	SDK2	Sdk2	LOC101392701
BUB3	Bub3	LOC101403963	KCNN3	Kcnn3	LOC101406223	SDR16C5	Sdr16c5	LOC101393324
BUD13	Bud13	LOC101406385	KCNN4	Kcnn4	LOC101390018	SDR39U1	Sdr39u1	LOC101389760
BUD23	Wbscr22	LOC101401197	KCNQ1	Kcnq1	LOC101388794	SDR42E1	Sdr42e1	LOC101397695
BUD31	Bud31	LOC101405559	KCNQ2	Kcnq2	LOC101394826	SDR42E2	Gm5737	LOC101401017
BVES	Bves	LOC101407583	KCNQ3	Kcnq3	LOC101390830	SDR9C7	Sdr9c7	LOC101387378
BYSL	Bysl	LOC101392419	KCNQ4	Kcnq4	LOC101402005	SDS	Sds	LOC101397582
BZW1	Bzw1	LOC101387292	KCNQ5	Kcnq5	LOC101389779	SDSL	Sdsl	LOC101390987
BZW2	Bzw2	LOC101393981	KCNRG	Kcnrg	LOC101394570	SEBOX	Sebox	LOC101399052
C10orf120	4933402N03	LOC106803233	KCNS1	Kcns1	LOC101389281	SEC11A	Sec11a	LOC101389122
C10orf53	1700024G13	LOC106802193	KCNS2	Kcns2	LOC101406039	SEC11C	Sec11c	LOC101405581
C10orf82	1700019N19	LOC101392170	KCNS3	Kcns3	LOC101402554	SEC13	Sec13	LOC101391639
C10orf88	2310057M21	LOC101405706	KCNT1	Kcnt1	LOC101405850	SEC14L1	Sec14l1	LOC101408404
C10orf90	D7Ertd443e	LOC101399680	KCNT2	Kcnt2	LOC101408240	SEC14L2	Sec14l2	LOC101397415
C10orf99	2610528A11	LOC101394832	KCNU1	Kcnu1	LOC101402182	SEC14L3	Sec14l3	LOC101396897
C11orf1	1110032A03	LOC101395497	KCNV1	Kcnv1	LOC101400878	SEC14L4	Sec14l4	LOC101389886
C11orf16	BC051019	LOC101398142	KCNV2	Kcnv2	LOC101395195	SEC14L5	Sec14l5	LOC101390599
C11orf24	1810055G02	LOC101408422	KCP	Kcp	LOC101398904	SEC16A	Sec16a	LOC101402683
C11orf42	Gm5901	LOC101394681	KCTD1	Kctd1	LOC101395729	SEC16B	Sec16b	LOC101393121
C11orf49	1110051M20	LOC101400632	KCTD10	Kctd10	LOC101396107	SEC22A	Sec22a	LOC101400687
C11orf52	2310030G06	LOC101394721	KCTD11	Kctd11	LOC106802263	SEC22B	Sec22b	LOC101392659

C11orf53	1810046K07F	LOC101398425	KCTD12	Kctd12	LOC101388394	SEC22C	Sec22c	LOC101392317
C11orf54	4931406C07F	LOC101398352	KCTD13	Kctd13	LOC101392575	SEC23A	Sec23a	LOC101407271
C11orf65	4930550C14F	LOC101401665	KCTD15	Kctd15	LOC101404238	SEC23B	Sec23b	LOC101409172
C11orf68	AI837181	LOC101389480	KCTD16	Kctd16	LOC101402316	SEC23IP	Sec23ip	LOC101399205
C11orf74	B230118H07	LOC101393400	KCTD17	Kctd17	LOC101398456	SEC24A	Sec24a	LOC101394690
C11orf80	Gm960	LOC101406579	KCTD18	Kctd18	LOC101387977	SEC24B	Sec24b	LOC101401317
C11orf86	2010003K11F	LOC101402730	KCTD19	Kctd19	LOC101389716	SEC24C	Sec24c	LOC101390401
C11orf87	AI593442	LOC101400344	KCTD2	Kctd2	LOC101394999	SEC24D	Sec24d	LOC101391233
C11orf88	4833427G06F	LOC101397246	KCTD20	Kctd20	LOC101404437	SEC31A	Sec31a	LOC101401329
C11orf91	A930018P22F	LOC101403164	KCTD3	Kctd3	LOC101393248	SEC31B	Sec31b	LOC101388325
C11orf94	1700029I15R	LOC101393834	KCTD4	Kctd4	LOC101407351	SEC61A1	Sec61a1	LOC101398836
C11orf95	2700081O15F	LOC101402382	KCTD5	Kctd5	LOC101405900	SEC61A2	Sec61a2	LOC101396270
C11orf96	Gm13889	LOC101390849	KCTD6	Kctd6	LOC101397884	SEC61B	Sec61b	LOC101392068
C11orf97	1700012B09F	LOC101396011	KCTD8	Kctd8	LOC101402134	SEC61G	Sec61g	LOC101399239
C11orf98	1810009A15F	LOC101398704	KCTD9	Kctd9	LOC101407527	SEC62	Sec62	LOC101395402
C12orf10	Myg1	LOC101387634	KDELR1	Kdelr1	LOC101391277	SEC63	Sec63	LOC101397901
C12orf29	4930430F08F	LOC101400995	KDELR2	Kdelr2	LOC101389169	SECISBP2	Secisbp2	LOC101402962
C12orf4	D6Wsu163e	LOC101389153	KDELR3	Kdelr3	LOC101392820	SECISBP2L	Secisbp2l	LOC101401547
C12orf43	2210016L21F	LOC101394728	KDF1	Kdf1	LOC101399287	SEH1L	Seh1l	LOC101393947
C12orf45	D10Wsu102e	LOC101388969	KDM1A	Kdm1a	LOC101399897	SEL1L	Sel1l	LOC101400071
C12orf49	2410131K14F	LOC101396805	KDM1B	Kdm1b	LOC101399823	SEL1L2	Sel1l2	LOC101393818
C12orf50	1700017N19F	LOC101400735	KDM2A	Kdm2a	LOC101403253	SEL1L3	Sel1l3	LOC101408772
C12orf56	D930020B18	LOC101408643	KDM2B	Kdm2b	LOC101404364	SELE	Sele	LOC101406705
C12orf57	Grcc10	LOC101396836	KDM3A	Kdm3a	LOC101393079	SELENBP1	Selenbp1	LOC101402896
C12orf65	2810006K23F	LOC101391496	KDM3B	Kdm3b	LOC101404065	SELL	Sell	LOC101406967
C12orf66	BC048403	LOC101406794	KDM4A	Kdm4a	LOC101408091	SELP	Selp	LOC101407232
C12orf75	1500009L16F	LOC106800449	KDM4B	Kdm4b	LOC101390789	SEMA3A	Sema3a	LOC101388846
C13orf42	Gm4131	LOC101405522	KDM4C	Kdm4c	LOC101391600	SEMA3B	Sema3b	LOC101388534
C13orf46	1700029H14F	LOC106800690	KDM5A	Kdm5a	LOC101394422	SEMA3C	Sema3c	LOC101393926
C14orf119	1700123O20F	LOC101407304	KDM5B	Kdm5b	LOC101396574	SEMA3D	Sema3d	LOC101389119
C14orf132	D430019H16	LOC106802464	KDM5C	Kdm5c	LOC101405847	SEMA3E	Sema3e	LOC101388583
C14orf180	A530016L24F	LOC101401508	KDM6A	Kdm6a	LOC101400536	SEMA3F	Sema3f	LOC101387336
C14orf28	Gm527	LOC101404120	KDM6B	Kdm6b	LOC101388256	SEMA3G	Sema3g	LOC101402655
C14orf39	4930447C04F	LOC101408803	KDM7A	Kdm7a	LOC101393722	SEMA4A	Sema4a	LOC101402628
C14orf93	4931414P19F	LOC101408092	KDM8	Kdm8	LOC101402222	SEMA4B	Sema4b	LOC101405341
C15orf39	1700017B05F	LOC101397132	KDR	Kdr	LOC101391691	SEMA4C	Sema4c	LOC101400465
C15orf40	3110040N11F	LOC101391750	KDSR	Kdsr	LOC101387614	SEMA4D	Sema4d	LOC101403226
C15orf41	BC052040	LOC101407029	KEAP1	Keap1	LOC101389431	SEMA4F	Sema4f	LOC101403166
C15orf48	AA467197	LOC101395734	KEL	Kel	LOC101403583	SEMA4G	Sema4g	LOC101403398
C15orf61	2300009A05F	LOC101398078	KERA	Kera	LOC101404664	SEMA5A	Sema5a	LOC101394011
C15orf62	Gm14137	LOC101393600	KHDRBS1	Khdrbs1	LOC101396365	SEMA5B	Sema5b	LOC101400155
C15orf65	Ccpg1os	LOC106800431	KHDRBS2	Khdrbs2	LOC101398588	SEMA6A	Sema6a	LOC101396065
C16orf58	BC017158	LOC101408504	KHDRBS3	Khdrbs3	LOC101388164	SEMA6B	Sema6b	LOC101398324
C16orf70	D230025D16	LOC101407617	KHK	Khk	LOC101407984	SEMA6C	Sema6c	LOC101398131
C16orf72	1810013L24F	LOC101388357	KHNYN	Khnyn	LOC101390030	SEMA6D	Sema6d	LOC101397633
C16orf74	1190005I06R	LOC101391515	KHSRP	Khsrp	LOC101395984	SEMA7A	Sema7a	LOC101391460
C16orf78	4933402J07R	LOC101394653	KIAA0100	2610507B11F	LOC101395690	SENP1	Senp1	LOC101392354
C16orf82	4930571K23F	LOC101396580	KIAA0232	D5Ertd579e	LOC101403063	SENP2	Senp2	LOC101408202
C16orf86	4933405L10F	LOC101393814	KIAA0319	D130043K22	LOC101394996	SENP3	Senp3	LOC101401417
C16orf87	4921524J17R	LOC101404427	KIAA0319L	AU040320	LOC101388663	SENP5	Senp5	LOC101389005
C16orf89	AU021092	LOC101389641	KIAA0355	4931406P16F	LOC101405449	SENP6	Senp6	LOC101391710
C16orf90	1700037C18F	LOC101399844	KIAA0408	9330159F19F	LOC101396425	SENP7	Senp7	LOC101387685
C16orf91	BC003965	LOC101395707	KIAA0513	6430548M08	LOC101392116	SENP8	Senp8	LOC101405647
C16orf92	4930451I11R	LOC101399426	KIAA0556	D430042O09	LOC101397107	SEPHS1	Sephs1	LOC101394377

C16orf96	4930562C15F	LOC101390177	KIAA0586	2700049A03F	LOC101392261	SEPHS2	Sephs2	LOC101398057
C17orf107	4930544D05I	LOC106802267	KIAA0753	4933427D14I	LOC101406047	SEPSECS	Sepsecs	LOC101397072
C17orf49	0610010K14F	LOC101391668	KIAA0895	9530077C05F	LOC101399336	SEPTIN1	1-Sep	LOC101397106
C17orf50	1700020L24F	LOC101397088	KIAA0895L	4931428F04F	LOC101387218	SEPTIN10	10-Sep	LOC101407270
C17orf64	1700125H20I	LOC101395005	KIAA0930	5031439G07I	LOC101398706	SEPTIN11	11-Sep	LOC101408127
C17orf75	5730455P16F	LOC101408783	KIAA1109	4932438A13F	LOC101408729	SEPTIN12	12-Sep	LOC101392030
C17orf80	D11Wsu47e	LOC101392184	KIAA1143	1110059G10I	LOC101398064	SEPTIN14	14-Sep	LOC101409020
C17orf97	1700016K19F	LOC101397264	KIAA1191	4833439L19F	LOC101409060	SEPTIN2	2-Sep	LOC101390057
C17orf98	1700001P01F	LOC101408573	KIAA1211L	2010300C02F	LOC101403859	SEPTIN3	3-Sep	LOC101409012
C18orf21	2700062C07F	LOC101399624	KIAA1217	Etl4	LOC101402591	SEPTIN4	4-Sep	LOC101408144
C18orf25	8030462N17I	LOC101407672	KIAA1324L	9330182L06F	LOC101389615	SEPTIN5	5-Sep	LOC101394447
C18orf32	BC031181	LOC101391711	KIAA1328	AW554918	LOC101402678	SEPTIN6	6-Sep	LOC101398330
C18orf54	4930503L19F	LOC101398167	KIAA1549	D630045J12F	LOC101395249	SEPTIN7	7-Sep	LOC101399856
C18orf63	Gm17266	LOC101389337	KIAA1549L	D430041D05	LOC101391864	SEPTIN8	8-Sep	LOC101399524
C19orf12	1600014C10F	LOC101387681	KIAA1614	BC034090	LOC101393364	SEPTIN9	9-Sep	LOC101408658
C19orf18	2900092C05F	LOC101395274	KIAA1755	D630003M21	LOC101397507	SERAC1	Serac1	LOC101388369
C19orf25	2310011J03R	LOC101387947	KIAA1841	0610010F05F	LOC101396125	SERBP1	Serbp1	LOC101390993
C19orf33	2200002D01I	LOC106803721	KIAA1958	E130308A19I	LOC101393116	SERF2	Serf2	LOC101388898
C19orf38	AB124611	LOC106803927	KIAA2012	Gm973	LOC101392430	SERGEF	Sergef	LOC101400999
C19orf44	1700030K09F	LOC101404537	KIAA2013	2510039O18I	LOC101405494	SERINC1	Serinc1	LOC101390634
C19orf47	2310022A10F	LOC101393345	KIAA2026	9930021J03R	LOC101393477	SERINC2	Serinc2	LOC101393882
C19orf53	D8Ertd738e	LOC101387863	KIDINS220	Kidins220	LOC101403250	SERINC3	Serinc3	LOC101392006
C19orf54	BC024978	LOC101397464	KIF11	Kif11	LOC101395585	SERINC4	Serinc4	LOC101403373
C19orf57	4930432K21F	LOC101397981	KIF12	Kif12	LOC101387195	SERINC5	Serinc5	LOC101398231
C19orf67	1700067K01F	LOC101399267	KIF13A	Kif13a	LOC101402886	SERP1	Serp1	LOC101387331
C19orf71	4930404N11I	LOC106803683	KIF13B	Kif13b	LOC101393906	SERPINA11	Serpina11	LOC101406814
C19orf84	Gm38999	LOC106804052	KIF14	Kif14	LOC101403964	SERPINA12	Serpina12	LOC101407083
C1D	C1d	LOC101407089	KIF15	Kif15	LOC101398319	SERPINA5	Serpina5	LOC101407969
C1GALT1	C1galt1	LOC101408994	KIF16B	Kif16b	LOC101392806	SERPINA6	Serpina6	LOC101405525
C1GALT1C1	C1galt1c1	LOC101397697	KIF17	Kif17	LOC101392608	SERPINA7	Serpina7	LOC101399539
C1QA	C1qa	LOC101398593	KIF18A	Kif18a	LOC101399943	SERPINA9	Serpina9	LOC101407705
C1QB	C1qb	LOC101399117	KIF18B	Kif18b	LOC101398529	SERPINB1	Serpinb1a	LOC101396465
C1QBP	C1qbp	LOC101401943	KIF19	Kif19a	LOC101407422	SERPINB10	Serpinb10	LOC101387526
C1QC	C1qc	LOC101398856	KIF1A	Kif1a	LOC101387453	SERPINB11	Serpinb11	LOC101391219
C1QL1	C1ql1	LOC101398787	KIF1B	Kif1b	LOC101388318	SERPINB12	Serpinb12	LOC101388816
C1QL2	C1ql2	LOC101392523	KIF1C	Kif1c	LOC101400364	SERPINB13	Serpinb13	LOC101389086
C1QL3	C1ql3	LOC101389199	KIF20A	Kif20a	LOC101406167	SERPINB2	Serpinb2	LOC101392336
C1QL4	C1ql4	LOC101403497	KIF20B	Kif20b	LOC101403920	SERPINB3	Serpinb3a	LOC101390463
C1QTNF1	C1qtnf1	LOC101390503	KIF21A	Kif21a	LOC101407827	SERPINB5	Serpinb5	LOC101388555
C1QTNF2	C1qtnf2	LOC101404023	KIF21B	Kif21b	LOC101392032	SERPINB6	Serpinb6a	LOC101395942
C1QTNF3	C1qtnf3	LOC101398660	KIF22	Kif22	LOC101399685	SERPINB7	Serpinb7	LOC101391636
C1QTNF5	C1qtnf5	LOC101387801	KIF23	Kif23	LOC101402064	SERPINB8	Serpinb8	LOC106800617
C1QTNF7	C1qtnf7	LOC101388082	KIF24	Kif24	LOC101389526	SERPINC1	Serpinc1	LOC101398499
C1R	C1ra	LOC101394588	KIF26A	Kif26a	LOC101401246	SERPIND1	Serpind1	LOC101401988
C1RL	C1rl	LOC101387245	KIF26B	Kif26b	LOC101391019	SERPINE1	Serpine1	LOC101389574
C1S	C1s2	LOC101395014	KIF27	Kif27	LOC101396592	SERPINE2	Serpine2	LOC101404811
C1orf100	1700016C15F	LOC101401969	KIF2A	Kif2a	LOC101389184	SERPINE3	Serpine3	LOC101396295
C1orf105	4930558K02F	LOC101395916	KIF2B	Kif2b	LOC101398614	SERPINF1	Serpinf1	LOC101401944
C1orf109	9930104L06F	LOC101396792	KIF2C	Kif2c	LOC101391382	SERPINF2	Serpinf2	LOC101401499
C1orf112	BC055324	LOC101397239	KIF3A	Kif3a	LOC101400400	SERPING1	Serping1	LOC101389735
C1orf115	C130074G19	LOC101397539	KIF3B	Kif3b	LOC101388378	SERPINH1	Serpinh1	LOC101402808
C1orf116	AA986860	LOC101391564	KIF3C	Kif3c	LOC101392468	SERPINI1	Serpini1	LOC101398420
C1orf122	1110065P20F	LOC101398260	KIF4A	Kif4	LOC101387178	SERPINI2	Serpini2	LOC101399376
C1orf127	Gm572	LOC101404445	KIF5A	Kif5a	LOC101396193	SERTAD1	Sertad1	LOC101395202

C1orf131	2810004N23R	LOC101388168	KIF5B	Kif5b	LOC101392528	SERTAD3	Sertad3	LOC101395464
C1orf141	4921539E11R	LOC101389714	KIF5C	Kif5c	LOC101402338	SERTAD4	Sertad4	LOC101406115
C1orf146	1700028K03R	LOC101387772	KIF6	Kif6	LOC101404005	SESN1	Sesn1	LOC101393016
C1orf158	1700012P22R	LOC101406783	KIF7	Kif7	LOC101401153	SESN2	Sesn2	LOC101407494
C1orf159	9430015G10R	LOC101388564	KIF9	Kif9	LOC101407207	SESN3	Sesn3	LOC101393967
C1orf162	I830077J02Ri	LOC106800345	KIFAP3	Kifap3	LOC101405594	SESTD1	Sestd1	LOC101408117
C1orf174	A430005L14R	LOC101399286	KIFBP	Kif1bp	LOC101408731	SET	Set	LOC101399630
C1orf185	4930522H14R	LOC101403608	KIFC1	Kifc1	LOC101396786	SETBP1	Setbp1	LOC101405214
C1orf194	1700013F07R	LOC101405913	KIFC2	Kifc2	LOC101388045	SETD1A	Setd1a	LOC101403091
C1orf198	2310022B05R	LOC101408824	KIFC3	Kifc3	LOC101395419	SETD1B	Setd1b	LOC101406130
C1orf21	1700025G04R	LOC101398670	KIN	Kin	LOC101400240	SETD2	Setd2	LOC101406941
C1orf210	2610528J11R	LOC101404519	KIRREL2	Kirrel2	LOC101393588	SETD3	Setd3	LOC101388259
C1orf216	5730409E04R	LOC101400161	KIRREL3	Kirrel3	LOC101402472	SETD4	Setd4	LOC101388410
C1orf35	2310033P09R	LOC101406359	KISS1R	Kiss1r	LOC101389756	SETD5	Setd5	LOC101394201
C1orf43	4933434E20R	LOC101403602	KIT	Kit	LOC101391446	SETD6	Setd6	LOC101399484
C1orf50	AU022252	LOC101405474	KITLG	Kitl	LOC101401777	SETD7	Setd7	LOC101398006
C1orf52	2410004B18R	LOC101389375	KL	Kl	LOC101408490	SETDB1	Setdb1	LOC101395349
C1orf53	2310009B15R	LOC101406155	KLB	Klb	LOC101393494	SETDB2	Setdb2	LOC101392373
C1orf54	BC028528	LOC101390510	KLC1	Klc1	LOC101397686	SETMAR	Setmar	LOC101393273
C1orf56	Gm128	LOC101397094	KLC2	Klc2	LOC101392563	SEZ6	Sez6	LOC101391670
C1orf74	A130010J15R	LOC101404704	KLC3	Klc3	LOC101395976	SEZ6L	Sez6l	LOC101391921
C1orf87	Gm12695	LOC101403321	KLC4	Klc4	LOC101405853	SEZ6L2	Sez6l2	LOC101393245
C1orf94	CK137956	LOC101407856	KLF1	Klf1	LOC101405839	SF1	Sf1	LOC101396746
C2	C2	LOC101390728	KLF10	Klf10	LOC101390832	SF3A1	Sf3a1	LOC101398108
C2CD2	C2cd2	LOC101401932	KLF11	Klf11	LOC101406330	SF3A2	Sf3a2	LOC101406088
C2CD2L	C2cd2l	LOC101389595	KLF12	Klf12	LOC101408456	SF3A3	Sf3a3	LOC101399196
C2CD3	C2cd3	LOC101396820	KLF13	Klf13	LOC101408398	SF3B1	Sf3b1	LOC101404015
C2CD4D	C2cd4d	LOC101408152	KLF14	Klf14	LOC101403767	SF3B2	Sf3b2	LOC101392027
C2CD5	C2cd5	LOC101387481	KLF15	Klf15	LOC101405218	SF3B3	Sf3b3	LOC101390995
C3	C3	LOC101395200	KLF16	Klf16	LOC101406944	SF3B4	Sf3b4	LOC101387743
C3AR1	C3ar1	LOC101408330	KLF17	Klf17	LOC101405571	SF3B5	Sf3b5	LOC101392320
C4orf17	4930579F01R	LOC101394305	KLF2	Klf2	LOC101404793	SF3B6	Sf3b6	LOC101397283
C4orf19	0610040J01R	LOC101389835	KLF3	Klf3	LOC101391023	SFI1	Sfi1	LOC101388578
C4orf33	D3Ertd751e	LOC101402517	KLF4	Klf4	LOC101406959	SFMBT1	Sfmbt1	LOC101407289
C4orf36	1700016H13R	LOC101402099	KLF5	Klf5	LOC101395827	SFMBT2	Sfmbt2	LOC101401041
C4orf46	4930579G24R	LOC101393266	KLF6	Klf6	LOC101388478	SFN	Sfn	LOC101397832
C4orf47	1700029J07R	LOC101408554	KLF7	Klf7	LOC101401318	SFPQ	Sfpq	LOC101399642
C4orf48	Gm1673	LOC101399824	KLF8	Klf8	LOC101399882	SFR1	Sfr1	LOC101400438
C4orf51	1700011L22R	LOC106800372	KLF9	Klf9	LOC101389063	SFRP1	Sfrp1	LOC101399776
C4orf54	LOC1026343	LOC106801868	KLHDC1	Klhdc1	LOC101400629	SFRP2	Sfrp2	LOC101407300
C5	Hc	LOC101404433	KLHDC10	Klhdc10	LOC101390185	SFRP4	Sfrp4	LOC101400736
C5AR1	C5ar1	LOC101395543	KLHDC2	Klhdc2	LOC101400372	SFRP5	Sfrp5	LOC101394637
C5orf15	9530068E07R	LOC101397293	KLHDC3	Klhdc3	LOC101407484	SFSWAP	Sfswap	LOC101399566
C5orf22	6030458C11R	LOC101395302	KLHDC4	Klhdc4	LOC101389302	SFT2D1	Sft2d1	LOC101390102
C5orf24	B230219D22R	LOC101393938	KLHDC7A	Klhdc7a	LOC101406782	SFT2D2	Sft2d2	LOC101387968
C5orf30	D1Ertd622e	LOC101407916	KLHDC8A	Klhdc8a	LOC101399377	SFT2D3	Sft2d3	LOC101391479
C5orf34	4833420G17R	LOC101393955	KLHDC8B	Klhdc8b	LOC101399606	SFTA2	Sfta2	LOC101403908
C5orf49	1700001L19R	LOC101396847	KLHDC9	Klhdc9	LOC101392766	SFTPA1	Sftpa1	LOC101397425
C5orf51	AW549877	LOC106800651	KLHL1	Klhl1	LOC101406520	SFTPB	Sftpb	LOC101390431
C6	C6	LOC101390390	KLHL10	Klhl10	LOC101403774	SFTPC	Sftpc	LOC101406610
C7	C7	LOC101387962	KLHL11	Klhl11	LOC101403511	SFTPD	Sftpd	LOC101397853
C8A	C8a	LOC101398383	KLHL12	Klhl12	LOC101390270	SFXN1	Sfxn1	LOC101388327
C8B	C8b	LOC101398629	KLHL13	Klhl13	LOC101393270	SFXN2	Sfxn2	LOC101393454
C8G	C8g	LOC101409137	KLHL14	Klhl14	LOC101393435	SFXN3	Sfxn3	LOC101404889

C8orf33	1110038F14F	LOC101400142	KLHL15	Klhl15	LOC101409003	SFXN4	Sfxn4	LOC101392948
C8orf34	A830018L16F	LOC101395086	KLHL17	Klhl17	LOC101391482	SFXN5	Sfxn5	LOC101391189
C8orf37	2610301B20F	LOC101401752	KLHL18	Klhl18	LOC101407656	SGCA	Sgca	LOC101407796
C8orf48	AI429214	LOC101398950	KLHL2	Klhl2	LOC101404905	SGCB	Sgcb	LOC101390614
C8orf58	9930012K11F	LOC101389504	KLHL20	Klhl20	LOC101399721	SGCD	Sgcd	LOC101389603
C8orf74	4930578I06R	LOC101395692	KLHL21	Klhl21	LOC101403217	SGCE	Sgce	LOC101403320
C8orf76	9130401M01	LOC101399567	KLHL22	Klhl22	LOC101398160	SGCG	Sgcg	LOC101402720
C8orf82	C030006K11I	LOC101408005	KLHL23	Klhl23	LOC101401478	SGCZ	Sgcz	LOC101397761
C8orf88	Gm11837	LOC101395418	KLHL24	Klhl24	LOC101398591	SGF29	Sgf29	LOC101398388
C8orf89	4930444P10F	LOC101399322	KLHL26	Klhl26	LOC101408275	SGIP1	Sgip1	LOC101388498
C9	C9	LOC101387873	KLHL28	Klhl28	LOC101403691	SGK1	Sgk1	LOC101406104
CA1	Car1	LOC101395032	KLHL29	Klhl29	LOC101398635	SGK2	Sgk2	LOC101395679
CA10	Car10	LOC101398370	KLHL3	Klhl3	LOC101388027	SGK3	Sgk3	LOC101392293
CA11	Car11	LOC101394256	KLHL30	Klhl30	LOC101406289	SGMS1	Sgms1	LOC101400293
CA12	Car12	LOC101404867	KLHL31	Klhl31	LOC101402776	SGMS2	Sgms2	LOC101404353
CA13	Car13	LOC101395290	KLHL32	Klhl32	LOC101401807	SGO1	Sgol1	LOC101403533
CA14	Car14	LOC101404835	KLHL33	Klhl33	LOC101407126	SGO2	Sgol2a	LOC106800124
CA2	Car2	LOC101394515	KLHL34	Klhl34	LOC101405435	SGPL1	Sgpl1	LOC101401474
CA3	Car3	LOC101394777	KLHL35	Klhl35	LOC101404653	SGPP1	Sgpp1	LOC101399723
CA4	Car4	LOC101395698	KLHL36	Klhl36	LOC101393077	SGPP2	Sgpp2	LOC101401922
CA5A	Car5a	LOC101390262	KLHL38	Klhl38	LOC101398022	SGSH	Sgsh	LOC101400972
CA5B	Car5b	LOC101396475	KLHL4	Klhl4	LOC101393156	SGSM1	Sgsm1	LOC101390915
CA6	Car6	LOC101406884	KLHL40	Klhl40	LOC101393758	SGSM2	Sgsm2	LOC101398610
CA7	Car7	LOC101404561	KLHL41	Klhl41	LOC101402961	SGSM3	Sgsm3	LOC101400547
CA8	Car8	LOC101395519	KLHL42	Klhl42	LOC101395440	SGTA	Sgta	LOC101403187
CA9	Car9	LOC101387612	KLHL5	Klhl5	LOC101392578	SGTB	Sgtb	LOC101393191
CAAP1	Caap1	LOC101399031	KLHL6	Klhl6	LOC101399114	SH2B1	Sh2b1	LOC101405973
CAB39	Cab39	LOC101389975	KLHL7	Klhl7	LOC101401186	SH2B2	Sh2b2	LOC101393598
CAB39L	Cab39l	LOC101392103	KLHL8	Klhl8	LOC101402021	SH2B3	Sh2b3	LOC101399907
CABCOCO1	1700040L02F	LOC101397660	KLHL9	Klhl9	LOC101393788	SH2D1A	Sh2d1a	LOC101395092
CABIN1	Cabin1	LOC101390805	KLK1	Klk1	LOC101393849	SH2D1B	Sh2d1b1	LOC101396435
CABLES1	Cables1	LOC101396509	KLK10	Klk10	LOC101396762	SH2D2A	Sh2d2a	LOC101389299
CABLES2	Cables2	LOC101404896	KLK11	Klk11	LOC101397020	SH2D3C	Sh2d3c	LOC101387965
CABP1	Cabp1	LOC101402016	KLK13	Klk13	LOC101407288	SH2D4A	Sh2d4a	LOC101397376
CABP2	Cabp2	LOC101408494	KLK14	Klk14	LOC101397714	SH2D4B	Sh2d4b	LOC101395943
CABP4	Cabp4	LOC101406830	KLK15	Klk15	LOC101394102	SH2D5	Sh2d5	LOC101392871
CABP5	Cabp5	LOC101389163	KLK4	Klk4	LOC101407013	SH2D6	Sh2d6	LOC101400019
CABP7	Cabp7	LOC101401744	KLK7	Klk7	LOC101395803	SH2D7	Sh2d7	LOC101393432
CABS1	Cabs1	LOC101387499	KLK8	Klk8	LOC101396241	SH3BGRL	Sh3bgrl	LOC101391540
CABYR	Cabyr	LOC101399613	KLK9	Klk9	LOC101397456	SH3BGRL2	Sh3bgrl2	LOC101404420
CACFD1	Cacfd1	LOC101397400	KLKB1	Klkb1	LOC101390825	SH3BGRL3	Sh3bgrl3	LOC101394460
CACHD1	Cachd1	LOC101408043	KLRC2	Klrc2	LOC101390718	SH3BP1	Sh3bp1	LOC101402127
CACNA1A	Cacna1a	LOC101397472	KLRD1	Klrd1	LOC101406069	SH3BP2	Sh3bp2	LOC101397935
CACNA1B	Cacna1b	LOC101395833	KLRG1	Klrg1	LOC101409094	SH3BP4	Sh3bp4	LOC101401216
CACNA1C	Cacna1c	LOC101398455	KLRG2	Klrg2	LOC101394475	SH3BP5	Sh3bp5	LOC101394869
CACNA1D	Cacna1d	LOC101409107	KLRK1	Klrk1	LOC101405800	SH3BP5L	Sh3bp5l	LOC101399972
CACNA1E	Cacna1e	LOC101406280	KMO	Kmo	LOC101400912	SH3D19	Sh3d19	LOC101402570
CACNA1F	Cacna1f	LOC101403244	KMT2A	Kmt2a	LOC101408380	SH3D21	Sh3d21	LOC101400690
CACNA1G	Cacna1g	LOC101389036	KMT2B	Kmt2b	LOC101397876	SH3GL1	Sh3gl1	LOC101393422
CACNA1H	Cacna1h	LOC101397703	KMT2C	Kmt2c	LOC101407163	SH3GL2	Sh3gl2	LOC101406332
CACNA1I	Cacna1i	LOC101391015	KMT2D	Kmt2d	LOC101399991	SH3GL3	Sh3gl3	LOC101392960
CACNA1S	Cacna1s	LOC101402735	KMT2E	Kmt2e	LOC101387172	SH3GLB1	Sh3glb1	LOC101406512
CACNA2D1	Cacna2d1	LOC101387816	KMT5A	Kmt5a	LOC101392797	SH3GLB2	Sh3glb2	LOC101403295
CACNA2D2	Cacna2d2	LOC101392654	KMT5B	Suv420h1	LOC101390087	SH3KBP1	Sh3kbp1	LOC101402202

CACNA2D3	Cacna2d3	LOC101388791	KMT5C	Suv420h2	LOC101390778	SH3PXD2A	Sh3pxd2a	LOC101398862
CACNA2D4	Cacna2d4	LOC101397794	KNCN	Kncn	LOC101402667	SH3PXD2B	Sh3pxd2b	LOC101392537
CACNB1	Cacnb1	LOC101406818	KNDC1	Kndc1	LOC101388274	SH3RF1	Sh3rf1	LOC101408486
CACNB2	Cacnb2	LOC101407145	KNG1	Kng1	LOC101403565	SH3RF2	Sh3rf2	LOC101401277
CACNB3	Cacnb3	LOC101397163	KNL1	Casc5	LOC101392145	SH3RF3	Sh3rf3	LOC101406999
CACNB4	Cacnb4	LOC101398849	KNOP1	Knop1	LOC101390199	SH3TC1	Sh3tc1	LOC101401596
CACNG1	Cacng1	LOC101398284	KNSTRN	Knstrn	LOC101390457	SH3TC2	Sh3tc2	LOC101405333
CACNG2	Cacng2	LOC101396489	KNTC1	Kntc1	LOC101388937	SH3YL1	Sh3yl1	LOC101391514
CACNG3	Cacng3	LOC101407217	KPNA1	Kpna1	LOC101399066	SHANK1	Shank1	LOC101405983
CACNG4	Cacng4	LOC101398530	KPNA3	Kpna3	LOC101393651	SHANK2	Shank2	LOC101400834
CACNG5	Cacng5	LOC101398788	KPNA4	Kpna4	LOC101397125	SHANK3	Shank3	LOC101403171
CACNG6	Cacng6	LOC101405541	KPNA6	Kpna6	LOC101397569	SHARPIN	Sharpin	LOC101404076
CACNG7	Cacng7	LOC101405279	KPNA7	Kpna7	LOC101406599	SHB	Shb	LOC101405848
CACNG8	Cacng8	LOC101402478	KPNB1	Kpnb1	LOC101390164	SHBG	Shbg	LOC101388501
CACTIN	Cactin	LOC101401192	KPTN	Kptn	LOC101395804	SHC1	Shc1	LOC101407436
CACUL1	Cacul1	LOC101395925	KRAS	Kras	LOC101390931	SHC2	Shc2	LOC101394184
CACYBP	Cacybp	LOC101396003	KRBA1	Krba1	LOC101390752	SHC4	Shc4	LOC101401014
CAD	Cad	LOC101405803	KRCC1	Krcc1	LOC101394848	SHCBP1	Shcbp1	LOC101404164
CADM1	Cadm1	LOC101406787	KREMEN1	Kremen1	LOC101391169	SHCBP1L	Shcbp1l	LOC101402688
CADM2	Cadm2	LOC101400208	KREMEN2	Kremen2	LOC101392463	SHE	She	LOC101405440
CADM3	Cadm3	LOC101406175	KRI1	Kri1	LOC101403633	SHF	Shf	LOC101394703
CADM4	Cadm4	LOC101403180	KRIT1	Krit1	LOC101397676	SHISA2	Shisa2	LOC101406474
CADPS	Cadps	LOC101400298	KRR1	Krr1	LOC101390522	SHISA3	Shisa3	LOC101401187
CADPS2	Cadps2	LOC101403796	KRT10	Krt10	LOC101391674	SHISA4	Shisa4	LOC101399681
CAGE1	Cage1	LOC101406554	KRT12	Krt12	LOC101391186	SHISA5	Shisa5	LOC101391532
CALB1	Calb1	LOC101394398	KRT13	Krt13	LOC101407629	SHISA6	Shisa6	LOC101407525
CALB2	Calb2	LOC101389025	KRT14	Krt14	LOC101406817	SHISA7	Shisa7	LOC106800153
CALCA	Calca	LOC101403968	KRT15	Krt15	LOC101407356	SHISA9	Shisa9	LOC101403960
CALCB	Calcb	LOC101404235	KRT16	Krt16	LOC101407087	SHISAL1	1810041L15R	LOC101398705
CALCOCO1	Calcoco1	LOC101392171	KRT17	Krt17	LOC101406321	SHISAL2B	Fam159b	LOC101392337
CALCR	Calcr	LOC101401148	KRT18	Krt18	LOC101405160	SHKBP1	Shkbp1	LOC101395986
CALCRL	Calcrl	LOC101393541	KRT19	Krt19	LOC101407086	SHLD2	Fam35a	LOC101406463
CALD1	Cald1	LOC101398688	KRT20	Krt20	LOC101390928	SHMT1	Shmt1	LOC101393901
CALHM1	Calhm1	LOC106799950	KRT222	Krt222	LOC101392971	SHMT2	Shmt2	LOC101390744
CALHM2	Calhm2	LOC101397919	KRT23	Krt23	LOC101390676	SHOC1	AI481877	LOC101391475
CALHM5	Fam26e	LOC101404938	KRT24	Krt24	LOC101392706	SHOC2	Shoc2	LOC101407773
CALHM6	Fam26f	LOC101400755	KRT25	Krt25	LOC101392453	SHOX2	Shox2	LOC101392050
CALM1	Calm1	LOC101397085	KRT26	Krt26	LOC101392191	SHPK	Shpk	LOC101391001
CALM2	Calm2	LOC101395264	KRT27	Krt27	LOC101391939	SHPRH	Shprh	LOC101391030
CALM3	Calm3	LOC101393756	KRT28	Krt28	LOC101391429	SHQ1	Shq1	LOC101387689
CALML3	Calml3	LOC101403482	KRT31	Krt31	LOC101407890	SHROOM1	Shroom1	LOC101388715
CALML4	Calml4	LOC101399443	KRT33A	Krt33a	LOC101408145	SHROOM2	Shroom2	LOC101404245
CALML5	Calm4	LOC101403748	KRT33B	Krt33b	LOC106802501	SHROOM3	Shroom3	LOC101400259
CALN1	Caln1	LOC101393750	KRT34	Krt34	LOC106802500	SHROOM4	Shroom4	LOC101400187
CALR	Calr	LOC101406765	KRT36	Krt36	LOC101407888	SHTN1	Shtn1	LOC101392615
CALR3	Calr3	LOC101399884	KRT39	Krt39	LOC101390423	SI	Sis	LOC101400334
CALU	Calu	LOC101395452	KRT4	Krt4	LOC101403923	SIAE	Siae	LOC101392983
CALY	Caly	LOC101394172	KRT40	Krt40	LOC101388862	SIAH2	Siah2	LOC106801955
CAMK1	Camk1	LOC101391980	KRT5	Krt5	LOC101401056	SIDT1	Sidt1	LOC101402725
CAMK1D	Camk1d	LOC101395750	KRT6B	Gm5414	LOC106801625	SIDT2	Sidt2	LOC101404552
CAMK1G	Camk1g	LOC101403042	KRT7	Krt7	LOC101399211	SIGIRR	Sigirr	LOC101389318
CAMK2A	Camk2a	LOC101400247	KRT73	Krt73	LOC101390234	SIGLEC1	Siglec1	LOC101407884
CAMK2B	Camk2b	LOC101401086	KRT75	Krt75	LOC101400788	SIGLEC15	Siglec15	LOC101406002
CAMK2D	Camk2d	LOC101393966	KRT78	Krt78	LOC101404891	SIGLECL1	Gm2511	LOC106804050

CAMK2G	Camk2g	LOC101409148	KRT79	Krt79	LOC101404193	SIGMAR1	Sigmar1	LOC101406891
CAMK2N1	Camk2n1	LOC101391156	KRT8	Krt8	LOC101404453	SIK1	Sik1	LOC101401224
CAMK2N2	Camk2n2	LOC101395835	KRT80	Krt80	LOC101398770	SIK2	Sik2	LOC101396714
CAMK4	Camk4	LOC101404510	KRT81	Krt81	LOC101389457	SIK3	Sik3	LOC101387296
CAMKK1	Camkk1	LOC101388684	KRT82	Krt82	LOC106801624	SIKE1	Sike1	LOC101401981
CAMKK2	Camkk2	LOC101402704	KRT84	Krt84	LOC101400517	SIL1	Sil1	LOC101401886
CAMKMT	Camkmt	LOC101399930	KRT86	Krt86	LOC101399476	SIM1	Sim1	LOC101405472
CAMKV	Camkv	LOC101407657	KRT9	Krt9	LOC101407358	SIM2	Sim2	LOC101399299
CAMLG	Caml	LOC101394436	KRTAP3-1	Krtap3-1	LOC101389387	SIMC1	Simc1	LOC101391835
CAMP	Camp	LOC101389167	KRTAP3-2	Krtap3-2	LOC101389905	SIN3A	Sin3a	LOC101398479
CAMSAP1	Camsap1	LOC101388917	KRTAP3-3	Krtap3-3	LOC101390163	SIN3B	Sin3b	LOC101398339
CAMSAP2	Camsap2	LOC101403256	KRTAP7-1	Krtap7-1	LOC101401838	SINHCAF	Fam60a	LOC101405295
CAMSAP3	Camsap3	LOC101392244	KRTAP8-1	Krtap8-1	LOC106801667	SIPA1	Sipa1	LOC101407366
CAMTA1	Camta1	LOC101402086	KRTCAP2	Krtcap2	LOC101390429	SIPA1L1	Sipa1l1	LOC101395141
CAMTA2	Camta2	LOC101399312	KRTCAP3	Krtcap3	LOC101401966	SIPA1L2	Sipa1l2	LOC101389378
CAND1	Cand1	LOC101388330	KRTDAP	Krtdap	LOC101401356	SIPA1L3	Sipa1l3	LOC101405989
CAND2	Cand2	LOC101387108	KSR1	Ksr1	LOC101391705	SIRPA	Sirpa	LOC101405346
CANT1	Cant1	LOC101390251	KSR2	Ksr2	LOC101387458	SIRT1	Sirt1	LOC101392681
CANX	Canx	LOC101393456	KTI12	Kti12	LOC101406671	SIRT2	Sirt2	LOC101406605
CAP1	Cap1	LOC101406118	KTN1	Ktn1	LOC101387797	SIRT3	Sirt3	LOC101404674
CAP2	Cap2	LOC101401331	KXD1	Kxd1	LOC101387359	SIRT4	Sirt4	LOC101405163
CAPG	Capg	LOC101387827	KY	Ky	LOC101387759	SIRT5	Sirt5	LOC101404725
CAPN1	Capn1	LOC101402729	KYAT1	Kyat1	LOC101401389	SIRT6	Sirt6	LOC101395116
CAPN10	Capn10	LOC101409058	KYAT3	Kyat3	LOC101403812	SIRT7	Sirt7	LOC101399488
CAPN11	Capn11	LOC101396432	KYNU	Kynu	LOC101405398	SIT1	Sit1	LOC101393954
CAPN12	Capn12	LOC101404513	L1CAM	L1cam	LOC101388815	SIVA1	Siva1	LOC101402715
CAPN13	Capn13	LOC101395370	L2HGDH	L2hgdh	LOC101399146	SIX1	Six1	LOC101395235
CAPN15	Capn15	LOC101404320	L3HYPDH	L3hypdh	LOC101393126	SIX2	Six2	LOC101399410
CAPN2	Capn2	LOC101404131	L3MBTL1	L3mbtl1	LOC101396541	SIX3	Six3	LOC101399674
CAPN3	Capn3	LOC101402410	L3MBTL2	L3mbtl2	LOC101403434	SIX4	Six4	LOC101395496
CAPN5	Capn5	LOC101407081	L3MBTL3	L3mbtl3	LOC101399182	SIX5	Six5	LOC101400120
CAPN6	Capn6	LOC101397715	L3MBTL4	L3mbtl4	LOC101400408	SIX6	Six6	LOC101394982
CAPN7	Capn7	LOC101396672	LACC1	Lacc1	LOC101405175	SKA1	Ska1	LOC101394967
CAPN8	Capn8	LOC101403870	LACTB	Lactb	LOC101403631	SKA2	Ska2	LOC101394488
CAPN9	Capn9	LOC101408221	LACTB2	Lactb2	LOC101396832	SKA3	Ska3	LOC101405349
CAPNS1	Capns1	LOC101392128	LACTBL1	Lactbl1	LOC101399641	SKAP1	Skap1	LOC101395171
CAPNS2	Capns2	LOC101407344	LAD1	Lad1	LOC101391268	SKAP2	Skap2	LOC101395020
CAPRIN1	Caprin1	LOC101403954	LAG3	Lag3	LOC101387748	SKI	Ski	LOC101395577
CAPRIN2	Caprin2	LOC101405562	LAGE3	Lage3	LOC101398743	SKIL	Skil	LOC101393704
CAPS2	Caps2	LOC101405367	LAIR1	Lair1	LOC101406929	SKIV2L	Skiv2l	LOC101389965
CAPSL	Capsl	LOC101401900	LALBA	Lalba	LOC101394821	SKOR1	Skor1	LOC101398736
CAPZA1	Capza1	LOC101388895	LAMA1	Lama1	LOC101399870	SKP1	Skp1a	LOC101396501
CAPZA2	Capza2	LOC101388281	LAMA2	Lama2	LOC101398483	SKP2	Skp2	LOC101404269
CAPZA3	Capza3	LOC101403514	LAMA3	Lama3	LOC101400308	SLA	Sla	LOC101389984
CAPZB	Capzb	LOC101387535	LAMA4	Lama4	LOC101402249	SLA2	Sla2	LOC101407781
CARD10	Card10	LOC101400907	LAMA5	Lama5	LOC101405165	SLAIN1	Slain1	LOC101390127
CARD11	Card11	LOC101394603	LAMB1	Lamb1	LOC101402999	SLAIN2	Slain2	LOC101407454
CARD14	Card14	LOC101392183	LAMB2	Lamb2	LOC101398823	SLAMF1	Slamf1	LOC101394271
CARD19	Card19	LOC101398080	LAMB3	Lamb3	LOC101403298	SLAMF6	Slamf6	LOC101403995
CARD6	Card6	LOC101389680	LAMC1	Lamc1	LOC101402428	SLAMF7	Slamf7	LOC101404531
CARD9	Card9	LOC101403905	LAMC2	Lamc2	LOC101402169	SLAMF8	Slamf8	LOC101408959
CARF	Carf	LOC101393886	LAMC3	Lamc3	LOC101405321	SLAMF9	Slamf9	LOC101388043
CARHSP1	Carhsp1	LOC101388097	LAMP1	Lamp1	LOC101390213	SLBP	Slbp	LOC101399572
CARM1	Carm1	LOC101404155	LAMP2	Lamp2	LOC101398804	SLC10A1	Slc10a1	LOC101392262

CARNMT1	Carnmt1	LOC101408855	LAMP3	Lamp3	LOC101388058	SLC10A2	Slc10a2	LOC101406873
CARNS1	Carns1	LOC101407099	LAMP5	Lamp5	LOC101398034	SLC10A3	Slc10a3	LOC101395997
CARS2	Cars2	LOC101401566	LAMTOR1	Lamtor1	LOC101388685	SLC10A4	Slc10a4	LOC101407730
CARTPT	Cartpt	LOC101395725	LAMTOR2	Lamtor2	LOC101401075	SLC10A5	Slc10a5	LOC101388320
CASC1	Casc1	LOC101390426	LAMTOR3	Lamtor3	LOC101395338	SLC10A6	Slc10a6	LOC101401328
CASC3	Casc3	LOC101396824	LAMTOR4	Lamtor4	LOC101390957	SLC10A7	Slc10a7	LOC101397551
CASC4	Casc4	LOC101390379	LAMTOR5	Lamtor5	LOC101395553	SLC11A1	Slc11a1	LOC101408120
CASD1	Casd1	LOC101403061	LANCL1	Lancl1	LOC101396535	SLC11A2	Slc11a2	LOC101390743
CASK	Cask	LOC101403857	LANCL2	Lancl2	LOC101399764	SLC12A1	Slc12a1	LOC101400046
CASKIN1	Caskin1	LOC101395271	LANCL3	Lancl3	LOC101389815	SLC12A2	Slc12a2	LOC101389757
CASKIN2	Caskin2	LOC101394998	LAP3	Lap3	LOC101391081	SLC12A3	Slc12a3	LOC101391247
CASP1	Casp1	LOC101391162	LAPTM4A	Laptm4a	LOC101400200	SLC12A4	Slc12a4	LOC101396377
CASP14	Casp14	LOC101398844	LAPTM4B	Laptm4b	LOC101404102	SLC12A5	Slc12a5	LOC101401747
CASP2	Casp2	LOC101400877	LAPTM5	Laptm5	LOC101391825	SLC12A6	Slc12a6	LOC101401638
CASP3	Casp3	LOC101405759	LARP1	Larp1	LOC101391238	SLC12A7	Slc12a7	LOC101395282
CASP6	Casp6	LOC101401047	LARP1B	Larp1b	LOC101403754	SLC12A8	Slc12a8	LOC101403744
CASP7	Casp7	LOC101388667	LARP4	Larp4	LOC101387891	SLC12A9	Slc12a9	LOC101398081
CASP8	Casp8	LOC101389874	LARP4B	Larp4b	LOC101390647	SLC13A1	Slc13a1	LOC101403263
CASP8AP2	Casp8ap2	LOC101398578	LARP6	Larp6	LOC101404338	SLC13A2	Slc13a2	LOC101398121
CASP9	Casp9	LOC101398346	LARP7	Larp7	LOC101396530	SLC13A3	Slc13a3	LOC101399908
CASQ1	Casq1	LOC101389856	LARS2	Lars2	LOC101400396	SLC13A4	Slc13a4	LOC101393977
CASQ2	Casq2	LOC101399780	LAS1L	Las1l	LOC101394342	SLC13A5	Slc13a5	LOC101406809
CASR	Casr	LOC101397437	LASP1	Lasp1	LOC101408055	SLC14A1	Slc14a1	LOC101405734
CASS4	Cass4	LOC101387213	LAT	Lat	LOC101397874	SLC14A2	Slc14a2	LOC101405476
CAST	Cast	LOC101390285	LAT2	Lat2	LOC101399616	SLC15A1	Slc15a1	LOC101399190
CASZ1	Casz1	LOC101390049	LATS1	Lats1	LOC101387851	SLC15A2	Slc15a2	LOC101398445
CAT	Cat	LOC101404742	LATS2	Lats2	LOC101400985	SLC15A3	Slc15a3	LOC101388354
CATIP	Catip	LOC101398861	LAX1	Lax1	LOC101404007	SLC15A4	Slc15a4	LOC101405164
CATSPER1	Catsper1	LOC101406333	LAYN	Layn	LOC101396974	SLC15A5	Slc15a5	LOC101404575
CATSPER3	Catsper3	LOC101393176	LBH	Lbh	LOC101393843	SLC16A1	Slc16a1	LOC101408348
CATSPER4	Catsper4	LOC101393964	LBP	Lbp	LOC101401674	SLC16A10	Slc16a10	LOC101399101
CATSPERD	Catsperd	LOC101397025	LBR	Lbr	LOC101406675	SLC16A11	Slc16a11	LOC101390924
CAV1	Cav1	LOC101388973	LBX1	Lbx1	LOC101405410	SLC16A12	Slc16a12	LOC101403662
CAV2	Cav2	LOC101389747	LBX2	Lbx2	LOC101401081	SLC16A13	Slc16a13	LOC101391181
CAV3	Cav3	LOC101402495	LCA5	Lca5	LOC101404157	SLC16A14	Slc16a14	LOC101389203
CAVIN1	Ptrf	LOC101397857	LCA5L	Lca5l	LOC101394643	SLC16A2	Slc16a2	LOC101396338
CAVIN2	Sdpr	LOC101401135	LCAT	Lcat	LOC101397173	SLC16A3	Slc16a3	LOC101402713
CAVIN3	Prkcdbp	LOC101396050	LCE6A	Lce6a	LOC101407438	SLC16A4	Slc16a4	LOC101395811
CAVIN4	Murc	LOC101394890	LCK	Lck	LOC101400070	SLC16A5	Slc16a5	LOC106802215
CBARP	Cbarp	LOC101388456	LCLAT1	Lclat1	LOC101393089	SLC16A6	Slc16a6	LOC101401599
CBFA2T2	Cbfa2t2	LOC101406099	LCMT1	Lcmt1	LOC101395796	SLC16A7	Slc16a7	LOC101404095
CBFA2T3	Cbfa2t3	LOC101407440	LCMT2	Lcmt2	LOC101406363	SLC16A8	Slc16a8	LOC101391349
CBFB	Cbfb	LOC101406903	LCN1	Lcn3	LOC101389960	SLC16A9	Slc16a9	LOC101400072
CBL	Cbl	LOC101407331	LCN10	Lcn10	LOC106800740	SLC17A1	Slc17a1	LOC101399744
CBLB	Cblb	LOC101398299	LCN2	Lcn2	LOC101393288	SLC17A2	Slc17a2	LOC101390577
CBLC	Cblc	LOC101406079	LCN6	Lcn6	LOC101390219	SLC17A3	Slc17a3	LOC101391175
CBLL1	Cbll1	LOC101403967	LCN8	Lcn8	LOC101390472	SLC17A4	Slc17a4	LOC101400001
CBLN1	Cbln1	LOC101401675	LCOR	Lcor	LOC101388069	SLC17A5	Slc17a5	LOC101399963
CBLN2	Cbln2	LOC101389087	LCORL	Lcorl	LOC101392544	SLC17A6	Slc17a6	LOC101396053
CBLN3	Cbln3	LOC101390290	LCP1	Lcp1	LOC101387475	SLC17A7	Slc17a7	LOC101403446
CBLN4	Cbln4	LOC101408989	LCP2	Lcp2	LOC101396801	SLC17A8	Slc17a8	LOC101398309
CBR1	Cbr1	LOC101388672	LCT	Lct	LOC101408015	SLC17A9	Slc17a9	LOC101396283
CBR3	Cbr3	LOC101388935	LCTL	Lctl	LOC101396686	SLC18A1	Slc18a1	LOC101389254
CBR4	Cbr4	LOC101408224	LDAH	Ldah	LOC101398899	SLC18A2	Slc18a2	LOC101394058

CBS	Cbs	LOC101403230	LDB1	Ldb1	LOC101388242	SLC18A3	Slc18a3	LOC101406728
CBX1	Cbx1	LOC101394487	LDB2	Ldb2	LOC101390248	SLC18B1	Slc18b1	LOC101408616
CBX2	Cbx2	LOC101400180	LDB3	Ldb3	LOC101391251	SLC19A1	Slc19a1	LOC101405869
CBX3	Cbx3	LOC101395539	LDHA	Ldha	LOC101388017	SLC19A2	Slc19a2	LOC101407489
CBX4	Cbx4	LOC101391420	LDHB	Ldhb	LOC101407972	SLC19A3	Slc19a3	LOC101408807
CBX5	Cbx5	LOC101395242	LDHC	Ldhc	LOC101389158	SLC1A1	Slc1a1	LOC101397118
CBX6	Cbx6	LOC101396045	LDHD	Ldhd	LOC101407188	SLC1A2	Slc1a2	LOC101408752
CBX7	Cbx7	LOC101396310	LDLR	Ldlr	LOC101404418	SLC1A3	Slc1a3	LOC101405583
CBX8	Cbx8	LOC101391179	LDLRAD1	Ldlrad1	LOC101392824	SLC1A4	Slc1a4	LOC101387570
CBY1	Cby1	LOC101394168	LDLRAD3	Ldlrad3	LOC101392900	SLC1A5	Slc1a5	LOC101404330
CBY2	Spert	LOC101408568	LDLRAD4	Ldlrad4	LOC101394608	SLC1A6	Slc1a6	LOC101393524
CC2D1A	Cc2d1a	LOC101388636	LDLRAP1	Ldlrap1	LOC101389592	SLC1A7	Slc1a7	LOC101389232
CC2D1B	Cc2d1b	LOC101408238	LDOC1	Ldoc1	LOC101389186	SLC20A1	Slc20a1	LOC101389297
CC2D2A	Cc2d2a	LOC101388681	LEAP2	Leap2	LOC101398724	SLC20A2	Slc20a2	LOC101394020
CC2D2B	Cc2d2b	LOC101407771	LECT2	Lect2	LOC101390368	SLC22A1	Slc22a1	LOC101393417
CCAR1	Ccar1	LOC101388154	LEF1	Lef1	LOC101403301	SLC22A12	Slc22a12	LOC101394498
CCAR2	Ccar2	LOC101403109	LEFTY2	Lefty2	LOC101407450	SLC22A13	Slc22a13	LOC101403534
CCBE1	Ccbe1	LOC101408364	LEKR1	Lekr1	LOC101390536	SLC22A14	Slc22a14	LOC101399772
CCDC102A	Ccdc102a	LOC101397771	LEMD1	Lemd1	LOC101398934	SLC22A15	Slc22a15	LOC101399259
CCDC103	Ccdc103	LOC101397681	LEMD2	Lemd2	LOC101408726	SLC22A16	Slc22a16	LOC101399184
CCDC105	Ccdc105	LOC101393783	LEMD3	Lemd3	LOC101409160	SLC22A17	Slc22a17	LOC101403724
CCDC106	Ccdc106	LOC101395447	LENEP	Lenep	LOC101408842	SLC22A18	Slc22a18	LOC101388538
CCDC107	Ccdc107	LOC101394200	LENG1	Leng1	LOC101408067	SLC22A2	Slc22a2	LOC101387425
CCDC112	Ccdc112	LOC101398994	LENG8	Leng8	LOC101387935	SLC22A23	Slc22a23	LOC101394231
CCDC113	Ccdc113	LOC101397770	LEO1	Leo1	LOC101387517	SLC22A3	Slc22a3	LOC101387944
CCDC114	Ccdc114	LOC101398641	LEP	Lep	LOC101398144	SLC22A4	Slc22a4	LOC101402659
CCDC115	Ccdc115	LOC101406112	LEPR	Lepr	LOC101387308	SLC22A5	Slc22a5	LOC101401976
CCDC116	Ccdc116	LOC101389672	LEPROT	Leprot	LOC106799918	SLC22A6	Slc22a6	LOC101405273
CCDC117	Ccdc117	LOC101408557	LEPROTL1	Leprotl1	LOC101402093	SLC22A7	Slc22a7	LOC101403127
CCDC12	Ccdc12	LOC101406683	LETM1	Letm1	LOC101395162	SLC22A8	Slc22a8	LOC101399236
CCDC120	Ccdc120	LOC101406569	LETM2	Letm2	LOC101393047	SLC23A1	Slc23a1	LOC101400032
CCDC122	Ccdc122	LOC101404906	LETMD1	Letmd1	LOC101391491	SLC23A2	Slc23a2	LOC101403072
CCDC124	Ccdc124	LOC101391902	LEXM	Lexm	LOC101404230	SLC23A3	Slc23a3	LOC101391487
CCDC125	Ccdc125	LOC101397989	LFNG	Lfng	LOC101405457	SLC24A1	Slc24a1	LOC101393601
CCDC126	Ccdc126	LOC101399687	LGALS1	Lgals1	LOC101402643	SLC24A2	Slc24a2	LOC101403692
CCDC127	Ccdc127	LOC101408594	LGALS12	Lgals12	LOC101406668	SLC24A3	Slc24a3	LOC101408138
CCDC13	Ccdc13	LOC101394259	LGALS2	Lgals2	LOC101401436	SLC24A4	Slc24a4	LOC101400534
CCDC130	Ccdc130	LOC101397730	LGALS3	Lgals3	LOC101409145	SLC24A5	Slc24a5	LOC101399356
CCDC134	Ccdc134	LOC101407801	LGALS3BP	Lgals3bp	LOC101389993	SLC25A1	Slc25a1	LOC101397130
CCDC136	Ccdc136	LOC101399167	LGALS4	Lgals4	LOC101405299	SLC25A10	Slc25a10	LOC101397076
CCDC137	Ccdc137	LOC101403067	LGALS7	Lgals7	LOC101404772	SLC25A11	Slc25a11	LOC101397427
CCDC138	Ccdc138	LOC101394241	LGALS8	Lgals8	LOC101394831	SLC25A12	Slc25a12	LOC101396632
CCDC14	Ccdc14	LOC101401655	LGALS9	Lgals9	LOC101391972	SLC25A13	Slc25a13	LOC101399742
CCDC141	Ccdc141	LOC101407859	LGALSL	Lgalsl	LOC101388689	SLC25A14	Slc25a14	LOC101388515
CCDC142	Ccdc142	LOC101399760	LGI1	Lgi1	LOC101400076	SLC25A16	Slc25a16	LOC101388829
CCDC146	Ccdc146	LOC101390605	LGI2	Lgi2	LOC101396813	SLC25A17	Slc25a17	LOC101401521
CCDC148	Ccdc148	LOC101398682	LGI3	Lgi3	LOC101407030	SLC25A18	Slc25a18	LOC106803178
CCDC149	Ccdc149	LOC101407699	LGI4	Lgi4	LOC101390526	SLC25A19	Slc25a19	LOC101393899
CCDC15	Ccdc15	LOC101396231	LGMN	Lgmn	LOC101401245	SLC25A2	Slc25a2	LOC101387504
CCDC150	Ccdc150	LOC101408805	LGR4	Lgr4	LOC101405190	SLC25A20	Slc25a20	LOC101394947
CCDC151	Ccdc151	LOC101395647	LGR5	Lgr5	LOC101397254	SLC25A21	Slc25a21	LOC101408668
CCDC152	Ccdc152	LOC101391638	LGR6	Lgr6	LOC101397871	SLC25A22	Slc25a22	LOC101393096
CCDC153	Ccdc153	LOC101407603	LHB	Lhb	LOC101399338	SLC25A23	Slc25a23	LOC101404929
CCDC154	Ccdc154	LOC101397868	LHCGR	Lhcgr	LOC101391434	SLC25A24	Slc25a24	LOC101387184

CCDC155	Ccdc155	LOC101402651	LHFPL1	Lhfpl1	LOC101399861	SLC25A25	Slc25a25	LOC101392253
CCDC157	Ccdc157	LOC101390145	LHFPL2	Lhfpl2	LOC101408695	SLC25A26	Slc25a26	LOC101404604
CCDC158	Ccdc158	LOC101407613	LHFPL3	Lhfpl3	LOC101387838	SLC25A27	Slc25a27	LOC101390893
CCDC159	Ccdc159	LOC101393861	LHFPL4	Lhfpl4	LOC101393781	SLC25A28	Slc25a28	LOC101387552
CCDC160	Ccdc160	LOC101404389	LHFPL5	Lhfpl5	LOC101407403	SLC25A29	Slc25a29	LOC101388176
CCDC166	Ccdc166	LOC101405652	LHFPL6	Lhfp	LOC101393520	SLC25A3	Slc25a3	LOC101393165
CCDC167	Ccdc167	LOC101400060	LHPP	Lhpp	LOC101392391	SLC25A30	Slc25a30	LOC101408051
CCDC169	Ccdc169	LOC101408749	LHX1	Lhx1	LOC101397689	SLC25A31	Slc25a31	LOC101388236
CCDC17	Ccdc17	LOC101397630	LHX2	Lhx2	LOC101398585	SLC25A32	Slc25a32	LOC101392441
CCDC170	Ccdc170	LOC101390448	LHX3	Lhx3	LOC101404620	SLC25A33	Slc25a33	LOC101407942
CCDC171	Ccdc171	LOC101407718	LHX4	Lhx4	LOC101408466	SLC25A34	Slc25a34	LOC101399896
CCDC172	Ccdc172	LOC101391833	LHX5	Lhx5	LOC101390747	SLC25A35	Slc25a35	LOC101392548
CCDC173	Ccdc173	LOC101402178	LHX6	Lhx6	LOC101407481	SLC25A36	Slc25a36	LOC101400211
CCDC174	Ccdc174	LOC101393518	LHX8	Lhx8	LOC101399219	SLC25A37	Slc25a37	LOC101408829
CCDC175	Ccdc175	LOC101408022	LHX9	Lhx9	LOC101405901	SLC25A38	Slc25a38	LOC101408688
CCDC177	Ccdc177	LOC101391312	LIAS	Lias	LOC101394344	SLC25A39	Slc25a39	LOC101393988
CCDC178	Ccdc178	LOC101403641	LIF	Lif	LOC101398955	SLC25A4	Slc25a4	LOC101406793
CCDC18	Ccdc18	LOC101405378	LIFR	Lifr	LOC101408620	SLC25A40	Slc25a40	LOC101391841
CCDC180	Ccdc180	LOC101395744	LIG1	Lig1	LOC101389413	SLC25A41	Slc25a41	LOC101405200
CCDC181	Ccdc181	LOC101407760	LIG3	Lig3	LOC101402755	SLC25A42	Slc25a42	LOC101404791
CCDC182	Ccdc182	LOC101393233	LILRA4	Lilra6	LOC101405632	SLC25A43	Slc25a43	LOC101396154
CCDC183	Ccdc183	LOC101407323	LILRA6	Pira1	LOC106800106	SLC25A44	Slc25a44	LOC101403081
CCDC184	Ccdc184	LOC101393048	LILRB3	Pirb	LOC101404590	SLC25A45	Slc25a45	LOC101404224
CCDC185	Ccdc185	LOC101396237	LIM2	Lim2	LOC101402071	SLC25A46	Slc25a46	LOC101405030
CCDC186	Ccdc186	LOC101389878	LIMA1	Lima1	LOC101387633	SLC25A47	Slc25a47	LOC101390161
CCDC187	Ccdc187	LOC101388145	LIMCH1	Limch1	LOC101398818	SLC25A48	Slc25a48	LOC101391124
CCDC188	Ccdc188	LOC106803867	LIMD1	Limd1	LOC101400654	SLC25A5	Slc25a5	LOC101396417
CCDC189	Ccdc189	LOC101400553	LIMD2	Limd2	LOC101389806	SLC26A10	Slc26a10	LOC101398864
CCDC190	Ccdc190	LOC101397828	LIME1	Lime1	LOC101402019	SLC26A11	Slc26a11	LOC101392446
CCDC191	Ccdc191	LOC101402463	LIMK1	Limk1	LOC106803803	SLC26A3	Slc26a3	LOC101403706
CCDC194	C430049E01R	LOC106800642	LIMK2	Limk2	LOC101391408	SLC26A4	Slc26a4	LOC101404234
CCDC196	Gm6657	LOC101405404	LIMS1	Lims1	LOC101393740	SLC26A5	Slc26a5	LOC101388968
CCDC198	1700011H14R	LOC101390050	LIMS2	Lims2	LOC101401391	SLC26A7	Slc26a7	LOC101396202
CCDC22	Ccdc22	LOC101402812	LIN28A	Lin28a	LOC101395579	SLC26A8	Slc26a8	LOC101406879
CCDC24	Ccdc24	LOC101405304	LIN28B	Lin28b	LOC101407133	SLC26A9	Slc26a9	LOC101396790
CCDC25	Ccdc25	LOC101401501	LIN37	Lin37	LOC101392649	SLC27A1	Slc27a1	LOC101394532
CCDC27	Ccdc27	LOC101399722	LIN52	Lin52	LOC101404446	SLC27A2	Slc27a2	LOC101403548
CCDC28A	Ccdc28a	LOC101389583	LIN54	Lin54	LOC101394993	SLC27A3	Slc27a3	LOC101389224
CCDC28B	Ccdc28b	LOC101398259	LIN7A	Lin7a	LOC101396397	SLC27A4	Slc27a4	LOC101395743
CCDC3	Ccdc3	LOC101401659	LIN7B	Lin7b	LOC101401005	SLC27A5	Slc27a5	LOC101406070
CCDC30	Ccdc30	LOC101407671	LIN7C	Lin7c	LOC101398219	SLC27A6	Slc27a6	LOC101408175
CCDC32	Ccdc32	LOC101391630	LIN9	Lin9	LOC101409021	SLC28A1	Slc28a1	LOC101402711
CCDC33	Ccdc33	LOC101391896	LINGO3	Lingo3	LOC101405641	SLC28A3	Slc28a3	LOC101398395
CCDC34	Ccdc34	LOC101404922	LINGO4	Lingo4	LOC101407435	SLC29A1	Slc29a1	LOC101396701
CCDC36	Ccdc36	LOC101407914	LINS1	Lins1	LOC101390996	SLC29A2	Slc29a2	LOC101395358
CCDC38	Ccdc38	LOC101408767	LIPA	Lipa	LOC101405192	SLC29A3	Slc29a3	LOC101400952
CCDC39	Ccdc39	LOC101402168	LIPC	Lipc	LOC101395897	SLC29A4	Slc29a4	LOC101392738
CCDC40	Ccdc40	LOC101400451	LIPE	Lipe	LOC101402395	SLC2A1	Slc2a1	LOC101406867
CCDC42	Ccdc42	LOC101389380	LIPF	Lipf	LOC101397877	SLC2A10	Slc2a10	LOC101399392
CCDC43	Ccdc43	LOC101395686	LIPG	Lipg	LOC101391979	SLC2A12	Slc2a12	LOC101405841
CCDC47	Ccdc47	LOC101391178	LIPH	Liph	LOC101408464	SLC2A13	Slc2a13	LOC101400301
CCDC50	Ccdc50	LOC101396787	LIPI	Lipi	LOC101393679	SLC2A2	Slc2a2	LOC101392156
CCDC51	Ccdc51	LOC101390281	LIPK	Lipk	LOC101397616	SLC2A3	Slc2a3	LOC101390856
CCDC54	Ccdc54	LOC101397784	LIPM	Lipm	LOC101397111	SLC2A4	Slc2a4	LOC101408918

CCDC57	Ccdc57	LOC101403415	LIPN	Lipn	LOC101397366	SLC2A5	Slc2a5	LOC101407147
CCDC58	Ccdc58	LOC101396916	LIPT2	Lipt2	LOC101398035	SLC2A6	Slc2a6	LOC101397823
CCDC59	Ccdc59	LOC101398385	LITAF	Litaf	LOC101406834	SLC2A7	Slc2a7	LOC101402593
CCDC6	Ccdc6	LOC101399809	LIX1	Lix1	LOC101388893	SLC2A8	Slc2a8	LOC101407852
CCDC60	Ccdc60	LOC101396282	LIX1L	Lix1l	LOC101400489	SLC2A9	Slc2a9	LOC101408401
CCDC61	Ccdc61	LOC101402396	LKAAEAR1	Lkaaear1	LOC101401229	SLC30A1	Slc30a1	LOC101387371
CCDC62	Ccdc62	LOC101402278	LLCFC1	1700034O15I	LOC101399998	SLC30A10	Slc30a10	LOC101396054
CCDC63	Ccdc63	LOC101400705	LLGL1	Llgl1	LOC101392628	SLC30A2	Slc30a2	LOC101391909
CCDC65	Ccdc65	LOC101398427	LLGL2	Llgl2	LOC101395510	SLC30A3	Slc30a3	LOC101405544
CCDC66	Ccdc66	LOC101390022	LLPH	Llph	LOC101387132	SLC30A4	Slc30a4	LOC101395993
CCDC68	Ccdc68	LOC101399272	LMAN1	Lman1	LOC101407222	SLC30A5	Slc30a5	LOC101396697
CCDC69	Ccdc69	LOC101397760	LMAN1L	Lman1l	LOC101394613	SLC30A6	Slc30a6	LOC101390011
CCDC70	Ccdc70	LOC101397079	LMAN2	Lman2	LOC101402438	SLC30A7	Slc30a7	LOC101391539
CCDC73	Ccdc73	LOC101407284	LMAN2L	Lman2l	LOC101399503	SLC30A8	Slc30a8	LOC101405510
CCDC77	Ccdc77	LOC101394675	LMBR1	Lmbr1	LOC101405607	SLC30A9	Slc30a9	LOC101400650
CCDC78	Ccdc78	LOC101399154	LMBR1L	Lmbr1l	LOC101401406	SLC31A1	Slc31a1	LOC101394801
CCDC80	Ccdc80	LOC101391860	LMBRD1	Lmbrd1	LOC101396431	SLC31A2	Slc31a2	LOC101394284
CCDC81	Ccdc81	LOC101397083	LMBRD2	Lmbrd2	LOC101403997	SLC32A1	Slc32a1	LOC101400613
CCDC82	Ccdc82	LOC101392532	LMCD1	Lmcd1	LOC101403020	SLC33A1	Slc33a1	LOC101404781
CCDC83	Ccdc83	LOC101395081	LMF1	Lmf1	LOC101399153	SLC34A1	Slc34a1	LOC101401926
CCDC84	Ccdc84	LOC101393543	LMF2	Lmf2	LOC101407898	SLC34A2	Slc34a2	LOC101408510
CCDC85A	Ccdc85a	LOC101398882	LMLN	Lmln	LOC101404799	SLC34A3	Slc34a3	LOC101392255
CCDC85B	Ccdc85b	LOC101389229	LMNA	Lmna	LOC101402036	SLC35A1	Slc35a1	LOC101393519
CCDC85C	Ccdc85c	LOC101387391	LMNB1	Lmnb1	LOC101388291	SLC35A2	Slc35a2	LOC101408836
CCDC86	Ccdc86	LOC101409180	LMNB2	Lmnb2	LOC101404511	SLC35A3	Slc35a3	LOC101395035
CCDC87	Ccdc87	LOC101398625	LMNTD1	Lmntd1	LOC101405886	SLC35A4	Slc35a4	LOC101392740
CCDC88A	Ccdc88a	LOC101400727	LMNTD2	Lmntd2	LOC101387764	SLC35A5	Slc35a5	LOC101392296
CCDC88B	Ccdc88b	LOC101393662	LMO1	Lmo1	LOC101389157	SLC35B1	Slc35b1	LOC101403775
CCDC88C	Ccdc88c	LOC101398036	LMO3	Lmo3	LOC101401512	SLC35B2	Slc35b2	LOC101395398
CCDC89	Ccdc89	LOC101393066	LMO4	Lmo4	LOC101405388	SLC35B3	Slc35b3	LOC101388339
CCDC9	Ccdc9	LOC101406489	LMO7	Lmo7	LOC101388140	SLC35B4	Slc35b4	LOC101401491
CCDC90B	Ccdc90b	LOC101407267	LMOD1	Lmod1	LOC101391016	SLC35C1	Slc35c1	LOC101393326
CCDC91	Ccdc91	LOC101396301	LMOD2	Lmod2	LOC101402049	SLC35C2	Slc35c2	LOC101400966
CCDC92	Ccdc92	LOC101396198	LMOD3	Lmod3	LOC101407015	SLC35D1	Slc35d1	LOC101389464
CCDC92B	Ccdc92b	LOC101395949	LMTK2	Lmtk2	LOC101408947	SLC35D2	Slc35d2	LOC101403102
CCDC93	Ccdc93	LOC101391814	LMTK3	Lmtk3	LOC101399168	SLC35D3	Slc35d3	LOC101387611
CCDC97	Ccdc97	LOC101401635	LMX1A	Lmx1a	LOC101399637	SLC35E1	Slc35e1	LOC101399366
CCER1	Ccer1	LOC101404128	LMX1B	Lmx1b	LOC101405395	SLC35E3	Slc35e3	LOC101391073
CCER2	Ccer2	LOC101407297	LNP1	Lnp1	LOC101408683	SLC35E4	Slc35e4	LOC101394912
CCHCR1	Cchcr1	LOC101395316	LNPEP	Lnpep	LOC101389171	SLC35F1	Slc35f1	LOC101387353
CCK	Cck	LOC101390785	LNPK	Lnp	LOC101399289	SLC35F2	Slc35f2	LOC101402959
CCKAR	Cckar	LOC101388283	LNX1	Lnx1	LOC101390278	SLC35F3	Slc35f3	LOC101390921
CCKBR	Cckbr	LOC101395628	LNX2	Lnx2	LOC101409004	SLC35F4	Slc35f4	LOC101390312
CCL1	Ccl1	LOC101393006	LOC10050584	1700034E13I	LOC101392842	SLC35F5	Slc35f5	LOC101388147
CCL17	Ccl17	LOC101397510	LOC10065287	Gstt4	LOC101391551	SLC35F6	Slc35f6	LOC101389055
CCL19	Ccl19	LOC106801520	LOC10272448	Syt15	LOC101389720	SLC35G2	Slc35g2	LOC101407818
CCL20	Ccl20	LOC101409057	LOC11226821	Oacyl	LOC101408104	SLC36A1	Slc36a1	LOC101396016
CCL22	Ccl22	LOC101393221	LOC11484103	1700001C19I	LOC101391148	SLC36A2	Slc36a2	LOC101396278
CCL24	Ccl24	LOC101392588	LONP1	Lonp1	LOC101387260	SLC36A3	Slc36a3	LOC101396537
CCL26	Ccl26	LOC101404071	LONP2	Lonp2	LOC101400880	SLC36A4	Slc36a4	LOC101399293
CCL28	Ccl28	LOC101393437	LONRF1	Lonrf1	LOC101399474	SLC37A1	Slc37a1	LOC101402967
CCL3	Ccl3	LOC101401949	LONRF2	Lonrf2	LOC101407181	SLC37A2	Slc37a2	LOC101395622
CCL4	Ccl4	LOC101401686	LONRF3	Lonrf3	LOC101395210	SLC37A3	Slc37a3	LOC101387641
CCL5	Ccl5	LOC101402719	LOX	Lox	LOC101393097	SLC37A4	Slc37a4	LOC101392431

CCL7	Ccl7	LOC101405465	LOXHD1	Loxhd1	LOC101408362	SLC38A1	Slc38a1	LOC101400080
CCL8	Ccl12	LOC101404932	LOXL1	Loxl1	LOC101388637	SLC38A10	Slc38a10	LOC101394656
CCM2	Ccm2	LOC101392823	LOXL2	Loxl2	LOC101387227	SLC38A11	Slc38a11	LOC101389015
CCM2L	Ccm2l	LOC101396076	LOXL3	Loxl3	LOC101401345	SLC38A2	Slc38a2	LOC101388329
CCN1	Cyr61	LOC101390330	LOXL4	Loxl4	LOC101395586	SLC38A3	Slc38a3	LOC101387849
CCN2	Ctgf	LOC101406513	LPAR1	Lpar1	LOC101390042	SLC38A4	Slc38a4	LOC101388750
CCN3	Nov	LOC101403934	LPAR2	Lpar2	LOC101401207	SLC38A5	Slc38a5	LOC101392894
CCN4	Wisp1	LOC101387462	LPAR3	Lpar3	LOC101388166	SLC38A6	Slc38a6	LOC101396179
CCN5	Wisp2	LOC101391242	LPAR5	Lpar5	LOC101404126	SLC38A7	Slc38a7	LOC101400615
CCN6	Wisp3	LOC101401462	LPCAT1	Lpcat1	LOC101390783	SLC38A8	Slc38a8	LOC101395524
CCNA1	Ccna1	LOC101398372	LPCAT2	Lpcat2	LOC101407073	SLC38A9	Slc38a9	LOC101402253
CCNA2	Ccna2	LOC101387713	LPCAT3	Lpcat3	LOC101395272	SLC39A1	Slc39a1	LOC101397095
CCNB1	Ccnb1	LOC101396959	LPCAT4	Lpcat4	LOC101400846	SLC39A10	Slc39a10	LOC101401830
CCNB2	Ccnb2	LOC101397817	LPGAT1	Lpgat1	LOC101387886	SLC39A11	Slc39a11	LOC101405516
CCNB3	Ccnb3	LOC101400724	LPIN1	Lpin1	LOC101389225	SLC39A12	Slc39a12	LOC101408798
CCNC	Ccnc	LOC101404683	LPIN2	Lpin2	LOC101404864	SLC39A13	Slc39a13	LOC101405270
CCND1	Ccnd1	LOC101402750	LPIN3	Lpin3	LOC101397767	SLC39A14	Slc39a14	LOC101404782
CCND2	Ccnd2	LOC101389919	LPL	Lpl	LOC101390544	SLC39A2	Slc39a2	LOC101403019
CCND3	Ccnd3	LOC101391815	LPO	Lpo	LOC101405694	SLC39A3	Slc39a3	LOC101403457
CCNDBP1	Ccndbp1	LOC101405835	LPP	Lpp	LOC101399635	SLC39A4	Slc39a4	LOC101389083
CCNE1	Ccne1	LOC101387418	LPXN	Lpxn	LOC101399411	SLC39A5	Slc39a5	LOC101401482
CCNE2	Ccne2	LOC101400796	LRAT	Lrat	LOC101393183	SLC39A6	Slc39a6	LOC101400144
CCNF	Ccnf	LOC101407802	LRBA	Lrba	LOC101401452	SLC39A7	Slc39a7	LOC101401036
CCNG1	Ccng1	LOC101400348	LRCH1	Lrch1	LOC101388173	SLC39A8	Slc39a8	LOC101404721
CCNG2	Ccng2	LOC101387461	LRCH2	Lrch2	LOC101401788	SLC39A9	Slc39a9	LOC101390480
CCNH	Ccnh	LOC101397888	LRCH3	Lrch3	LOC101405486	SLC3A1	Slc3a1	LOC101400811
CCNI	Ccni	LOC101409067	LRCH4	Lrch4	LOC101394526	SLC3A2	Slc3a2	LOC101404316
CCNJ	Ccnj	LOC101408210	LRFN1	Lrfn1	LOC101408604	SLC40A1	Slc40a1	LOC101396009
CCNJL	Ccnjl	LOC101404291	LRFN2	Lrfn2	LOC101396964	SLC41A1	Slc41a1	LOC101397050
CCNK	Ccnk	LOC101388505	LRFN3	Lrfn3	LOC101398390	SLC41A2	Slc41a2	LOC101407646
CCNL1	Ccnl1	LOC101391036	LRFN4	Lrfn4	LOC101402469	SLC43A1	Slc43a1	LOC101388693
CCNL2	Ccnl2	LOC101391060	LRFN5	Lrfn5	LOC101404386	SLC43A2	Slc43a2	LOC101403507
CCNO	Ccno	LOC101401026	LRG1	Lrg1	LOC101398569	SLC43A3	Slc43a3	LOC101388186
CCNT1	Ccnt1	LOC101396191	LRGUK	Lrguk	LOC101399394	SLC44A1	Slc44a1	LOC101405146
CCNT2	Ccnt2	LOC101388735	LRIF1	Lrif1	LOC101393765	SLC44A2	Slc44a2	LOC101390458
CCNY	Ccny	LOC101406281	LRIG1	Lrig1	LOC101391452	SLC44A3	Slc44a3	LOC101400841
CCNYL1	Ccnyl1	LOC101392874	LRIG2	Lrig2	LOC101408086	SLC44A4	Slc44a4	LOC101391993
CCP110	Ccp110	LOC101390708	LRIG3	Lrig3	LOC101407608	SLC44A5	Slc44a5	LOC101397334
CCPG1	Ccpg1	LOC101389854	LRIT1	Lrit1	LOC101394309	SLC45A1	Slc45a1	LOC101405228
CCR1	Ccr1	LOC101402747	LRIT2	Lrit2	LOC101407522	SLC45A2	Slc45a2	LOC101398245
CCR10	Ccr10	LOC101394077	LRIT3	Lrit3	LOC101399724	SLC45A3	Slc45a3	LOC101398000
CCR3	Ccr3	LOC101403008	LRMDA	1700112E06F	LOC101402866	SLC45A4	Slc45a4	LOC101408220
CCR4	Ccr4	LOC101392998	LRMP	Lrmp	LOC101390167	SLC46A1	Slc46a1	LOC101398367
CCR7	Ccr7	LOC101393823	LRP1	Lrp1	LOC101389606	SLC46A2	Slc46a2	LOC101394044
CCR8	Ccr8	LOC101408434	LRP10	Lrp10	LOC101388546	SLC46A3	Slc46a3	LOC101389729
CCS	Ccs	LOC101399150	LRP11	Lrp11	LOC101395197	SLC47A1	Slc47a1	LOC101405956
CCSAP	Ccsap	LOC106801340	LRP12	Lrp12	LOC101394148	SLC49A3	Mfsd7a	LOC101398283
CCSER1	Ccser1	LOC101387307	LRP1B	Lrp1b	LOC101405659	SLC49A4	Dirc2	LOC101403132
CCSER2	Ccser2	LOC101393642	LRP2	Lrp2	LOC101403494	SLC4A1	Slc4a1	LOC101392964
CCT2	Cct2	LOC101394062	LRP2BP	Lrp2bp	LOC101407775	SLC4A10	Slc4a10	LOC101392535
CCT3	Cct3	LOC101405889	LRP3	Lrp3	LOC101405549	SLC4A11	Slc4a11	LOC101408831
CCT4	Cct4	LOC101393394	LRP4	Lrp4	LOC101399931	SLC4A1AP	Slc4a1ap	LOC101398633
CCT5	Cct5	LOC101404807	LRP5	Lrp5	LOC101408673	SLC4A2	Slc4a2	LOC101408990
CCT6B	Cct6b	LOC101403369	LRP6	Lrp6	LOC101392378	SLC4A3	Slc4a3	LOC101399903

CCT7	Cct7	LOC101389039	LRP8	Lrp8	LOC101403438	SLC4A4	Slc4a4	LOC101390698
CCT8	Cct8	LOC101396896	LRPAP1	Lrpap1	LOC101400448	SLC4A5	Slc4a5	LOC101395615
CD101	Cd101	LOC101397548	LRPPRC	Lrpprc	LOC101402037	SLC4A7	Slc4a7	LOC101408515
CD109	Cd109	LOC101400221	LRRC1	Lrrc1	LOC101402508	SLC4A8	Slc4a8	LOC101395412
CD14	Cd14	LOC101392223	LRRC10	Lrrc10	LOC101394301	SLC4A9	Slc4a9	LOC101395635
CD151	Cd151	LOC101391619	LRRC10B	Lrrc10b	LOC101393401	SLC50A1	Slc50a1	LOC101389730
CD160	Cd160	LOC101401281	LRRC14	Lrrc14	LOC101408270	SLC51A	Slc51a	LOC101406966
CD163	Cd163	LOC101393084	LRRC14B	Lrrc14b	LOC106803483	SLC51B	Slc51b	LOC101388044
CD164	Cd164	LOC101394365	LRRC17	Lrrc17	LOC101390356	SLC52A2	Slc52a2	LOC101390037
CD164L2	Cd164l2	LOC101401733	LRRC18	Lrrc18	LOC101408309	SLC52A3	Slc52a3	LOC101397342
CD180	Cd180	LOC101395824	LRRC19	Lrrc19	LOC101398509	SLC5A1	Slc5a1	LOC101408216
CD19	Cd19	LOC101404848	LRRC2	Lrrc2	LOC101404504	SLC5A10	Slc5a10	LOC101395078
CD1D	Cd1d1	LOC101391808	LRRC20	Lrrc20	LOC101403048	SLC5A11	Slc5a11	LOC101405727
CD2	Cd2	LOC101397972	LRRC23	Lrrc23	LOC101397793	SLC5A12	Slc5a12	LOC101397450
CD200	Cd200	LOC101393236	LRRC24	Lrrc24	LOC101401993	SLC5A2	Slc5a2	LOC101408244
CD200R1	Cd200r1	LOC101403251	LRRC25	Lrrc25	LOC101389772	SLC5A3	Slc5a3	LOC101408214
CD207	Cd207	LOC101397521	LRRC26	Lrrc26	LOC101389959	SLC5A4	Slc5a4a	LOC101388333
CD22	Cd22	LOC101391777	LRRC27	Lrrc27	LOC101389051	SLC5A5	Slc5a5	LOC101392152
CD226	Cd226	LOC101394036	LRRC28	Lrrc28	LOC101393314	SLC5A6	Slc5a6	LOC101406484
CD244	Cd244	LOC101405048	LRRC30	Lrrc30	LOC101399783	SLC5A7	Slc5a7	LOC101392555
CD247	Cd247	LOC101400066	LRRC31	Lrrc31	LOC101395914	SLC5A8	Slc5a8	LOC101399595
CD248	Cd248	LOC101394327	LRRC32	Lrrc32	LOC101406051	SLC5A9	Slc5a9	LOC101400382
CD27	Cd27	LOC101407003	LRRC34	Lrrc34	LOC101390643	SLC6A1	Slc6a1	LOC101391805
CD274	Cd274	LOC101395089	LRRC36	Lrrc36	LOC101389987	SLC6A11	Slc6a11	LOC101407675
CD276	Cd276	LOC101391631	LRRC38	Lrrc38	LOC101407328	SLC6A12	Slc6a12	LOC101393581
CD28	Cd28	LOC101396711	LRRC39	Lrrc39	LOC101394266	SLC6A13	Slc6a13	LOC101393839
CD2AP	Cd2ap	LOC101389448	LRRC40	Lrrc40	LOC101394226	SLC6A14	Slc6a14	LOC101392751
CD2BP2	Cd2bp2	LOC101396316	LRRC41	Lrrc41	LOC101400844	SLC6A15	Slc6a15	LOC101399157
CD300A	Cd300a	LOC101393731	LRRC42	Lrrc42	LOC101392568	SLC6A16	Slc6a16	LOC101401786
CD300E	Cd300e	LOC101408403	LRRC43	Lrrc43	LOC101401485	SLC6A17	Slc6a17	LOC101396771
CD300LF	Cd300lf	LOC101394740	LRRC45	Lrrc45	LOC101400971	SLC6A18	Slc6a18	LOC101395540
CD300LG	Cd300lg	LOC101406312	LRRC46	Lrrc46	LOC101391506	SLC6A19	Slc6a19	LOC101389560
CD302	Cd302	LOC101395762	LRRC47	Lrrc47	LOC101398759	SLC6A2	Slc6a2	LOC101407616
CD34	Cd34	LOC101402509	LRRC49	Lrrc49	LOC101404610	SLC6A3	Slc6a3	LOC101390528
CD36	Cd36	LOC101393487	LRRC4B	Lrrc4b	LOC101392581	SLC6A4	Slc6a4	LOC101387311
CD37	Cd37	LOC101402138	LRRC52	Lrrc52	LOC101400507	SLC6A5	Slc6a5	LOC101394942
CD38	Cd38	LOC101389719	LRRC55	Lrrc55	LOC101409010	SLC6A6	Slc6a6	LOC101390461
CD3D	Cd3d	LOC101398764	LRRC56	Lrrc56	LOC101388026	SLC6A7	Slc6a7	LOC101400700
CD3E	Cd3e	LOC101399201	LRRC57	Lrrc57	LOC101404258	SLC6A8	Slc6a8	LOC101407475
CD3EAP	Cd3eap	LOC101408433	LRRC58	Lrrc58	LOC101402378	SLC6A9	Slc6a9	LOC101389251
CD3G	Cd3g	LOC101398506	LRRC59	Lrrc59	LOC101387232	SLC7A1	Slc7a1	LOC101390425
CD4	Cd4	LOC101387491	LRRC6	Lrrc6	LOC101387988	SLC7A10	Slc7a10	LOC101395372
CD40	Cd40	LOC101401228	LRRC63	Lrrc63	LOC106802335	SLC7A11	Slc7a11	LOC101400868
CD40LG	Cd40lg	LOC101402754	LRRC66	Lrrc66	LOC101388621	SLC7A13	Slc7a13	LOC101398229
CD44	Cd44	LOC101407637	LRRC69	Lrrc69	LOC101396460	SLC7A14	Slc7a14	LOC101393202
CD47	Cd47	LOC101407717	LRRC7	Lrrc7	LOC101393982	SLC7A2	Slc7a2	LOC101394639
CD48	Cd48	LOC101394524	LRRC71	Lrrc71	LOC101390846	SLC7A3	Slc7a3	LOC101407988
CD5	Cd5	LOC101393837	LRRC72	Lrrc72	LOC101393463	SLC7A4	Slc7a4	LOC101387861
CD53	Cd53	LOC101394022	LRRC73	Lrrc73	LOC101400685	SLC7A5	Slc7a5	LOC101389049
CD55	Cd55	LOC101388477	LRRC74A	Lrrc74a	LOC101391827	SLC7A6	Slc7a6	LOC101399304
CD6	Cd6	LOC101393578	LRRC74B	Lrrc74b	LOC101387348	SLC7A6OS	Slc7a6os	LOC101399569
CD63	Cd63	LOC101393303	LRRC75A	Lrrc75a	LOC101404648	SLC7A7	Slc7a7	LOC101389671
CD68	Cd68	LOC101401158	LRRC75B	Lrrc75b	LOC101397476	SLC7A8	Slc7a8	LOC101405832
CD69	Cd69	LOC101407805	LRRC8A	Lrrc8a	LOC101401651	SLC7A9	Slc7a9	LOC101406593

CD7	Cd7	LOC101405426	LRRC8B	Lrrc8b	LOC101392227	SLC8A1	Slc8a1	LOC101406149
CD70	Cd70	LOC101395462	LRRC8C	Lrrc8c	LOC101391627	SLC8A2	Slc8a2	LOC101407554
CD72	Cd72	LOC101393696	LRRC8D	Lrrc8d	LOC101391038	SLC8A3	Slc8a3	LOC101392785
CD74	Cd74	LOC101398268	LRRC8E	Lrrc8e	LOC101407477	SLC8B1	Slc8b1	LOC101391495
CD79A	Cd79a	LOC101407455	LRRC9	Lrrc9	LOC101408287	SLC9A1	Slc9a1	LOC101399805
CD79B	Cd79b	LOC101393643	LRRCC1	Lrrcc1	LOC101395982	SLC9A2	Slc9a2	LOC101389393
CD81	Cd81	LOC101397121	LRRD1	Lrrd1	LOC101397421	SLC9A3	Slc9a3	LOC101387943
CD82	Cd82	LOC101391601	LRRFIP1	Lrrfip1	LOC101404288	SLC9A3R1	Slc9a3r1	LOC101409076
CD83	Cd83	LOC101403676	LRRFIP2	Lrrfip2	LOC101398986	SLC9A3R2	Slc9a3r2	LOC101388963
CD84	Cd84	LOC101404264	LRRIQ1	Lrriq1	LOC101406933	SLC9A4	Slc9a4	LOC101389139
CD86	Cd86	LOC101399589	LRRIQ3	Lrriq3	LOC101396810	SLC9A5	Slc9a5	LOC101389466
CD8A	Cd8a	LOC101394582	LRRIQ4	Lrriq4	LOC101390392	SLC9A6	Slc9a6	LOC101400217
CD8B	Cd8b1	LOC101401346	LRRK1	Lrrk1	LOC101390247	SLC9A7	Slc9a7	LOC101399499
CD9	Cd9	LOC101408239	LRRK2	Lrrk2	LOC101400033	SLC9A8	Slc9a8	LOC101394729
CD96	Cd96	LOC101395180	LRRN3	Lrrn3	LOC101396322	SLC9A9	Slc9a9	LOC101394769
CD99L2	Cd99l2	LOC101396515	LRRN4	Lrrn4	LOC101402714	SLC9B1	Slc9b1	LOC101404976
CDA	Cda	LOC101391908	LRRN4CL	Lrrn4cl	LOC106803001	SLC9B2	Slc9b2	LOC101387168
CDADC1	Cdadc1	LOC101391850	LRRTM1	Lrrtm1	LOC101407536	SLC9C1	Slc9c1	LOC101403782
CDAN1	Cdan1	LOC101404783	LRRTM2	Lrrtm2	LOC101402568	SLCO1A2	Slco1a5	LOC101405885
CDC123	Cdc123	LOC101396005	LRRTM3	Lrrtm3	LOC101393799	SLCO1C1	Slco1c1	LOC101405436
CDC14A	Cdc14a	LOC101393102	LRSAM1	Lrsam1	LOC101408973	SLCO2A1	Slco2a1	LOC101389314
CDC14B	Cdc14b	LOC101394774	LRTM1	Lrtm1	LOC101389065	SLCO2B1	Slco2b1	LOC101401243
CDC16	Cdc16	LOC101392516	LRTM2	Lrtm2	LOC101397532	SLCO3A1	Slco3a1	LOC101396378
CDC20	Cdc20	LOC101405467	LRRC51	Lrrc51	LOC106802433	SLCO4A1	Slco4a1	LOC101398019
CDC20B	Cdc20b	LOC101400230	TOMT	Tomt	LOC101388426	SLCO4C1	Slco4c1	LOC101387854
CDC23	Cdc23	LOC101405718	LRWD1	Lrwd1	LOC101407836	SLCO5A1	Slco5a1	LOC101395612
CDC25A	Cdc25a	LOC101388709	LSAMP	Lsamp	LOC101407798	SLF1	Slf1	LOC101393423
CDC25B	Cdc25b	LOC101406050	LSG1	Lsg1	LOC101393200	SLFN14	Slfn14	LOC101405262
CDC25C	Cdc25c	LOC101405027	LSM1	Lsm1	LOC101391321	SLFN5	Slfn5	LOC101392491
CDC26	Cdc26	LOC101395049	LSM10	Lsm10	LOC101393623	SLFNL1	Slfnl1	LOC101390136
CDC27	Cdc27	LOC101406139	LSM11	Lsm11	LOC101396188	SLIRP	Slirp	LOC101396529
CDC34	Cdc34	LOC101393686	LSM12	Lsm12	LOC101390502	SLIT1	Slit1	LOC101388488
CDC37	Cdc37	LOC101388726	LSM14A	Lsm14a	LOC101408768	SLIT2	Slit2	LOC101393060
CDC37L1	Cdc37l1	LOC101397881	LSM14B	Lsm14b	LOC101400706	SLIT3	Slit3	LOC101398267
CDC40	Cdc40	LOC101396692	LSM2	Lsm2	LOC101393443	SLITRK1	Slitrk1	LOC101393023
CDC42	Cdc42	LOC101397657	LSM3	Lsm3	LOC101390204	SLITRK2	Slitrk2	LOC101387860
CDC42BPA	Cdc42bpa	LOC101388618	LSM4	Lsm4	LOC101390040	SLITRK3	Slitrk3	LOC106799954
CDC42BPB	Cdc42bpb	LOC101394926	LSM5	Lsm5	LOC101404501	SLITRK4	Slitrk4	LOC101388724
CDC42BPG	Cdc42bpg	LOC101397787	LSM6	Lsm6	LOC101397030	SLITRK5	Slitrk5	LOC101393526
CDC42EP1	Cdc42ep1	LOC101401177	LSM8	Lsm8	LOC101408509	SLITRK6	Slitrk6	LOC101393282
CDC42EP2	Cdc42ep2	LOC101405535	LSMEM1	Lsmem1	LOC101394763	SLK	Slk	LOC101399985
CDC42EP3	Cdc42ep3	LOC101405364	LSMEM2	Lsmem2	LOC101408692	SLMAP	Slmap	LOC101393760
CDC42EP4	Cdc42ep4	LOC101406906	LSP1	Lsp1	LOC101399773	SLN	Sln	LOC101403223
CDC42SE1	Cdc42se1	LOC101397350	LSR	Lsr	LOC101390780	SLPI	Slpi	LOC101394146
CDC42SE2	Cdc42se2	LOC101406603	LSS	Lss	LOC101388840	SLTM	Sltm	LOC101409128
CDC45	Cdc45	LOC101395122	LST1	Lst1	LOC101390477	SLU7	Slu7	LOC101403496
CDC5L	Cdc5l	LOC101394127	LTA	Lta	LOC101400426	SLX4	Slx4	LOC101398891
CDC6	Cdc6	LOC101395863	LTA4H	Lta4h	LOC101390940	SLX4IP	Slx4ip	LOC101401945
CDC7	Cdc7	LOC101389248	LTB	Ltb	LOC101399892	SMAD1	Smad1	LOC101395812
CDC73	Cdc73	LOC101394856	LTB4R2	Ltb4r2	LOC101392233	SMAD2	Smad2	LOC101389509
CDCA2	Cdca2	LOC101394407	LTBP1	Ltbp1	LOC101387751	SMAD3	Smad3	LOC101397221
CDCA3	Cdca3	LOC101399155	LTBP2	Ltbp2	LOC101406285	SMAD4	Smad4	LOC101396872
CDCA5	Cdca5	LOC101400467	LTBP3	Ltbp3	LOC101404745	SMAD5	Smad5	LOC101389662
CDCA7	Cdca7	LOC101395322	LTBP4	Ltbp4	LOC101396249	SMAD6	Smad6	LOC101396950

CDCA7L	Cdca7l	LOC101402831	LTBR	Ltbr	LOC101407275	SMAD7	Smad7	LOC101390636
CDCA8	Cdca8	LOC101397052	LTC4S	Ltc4s	LOC101393212	SMAD9	Smad9	LOC101398881
CDCP1	Cdcp1	LOC101400123	LTF	Ltf	LOC101404243	SMAGP	Smagp	LOC101388158
CDCP2	Cdcp2	LOC101403962	LTK	Ltk	LOC101402066	SMAP1	Smap1	LOC101394624
CDH1	Cdh1	LOC101401233	LTN1	Ltn1	LOC101395931	SMAP2	Smap2	LOC101408208
CDH10	Cdh10	LOC101394122	LTO1	Oraov1	LOC101402487	SMARCA1	Smarca1	LOC101392978
CDH11	Cdh11	LOC101401412	LTV1	Ltv1	LOC101393761	SMARCA2	Smarca2	LOC101394512
CDH12	Cdh12	LOC101404876	LUC7L	Luc7l	LOC101406067	SMARCA4	Smarca4	LOC101391461
CDH13	Cdh13	LOC101396568	LUC7L2	Luc7l2	LOC101388581	SMARCA5	Smarca5	LOC101394110
CDH15	Cdh15	LOC101406921	LUC7L3	Luc7l3	LOC101393994	SMARCAD1	Smarcad1	LOC101388251
CDH16	Cdh16	LOC101405253	LUM	Lum	LOC101404920	SMARCAL1	Smarcal1	LOC101404287
CDH17	Cdh17	LOC101398360	LURAP1	Lurap1	LOC101400310	SMARCB1	Smarcb1	LOC101408789
CDH18	Cdh18	LOC101393869	LURAP1L	Lurap1l	LOC101389821	SMARCC1	Smarcc1	LOC101406086
CDH19	Cdh19	LOC101393018	LUZP1	Luzp1	LOC101400160	SMARCC2	Smarcc2	LOC101400441
CDH2	Cdh2	LOC101389336	LUZP2	Luzp2	LOC101404397	SMARCD1	Smarcd1	LOC101408386
CDH20	Cdh20	LOC101408010	LVRN	Lvrn	LOC101397214	SMARCD2	Smarcd2	LOC101392699
CDH22	Cdh22	LOC101390571	LXN	Lxn	LOC101393944	SMARCD3	Smarcd3	LOC101406132
CDH23	Cdh23	LOC101399290	LY6D	Ly6d	LOC101405767	SMARCE1	Smarce1	LOC101393392
CDH24	Cdh24	LOC101400672	LY6E	Ly6e	LOC101399878	SMC1A	Smc1a	LOC101397482
CDH26	Cdh26	LOC101405948	LY6G5B	Ly6g5b	LOC101397401	SMC1B	Smc1b	LOC101400817
CDH3	Cdh3	LOC101400969	LY6G5C	Ly6g5c	LOC101397151	SMC2	Smc2	LOC101398492
CDH4	Cdh4	LOC101400965	LY6G6C	Ly6g6c	LOC101396094	SMC3	Smc3	LOC101406386
CDH5	Cdh5	LOC101401845	LY6G6D	Ly6g6d	LOC101396359	SMC4	Smc4	LOC101396597
CDH6	Cdh6	LOC101394620	LY6H	Ly6h	LOC101399449	SMC5	Smc5	LOC101389315
CDH7	Cdh7	LOC101392600	LY75	Ly75	LOC101395501	SMC6	Smc6	LOC101403349
CDH8	Cdh8	LOC101401150	LY86	Ly86	LOC101390920	SMCHD1	Smchd1	LOC101405379
CDH9	Cdh9	LOC101394367	LY9	Ly9	LOC101394789	SMCO1	Smco1	LOC101409139
CDHR1	Cdhr1	LOC101394566	LY96	Ly96	LOC101402298	SMCO2	Smco2	LOC101406914
CDHR2	Cdhr2	LOC101406892	LYAR	Lyar	LOC101407621	SMCO3	Smco3	LOC101398540
CDHR3	Cdhr3	LOC101398311	LYG1	Lyg1	LOC101405887	SMCR8	Smcr8	LOC101393646
CDHR5	Cdhr5	LOC101387257	LYG2	Lyg2	LOC101405437	SMDT1	Smdt1	LOC101387668
CDIP1	Cdip1	LOC101394673	LYL1	Lyl1	LOC101397222	SMG1	Smg1	LOC101393182
CDIPT	Cdipt	LOC101394503	LYN	Lyn	LOC101391756	SMG5	Smg5	LOC101404913
CDK1	Cdk1	LOC101398942	LYNX1	Lynx1	LOC101406037	SMG6	Smg6	LOC101399051
CDK10	Cdk10	LOC101404223	LYPD1	Lypd1	LOC101389961	SMG7	Smg7	LOC101401129
CDK11B	Cdk11b	LOC101392782	LYPD2	Lypd2	LOC101407518	SMG8	Smg8	LOC101388593
CDK12	Cdk12	LOC101404989	LYPD3	Lypd3	LOC101408940	SMG9	Smg9	LOC101389749
CDK13	Cdk13	LOC101396068	LYPD4	Lypd4	LOC101408432	SMIM11A	Smim11	LOC101408734
CDK14	Cdk14	LOC101396021	LYPD5	Lypd5	LOC101403712	SMIM13	Smim13	LOC101404726
CDK15	Cdk15	LOC101392163	LYPD6	Lypd6	LOC101402082	SMIM14	Smim14	LOC101395192
CDK16	Cdk16	LOC101394410	LYPD6B	Lypd6b	LOC101407402	SMIM17	Smim17	LOC106803307
CDK17	Cdk17	LOC101391683	LYPD8	Lypd8	LOC101395655	SMIM18	Smim18	LOC101403763
CDK18	Cdk18	LOC101398500	LYPLA1	Lypla1	LOC101390082	SMIM19	Smim19	LOC101394437
CDK19	Cdk19	LOC101397475	LYPLA2	Lypla2	LOC101404957	SMIM20	Smim20	LOC101409026
CDK2	Cdk2	LOC101396192	LYPLAL1	Lyplal1	LOC101395798	SMIM22	Smim22	LOC106803090
CDK20	Cdk20	LOC101399268	LYRM1	Lyrm1	LOC101395391	SMIM23	Smim23	LOC101394386
CDK2AP1	Cdk2ap1	LOC101391743	LYRM2	Lyrm2	LOC101397897	SMIM24	Smim24	LOC101402235
CDK2AP2	Cdk2ap2	LOC101408235	LYRM4	Lyrm4	LOC101391928	SMIM29	AI413582	LOC101392864
CDK4	Cdk4	LOC101401407	LYRM7	Lyrm7	LOC101406855	SMIM4	Smim4	LOC101404141
CDK5	Cdk5	LOC101387136	LYRM9	Lyrm9	LOC101401239	SMIM5	Smim5	LOC101396210
CDK5R1	Cdk5r1	LOC101407301	LYSMD1	Lysmd1	LOC101399145	SMIM7	Smim7	LOC101404271
CDK5R2	Cdk5r2	LOC101390486	LYSMD2	Lysmd2	LOC101408703	SMIM8	Smim8	LOC101392332
CDK5RAP1	Cdk5rap1	LOC101406613	LYSMD3	Lysmd3	LOC101395291	SMN1	Smn1	LOC101395985
CDK5RAP2	Cdk5rap2	LOC101402081	LYSMD4	Lysmd4	LOC101391749	SMNDC1	Smndc1	LOC101405865

CDK5RAP3	Cdk5rap3	LOC101393232	LYST	Lyst	LOC101393562	SMO	Smo	LOC101392396
CDK6	Cdk6	LOC101399133	LYVE1	Lyve1	LOC101395450	SMOC1	Smoc1	LOC101392530
CDK7	Cdk7	LOC101397738	LYZ	9530003J23R	LOC101392618	SMOC2	Smoc2	LOC101392399
CDK8	Cdk8	LOC101407177	LYZL1	Lyzl1	LOC101395491	SMOX	Smox	LOC101404301
CDK9	Cdk9	LOC101388559	LYZL4	Lyzl4	LOC101391027	SMPD1	Smpd1	LOC101396315
CDKAL1	Cdkal1	LOC101397851	LYZL6	Lyzl6	LOC101401598	SMPD2	Smpd2	LOC101394616
CDKL1	Cdkl1	LOC101394668	LZIC	Lzic	LOC101409051	SMPD3	Smpd3	LOC101400447
CDKL2	Cdkl2	LOC101399482	LZTFL1	Lztfl1	LOC101401447	SMPDL3A	Smpdl3a	LOC101391387
CDKL3	Cdkl3	LOC101395983	LZTR1	Lztr1	LOC101400413	SMPDL3B	Smpdl3b	LOC101396793
CDKL4	Cdkl4	LOC101407093	LZTS1	Lzts1	LOC101388725	SMPX	Smpx	LOC101405695
CDKL5	Cdkl5	LOC101400806	LZTS2	Lzts2	LOC101404358	SMS	Sms	LOC101406220
CDKN1A	Cdkn1a	LOC101403388	LZTS3	Lzts3	LOC101387650	SMTN	Smtn	LOC101393554
CDKN1B	Cdkn1b	LOC101394238	M1AP	M1ap	LOC101402899	SMTNL1	Smtnl1	LOC101389228
CDKN1C	Cdkn1c	LOC101396594	M6PR	M6pr	LOC101387244	SMTNL2	Smtnl2	LOC101407965
CDKN2AIP	Cdkn2aip	LOC101403576	MAATS1	Maats1	LOC101403519	SMU1	Smu1	LOC101393045
CDKN2AIPNL	Cdkn2aipnl	LOC101395461	MAB21L1	Mab21l1	LOC101396826	SMURF1	Smurf1	LOC101400747
CDKN2B	Cdkn2b	LOC101400515	MAB21L2	Mab21l2	LOC101401892	SMURF2	Smurf2	LOC101389620
CDKN2C	Cdkn2c	LOC101403347	MAB21L3	Mab21l3	LOC101403629	SMYD1	Smyd1	LOC101395091
CDKN2D	Cdkn2d	LOC101389944	MAB21L4	2310007B03F	LOC101388156	SMYD2	Smyd2	LOC101391821
CDKN3	Cdkn3	LOC101406119	MACC1	Macc1	LOC101404853	SMYD3	Smyd3	LOC101390609
CDNF	Cdnf	LOC101392941	MACF1	Macf1	LOC101402863	SMYD4	Smyd4	LOC101401238
CDO1	Cdo1	LOC101397887	MACROD1	Macrod1	LOC101388439	SMYD5	Smyd5	LOC101390427
CDON	Cdon	LOC101399845	MACROD2	Macrod2	LOC101401682	SNAI1	Snai1	LOC101393719
CDR2	Cdr2	LOC101398655	MAD1L1	Mad1l1	LOC101396856	SNAI2	Snai2	LOC101392011
CDR2L	Cdr2l	LOC101390416	MAD2L1	Mad2l1	LOC101389272	SNAI3	Snai3	LOC101387489
CDRT1	Fbxw10	LOC101402803	MAD2L1BP	Mad2l1bp	LOC101398931	SNAP23	Snap23	LOC101403809
CDRT4	Cdrt4	LOC101406732	MAD2L2	Mad2l2	LOC101393125	SNAP25	Snap25	LOC101396645
CDS1	Cds1	LOC101398194	MADD	Madd	LOC101403429	SNAP29	Snap29	LOC101401720
CDS2	Cds2	LOC101402291	MAEA	Maea	LOC101392888	SNAP47	Snap47	LOC101402752
CDSN	Cdsn	LOC101403653	MAEL	Mael	LOC101389968	SNAP91	Snap91	LOC101408962
CDT1	Cdt1	LOC101388436	MAF1	Maf1	LOC101391216	SNAPC1	Snapc1	LOC101397243
CDV3	Cdv3	LOC101391026	MAFB	Mafb	LOC101398777	SNAPC2	Snapc2	LOC101406773
CDX1	Cdx1	LOC101400958	MAFF	Maff	LOC101389482	SNAPC3	Snapc3	LOC101408418
CDX2	Cdx2	LOC101387917	MAFG	Mafg	LOC101399745	SNAPC4	Snapc4	LOC101403650
CDX4	Cdx4	LOC101395382	MAFK	Mafk	LOC101397885	SNAPC5	Snapc5	LOC101395558
CDYL	Cdyl	LOC101392698	MAG	Mag	LOC101389745	SNAPIN	Snapin	LOC101395263
CDYL2	Cdyl2	LOC101400376	MAGED1	Maged1	LOC101407396	SNCA	Snca	LOC101408909
CEACAM1	Ceacam1	LOC101404147	MAGED2	Maged2	LOC101399709	SNCAIP	Sncaip	LOC101392224
CEACAM16	Ceacam16	LOC101392313	MAGEE2	Magee2	LOC101398402	SNCB	Sncb	LOC101406293
CEACAM19	Ceacam19	LOC101405811	MAGI1	Magi1	LOC101403978	SNCG	Sncg	LOC101390501
CEACAM5	Ceacam2	LOC101403190	MAGI2	Magi2	LOC101399852	SND1	Snd1	LOC101398558
CEBPA	Cebpa	LOC101405286	MAGI3	Magi3	LOC101407663	SNED1	Sned1	LOC101388406
CEBPB	Cebpb	LOC101392622	MAGIX	Magix	LOC101404832	SNF8	Snf8	LOC101399753
CEBPE	Cebpe	LOC101406430	MAGOH	Magoh	LOC101390007	SNIP1	Snip1	LOC101395752
CEBPG	Cebpg	LOC101405019	MAGOHB	Magohb	LOC101388864	SNN	Snn	LOC101406582
CEBPZ	Cebpz	LOC101407196	MAGT1	Magt1	LOC101408954	SNORC	3110079O15F	LOC101396184
CECR2	Cecr2	LOC101401172	MAJIN	Majin	LOC101404747	SNPH	Snph	LOC101396028
CEL	Cel	LOC101395225	MAK	Mak	LOC101408045	SNRK	Snrk	LOC101395979
CELA1	Cela1	LOC101393973	MAK16	Mak16	LOC101407947	SNRNP200	Snrnp200	LOC101398375
CELA2A	Cela2a	LOC101409144	MAL2	Mal2	LOC101391079	SNRNP25	Snrnp25	LOC101402552
CELA3A	Cela3a	LOC101397155	MALRD1	Malrd1	LOC101398258	SNRNP27	Snrnp27	LOC101401251
CELF1	Celf1	LOC101406407	MALSU1	Malsu1	LOC101400649	SNRNP40	Snrnp40	LOC101392783
CELF2	Celf2	LOC101398421	MALT1	Malt1	LOC101404532	SNRNP48	Snrnp48	LOC101389123
CELF3	Celf3	LOC101405888	MAMDC2	Mamdc2	LOC101389563	SNRNP70	Snrnp70	LOC101400294

CELF4	Celf4	LOC101403115	MAMDC4	Mamdc4	LOC101408110	SNRPA	Snrpa	LOC101397722
CELF5	Celf5	LOC101400567	MAML1	Maml1	LOC101387456	SNRPA1	Snrpa1	LOC101389718
CELF6	Celf6	LOC101407033	MAML2	Maml2	LOC101392265	SNRPB	Snrpb	LOC101393564
CELSR1	Celsr1	LOC101399238	MAML3	Maml3	LOC101397493	SNRPB2	Snrpb2	LOC101392187
CELSR2	Celsr2	LOC101404775	MAMLD1	Mamld1	LOC101398408	SNRPC	Snrpc	LOC101390895
CEMIP	Cemip	LOC101395682	MAMSTR	Mamstr	LOC101395025	SNRPD1	Snrpd1	LOC101387951
CEND1	Cend1	LOC101393762	MAN1A1	Man1a	LOC101389258	SNRPD2	Snrpd2	LOC101398983
CENPA	Cenpa	LOC101388783	MAN1A2	Man1a2	LOC101396596	SNRPD3	Snrpd3	LOC101389767
CENPB	Cenpb	LOC101406469	MAN1C1	Man1c1	LOC101389871	SNRPE	Snrpe	LOC101404625
CENPC	Cenpc1	LOC101399246	MAN2A1	Man2a1	LOC101405296	SNRPF	Snrpf	LOC101389646
CENPE	Cenpe	LOC101405248	MAN2A2	Man2a2	LOC101408653	SNRPG	Snrpg	LOC101398799
CENPF	Cenpf	LOC101393446	MAN2B1	Man2b1	LOC101396687	SNRPN	Snrpn	LOC106799944
CENPH	Cenph	LOC101397229	MAN2B2	Man2b2	LOC101404107	SNTA1	Snta1	LOC101406365
CENPI	Cenpi	LOC101401101	MAN2C1	Man2c1	LOC101397894	SNTB1	Sntb1	LOC101402022
CENPJ	Cenpj	LOC101397347	MANBA	Manba	LOC101397508	SNTB2	Sntb2	LOC101403235
CENPK	Cenpk	LOC101391982	MANBAL	Manbal	LOC101404719	SNTG1	Sntg1	LOC101402377
CENPL	Cenpl	LOC101399283	MANEA	Manea	LOC101400053	SNTG2	Sntg2	LOC101392295
CENPM	Cenpm	LOC101408583	MANEAL	Maneal	LOC101397570	SNTN	Sntn	LOC101401539
CENPN	Cenpn	LOC101399234	MANF	Manf	LOC101394105	SNU13	Nhp2l1	LOC101407100
CENPO	Cenpo	LOC101395107	MANSC1	Mansc1	LOC101392811	SNUPN	Snupn	LOC101399175
CENPP	Cenpp	LOC101400938	MANSC4	Mansc4	LOC101395177	SNURF	Snurf	LOC101406988
CENPQ	Cenpq	LOC101387883	MAOA	Maoa	LOC101403421	SNW1	Snw1	LOC101396794
CENPT	Cenpt	LOC101394830	MAOB	Maob	LOC101403158	SNX1	Snx1	LOC101406509
CENPU	Cenpu	LOC101397499	MAP11	BC037034	LOC101391214	SNX10	Snx10	LOC101395280
CENPV	Cenpv	LOC106802284	MAP1A	Map1a	LOC101408000	SNX11	Snx11	LOC101394745
CENPW	Cenpw	LOC101394885	MAP1B	Map1b	LOC101395463	SNX12	Snx12	LOC101407552
CENPX	Stra13	LOC101404647	MAP1LC3A	Map1lc3a	LOC101399996	SNX13	Snx13	LOC101407989
CEP104	Cep104	LOC101399025	MAP1LC3B	Map1lc3b	LOC101390085	SNX14	Snx14	LOC101389433
CEP112	Cep112	LOC101399917	MAP1S	Map1s	LOC101393784	SNX15	Snx15	LOC101399509
CEP120	Cep120	LOC101390951	MAP2	Map2	LOC101396799	SNX16	Snx16	LOC101389275
CEP126	Cep126	LOC101392432	MAP2K1	Map2k1	LOC101395298	SNX17	Snx17	LOC101403089
CEP128	Cep128	LOC101398858	MAP2K2	Map2k2	LOC101395808	SNX18	Snx18	LOC101398246
CEP131	Cep131	LOC101402025	MAP2K3	Map2k3	LOC101401757	SNX19	Snx19	LOC101408496
CEP135	Cep135	LOC101392397	MAP2K4	Map2k4	LOC101405518	SNX2	Snx2	LOC101391967
CEP152	Cep152	LOC101404935	MAP2K5	Map2k5	LOC101398332	SNX20	Snx20	LOC101402972
CEP162	Cep162	LOC101388468	MAP2K6	Map2k6	LOC101404109	SNX21	Snx21	LOC101405764
CEP164	Cep164	LOC101409152	MAP2K7	Map2k7	LOC101407043	SNX22	Snx22	LOC101407129
CEP170	Cep170	LOC101393928	MAP3K1	Map3k1	LOC101404534	SNX24	Snx24	LOC101391700
CEP170B	Cep170b	LOC101403509	MAP3K10	Map3k10	LOC101388293	SNX25	Snx25	LOC101407506
CEP19	Cep19	LOC101388057	MAP3K11	Map3k11	LOC101406578	SNX27	Snx27	LOC101405439
CEP192	Cep192	LOC101394358	MAP3K12	Map3k12	LOC101390659	SNX29	Snx29	LOC101404917
CEP250	Cep250	LOC101394145	MAP3K13	Map3k13	LOC101409140	SNX3	Snx3	LOC101391217
CEP290	Cep290	LOC101401265	MAP3K14	Map3k14	LOC101400529	SNX30	Snx30	LOC101393789
CEP295	Cep295	LOC101393710	MAP3K15	Map3k15	LOC101393070	SNX31	Snx31	LOC101408910
CEP350	Cep350	LOC101408980	MAP3K19	Map3k19	LOC101388146	SNX32	Snx32	LOC101387242
CEP41	Cep41	LOC101387328	MAP3K2	Map3k2	LOC101400425	SNX33	Snx33	LOC101400050
CEP44	Cep44	LOC101391002	MAP3K3	Map3k3	LOC101389534	SNX4	Snx4	LOC101404276
CEP55	Cep55	LOC101398104	MAP3K4	Map3k4	LOC101388450	SNX5	Snx5	LOC101389899
CEP57	Cep57	LOC101393297	MAP3K5	Map3k5	LOC101387109	SNX6	Snx6	LOC101399065
CEP57L1	Cep57l1	LOC101393433	MAP3K6	Map3k6	LOC101401043	SNX7	Snx7	LOC101397465
CEP63	Cep63	LOC101388203	MAP3K7	Map3k7	LOC101399361	SNX8	Snx8	LOC101396593
CEP68	Cep68	LOC101405790	MAP3K7CL	Map3k7cl	LOC101397328	SNX9	Snx9	LOC101391961
CEP70	Cep70	LOC101403619	MAP3K8	Map3k8	LOC101394459	SOAT1	Soat1	LOC101388824
CEP72	Cep72	LOC101388530	MAP3K9	Map3k9	LOC101394632	SOAT2	Soat2	LOC101390745

CEP76	Cep76	LOC101392921	MAP4	Map4	LOC101388451	SOBP	Sobp	LOC101389859
CEP78	Cep78	LOC101405907	MAP4K1	Map4k1	LOC101403541	SOCS2	Socs2	LOC101407901
CEP83	Cep83	LOC101409016	MAP4K2	Map4k2	LOC101404488	SOCS3	Socs3	LOC101388949
CEP85	Cep85	LOC101394210	MAP4K3	Map4k3	LOC101406665	SOCS4	Socs4	LOC101408632
CEP85L	Cep85l	LOC101387610	MAP4K4	Map4k4	LOC101404912	SOCS5	Socs5	LOC101396655
CEP89	Cep89	LOC101395882	MAP4K5	Map4k5	LOC101394412	SOCS7	Socs7	LOC101388953
CEP95	Cep95	LOC101395687	MAP6	Map6	LOC101403074	SOD1	Sod1	LOC101402877
CEP97	Cep97	LOC101389411	MAP6D1	Map6d1	LOC101398090	SOD2	Sod2	LOC101407206
CEPT1	Cept1	LOC101393263	MAP7	Map7	LOC101387782	SOGA1	Soga1	LOC101406645
CER1	Cer1	LOC101387239	MAP7D1	Map7d1	LOC101392426	SOGA3	Soga3	LOC101396694
CERCAM	Cercam	LOC101396264	MAP7D2	Map7d2	LOC101402813	SOHLH1	Sohlh1	LOC101389193
CERK	Cerk	LOC101399511	MAP9	Map9	LOC101387950	SOHLH2	Sohlh2	LOC101397692
CERKL	Cerkl	LOC101388484	MAPK1	Mapk1	LOC101389427	SON	Son	LOC101396724
CERS1	Cers1	LOC101398746	MAPK10	Mapk10	LOC101399912	SORBS1	Sorbs1	LOC101406198
CERS2	Cers2	LOC101395611	MAPK11	Mapk11	LOC101401867	SORBS2	Sorbs2	LOC101387808
CERS3	Cers3	LOC101391249	MAPK12	Mapk12	LOC101406833	SORBS3	Sorbs3	LOC101403546
CERS4	Cers4	LOC101405737	MAPK13	Mapk13	LOC101406277	SORCS1	Sorcs1	LOC101402347
CERS5	Cers5	LOC101387377	MAPK14	Mapk14	LOC101406526	SORCS2	Sorcs2	LOC101403237
CERS6	Cers6	LOC101405239	MAPK15	Mapk15	LOC101395903	SORCS3	Sorcs3	LOC101402091
CERT1	Col4a3bp	LOC101390875	MAPK1IP1L	Mapk1ip1l	LOC101408891	SORD	Sord	LOC101393272
CES2	Ces2h	LOC101406394	MAPK3	Mapk3	LOC101399162	SORL1	Sorl1	LOC101405330
CES3	Ces3b	LOC101406649	MAPK4	Mapk4	LOC101395217	SORT1	Sort1	LOC101407571
CES4A	Ces4a	LOC101401151	MAPK6	Mapk6	LOC101387955	SOS1	Sos1	LOC101407363
CES5A	Ces5a	LOC101387463	MAPK7	Mapk7	LOC101396907	SOS2	Sos2	LOC101394931
CETN1	Cetn1	LOC101407737	MAPK8	Mapk8	LOC101409170	SOST	Sost	LOC101387818
CETN2	Cetn2	LOC101405736	MAPK8IP1	Mapk8ip1	LOC101393575	SOSTDC1	Sostdc1	LOC101393219
CETN3	Cetn3	LOC101396067	MAPK8IP2	Mapk8ip2	LOC101388190	SOWAHA	Sowaha	LOC101400128
CFAP100	Cfap100	LOC101406007	MAPK8IP3	Mapk8ip3	LOC101393580	SOWAHD	Sowahd	LOC101399442
CFAP126	Cfap126	LOC101400764	MAPK9	Mapk9	LOC101391577	SOX10	Sox10	LOC101390517
CFAP157	Cfap157	LOC106800761	MAPKAP1	Mapkap1	LOC101403209	SOX11	Sox11	LOC101402207
CFAP161	Cfap161	LOC101403505	MAPKAPK2	Mapkapk2	LOC101392867	SOX12	Sox12	LOC101399581
CFAP20	Cfap20	LOC101397261	MAPKAPK3	Mapkapk3	LOC101393851	SOX13	Sox13	LOC101404351
CFAP206	Cfap206	LOC101392858	MAPKAPK5	Mapkapk5	LOC101396281	SOX14	Sox14	LOC101406938
CFAP221	Cfap221	LOC101394126	MAPKBP1	Mapkbp1	LOC101399354	SOX15	Sox15	LOC101400623
CFAP36	Cfap36	LOC101400274	MAPRE1	Mapre1	LOC101408786	SOX17	Sox17	LOC101390765
CFAP43	Cfap43	LOC101400698	MAPRE2	Mapre2	LOC101396695	SOX18	Sox18	LOC101401748
CFAP44	Cfap44	LOC101390769	MAPRE3	Mapre3	LOC101388104	SOX2	Sox2	LOC101400862
CFAP45	Cfap45	LOC101387349	MAPT	Mapt	LOC101404209	SOX3	Sox3	LOC101387770
CFAP46	Cfap46	LOC101388779	MARC1	1-Mar	LOC101398059	SOX30	Sox30	LOC101408552
CFAP52	Cfap52	LOC101387470	MARC2	2-Mar	LOC101397801	SOX4	Sox4	LOC101397589
CFAP53	Cfap53	LOC101392505	MARCKS	Marcks	LOC101399708	SOX5	Sox5	LOC101389138
CFAP54	Cfap54	LOC106800353	MARCKSL1	Marcksl1	LOC101400603	SOX6	Sox6	LOC101404921
CFAP57	Cfap57	LOC101404779	MARCO	Marco	LOC101389195	SOX7	Sox7	LOC101395167
CFAP58	Cfap58	LOC101389879	MARF1	Marf1	LOC101395813	SOX8	Sox8	LOC101398892
CFAP69	Cfap69	LOC101395339	MARK1	Mark1	LOC101397108	SOX9	Sox9	LOC101405256
CFAP70	Cfap70	LOC101392613	MARK2	Mark2	LOC101408325	SP1	Sp1	LOC101388931
CFAP73	Cfap73	LOC101398110	MARK3	Mark3	LOC101396217	SP100	Sp100	LOC101402523
CFAP74	Cfap74	LOC101397405	MARK4	Mark4	LOC101407910	SP140	Sp140	LOC101402011
CFAP77	Cfap77	LOC101392775	MARS2	Mars2	LOC101406121	SP2	Sp2	LOC101392287
CFAP97	Cfap97	LOC101407063	MARVELD2	Marveld2	LOC101397719	SP3	Sp3	LOC101395921
CFAP99	Cfap99	LOC101397175	MARVELD3	Marveld3	LOC101387905	SP4	Sp4	LOC101403355
CFB	Cfb	LOC101390476	MASP1	Masp1	LOC101401037	SP5	Sp5	LOC101400439
CFC1B	Cfc1	LOC101392778	MASP2	Masp2	LOC101390732	SP6	Sp6	LOC101392017
CFDP1	Cfdp1	LOC101405624	MAST1	Mast1	LOC101405309	SP7	Sp7	LOC101388490

CFH	Cfh	LOC101394092	MAST2	Mast2	LOC101406859	SP8	Sp8	LOC101403873
CFI	Cfi	LOC101400511	MAST3	Mast3	LOC101400588	SP9	Sp9	LOC101396796
CFL1	Cfl1	LOC101387490	MAST4	Mast4	LOC101395395	SPA17	Spa17	LOC101393241
CFL2	Cfl2	LOC101392459	MASTL	Mastl	LOC101397240	SPACA1	Spaca1	LOC101394198
CFLAR	Cflar	LOC101389454	MAT1A	Mat1a	LOC101397176	SPACA3	Spaca3	LOC101406505
CFP	Cfp	LOC101399227	MAT2A	Mat2a	LOC101388437	SPACA4	Spaca4	LOC101392995
CFTR	Cftr	LOC101409024	MAT2B	Mat2b	LOC101399648	SPACA5	Spaca5	LOC101397691
CGA	Cga	LOC101391550	MATK	Matk	LOC101397810	SPACA6	Spaca6	LOC101390859
CGGBP1	Cggbp1	LOC101398146	MATN1	Matn1	LOC101391568	SPACA9	Spaca9	LOC101406376
CGN	Cgn	LOC101404739	MATN2	Matn2	LOC101404371	SPAG1	Spag1	LOC101405512
CGNL1	Cgnl1	LOC101393773	MATN3	Matn3	LOC101400554	SPAG11B	Spag11b	LOC101396763
CGREF1	Cgref1	LOC101408589	MATN4	Matn4	LOC101387212	SPAG16	Spag16	LOC101399644
CGRRF1	Cgrrf1	LOC101407236	MATR3	Matr3	LOC101400923	SPAG17	Spag17	LOC101395379
CHAC1	Chac1	LOC101395384	MAU2	Mau2	LOC101402504	SPAG4	Spag4	LOC101393055
CHAD	Chad	LOC101387915	MAVS	Mavs	LOC101405523	SPAG5	Spag5	LOC101396212
CHAF1A	Chaf1a	LOC101393177	MAX	Max	LOC101403825	SPAG6	Spag6	LOC101404008
CHAF1B	Chaf1b	LOC101389458	MAZ	Maz	LOC106803361	SPAG7	Spag7	LOC101399919
CHAT	Chat	LOC101406311	MB	Mb	LOC101393841	SPAG8	Spag8	LOC101394453
CHCHD1	Chchd1	LOC101389700	MB21D2	Mb21d2	LOC101395913	SPAG9	Spag9	LOC101395607
CHCHD10	Chchd10	LOC101408006	MBD1	Mbd1	LOC101392759	SPARC	Sparc	LOC101395502
CHCHD2	Chchd2	LOC101390359	MBD2	Mbd2	LOC101397391	SPARCL1	Sparcl1	LOC101402358
CHCHD3	Chchd3	LOC101402188	MBD3	Mbd3	LOC101407734	SPAST	Spast	LOC101390438
CHCHD4	Chchd4	LOC101392502	MBD4	Mbd4	LOC101393772	SPATA1	Spata1	LOC101399914
CHCHD5	Chchd5	LOC101389542	MBD5	Mbd5	LOC101403039	SPATA13	Spata13	LOC101405265
CHCHD7	Chchd7	LOC101393072	MBD6	Mbd6	LOC101395333	SPATA16	Spata16	LOC101387885
CHD1	Chd1	LOC101396149	MBIP	Mbip	LOC101387922	SPATA17	Spata17	LOC101395109
CHD1L	Chd1l	LOC101390705	MBL2	Mbl2	LOC101407816	SPATA18	Spata18	LOC101390869
CHD2	Chd2	LOC101395508	MBLAC1	Mblac1	LOC101401723	SPATA19	Spata19	LOC101388101
CHD3	Chd3	LOC101397594	MBLAC2	Mblac2	LOC101395809	SPATA2	Spata2	LOC101394221
CHD5	Chd5	LOC101400510	MBNL1	Mbnl1	LOC101407127	SPATA20	Spata20	LOC101388430
CHD6	Chd6	LOC101397065	MBNL2	Mbnl2	LOC101397309	SPATA21	Spata21	LOC101403484
CHD7	Chd7	LOC101396033	MBNL3	Mbnl3	LOC101406479	SPATA22	Spata22	LOC101392185
CHD8	Chd8	LOC101397554	MBOAT1	Mboat1	LOC101398693	SPATA24	Spata24	LOC101399522
CHD9	Chd9	LOC101404724	MBOAT2	Mboat2	LOC101403518	SPATA25	Spata25	LOC101390829
CHDH	Chdh	LOC101387762	MBOAT4	Mboat4	LOC101402351	SPATA2L	Spata2l	LOC101405004
CHEK1	Chek1	LOC101397199	MBOAT7	Mboat7	LOC101408332	SPATA3	Spata3	LOC101390823
CHEK2	Chek2	LOC106799942	MBP	Mbp	LOC101398083	SPATA32	Spata32	LOC101408405
CHERP	Cherp	LOC101399627	MBTD1	Mbtd1	LOC101397858	SPATA4	Spata4	LOC101399989
CHFR	Chfr	LOC101401839	MBTPS1	Mbtps1	LOC101395267	SPATA45	Spata45	LOC101390393
CHGA	Chga	LOC101401763	MBTPS2	Mbtps2	LOC101405959	SPATA5	Spata5	LOC101406449
CHGB	Chgb	LOC101401503	MC3R	Mc3r	LOC101408736	SPATA6	Spata6	LOC101400821
CHI3L1	Chil1	LOC101394005	MC4R	Mc4r	LOC101407748	SPATA7	Spata7	LOC101402088
CHIC1	Chic1	LOC101395641	MCAM	Mcam	LOC101388321	SPATA9	Spata9	LOC101391884
CHIC2	Chic2	LOC101390696	MCAT	Mcat	LOC101396484	SPATC1	Spatc1	LOC101404615
CHID1	Chid1	LOC101390704	MCC	Mcc	LOC101400924	SPATC1L	Spatc1l	LOC101388577
CHIT1	Chit1	LOC101393407	MCCC1	Mccc1	LOC101399894	SPATS1	Spats1	LOC101394373
CHKA	Chka	LOC101389825	MCCC2	Mccc2	LOC101401369	SPATS2	Spats2	LOC101404025
CHKB	Chkb	LOC101387669	MCEE	Mcee	LOC101387728	SPATS2L	Spats2l	LOC101408546
CHM	Chm	LOC101393833	MCF2	Mcf2	LOC101405463	SPC24	Spc24	LOC101392146
CHML	Chml	LOC101392208	MCF2L	Mcf2l	LOC101402334	SPC25	Spc25	LOC101404714
CHMP1A	Chmp1a	LOC101405894	MCFD2	Mcfd2	LOC101396040	SPCS1	Spcs1	LOC101405715
CHMP1B	Chmp1b	LOC101390954	MCHR1	Mchr1	LOC101401258	SPCS2	Spcs2	LOC101400182
CHMP2A	Chmp2a	LOC101406588	MCIDAS	Mcidas	LOC101400761	SPCS3	Spcs3	LOC101400701
CHMP2B	Chmp2b	LOC101399245	MCL1	Mcl1	LOC101392975	SPDEF	Spdef	LOC101391561

CHMP3	Chmp3	LOC101393660	MCM10	Mcm10	LOC101395231	SPDL1	Spdl1	LOC101398011
CHMP4B	Chmp4b	LOC101405130	MCM2	Mcm2	LOC101397471	SPDYA	Spdya	LOC101395880
CHMP4C	Chmp4c	LOC101389012	MCM3	Mcm3	LOC101407228	SPECC1L	Specc1l	LOC101388384
CHMP5	Chmp5	LOC101392269	MCM3AP	Mcm3ap	LOC101389114	SPEF1	Spef1	LOC101406733
CHMP6	Chmp6	LOC101393900	MCM4	Mcm4	LOC101392954	SPEF2	Spef2	LOC101401383
CHMP7	Chmp7	LOC101387738	MCM5	Mcm5	LOC101393167	SPEG	Speg	LOC101396975
CHN1	Chn1	LOC101398351	MCM6	Mcm6	LOC101407754	SPEM1	Spem1	LOC101405173
CHN2	Chn2	LOC101389241	MCM7	Mcm7	LOC101389579	SPEM2	4933402P03F	LOC101404211
CHODL	Chodl	LOC101389367	MCM8	Mcm8	LOC101401069	SPEM3	Gm39566	LOC106802261
CHORDC1	Chordc1	LOC101400345	MCM9	Mcm9	LOC101388132	SPEN	Spen	LOC101400601
CHP1	Chp1	LOC101396339	MCMBP	Mcmbp	LOC101398947	SPESP1	Spesp1	LOC106800492
CHP2	Chp2	LOC101407741	MCMDC2	Mcmdc2	LOC101392556	SPG11	Spg11	LOC101391459
CHPF	Chpf	LOC101398102	MCOLN1	Mcoln1	LOC101389183	SPG21	Spg21	LOC101388548
CHPF2	Chpf2	LOC101406547	MCOLN2	Mcoln2	LOC101388417	SPG7	Spg7	LOC101402549
CHPT1	Chpt1	LOC106800399	MCOLN3	Mcoln3	LOC101388678	SPHK1	Sphk1	LOC101404467
CHRAC1	Chrac1	LOC101408993	MCPH1	Mcph1	LOC101398719	SPHK2	Sphk2	LOC101393675
CHRD	Chrd	LOC101392422	MCRS1	Mcrs1	LOC101404557	SPHKAP	Sphkap	LOC101387452
CHRDL1	Chrdl1	LOC101396326	MCTP1	Mctp1	LOC101393178	SPI1	Spi1	LOC101405003
CHRDL2	Chrdl2	LOC101398695	MCTP2	Mctp2	LOC101399822	SPIC	Spic	LOC101400551
CHRM1	Chrm1	LOC101404582	MCTS1	Mcts1	LOC101398133	SPICE1	Spice1	LOC101390512
CHRM3	Chrm3	LOC101396814	MCU	Mcu	LOC101395582	SPIDR	Spidr	LOC101402359
CHRM4	Chrm4	LOC101396920	MCUR1	Mcur1	LOC101404207	SPIN1	Spin1	LOC101399877
CHRNA1	Chrna1	LOC101398099	MDC1	Mdc1	LOC101406626	SPIN4	Spin4	LOC101396671
CHRNA10	Chrna10	LOC101401762	MDFI	Mdfi	LOC101394455	SPINDOC	AI846148	LOC101402634
CHRNA2	Chrna2	LOC101402539	MDFIC	Mdfic	LOC101391363	SPINK1	Spink1	LOC101394689
CHRNA3	Chrna3	LOC101393186	MDGA1	Mdga1	LOC101399798	SPINK13	Spink13	LOC101403013
CHRNA4	Chrna4	LOC101395594	MDGA2	Mdga2	LOC101401861	SPINK14	Spink14	LOC106803174
CHRNA5	Chrna5	LOC101404869	MDH1	Mdh1	LOC101390256	SPINK4	Spink4	LOC101392536
CHRNA6	Chrna6	LOC101394951	MDH1B	Mdh1b	LOC101392350	SPINK5	Spink5	LOC101393937
CHRNA7	Chrna7	LOC101408132	MDH2	Mdh2	LOC101405382	SPINK6	Spink6	LOC106803326
CHRNA9	Chrna9	LOC101396846	MDK	Mdk	LOC101396392	SPINT1	Spint1	LOC101394361
CHRNB1	Chrnb1	LOC101403944	MDM1	Mdm1	LOC101389607	SPINT2	Spint2	LOC101406251
CHRNB2	Chrnb2	LOC101405697	MDM2	Mdm2	LOC101391324	SPINT3	Spint3	LOC106801788
CHRNB3	Chrnb3	LOC101394691	MDM4	Mdm4	LOC101401913	SPIRE1	Spire1	LOC101392230
CHRNB4	Chrnb4	LOC101404611	MDN1	Mdn1	LOC101398336	SPIRE2	Spire2	LOC101406150
CHRND	Chrnd	LOC101394636	MDP1	Mdp1	LOC101394268	SPNS1	Spns1	LOC101403791
CHRNE	Chrne	LOC101396908	ME1	Me1	LOC101408269	SPNS2	Spns2	LOC101392101
CHRNG	Chrng	LOC101395063	ME2	Me2	LOC101396165	SPNS3	Spns3	LOC101409077
CHST1	Chst1	LOC101393076	ME3	Me3	LOC101397598	SPO11	Spo11	LOC101406546
CHST10	Chst10	LOC101407434	MEA1	Mea1	LOC101387197	SPOCD1	Spocd1	LOC101397833
CHST11	Chst11	LOC101407194	MEAF6	Meaf6	LOC101395494	SPOCK1	Spock1	LOC101388290
CHST13	Chst13	LOC101408105	MECOM	Mecom	LOC101396886	SPOCK2	Spock2	LOC101398008
CHST14	Chst14	LOC101391384	MECP2	Mecp2	LOC101391142	SPOCK3	Spock3	LOC101406048
CHST15	Chst15	LOC101402995	MECR	Mecr	LOC101390734	SPON1	Spon1	LOC101401182
CHST3	Chst3	LOC101398597	MED1	Med1	LOC101405432	SPON2	Spon2	LOC101392367
CHST4	Chst4	LOC101388758	MED10	Med10	LOC101392836	SPOP	Spop	LOC101403337
CHSY1	Chsy1	LOC101399044	MED11	Med11	LOC101394071	SPOPL	Spopl	LOC101406183
CHSY3	Chsy3	LOC101407383	MED12	Med12	LOC101405978	SPOUT1	D2Wsu81e	LOC101401124
CHTF18	Chtf18	LOC101399677	MED12L	Med12l	LOC101408253	SPP1	Spp1	LOC101404975
CHTF8	Chtf8	LOC101402364	MED13	Med13	LOC101392377	SPP2	Spp2	LOC101400514
CHTOP	Chtop	LOC101394842	MED13L	Med13l	LOC101389609	SPPL2A	Sppl2a	LOC101406172
CHUK	Chuk	LOC101399647	MED14	Med14	LOC101406570	SPPL2B	Sppl2b	LOC101405297
CHURC1	Churc1	LOC106801193	MED15	Med15	LOC101398403	SPPL2C	Sppl2c	LOC101403942
CIAO1	Ciao1	LOC101398129	MED16	Med16	LOC101391454	SPPL3	Sppl3	LOC101400963

CIAO2A	Fam96a	LOC101406100	MED17	Med17	LOC101398100	SPR	Spr	LOC101391677
CIAO2B	Fam96b	LOC101406135	MED18	Med18	LOC101407764	SPRED1	Spred1	LOC101408610
CIAO3	Narfl	LOC101400468	MED19	Med19	LOC101390934	SPRED2	Spred2	LOC101408575
CIAPIN1	Ciapin1	LOC101393984	MED20	Med20	LOC101392670	SPRED3	Spred3	LOC101402753
CIART	Ciart	LOC101390767	MED21	Med21	LOC101393234	SPRN	Sprn	LOC101393406
CIB1	Cib1	LOC101405611	MED22	Med22	LOC101396429	SPRTN	Sprtn	LOC101398363
CIB2	Cib2	LOC101408096	MED23	Med23	LOC101401898	SPRY2	Spry2	LOC101392768
CIB3	Cib3	LOC101401468	MED24	Med24	LOC101398202	SPRY4	Spry4	LOC101404333
CIB4	Cib4	LOC101389555	MED25	Med25	LOC101387843	SPRYD3	Spryd3	LOC101406640
CIC	Cic	LOC101404060	MED26	Med26	LOC101399106	SPRYD4	Spryd4	LOC101405676
CIDEA	Cidea	LOC101397553	MED27	Med27	LOC101391812	SPRYD7	Spryd7	LOC101393907
CIDEB	Cideb	LOC101392493	MED28	Med28	LOC101391502	SPSB1	Spsb1	LOC101407686
CIITA	Ciita	LOC101408160	MED29	Med29	LOC101388211	SPSB2	Spsb2	LOC101398307
CILP	Cilp	LOC101390296	MED30	Med30	LOC101405249	SPSB3	Spsb3	LOC101392981
CILP2	Cilp2	LOC101401467	MED31	Med31	LOC101406559	SPSB4	Spsb4	LOC101399950
CINP	Cinp	LOC101393566	MED4	Med4	LOC101389538	SPTA1	Spta1	LOC101397983
CIPC	Cipc	LOC101392872	MED6	Med6	LOC101394380	SPTAN1	Sptan1	LOC101398751
CIR1	Cir1	LOC101397055	MED7	Med7	LOC101388838	SPTB	Sptb	LOC101402594
CISD1	Cisd1	LOC101402009	MED8	Med8	LOC101406507	SPTBN1	Sptbn1	LOC101388095
CISD2	Cisd2	LOC101391392	MED9	Med9	LOC101389621	SPTBN2	Sptbn2	LOC101401347
CISD3	Cisd3	LOC101389907	MEDAG	Medag	LOC101392554	SPTBN4	Sptbn4	LOC101389321
CISH	Cish	LOC101393593	MEF2A	Mef2a	LOC101392012	SPTBN5	Sptbn5	LOC101402322
CIT	Cit	LOC101406297	MEF2B	Mef2b	LOC101404535	SPTLC1	Sptlc1	LOC101405411
CITED1	Cited1	LOC101399165	MEF2C	Mef2c	LOC101396332	SPTLC2	Sptlc2	LOC101396008
CITED2	Cited2	LOC101394959	MEF2D	Mef2d	LOC101406917	SPTLC3	Sptlc3	LOC101395254
CITED4	Cited4	LOC101389593	MEFV	Mefv	LOC101391192	SPTSSB	Sptssb	LOC101401573
CIZ1	Ciz1	LOC101393792	MEGF10	Megf10	LOC101387093	SPTY2D1	Spty2d1	LOC101391113
CKAP2	Ckap2	LOC101398536	MEGF11	Megf11	LOC101394523	SPX	Spx	LOC101407432
CKAP2L	Ckap2l	LOC101389041	MEGF6	Megf6	LOC101399465	SQLE	Sqle	LOC101394650
CKAP4	Ckap4	LOC101389487	MEGF8	Megf8	LOC101398465	SQOR	Sqrdl	LOC101396511
CKAP5	Ckap5	LOC101399506	MEGF9	Megf9	LOC101402336	SQSTM1	Sqstm1	LOC101392687
CKB	Ckb	LOC101396647	MEI1	Mei1	LOC101407537	SRA1	Sra1	LOC101394019
CKLF	Cklf	LOC101402613	MEI4	Mei4	LOC101392239	SRBD1	Srbd1	LOC101398968
CKM	Ckm	LOC101408170	MEIKIN	Meikin	LOC106803232	SRC	Src	LOC101404460
CKMT2	Ckmt2	LOC101402925	MEIOB	Meiob	LOC101391350	SRCAP	Srcap	LOC101399766
CKS1B	Cks1b	LOC101407893	MEIOC	Meioc	LOC101395425	SRCIN1	Srcin1	LOC101388687
CKS2	Cks2	LOC101402697	MEIS1	Meis1	LOC101407633	SRD5A1	Srd5a1	LOC101393092
CLASP1	Clasp1	LOC101396266	MEIS2	Meis2	LOC101407305	SRD5A2	Srd5a2	LOC101392307
CLASP2	Clasp2	LOC101394867	MEIS3	Meis3	LOC101407286	SRD5A3	Srd5a3	LOC101391956
CLASRP	Clasrp	LOC101394346	MELK	Melk	LOC101396785	SREBF1	Srebf1	LOC101403330
CLCA1	Clca1	LOC101391927	MEMO1	Memo1	LOC101391439	SREBF2	Srebf2	LOC101408061
CLCA2	Clca2	LOC101391660	MEN1	Men1	LOC101397355	SREK1	Srek1	LOC101394969
CLCA4	Clca4a	LOC101407843	MEOX1	Meox1	LOC101387561	SREK1IP1	Srek1ip1	LOC101391467
CLCC1	Clcc1	LOC101407567	MEOX2	Meox2	LOC101392957	SRF	Srf	LOC101404797
CLCF1	Clcf1	LOC101404746	MEP1A	Mep1a	LOC101398497	SRFBP1	Srfbp1	LOC101393502
CLCN1	Clcn1	LOC101400614	MEP1B	Mep1b	LOC101392928	SRGAP1	Srgap1	LOC101406542
CLCN2	Clcn2	LOC101390391	MEPCE	Mepce	LOC101392756	SRGAP2	Srgap2	LOC101394809
CLCN3	Clcn3	LOC101387142	MEPE	Mepe	LOC101404201	SRGAP3	Srgap3	LOC101394886
CLCN4	Clcn4	LOC101406737	MERTK	Mertk	LOC101397604	SRGN	Srgn	LOC101408293
CLCN5	Clcn5	LOC101401422	MESP1	Mesp1	LOC106802087	SRI	Sri	LOC101392956
CLCN6	Clcn6	LOC101393796	MESP2	Mesp2	LOC101403412	SRL	Srl	LOC101397446
CLCN7	Clcn7	LOC101395270	MEST	Mest	LOC101408593	SRM	Srm	LOC101404708
CLCNKB	Clcnka	LOC101401828	MET	Met	LOC101388527	SRMS	Srms	LOC101393638
CLDN1	Cldn1	LOC101398089	METAP1	Metap1	LOC101393058	SRP14	Srp14	LOC101388375

CLDN10	Cldn10	LOC101395656	METAP1D	Metap1d	LOC101395926	SRP19	Srp19	LOC101402060
CLDN11	Cldn11	LOC101393445	METAP2	Metap2	LOC101388521	SRP54	Srp54a	LOC101391598
CLDN12	Cldn12	LOC101395767	METRN	Metrn	LOC101400992	SRP68	Srp68	LOC101400887
CLDN14	Cldn14	LOC101389705	METTL1	Mettl1	LOC101402095	SRP72	Srp72	LOC101394345
CLDN15	Cldn15	LOC101388299	METTL11B	Mettl11b	LOC101405325	SRP9	Srp9	LOC101406932
CLDN16	Cldn16	LOC101397827	METTL14	Mettl14	LOC101391646	SRPK1	Srpk1	LOC101407142
CLDN17	Cldn17	LOC101398954	METTL15	Mettl15	LOC101400204	SRPK2	Srpk2	LOC101409019
CLDN18	Cldn18	LOC101406492	METTL16	Mettl16	LOC101398120	SRPK3	Srpk3	LOC101408617
CLDN19	Cldn19	LOC101404939	METTL17	Mettl17	LOC101403278	SRPRA	Srpr	LOC101400381
CLDN2	Cldn2	LOC101387760	METTL18	Mettl18	LOC101406444	SRPRB	Srprb	LOC101390020
CLDN20	Cldn20	LOC101387088	METTL21A	Mettl21a	LOC101402520	SRPX	Srpx	LOC101387823
CLDN23	Cldn23	LOC101399990	METTL21C	Mettl21c	LOC101405483	SRPX2	Srpx2	LOC101403454
CLDN24	Cldn24	LOC101403308	METTL22	Mettl22	LOC101389148	SRR	Srr	LOC101399577
CLDN3	Cldn3	LOC101400410	METTL23	Mettl23	LOC101406465	SRRD	Srrd	LOC101391653
CLDN4	Cldn4	LOC101400136	METTL24	Mettl24	LOC101396955	SRRM1	Srrm1	LOC101387536
CLDN5	Cldn5	LOC101394877	METTL25	Mettl25	LOC101398631	SRRM2	Srrm2	LOC101405359
CLDN6	Cldn6	LOC106803097	METTL3	Mettl3	LOC101396255	SRRM3	Srrm3	LOC101392849
CLDN7	Cldn7	LOC101387224	METTL5	Mettl5	LOC101401218	SRRM4	Srrm4	LOC101396539
CLDN8	Cldn8	LOC101399213	METTL6	Mettl6	LOC101397208	SRRT	Srrt	LOC101398740
CLDN9	Cldn9	LOC101404051	METTL7B	Mettl7b	LOC101392087	SRSF1	Srsf1	LOC101404477
CLDND1	Cldnd1	LOC101404398	METTL8	Mettl8	LOC101402349	SRSF10	Srsf10	LOC101406708
CLDND2	Cldnd2	LOC101401812	METTL9	Mettl9	LOC101393354	SRSF11	Srsf11	LOC101395768
CLEC10A	Mgl2	LOC101388760	MEX3A	Mex3a	LOC101401770	SRSF12	Srsf12	LOC101395300
CLEC11A	Clec11a	LOC101393339	MEX3B	Mex3b	LOC101394150	SRSF2	Srsf2	LOC101407169
CLEC12A	Clec12a	LOC101404918	MEX3C	Mex3c	LOC101406265	SRSF3	Srsf3	LOC101403907
CLEC12B	Clec12b	LOC101404392	MFAP2	Mfap2	LOC101404010	SRSF4	Srsf4	LOC101390478
CLEC14A	Clec14a	LOC101407894	MFAP3	Mfap3	LOC101392616	SRSF5	Srsf5	LOC101391826
CLEC16A	Clec16a	LOC101407642	MFAP3L	Mfap3l	LOC101388342	SRSF6	Srsf6	LOC101396806
CLEC19A	Gm32766	LOC101399264	MFAP4	Mfap4	LOC101397178	SRSF7	Srsf7	LOC101402909
CLEC1A	Clec1a	LOC101406585	MFAP5	Mfap5	LOC101389152	SRSF9	Srsf9	LOC101403668
CLEC2L	Clec2l	LOC101394223	MFF	Mff	LOC101406975	SS18	Ss18	LOC101397031
CLEC3A	Clec3a	LOC101401427	MFGE8	Mfge8	LOC101400883	SS18L1	Ss18l1	LOC101400443
CLEC3B	Clec3b	LOC101399694	MFHAS1	Mfhas1	LOC101400249	SSB	Ssb	LOC101400957
CLEC4D	Clec4d	LOC101392825	MFN1	Mfn1	LOC101388562	SSBP2	Ssbp2	LOC101401105
CLEC4E	Clec4e	LOC101408586	MFN2	Mfn2	LOC101405750	SSBP3	Ssbp3	LOC101394249
CLEC4F	Clec4f	LOC101404218	MFNG	Mfng	LOC101400473	SSBP4	Ssbp4	LOC101388998
CLEC4G	Clec4g	LOC101408366	MFRP	Mfrp	LOC101387546	SSC4D	Ssc4d	LOC101393104
CLEC5A	Clec5a	LOC101405949	MFSD1	Mfsd1	LOC101394187	SSC5D	Ssc5d	LOC101391524
CLEC7A	Clec7a	LOC101406837	MFSD10	Mfsd10	LOC101399398	SSH1	Ssh1	LOC101393458
CLEC9A	Clec9a	LOC101404127	MFSD11	Mfsd11	LOC101406730	SSH2	Ssh2	LOC101388172
CLGN	Clgn	LOC101396439	MFSD12	Mfsd12	LOC101401712	SSH3	Ssh3	LOC101404225
CLHC1	Clhc1	LOC101408151	MFSD14A	Mfsd14a	LOC101394780	SSMEM1	Ssmem1	LOC101389239
CLIC1	Clic1	LOC101394542	MFSD14B	Mfsd14b	LOC101394118	SSPN	Sspn	LOC101391595
CLIC3	Clic3	LOC101387788	MFSD2A	Mfsd2a	LOC101405858	SSR1	Ssr1	LOC101390072
CLIC4	Clic4	LOC101393707	MFSD2B	Mfsd2b	LOC101398713	SSR2	Ssr2	LOC101400371
CLIC5	Clic5	LOC101393034	MFSD3	Mfsd3	LOC101408963	SSR3	Ssr3	LOC101389846
CLIC6	Clic6	LOC101397764	MFSD4A	Mfsd4a	LOC101398257	SSR4	Ssr4	LOC101387783
CLINT1	Clint1	LOC101407504	MFSD5	Mfsd5	LOC101408814	SSRP1	Ssrp1	LOC101387665
CLIP1	Clip1	LOC101409162	MFSD6	Mfsd6	LOC101399120	SST	Sst	LOC101400771
CLIP2	Clip2	LOC101390542	MFSD6L	Mfsd6l	LOC101389126	SSTR2	Sstr2	LOC101405952
CLIP3	Clip3	LOC101390275	MFSD8	Mfsd8	LOC101404548	SSTR5	Sstr5	LOC101398627
CLIP4	Clip4	LOC101395106	MFSD9	Mfsd9	LOC101405438	SSU72	Ssu72	LOC101392078
CLK1	Clk1	LOC101387715	MGA	Mga	LOC101398914	SSUH2	Ssu2	LOC101402758
CLK2	Clk2	LOC101394243	MGAM	Mgam	LOC106801825	SSX2IP	Ssx2ip	LOC101387141

CLK3	Clk3	LOC101393013	MGARP	Mgarp	LOC101399199	ST13	St13	LOC101401959
CLK4	Clk4	LOC101396633	MGAT4A	Mgat4a	LOC101403426	ST14	St14	LOC101397277
CLMN	Clmn	LOC101408408	MGAT4B	Mgat4b	LOC101392949	ST18	St18	LOC101387156
CLMP	Clmp	LOC101405753	MGAT4D	Mgat4d	LOC106801248	ST3GAL1	St3gal1	LOC101389021
CLN3	Cln3	LOC101408591	MGAT5	Mgat5	LOC101389446	ST3GAL2	St3gal2	LOC101392095
CLN5	Cln5	LOC101388912	MGAT5B	Mgat5b	LOC101407963	ST3GAL3	St3gal3	LOC101408353
CLN6	Cln6	LOC101399875	MGLL	Mgll	LOC101397980	ST3GAL4	St3gal4	LOC101402044
CLN8	Cln8	LOC101404926	MGME1	Mgme1	LOC101389624	ST3GAL5	St3gal5	LOC101390933
CLNK	Clnk	LOC101405344	MGMT	Mgmt	LOC101397870	ST3GAL6	St3gal6	LOC101405369
CLNS1A	Clns1a	LOC101408315	MGP	Mgp	LOC101398797	ST6GAL1	St6gal1	LOC101402339
CLOCK	Clock	LOC101392214	MGRN1	Mgrn1	LOC101393404	ST6GAL2	St6gal2	LOC101392291
CLP1	Clp1	LOC101390263	MGST1	Mgst1	LOC101401250	ST6GALNAC2	St6galnac2	LOC101405953
CLPB	Clpb	LOC101389901	MGST2	Mgst2	LOC101397755	ST6GALNAC3	St6galnac3	LOC101397848
CLPP	Clpp	LOC101406249	MGST3	Mgst3	LOC101400772	ST6GALNAC4	St6galnac4	LOC101391056
CLPS	Clps	LOC101407682	MIA	Mia	LOC101398232	ST6GALNAC5	St6galnac5	LOC101400707
CLPSL2	Clpsl2	LOC106800909	MIA2	Mia2	LOC101404914	ST6GALNAC6	St6galnac6	LOC101390812
CLPTM1	Clptm1	LOC101393932	MIA3	Mia3	LOC101404499	ST7	St7	LOC101387682
CLPTM1L	Clptm1l	LOC101390277	MIB1	Mib1	LOC101388461	ST7L	St7l	LOC101388459
CLPX	Clpx	LOC101390034	MIB2	Mib2	LOC101392344	ST8SIA1	St8sia1	LOC101387233
CLRN1	Clrn1	LOC101408513	MICAL1	Mical1	LOC101394883	ST8SIA2	St8sia2	LOC101395769
CLRN2	Clrn2	LOC101390834	MICAL2	Mical2	LOC101397706	ST8SIA3	St8sia3	LOC101401899
CLRN3	Clrn3	LOC101398709	MICAL3	Mical3	LOC101402822	ST8SIA4	St8sia4	LOC101388120
CLSPN	Clspn	LOC101389783	MICALCL	Micalcl	LOC101399686	ST8SIA5	St8sia5	LOC101387189
CLSTN1	Clstn1	LOC101403657	MICALL1	Micall1	LOC101388962	ST8SIA6	St8sia6	LOC101387970
CLSTN2	Clstn2	LOC101391281	MICALL2	Micall2	LOC101398397	STAB1	Stab1	LOC101403622
CLSTN3	Clstn3	LOC101394091	MICU1	Micu1	LOC101395840	STAB2	Stab2	LOC101404322
CLTA	Clta	LOC101393956	MICU2	Micu2	LOC101402203	STAC	Stac	LOC101396851
CLTB	Cltb	LOC101407867	MICU3	Micu3	LOC101396189	STAC2	Stac2	LOC101406322
CLTC	Cltc	LOC101389814	MID1	Mid1	LOC101406998	STAC3	Stac3	LOC101392357
CLTRN	Tmem27	LOC101396220	MID1IP1	Mid1ip1	LOC101408147	STAG1	Stag1	LOC101408254
CLU	Clu	LOC101402289	MID2	Mid2	LOC101391120	STAG2	Stag2	LOC101395617
CLUAP1	Cluap1	LOC101399415	MIDN	Midn	LOC101388209	STAG3	Stag3	LOC101391976
CLUH	Cluh	LOC101397428	MIEF1	Mief1	LOC101398214	STAM	Stam	LOC101387534
CLVS1	Clvs1	LOC101396302	MIEF2	Mief2	LOC101393146	STAM2	Stam2	LOC101398587
CLVS2	Clvs2	LOC101391634	MIEN1	Mien1	LOC101402372	STAMBP	Stambp	LOC101387154
CLYBL	Clybl	LOC101401564	MIER1	Mier1	LOC101389024	STAMBPL1	Stambpl1	LOC101396584
CMA1	Cma1	LOC101389502	MIER2	Mier2	LOC101394107	STAP1	Stap1	LOC101399518
CMAS	Cmas	LOC101409084	MIER3	Mier3	LOC101405314	STAP2	Stap2	LOC101393684
CMBL	Cmbl	LOC101404550	MIF	Mif	LOC101392061	STAR	Star	LOC101390657
CMC2	Cmc2	LOC101400104	MIF4GD	Mif4gd	LOC101393644	STARD10	Stard10	LOC101391504
CMC4	Cmc4	LOC106799935	MIGA1	Fam73a	LOC101402362	STARD13	Stard13	LOC101395700
CMIP	Cmip	LOC101398209	MIGA2	Fam73b	LOC101403475	STARD3	Stard3	LOC101403855
CMPK1	Cmpk1	LOC101399418	MIIP	Miip	LOC106801128	STARD3NL	Stard3nl	LOC101400199
CMPK2	Cmpk2	LOC101394084	MILR1	Milr1	LOC101389379	STARD4	Stard4	LOC101404247
CMSS1	Cmss1	LOC101407452	MINDY1	Fam63a	LOC101396304	STARD5	Stard5	LOC101394401
CMTM1	Cmtm1	LOC101400616	MINDY2	Fam63b	LOC101396603	STARD6	Stard6	LOC101397902
CMTM2	Cmtm2b	LOC101402884	MINDY3	Fam188a	LOC101389868	STARD7	Stard7	LOC101397603
CMTM3	Cmtm3	LOC101400882	MINDY4	Fam188b	LOC101407651	STARD8	Stard8	LOC101390867
CMTM4	Cmtm4	LOC101403150	MINK1	Mink1	LOC101396116	STAT1	Stat1	LOC101399901
CMTM5	Cmtm5	LOC101403277	MINPP1	Minpp1	LOC101399857	STAT2	Stat2	LOC101404454
CMTM6	Cmtm6	LOC101391618	MIOS	Mios	LOC101387386	STAT3	Stat3	LOC101398124
CMTM7	Cmtm7	LOC101398227	MIOX	Miox	LOC101407641	STAT4	Stat4	LOC101400163
CMTM8	Cmtm8	LOC101391369	MIP	Mip	LOC101405417	STAT5A	Stat5a	LOC101399057
CMTR1	Cmtr1	LOC101400331	MIPEP	Mipep	LOC101404306	STAT5B	Stat5b	LOC101399667

CMYA5	Cmya5	LOC101405565	MIPOL1	Mipol1	LOC101398298	STAT6	Stat6	LOC101389884
CNBD1	Cnbd1	LOC101397460	MIS18A	Mis18a	LOC101403579	STAU1	Stau1	LOC101396199
CNBD2	Cnbd2	LOC101398276	MIS18BP1	Mis18bp1	LOC101402723	STAU2	Stau2	LOC101400101
CNBP	Cnbp	LOC101408961	MISP	Misp	LOC101392743	STBD1	Stbd1	LOC101407341
CNDP1	Cndp1	LOC101396610	MITD1	Mitd1	LOC101404911	STC1	Stc1	LOC101396215
CNDP2	Cndp2	LOC101396166	MITF	Mitf	LOC101407560	STC2	Stc2	LOC101390061
CNEP1R1	Cnep1r1	LOC101402189	MIXL1	Mixl1	LOC101408766	STEAP1	Steap1	LOC101394476
CNFN	Cnfn	LOC101402649	MKI67	Mki67	LOC101389829	STEAP2	Steap2	LOC101394732
CNGA1	Cnga1	LOC101406421	MKKS	Mkks	LOC101396381	STEAP3	Steap3	LOC101392934
CNGA2	Cnga2	LOC101406269	MKLN1	Mkln1	LOC101403058	STEAP4	Steap4	LOC101393725
CNGA3	Cnga3	LOC101401768	MKNK1	Mknk1	LOC101407388	STIL	Stil	LOC101398808
CNGA4	Cnga4	LOC101395369	MKNK2	Mknk2	LOC101407384	STIM1	Stim1	LOC101407528
CNGB1	Cngb1	LOC101398782	MKRN1	Mkrn1	LOC101387137	STIM2	Stim2	LOC101388785
CNGB3	Cngb3	LOC101393175	MKRN2	Mkrn2	LOC101387959	STIMATE	Tmem110	LOC101407014
CNIH1	Cnih1	LOC101406710	MKRN2OS	Mkrn2os	LOC101392860	STIP1	Stip1	LOC101388694
CNIH2	Cnih2	LOC101393577	MKS1	Mks1	LOC101405433	STK10	Stk10	LOC101393046
CNIH3	Cnih3	LOC101406416	MKX	Mkx	LOC101394630	STK11	Stk11	LOC101389172
CNIH4	Cnih4	LOC101405366	MLANA	Mlana	LOC101393741	STK11IP	Stk11ip	LOC101399645
CNKSR1	Cnksr1	LOC101393706	MLC1	Mlc1	LOC101406065	STK16	Stk16	LOC101394213
CNKSR2	Cnksr2	LOC101404655	MLEC	Mlec	LOC101401745	STK17B	Stk17b	LOC101402519
CNKSR3	Cnksr3	LOC101407558	MLF1	Mlf1	LOC101393101	STK19	Stk19	LOC101389449
CNMD	Lect1	LOC101400094	MLF2	Mlf2	LOC101400640	STK24	Stk24	LOC101399015
CNN1	Cnn1	LOC101397637	MLH1	Mlh1	LOC101398063	STK25	Stk25	LOC101390317
CNN2	Cnn2	LOC101390370	MLH3	Mlh3	LOC101408802	STK26	Stk26	LOC101407638
CNN3	Cnn3	LOC101400571	MLIP	Mlip	LOC101393361	STK3	Stk3	LOC101406304
CNNM1	Cnnm1	LOC101408638	MLKL	Mlkl	LOC101394847	STK31	Stk31	LOC101398716
CNNM2	Cnnm2	LOC101395064	MLLT1	Mllt1	LOC101396248	STK32A	Stk32a	LOC101396676
CNNM3	Cnnm3	LOC101402816	MLLT10	Mllt10	LOC101405061	STK32B	Stk32b	LOC101406044
CNNM4	Cnnm4	LOC101399927	MLLT11	Mllt11	LOC101397605	STK32C	Stk32c	LOC101395968
CNOT1	Cnot1	LOC101399743	MLLT3	Mllt3	LOC101403427	STK33	Stk33	LOC101389407
CNOT10	Cnot10	LOC101392130	MLLT6	Mllt6	LOC101387914	STK35	Stk35	LOC101393990
CNOT11	Cnot11	LOC101408750	MLPH	Mlph	LOC101403759	STK36	Stk36	LOC101388324
CNOT2	Cnot2	LOC101395243	MLST8	Mlst8	LOC101387321	STK38	Stk38	LOC101404175
CNOT3	Cnot3	LOC101407545	MLX	Mlx	LOC101395258	STK38L	Stk38l	LOC101393654
CNOT4	Cnot4	LOC101394730	MLXIP	Mlxip	LOC101407956	STK39	Stk39	LOC101405671
CNOT6	Cnot6	LOC101390911	MLXIPL	Mlxipl	LOC101401717	STK4	Stk4	LOC101389529
CNOT6L	Cnot6l	LOC101388163	MLYCD	Mlycd	LOC101396041	STK40	Stk40	LOC101393366
CNOT7	Cnot7	LOC101395764	MMAA	Mmaa	LOC101396251	STKLD1	Stkld1	LOC101407936
CNOT8	Cnot8	LOC101390566	MMAB	Mmab	LOC101394220	STMN1	Stmn1	LOC101390976
CNOT9	Cnot9	LOC101409153	MMACHC	Mmachc	LOC101395892	STMN2	Stmn2	LOC101407152
CNP	Cnp	LOC101402717	MMADHC	Mmadhc	LOC101401823	STMN3	Stmn3	LOC101392883
CNPPD1	Cnppd1	LOC101391914	MMD	Mmd	LOC101399923	STMN4	Stmn4	LOC101403681
CNPY1	Cnpy1	LOC101406392	MMD2	Mmd2	LOC101393419	STMND1	Stmnd1	LOC101403153
CNPY2	Cnpy2	LOC101403143	MME	Mme	LOC101405725	STN1	Obfc1	LOC101399727
CNPY3	Cnpy3	LOC101388313	MMEL1	Mmel1	LOC101396626	STOM	Stom	LOC101405849
CNPY4	Cnpy4	LOC101390712	MMGT1	Mmgt1	LOC101399957	STOML1	Stoml1	LOC101389079
CNR1	Cnr1	LOC101394450	MMP1	Mmp1a	LOC101408894	STOML2	Stoml2	LOC101407747
CNRIP1	Cnrip1	LOC101406059	MMP10	Mmp10	LOC101409151	STON1	Ston1	LOC106799952
CNST	Cnst	LOC101389923	MMP11	Mmp11	LOC101393608	STON2	Ston2	LOC101399807
CNTD1	Cntd1	LOC101391428	MMP12	Mmp12	LOC101408379	STOX1	Stox1	LOC101387544
CNTF	Cntf	LOC101399840	MMP13	Mmp13	LOC101408118	STOX2	Stox2	LOC101404815
CNTFR	Cntfr	LOC101408473	MMP14	Mmp14	LOC101388804	STPG1	Stpg1	LOC101393205
CNTLN	Cntln	LOC101406827	MMP15	Mmp15	LOC101396991	STPG2	Stpg2	LOC101403148
CNTN1	Cntn1	LOC101402964	MMP16	Mmp16	LOC101392915	STPG3	Stpg3	LOC101392776

CNTN2	Cntn2	LOC101400773	MMP17	Mmp17	LOC101406207	STPG4	1700011E24R	LOC101395523
CNTN3	Cntn3	LOC101402418	MMP19	Mmp19	LOC101394822	STRA6	Stra6	LOC101390631
CNTN4	Cntn4	LOC101389942	MMP2	Mmp2	LOC101406803	STRA8	Stra8	LOC101396807
CNTN5	Cntn5	LOC101391575	MMP20	Mmp20	LOC101387803	STRADA	Strada	LOC101390073
CNTNAP1	Cntnap1	LOC101393822	MMP21	Mmp21	LOC101400734	STRADB	Stradb	LOC101390400
CNTNAP2	Cntnap2	LOC101395071	MMP24	Mmp24	LOC101395936	STRAP	Strap	LOC101400986
CNTNAP4	Cntnap4	LOC101403165	MMP25	Mmp25	LOC101402732	STRBP	Strbp	LOC101397909
CNTNAP5	Cntnap5a	LOC101397825	MMP27	Mmp27	LOC101387548	STRC	Strc	LOC101408872
CNTRL	Cntrl	LOC101404698	MMP28	Mmp28	LOC101403243	STRIP1	Strip1	LOC101397298
CNTROB	Cntrob	LOC101396817	MMP3	Mmp3	LOC101408634	STRIP2	Strip2	LOC101391362
COA3	Coa3	LOC101391673	MMP7	Mmp7	LOC101391913	STRN	Strn	LOC101407983
COA4	Coa4	LOC101395516	MMP8	Mmp8	LOC101387295	STRN3	Strn3	LOC101405644
COA5	Coa5	LOC101403162	MMP9	Mmp9	LOC101402187	STRN4	Strn4	LOC101394509
COA6	Coa6	LOC101391177	MMRN1	Mmrn1	LOC101409165	STT3A	Stt3a	LOC101396926
COA7	Coa7	LOC101387407	MMRN2	Mmrn2	LOC101406729	STT3B	Stt3b	LOC101390447
COA8	Apopt1	LOC101397431	MMS19	Mms19	LOC101391067	STUB1	Stub1	LOC101401774
COASY	Coasy	LOC101396120	MMS22L	Mms22l	LOC101402419	STUM	Stum	LOC101387416
COBL	Cobl	LOC101403961	MMUT	Mut	LOC101388148	STX12	Stx12	LOC101404802
COBLL1	Cobll1	LOC101403921	MN1	Mn1	LOC101388159	STX16	Stx16	LOC101404459
COCH	Coch	LOC101406361	MNAT1	Mnat1	LOC101395754	STX17	Stx17	LOC101392772
COG1	Cog1	LOC101406212	MND1	Mnd1	LOC101406093	STX18	Stx18	LOC101406806
COG2	Cog2	LOC101395922	MNDA	Ifi205	LOC101400052	STX19	Stx19	LOC101401532
COG3	Cog3	LOC101408314	MNS1	Mns1	LOC101392496	STX1A	Stx1a	LOC101400933
COG4	Cog4	LOC101391248	MNT	Mnt	LOC101398366	STX1B	Stx1b	LOC101403610
COG5	Cog5	LOC101397798	MOB1A	Mob1a	LOC101407433	STX2	Stx2	LOC101405679
COG6	Cog6	LOC101393778	MOB1B	Mob1b	LOC101390019	STX3	Stx3	LOC101406667
COG7	Cog7	LOC101388302	MOB2	Mob2	LOC101389842	STX4	Stx4a	LOC101403869
COG8	Cog8	LOC101403151	MOB3A	Mob3a	LOC101407121	STX5	Stx5a	LOC101403431
COIL	Coil	LOC101402456	MOB3B	Mob3b	LOC106801526	STX6	Stx6	LOC101407488
COL10A1	Col10a1	LOC101403892	MOB3C	Mob3c	LOC101402931	STX7	Stx7	LOC101402576
COL11A1	Col11a1	LOC101389847	MOCOS	Mocos	LOC101401557	STX8	Stx8	LOC101387734
COL11A2	Col11a2	LOC101401307	MOCS1	Mocs1	LOC101397237	STXBP1	Stxbp1	LOC101387530
COL12A1	Col12a1	LOC101400853	MOCS2	Mocs2	LOC101397228	STXBP2	Stxbp2	LOC101409134
COL13A1	Col13a1	LOC101404549	MOCS3	Mocs3	LOC101391411	STXBP3	Stxbp3	LOC101408261
COL14A1	Col14a1	LOC101390572	MOG	Mog	LOC101400663	STXBP4	Stxbp4	LOC101399404
COL15A1	Col15a1	LOC101391304	MOGAT1	Mogat1	LOC101402435	STXBP5	Stxbp5	LOC101389754
COL16A1	Col16a1	LOC101395232	MOGAT2	Mogat2	LOC101403333	STXBP5L	Stxbp5l	LOC101400810
COL17A1	Col17a1	LOC101389600	MOGS	Mogs	LOC101398886	STXBP6	Stxbp6	LOC101387345
COL18A1	Col18a1	LOC101408904	MOK	Mok	LOC101389726	STYK1	Styk1	LOC101389137
COL19A1	Col19a1	LOC101396172	MON1A	Mon1a	LOC101408517	STYX	Styx	LOC101403658
COL1A1	Col1a1	LOC101408228	MON1B	Mon1b	LOC101402727	STYXL1	Styxl1	LOC101405128
COL1A2	Col1a2	LOC101402610	MON2	Mon2	LOC101405241	SUB1	Sub1	LOC101396781
COL20A1	Col20a1	LOC101402794	MORC1	Morc1	LOC101405891	SUCLA2	Sucla2	LOC101389291
COL22A1	Col22a1	LOC101387643	MORC3	Morc3	LOC101399036	SUCLG1	Suclg1	LOC101408060
COL23A1	Col23a1	LOC101390323	MORC4	Morc4	LOC101388022	SUCLG2	Suclg2	LOC101405120
COL24A1	Col24a1	LOC101390994	MORF4L1	Morf4l1	LOC101408787	SUCO	Suco	LOC101401211
COL25A1	Col25a1	LOC101401735	MORF4L2	Morf4l2	LOC101408106	SUDS3	Suds3	LOC101407694
COL26A1	Col26a1	LOC101393856	MORN1	Morn1	LOC101397914	SUFU	Sufu	LOC101392685
COL27A1	Col27a1	LOC101398846	MORN2	Morn2	LOC101407895	SUGCT	Sugct	LOC101395552
COL28A1	Col28a1	LOC101387140	MORN3	Morn3	LOC101400964	SUGP1	Sugp1	LOC101402769
COL2A1	Col2a1	LOC101391322	MORN4	Morn4	LOC101392614	SUGP2	Sugp2	LOC101405481
COL3A1	Col3a1	LOC101395059	MORN5	Morn5	LOC101407226	SULF1	Sulf1	LOC101395350
COL4A1	Col4a1	LOC101387192	MOS	Mos	LOC101404738	SULF2	Sulf2	LOC101397166
COL4A2	Col4a2	LOC101401300	MOSPD1	Mospd1	LOC101402380	SULT1B1	Sult1b1	LOC101408338

COL4A3	Col4a3	LOC101400954	MOSPD2	Mospd2	LOC101393152	SULT1C2	Sult1c2	LOC101393073
COL4A4	Col4a4	LOC101406715	MOSPD3	Mospd3	LOC101395650	SULT1E1	Sult1e1	LOC101408857
COL4A5	Col4a5	LOC101393591	MOV10	Mov10	LOC101388032	SULT2A1	Sult2a6	LOC101388109
COL4A6	Col4a6	LOC101392840	MOV10L1	Mov10l1	LOC101400818	SULT2B1	Sult2b1	LOC101392478
COL5A1	Col5a1	LOC101408975	MOXD1	Moxd1	LOC101406770	SULT4A1	Sult4a1	LOC101397529
COL5A2	Col5a2	LOC101395323	MPC1	Mpc1	LOC101390364	SULT6B1	Sult6b1	LOC106800553
COL5A3	Col5a3	LOC101406257	MPC2	Mpc2	LOC101398757	SUMF1	Sumf1	LOC101391294
COL6A1	Col6a1	LOC101387638	MPDU1	Mpdu1	LOC101400888	SUMF2	Sumf2	LOC101389236
COL6A2	Col6a2	LOC101387896	MPDZ	Mpdz	LOC101389047	SUMO1	Sumo1	LOC101392680
COL6A3	Col6a3	LOC101403137	MPEG1	Mpeg1	LOC101401692	SUMO2	Sumo2	LOC101392098
COL6A5	Col6a5	LOC101396405	MPG	Mpg	LOC101402302	SUMO3	Sumo3	LOC101407158
COL6A6	Col6a6	LOC101394352	MPHOSPH10	Mphosph10	LOC101387464	SUN1	Sun1	LOC101408080
COL7A1	Col7a1	LOC101392400	MPHOSPH6	Mphosph6	LOC101397193	SUN2	Sun2	LOC101395527
COL8A2	Col8a2	LOC101391400	MPHOSPH8	Mphosph8	LOC106802631	SUN3	Sun3	LOC101395100
COL9A1	Col9a1	LOC101395911	MPHOSPH9	Mphosph9	LOC101391241	SUN5	Sun5	LOC101408526
COL9A2	Col9a2	LOC101407944	MPI	Mpi	LOC101395646	SUOX	Suox	LOC101397253
COL9A3	Col9a3	LOC101397505	MPL	Mpl	LOC101405206	SUPT16H	Supt16	LOC101397816
COLCA2	Gm684	LOC101398185	MPLKIP	Mplkip	LOC101395810	SUPT20H	Supt20	LOC101399926
COLEC10	Colec10	LOC101404203	MPND	Mpnd	LOC101399090	SUPT3H	Supt3	LOC101393701
COLEC11	Colec11	LOC106802826	MPO	Mpo	LOC101406472	SUPT4H1	Supt4a	LOC101407357
COLEC12	Colec12	LOC101407998	MPP1	Mpp1	LOC101402330	SUPT5H	Supt5	LOC101388984
COLGALT1	Colgalt1	LOC101401384	MPP2	Mpp2	LOC101388850	SUPT6H	Supt6	LOC101395165
COLGALT2	Colgalt2	LOC101392605	MPP3	Mpp3	LOC101388340	SUPT7L	Supt7l	LOC101399074
COLQ	Colq	LOC101397621	MPP4	Mpp4	LOC101391234	SUPV3L1	Supv3l1	LOC101407151
COMMD1	Commd1	LOC101392895	MPP5	Mpp5	LOC101406120	SURF1	Surf1	LOC101407401
COMMD10	Commd10	LOC101396678	MPP6	Mpp6	LOC101398060	SURF2	Surf2	LOC101407679
COMMD2	Commd2	LOC101388887	MPP7	Mpp7	LOC101394378	SURF4	Surf4	LOC101397148
COMMD3	Commd3	LOC106799938	MPPE1	Mppe1	LOC101397301	SURF6	Surf6	LOC101396170
COMMD4	Commd4	LOC101397384	MPPED1	Mpped1	LOC101397275	SUSD1	Susd1	LOC101407483
COMMD5	Commd5	LOC101407312	MPPED2	Mpped2	LOC101400998	SUSD2	Susd2	LOC101390549
COMMD6	Commd6	LOC101387619	MPRIP	Mprip	LOC101402538	SUSD3	Susd3	LOC101401459
COMMD7	Commd7	LOC106803883	MPST	Mpst	LOC101398055	SUSD4	Susd4	LOC101403611
COMMD8	Commd8	LOC101405193	MPV17	Mpv17	LOC101404753	SUSD5	Susd5	LOC101398989
COMMD9	Commd9	LOC101387401	MPV17L	Mpv17l	LOC101400309	SUSD6	Susd6	LOC101391569
COMP	Comp	LOC101407588	MPV17L2	Mpv17l2	LOC101400056	SUV39H1	Suv39h1	LOC101389632
COMT	Comt	LOC101393512	MPZ	Mpz	LOC101399457	SUV39H2	Suv39h2	LOC101392076
COMTD1	Comtd1	LOC101403756	MPZL2	Mpzl2	LOC101399469	SUZ12	Suz12	LOC101389322
COP1	Rfwd2	LOC101394544	MPZL3	Mpzl3	LOC101399725	SV2A	Sv2a	LOC101387482
COPA	Copa	LOC101392924	MR1	Mr1	LOC101406881	SV2B	Sv2b	LOC101396811
COPB1	Copb1	LOC101401873	MRAP	Mrap	LOC101403836	SV2C	Sv2c	LOC101390108
COPB2	Copb2	LOC101401360	MRAP2	Mrap2	LOC101388218	SVBP	Svbp	LOC101406000
COPE	Cope	LOC101406438	MRAS	Mras	LOC101404332	SVEP1	Svep1	LOC101406963
COPG1	Copg1	LOC101408708	MRC1	Mrc1	LOC101387281	SVOP	Svop	LOC101394393
COPG2	Copg2	LOC101404296	MRC2	Mrc2	LOC101408826	SVOPL	Svopl	LOC101390990
COPRS	Coprs	LOC101389849	MRE11	Mre11a	LOC101396533	SWAP70	Swap70	LOC101393844
COPS2	Cops2	LOC101401989	MREG	Mreg	LOC101402960	SWI5	Swi5	LOC101402684
COPS3	Cops3	LOC101389219	MRFAP1	Mrfap1	LOC101403846	SWSAP1	Swsap1	LOC101394704
COPS4	Cops4	LOC101395598	MRGBP	Mrgbp	LOC101404367	SWT1	Swt1	LOC101397049
COPS5	Cops5	LOC101393235	MRGPRD	Mrgprd	LOC101392133	SYAP1	Syap1	LOC101398371
COPS6	Cops6	LOC101389331	MRGPRF	Mrgprf	LOC101403011	SYBU	Sybu	LOC101399396
COPS7A	Cops7a	LOC101401264	MRI1	Mri1	LOC106803898	SYCE1	Syce1	LOC101392645
COPS7B	Cops7b	LOC101392684	MRLN	Mrln	LOC106801372	SYCE1L	Syce1l	LOC101393327
COPS8	Cops8	LOC101402868	MRM1	Mrm1	LOC101397434	SYCP1	Sycp1	LOC101401715
COPS9	Myeov2	LOC101408295	MRM2	Mrm2	LOC101406495	SYCP2	Sycp2	LOC101401227

COPZ1	Copz1	LOC101397061	MRM3	Rnmtl1	LOC101407703	SYCP2L	Sycp2l	LOC101404983
COPZ2	Copz2	LOC101393472	MRNIP	3010026O09I	LOC101387208	SYCP3	Sycp3	LOC101401088
COQ10A	Coq10a	LOC101402094	MRO	Mro	LOC101395478	SYDE1	Syde1	LOC101394038
COQ10B	Coq10b	LOC101404447	MROH1	Mroh1	LOC101390959	SYDE2	Syde2	LOC101389120
COQ2	Coq2	LOC101396903	MROH2A	Mroh2a	LOC101399984	SYF2	Syf2	LOC101388404
COQ3	Coq3	LOC101403469	MROH2B	Mroh2b	LOC101388221	SYK	Syk	LOC101404022
COQ4	Coq4	LOC101395485	MROH6	Mroh6	LOC101406177	SYMPK	Sympk	LOC101401095
COQ5	Coq5	LOC101402968	MROH7	Mroh7	LOC101396047	SYN1	Syn1	LOC101397942
COQ6	Coq6	LOC101403221	MROH8	Mroh8	LOC101398021	SYN2	Syn2	LOC101389582
COQ7	Coq7	LOC101392229	MROH9	Mroh9	LOC101396705	SYNC	Sync	LOC101402955
COQ8A	Adck3	LOC101388362	MRPL1	Mrpl1	LOC101388414	SYNCRIP	Syncrip	LOC101390036
COQ8B	Adck4	LOC101396945	MRPL10	Mrpl10	LOC101391256	SYNDIG1	Syndig1	LOC101395990
COQ9	Coq9	LOC101393728	MRPL11	Mrpl11	LOC101395873	SYNDIG1L	Syndig1l	LOC101405495
CORIN	Corin	LOC101405710	MRPL12	Mrpl12	LOC101396815	SYNE1	Syne1	LOC101390701
CORO1A	Coro1a	LOC101387937	MRPL13	Mrpl13	LOC101402282	SYNE2	Syne2	LOC101399981
CORO1B	Coro1b	LOC101406227	MRPL14	Mrpl14	LOC101397747	SYNE3	Syne3	LOC101408663
CORO1C	Coro1c	LOC101393716	MRPL15	Mrpl15	LOC101390509	SYNE4	Syne4	LOC101394098
CORO2A	Coro2a	LOC101389687	MRPL16	Mrpl16	LOC101406924	SYNGAP1	Syngap1	LOC101395488
CORO2B	Coro2b	LOC101400414	MRPL17	Mrpl17	LOC101402223	SYNGR1	Syngr1	LOC101390518
CORO6	Coro6	LOC101388588	MRPL18	Mrpl18	LOC101408597	SYNGR2	Syngr2	LOC106800160
CORT	Cort	LOC106799991	MRPL19	Mrpl19	LOC101404838	SYNGR3	Syngr3	LOC101389549
COTL1	Cotl1	LOC101393478	MRPL2	Mrpl2	LOC101406965	SYNGR4	Syngr4	LOC101391025
COX10	Cox10	LOC101404300	MRPL20	Mrpl20	LOC101391311	SYNJ1	Synj1	LOC101404637
COX11	Cox11	LOC101398879	MRPL21	Mrpl21	LOC101403269	SYNJ2	Synj2	LOC101388114
COX14	Cox14	LOC101387130	MRPL22	Mrpl22	LOC101389881	SYNJ2BP	Synj2bp	LOC106800001
COX15	Cox15	LOC101397496	MRPL23	Mrpl23	LOC101399088	SYNM	Synm	LOC101393815
COX16	Cox16	LOC101393206	MRPL24	Mrpl24	LOC101388773	SYNPO2	Synpo2	LOC101390979
COX17	Cox17	LOC101404121	MRPL27	Mrpl27	LOC101408664	SYNPO2L	Synpo2l	LOC101390822
COX18	Cox18	LOC101402745	MRPL28	Mrpl28	LOC101400993	SYNPR	Synpr	LOC101400922
COX19	Cox19	LOC101407823	MRPL3	Mrpl3	LOC101393850	SYNRG	Synrg	LOC101399497
COX20	Cox20	LOC101395972	MRPL30	Mrpl30	LOC101405181	SYP	Syp	LOC101403856
COX4I1	Cox4i1	LOC101391098	MRPL32	Mrpl32	LOC101407539	SYPL2	Sypl2	LOC101403804
COX4I2	Cox4i2	LOC101392592	MRPL33	Mrpl33	LOC101398386	SYT1	Syt1	LOC101394333
COX5A	Cox5a	LOC101396341	MRPL34	Mrpl34	LOC101396349	SYT10	Syt10	LOC101401887
COX5B	Cox5b	LOC101400987	MRPL35	Mrpl35	LOC101392386	SYT11	Syt11	LOC101398377
COX6A1	Cox6a1	LOC101404457	MRPL36	Mrpl36	LOC101391024	SYT12	Syt12	LOC101406831
COX6A2	Cox6a2	LOC101407449	MRPL37	Mrpl37	LOC101394004	SYT13	Syt13	LOC101392817
COX6B1	Cox6b1	LOC101396668	MRPL38	Mrpl38	LOC101399309	SYT14	Syt14	LOC101405661
COX6C	Cox6c	LOC101407614	MRPL39	Mrpl39	LOC101390490	SYT16	Syt16	LOC101397491
COX7A1	Cox7a1	LOC101401876	MRPL4	Mrpl4	LOC101408613	SYT17	Syt17	LOC101392747
COX7A2	Cox7a2	LOC101401382	MRPL40	Mrpl40	LOC101396602	SYT2	Syt2	LOC101396837
COX7A2L	Cox7a2l	LOC101405001	MRPL41	Mrpl41	LOC101394974	SYT3	Syt3	LOC101392838
COX7B	Cox7b	LOC101387098	MRPL42	Mrpl42	LOC101407645	SYT4	Syt4	LOC101404941
COX7C	Cox7c	LOC101398568	MRPL43	Mrpl43	LOC101403661	SYT5	Syt5	LOC101390523
COX8A	Cox8a	LOC101387666	MRPL44	Mrpl44	LOC101404355	SYT6	Syt6	LOC101405035
CP	Cp	LOC101390699	MRPL46	Mrpl46	LOC101398783	SYT7	Syt7	LOC101394584
CPA1	Cpa1	LOC101388019	MRPL47	Mrpl47	LOC101405593	SYT8	Syt8	LOC101389843
CPA2	Cpa2	LOC101398392	MRPL48	Mrpl48	LOC101395256	SYT9	Syt9	LOC101392210
CPA3	Cpa3	LOC101391784	MRPL49	Mrpl49	LOC101402468	SYTL1	Sytl1	LOC101400602
CPA4	Cpa4	LOC101388704	MRPL50	Mrpl50	LOC101396090	SYTL2	Sytl2	LOC101393319
CPA5	Cpa5	LOC101388280	MRPL51	Mrpl51	LOC101406230	SYTL3	Sytl3	LOC101405813
CPA6	Cpa6	LOC101394321	MRPL52	Mrpl52	LOC101389076	SYTL4	Sytl4	LOC101403186
CPB1	Cpb1	LOC101392043	MRPL53	Mrpl53	LOC101399507	SYTL5	Sytl5	LOC101388596
CPB2	Cpb2	LOC101387228	MRPL54	Mrpl54	LOC101398322	SYVN1	Syvn1	LOC101402212

CPD	Cpd	LOC101398534	MRPL57	Mrpl57	LOC101401511	SZRD1	Szrd1	LOC101403044
CPE	Cpe	LOC101390076	MRPL9	Mrpl9	LOC101406574	SZT2	Szt2	LOC101406948
CPEB1	Cpeb1	LOC101390151	MRPS10	Mrps10	LOC101400861	TAAR1	Taar1	LOC101403112
CPEB2	Cpeb2	LOC101406138	MRPS11	Mrps11	LOC101399043	TAAR2	Taar2	LOC101408101
CPEB3	Cpeb3	LOC101394467	MRPS12	Mrps12	LOC101407462	TAAR5	Taar5	LOC101402847
CPEB4	Cpeb4	LOC101389362	MRPS14	Mrps14	LOC101395747	TAAR6	Taar6	LOC101407313
CPED1	Cped1	LOC101407111	MRPS15	Mrps15	LOC101394133	TAAR9	Taar9	LOC101407039
CPLX1	Cplx1	LOC101398527	MRPS16	Mrps16	LOC101392875	TAB1	Tab1	LOC101397274
CPLX2	Cplx2	LOC101387890	MRPS18A	Mrps18a	LOC101398417	TAB2	Tab2	LOC101388792
CPLX3	Cplx3	LOC101394879	MRPS18B	Mrps18b	LOC101387122	TAB3	Tab3	LOC101394159
CPLX4	Cplx4	LOC101406956	MRPS18C	Mrps18c	LOC101397420	TAC1	Tac1	LOC101407519
CPM	Cpm	LOC101392088	MRPS2	Mrps2	LOC101387623	TAC4	Tac4	LOC101405618
CPN1	Cpn1	LOC101398946	MRPS21	Mrps21	LOC101391009	TACC1	Tacc1	LOC101394217
CPNE1	Cpne1	LOC101391412	MRPS22	Mrps22	LOC101401971	TACC2	Tacc2	LOC101401052
CPNE2	Cpne2	LOC101392443	MRPS23	Mrps23	LOC101403685	TACC3	Tacc3	LOC101394307
CPNE3	Cpne3	LOC101397718	MRPS24	Mrps24	LOC106803105	TACO1	Taco1	LOC101389287
CPNE4	Cpne4	LOC101393255	MRPS25	Mrps25	LOC101395994	TACR1	Tacr1	LOC101404314
CPNE5	Cpne5	LOC101403128	MRPS26	Mrps26	LOC101389031	TACR2	Tacr2	LOC101406384
CPNE6	Cpne6	LOC101399351	MRPS27	Mrps27	LOC101395201	TACR3	Tacr3	LOC101394381
CPNE7	Cpne7	LOC101388185	MRPS28	Mrps28	LOC101407688	TACSTD2	Tacstd2	LOC101399593
CPNE8	Cpne8	LOC101401366	MRPS30	Mrps30	LOC101389511	TADA1	Tada1	LOC101390394
CPNE9	Cpne9	LOC101392930	MRPS33	Mrps33	LOC101407957	TADA2A	Tada2a	LOC101398795
CPOX	Cpox	LOC101405111	MRPS34	Mrps34	LOC106803075	TADA2B	Tada2b	LOC101403506
CPPED1	Cpped1	LOC101404490	MRPS35	Mrps35	LOC101394928	TADA3	Tada3	LOC101391143
CPQ	Cpq	LOC101403319	MRPS36	Mrps36	LOC101397480	TAF1	Taf1	LOC101402393
CPS1	Cps1	LOC101398943	MRPS5	Mrps5	LOC101395261	TAF10	Taf10	LOC101389925
CPSF1	Cpsf1	LOC101389333	MRPS7	Mrps7	LOC101393385	TAF11	Taf11	LOC101390224
CPSF2	Cpsf2	LOC101400267	MRPS9	Mrps9	LOC101389910	TAF12	Taf12	LOC101387794
CPSF3	Cpsf3	LOC101404221	MRRF	Mrrf	LOC101408720	TAF13	Taf13	LOC101406688
CPSF4	Cpsf4	LOC101405119	MRS2	Mrs2	LOC101396291	TAF15	Taf15	LOC101402983
CPSF4L	Cpsf4l	LOC101392447	MRTFA	Mkl1	LOC101400816	TAF1B	Taf1b	LOC101394578
CPSF6	Cpsf6	LOC101392358	MRTFB	Mkl2	LOC101398327	TAF1C	Taf1c	LOC101394496
CPSF7	Cpsf7	LOC101391679	MRTO4	Mrto4	LOC101408888	TAF2	Taf2	LOC101403059
CPT1A	Cpt1a	LOC101403536	MRVI1	Mrvi1	LOC101395713	TAF3	Taf3	LOC101399378
CPT1B	Cpt1b	LOC101387243	MS4A1	Ms4a1	LOC101408421	TAF4	Taf4	LOC101405680
CPT1C	Cpt1c	LOC101408252	MS4A12	Ms4a12	LOC101392564	TAF4B	Taf4b	LOC101396150
CPT2	Cpt2	LOC101389483	MS4A13	Ms4a13	LOC101392819	TAF5	Taf5	LOC101396892
CPTP	Cptp	LOC101390559	MS4A14	Ms4a14	LOC101392028	TAF5L	Taf5l	LOC101395143
CPVL	Cpvl	LOC101389491	MS4A15	Ms4a15	LOC101408929	TAF6	Taf6	LOC101389857
CPXM1	Cpxm1	LOC101391425	MS4A2	Ms4a2	LOC101407720	TAF6L	Taf6l	LOC101398974
CPXM2	Cpxm2	LOC101403439	MS4A3	Ms4a3	LOC101390516	TAF7	Taf7	LOC101387256
CPZ	Cpz	LOC101400709	MS4A5	Ms4a5	LOC101392302	TAF7L	Taf7l	LOC101406943
CR2	Cr2	LOC106800998	MS4A6A	Ms4a6b	LOC101390772	TAF8	Taf8	LOC101391394
CRABP1	Crabp1	LOC101406433	MS4A7	Ms4a7	LOC101407978	TAF9	Taf9	TAF9
CRABP2	Crabp2	LOC101387236	MS4A8	Ms4a8a	LOC101408672	TAF9B	Taf9b	LOC101387859
CRACR2A	Cracr2a	LOC101390437	MSANTD1	Msantd1	LOC101401154	TAFA1	Fam19a1	LOC101405374
CRACR2B	Cracr2b	LOC101391880	MSANTD2	Msantd2	LOC101394420	TAFA2	Fam19a2	LOC101404362
CRAMP1	Cramp1l	LOC101394247	MSANTD3	Msantd3	LOC106799929	TAFA3	Fam19a3	LOC101387096
CRAT	Crat	LOC101404172	MSANTD4	Msantd4	LOC101405234	TAFA4	Fam19a4	LOC101391699
CRB1	Crb1	LOC101406672	MSC	Msc	LOC101398132	TAFA5	Fam19a5	LOC101400021
CRB2	Crb2	LOC101401652	MSGN1	Msgn1	LOC101402824	TAGAP	Tagap	LOC101406682
CRB3	Crb3	LOC101404675	MSH2	Msh2	LOC101394579	TAGLN	Tagln	LOC101404286
CRBN	Crbn	LOC101390628	MSH3	Msh3	LOC101403458	TAGLN2	Tagln2	LOC101403468
CRCP	Crcp	LOC101391614	MSH4	Msh4	LOC101400174	TAGLN3	Tagln3	LOC101393476

CREB1	Creb1	LOC101401919	MSH5	Msh5	LOC101393961	TAL1	Tal1	LOC101401618
CREB3	Creb3	LOC101389181	MSH6	Msh6	LOC101393912	TAL2	Tal2	LOC101405744
CREB3L1	Creb3l1	LOC101395009	MSI1	Msi1	LOC101395246	TALDO1	Taldo1	LOC101408600
CREB3L2	Creb3l2	LOC101392624	MSI2	Msi2	LOC101403242	TAMM41	Tamm41	LOC101390123
CREB3L3	Creb3l3	LOC101395550	MSL1	Msl1	LOC101397087	TANC1	Tanc1	LOC101397411
CREB3L4	Creb3l4	LOC101397522	MSL2	Msl2	LOC101408943	TANC2	Tanc2	LOC101387222
CREB5	Creb5	LOC101390017	MSL3	Msl3	LOC101387150	TANGO2	Tango2	LOC101393010
CREBBP	Crebbp	LOC101397953	MSLN	Msln	LOC101400193	TANGO6	Tango6	LOC101401495
CREBL2	Crebl2	LOC101393570	MSMB	Msmb	LOC101404646	TANK	Tank	LOC101393632
CREBRF	Crebrf	LOC101391488	MSMO1	Msmo1	LOC101405520	TAOK1	Taok1	LOC101389623
CREBZF	Crebzf	LOC101392807	MSMP	Msmp	LOC101389677	TAOK2	Taok2	LOC101391359
CREG1	Creg1	LOC101399549	MSN	Msn	LOC101393930	TAOK3	Taok3	LOC101407955
CREG2	Creg2	LOC101387157	MSR1	Msr1	LOC101396720	TAP1	Tap1	LOC101403297
CRELD1	Creld1	LOC101389679	MSRA	Msra	LOC101396471	TAP2	Tap2	LOC101403822
CRELD2	Creld2	LOC101405795	MSRB1	Msrb1	LOC101391103	TAPBP	Tapbp	LOC101408979
CREM	Crem	LOC101406529	MSRB2	Msrb2	LOC101396968	TAPBPL	Tapbpl	LOC101406741
CRH	Crh	LOC101399586	MSRB3	Msrb3	LOC101408556	TAPT1	Tapt1	LOC101406905
CRHBP	Crhbp	LOC101388797	MSS51	Mss51	LOC101391731	TARBP2	Tarbp2	LOC101391071
CRHR1	Crhr1	LOC101403325	MST1	Mst1	LOC101404505	TARDBP	Tardbp	LOC101390310
CRHR2	Crhr2	LOC101408167	MST1R	Mst1r	LOC101408437	TARS2	Tars2	LOC101392111
CRIM1	Crim1	LOC101409098	MSTN	Mstn	LOC101397915	TAS1R1	Tas1r1	LOC101402513
CRIP1	Crip1	LOC101406815	MSX1	Msx1	LOC101406555	TAS1R2	Tas1r2	LOC101407492
CRIP2	Crip2	LOC101392286	MSX2	Msx2	LOC101388837	TAS1R3	Tas1r3	LOC101394547
CRIP3	Crip3	LOC101403563	MT2A	Mt2	LOC101389715	TAS2R1	Tas2r119	LOC101394255
CRIPT	Cript	LOC101397271	MTA1	Mta1	LOC101406054	TAS2R16	Tas2r118	LOC101403529
CRISP2	Crisp2	LOC101387625	MTA2	Mta2	LOC101398212	TAS2R3	Tas2r137	LOC101406646
CRISPLD1	Crispld1	LOC101403517	MTA3	Mta3	LOC101388776	TAS2R38	Tas2r138	LOC101391925
CRISPLD2	Crispld2	LOC101392641	MTAP	Mtap	LOC101400785	TAS2R39	Tas2r139	LOC101399481
CRK	Crk	LOC101404903	MTBP	Mtbp	LOC101390328	TAS2R4	Tas2r108	LOC101393216
CRKL	Crkl	LOC101401283	MTCH1	Mtch1	LOC101401824	TAS2R40	Tas2r144	LOC101402530
CRLF1	Crlf1	LOC101408792	MTCH2	Mtch2	LOC101408058	TAS2R42	Tas2r131	LOC101402986
CRLF3	Crlf3	LOC101389074	MTCL1	Mtcl1	LOC101398733	TAS2R60	Tas2r135	LOC101397674
CRLS1	Crls1	LOC101400533	MTCP1	Mtcp1	LOC101403471	TAS2R7	Tas2r130	LOC101389816
CRMP1	Crmp1	LOC101405169	MTDH	Mtdh	LOC101403842	TASOR	Fam208a	LOC101390283
CRNKL1	Crnkl1	LOC101407883	MTERF3	Mterf3	LOC101402532	TASOR2	Fam208b	LOC101403656
CRNN	Crnn	LOC101387572	MTERF4	Mterf4	LOC101389014	TASP1	Tasp1	LOC101394742
CROCC	Crocc	LOC101388319	MTF1	Mtf1	LOC101398672	TAT	Tat	LOC101388337
CROCC2	Crocc2	LOC101388748	MTF2	Mtf2	LOC101406503	TATDN1	Tatdn1	LOC101396111
CROT	Crot	LOC101390414	MTFMT	Mtfmt	LOC101388992	TATDN2	Tatdn2	LOC101392246
CRPPA	Ispd	LOC101402361	MTFP1	Mtfp1	LOC101397165	TATDN3	Tatdn3	LOC101391059
CRTAC1	Crtac1	LOC101394906	MTFR1	Mtfr1	LOC101397861	TAX1BP1	Tax1bp1	LOC101390868
CRTAM	Crtam	LOC101404808	MTFR1L	Mtfr1l	LOC101390311	TAX1BP3	Tax1bp3	LOC101390334
CRTAP	Crtap	LOC101393757	MTFR2	Mtfr2	LOC101387525	TAZ	Taz	LOC101393868
CRTC1	Crtc1	LOC101407848	MTG1	Mtg1	LOC101409096	TBATA	Tbata	LOC101392263
CRTC2	Crtc2	LOC101396831	MTG2	Mtg2	LOC101400171	TBC1D1	Tbc1d1	LOC101390782
CRTC3	Crtc3	LOC101401678	MTHFD1	Mthfd1	LOC101400866	TBC1D10A	Tbc1d10a	LOC101390410
CRX	Crx	LOC101387687	MTHFD1L	Mthfd1l	LOC101390192	TBC1D10B	Tbc1d10b	LOC101400203
CRY1	Cry1	LOC101389155	MTHFD2	Mthfd2	LOC101395354	TBC1D10C	Tbc1d10c	LOC101405703
CRY2	Cry2	LOC101395443	MTHFD2L	Mthfd2l	LOC101394683	TBC1D12	Tbc1d12	LOC101401666
CRYAA	Cryaa	LOC101400962	MTHFR	Mthfr	LOC101394209	TBC1D13	Tbc1d13	LOC101400857
CRYAB	Cryab	LOC101395237	MTHFS	Mthfs	LOC101393951	TBC1D14	Tbc1d14	LOC101402799
CRYBA1	Cryba1	LOC101390580	MTHFSD	Mthfsd	LOC101391013	TBC1D15	Tbc1d15	LOC101387673
CRYBA2	Cryba2	LOC101390740	MTIF2	Mtif2	LOC101401607	TBC1D16	Tbc1d16	LOC101391666
CRYBA4	Cryba4	LOC101391409	MTIF3	Mtif3	LOC101408748	TBC1D17	Tbc1d17	LOC101388533

CRYBB1	Crybb1	LOC101388411	MTM1	Mtm1	LOC101397388	TBC1D19	Tbc1d19	LOC101388529
CRYBB2	Crybb2	LOC101389979	MTMR1	Mtmr1	LOC101397136	TBC1D2	Tbc1d2	LOC101390215
CRYBB3	Crybb3	LOC101390237	MTMR10	Mtmr10	LOC101409071	TBC1D20	Tbc1d20	LOC101398199
CRYBG1	Aim1	LOC101387699	MTMR11	Mtmr11	LOC101388006	TBC1D21	Tbc1d21	LOC101388040
CRYBG2	Aim1l	LOC101395495	MTMR12	Mtmr12	LOC101396082	TBC1D22A	Tbc1d22a	LOC101404318
CRYBG3	Crybg3	LOC101390695	MTMR14	Mtmr14	LOC101393190	TBC1D22B	Tbc1d22b	LOC101401035
CRYGB	Crygb	LOC101403569	MTMR2	Mtmr2	LOC101393043	TBC1D23	Tbc1d23	LOC101407729
CRYGC	Crygc	LOC101403302	MTMR3	Mtmr3	LOC101399651	TBC1D24	Tbc1d24	LOC101407273
CRYGN	Crygn	LOC101405422	MTMR4	Mtmr4	LOC101407889	TBC1D25	Tbc1d25	LOC101390841
CRYGS	Crygs	LOC101405487	MTMR6	Mtmr6	LOC101405960	TBC1D2B	Tbc1d2b	LOC101393694
CRYL1	Cryl1	LOC101404307	MTMR7	Mtmr7	LOC101395067	TBC1D30	Tbc1d30	LOC101408903
CRYM	Crym	LOC101396607	MTMR9	Mtmr9	LOC101394659	TBC1D31	Tbc1d31	LOC101400444
CRYZ	Cryz	LOC101398524	MTNR1A	Mtnr1a	LOC101391490	TBC1D32	Tbc1d32	LOC101389508
CRYZL1	Cryzl1	LOC101407251	MTNR1B	Mtnr1b	LOC101399557	TBC1D4	Tbc1d4	LOC101408716
CS	Cs	LOC101402352	MTO1	Mto1	LOC101399447	TBC1D5	Tbc1d5	LOC101401361
CSAD	Csad	LOC101407156	MTOR	Mtor	LOC101391567	TBC1D7	Tbc1d7	LOC101405612
CSDC2	Csdc2	LOC101391518	MTPAP	Mtpap	LOC101394208	TBC1D8	Tbc1d8	LOC101408491
CSDE1	Csde1	LOC101402238	MTPN	Mtpn	LOC101393721	TBC1D8B	Tbc1d8b	LOC101387502
CSE1L	Cse1l	LOC101396457	MTR	Mtr	LOC101396115	TBC1D9	Tbc1d9	LOC101395664
CSF1	Csf1	LOC101398158	MTRF1	Mtrf1	LOC101402028	TBC1D9B	Tbc1d9b	LOC101409061
CSF1R	Csf1r	LOC101401479	MTRF1L	Mtrf1l	LOC101405815	TBCA	Tbca	LOC101387094
CSF2	Csf2	LOC101404146	MTRR	Mtrr	LOC101393754	TBCB	Tbcb	LOC101392395
CSF2RB	Csf2rb	LOC106800473	MTSS1	Mtss1	LOC101395156	TBCC	Tbcc	LOC101389693
CSF3	Csf3	LOC101399495	MTSS2	Mtss1l	LOC101390246	TBCCD1	Tbccd1	LOC101405225
CSF3R	Csf3r	LOC101394379	MTTP	Mttp	LOC101394829	TBCD	Tbcd	LOC101407264
CSGALNACT1	Csgalnact1	LOC101397122	MTURN	Mturn	LOC101387421	TBCE	Tbce	LOC101392802
CSGALNACT2	Csgalnact2	LOC101401597	MTUS1	Mtus1	LOC101393634	TBCEL	Tbcel	LOC101406123
CSK	Csk	LOC101394195	MTUS2	Mtus2	LOC101389996	TBCK	Tbck	LOC101405666
CSMD1	Csmd1	LOC101404139	MTX2	Mtx2	LOC101402268	TBK1	Tbk1	LOC101407511
CSMD2	Csmd2	LOC101407595	MTX3	Mtx3	LOC101405298	TBKBP1	Tbkbp1	LOC101390677
CSMD3	Csmd3	LOC101401147	MUC1	Muc1	LOC101391433	TBL1X	Tbl1x	LOC101399607
CSN3	Csn3	LOC101387253	MUC13	Muc13	LOC101403654	TBL1XR1	Tbl1xr1	LOC101408282
CSNK1A1	Csnk1a1	LOC101403575	MUC15	Muc15	LOC101397203	TBL2	Tbl2	LOC101402150
CSNK1D	Csnk1d	LOC101402976	MUC16	Muc16	LOC101388001	TBL3	Tbl3	LOC101390089
CSNK1E	Csnk1e	LOC101392119	MUC2	Muc2	LOC101407825	TBP	Tbp	LOC101404687
CSNK1G1	Csnk1g1	LOC101407577	MUC20	Muc20	LOC101404703	TBPL1	Tbpl1	LOC101405579
CSNK1G2	Csnk1g2	LOC101407917	MUC4	Muc4	LOC101406186	TBPL2	Tbpl2	LOC101405665
CSNK1G3	Csnk1g3	LOC101390369	MUC5AC	Muc5ac	LOC101407562	TBR1	Tbr1	LOC101393138
CSNK2A1	Csnk2a1	LOC101397596	MUC5B	Muc5b	LOC101407293	TBRG1	Tbrg1	LOC101392717
CSNK2A2	Csnk2a2	LOC101397509	MUC6	Muc6	LOC101408082	TBRG4	Tbrg4	LOC101393240
CSNK2B	Csnk2b	LOC101397652	MUL1	Mul1	LOC101391398	TBX1	Tbx1	LOC101406364
CSPG4	Cspg4	LOC101400318	MUS81	Mus81	LOC101387747	TBX10	Tbx10	LOC101408158
CSPG5	Cspg5	LOC101387588	MUSK	Musk	LOC101389443	TBX15	Tbx15	LOC101395118
CSPP1	Cspp1	LOC101393656	MUSTN1	Mustn1	LOC106803003	TBX18	Tbx18	LOC101388728
CSRNP1	Csrnp1	LOC101407655	MUTYH	Mutyh	LOC101394783	TBX19	Tbx19	LOC101387709
CSRNP2	Csrnp2	LOC101391918	MVB12A	Mvb12a	LOC101394970	TBX2	Tbx2	LOC101393737
CSRNP3	Csrnp3	LOC101409157	MVB12B	Mvb12b	LOC101405145	TBX20	Tbx20	LOC101400648
CSRP1	Csrp1	LOC101400471	MVD	Mvd	LOC101388957	TBX21	Tbx21	LOC101390587
CSRP2	Csrp2	LOC101393087	MVK	Mvk	LOC101396372	TBX22	Tbx22	LOC101390291
CSRP3	Csrp3	LOC101392723	MVP	Mvp	LOC101394760	TBX3	Tbx3	LOC101397064
CST11	Cst11	LOC101397552	MXD1	Mxd1	LOC101400808	TBX4	Tbx4	LOC101393069
CST3	Cst3	LOC101396253	MXD3	Mxd3	LOC101402698	TBX5	Tbx5	LOC101389885
CST6	Cst6	LOC101391516	MXD4	Mxd4	LOC101400361	TBX6	Tbx6	LOC101389057
CST7	Cst7	LOC101395731	MXI1	Mxi1	LOC101405237	TBXA2R	Tbxa2r	LOC101398827

CST8	Cst8	LOC101396775	MXRA8	Mxra8	LOC106801112	TBXAS1	Tbxas1	LOC101387898
CSTB	Cstb	LOC101402526	MYADM	Myadm	LOC101405011	TBXT	T	LOC101389659
CSTF1	Cstf1	LOC101407780	MYB	Myb	LOC101408100	TC2N	Tc2n	LOC101398537
CSTF2	Cstf2	LOC101402749	MYBBP1A	Mybbp1a	LOC101408827	TCAF1	Tcaf1	LOC101399215
CSTF2T	Cstf2t	LOC101401877	MYBL1	Mybl1	LOC101400628	TCAIM	Tcaim	LOC101397117
CSTF3	Cstf3	LOC101402464	MYBL2	Mybl2	LOC101395417	TCAP	Tcap	LOC101403598
CSTL1	Cstl1	LOC101397814	MYBPC1	Mybpc1	LOC101387679	TCEA1	Tcea1	LOC101389634
CTBP1	Ctbp1	LOC101392627	MYBPC2	Mybpc2	LOC101405454	TCEA2	Tcea2	LOC101401488
CTBP2	Ctbp2	LOC101400994	MYBPC3	Mybpc3	LOC101404743	TCEA3	Tcea3	LOC101402342
CTBS	Ctbs	LOC101408995	MYBPH	Mybph	LOC101394250	TCEAL1	Tceal1	LOC101407849
CTC1	Ctc1	LOC101387473	MYBPHL	Mybphl	LOC101407834	TCEAL3	Tceal3	LOC101407589
CTCF	Ctcf	LOC101392094	MYC	Myc	LOC101393217	TCEAL5	Tceal5	LOC101403817
CTDNEP1	Ctdnep1	LOC101387735	MYCBP	Mycbp	LOC101400776	TCEAL7	Tceal7	LOC101403291
CTDP1	Ctdp1	LOC101399541	MYCBP2	Mycbp2	LOC101389587	TCEAL8	Tceal8	LOC101404080
CTDSP1	Ctdsp1	LOC101408381	MYCBPAP	Mycbpap	LOC101391675	TCEAL9	Wbp5	LOC101403032
CTDSP2	Ctdsp2	LOC101403311	MYCL	Mycl	LOC101405596	TCEANC2	Tceanc2	LOC101393483
CTDSPL	Ctdspl	LOC101400484	MYCN	Mycn	LOC101395613	TCERG1	Tcerg1	LOC101398646
CTDSPL2	Ctdspl2	LOC101390800	MYCT1	Myct1	LOC101404603	TCERG1L	Tcerg1l	LOC101396751
CTF1	Ctf1	LOC101401528	MYD88	Myd88	LOC101402312	TCF12	Tcf12	LOC101393185
CTH	Cth	LOC101395159	MYDGF	Mydgf	LOC101398069	TCF15	Tcf15	LOC101408999
CTHRC1	Cthrc1	LOC101392179	MYEF2	Myef2	LOC101398735	TCF19	Tcf19	LOC101403129
CTIF	Ctif	LOC101390386	MYF5	Myf5	LOC101396136	TCF20	Tcf20	LOC101393744
CTLA4	Ctla4	LOC101396971	MYF6	Myf6	LOC101395878	TCF21	Tcf21	LOC101405312
CTNNA1	Ctnna1	LOC101402317	MYH1	Myh1	LOC101408049	TCF23	Tcf23	LOC101407007
CTNNA2	Ctnna2	LOC101406922	MYH10	Myh10	LOC101389807	TCF24	Tcf24	LOC101392022
CTNNA3	Ctnna3	LOC101393374	MYH11	Myh11	LOC101394782	TCF25	Tcf25	LOC101403342
CTNNAL1	Ctnnal1	LOC101408196	MYH13	Myh13	LOC101408566	TCF3	Tcf3	LOC101409115
CTNNB1	Ctnnb1	LOC101388978	MYH14	Myh14	LOC101389751	TCF4	Tcf4	LOC101399540
CTNNBIP1	Ctnnbip1	LOC101408629	MYH2	Myh2	LOC101407350	TCF7	Tcf7	LOC101396766
CTNNBL1	Ctnnbl1	LOC101403146	MYH3	Myh3	LOC101407078	TCF7L1	Tcf7l1	LOC101399508
CTNND1	Ctnnd1	LOC101392117	MYH4	Myh4	LOC101408312	TCF7L2	Tcf7l2	LOC101390141
CTNND2	Ctnnd2	LOC101403304	MYH6	Myh6	LOC101399876	TCFL5	Tcfl5	LOC101403839
CTNS	Ctns	LOC101390759	MYH7	Myh7	LOC101402408	TCHH	Tchh	LOC101405890
CTPS1	Ctps	LOC101389872	MYH7B	Myh7b	LOC101398779	TCHHL1	Tchhl1	LOC101387317
CTPS2	Ctps2	LOC101397519	MYH8	Myh8	LOC101407964	TCHP	Tchp	LOC101398017
CTR9	Ctr9	LOC101396317	MYH9	Myh9	LOC101395104	TCIM	1810011O10I	LOC101388713
CTRB2	Ctrb1	LOC101394581	MYL1	Myl1	LOC101398507	TCIRG1	Tcirg1	LOC101389548
CTRL	Ctrl	LOC101402448	MYL10	Myl10	LOC101387515	TCL1A	Tcl1	LOC101408921
CTSA	Ctsa	LOC101404098	MYL12A	Myl12a	LOC101403805	TCN2	Tcn2	LOC101395152
CTSB	Ctsb	LOC101392448	MYL12B	Myl12b	LOC101403368	TCP1	Tcp1	LOC101408340
CTSC	Ctsc	LOC101399831	MYL2	Myl2	LOC101397329	TCP11	Tcp11	LOC101389341
CTSD	Ctsd	LOC101400031	MYL3	Myl3	LOC101405814	TCP11L1	Tcp11l1	LOC101401771
CTSE	Ctse	LOC101396269	MYL4	Myl4	LOC101406556	TCP11L2	Tcp11l2	LOC101387324
CTSF	Ctsf	LOC101398889	MYL6	Myl6	LOC101399649	TCP11X2	Tcp11x2	LOC101408879
CTSG	Ctsg	LOC101389252	MYL6B	Myl6b	LOC101399387	TCTA	Tcta	LOC101402564
CTSH	Ctsh	LOC101387604	MYL7	Myl7	LOC101388609	TCTE1	Tcte1	LOC101394893
CTSK	Ctsk	LOC101395085	MYL9	Myl9	LOC101408818	TCTE3	Tcte3	LOC101403895
CTSO	Ctso	LOC101389759	MYLIP	Mylip	LOC101402365	TCTEX1D1	Tctex1d1	LOC106802000
CTSS	Ctss	LOC101394667	MYLK	Mylk	LOC101401210	TCTEX1D2	Tctex1d2	LOC101404953
CTSV	Ctsl	LOC101395805	MYLK2	Mylk2	LOC101391296	TCTN1	Tctn1	LOC101398109
CTSW	Ctsw	LOC101388960	MYLK3	Mylk3	LOC101403379	TCTN2	Tctn2	LOC101395677
CTSZ	Ctsz	LOC101403405	MYLK4	Mylk4	LOC101397174	TCTN3	Tctn3	LOC101407060
CTTN	Cttn	LOC101401102	MYLPF	Mylpf	LOC101396578	TDG	Tdg	LOC101405103
CTTNBP2	Cttnbp2	LOC101408770	MYMK	Tmem8c	LOC101398253	TDGF1	Tdgf1	LOC101404763

CTTNBP2NL	Cttnbp2nl	LOC101389424	MYNN	Mynn	LOC101396361	TDO2	Tdo2	LOC101389501
CTU2	Ctu2	LOC101408927	MYO10	Myo10	LOC101393358	TDP1	Tdp1	LOC101395694
CTXN1	Ctxn1	LOC101406517	MYO15A	Myo15	LOC101403850	TDP2	Tdp2	LOC101394737
CTXN2	Ctxn2	LOC101399785	MYO16	Myo16	LOC101409044	TDRD1	Tdrd1	LOC101390140
CTXN3	Ctxn3	LOC101408693	MYO18A	Myo18a	LOC101390835	TDRD12	Tdrd12	LOC101396140
CUBN	Cubn	LOC101399550	MYO18B	Myo18b	LOC101389706	TDRD3	Tdrd3	LOC101397514
CUEDC1	Cuedc1	LOC101393474	MYO19	Myo19	LOC101396219	TDRD5	Tdrd5	LOC101388151
CUEDC2	Cuedc2	LOC101391404	MYO1A	Myo1a	LOC101388670	TDRD6	Tdrd6	LOC101399020
CUL1	Cul1	LOC101394827	MYO1B	Myo1b	LOC101400433	TDRD7	Tdrd7	LOC101387621
CUL2	Cul2	LOC101389345	MYO1C	Myo1c	LOC101404469	TDRD9	Tdrd9	LOC101400720
CUL3	Cul3	LOC101405499	MYO1D	Myo1d	LOC101407026	TDRKH	Tdrkh	LOC101406825
CUL4A	Cul4a	LOC101389685	MYO1E	Myo1e	LOC101398077	TDRP	Tdrp	LOC101405455
CUL4B	Cul4b	LOC101398546	MYO1F	Myo1f	LOC101401648	TEAD1	Tead1	LOC101398814
CUL5	Cul5	LOC101402694	MYO1G	Myo1g	LOC101392567	TEAD2	Tead2	LOC101401706
CUL7	Cul7	LOC101407229	MYO1H	Myo1h	LOC101395850	TEAD3	Tead3	LOC101387121
CUL9	Cul9	LOC101404349	MYO3A	Myo3a	LOC101395663	TEAD4	Tead4	LOC101391609
CUTA	Cuta	LOC101395746	MYO3B	Myo3b	LOC101402872	TEC	Tec	LOC101407202
CUTC	Cutc	LOC101397758	MYO5A	Myo5a	LOC101407310	TECPR1	Tecpr1	LOC101408436
CUX1	Cux1	LOC101408870	MYO5B	Myo5b	LOC101406003	TECPR2	Tecpr2	LOC101393820
CUX2	Cux2	LOC101400442	MYO5C	Myo5c	LOC101407036	TECR	Tecr	LOC101391385
CUZD1	Cuzd1	LOC101406483	MYO6	Myo6	LOC101402499	TECRL	Tecrl	LOC101398391
CWC15	Cwc15	LOC101394635	MYO7A	Myo7a	LOC101407352	TECTA	Tecta	LOC101405862
CWC22	Cwc22	LOC101409146	MYO7B	Myo7b	LOC101391230	TECTB	Tectb	LOC101408898
CWC25	Cwc25	LOC101408835	MYO9A	Myo9a	LOC101405385	TEDC1	4930427A07R	LOC101407084
CWC27	Cwc27	LOC101392601	MYO9B	Myo9b	LOC101397822	TEDDM1	Teddm1b	LOC101405227
CWF19L1	Cwf19l1	LOC101399905	MYOC	Myoc	LOC101403392	TEF	Tef	LOC101404489
CWF19L2	Cwf19l2	LOC101404187	MYOCD	Myocd	LOC101405086	TEFM	Tefm	LOC101388544
CWH43	Cwh43	LOC101387756	MYOD1	Myod1	LOC101407374	TEK	Tek	LOC101397666
CX3CR1	Cx3cr1	LOC101408171	MYOF	Myof	LOC101397663	TEKT1	Tekt1	LOC101407702
CXADR	Cxadr	LOC101396061	MYOG	Myog	LOC101394758	TEKT2	Tekt2	LOC101390903
CXCL10	Cxcl10	LOC101405872	MYOM1	Myom1	LOC101404413	TEKT3	Tekt3	LOC101403328
CXCL12	Cxcl12	LOC101399046	MYOM2	Myom2	LOC101399433	TEKT5	Tekt5	LOC101408675
CXCL13	Cxcl13	LOC101400524	MYOM3	Myom3	LOC101392677	TELO2	Telo2	LOC101395012
CXCL14	Cxcl14	LOC101391374	MYORG	AI464131	LOC101388835	TEN1	Ten1	LOC101396294
CXCL16	Cxcl16	LOC101389535	MYOT	Myot	LOC101387258	TENM1	Tenm1	LOC101394670
CXCL17	Cxcl17	LOC101402135	MYOZ1	Myoz1	LOC101391235	TENM2	Tenm2	LOC101394907
CXCL6	Cxcl5	LOC101393674	MYOZ2	Myoz2	LOC101390736	TENM3	Tenm3	LOC101401928
CXCL9	Cxcl9	LOC101405608	MYOZ3	Myoz3	LOC101399208	TENM4	Tenm4	LOC101388765
CXCR1	Cxcr1	LOC101406024	MYPN	Mypn	LOC101391161	TENT2	Papd4	LOC101405824
CXCR2	Cxcr2	LOC101405754	MYPOP	Mypop	LOC101387332	TENT4A	Papd7	LOC101393338
CXCR3	Cxcr3	LOC101401442	MYRF	Myrf	LOC101393916	TENT4B	Papd5	LOC101394917
CXCR4	Cxcr4	LOC101406876	MYRFL	Myrfl	LOC101387556	TENT5A	Fam46a	LOC101405650
CXCR5	Cxcr5	LOC101394720	MYRIP	Myrip	LOC101387761	TENT5B	Fam46b	LOC101399552
CXCR6	Cxcr6	LOC101402228	MYSM1	Mysm1	LOC101399847	TEP1	Tep1	LOC101407574
CXXC1	Cxxc1	LOC101394708	MYT1	Myt1	LOC101388247	TEPP	Tepp	LOC101396023
CXXC5	Cxxc5	LOC101398154	MYT1L	Myt1l	LOC101400373	TEPSIN	Enthd2	LOC101394403
CXorf38	1810030O07R	LOC101406821	MYZAP	Myzap	LOC101394029	TERB1	Terb1	LOC101403844
CXorf58	Fam90a1b	LOC101408747	MZB1	Mzb1	LOC101399774	TERB2	Terb2	LOC101393011
CXorf65	Gm614	LOC101406846	MZF1	Mzf1	LOC101405801	TERF1	Terf1	LOC101398622
CXorf66	Gm7073	LOC101387263	MZT1	Mzt1	LOC101407047	TERF2	Terf2	LOC101403768
CYB561	Cyb561	LOC101387467	N4BP1	N4bp1	LOC101401411	TERF2IP	Terf2ip	LOC101403604
CYB561A3	Cyb561a3	LOC101394328	N4BP2	N4bp2	LOC101396323	TERT	Tert	LOC101389836
CYB561D1	Cyb561d1	LOC101403191	N4BP2L1	N4bp2l1	LOC101394750	TES	Tes	LOC101390016
CYB561D2	Cyb561d2	LOC101392218	N4BP2L2	N4bp2l2	LOC101395175	TESC	Tesc	LOC101388412

CYB5A	Cyb5a	LOC101395739	N4BP3	N4bp3	LOC101397840	TESK1	Tesk1	LOC101387110
CYB5B	Cyb5b	LOC101404106	N6AMT1	N6amt1	LOC101395503	TESK2	Tesk2	LOC101395208
CYB5D1	Cyb5d1	LOC101398030	NAA10	Naa10	LOC101389770	TESPA1	Tespa1	LOC101400169
CYB5D2	Cyb5d2	LOC101387736	NAA15	Naa15	LOC101398505	TET1	Tet1	LOC101388566
CYB5R1	Cyb5r1	LOC101395532	NAA16	Naa16	LOC101402290	TET2	Tet2	LOC101407239
CYB5R2	Cyb5r2	LOC101405017	NAA20	Naa20	LOC101400095	TET3	Tet3	LOC101408150
CYB5R3	Cyb5r3	LOC101394852	NAA25	Naa25	LOC101394990	TEX10	Tex10	LOC101393872
CYB5R4	Cyb5r4	LOC101387957	NAA30	Naa30	LOC101407238	TEX101	Tex101	LOC101400560
CYB5RL	Cyb5rl	LOC101393747	NAA35	Naa35	LOC101391280	TEX11	Tex11	LOC101397711
CYBA	Cyba	LOC101387746	NAA38	Naa38	LOC101398286	TEX12	Tex12	LOC101391998
CYBB	Cybb	LOC101389295	NAA40	Naa40	LOC101387241	TEX13A	Tex13a	LOC106804013
CYBC1	BC017643	LOC101404110	NAA50	Naa50	LOC101389819	TEX14	Tex14	LOC101408924
CYBRD1	Cybrd1	LOC101398010	NAA60	Naa60	LOC101400109	TEX15	Tex15	LOC101401670
CYC1	Cyc1	LOC101391632	NAAA	Naaa	LOC101404898	TEX2	Tex2	LOC101394310
CYCS	Cycs	LOC101396758	NAALAD2	Naalad2	LOC101400781	TEX22	Tex22	LOC101405784
CYFIP1	Cyfip1	LOC101391136	NAALADL1	Naaladl1	LOC101400192	TEX26	Tex26	LOC101407971
CYFIP2	Cyfip2	LOC101387129	NAALADL2	Naaladl2	LOC101408539	TEX261	Tex261	LOC101396740
CYGB	Cygb	LOC101405686	NAB1	Nab1	LOC101399643	TEX264	Tex264	LOC101395287
CYHR1	Cyhr1	LOC101388305	NAB2	Nab2	LOC101389365	TEX30	Tex30	LOC101405741
CYLC1	Cylc1	LOC101401078	NABP1	Nabp1	LOC101400692	TEX33	Tex33	LOC101397535
CYLC2	Cylc2	LOC101397993	NABP2	Nabp2	LOC101400961	TEX35	Tex35	LOC101392259
CYLD	Cyld	LOC101403503	NACA	Naca	LOC101407950	TEX36	Tex36	LOC101391354
CYP11A1	Cyp11a1	LOC101391211	NACAD	Nacad	LOC101401617	TEX37	Tex37	LOC101395872
CYP17A1	Cyp17a1	LOC101394138	NACC1	Nacc1	LOC101408874	TEX38	Tex38	LOC101403194
CYP19A1	Cyp19a1	LOC101406950	NACC2	Nacc2	LOC101405320	TEX43	Tex43	LOC101388539
CYP1A1	Cyp1a1	LOC101393693	NADK	Nadk	LOC101396629	TEX45	1700019B03F	LOC101392509
CYP1A2	Cyp1a2	LOC101393950	NADK2	Nadk2	LOC101404691	TEX46	4930549C01F	LOC106801174
CYP20A1	Cyp20a1	LOC101394382	NADSYN1	Nadsyn1	LOC101400300	TEX49	4930415O20F	LOC101396452
CYP24A1	Cyp24a1	LOC101387640	NAE1	Nae1	LOC101404105	TEX51	Gm35060	LOC106800815
CYP26A1	Cyp26a1	LOC101397248	NAF1	Naf1	LOC101391543	TEX52	4933413G19F	LOC101389050
CYP26B1	Cyp26b1	LOC101392456	NAGA	Naga	LOC101387164	TEX55	4930435E12F	LOC101401425
CYP26C1	Cyp26c1	LOC101396977	NAGK	Nagk	LOC101396476	TEX9	Tex9	LOC101408876
CYP27A1	Cyp27a1	LOC101389105	NAGLU	Naglu	LOC101396823	TF	Trf	LOC101390280
CYP27B1	Cyp27b1	LOC101401836	NAGPA	Nagpa	LOC101390349	TFAM	Tfam	LOC101401319
CYP2A6	Cyp2a12	LOC101399438	NAGS	Nags	LOC101389992	TFAP2A	Tfap2a	LOC101387309
CYP2C19	Cyp2c55	LOC101403493	NAIF1	Naif1	LOC101391989	TFAP2B	Tfap2b	LOC101407938
CYP2C8	Cyp2c38	LOC101404357	NALCN	Nalcn	LOC101403646	TFAP2C	Tfap2c	LOC101407254
CYP2D6	Cyp2d22	LOC101388189	NAMPT	Nampt	LOC101406745	TFAP2D	Tfap2d	LOC101408725
CYP2D7	Cyp2d11	LOC101388606	NANOG	Nanog	LOC101390436	TFAP2E	Tfap2e	LOC101399899
CYP2E1	Cyp2e1	LOC101392904	NANOS1	Nanos1	LOC101392686	TFAP4	Tfap4	LOC101397198
CYP2R1	Cyp2r1	LOC101403002	NANOS2	Nanos2	LOC101401880	TFB1M	Tfb1m	LOC101408945
CYP2S1	Cyp2s1	LOC101393687	NANOS3	Nanos3	LOC101388379	TFB2M	Tfb2m	LOC101390360
CYP2U1	Cyp2u1	LOC101393129	NANP	Nanp	LOC101394190	TFCP2	Tfcp2	LOC101392355
CYP2W1	Cyp2w1	LOC101407561	NANS	Nans	LOC101389191	TFCP2L1	Tfcp2l1	LOC101396001
CYP39A1	Cyp39a1	LOC101391480	NAP1L1	Nap1l1	LOC101390939	TFDP1	Tfdp1	LOC101391226
CYP3A5	Cyp3a13	LOC101408097	NAP1L2	Nap1l2	LOC101395119	TFDP2	Tfdp2	LOC101396936
CYP46A1	Cyp46a1	LOC101388766	NAP1L4	Nap1l4	LOC101396148	TFE3	Tfe3	LOC101407175
CYP4A11	Cyp4a31	LOC101404254	NAPA	Napa	LOC101407991	TFEB	Tfeb	LOC101393876
CYP4A22	Cyp4a10	LOC101398050	NAPB	Napb	LOC101398073	TFEC	Tfec	LOC101390610
CYP4B1	Cyp4b1	LOC101403985	NAPEPLD	Napepld	LOC101390096	TFF2	Tff2	LOC101402441
CYP4V2	Cyp4v3	LOC101390324	NAPG	Napg	LOC101396507	TFF3	Tff3	LOC101402185
CYP4X1	Cyp4x1	LOC101398306	NAPRT	Naprt	LOC101397387	TFG	Tfg	LOC101387082
CYP51A1	Cyp51	LOC101397171	NARF	Narf	LOC101404567	TFIP11	Tfip11	LOC101388936
CYP7A1	Cyp7a1	LOC101394575	NARS2	Nars2	LOC101388504	TFPI	Tfpi	LOC101393798

CYP7B1	Cyp7b1	LOC101407635	NASP	Nasp	LOC101396861	TFPI2	Tfpi2	LOC101401592
CYP8B1	Cyp8b1	LOC101395029	NAT10	Nat10	LOC101404222	TFPT	Tfpt	LOC101407279
CYREN	3110062M04	LOC101398192	NAT14	Nat14	LOC101393408	TFR2	Tfr2	LOC101396260
CYSLTR1	Cysltr1	LOC101388374	NAT9	Nat9	LOC101387223	TFRC	Tfrc	LOC101406704
CYTH1	Cyth1	LOC101399137	NAV1	Nav1	LOC101400196	TG	Tg	LOC101389712
CYTH2	Cyth2	LOC101392042	NAV2	Nav2	LOC101393412	TGDS	Tgds	LOC101394710
CYTH3	Cyth3	LOC101388370	NAV3	Nav3	LOC101393923	TGFA	Tgfa	LOC101398297
CYTH4	Cyth4	LOC101399939	NAXD	Carkd	LOC101387702	TGFB1	Tgfb1	LOC101401889
CYTIP	Cytip	LOC101399813	NAXE	Apoa1bp	LOC101407714	TGFB1I1	Tgfb1i1	LOC101407985
CYTL1	Cytl1	LOC101406310	NBAS	Nbas	LOC101395352	TGFB2	Tgfb2	LOC101395371
CYYR1	Cyyr1	LOC101394470	NBEA	Nbea	LOC101396221	TGFB3	Tgfb3	LOC101390228
CZIB	0610037L13R	LOC101389740	NBEAL1	Nbeal1	LOC101394137	TGFBI	Tgfbi	LOC101390106
D2HGDH	D2hgdh	LOC101392533	NBEAL2	Nbeal2	LOC101405293	TGFBR1	Tgfbr1	LOC101391557
DAAM1	Daam1	LOC101392678	NBL1	Nbl1	LOC101388661	TGFBR2	Tgfbr2	LOC101389658
DAAM2	Daam2	LOC101397826	NBN	Nbn	LOC101394147	TGFBR3	Tgfbr3	LOC101390114
DAB1	Dab1	LOC101398894	NBR1	Nbr1	LOC101409001	TGFBR3L	Tgfbr3l	LOC101390717
DAB2	Dab2	LOC101388135	NCALD	Ncald	LOC101388943	TGFBRAP1	Tgfbrap1	LOC101390428
DAB2IP	Dab2ip	LOC101406375	NCAM1	Ncam1	LOC101390739	TGIF1	Tgif1	LOC101403106
DACH1	Dach1	LOC101406775	NCAM2	Ncam2	LOC101390235	TGIF2	Tgif2	LOC106799916
DACH2	Dach2	LOC101393399	NCAN	Ncan	LOC101403290	TGM1	Tgm1	LOC101393269
DACT1	Dact1	LOC101407497	NCAPD2	Ncapd2	LOC101405969	TGM2	Tgm2	LOC101401934
DACT3	Dact3	LOC101394257	NCAPD3	Ncapd3	LOC101388877	TGM3	Tgm3	LOC101405087
DAD1	Dad1	LOC101391133	NCAPG	Ncapg	LOC101392281	TGM4	Tgm4	LOC101403185
DAG1	Dag1	LOC101403714	NCAPG2	Ncapg2	LOC101400522	TGM5	Tgm5	LOC101402846
DAGLA	Dagla	LOC101393661	NCAPH	Ncaph	LOC101401769	TGM6	Tgm6	LOC101404830
DAGLB	Daglb	LOC101402566	NCAPH2	Ncaph2	LOC101408159	TGM7	Tgm7	LOC101403111
DALRD3	Dalrd3	LOC101396327	NCBP1	Ncbp1	LOC101388397	TGS1	Tgs1	LOC101404480
DAND5	Dand5	LOC101407740	NCBP2	Ncbp2	LOC101389268	TH	Th	LOC101397375
DAO	Dao	LOC101394143	NCBP3	Ncbp3	LOC101389128	THADA	Thada	LOC101403341
DAP	Dap	LOC101403757	NCCRP1	Nccrp1	LOC101408696	THAP1	Thap1	LOC101395377
DAP3	Dap3	LOC101396742	NCDN	Ncdn	LOC101388925	THAP11	Thap11	LOC101395075
DAPK1	Dapk1	LOC101392482	NCEH1	Nceh1	LOC101388738	THAP2	Thap2	LOC101397763
DAPK2	Dapk2	LOC101405838	NCF1	Ncf1	LOC101397725	THAP3	Thap3	LOC101401829
DAPK3	Dapk3	LOC101396767	NCF2	Ncf2	LOC101400508	THAP4	Thap4	LOC101391403
DAPL1	Dapl1	LOC101397665	NCF4	Ncf4	LOC101397282	THAP7	Thap7	LOC101400138
DAPP1	Dapp1	LOC101395073	NCK1	Nck1	LOC101407205	THBD	Thbd	LOC101399439
DARS2	Dars2	LOC101399022	NCK2	Nck2	LOC101391340	THBS1	Thbs1	LOC101387432
DAW1	Daw1	LOC101387205	NCKAP1	Nckap1	LOC101391160	THBS2	Thbs2	LOC101403114
DAXX	Daxx	LOC101397312	NCKAP1L	Nckap1l	LOC101399034	THBS3	Thbs3	LOC101391678
DAZAP1	Dazap1	LOC101387182	NCKAP5	Nckap5	LOC101389690	THBS4	Thbs4	LOC101405032
DAZAP2	Dazap2	LOC101394218	NCKAP5L	Nckap5l	LOC101406126	THEG	Theg	LOC101393853
DAZL	Dazl	LOC101400653	NCKIPSD	Nckipsd	LOC101393678	THEGL	Thegl	LOC101392911
DBF4	Dbf4	LOC101392092	NCL	Ncl	LOC101391915	THEM4	Them4	LOC101408667
DBH	Dbh	LOC101399018	NCLN	Ncln	LOC101400837	THEM5	Them5	LOC101408413
DBI	Dbi	LOC101393614	NCMAP	Ncmap	LOC101387283	THEM6	Them6	LOC101406302
DBN1	Dbn1	LOC101399730	NCOA1	Ncoa1	LOC101395627	THEMIS	Themis	LOC101397477
DBNDD1	Dbndd1	LOC101406408	NCOA2	Ncoa2	LOC101396126	THEMIS2	Themis2	LOC101405663
DBNDD2	Dbndd2	LOC101408390	NCOA3	Ncoa3	LOC101397416	THG1L	Thg1l	LOC101408298
DBNL	Dbnl	LOC101387165	NCOA4	Ncoa4	LOC101405170	THNSL2	Thnsl2	LOC101395616
DBP	Dbp	LOC101399432	NCOA5	Ncoa5	LOC101401487	THOC1	Thoc1	LOC101408263
DBR1	Dbr1	LOC101405812	NCOA6	Ncoa6	LOC101398778	THOC2	Thoc2	LOC101396569
DBT	Dbt	LOC101393766	NCOA7	Ncoa7	LOC101393867	THOC3	Thoc3	LOC101387632
DBX1	Dbx1	LOC101394177	NCOR1	Ncor1	LOC101398438	THOC5	Thoc5	LOC101404028
DBX2	Dbx2	LOC101387303	NCOR2	Ncor2	LOC101403314	THOC6	Thoc6	LOC101402994

DCAF1	Vprbp	LOC101394602	NCR1	Ncr1	LOC101389154	THOC7	Thoc7	LOC101401792
DCAF10	Dcaf10	LOC101406374	NCS1	Ncs1	LOC101408974	THOP1	Thop1	LOC101402924
DCAF11	Dcaf11	LOC101397891	NCSTN	Ncstn	LOC101393356	THPO	Thpo	LOC101394628
DCAF12	Dcaf12	LOC101390406	NDC1	Ndc1	LOC101390775	THRA	Thra	LOC101397778
DCAF12L1	Dcaf12l1	LOC101394415	NDC80	Ndc80	LOC101405914	THRAP3	Thrap3	LOC101392676
DCAF12L2	Dcaf12l2	LOC101404916	NDE1	Nde1	LOC101395380	THRB	Thrb	LOC101406080
DCAF13	Dcaf13	LOC101392693	NDEL1	Ndel1	LOC101390579	THRSP	Thrsp	LOC101387229
DCAF15	Dcaf15	LOC101388902	NDFIP1	Ndfip1	LOC101404605	THSD1	Thsd1	LOC101399054
DCAF17	Dcaf17	LOC101402092	NDFIP2	Ndfip2	LOC101392067	THSD4	Thsd4	LOC101404868
DCAF4	Dcaf4	LOC101396709	NDN	Ndn	LOC101407618	THSD7A	Thsd7a	LOC101389532
DCAF5	Dcaf5	LOC101389099	NDNF	Ndnf	LOC101389009	THSD7B	Thsd7b	LOC101387366
DCAF6	Dcaf6	LOC101398501	NDOR1	Ndor1	LOC101390972	THTPA	Thtpa	LOC101401893
DCAF7	Dcaf7	LOC101388851	NDP	Ndp	LOC101402892	THUMPD1	Thumpd1	LOC101399448
DCAF8	Dcaf8	LOC101390384	NDRG1	Ndrg1	LOC101389283	THUMPD2	Thumpd2	LOC101389300
DCAKD	Dcakd	LOC101399047	NDRG2	Ndrg2	LOC101401374	THUMPD3	Thumpd3	LOC101394619
DCBLD1	Dcbld1	LOC101402325	NDRG3	Ndrg3	LOC101407338	THY1	Thy1	LOC101408806
DCBLD2	Dcbld2	LOC101405979	NDRG4	Ndrg4	LOC101398692	THYN1	Thyn1	LOC101389400
DCC	Dcc	LOC101397138	NDST1	Ndst1	LOC101399473	TIA1	Tia1	LOC101399502
DCDC5	Dcdc5	LOC101406487	NDST2	Ndst2	LOC101388828	TIAL1	Tial1	LOC101398105
DCDC1	Dcdc1	LOC101406747	NDST3	Ndst3	LOC101392162	TIAM1	Tiam1	LOC101402605
DCDC2B	Dcdc2b	LOC101398761	NDST4	Ndst4	LOC101392944	TIAM2	Tiam2	LOC101408078
DCDC2C	Dcdc2c	LOC101393075	NDUFA1	Ndufa1	LOC101400411	TICAM1	Ticam1	LOC101397811
DCHS1	Dchs1	LOC101401968	NDUFA10	Ndufa10	LOC101407243	TICRR	Ticrr	LOC101402449
DCK	Dck	LOC101390444	NDUFA11	Ndufa11	LOC101408439	TIE1	Tie1	LOC101404778
DCLK1	Dclk1	LOC101397091	NDUFA12	Ndufa12	LOC101387411	TIFAB	Tifab	LOC101391882
DCLK2	Dclk2	LOC101400843	NDUFA13	Ndufa13	LOC101401729	TIGAR	Tigar	LOC101389644
DCLK3	Dclk3	LOC101397289	NDUFA2	Ndufa2	LOC101391535	TIGD2	Tigd2	LOC101408393
DCLRE1A	Dclre1a	LOC101389359	NDUFA3	Ndufa3	LOC101406586	TIGD3	Tigd3	LOC101403956
DCLRE1B	Dclre1b	LOC101404679	NDUFA4	Ndufa4	LOC101389023	TIGIT	Tigit	LOC101401952
DCLRE1C	Dclre1c	LOC101391154	NDUFA4L2	Ndufa4l2	LOC101391493	TIMD4	Timd4	LOC101396719
DCN	Dcn	LOC101405189	NDUFA5	Ndufa5	LOC101402558	TIMELESS	Timeless	LOC101405162
DCP1A	Dcp1a	LOC101408690	NDUFA6	Ndufa6	LOC101387929	TIMM10	Timm10	LOC101388959
DCP1B	Dcp1b	LOC101398052	NDUFA7	Ndufa7	LOC101404000	TIMM10B	Timm10b	LOC101400201
DCP2	Dcp2	LOC101401368	NDUFA8	Ndufa8	LOC101406961	TIMM13	Timm13	LOC101404770
DCPS	Dcps	LOC101401776	NDUFA9	Ndufa9	LOC101387933	TIMM17A	Timm17a	LOC101399420
DCST1	Dcst1	LOC101387235	NDUFAB1	Ndufab1	LOC101387271	TIMM17B	Timm17b	LOC101388091
DCST2	Dcst2	LOC101409086	NDUFAF1	Ndufaf1	LOC101397470	TIMM21	Timm21	LOC101395480
DCSTAMP	Dcstamp	LOC101393724	NDUFAF2	Ndufaf2	LOC101388645	TIMM22	Timm22	LOC101407966
DCT	Dct	LOC101394454	NDUFAF3	Ndufaf3	LOC101396938	TIMM23B	Timm23	LOC101405614
DCTD	Dctd	LOC101402350	NDUFAF4	Ndufaf4	LOC101401553	TIMM44	Timm44	LOC101406267
DCTN1	Dctn1	LOC101396042	NDUFAF5	Ndufaf5	LOC101394073	TIMM50	Timm50	LOC101390196
DCTN2	Dctn2	LOC101395591	NDUFAF6	Ndufaf6	LOC101404464	TIMM8A	Timm8a1	LOC101400399
DCTN3	Dctn3	LOC101407605	NDUFAF7	Ndufaf7	LOC101406414	TIMM8B	Timm8b	LOC101393134
DCTN4	Dctn4	LOC101398107	NDUFB10	Ndufb10	LOC101390600	TIMM9	Timm9	LOC101391996
DCTN5	Dctn5	LOC101408705	NDUFB11	Ndufb11	LOC101396650	TIMMDC1	Timmdc1	LOC101405699
DCTN6	Dctn6	LOC101402601	NDUFB2	Ndufb2	LOC101408480	TIMP1	Timp1	LOC101399754
DCTPP1	Dctpp1	LOC101397799	NDUFB3	Ndufb3	LOC101389202	TIMP2	Timp2	LOC101389722
DCUN1D1	Dcun1d1	LOC101400156	NDUFB4	Ndufb4	LOC101401862	TIMP3	Timp3	LOC101391523
DCUN1D2	Dcun1d2	LOC101390722	NDUFB5	Ndufb5	LOC101405150	TIMP4	Timp4	LOC101389860
DCUN1D3	Dcun1d3	LOC101395127	NDUFB6	Ndufb6	LOC101394725	TINAG	Tinag	LOC101402258
DCUN1D4	Dcun1d4	LOC101388202	NDUFB7	Ndufb7	LOC101391801	TINAGL1	Tinagl1	LOC101394291
DCUN1D5	Dcun1d5	LOC101407861	NDUFB8	Ndufb8	LOC101401582	TINF2	Tinf2	LOC101393769
DCX	Dcx	LOC101397964	NDUFB9	Ndufb9	LOC101395600	TIPARP	Tiparp	LOC101390111
DCXR	Dcxr	LOC101401235	NDUFC1	Ndufc1	LOC101398939	TIPIN	Tipin	LOC101395040

DDA1	Dda1	LOC101395654	NDUFC2	Ndufc2	LOC101387477	TIPRL	Tiprl	LOC101388229
DDAH1	Ddah1	LOC101389891	NDUFS1	Ndufs1	LOC101399383	TJAP1	Tjap1	LOC101400947
DDAH2	Ddah2	LOC101395054	NDUFS2	Ndufs2	LOC101397308	TJP1	Tjp1	LOC101388419
DDB1	Ddb1	LOC101390935	NDUFS3	Ndufs3	LOC101407535	TJP2	Tjp2	LOC101390617
DDB2	Ddb2	LOC101401953	NDUFS4	Ndufs4	LOC101397479	TJP3	Tjp3	LOC101398567
DDC	Ddc	LOC101397357	NDUFS5	Ndufs5	LOC101402343	TK1	Tk1	LOC101398119
DDHD1	Ddhd1	LOC101404805	NDUFS6	Ndufs6	LOC101391276	TK2	Tk2	LOC101402363
DDHD2	Ddhd2	LOC101391579	NDUFS7	Ndufs7	LOC106799946	TKFC	Tkfc	LOC101391191
DDI1	Ddi1	LOC101407153	NDUFS8	Ndufs8	LOC101389145	TKT	Tkt	LOC101408079
DDI2	Ddi2	LOC101398855	NDUFV1	Ndufv1	LOC101408754	TKTL1	Tktl1	LOC101392063
DDIAS	Ddias	LOC101389472	NDUFV2	Ndufv2	LOC101398474	TKTL2	Tktl2	LOC101390627
DDIT3	Ddit3	LOC101394909	NDUFV3	Ndufv3	LOC101399650	TLCD3A	Fam57a	LOC101406908
DDIT4	Ddit4	LOC101396890	NEB	Neb	LOC101399890	TLCD3B	Fam57b	LOC101390358
DDIT4L	Ddit4l	LOC101396287	NEBL	Nebl	LOC101405490	TLDC2	Tldc2	LOC101406390
DDN	Ddn	LOC101399733	NECAB1	Necab1	LOC101395157	TLE1	Tle1	LOC101404059
DDO	Ddo	LOC101397225	NECAB2	Necab2	LOC101392818	TLE2	Tle2	LOC101402233
DDOST	Ddost	LOC101392158	NECAB3	Necab3	LOC101395297	TLE3	Tle3	LOC101402760
DDR1	Ddr1	LOC101404701	NECAP1	Necap1	LOC101391520	TLE4	Tle4	LOC101404850
DDR2	Ddr2	LOC101397565	NECAP2	Necap2	LOC101403750	TLE5	Aes	LOC101402489
DDRGK1	Ddrgk1	LOC101387388	NECTIN1	Nectin1	LOC101408548	TLE6	Tle6	LOC101402751
DDX1	Ddx1	LOC101390430	NECTIN2	Nectin2	LOC101392731	TLK1	Tlk1	LOC101399030
DDX10	Ddx10	LOC101400606	NECTIN3	Nectin3	LOC101404836	TLK2	Tlk2	LOC101408402
DDX17	Ddx17	LOC101393238	NECTIN4	Nectin4	LOC101392512	TLL1	Tll1	LOC101405778
DDX18	Ddx18	LOC101391306	NEDD1	Nedd1	LOC101391947	TLL2	Tll2	LOC101387298
DDX19A	Ddx19a	LOC101392365	NEDD4	Nedd4	LOC101391799	TLN1	Tln1	LOC101388643
DDX19B	Ddx19b	LOC101392800	NEDD4L	Nedd4l	LOC101403640	TLN2	Tln2	LOC101401377
DDX20	Ddx20	LOC101390110	NEDD8	Nedd8	LOC101394521	TLNRD1	Mesdc1	LOC101395161
DDX21	Ddx21	LOC101408983	NEDD9	Nedd9	LOC101407260	TLR3	Tlr3	LOC101389604
DDX23	Ddx23	LOC101397921	NEFH	Nefh	LOC101404819	TLR4	Tlr4	LOC101401387
DDX24	Ddx24	LOC101405260	NEFL	Nefl	LOC101408313	TLR7	Tlr7	LOC101388262
DDX25	Ddx25	LOC101399327	NEFM	Nefm	LOC101408567	TLR9	Tlr9	LOC106799927
DDX27	Ddx27	LOC101395935	NEGR1	Negr1	LOC101397678	TLX1	Tlx1	LOC101388572
DDX28	Ddx28	LOC101402973	NEIL1	Neil1	LOC101397636	TLX2	Tlx2	LOC101400813
DDX31	Ddx31	LOC101406111	NEIL2	Neil2	LOC101393650	TLX3	Tlx3	LOC101394553
DDX39A	Ddx39	LOC101390630	NEIL3	Neil3	LOC101401221	TM2D1	Tm2d1	LOC101403585
DDX39B	Ddx39b	LOC101401572	NEK1	Nek1	LOC101408998	TM2D2	Tm2d2	LOC101394908
DDX3X	Ddx3x	LOC101405263	NEK10	Nek10	LOC101408255	TM2D3	Tm2d3	LOC101389121
DDX4	Ddx4	LOC101402679	NEK11	Nek11	LOC101394103	TM4SF1	Tm4sf1	LOC101389656
DDX41	Ddx41	LOC101398601	NEK2	Nek2	LOC101387628	TM4SF19	Tm4sf19	LOC101407684
DDX42	Ddx42	LOC101391931	NEK3	Nek3	LOC101406316	TM4SF20	Tm4sf20	LOC101407946
DDX43	Ddx43	LOC101399179	NEK4	Nek4	LOC101389170	TM4SF4	Tm4sf4	LOC101389414
DDX46	Ddx46	LOC101394183	NEK5	Nek5	LOC101406049	TM4SF5	Tm4sf5	LOC101389808
DDX47	Ddx47	LOC101394751	NEK6	Nek6	LOC101398847	TM6SF1	Tm6sf1	LOC101392280
DDX49	Ddx49	LOC101406179	NEK7	Nek7	LOC101405361	TM6SF2	Tm6sf2	LOC101403031
DDX5	Ddx5	LOC101395077	NEK8	Nek8	LOC101393903	TM7SF2	Tm7sf2	LOC101401520
DDX50	Ddx50	LOC101387127	NEK9	Nek9	LOC101387374	TM7SF3	Tm7sf3	LOC101392974
DDX51	Ddx51	LOC101406458	NELFA	Nelfa	LOC101395858	TM9SF1	Tm9sf1	LOC101395893
DDX52	Ddx52	LOC101400462	NELFB	Nelfb	LOC101394047	TM9SF2	Tm9sf2	LOC101401298
DDX54	Ddx54	LOC101392437	NELFCD	Nelfcd	LOC101403669	TM9SF3	Tm9sf3	LOC101387551
DDX55	Ddx55	LOC101394557	NELFE	Nelfe	LOC101390225	TM9SF4	Tm9sf4	LOC101389429
DDX56	Ddx56	LOC101401350	NELL1	Nell1	LOC101395189	TMA16	Tma16	LOC101391042
DDX58	Ddx58	LOC101395587	NELL2	Nell2	LOC101408642	TMBIM1	Tmbim1	LOC101407863
DDX59	Ddx59	LOC101403703	NEMF	Nemf	LOC101399929	TMBIM4	Tmbim4	LOC101387379
DDX6	Ddx6	LOC101395144	NENF	Nenf	LOC101390309	TMBIM6	Tmbim6	LOC101405867

DDX60	Ddx60	LOC101390837	NEO1	Neo1	LOC101391386	TMC1	Tmc1	LOC101392725
DEAF1	Deaf1	LOC101394261	NEPRO	Nepro	LOC101391011	TMC2	Tmc2	LOC101393318
DECR1	Decr1	LOC101403149	NES	Nes	LOC101409087	TMC3	Tmc3	LOC101394654
DECR2	Decr2	LOC101404586	NET1	Net1	LOC101404180	TMC4	Tmc4	LOC101407809
DEDD	Dedd	LOC101393610	NETO1	Neto1	LOC101394793	TMC5	Tmc5	LOC101391380
DEDD2	Dedd2	LOC101405113	NETO2	Neto2	LOC101402580	TMC6	Tmc6	LOC101387908
DEF6	Def6	LOC101388225	NEU1	Neu1	LOC101392420	TMC7	Tmc7	LOC101391970
DEF8	Def8	LOC101403603	NEU2	Neu2	LOC101396976	TMC8	Tmc8	LOC101388170
DEFB1	Defb9	LOC106803559	NEU3	Neu3	LOC101400456	TMCC1	Tmcc1	LOC101395038
DEFB103B	Defb14	LOC101397209	NEU4	Neu4	LOC101392788	TMCC2	Tmcc2	LOC101399895
DEFB107A	Defb13	LOC106803546	NEURL1	Neurl1a	LOC101398600	TMCC3	Tmcc3	LOC101387170
DEFB116	Defb29	LOC101397634	NEURL1B	Neurl1b	LOC101392271	TMCO1	Tmco1	LOC101401470
DEFB123	Defb36	LOC106803887	NEURL2	Neurl2	LOC101404366	TMCO2	Tmco2	LOC101407408
DEFB129	Defb23	LOC101388174	NEURL3	Neurl3	LOC101402035	TMCO3	Tmco3	LOC101390968
DEGS1	Degs1	LOC101405108	NEURL4	Neurl4	LOC101407170	TMCO4	Tmco4	LOC101389347
DEK	Dek	LOC101399220	NEUROD1	Neurod1	LOC101389100	TMCO5A	Tmco5	LOC101400752
DELE1	0610009O20R	LOC101405820	NEUROD2	Neurod2	LOC101404734	TMCO6	Tmco6	LOC101391966
DENND10	Fam45a	LOC101396446	NEUROD4	Neurod4	LOC101400440	TMED1	Tmed1	LOC101391212
DENND11	E330009J07R	LOC101407162	NEUROD6	Neurod6	LOC101405908	TMED10	Tmed10	LOC101387630
DENND1A	Dennd1a	LOC101398174	NEUROG1	Neurog1	LOC101391621	TMED2	Tmed2	LOC101393974
DENND1B	Dennd1b	LOC101406412	NEUROG3	Neurog3	LOC101405599	TMED3	Tmed3	LOC101388380
DENND1C	Dennd1c	LOC101404409	NEXN	Nexn	LOC101402612	TMED4	Tmed4	LOC101389827
DENND2A	Dennd2a	LOC101408991	NF1	Nf1	LOC101390113	TMED5	Tmed5	LOC101406091
DENND2B	St5	LOC101390182	NF2	Nf2	LOC101402015	TMED6	Tmed6	LOC101403845
DENND2C	Dennd2c	LOC101404068	NFAM1	Nfam1	LOC101396661	TMED7	Tmed7	LOC101398156
DENND2D	Dennd2d	LOC101392846	NFASC	Nfasc	LOC101401039	TMED8	Tmed8	LOC101392609
DENND3	Dennd3	LOC101408560	NFAT5	Nfat5	LOC101404374	TMED9	Tmed9	LOC101398356
DENND4A	Dennd4a	LOC101393859	NFATC1	Nfatc1	LOC101390638	TMEFF1	Tmeff1	LOC101394124
DENND4B	Dennd4b	LOC101396567	NFATC2	Nfatc2	LOC101390238	TMEFF2	Tmeff2	LOC101401399
DENND4C	Dennd4c	LOC101404659	NFATC2IP	Nfatc2ip	LOC101404592	TMEM100	Tmem100	LOC101400186
DENND5A	Dennd5a	LOC101398637	NFATC3	Nfatc3	LOC101398115	TMEM101	Tmem101	LOC101390250
DENND5B	Dennd5b	LOC101408864	NFATC4	Nfatc4	LOC101390795	TMEM102	Tmem102	LOC101404728
DENND6A	Dennd6a	LOC101393500	NFE2	Nfe2	LOC101396279	TMEM104	Tmem104	LOC101387468
DENND6B	Dennd6b	LOC101402123	NFE2L1	Nfe2l1	LOC101393735	TMEM106A	Tmem106a	LOC101408572
DENR	Denr	LOC101389208	NFE2L2	Nfe2l2	LOC101402785	TMEM106B	Tmem106b	LOC101389803
DEPDC1B	Depdc1b	LOC101387528	NFE2L3	Nfe2l3	LOC101408074	TMEM106C	Tmem106c	LOC101390913
DEPDC5	Depdc5	LOC101408735	NFIA	Nfia	LOC101392442	TMEM107	Tmem107	LOC101393902
DEPDC7	Depdc7	LOC101402038	NFIB	Nfib	LOC101387923	TMEM108	Tmem108	LOC101391531
DEPTOR	Deptor	LOC101402531	NFIC	Nfic	LOC101399777	TMEM109	Tmem109	LOC101387828
DERA	Dera	LOC101404308	NFIL3	Nfil3	LOC101404890	TMEM11	Tmem11	LOC101387993
DERL1	Derl1	LOC101401063	NFIX	Nfix	LOC101408001	TMEM114	Tmem114	LOC101388873
DERL2	Derl2	LOC101403069	NFKB1	Nfkb1	LOC101397259	TMEM115	Tmem115	LOC101391962
DERL3	Derl3	LOC101393866	NFKB2	Nfkb2	LOC101390320	TMEM116	Tmem116	LOC101395676
DES	Des	LOC101396715	NFKBIA	Nfkbia	LOC101389998	TMEM117	Tmem117	LOC101408385
DESI1	Desi1	LOC101406832	NFKBIB	Nfkbib	LOC101407211	TMEM119	Tmem119	LOC101393457
DESI2	Desi2	LOC101395714	NFKBID	Nfkbid	LOC101399854	TMEM120A	Tmem120a	LOC101404866
DET1	Det1	LOC101399485	NFKBIE	Nfkbie	LOC101395134	TMEM120B	Tmem120b	LOC101405419
DEUP1	Ccdc67	LOC101398860	NFKBIL1	Nfkbil1	LOC101400686	TMEM121	Tmem121	LOC101407530
DEXI	Dexi	LOC101407899	NFKBIZ	Nfkbiz	LOC101389653	TMEM123	Tmem123	LOC101392166
DFFA	Dffa	LOC101404183	NFRKB	Nfrkb	LOC101405799	TMEM125	Tmem125	LOC101404253
DFFB	Dffb	LOC101399980	NFS1	Nfs1	LOC101390750	TMEM126A	Tmem126a	LOC101392550
DGAT1	Dgat1	LOC101390298	NFU1	Nfu1	LOC101402545	TMEM126B	Tmem126b	LOC101392285
DGAT2	Dgat2	LOC101403595	NFX1	Nfx1	LOC101407606	TMEM127	Tmem127	LOC101397862
DGAT2L6	Dgat2l6	LOC101398461	NFXL1	Nfxl1	LOC101406165	TMEM128	Tmem128	LOC101408135

DGCR2	Dgcr2	LOC101407390	NFYA	Nfya	LOC101395399	TMEM129	Tmem129	LOC101393898
DGCR8	Dgcr8	LOC101392590	NFYB	Nfyb	LOC101405902	TMEM130	Tmem130	LOC101407290
DGKA	Dgka	LOC101395332	NFYC	Nfyc	LOC101388233	TMEM131	Tmem131	LOC101403338
DGKB	Dgkb	LOC101392093	NGB	Ngb	LOC101393368	TMEM132A	Tmem132a	LOC101388096
DGKD	Dgkd	LOC101398679	NGDN	Ngdn	LOC101402149	TMEM132B	Tmem132b	LOC101404097
DGKE	Dgke	LOC101401948	NGEF	Ngef	LOC101396716	TMEM132C	Tmem132c	LOC101404895
DGKG	Dgkg	LOC101406114	NGF	Ngf	LOC101400661	TMEM132D	Tmem132d	LOC101398018
DGKH	Dgkh	LOC101403071	NGFR	Ngfr	LOC101402811	TMEM132E	Tmem132e	LOC101404414
DGKI	Dgki	LOC101396018	NGLY1	Ngly1	LOC101407115	TMEM134	Tmem134	LOC101407098
DGKK	Dgkk	LOC101400463	NGRN	Ngrn	LOC101406396	TMEM135	Tmem135	LOC101398877
DGKQ	Dgkq	LOC101398785	NHLH1	Nhlh1	LOC101393607	TMEM138	Tmem138	LOC101391435
DGKZ	Dgkz	LOC101395266	NHLH2	Nhlh2	LOC101399530	TMEM140	Tmem140	LOC101398433
DGLUCY	9030617O03I	LOC101404831	NHLRC1	Nhlrc1	LOC101400360	TMEM141	Tmem141	LOC101390973
DGUOK	Dguok	LOC101408412	NHLRC2	Nhlrc2	LOC101389599	TMEM143	Tmem143	LOC101390613
DHCR24	Dhcr24	LOC101397101	NHLRC3	Nhlrc3	LOC101393114	TMEM144	Tmem144	LOC101391703
DHCR7	Dhcr7	LOC101400564	NHLRC4	Nhlrc4	LOC106803063	TMEM145	Tmem145	LOC101402915
DHDDS	Dhdds	LOC101394979	NHP2	Nhp2	LOC101397325	TMEM147	Tmem147	LOC101393169
DHDH	Dhdh	LOC101397369	NHS	Nhs	LOC101391257	TMEM14A	Tmem14a	LOC101394894
DHFR	Dhfr	LOC101403719	NHSL1	Nhsl1	LOC101389180	TMEM150A	Tmem150a	LOC101389639
DHH	Dhh	LOC101401141	NHSL2	Nhsl2	LOC101400913	TMEM150B	Tmem150b	LOC101390524
DHODH	Dhodh	LOC101407420	NIBAN1	Fam129a	LOC101397999	TMEM150C	Tmem150c	LOC101394065
DHPS	Dhps	LOC101401378	NIBAN2	Fam129b	LOC101387278	TMEM151A	Tmem151a	LOC101394088
DHRS1	Dhrs1	LOC101393008	NIBAN3	Fam129c	LOC101401647	TMEM151B	Tmem151b	LOC101399282
DHRS11	Dhrs11	LOC101397186	NICN1	Nicn1	LOC101403450	TMEM154	Tmem154	LOC101392143
DHRS13	Dhrs13	LOC101399828	NID1	Nid1	LOC101393816	TMEM156	Tmem156	LOC101392311
DHRS3	Dhrs3	LOC101397051	NID2	Nid2	LOC101393657	TMEM159	Tmem159	LOC101395819
DHRS7	Dhrs7	LOC101394293	NIF3L1	Nif3l1	LOC101388238	TMEM160	Tmem160	LOC101405553
DHRS7B	Dhrs7b	LOC101388423	NIFK	Nifk	LOC101396883	TMEM161A	Tmem161a	LOC101398490
DHRS7C	Dhrs7c	LOC101408996	NIM1K	Nim1k	LOC101392763	TMEM161B	Tmem161b	LOC101397627
DHRS9	Dhrs9	LOC101403760	NIN	Nin	LOC101397606	TMEM163	Tmem163	LOC101387882
DHTKD1	Dhtkd1	LOC101396967	NINL	Ninl	LOC101394443	TMEM164	Tmem164	LOC101395456
DHX15	Dhx15	LOC101396292	NIP7	Nip7	LOC101403411	TMEM165	Tmem165	LOC101391878
DHX16	Dhx16	LOC101407756	NIPA1	Nipa1	LOC101392329	TMEM167A	Tmem167	LOC101397721
DHX29	Dhx29	LOC101401296	NIPA2	Nipa2	LOC101391547	TMEM167B	Tmem167b	LOC101406428
DHX30	Dhx30	LOC101387847	NIPAL1	Nipal1	LOC101406680	TMEM168	Tmem168	LOC101394341
DHX32	Dhx32	LOC101399936	NIPAL2	Nipal2	LOC101405769	TMEM169	Tmem169	LOC101403492
DHX33	Dhx33	LOC101402804	NIPAL3	Nipal3	LOC101408206	TMEM17	Tmem17	LOC101391856
DHX34	Dhx34	LOC101407011	NIPAL4	Nipal4	LOC101396449	TMEM170B	Tmem170b	LOC101406989
DHX35	Dhx35	LOC101399039	NIPBL	Nipbl	LOC101406005	TMEM171	Tmem171	LOC101394185
DHX36	Dhx36	LOC101407922	NIPSNAP1	Nipsnap1	LOC101403580	TMEM174	Tmem174	LOC101400570
DHX37	Dhx37	LOC101403837	NIPSNAP2	Gbas	LOC101387415	TMEM175	Tmem175	LOC101391329
DHX38	Dhx38	LOC101406904	NIPSNAP3A	Nipsnap3a	LOC101404273	TMEM176A	Tmem176a	LOC101389461
DHX40	Dhx40	LOC101389389	NIPSNAP3B	Nipsnap3b	LOC101404878	TMEM176B	Tmem176b	LOC101389709
DHX57	Dhx57	LOC101408417	NISCH	Nisch	LOC101403184	TMEM178B	Tmem178b	LOC101407695
DHX58	Dhx58	LOC101401247	NIT1	Nit1	LOC101394533	TMEM179	Tmem179	LOC101401764
DHX8	Dhx8	LOC101408825	NIT2	Nit2	LOC101408168	TMEM179B	Tmem179b	LOC101402900
DHX9	Dhx9	LOC101402949	NKAIN1	Nkain1	LOC101397318	TMEM18	Tmem18	LOC101392023
DIABLO	Diablo	LOC101408478	NKAIN2	Nkain2	LOC101392334	TMEM182	Tmem182	LOC101389635
DIAPH1	Diaph1	LOC101407382	NKAIN3	Nkain3	LOC101406573	TMEM183A	Tmem183a	LOC101395273
DIAPH2	Diaph2	LOC101405375	NKAIN4	Nkain4	LOC101403056	TMEM184A	Tmem184a	LOC101397625
DIAPH3	Diaph3	LOC101397938	NKAP	Nkap	LOC101400668	TMEM184B	Tmem184b	LOC101391868
DICER1	Dicer1	LOC101408141	NKAPL	Nkapl	LOC101389668	TMEM184C	Tmem184c	LOC101399263
DIDO1	Dido1	LOC101403582	NKD1	Nkd1	LOC101395420	TMEM185B	Tmem185b	LOC101395313
DIMT1	Dimt1	LOC101389771	NKD2	Nkd2	LOC101395022	TMEM186	Tmem186	LOC101388874

DIO1	Dio1	LOC101391946	NKG7	Nkg7	LOC101404533	TMEM189	Tmem189	LOC101388495
DIO2	Dio2	LOC101398596	NKIRAS1	Nkiras1	LOC101405116	TMEM19	Tmem19	LOC101398015
DIO3	Dio3	LOC101391086	NKIRAS2	Nkiras2	LOC101401509	TMEM190	Tmem190	LOC101392202
DIP2A	Dip2a	LOC101406897	NKPD1	Nkpd1	LOC101406849	TMEM192	Tmem192	LOC101389810
DIP2B	Dip2b	LOC101388669	NKRF	Nkrf	LOC101397129	TMEM196	Tmem196	LOC101405112
DIP2C	Dip2c	LOC101387373	NKTR	Nktr	LOC101392044	TMEM198	Tmem198	LOC101398678
DIPK1A	Fam69a	LOC101407831	NKX1-2	Nkx1-2	LOC101392646	TMEM199	Tmem199	LOC101399314
DIPK1B	Fam69b	LOC101401303	NKX2-1	Nkx2-1	LOC101387485	TMEM201	Tmem201	LOC101403394
DIPK1C	Fam69c	LOC101389584	NKX2-2	Nkx2-2	LOC101400664	TMEM202	Tmem202	LOC101391137
DIPK2A	1190002N15I	LOC101394348	NKX2-3	Nkx2-3	LOC101409156	TMEM203	Tmem203	LOC101390725
DIPK2B	4930578C19I	LOC101400271	NKX2-5	Nkx2-5	LOC101390321	TMEM204	Tmem204	LOC101394756
DIRAS1	Diras1	LOC101403718	NKX2-6	Nkx2-6	LOC106802317	TMEM205	Tmem205	LOC101393603
DIRAS2	Diras2	LOC101403761	NKX2-8	Nkx2-9	LOC101387237	TMEM207	Tmem207	LOC101407594
DIS3	Dis3	LOC101407590	NKX3-1	Nkx3-1	LOC106802318	TMEM208	Tmem208	LOC101388945
DIS3L	Dis3l	LOC101394788	NKX3-2	Nkx3-2	LOC101387648	TMEM209	Tmem209	LOC101389489
DIS3L2	Dis3l2	LOC101393545	NKX6-1	Nkx6-1	LOC101397930	TMEM210	Tmem210	LOC101393032
DISC1	Disc1	LOC101399136	NKX6-2	Nkx6-2	LOC101395184	TMEM211	Tmem211	LOC101390660
DISP1	Disp1	LOC101400026	NKX6-3	Nkx6-3	LOC101400302	TMEM212	Tmem212	LOC101389343
DISP2	Disp2	LOC101390200	NLE1	Nle1	LOC101401799	TMEM213	Tmem213	LOC101390495
DIXDC1	Dixdc1	LOC101394297	NLGN1	Nlgn1	LOC101409047	TMEM214	Tmem214	LOC101387676
DKC1	Dkc1	LOC101401728	NLGN2	Nlgn2	LOC101405427	TMEM215	Tmem215	LOC101394469
DKK1	Dkk1	LOC101402309	NLGN3	Nlgn3	LOC101405191	TMEM216	Tmem216	LOC101392300
DKK2	Dkk2	LOC101404885	NLK	Nlk	LOC101400800	TMEM217	Tmem217	LOC106800867
DKK3	Dkk3	LOC101397285	NLN	Nln	LOC101393438	TMEM218	Tmem218	LOC101395875
DKK4	Dkk4	LOC101393002	NLRC3	Nlrc3	LOC101399152	TMEM219	Tmem219	LOC101391950
DKKL1	Dkkl1	LOC101402397	NLRC4	Nlrc4	LOC101389554	TMEM220	Tmem220	LOC101406313
DLAT	Dlat	LOC101393631	NLRC5	Nlrc5	LOC101396992	TMEM221	Tmem221	LOC101402158
DLC1	Dlc1	LOC101398187	NLRP12	Nlrp12	LOC101404321	TMEM222	Tmem222	LOC101400338
DLD	Dld	LOC101403443	NLRP14	Nlrp14	LOC101404326	TMEM223	Tmem223	LOC101403167
DLEC1	Dlec1	LOC101401629	NLRP3	Nlrp3	LOC101399867	TMEM225	Tmem225	LOC101402174
DLEU7	Dleu7	LOC101394836	NLRP5	Nlrp5	LOC101397954	TMEM230	Tmem230	LOC101402806
DLG1	Dlg1	LOC101389967	NLRP6	Nlrp6	LOC101405199	TMEM231	Tmem231	LOC101404837
DLG2	Dlg2	LOC101391003	NLRX1	Nlrx1	LOC101388830	TMEM232	Tmem232	LOC101393854
DLG3	Dlg3	LOC101408248	NMB	Nmb	LOC101388847	TMEM233	Tmem233	LOC101407161
DLG4	Dlg4	LOC101389127	NMBR	Nmbr	LOC101394700	TMEM234	Tmem234	LOC101399027
DLG5	Dlg5	LOC101398676	NMD3	Nmd3	LOC101401826	TMEM236	Tmem236	LOC101399024
DLGAP1	Dlgap1	LOC101401798	NME2	Nme2	LOC101396736	TMEM237	Tmem237	LOC101390820
DLGAP2	Dlgap2	LOC101399693	NME3	Nme3	LOC101397356	TMEM239	Tmem239	LOC101390925
DLGAP3	Dlgap3	LOC101408889	NME4	Nme4	LOC101404842	TMEM240	Tmem240	LOC101391823
DLGAP4	Dlgap4	LOC101387459	NME5	Nme5	LOC101407563	TMEM241	Tmem241	LOC106800319
DLGAP5	Dlgap5	LOC101405405	NME6	Nme6	LOC101389418	TMEM242	Tmem242	LOC101387848
DLK1	Dlk1	LOC101390675	NME7	Nme7	LOC101409048	TMEM243	Tmem243	LOC101390150
DLK2	Dlk2	LOC101402259	NME8	Nme8	LOC101397537	TMEM245	Tmem245	LOC101408460
DLL1	Dll1	LOC101404162	NME9	Nme9	LOC101404602	TMEM247	Tmem247	LOC101387744
DLL3	Dll3	LOC101408866	NMI	Nmi	LOC101401304	TMEM249	Gm8140	LOC101402500
DLL4	Dll4	LOC101395121	NMNAT1	Nmnat1	LOC101387201	TMEM25	Tmem25	LOC101408119
DLST	Dlst	LOC101407597	NMNAT2	Nmnat2	LOC101401911	TMEM251	Tmem251	LOC101402543
DLX1	Dlx1	LOC101395668	NMNAT3	Nmnat3	LOC101391787	TMEM252	Tmem252	LOC101400029
DLX2	Dlx2	LOC101395408	NMRAL1	Nmral1	LOC101395528	TMEM253	Tmem253	LOC101400044
DLX3	Dlx3	LOC101406142	NMRK1	Nmrk1	LOC101408336	TMEM255A	Tmem255a	LOC101399675
DLX4	Dlx4	LOC101405881	NMRK2	Nmrk2	LOC101397294	TMEM255B	Tmem255b	LOC101404170
DLX5	Dlx5	LOC101406987	NMS	Nms	LOC101404657	TMEM256	Tmem256	LOC101405688
DLX6	Dlx6	LOC101406726	NMT1	Nmt1	LOC101400002	TMEM258	Tmem258	LOC101394166
DMAC1	Tmem261	LOC101400543	NMT2	Nmt2	LOC101400068	TMEM259	Tmem259	LOC101389246

DMAP1	Dmap1	LOC101389670	NMU	Nmu	LOC101392477	TMEM26	Tmem26	LOC101397916
DMBX1	Dmbx1	LOC101402242	NMUR2	Nmur2	LOC101394139	TMEM260	Tmem260	LOC101406971
DMC1	Dmc1	LOC101393480	NNAT	Nnat	LOC101403406	TMEM265	Tmem265	LOC101400024
DMD	Dmd	LOC101392552	NNMT	Nnmt	LOC101387547	TMEM266	Tmem266	LOC101403632
DMGDH	Dmgdh	LOC101408178	NNT	Nnt	LOC101394795	TMEM270	Wbscr28	LOC101399872
DMKN	Dmkn	LOC101392577	NOA1	Noa1	LOC101395883	TMEM273	1810011H11I	LOC101405171
DMP1	Dmp1	LOC101403233	NOB1	Nob1	LOC101405254	TMEM30A	Tmem30a	LOC101401644
DMPK	Dmpk	LOC101400392	NOBOX	Nobox	LOC101393056	TMEM30B	Tmem30b	LOC101396438
DMRT1	Dmrt1	LOC101394016	NOC2L	Noc2l	LOC101388059	TMEM33	Tmem33	LOC101400391
DMRT2	Dmrt2	LOC101400832	NOC3L	Noc3l	LOC101401402	TMEM35A	Tmem35	LOC101401365
DMRT3	Dmrt3	LOC101394260	NOC4L	Noc4l	LOC101400791	TMEM35B	Gm12942	LOC101399118
DMRTA1	Dmrta1	LOC101400246	NOCT	Noct	LOC101400431	TMEM37	Tmem37	LOC101389447
DMRTA2	Dmrta2	LOC101402639	NOD1	Nod1	LOC101387179	TMEM38A	Tmem38a	LOC101398845
DMRTB1	Dmrtb1	LOC101390269	NOD2	Nod2	LOC101403234	TMEM38B	Tmem38b	LOC101406012
DMRTC1	Dmrtc1a	LOC101398750	NODAL	Nodal	LOC101402521	TMEM39A	Tmem39a	LOC101406331
DMRTC2	Dmrtc2	LOC101408169	NOG	Nog	LOC101401420	TMEM39B	Tmem39b	LOC101397156
DMTF1	Dmtf1	LOC101389890	NOL10	Nol10	LOC101407716	TMEM40	Tmem40	LOC101392599
DMTN	Dmtn	LOC101408094	NOL11	Nol11	LOC101396906	TMEM41A	Tmem41a	LOC101408886
DMWD	Dmwd	LOC101387084	NOL12	Nol12	LOC101393090	TMEM41B	Tmem41b	LOC101392469
DMXL1	Dmxl1	LOC101395115	NOL3	Nol3	LOC101408912	TMEM42	Tmem42	LOC101398720
DMXL2	Dmxl2	LOC101407465	NOL4	Nol4	LOC101393953	TMEM43	Tmem43	LOC101389507
DNA2	Dna2	LOC101393800	NOL4L	Nol4l	LOC101387862	TMEM44	Tmem44	LOC101393444
DNAAF1	Dnaaf1	LOC101394754	NOL6	Nol6	LOC101390910	TMEM45A	Tmem45a	LOC101408939
DNAAF2	Dnaaf2	LOC101395703	NOL7	Nol7	LOC101403940	TMEM45B	Tmem45b	LOC101405539
DNAAF3	Dnaaf3	LOC101389742	NOL8	Nol8	LOC101395559	TMEM47	Tmem47	LOC101391593
DNAAF4	Dyx1c1	LOC101390629	NOL9	Nol9	LOC106801105	TMEM50A	Tmem50a	LOC101388662
DNAAF5	Dnaaf5	LOC101408342	NOLC1	Nolc1	LOC101389278	TMEM50B	Tmem50b	LOC101406457
DNAH1	Dnah1	LOC101388892	NOM1	Nom1	LOC101405338	TMEM51	Tmem51	LOC101398094
DNAH10	Dnah10	LOC101402793	NOMO3	Nomo1	LOC101394357	TMEM52	Tmem52	LOC101397157
DNAH11	Dnah11	LOC101403097	NONO	Nono	LOC101403530	TMEM52B	Tmem52b	LOC101403609
DNAH12	Dnah12	LOC101390194	NOP10	Nop10	LOC101401375	TMEM53	Tmem53	LOC101390625
DNAH14	Dnah14	LOC101397286	NOP14	Nop14	LOC101399135	TMEM54	Tmem54	LOC101404546
DNAH17	Dnah17	LOC101389218	NOP16	Nop16	LOC101408551	TMEM59	Tmem59	LOC101393083
DNAH2	Dnah2	LOC101398789	NOP53	Gltscr2	LOC101409027	TMEM59L	Tmem59l	LOC101408533
DNAH3	Dnah3	LOC101399706	NOP56	Nop56	LOC101392284	TMEM60	Tmem60	LOC101391949
DNAH5	Dnah5	LOC101396631	NOP58	Nop58	LOC101393130	TMEM61	Tmem61	LOC101404492
DNAH6	Dnah6	LOC101408324	NOP9	Nop9	LOC101392750	TMEM62	Tmem62	LOC101405573
DNAH7	Dnah7a	LOC101402269	NOS1	Nos1	LOC101387723	TMEM63A	Tmem63a	LOC101407199
DNAH8	Dnah8	LOC101398851	NOS1AP	Nos1ap	LOC101395489	TMEM63B	Tmem63b	LOC101397311
DNAH9	Dnah9	LOC101406046	NOS2	Nos2	LOC101401500	TMEM63C	Tmem63c	LOC101392081
DNAI1	Dnaic1	LOC101387128	NOS3	Nos3	LOC101388496	TMEM64	Tmem64	LOC101394916
DNAI2	Dnaic2	LOC101393227	NOSIP	Nosip	LOC101406491	TMEM65	Tmem65	LOC101396808
DNAJA1	Dnaja1	LOC101393298	NOSTRIN	Nostrin	LOC101404966	TMEM67	Tmem67	LOC101397847
DNAJA2	Dnaja2	LOC101402852	NOTCH1	Notch1	LOC101402423	TMEM68	Tmem68	LOC101391510
DNAJA3	Dnaja3	LOC101395789	NOTCH2	Notch2	LOC101392918	TMEM69	Tmem69	LOC101398329
DNAJA4	Dnaja4	LOC101406951	NOTCH3	Notch3	LOC101394531	TMEM71	Tmem71	LOC101387726
DNAJB11	Dnajb11	LOC101404952	NOTCH4	Notch4	LOC101407755	TMEM72	Tmem72	LOC101398528
DNAJB12	Dnajb12	LOC101396630	NOTO	Noto	LOC101390680	TMEM74	Tmem74	LOC101397769
DNAJB13	Dnajb13	LOC101396030	NOTUM	Notum	LOC101404111	TMEM79	Tmem79	LOC101405183
DNAJB14	Dnajb14	LOC101395599	NOVA1	Nova1	LOC101409035	TMEM80	Tmem80	LOC101408863
DNAJB2	Dnajb2	LOC101395326	NOVA2	Nova2	LOC101402137	TMEM81	Tmem81	LOC101403213
DNAJB4	Dnajb4	LOC101403674	NOX1	Nox1	LOC101402142	TMEM82	Tmem82	LOC101399640
DNAJB5	Dnajb5	LOC101406618	NOX3	Nox3	LOC101387335	TMEM86A	Tmem86a	LOC101391361
DNAJB6	Dnajb6	LOC101400792	NOX4	Nox4	LOC101400978	TMEM86B	Tmem86b	LOC101390273

DNAJB7	Dnajb7	LOC101402635	NOXA1	Noxa1	LOC101394541	TMEM87A	Tmem87a	LOC101401719
DNAJB9	Dnajb9	LOC101396844	NOXO1	Noxo1	LOC101396311	TMEM87B	Tmem87b	LOC101391943
DNAJC1	Dnajc1	LOC101397242	NOXRED1	Noxred1	LOC101394718	TMEM88	Tmem88	LOC101398532
DNAJC10	Dnajc10	LOC101406450	NPAS1	Npas1	LOC101405287	TMEM89	Tmem89	LOC101392913
DNAJC11	Dnajc11	LOC101403749	NPAS2	Npas2	LOC101407973	TMEM8B	Tmem8b	LOC101390462
DNAJC12	Dnajc12	LOC101393133	NPAS3	Npas3	LOC101394964	TMEM9	Tmem9	LOC101402475
DNAJC13	Dnajc13	LOC101392734	NPAS4	Npas4	LOC101395618	TMEM91	Tmem91	LOC101402145
DNAJC14	Dnajc14	LOC101394141	NPAT	Npat	LOC101401921	TMEM94	Tmem94	LOC101394739
DNAJC15	Dnajc15	LOC101404650	NPB	Npb	LOC101403849	TMEM95	Tmem95	LOC106802264
DNAJC16	Dnajc16	LOC101387540	NPBWR1	Npbwr1	LOC101387919	TMEM97	Tmem97	LOC101400530
DNAJC17	Dnajc17	LOC101393350	NPC1	Npc1	LOC101388987	TMEM98	Tmem98	LOC101406762
DNAJC18	Dnajc18	LOC101398990	NPC1L1	Npc1l1	LOC101389550	TMF1	Tmf1	LOC101406085
DNAJC19	Dnajc19	LOC101401128	NPC2	Npc2	LOC101405751	TMIE	Tmie	LOC101405290
DNAJC2	Dnajc2	LOC101389648	NPDC1	Npdc1	LOC101388312	TMIGD1	Tmigd1	LOC106802298
DNAJC21	Dnajc21	LOC101400228	NPEPL1	Npepl1	LOC101404199	TMLHE	Tmlhe	LOC101407587
DNAJC22	Dnajc22	LOC101403764	NPFF	Npff	LOC101391239	TMOD1	Tmod1	LOC101387879
DNAJC24	Dnajc24	LOC101407009	NPFFR1	Npffr1	LOC101392786	TMOD2	Tmod2	LOC101408957
DNAJC25	Dnajc25	LOC101391055	NPFFR2	Npffr2	LOC101391366	TMOD3	Tmod3	LOC101387268
DNAJC27	Dnajc27	LOC101394252	NPHP1	Nphp1	LOC101393830	TMOD4	Tmod4	LOC101399671
DNAJC28	Dnajc28	LOC101406720	NPHP3	Nphp3	LOC101394106	TMPO	Tmpo	LOC101392572
DNAJC3	Dnajc3	LOC101396518	NPHP4	Nphp4	LOC101400241	TMPPE	Tmppe	LOC101393497
DNAJC30	Dnajc30	LOC101401455	NPHS1	Nphs1	LOC101389238	TMPRSS11A	Tmprss11a	LOC101397019
DNAJC4	Dnajc4	LOC101390598	NPHS2	Nphs2	LOC101388563	TMPRSS11D	Tmprss11d	LOC101396761
DNAJC5	Dnajc5	LOC101390411	NPL	Npl	LOC101403391	TMPRSS11E	Tmprss11e	LOC101401002
DNAJC5B	Dnajc5b	LOC101398374	NPLOC4	Nploc4	LOC101396026	TMPRSS11F	Tmprss11f	LOC101400742
DNAJC6	Dnajc6	LOC101408821	NPM1	Npm1	LOC101393804	TMPRSS12	Tmprss12	LOC101389703
DNAJC7	Dnajc7	LOC101402112	NPM2	Npm2	LOC101387516	TMPRSS13	Tmprss13	LOC101408895
DNAJC8	Dnajc8	LOC101406885	NPM3	Npm3	LOC101407772	TMPRSS15	Tmprss15	LOC101389799
DNAJC9	Dnajc9	LOC101393132	NPNT	Npnt	LOC101393370	TMPRSS2	Tmprss2	LOC101401409
DNAL1	Dnal1	LOC101401133	NPPA	Nppa	LOC101395578	TMPRSS3	Tmprss3	LOC101398431
DNAL4	Dnal4	LOC101395788	NPPB	Nppb	LOC101405231	TMPRSS4	Tmprss4	LOC101400953
DNALI1	Dnali1	LOC101396006	NPPC	Nppc	LOC101393136	TMPRSS5	Tmprss5	LOC101388323
DNASE1	Dnase1	LOC101398626	NPR1	Npr1	LOC101395782	TMPRSS6	Tmprss6	LOC101398711
DNASE1L1	Dnase1l1	LOC101394709	NPR2	Npr2	LOC101389946	TMPRSS7	Tmprss7	LOC101404577
DNASE1L2	Dnase1l2	LOC101408932	NPR3	Npr3	LOC101397041	TMPRSS9	Tmprss9	LOC101405031
DNASE1L3	Dnase1l3	LOC101395546	NPRL2	Nprl2	LOC101391697	TMSB10	Tmsb10	LOC101409009
DNASE2	Dnase2a	LOC101405576	NPRL3	Nprl3	LOC101407445	TMSB15A	Tmsb15a	LOC101408619
DNASE2B	Dnase2b	LOC101408044	NPS	Nps	LOC101398978	TMSB4X	Tmsb4x	LOC101388769
DND1	Dnd1	LOC101391031	NPSR1	Npsr1	LOC101401185	TMTC1	Tmtc1	LOC101406248
DNER	Dner	LOC101401477	NPTN	Nptn	LOC101387102	TMTC2	Tmtc2	LOC101398896
DNHD1	Dnhd1	LOC101389652	NPTX1	Nptx1	LOC101392966	TMTC3	Tmtc3	LOC101401527
DNLZ	Dnlz	LOC106800739	NPTX2	Nptx2	LOC101401009	TMTC4	Tmtc4	LOC101403382
DNM1	Dnm1	LOC101394205	NPTXR	Nptxr	LOC101389739	TMUB1	Tmub1	LOC101408125
DNM1L	Dnm1l	LOC101402658	NPVF	Npvf	LOC101396238	TMUB2	Tmub2	LOC101391665
DNM2	Dnm2	LOC101403890	NPY	Npy	LOC101398462	TMX1	Tmx1	LOC101396479
DNM3	Dnm3	LOC101401912	NPY1R	Npy1r	LOC101391292	TMX2	Tmx2	LOC101391190
DNMBP	Dnmbp	LOC101398680	NPY2R	Npy2r	LOC101387693	TMX3	Tmx3	LOC101393522
DNMT1	Dnmt1	LOC101408095	NQO1	Nqo1	LOC101404982	TMX4	Tmx4	LOC101399490
DNMT3A	Dnmt3a	LOC101393486	NQO2	Nqo2	LOC101395683	TNC	Tnc	LOC101388224
DNMT3B	Dnmt3b	LOC101409038	NR0B1	Nr0b1	LOC101395608	TNF	Tnf	LOC101400154
DNMT3L	Dnmt3l	LOC101405076	NR0B2	Nr0b2	LOC101398760	TNFAIP1	Tnfaip1	LOC101400005
DNPEP	Dnpep	LOC101396443	NR1D1	Nr1d1	LOC101397518	TNFAIP2	Tnfaip2	LOC101390507
DNPH1	Dnph1	LOC101404084	NR1D2	Nr1d2	LOC101405639	TNFAIP3	Tnfaip3	LOC101388387
DNTT	Dntt	LOC101409154	NR1H2	Nr1h2	LOC101390188	TNFAIP6	Tnfaip6	LOC101400769

DNTTIP1	Dnttip1	LOC101407066	NR1H3	Nr1h3	LOC101402631	TNFAIP8	Tnfaip8	LOC101392587
DNTTIP2	Dnttip2	LOC101403460	NR1H4	Nr1h4	LOC101398710	TNFAIP8L1	Tnfaip8l1	LOC101392403
DOC2A	Doc2a	LOC101390607	NR1I2	Nr1i2	LOC101400895	TNFAIP8L2	Tnfaip8l2	LOC101398698
DOC2B	Doc2b	LOC101405615	NR1I3	Nr1i3	LOC101398489	TNFAIP8L3	Tnfaip8l3	LOC101405997
DOCK1	Dock1	LOC101390352	NR2C1	Nr2c1	LOC101387836	TNFRSF11A	Tnfrsf11a	LOC101408878
DOCK10	Dock10	LOC101405755	NR2C2	Nr2c2	LOC101396512	TNFRSF11B	Tnfrsf11b	LOC101404463
DOCK11	Dock11	LOC101394446	NR2C2AP	Nr2c2ap	LOC101403998	TNFRSF12A	Tnfrsf12a	LOC101404319
DOCK2	Dock2	LOC101397497	NR2E1	Nr2e1	LOC101390960	TNFRSF13C	Tnfrsf13c	LOC101408326
DOCK3	Dock3	LOC101387091	NR2E3	Nr2e3	LOC101405132	TNFRSF14	Tnfrsf14	LOC101398937
DOCK4	Dock4	LOC101396056	NR2F1	Nr2f1	LOC101394354	TNFRSF17	Tnfrsf17	LOC101408933
DOCK5	Dock5	LOC101408050	NR2F2	Nr2f2	LOC101394736	TNFRSF18	Tnfrsf18	LOC101389095
DOCK6	Dock6	LOC101393109	NR2F6	Nr2f6	LOC101396877	TNFRSF19	Tnfrsf19	LOC101403687
DOCK7	Dock7	LOC101404822	NR3C1	Nr3c1	LOC101403010	TNFRSF1A	Tnfrsf1a	LOC101388013
DOCK8	Dock8	LOC101393173	NR3C2	Nr3c2	LOC101400406	TNFRSF1B	Tnfrsf1b	LOC101406284
DOCK9	Dock9	LOC101399276	NR4A1	Nr4a1	LOC101397922	TNFRSF21	Tnfrsf21	LOC101389692
DOHH	Dohh	LOC101401978	NR4A2	Nr4a2	LOC101401219	TNFRSF25	Tnfrsf25	LOC101401316
DOK1	Dok1	LOC101402466	NR4A3	Nr4a3	LOC101392338	TNFRSF4	Tnfrsf4	LOC101388825
DOK2	Dok2	LOC101388038	NR5A1	Nr5a1	LOC101399368	TNFRSF8	Tnfrsf8	LOC101406020
DOK3	Dok3	LOC101398863	NR5A2	Nr5a2	LOC101404493	TNFRSF9	Tnfrsf9	LOC101404444
DOK4	Dok4	LOC101394479	NR6A1	Nr6a1	LOC101399629	TNFSF10	Tnfsf10	LOC101389342
DOK5	Dok5	LOC101387135	NRAP	Nrap	LOC101388243	TNFSF11	Tnfsf11	LOC101404114
DOK6	Dok6	LOC101393780	NRAS	Nras	LOC101403366	TNFSF12	Tnfsf12	LOC101402026
DOK7	Dok7	LOC101400177	NRBF2	Nrbf2	LOC101395060	TNFSF13	Tnfsf13	LOC106799948
DOLK	Dolk	LOC101403037	NRBP1	Nrbp1	LOC101401699	TNFSF13B	Tnfsf13b	LOC101408458
DOLPP1	Dolpp1	LOC101403740	NRBP2	Nrbp2	LOC101394199	TNFSF14	Tnfsf14	LOC101403538
DONSON	Donson	LOC101396985	NRCAM	Nrcam	LOC101401778	TNFSF15	Tnfsf15	LOC101400148
DOP1A	Dopey1	LOC101407132	NRDC	Nrd1	LOC101405629	TNFSF18	Tnfsf18	LOC101400688
DOP1B	Dopey2	LOC101389206	NRDE2	Nrde2	LOC101396646	TNFSF4	Tnfsf4	LOC101395662
DOT1L	Dot1l	LOC101406357	NREP	Nrep	LOC101403625	TNFSF8	Tnfsf8	LOC101400421
DPAGT1	Dpagt1	LOC101389876	NRF1	Nrf1	LOC101391114	TNIK	Tnik	LOC101390730
DPCD	Dpcd	LOC101406290	NRG1	Nrg1	LOC101405502	TNIP1	Tnip1	LOC101397326
DPEP1	Dpep1	LOC101404485	NRG2	Nrg2	LOC101387692	TNIP2	Tnip2	LOC101397680
DPEP2	Dpep2	LOC101402709	NRG3	Nrg3	LOC101395685	TNIP3	Tnip3	LOC101388744
DPEP3	Dpep3	LOC101397587	NRG4	Nrg4	LOC101392925	TNK1	Tnk1	LOC101405955
DPF1	Dpf1	LOC101401106	NRGN	Nrgn	LOC101393482	TNK2	Tnk2	LOC101406443
DPF2	Dpf2	LOC101403695	NRIP2	Nrip2	LOC101389304	TNKS	Tnks	LOC101401323
DPF3	Dpf3	LOC101389008	NRIP3	Nrip3	LOC101392206	TNKS1BP1	Tnks1bp1	LOC101387403
DPH1	Dph1	LOC101400453	NRK	Nrk	LOC101399792	TNKS2	Tnks2	LOC101393712
DPH2	Dph2	LOC101388462	NRL	Nrl	LOC101399094	TNMD	Tnmd	LOC101404144
DPH3	Dph3	LOC101399952	NRM	Nrm	LOC101406878	TNN	Tnn	LOC101395318
DPH5	Dph5	LOC101391288	NRN1	Nrn1	LOC101391417	TNNC1	Tnnc1	LOC101402921
DPH6	Dph6	LOC101406764	NRN1L	Nrn1l	LOC101402190	TNNC2	Tnnc2	LOC101406034
DPH7	Dph7	LOC101395052	NRP1	Nrp1	LOC101390731	TNNI1	Tnni1	LOC101401173
DPM1	Dpm1	LOC101391655	NRP2	Nrp2	LOC101398007	TNNI3	Tnni3	LOC101390272
DPM2	Dpm2	LOC101402424	NRROS	Nrros	LOC101387791	TNNI3K	Tnni3k	LOC101397933
DPP10	Dpp10	LOC101390892	NRSN1	Nrsn1	LOC101396812	TNNT1	Tnnt1	LOC101389645
DPP3	Dpp3	LOC101397098	NRTN	Nrtn	LOC101396768	TNNT2	Tnnt2	LOC101401432
DPP4	Dpp4	LOC101392268	NRXN1	Nrxn1	LOC101390260	TNNT3	Tnnt3	LOC101399345
DPP6	Dpp6	LOC101402795	NRXN2	Nrxn2	LOC101395094	TNP1	Tnp1	LOC101405071
DPP7	Dpp7	LOC101392522	NRXN3	Nrxn3	LOC101397659	TNP2	Tnp2	LOC101387832
DPP8	Dpp8	LOC101390802	NSA2	Nsa2	LOC101400036	TNPO1	Tnpo1	LOC101394439
DPP9	Dpp9	LOC101392136	NSD1	Nsd1	LOC101403664	TNPO2	Tnpo2	LOC101402572
DPPA2	Dppa2	LOC106802840	NSD2	Whsc1	LOC101395423	TNPO3	Tnpo3	LOC101392910
DPPA3	Dppa3	LOC101407276	NSD3	Whsc1l1	LOC101392273	TNR	Tnr	LOC101394808

DPPA4	Dppa4	LOC101405352	NSDHL	Nsdhl	LOC101405478	TNRC18	Tnrc18	LOC101392484
DPPA5	Dppa5a	LOC101390715	NSF	Nsf	LOC101402617	TNRC6A	Tnrc6a	LOC101406367
DPT	Dpt	LOC101387198	NSFL1C	Nsfl1c	LOC101394925	TNRC6B	Tnrc6b	LOC101399676
DPY19L1	Dpy19l1	LOC101405453	NSG1	Nsg1	LOC101407076	TNRC6C	Tnrc6c	LOC101387469
DPY19L3	Dpy19l3	LOC101407814	NSG2	Nsg2	LOC101389111	TNS1	Tns1	LOC101405332
DPY19L4	Dpy19l4	LOC101400260	NSL1	Nsl1	LOC101390131	TNS2	Tns2	LOC101406202
DPY30	Dpy30	LOC101390861	NSMAF	Nsmaf	LOC101405532	TNS3	Tns3	LOC101394587
DPYD	Dpyd	LOC101398071	NSMCE1	Nsmce1	LOC101402479	TNS4	Tns4	LOC101394078
DPYS	Dpys	LOC101406550	NSMCE2	Nsmce2	LOC101393979	TNXB	Tnxb	LOC101388474
DPYSL2	Dpysl2	LOC101404729	NSMCE4A	Nsmce4a	LOC101400783	TOB1	Tob1	LOC101395003
DPYSL3	Dpysl3	LOC101395722	NSMF	Nsmf	LOC101393873	TOB2	Tob2	LOC101405098
DPYSL4	Dpysl4	LOC101396233	NSRP1	Nsrp1	LOC101387563	TOE1	Toe1	LOC101395639
DPYSL5	Dpysl5	LOC101388524	NSUN2	Nsun2	LOC101396324	TOLLIP	Tollip	LOC101407017
DQX1	Dqx1	LOC101400278	NSUN3	Nsun3	LOC101401783	TOM1	Tom1	LOC101392466
DR1	Dr1	LOC101405124	NSUN4	Nsun4	LOC101401373	TOM1L1	Tom1l1	LOC101399142
DRAM1	Dram1	LOC101401352	NSUN6	Nsun6	LOC101406882	TOM1L2	Tom1l2	LOC101391000
DRAM2	Dram2	LOC101393504	NSUN7	Nsun7	LOC101397712	TOMM20	Tomm20	LOC101391418
DRAP1	Drap1	LOC101389736	NT5C	Nt5c	LOC101395252	TOMM20L	Tomm20l	LOC101391728
DRAXIN	Draxin	LOC101393538	NT5C1A	Nt5c1a	LOC101404282	TOMM22	Tomm22	LOC101394585
DRC1	Drc1	LOC101390862	NT5C1B	Nt5c1b	LOC101401621	TOMM34	Tomm34	LOC101389981
DRC7	Drc7	LOC101394735	NT5C2	Nt5c2	LOC101395666	TOMM40	Tomm40	LOC101393170
DRD1	Drd1	LOC101388574	NT5C3B	Nt5c3b	LOC101404216	TOMM40L	Tomm40l	LOC101398248
DRD2	Drd2	LOC101390055	NT5DC1	Nt5dc1	LOC101404159	TOMM5	Tomm5	LOC101407398
DRD3	Drd3	LOC101388007	NT5DC2	Nt5dc2	LOC101403880	TOMM6	Tomm6	LOC101392935
DRD4	Drd4	LOC101409111	NT5DC3	Nt5dc3	LOC101388702	TOMM7	Tomm7	LOC101402052
DRD5	Drd5	LOC101404826	NT5E	Nt5e	LOC101388995	TONSL	Tonsl	LOC101388552
DRG1	Drg1	LOC101389800	NT5M	Nt5m	LOC101403070	TOP1	Top1	LOC101398520
DRG2	Drg2	LOC101392099	NTAN1	Ntan1	LOC101396773	TOP1MT	Top1mt	LOC101397640
DRGX	Prrxl1	LOC101407261	NTF3	Ntf3	LOC101409014	TOP2A	Top2a	LOC101394573
DROSHA	Drosha	LOC101394887	NTF4	Ntf5	LOC101399859	TOP2B	Top2b	LOC101396675
DRP2	Drp2	LOC101400656	NTHL1	Nthl1	LOC101388697	TOP3A	Top3a	LOC101393386
DSC1	Dsc1	LOC101390206	NTM	Ntm	LOC101408756	TOP3B	Top3b	LOC101402932
DSC2	Dsc2	LOC101389769	NTMT1	Ntmt1	LOC101405396	TOPAZ1	Topaz1	LOC101396852
DSC3	Dsc3	LOC101402579	NTN1	Ntn1	LOC101387994	TOPBP1	Topbp1	LOC101390784
DSCAM	Dscam	LOC101395674	NTN3	Ntn3	LOC101407538	TOPORS	Topors	LOC101395329
DSCAML1	Dscaml1	LOC101402010	NTN4	Ntn4	LOC101389234	TOR1A	Tor1b	LOC101406778
DSCC1	Dscc1	LOC101402797	NTN5	Ntn5	LOC101399691	TOR1AIP1	Tor1aip1	LOC101387125
DSE	Dse	LOC101404684	NTNG1	Ntng1	LOC101387856	TOR1AIP2	Tor1aip2	LOC101387629
DSG1	Dsg1a	LOC101390635	NTNG2	Ntng2	LOC101405851	TOR1B	Tor1a	LOC101406524
DSG2	Dsg2	LOC101403117	NTPCR	Ntpcr	LOC101389894	TOR2A	Tor2a	LOC101388820
DSG3	Dsg3	LOC101390884	NTRK1	Ntrk1	LOC101390171	TOR3A	Tor3a	LOC101390898
DSG4	Dsg4	LOC101402851	NTRK2	Ntrk2	LOC101390282	TOR4A	Tor4a	LOC101393196
DSN1	Dsn1	LOC101407067	NTRK3	Ntrk3	LOC101397850	TOX	Tox	LOC101395260
DSP	Dsp	LOC101389377	NTS	Nts	LOC101400197	TOX2	Tox2	LOC101394647
DSPP	Dspp	LOC101403671	NTSR1	Ntsr1	LOC101397766	TOX3	Tox3	LOC101404206
DST	Dst	LOC101399633	NTSR2	Ntsr2	LOC101394845	TOX4	Tox4	LOC101396510
DSTN	Dstn	LOC101390672	NUAK1	Nuak1	LOC101408762	TP53	Trp53	LOC101399576
DSTYK	Dstyk	LOC101402951	NUAK2	Nuak2	LOC101399638	TP53BP1	Trp53bp1	LOC101407739
DTD1	Dtd1	LOC101408661	NUB1	Nub1	LOC101405681	TP53BP2	Trp53bp2	LOC101404396
DTD2	Dtd2	LOC101404865	NUBP1	Nubp1	LOC101408424	TP53I11	Trp53i11	LOC101392297
DTHD1	Dthd1	LOC101389311	NUBP2	Nubp2	LOC101392715	TP53I13	Trp53i13	LOC101399315
DTL	Dtl	LOC101389094	NUBPL	Nubpl	LOC101404070	TP53INP1	Trp53inp1	LOC101401064
DTNA	Dtna	LOC101394706	NUCB1	Nucb1	LOC101397115	TP53INP2	Trp53inp2	LOC101399214
DTNB	Dtnb	LOC101393243	NUCB2	Nucb2	LOC101406160	TP53RK	Trp53rka	LOC101399653

DTNBP1	Dtnbp1	LOC101402615	NUCKS1	Nucks1	LOC101397751	TP63	Trp63	LOC101398344
DTWD1	Dtwd1	LOC101403110	NUDC	Nudc	LOC101399026	TP73	Trp73	LOC101398093
DTWD2	Dtwd2	LOC101395807	NUDCD1	Nudcd1	LOC101398278	TPBG	Tpbg	LOC101406616
DTX1	Dtx1	LOC101393307	NUDCD2	Nudcd2	LOC101400078	TPCN1	Tpcn1	LOC101391742
DTX2	Dtx2	LOC101406431	NUDCD3	Nudcd3	LOC101389149	TPCN2	Tpcn2	LOC101391881
DTX3	Dtx3	LOC101397579	NUDT1	Nudt1	LOC101396329	TPD52	Tpd52	LOC101407945
DTX4	Dtx4	LOC101401428	NUDT12	Nudt12	LOC101407295	TPD52L1	Tpd52l1	LOC101392927
DTYMK	Dtymk	LOC101391831	NUDT13	Nudt13	LOC101393887	TPD52L2	Tpd52l2	LOC101390662
DUOX1	Duox1	LOC101394448	NUDT14	Nudt14	LOC101404214	TPGS1	Tpgs1	LOC101393941
DUOX2	Duox2	LOC101393513	NUDT15	Nudt15	LOC101404985	TPGS2	Tpgs2	LOC101402251
DUOXA1	Duoxa1	LOC101394028	NUDT16	Nudt16	LOC101394685	TPH1	Tph1	LOC101407905
DUOXA2	Duoxa2	LOC101393771	NUDT16L1	Nudt16l1	LOC101393160	TPH2	Tph2	LOC101388102
DUPD1	Dupd1	LOC101406383	NUDT17	Nudt17	LOC101401012	TPI1	Tpi1	LOC101398051
DUS1L	Dus1l	LOC101402195	NUDT18	Nudt18	LOC101407838	TPK1	Tpk1	LOC101392798
DUS2	Dus2	LOC101397849	NUDT19	Nudt19	LOC106803418	TPM1	Tpm1	LOC101401640
DUS3L	Dus3l	LOC101408694	NUDT2	Nudt2	LOC101389109	TPM2	Tpm2	LOC101387871
DUS4L	Dus4l	LOC101404754	NUDT21	Nudt21	LOC101388418	TPM3	Tpm3	LOC101399672
DUSP1	Dusp1	LOC101392003	NUDT22	Nudt22	LOC101390176	TPM4	Tpm4	LOC101402157
DUSP10	Dusp10	LOC101398557	NUDT3	Nudt3	LOC106799956	TPMT	Tpmt	LOC101400089
DUSP11	Dusp11	LOC101387569	NUDT4	Nudt4	LOC101407105	TPO	Tpo	LOC101399758
DUSP12	Dusp12	LOC101394714	NUDT5	Nudt5	LOC101396706	TPP1	Tpp1	LOC101401701
DUSP13	Dusp13	LOC101405067	NUDT6	Nudt6	LOC101406886	TPP2	Tpp2	LOC101405220
DUSP14	Dusp14	LOC101399226	NUDT7	Nudt7	LOC101402210	TPPP	Tppp	LOC101388786
DUSP15	Dusp15	LOC101390801	NUDT8	Nudt8	LOC101387319	TPPP2	Tppp2	LOC101401111
DUSP16	Dusp16	LOC101393323	NUDT9	Nudt9	LOC101402971	TPPP3	Tppp3	LOC101390245
DUSP18	Dusp18	LOC101394645	NUF2	Nuf2	LOC101398755	TPR	Tpr	LOC101396002
DUSP19	Dusp19	LOC101391572	NUFIP1	Nufip1	LOC101406560	TPRA1	Tpra1	LOC101397220
DUSP2	Dusp2	LOC101401515	NUFIP2	Nufip2	LOC101390335	TPRKB	Tprkb	LOC101387824
DUSP21	Dusp21	LOC101401857	NUGGC	Nuggc	LOC101400454	TPRN	Tprn	LOC101390471
DUSP22	Dusp22	LOC101398147	NUMA1	Numa1	LOC101402031	TPSB2	Tpsb2	LOC106800125
DUSP23	Dusp23	LOC101408706	NUMB	Numb	LOC101398595	TPSG1	Tpsg1	LOC101398628
DUSP26	Dusp26	LOC101408902	NUMBL	Numbl	LOC101396681	TPST1	Tpst1	LOC101392036
DUSP27	Dusp27	LOC101389697	NUP107	Nup107	LOC101390827	TPST2	Tpst2	LOC101388673
DUSP3	Dusp3	LOC101388083	NUP133	Nup133	LOC101394634	TPT1	Tpt1	LOC101407623
DUSP4	Dusp4	LOC101401584	NUP153	Nup153	LOC101400619	TPX2	Tpx2	LOC101391546
DUSP5	Dusp5	LOC101406125	NUP155	Nup155	LOC101406516	TRA2A	Tra2a	LOC101399946
DUSP6	Dusp6	LOC101402641	NUP160	Nup160	LOC101408579	TRA2B	Tra2b	LOC101407487
DUSP7	Dusp7	LOC101399343	NUP188	Nup188	LOC101402773	TRABD	Trabd	LOC101406581
DUSP8	Dusp8	LOC106800093	NUP205	Nup205	LOC101394474	TRABD2B	Trabd2b	LOC101399846
DUSP9	Dusp9	LOC101406955	NUP210L	Nup210l	LOC101398620	TRADD	Tradd	LOC101408131
DUT	Dut	LOC101400313	NUP214	Nup214	LOC101389514	TRAF1	Traf1	LOC101404002
DVL1	Dvl1	LOC101394812	NUP35	Nup35	LOC101391829	TRAF2	Traf2	LOC101408624
DVL2	Dvl2	LOC101388854	NUP37	Nup37	LOC101401871	TRAF3	Traf3	LOC101394075
DVL3	Dvl3	LOC101392155	NUP42	Nupl2	LOC101400915	TRAF3IP1	Traf3ip1	LOC101406537
DXO	Dxo	LOC101389694	NUP43	Nup43	LOC101387592	TRAF3IP2	Traf3ip2	LOC101400054
DYDC1	Dydc1	LOC101396905	NUP50	Nup50	LOC101399843	TRAF3IP3	Traf3ip3	LOC101404441
DYDC2	Dydc2	LOC101408047	NUP54	Nup54	LOC101406648	TRAF4	Traf4	LOC101393648
DYM	Dym	LOC101390886	NUP58	Nupl1	LOC101405884	TRAF5	Traf5	LOC101408465
DYNC1H1	Dync1h1	LOC101391752	NUP62CL	Nup62cl	LOC101403182	TRAF6	Traf6	LOC101387924
DYNC1I1	Dync1i1	LOC101405873	NUP85	Nup85	LOC101392547	TRAF7	Traf7	LOC101387578
DYNC1I2	Dync1i2	LOC101396893	NUP88	Nup88	LOC101401681	TRAFD1	Trafd1	LOC101394727
DYNC1LI1	Dync1li1	LOC101391879	NUP93	Nup93	LOC101390497	TRAIP	Traip	LOC101407379
DYNC1LI2	Dync1li2	LOC101403586	NUP98	Nup98	LOC101408832	TRAK1	Trak1	LOC101390191
DYNC2H1	Dync2h1	LOC101407411	NUPR1	Nupr1	LOC101387677	TRAK2	Trak2	LOC101390138

DYNC2LI1	Dync2li1	LOC101402817	NUS1	Nus1	LOC101387107	TRAM1	Tram1	LOC101396388
DYNLL1	Dynll1	LOC101403231	NUSAP1	Nusap1	LOC101396864	TRAM1L1	Tram1l1	LOC101392429
DYNLL2	Dynll2	LOC101404735	NUTF2	Nutf2	LOC101395602	TRAM2	Tram2	LOC101406442
DYNLRB1	Dynlrb1	LOC101400256	NUTM1	Nutm1	LOC101401112	TRANK1	Trank1	LOC101397541
DYNLRB2	Dynlrb2	LOC101400634	NVL	Nvl	LOC101397015	TRAP1	Trap1	LOC101398380
DYNLT1	Dynlt1f	LOC101405556	NWD1	Nwd1	LOC101398583	TRAPPC1	Trappc1	LOC101397078
DYNLT3	Dynlt3	LOC101389037	NWD2	Nwd2	LOC101389559	TRAPPC10	Trappc10	LOC101404027
DYRK1A	Dyrk1a	LOC101391168	NXF1	Nxf1	LOC101402633	TRAPPC11	Trappc11	LOC101404360
DYRK1B	Dyrk1b	LOC101390453	NXF3	Nxf3	LOC101405053	TRAPPC12	Trappc12	LOC101400894
DYRK2	Dyrk2	LOC101388576	NXN	Nxn	LOC101397774	TRAPPC13	Trappc13	LOC101392765
DYRK3	Dyrk3	LOC101393122	NXNL1	Nxnl1	LOC101401902	TRAPPC2	Trappc2	LOC101390342
DYRK4	Dyrk4	LOC101388442	NXNL2	Nxnl2	LOC101400416	TRAPPC2L	Trappc2l	LOC101408157
DYSF	Dysf	LOC101392709	NXPE2	Nxpe2	LOC101407602	TRAPPC3	Trappc3	LOC101391644
DYTN	Dytn	LOC106801334	NXPE3	Nxpe3	LOC101389161	TRAPPC3L	Trappc3l	LOC101405211
DZANK1	Dzank1	LOC101400625	NXPE4	Nxpe4	LOC101407860	TRAPPC4	Trappc4	LOC101393042
DZIP1	Dzip1	LOC101396262	NXPH1	Nxph1	LOC101388756	TRAPPC5	Trappc5	LOC101408881
DZIP1L	Dzip1l	LOC101393095	NXPH3	Nxph3	LOC101403078	TRAPPC6A	Trappc6a	LOC101395541
DZIP3	Dzip3	LOC101396224	NXPH4	Nxph4	LOC101390488	TRAPPC6B	Trappc6b	LOC101406224
E2F1	E2f1	LOC101395039	NXT2	Nxt2	LOC101394510	TRAPPC8	Trappc8	LOC101391806
E2F2	E2f2	LOC101403218	NYAP1	Nyap1	LOC101394032	TRAPPC9	Trappc9	LOC101387139
E2F3	E2f3	LOC101398282	NYNRIN	Nynrin	LOC101390540	TRAT1	Trat1	LOC101406405
E2F4	E2f4	LOC101388080	OAF	Oaf	LOC101407059	TRDMT1	Trdmt1	LOC101388660
E2F5	E2f5	LOC101398723	OARD1	Oard1	LOC101396173	TRDN	Trdn	LOC101391899
E2F6	E2f6	LOC101388353	OAS2	Oas2	LOC106801728	TREH	Treh	LOC101395843
E2F7	E2f7	LOC101393484	OAS3	Oas3	LOC101398358	TREM2	Trem2	LOC101402947
E2F8	E2f8	LOC101392988	OAT	Oat	LOC101402734	TREML1	Treml1	LOC101395135
E4F1	E4f1	LOC101409183	OAZ1	Oaz1	OAZ1	TREML2	Treml2	LOC101402687
EAF1	Eaf1	LOC101396937	OAZ2	Oaz2	OAZ2	TRERF1	Trerf1	LOC101390475
EAF2	Eaf2	LOC101398885	OAZ3	Oaz3	OAZ3	TREX1	Trex1	LOC106799920
EAPP	Eapp	LOC101402757	OBI1	Rnf219	LOC101391224	TREX2	Trex2	LOC101405392
EARS2	Ears2	LOC101387779	OBP2B	Obp2a	LOC106803952	TRH	Trh	LOC101395296
EBAG9	Ebag9	LOC101398960	OBSCN	Obscn	LOC101405567	TRHDE	Trhde	LOC101388520
EBF1	Ebf1	LOC101406717	OBSL1	Obsl1	LOC101399122	TRHR	Trhr	LOC101398023
EBF2	Ebf2	LOC101407079	OC90	Oc90	LOC101388252	TRIAP1	Triap1	LOC101404197
EBF3	Ebf3	LOC101397279	OCA2	Oca2	LOC101392855	TRIB1	Trib1	LOC101393723
EBF4	Ebf4	LOC101391851	OCIAD1	Ociad1	LOC101408684	TRIB2	Trib2	LOC101389637
EBI3	Ebi3	LOC101394870	OCIAD2	Ociad2	LOC101387329	TRIB3	Trib3	LOC101398875
EBNA1BP2	Ebna1bp2	LOC101403806	OCLN	Ocln	LOC101397123	TRIL	Tril	LOC101389748
EBP	Ebp	LOC101391088	OCM2	Ocm	LOC101387090	TRIM10	Trim10	LOC101400132
EBPL	Ebpl	LOC101393389	OCRL	Ocrl	LOC101392562	TRIM11	Trim11	LOC101401450
ECD	Ecd	LOC101393630	OCSTAMP	Ocstamp	LOC101389530	TRIM14	Trim14	LOC101389442
ECE1	Ece1	LOC101394899	ODAM	Odam	LOC101409103	TRIM15	Trim15	LOC101399869
ECE2	Ece2	LOC101390642	ODC1	Odc1	LOC101407272	TRIM16	Trim16	LOC101402537
ECEL1	Ecel1	LOC101394057	ODF1	Odf1	LOC101390573	TRIM17	Trim17	LOC101401193
ECH1	Ech1	LOC101405566	ODF2	Odf2	LOC101396520	TRIM2	Trim2	LOC101405830
ECHDC1	Echdc1	LOC101396164	ODF2L	Odf2l	LOC101391246	TRIM21	Trim21	LOC101398369
ECHDC2	Echdc2	LOC101387932	ODF3	Odf3	LOC101408778	TRIM23	Trim23	LOC101392510
ECHDC3	Echdc3	LOC101401915	ODF3B	Odf3b	LOC101408674	TRIM24	Trim24	LOC101391414
ECHS1	Echs1	LOC101387246	ODF3L1	Odf3l1	LOC101400581	TRIM25	Trim25	LOC101402200
ECI1	Eci1	LOC101394500	ODF3L2	Odf3l2	LOC101393343	TRIM26	Trim26	LOC101399612
ECI2	Eci2	LOC101392962	ODF4	Odf4	LOC101387226	TRIM27	Trim27	LOC101388295
ECM1	Ecm1	LOC101405093	ODR4	BC003331	LOC101395403	TRIM28	Trim28	LOC101399683
ECM2	Ecm2	LOC101395817	OFD1	Ofd1	LOC101389995	TRIM29	Trim29	LOC101408029
ECRG4	1500015O10I	LOC101391596	OGA	Mgea5	LOC101408032	TRIM3	Trim3	LOC101398812

ECSIT	Ecsit	LOC101397035	OGDH	Ogdh	LOC101390090	TRIM31	Trim31	LOC101398234
ECT2	Ect2	LOC101388150	OGDHL	Ogdhl	LOC101405877	TRIM32	Trim32	LOC101400681
ECT2L	Ect2l	LOC101389861	OGFOD1	Ogfod1	LOC101388679	TRIM33	Trim33	LOC101404152
EDA	Eda	LOC101388974	OGFOD2	Ogfod2	LOC101390493	TRIM35	Trim35	LOC101403417
EDAR	Edar	LOC101394493	OGFOD3	Ogfod3	LOC101406731	TRIM36	Trim36	LOC101399526
EDARADD	Edaradd	LOC101394565	OGFR	Ogfr	LOC101404099	TRIM37	Trim37	LOC101387655
EDC3	Edc3	LOC101393431	OGFRL1	Ogfrl1	LOC101394372	TRIM38	Trim38	LOC101390331
EDC4	Edc4	LOC101395341	OGG1	Ogg1	LOC101392242	TRIM39	Trim39	LOC101389197
EDEM1	Edem1	LOC101403988	OGN	Ogn	LOC101396259	TRIM40	Trim40	LOC101397973
EDEM2	Edem2	LOC101396458	OGT	Ogt	LOC101401704	TRIM41	Trim41	LOC101387616
EDEM3	Edem3	LOC101398256	OIP5	Oip5	LOC101396601	TRIM42	Trim42	LOC101400652
EDF1	Edf1	LOC101408369	OIT3	Oit3	LOC101395324	TRIM44	Trim44	LOC101387159
EDIL3	Edil3	LOC101398828	OLA1	Ola1	LOC101396181	TRIM45	Trim45	LOC101397124
EDN1	Edn1	LOC101406210	OLAH	Olah	LOC101400600	TRIM46	Trim46	LOC101390682
EDN2	Edn2	LOC101402323	OLFM1	Olfm1	LOC101400237	TRIM47	Trim47	LOC101399049
EDN3	Edn3	LOC101401933	OLFM2	Olfm2	LOC101402321	TRIM50	Trim50	LOC101403108
EDNRA	Ednra	LOC101398649	OLFM3	Olfm3	LOC101390623	TRIM54	Trim54	LOC101405282
EDNRB	Ednrb	LOC101390720	OLFM4	Olfm4	LOC101402368	TRIM55	Trim55	LOC101398800
EDRF1	Edrf1	LOC101391106	OLFML1	Olfml1	LOC101392473	TRIM58	Trim58	LOC101398488
EEA1	Eea1	LOC101406839	OLFML2A	Olfml2a	LOC101399887	TRIM6	Trim6	LOC106800778
EED	Eed	LOC101396558	OLFML2B	Olfml2b	LOC101395228	TRIM62	Trim62	LOC101405857
EEF1A1	Eef1a1	LOC101399705	OLFML3	Olfml3	LOC101405301	TRIM63	Trim63	LOC101392159
EEF1A2	Eef1a2	LOC101394394	OLIG3	Olig3	LOC101388133	TRIM65	Trim65	LOC101395772
EEF1AKMT1	Eef1akmt1	LOC101400464	OLR1	Olr1	LOC101403865	TRIM66	Trim66	LOC101389649
EEF1AKMT2	Mettl10	LOC101391873	OMA1	Oma1	LOC101399328	TRIM67	Trim67	LOC101387907
EEF1AKMT3	Mettl21b	LOC101402353	OMD	Omd	LOC101397133	TRIM68	Trim68	LOC101402807
EEF1AKNMT	Mettl13	LOC101402510	OMG	Omg	LOC101390794	TRIM69	Trim69	LOC101392591
EEF1B2	Eef1b2	LOC101399983	OMP	Omp	LOC106802438	TRIM7	Trim7	LOC101388646
EEF1D	Eef1d	LOC101396422	ONECUT1	Onecut1	LOC101388900	TRIM71	Trim71	LOC101392735
EEF1E1	Eef1e1	LOC101388585	OOEP	Ooep	LOC101398654	TRIM72	Trim72	LOC101406930
EEF1G	Eef1g	LOC101397698	OOSP2	Oosp2	LOC101390265	TRIM8	Trim8	LOC101392947
EEF2	Eef2	LOC101396331	OPA1	Opa1	LOC101394895	TRIM9	Trim9	LOC101394162
EEF2K	Eef2k	LOC101397135	OPA3	Opa3	LOC101397962	TRIML1	Triml1	LOC101399297
EEF2KMT	Eef2kmt	LOC101389398	OPALIN	Opalin	LOC101407061	TRIML2	Triml2	LOC101392272
EEFSEC	Eefsec	LOC101403728	OPCML	Opcml	LOC101409186	TRIO	Trio	LOC101403049
EEPD1	Eepd1	LOC101399601	OPHN1	Ophn1	LOC101391779	TRIOBP	Triobp	LOC101388355
EFCAB1	Efcab1	LOC101392279	OPLAH	Oplah	LOC101392414	TRIP10	Trip10	LOC101402839
EFCAB11	Efcab11	LOC101395431	OPN1LW	Opn1mw	LOC101391553	TRIP11	Trip11	LOC101399223
EFCAB12	Efcab12	LOC106803874	OPN1SW	Opn1sw	LOC101395191	TRIP12	Trip12	LOC101388155
EFCAB13	Efcab13	LOC101407263	OPN3	Opn3	LOC101401183	TRIP13	Trip13	LOC101389312
EFCAB14	Efcab14	LOC101403462	OPN4	Opn4	LOC101392546	TRIP4	Trip4	LOC101408873
EFCAB5	Efcab5	LOC101399053	OPN5	Opn5	LOC101388919	TRIP6	Trip6	LOC101398335
EFCAB6	Efcab6	LOC101397445	OPRD1	Oprd1	LOC101389453	TRIQK	Triqk	LOC101396990
EFCAB7	Efcab7	LOC101407072	OPRK1	Oprk1	LOC101388181	TRIR	2310036O22I	LOC101403197
EFCAB8	Gm38434	LOC101395815	OPRL1	Oprl1	LOC101388492	TRIT1	Trit1	LOC101405327
EFCAB9	Efcab9	LOC101394140	OPRM1	Oprm1	LOC101406942	TRMO	Trmo	LOC101398085
EFCC1	Efcc1	LOC101391215	OPTC	Optc	LOC101406706	TRMT1	Trmt1	LOC101408612
EFEMP1	Efemp1	LOC101399143	OPTN	Optn	LOC101395492	TRMT10A	Trmt10a	LOC101394560
EFEMP2	Efemp2	LOC101388012	OR10A3	Olfr518	LOC101387326	TRMT10B	Trmt10b	LOC101406874
EFHB	Efhb	LOC101402653	OR10A4	Olfr17	LOC101403001	TRMT10C	Trmt10c	LOC101388107
EFHC1	Efhc1	LOC101406703	OR10A6	Olfr517	LOC101408936	TRMT11	Trmt11	LOC101394618
EFHC2	Efhc2	LOC101402623	OR10AG1	Olfr1123	LOC106800235	TRMT112	Trmt112	LOC101393158
EFHD1	Efhd1	LOC101404888	OR10C1	Olfr95	LOC101401108	TRMT12	Trmt12	LOC101396544
EFHD2	Efhd2	LOC101408631	OR10D3	Olfr958	LOC101401581	TRMT13	Trmt13	LOC101394023

EFL1	Efl1	LOC101393729	OR10G2	Olfr1510	LOC101395209	TRMT1L	Trmt1l	LOC101397314
EFNA1	Efna1	LOC101389298	OR10G3	Olfr1512	LOC101395733	TRMT2A	Trmt2a	LOC101405836
EFNA2	Efna2	LOC101387427	OR10G6	Olfr981	LOC101403660	TRMT2B	Trmt2b	LOC101401632
EFNA3	Efna3	LOC101389043	OR10G7	Olfr978	LOC101400605	TRMT44	Trmt44	LOC101402451
EFNA4	Efna4	LOC101388602	OR10G9	Olfr980	LOC101400869	TRMT5	Trmt5	LOC101409053
EFNA5	Efna5	LOC101406757	OR10H5	Gm4461	LOC101404877	TRMT6	Trmt6	LOC101400803
EFNB1	Efnb1	LOC101390611	OR10J3	Olfr218	LOC101406863	TRMT61A	Trmt61a	LOC101396911
EFNB2	Efnb2	LOC101407138	OR10J5	Olfr16	LOC101408185	TRMU	Trmu	LOC101403606
EFNB3	Efnb3	LOC101399050	OR10K2	Olfr370	LOC101395649	TRNAU1AP	Trnau1ap	LOC101387284
EFR3A	Efr3a	LOC101391327	OR10P1	Olfr796	LOC101391837	TRNT1	Trnt1	LOC101390380
EFR3B	Efr3b	LOC101394008	OR10Q1	Olfr1494	LOC101398303	TRO	Tro	LOC101399967
EFS	Efs	LOC101402668	OR10S1	Olfr982	LOC101401136	TROAP	Troap	LOC101403227
EFTUD2	Eftud2	LOC101396466	OR10V1	Olfr1420	LOC101406409	TRPA1	Trpa1	LOC101392557
EGF	Egf	LOC101399468	OR10X1	Olfr248	LOC101397474	TRPC1	Trpc1	LOC101395542
EGFL6	Egfl6	LOC101389038	OR10Z1	Olfr419	LOC101397732	TRPC3	Trpc3	LOC101408982
EGFL7	Egfl7	LOC101402161	OR11A1	Olfr96	LOC101404149	TRPC4	Trpc4	LOC101401606
EGFL8	Egfl8	LOC101389198	OR11G2	Olfr744	LOC106800513	TRPC4AP	Trpc4ap	LOC101396899
EGFLAM	Egflam	LOC101407847	OR11H4	Olfr748	LOC101407131	TRPC5	Trpc5	LOC101399340
EGFR	Egfr	LOC101399512	OR11H6	Olfr745	LOC101409037	TRPC6	Trpc6	LOC101390316
EGLN1	Egln1	LOC101398607	OR12D1	Olfr101	LOC101405721	TRPC7	Trpc7	LOC101388712
EGLN2	Egln2	LOC101398471	OR12D2	Olfr103	LOC101405990	TRPM2	Trpm2	LOC101403925
EGLN3	Egln3	LOC101403276	OR12D3	Olfr109	LOC101402407	TRPM3	Trpm3	LOC101406940
EGR1	Egr1	LOC101403537	OR13C5	Olfr272	LOC101399544	TRPM4	Trpm4	LOC101401272
EGR2	Egr2	LOC101395841	OR13F1	Olfr275	LOC101400151	TRPM5	Trpm5	LOC101389068
EGR3	Egr3	LOC101402244	OR13J1	Olfr71	LOC101390885	TRPM6	Trpm6	LOC101387251
EGR4	Egr4	LOC101388509	OR14J1	Olfr125	LOC101406502	TRPM7	Trpm7	LOC101405916
EHBP1	Ehbp1	LOC101391091	OR1A1	Olfr43	LOC101396909	TRPM8	Trpm8	LOC101400245
EHBP1L1	Ehbp1l1	LOC101405185	OR1D2	Olfr412	LOC101395691	TRPS1	Trps1	LOC101406724
EHD1	Ehd1	LOC101398045	OR1E2	Olfr20	LOC101392804	TRPT1	Trpt1	LOC101389547
EHD2	Ehd2	LOC101408511	OR1I1	Olfr1357	LOC101399105	TRPV1	Trpv1	LOC101391252
EHD3	Ehd3	LOC101392574	OR1J1	Olfr3	LOC101395397	TRPV2	Trpv2	LOC101397937
EHD4	Ehd4	LOC101400412	OR1J4	Olfr350	LOC101391719	TRPV3	Trpv3	LOC101391669
EHF	Ehf	LOC101405963	OR1N2	Olfr354	LOC101393532	TRPV4	Trpv4	LOC101397062
EHHADH	Ehhadh	LOC101387708	OR1Q1	Olfr357	LOC101398753	TRPV5	Trpv5	LOC101403841
EHMT1	Ehmt1	LOC101395226	OR1S2	Olfr1496	LOC101398047	TRPV6	Trpv6	LOC101404101
EHMT2	Ehmt2	LOC101391395	OR2A12	Olfr446	LOC101397672	TRRAP	Trrap	LOC101406852
EI24	Ei24	LOC101396663	OR2A14	Olfr237-ps1	LOC101393811	TRUB1	Trub1	LOC101391165
EID2	Eid2	LOC101387262	OR2A25	Olfr447	LOC101394558	TRUB2	Trub2	LOC101395224
EID3	Eid3	LOC101406928	OR2A5	Olfr448	LOC101397927	TSACC	Tsacc	LOC106802796
EIF1	Eif1	LOC101406056	OR2AG2	Olfr705	LOC101402480	TSC1	Tsc1	LOC101393959
EIF1AD	Eif1ad	LOC101390687	OR2AT4	Olfr520	LOC101400977	TSC2	Tsc2	LOC101388098
EIF1AX	Eif1ax	LOC101403599	OR2B11	Olfr222	LOC101399611	TSC22D1	Tsc22d1	LOC101405690
EIF1B	Eif1b	LOC101388023	OR2B6	Olfr11	LOC101394696	TSC22D2	Tsc22d2	LOC101387585
EIF2A	Eif2a	LOC101409028	OR2C1	Olfr15	LOC101401430	TSC22D3	Tsc22d3	LOC101390363
EIF2AK1	Eif2ak1	LOC101401276	OR2D2	Gm10081	LOC101403260	TSC22D4	Tsc22d4	LOC101393776
EIF2AK2	Eif2ak2	LOC101393845	OR2H2	Olfr90	LOC101401371	TSEN15	Tsen15	LOC101398933
EIF2AK3	Eif2ak3	LOC101396129	OR2K2	Olfr267	LOC101390303	TSEN2	Tsen2	LOC101388386
EIF2AK4	Eif2ak4	LOC101401016	OR2T1	Olfr31	LOC106800151	TSEN34	Tsen34	LOC101408760
EIF2B1	Eif2b1	LOC101394825	OR2T29	Olfr325	LOC101394426	TSEN54	Tsen54	LOC101395251
EIF2B2	Eif2b2	LOC101408543	OR2T6	Olfr720	LOC101394214	TSFM	Tsfm	LOC101402603
EIF2B3	Eif2b3	LOC101393510	OR2T8	Olfr314	LOC101396012	TSG101	Tsg101	LOC101389743
EIF2B4	Eif2b4	LOC101403868	OR2V2	Olfr1396	LOC101388391	TSGA10	Tsga10	LOC101403690
EIF2B5	Eif2b5	LOC101392938	OR2W1	Olfr263	LOC101387771	TSGA10IP	Tsga10ip	LOC101390001
EIF2D	Eif2d	LOC101393537	OR2Z1	Olfr372	LOC101408920	TSGA13	Tsga13	LOC101404029

EIF2S1	Eif2s1	LOC101406784	OR3A1	Olfr401	LOC101395604	TSHB	Tshb	LOC101401451
EIF2S2	Eif2s2	LOC101404260	OR4A47	Olfr1256	LOC101389822	TSHR	Tshr	LOC101399119
EIF3A	Eif3a	LOC101396186	OR4B1	Olfr1270	LOC101409091	TSHZ1	Tshz1	LOC101389862
EIF3B	Eif3b	LOC101406246	OR4C5	Olfr1260	LOC101388777	TSHZ2	Tshz2	LOC101388580
EIF3D	Eif3d	LOC101396048	OR4D1	Olfr464	LOC106802512	TSKS	Tsks	LOC101408512
EIF3E	Eif3e	LOC101397068	OR4D2	Olfr462	LOC106802506	TSKU	Tsku	LOC101406317
EIF3F	Eif3f	LOC101387839	OR4D5	Olfr984	LOC101401664	TSLP	Tslp	LOC101393596
EIF3G	Eif3g	LOC101407840	OR4D6	Olfr1428	LOC101403785	TSN	Tsn	LOC101397149
EIF3H	Eif3h	LOC101406303	OR4E1	Olfr1508	LOC101394701	TSNAX	Tsnax	LOC101389124
EIF3I	Eif3i	LOC101399288	OR4E2	Olfr1509	LOC101394960	TSNAXIP1	Tsnaxip1	LOC101394562
EIF3K	Eif3k	LOC101403803	OR4F15	Olfr1309	LOC101394113	TSPAN1	Tspan1	LOC101399440
EIF3L	Eif3l	LOC101389397	OR4K1	Olfr728	LOC101390543	TSPAN10	Tspan10	LOC101402802
EIF3M	Eif3m	LOC101403527	OR4K15	Olfr727	LOC101391291	TSPAN12	Tspan12	LOC101407815
EIF4A1	Eif4a1	LOC106799947	OR4K2	Olfr730	LOC101389761	TSPAN13	Tspan13	LOC101394399
EIF4A2	Eif4a2	LOC101402860	OR4K5	Olfr729	LOC101390293	TSPAN14	Tspan14	LOC101396207
EIF4A3	Eif4a3	LOC101400712	OR4L1	Olfr724	LOC101395895	TSPAN15	Tspan15	LOC101406122
EIF4B	Eif4b	LOC101405415	OR4M1	Olfr734	LOC101388723	TSPAN17	Tspan17	LOC101405758
EIF4E	Eif4e	LOC101392799	OR4N2	Olfr732	LOC101387104	TSPAN18	Tspan18	LOC101392024
EIF4E1B	Eif4e1b	LOC101406027	OR4S1	Olfr140	LOC101397272	TSPAN2	Tspan2	LOC101400927
EIF4E2	Eif4e2	LOC101395327	OR4X2	Olfr1269	LOC101387488	TSPAN3	Tspan3	LOC101401803
EIF4E3	Eif4e3	LOC101409108	OR51A7	Olfr576	LOC101398611	TSPAN31	Tspan31	LOC101401142
EIF4EBP1	Eif4ebp1	LOC101390143	OR51B4	Olfr66	LOC101390158	TSPAN32	Tspan32	LOC101389319
EIF4EBP2	Eif4ebp2	LOC101402787	OR51B6	Olfr65	LOC101389384	TSPAN33	Tspan33	LOC101392650
EIF4EBP3	Eif4ebp3	LOC106799939	OR51D1	Olfr557	LOC101402541	TSPAN4	Tspan4	LOC101391122
EIF4ENIF1	Eif4enif1	LOC101388841	OR51E1	Olfr558	LOC106800064	TSPAN5	Tspan5	LOC101392543
EIF4G1	Eif4g1	LOC101388659	OR51F1	Olfr566	LOC101400716	TSPAN6	Tspan6	LOC101403717
EIF4G2	Eif4g2	LOC101399164	OR51G1	Olfr578	LOC101398368	TSPAN7	Tspan7	LOC101408746
EIF4G3	Eif4g3	LOC101393539	OR51I1	Olfr640	LOC101394040	TSPAN8	Tspan8	LOC101396983
EIF4H	Eif4h	LOC101390797	OR51I2	Olfr641	LOC101388857	TSPAN9	Tspan9	LOC101391195
EIF5	Eif5	LOC101395953	OR51Q1	Olfr638	LOC101394534	TSPO	Tspo	LOC101396748
EIF5A	Eif5a	LOC101407701	OR51S1	Olfr571	LOC101399665	TSPO2	Tspo2	LOC101403478
EIF5A2	Eif5a2	LOC101392423	OR51T1	Olfr574	LOC101398876	TSPYL2	Tspyl2	LOC101405586
EIF5B	Eif5b	LOC101406404	OR52A1	Olfr68	LOC101404213	TSPYL4	Tspyl4	LOC101404422
EIF6	Eif6	LOC101395505	OR52D1	Olfr646	LOC101391983	TSPYL5	Tspyl5	LOC101403584
EIPR1	Tssc1	LOC101400630	OR52E4	Olfr677	LOC101392648	TSR1	Tsr1	LOC101399313
ELAC1	Elac1	LOC101396609	OR52E5	Olfr678	LOC101392394	TSR2	Tsr2	LOC101398923
ELAC2	Elac2	LOC101407265	OR52E8	Olfr675	LOC101407108	TSR3	Tsr3	LOC101396230
ELAVL1	Elavl1	LOC101406006	OR52H1	Olfr648	LOC101390965	TSSK2	Tssk2	LOC101397632
ELAVL2	Elavl2	LOC101399559	OR52I2	Olfr556	LOC101403332	TSSK3	Tssk3	LOC101401044
ELAVL3	Elavl3	LOC101396342	OR52K2	Olfr552	LOC101397856	TSSK4	Tssk4	LOC101395037
ELAVL4	Elavl4	LOC101401526	OR52M1	Olfr554	LOC101403594	TSSK6	Tssk6	LOC101401997
ELF1	Elf1	LOC101400801	OR52N2	Olfr669	LOC101408590	TST	Tst	LOC101397797
ELF2	Elf2	LOC101399467	OR52W1	Olfr692	LOC101394427	TSTA3	Tsta3	LOC101398407
ELF3	Elf3	LOC101398895	OR56A3	Olfr679	LOC101387249	TSTD1	Tstd1	LOC101401028
ELF4	Elf4	LOC101389824	OR56A4	Olfr684	LOC101387752	TSTD2	Tstd2	LOC101388143
ELF5	Elf5	LOC101405002	OR56A5	Olfr683	LOC101387496	TSTD3	Tstd3	LOC101404421
ELK1	Elk1	LOC101398796	OR5A1	Olfr76	LOC101403521	TTBK1	Ttbk1	LOC101403821
ELK3	Elk3	LOC101391438	OR5AL1	Olfr1039	LOC106803035	TTBK2	Ttbk2	LOC101405042
ELK4	Elk4	LOC106801034	OR5AN1	Olfr1437	LOC101404840	TTC1	Ttc1	LOC101405412
ELL	Ell	LOC101388469	OR5AS1	Olfr1111	LOC101396354	TTC12	Ttc12	LOC101388832
ELL2	Ell2	LOC101391126	OR5AU1	Olfr221	LOC101399353	TTC13	Ttc13	LOC101409073
ELL3	Ell3	LOC101388632	OR5B12	Olfr1445	LOC101402993	TTC14	Ttc14	LOC101402427
ELMO1	Elmo1	LOC101397365	OR5B17	Olfr1457	LOC101399326	TTC16	Ttc16	LOC101402162
ELMO2	Elmo2	LOC101400354	OR5B2	Olfr1447	LOC106799986	TTC17	Ttc17	LOC101389733

ELMO3	Elmo3	LOC101388336	OR5B21	Olfr1444	LOC101403254	TTC19	Ttc19	LOC101398873
ELMOD1	Elmod1	LOC101403491	OR5B3	Olfr1446	LOC101397867	TTC21A	Ttc21a	LOC101407378
ELMOD2	Elmod2	LOC101396180	OR5C1	Olfr368	LOC101396093	TTC21B	Ttc21b	LOC101408639
ELMOD3	Elmod3	LOC101387573	OR5D14	Olfr1162	LOC106800174	TTC22	Ttc22	LOC101396835
ELMSAN1	Elmsan1	LOC101401662	OR5D16	Olfr1155	LOC106800211	TTC23	Ttc23	LOC101393561
ELN	Eln	LOC101391040	OR5D18	Olfr74	LOC101400589	TTC23L	Ttc23l	LOC101407479
ELOA	Tceb3	LOC101404707	OR5H14	Olfr196	LOC106800036	TTC24	Ttc24	LOC101407437
ELOC	Tceb1	LOC101401518	OR5H15	Olfr193	LOC101402830	TTC25	Ttc25	LOC101402981
ELOF1	Elof1	LOC101397895	OR5H2	Olfr186	LOC101392213	TTC26	Ttc26	LOC101389888
ELOVL1	Elovl1	LOC101405724	OR5H6	Olfr187	LOC101403096	TTC27	Ttc27	LOC101388782
ELOVL2	Elovl2	LOC101407520	OR5I1	Olfr152	LOC101397741	TTC28	Ttc28	LOC101409064
ELOVL3	Elovl3	LOC101389525	OR5J2	Olfr1052	LOC101408969	TTC29	Ttc29	LOC101398401
ELOVL4	Elovl4	LOC101404682	OR5K1	Olfr173	LOC101404133	TTC3	Ttc3	LOC101399816
ELOVL5	Elovl5	LOC101403477	OR5K3	Olfr175-ps1	LOC101403872	TTC30B	Ttc30b	LOC101403659
ELOVL6	Elovl6	LOC101399200	OR5K4	Olfr181	LOC101392476	TTC32	Ttc32	LOC101400909
ELOVL7	Elovl7	LOC101387961	OR5L2	Olfr1157	LOC106800212	TTC33	Ttc33	LOC101388997
ELP1	Ikbkap	LOC101407678	OR5M9	Olfr1034	LOC101391867	TTC36	Ttc36	LOC106801384
ELP2	Elp2	LOC101400586	OR5R1	Olfr1038-ps	LOC101387618	TTC37	Ttc37	LOC101392658
ELP3	Elp3	LOC101399829	OR5T1	Olfr1102	LOC101400236	TTC38	Ttc38	LOC101402903
ELP4	Elp4	LOC101401529	OR5V1	Olfr110	LOC101406253	TTC39A	Ttc39a	LOC101404125
ELP5	Elp5	LOC101387471	OR5W2	Olfr1135	LOC101398250	TTC39B	Ttc39b	LOC101408844
ELP6	Elp6	LOC101387334	OR6B1	Olfr449	LOC101398191	TTC39C	Ttc39c	LOC101400041
EMB	Emb	LOC101389773	OR6B2	Olfr1415	LOC101407500	TTC4	Ttc4	LOC101396312
EMC1	Emc1	LOC101408285	OR6B3	Olfr1414	LOC101407769	TTC5	Ttc5	LOC101408525
EMC10	Emc10	LOC101391447	OR6C1	Olfr802	LOC101398953	TTC6	Ttc6	LOC101398041
EMC2	Emc2	LOC101397333	OR6C4	Olfr767	LOC101391580	TTC7B	Ttc7b	LOC101404571
EMC3	Emc3	LOC101389435	OR6F1	Olfr308	LOC101395499	TTC8	Ttc8	LOC101394074
EMC4	Emc4	LOC101402759	OR6K2	Olfr420	LOC101398481	TTC9	Ttc9	LOC101388743
EMC6	Emc6	LOC101389898	OR6K6	Olfr231	LOC101399270	TTC9B	Ttc9b	LOC101388541
EMC7	Emc7	LOC101403280	OR6N2	Olfr430	LOC101399538	TTC9C	Ttc9c	LOC101401865
EMC8	Emc8	LOC101391345	OR6P1	Olfr414	LOC101405649	TTF1	Ttf1	LOC101392520
EMC9	Emc9	LOC101397379	OR6S1	Olfr750	LOC101405578	TTF2	Ttf2	LOC101403107
EMCN	Emcn	LOC106801869	OR6X1	Olfr986	LOC101402693	TTI1	Tti1	LOC101402606
EMD	Emd	LOC101392506	OR6Y1	Olfr220	LOC101396690	TTI2	Tti2	LOC101408384
EME1	Eme1	LOC101408923	OR7E24	Olfr18	LOC101404037	TTK	Ttk	LOC101404936
EME2	Eme2	LOC101397099	OR7G1	Olfr829	LOC106802452	TTL	Ttl	LOC101390766
EMG1	Emg1	LOC101395531	OR7G2	Olfr854	LOC106800078	TTLL1	Ttll1	LOC101396228
EMID1	Emid1	LOC101407160	OR7G3	Olfr834	LOC106802461	TTLL10	Ttll10	LOC101393293
EMILIN1	Emilin1	LOC101408853	OR8A1	Olfr160	LOC101392200	TTLL11	Ttll11	LOC101389777
EMILIN2	Emilin2	LOC101405125	OR8B12	Olfr874	LOC101391682	TTLL12	Ttll12	LOC101397006
EMILIN3	Emilin3	LOC101396201	OR8B2	Olfr147	LOC101389150	TTLL3	Ttll3	LOC101396346
EML1	Eml1	LOC101389033	OR8B4	Olfr878	LOC101389917	TTLL4	Ttll4	LOC101388833
EML2	Eml2	LOC101398463	OR8B8	Olfr145	LOC101390178	TTLL5	Ttll5	LOC101389786
EML3	Eml3	LOC101398450	OR8D1	Olfr26	LOC101408161	TTLL6	Ttll6	LOC101398203
EML4	Eml4	LOC101405269	OR8D2	Olfr924	LOC101408426	TTLL7	Ttll7	LOC101405950
EML5	Eml5	LOC101403568	OR8D4	Olfr985	LOC101404188	TTLL8	Ttll8	LOC101400549
EML6	Eml6	LOC101387826	OR8G1	Olfr937	LOC101407370	TTLL9	Ttll9	LOC101390545
EMP1	Emp1	LOC101396563	OR8G5	Olfr936	LOC101407643	TTN	Ttn	LOC101407599
EMP2	Emp2	LOC101408931	OR8J1	Olfr1045	LOC101391101	TTPA	Ttpa	LOC106802699
EMP3	Emp3	LOC101390187	OR8K1	Olfr1046	LOC101387113	TTPAL	Ttpal	LOC101392276
EMSY	Emsy	LOC101405088	OR8K3	Olfr1047	LOC106800215	TTR	Ttr	LOC101391139
EMX1	Emx1	LOC101391432	OR8S1	Olfr282	LOC101404558	TTYH1	Ttyh1	LOC101388195
EMX2	Emx2	LOC101394551	OR8U1	Olfr52	LOC101391348	TTYH2	Ttyh2	LOC101407168
EN2	En2	LOC101402020	OR9A2	Olfr459	LOC101403316	TTYH3	Ttyh3	LOC101395806

ENAH	Enah	LOC101397538	OR9A4	Olfr460	LOC101392178	TUB	Tub	LOC101388105
ENAM	Enam	LOC101388020	OR9G1	Olfr1016	LOC101407674	TUBA1A	Tuba1a	LOC101402700
ENC1	Enc1	LOC101392225	OR9I1	Olfr1502	LOC101397273	TUBA1B	Tuba1b	LOC101402965
ENDOD1	Endod1	LOC101394383	OR9K2	Olfr825	LOC101401742	TUBA1C	Tuba1c	LOC101402274
ENDOG	Endog	LOC101403210	OR9Q2	Olfr1497	LOC101397789	TUBA4A	Tuba4a	LOC101394465
ENDOU	Endou	LOC101389883	ORAI1	Orai1	LOC101404971	TUBA8	Tuba8	LOC101403087
ENDOV	Endov	LOC101392700	ORAI2	Orai2	LOC101408355	TUBAL3	Tubal3	LOC101404009
ENG	Eng	LOC101389775	ORAI3	Orai3	LOC101402825	TUBB1	Tubb1	LOC101403145
ENGASE	Engase	LOC101399663	ORC1	Orc1	LOC101408497	TUBB2A	Tubb2a	LOC101394738
ENHO	Enho	LOC101387375	ORC2	Orc2	LOC101388664	TUBB3	Tubb3	LOC101402818
ENKD1	Enkd1	LOC101393560	ORC3	Orc3	LOC101394792	TUBB4A	Tubb4a	LOC101403980
ENKUR	Enkur	LOC101401827	ORC4	Orc4	LOC101403476	TUBB4B	Tubb4b	LOC101392521
ENO1	Eno1	LOC101406282	ORC5	Orc5	LOC101388277	TUBB6	Tubb6	LOC101391458
ENO2	Eno2	LOC101397531	ORC6	Orc6	LOC101403642	TUBD1	Tubd1	LOC101390588
ENO3	Eno3	LOC101398533	ORM1	Orm1	LOC101387441	TUBE1	Tube1	LOC101401725
ENO4	Eno4	LOC101392435	ORM2	Orm2	LOC101387705	TUBG1	Tubg1	LOC101394572
ENOPH1	Enoph1	LOC101393812	ORMDL1	Ormdl1	LOC101397056	TUBG2	Tubg2	LOC101394317
ENOX1	Enox1	LOC101404212	ORMDL2	Ormdl2	LOC101394389	TUBGCP2	Tubgcp2	LOC101394676
ENOX2	Enox2	LOC101387574	ORMDL3	Ormdl3	LOC101400010	TUBGCP3	Tubgcp3	LOC101389189
ENPEP	Enpep	LOC101398940	OS9	Os9	LOC101399388	TUBGCP4	Tubgcp4	LOC101407464
ENPP1	Enpp1	LOC101402324	OSBP	Osbp	LOC101389230	TUBGCP5	Tubgcp5	LOC101390881
ENPP2	Enpp2	LOC101403318	OSBP2	Osbp2	LOC101394392	TUBGCP6	Tubgcp6	LOC101401349
ENPP3	Enpp3	LOC101406263	OSBPL10	Osbpl10	LOC101390871	TUFM	Tufm	LOC101406590
ENPP4	Enpp4	LOC101392779	OSBPL11	Osbpl11	LOC101404543	TUFT1	Tuft1	LOC101404997
ENPP5	Enpp5	LOC101392525	OSBPL1A	Osbpl1a	LOC101399000	TULP1	Tulp1	LOC101408978
ENPP6	Enpp6	LOC101405074	OSBPL2	Osbpl2	LOC101399302	TULP2	Tulp2	LOC101396849
ENPP7	Enpp7	LOC101399918	OSBPL3	Osbpl3	LOC101397016	TULP3	Tulp3	LOC101391871
ENSA	Ensa	LOC101393395	OSBPL5	Osbpl5	LOC101395634	TUSC2	Tusc2	LOC101390449
ENTHD1	Enthd1	LOC101391266	OSBPL6	Osbpl6	LOC101405233	TUSC3	Tusc3	LOC101397327
ENTPD1	Entpd1	LOC101407502	OSBPL7	Osbpl7	LOC101390840	TUT1	Tut1	LOC101397949
ENTPD2	Entpd2	LOC101388560	OSBPL8	Osbpl8	LOC101392392	TUT4	Zcchc11	LOC101408757
ENTPD3	Entpd3	LOC101388286	OSBPL9	Osbpl9	LOC101402640	TUT7	Zcchc6	LOC101392219
ENTPD4	Entpd4	LOC101409079	OSCAR	Oscar	LOC101406156	TVP23A	Tvp23a	LOC101388099
ENTPD5	Entpd5	LOC101403487	OSCP1	Oscp1	LOC101393883	TVP23C	Tvp23b	TVP23B
ENTPD6	Entpd6	LOC101401453	OSER1	Oser1	LOC101394395	TWF1	Twf1	LOC101407948
ENTPD7	Entpd7	LOC101397249	OSGEP	Osgep	LOC101406860	TWF2	Twf2	LOC101400126
ENTPD8	Entpd8	LOC101393441	OSGEPL1	Osgepl1	LOC101396797	TWIST1	Twist1	LOC101405909
ENTR1	Sdccag3	LOC101387881	OSGIN1	Osgin1	LOC101395785	TWIST2	Twist2	LOC101406976
ENY2	Eny2	LOC101398523	OSGIN2	Osgin2	LOC101393896	TWISTNB	Twistnb	LOC101405370
EOGT	Eogt	LOC101405818	OSM	Osm	LOC101390661	TWNK	Peo1	LOC101403919
EOMES	Eomes	LOC101409105	OSMR	Osmr	LOC101408880	TWSG1	Twsg1	LOC101397975
EP300	Ep300	LOC101403170	OSR1	Osr1	LOC101401180	TXK	Txk	LOC101406935
EP400	Ep400	LOC101400520	OSR2	Osr2	LOC101406725	TXLNA	Txlna	LOC101398003
EPAS1	Epas1	LOC101398300	OST4	Ost4	LOC101387413	TXLNB	Txlnb	LOC101395381
EPB41	Epb41	LOC101389699	OSTC	Ostc	LOC101402432	TXLNG	Txlng	LOC101398615
EPB41L1	Epb41l1	LOC101388494	OSTF1	Ostf1	LOC101408070	TXN	Txn1	LOC101389000
EPB41L2	Epb41l2	LOC101400143	OSTM1	Ostm1	LOC101390714	TXN2	Txn2	LOC101395533
EPB41L3	Epb41l3	LOC101400931	OSTN	Ostn	LOC101397313	TXNDC11	Txndc11	LOC101409184
EPB41L4A	Epb41l4a	LOC101403365	OTC	Otc	LOC101387149	TXNDC12	Txndc12	LOC101406927
EPB41L4B	Epb41l4b	LOC101408972	OTOA	Otoa	LOC101389577	TXNDC15	Txndc15	LOC101393683
EPB41L5	Epb41l5	LOC101394892	OTOF	Otof	LOC101390095	TXNDC16	Txndc16	LOC101402515
EPB42	Epb42	LOC101406097	OTOG	Otog	LOC101400740	TXNDC17	Txndc17	LOC101406314
EPC1	Epc1	LOC101391726	OTOGL	Otogl	LOC101395365	TXNDC2	Txndc2	LOC106800385
EPC2	Epc2	LOC101402775	OTOL1	Otol1	LOC101401308	TXNDC5	Txndc5	LOC101388848

EPCAM	Epcam	LOC101395007	OTOP1	Otop1	LOC101407881	TXNDC8	Txndc8	LOC101406702
EPDR1	Epdr1	LOC101400474	OTOP2	Otop2	LOC101389619	TXNDC9	Txndc9	LOC101406146
EPG5	Epg5	LOC101404943	OTOP3	Otop3	LOC101389895	TXNIP	Txnip	LOC101407662
EPGN	Epgn	LOC101394943	OTOR	Otor	LOC101391935	TXNL1	Txnl1	LOC101401205
EPHA1	Epha1	LOC101399655	OTOS	Otos	LOC101408549	TXNL4A	Txnl4a	LOC101400226
EPHA2	Epha2	LOC101402264	OTP	Otp	LOC101387340	TXNL4B	Txnl4b	LOC101407166
EPHA3	Epha3	LOC101397367	OTUB1	Otub1	LOC101387927	TXNRD1	Txnrd1	LOC101406157
EPHA4	Epha4	LOC101400513	OTUB2	Otub2	LOC101404987	TXNRD2	Txnrd2	LOC101393770
EPHA5	Epha5	LOC101398639	OTUD3	Otud3	LOC101389870	TXNRD3	Txnrd3	LOC101407846
EPHA6	Epha6	LOC101390443	OTUD4	Otud4	LOC101395294	TYK2	Tyk2	LOC101388466
EPHA7	Epha7	LOC101399621	OTUD5	Otud5	LOC101408146	TYMP	Tymp	LOC101402904
EPHA8	Epha8	LOC101391645	OTUD6A	Otud6a	LOC101388448	TYMS	Tyms	LOC101407212
EPHB1	Ephb1	LOC101387501	OTUD6B	Otud6b	LOC101395938	TYR	Tyr	LOC101400718
EPHB2	Ephb2	LOC101399380	OTUD7A	Otud7a	LOC101398026	TYRO3	Tyro3	LOC101398477
EPHB3	Ephb3	LOC101390129	OTUD7B	Otud7b	LOC101388266	TYROBP	Tyrobp	LOC101395973
EPHB4	Ephb4	LOC101397819	OTULIN	Otulin	LOC101402788	TYRP1	Tyrp1	LOC101390083
EPHB6	Ephb6	LOC101404369	OTX1	Otx1	LOC101390844	TYSND1	Tysnd1	LOC101393041
EPHX1	Ephx1	LOC101397800	OTX2	Otx2	LOC101388741	TYW1B	Tyw1	LOC101393488
EPHX2	Ephx2	LOC101394156	OVCA2	Ovca2	LOC101396470	TYW3	Tyw3	LOC101398780
EPHX3	Ephx3	LOC101394796	OVCH2	Ovch2	LOC101392726	TYW5	Tyw5	LOC101407600
EPHX4	Ephx4	LOC101388986	OVGP1	Ovgp1	LOC101405466	U2AF1	U2af1	LOC101400351
EPM2A	Epm2a	LOC101391534	OVOL1	Ovol1	LOC101409093	U2AF1L4	U2af1l4	LOC101394863
EPM2AIP1	Epm2aip1	LOC101397804	OVOL2	Ovol2	LOC101389383	U2AF2	U2af2	LOC101392306
EPN1	Epn1	LOC101395710	OVOL3	Ovol3	LOC101401092	U2SURP	U2surp	LOC101395026
EPN2	Epn2	LOC101396211	OXA1L	Oxa1l	LOC101390116	UACA	Uaca	LOC101403549
EPN3	Epn3	LOC101391940	OXCT1	Oxct1	LOC101390890	UAP1	Uap1	LOC101397153
EPO	Epo	LOC101397556	OXR1	Oxr1	LOC101395158	UAP1L1	Uap1l1	LOC101392256
EPOR	Epor	LOC101394963	OXSM	Oxsm	LOC101407557	UBA1	Uba1	LOC101393393
EPPIN	Eppin	LOC101392885	OXSR1	Oxsr1	LOC101403267	UBA2	Uba2	LOC101407813
EPS15	Eps15	LOC101404749	OXT	Oxt	LOC101388764	UBA3	Uba3	LOC101406354
EPS15L1	Eps15l1	LOC101400323	P2RX1	P2rx1	LOC101389622	UBA5	Uba5	LOC101392216
EPS8	Eps8	LOC101400726	P2RX2	P2rx2	LOC101407253	UBA52	Uba52	LOC101387617
EPS8L1	Eps8l1	LOC101389235	P2RX3	P2rx3	LOC101387926	UBA6	Uba6	LOC101399948
EPS8L2	Eps8l2	LOC101394018	P2RX4	P2rx4	LOC101402277	UBA7	Uba7	LOC101407117
EPS8L3	Eps8l3	LOC101398831	P2RX5	P2rx5	LOC101392630	UBAC1	Ubac1	LOC101405588
EPSTI1	Epsti1	LOC101404381	P2RX7	P2rx7	LOC101402017	UBAC2	Ubac2	LOC101400233
EPX	Epx	LOC101393738	P2RY1	P2ry1	LOC101406861	UBALD1	Ubald1	LOC101394170
EPYC	Epyc	LOC101404394	P2RY12	P2ry12	LOC101407731	UBAP1	Ubap1	LOC101389794
EQTN	Eqtn	LOC101409158	P2RY13	P2ry13	LOC101407456	UBAP1L	Ubap1l	LOC101389763
ERAL1	Eral1	LOC101392805	P3H1	P3h1	LOC101405212	UBAP2	Ubap2	LOC101390655
ERAP1	Erap1	LOC101390025	P3H2	P3h2	LOC101407855	UBAP2L	Ubap2l	LOC101402460
ERAS	Eras	LOC101388595	P3H3	P3h3	LOC101399679	UBASH3A	Ubash3a	LOC101402702
ERBB2	Erbb2	LOC101402622	P3H4	P3h4	LOC101404909	UBASH3B	Ubash3b	LOC101405069
ERBB3	Erbb3	LOC101398189	P4HA1	P4ha1	LOC101394294	UBD	Ubd	LOC101398730
ERBB4	Erbb4	LOC101396800	P4HA2	P4ha2	LOC101403364	UBE2A	Ube2a	LOC101397727
ERBIN	Erbb2ip	LOC101393870	P4HA3	P4ha3	LOC101403334	UBE2B	Ube2b	LOC101395723
ERC1	Erc1	LOC101396134	P4HB	P4hb	LOC101398285	UBE2C	Ube2c	LOC101406459
ERC2	Erc2	LOC101389752	P4HTM	P4htm	LOC101395196	UBE2D1	Ube2d1	LOC101401580
ERCC1	Ercc1	LOC101396760	PA2G4	Pa2g4	LOC101398428	UBE2D2	Ube2d2a	LOC101397544
ERCC2	Ercc2	LOC101396239	PABPC1	Pabpc1	LOC101409166	UBE2D3	Ube2d3	LOC101397931
ERCC3	Ercc3	LOC101400153	PABPC1L	Pabpc1l	LOC101390239	UBE2E1	Ube2e1	LOC101404670
ERCC4	Ercc4	LOC101398732	PABPC4	Pabpc4	LOC101403393	UBE2E2	Ube2e2	LOC101396409
ERCC5	Ercc5	LOC101400235	PABPC5	Pabpc5	LOC101391862	UBE2E3	Ube2e3	LOC101387799
ERCC6	Ercc6	LOC101406990	PABPN1	Pabpn1	LOC101403986	UBE2F	Ube2f	LOC101405156

ERCC6L	Ercc6l	LOC101400206	PABPN1L	Pabpn1l	LOC101407896	UBE2G1	Ube2g1	LOC101387225
ERCC6L2	Ercc6l2	LOC101394351	PACC1	Tmem206	LOC101389589	UBE2G2	Ube2g2	LOC101406896
ERCC8	Ercc8	LOC101388390	PACRG	Pacrg	LOC101389166	UBE2H	Ube2h	LOC101390694
EREG	Ereg	LOC101404062	PACRGL	Pacrgl	LOC101393987	UBE2I	Ube2i	LOC101397276
ERF	Erf	LOC101404329	PACS1	Pacs1	LOC101392301	UBE2J1	Ube2j1	LOC101396160
ERG	Erg	LOC101392619	PACS2	Pacs2	LOC101405347	UBE2J2	Ube2j2	LOC101389590
ERGIC1	Ergic1	LOC101393890	PACSIN1	Pacsin1	LOC101391816	UBE2K	Ube2k	LOC101395453
ERGIC2	Ergic2	LOC101407018	PACSIN2	Pacsin2	LOC101395965	UBE2L3	Ube2l3	LOC101404526
ERGIC3	Ergic3	LOC101393459	PACSIN3	Pacsin3	LOC101401344	UBE2L6	Ube2l6	LOC101389479
ERH	Erh	LOC101390051	PADI1	Padi1	LOC101405229	UBE2M	Ube2m	LOC101407006
ERI1	Eri1	LOC101400516	PADI2	Padi2	LOC101404801	UBE2N	Ube2n	LOC101407371
ERI2	Eri2	LOC101394274	PADI3	Padi3	LOC101405492	UBE2O	Ube2o	LOC101404727
ERI3	Eri3	LOC101390115	PADI4	Padi4	LOC101405749	UBE2Q1	Ube2q1	LOC101389997
ERICH2	Erich2	LOC101402598	PADI6	Padi6	LOC101406019	UBE2Q2	Ube2q2	LOC101404154
ERICH3	Erich3	LOC101397069	PAF1	Paf1	LOC101387596	UBE2R2	Ube2r2	LOC101391318
ERICH4	Erich4	LOC101394440	PAFAH1B1	Pafah1b1	LOC101397684	UBE2S	Ube2s	LOC101392718
ERICH5	Erich5	LOC101404723	PAFAH1B2	Pafah1b2	LOC101404809	UBE2T	Ube2t	LOC101397612
ERICH6	Erich6	LOC101388979	PAFAH1B3	Pafah1b3	LOC101403616	UBE2U	Ube2u	LOC101407784
ERICH6B	Erich6b	LOC101404471	PAFAH2	Pafah2	LOC101391399	UBE2V1	Ube2v1	LOC101392884
ERLEC1	Erlec1	LOC101389045	PAG1	Pag1	LOC101408633	UBE2V2	Ube2v2	LOC101392692
ERLIN1	Erlin1	LOC101399204	PAGR1	Pagr1a	LOC101395017	UBE2W	Ube2w	LOC101401255
ERLIN2	Erlin2	LOC101387809	PAH	Pah	LOC101403788	UBE2Z	Ube2z	LOC101399496
ERMAP	Ermap	LOC101405732	PAICS	Paics	LOC101394101	UBE3A	Ube3a	LOC101394196
ERMARD	Ermard	LOC101405842	PAIP1	Paip1	LOC101394202	UBE3B	Ube3b	LOC101393975
ERMN	Ermn	LOC101400077	PAIP2	Paip2	LOC101400487	UBE3C	Ube3c	LOC101404820
ERMP1	Ermp1	LOC101393998	PAIP2B	Paip2b	LOC101396222	UBE3D	Ube2cbp	LOC101406865
ERN1	Ern1	LOC101389125	PAK1	Pak1	LOC101407625	UBE4A	Ube4a	LOC101398263
ERN2	Ern2	LOC101408002	PAK1IP1	Pak1ip1	LOC101408914	UBE4B	Ube4b	LOC101387448
ERO1A	Ero1l	LOC101402782	PAK2	Pak2	LOC101388314	UBFD1	Ubfd1	LOC101387521
ERO1B	Ero1lb	LOC101394308	PAK3	Pak3	LOC101396590	UBIAD1	Ubiad1	LOC101392079
ERP27	Erp27	LOC101399059	PAK4	Pak4	LOC101408260	UBL3	Ubl3	LOC101390678
ERP29	Erp29	LOC101395245	PAK6	Pak6	LOC101401287	UBL4A	Ubl4a	LOC101399010
ERP44	Erp44	LOC101393612	PALB2	Palb2	LOC101409127	UBL5	Ubl5	LOC101405577
ERRFI1	Errfi1	LOC101404956	PALD1	Pald1	LOC101402270	UBL7	Ubl7	LOC101391894
ESAM	Esam	LOC101394003	PALLD	Palld	LOC101407791	UBLCP1	Ublcp1	LOC101406201
ESCO1	Esco1	LOC101387694	PALM	Palm	LOC101391033	UBN1	Ubn1	LOC101391102
ESCO2	Esco2	LOC101401240	PALM3	Palm3	LOC101389430	UBN2	Ubn2	LOC101389613
ESD	Esd	LOC101388763	PALMD	Palmd	LOC101396335	UBOX5	Ubox5	LOC101388086
ESF1	Esf1	LOC101394484	PAM	Pam	LOC101409030	UBP1	Ubp1	LOC101394431
ESM1	Esm1	LOC101399011	PAMR1	Pamr1	LOC101409008	UBQLN1	Ubqln1	LOC101387089
ESPL1	Espl1	LOC101409062	PAN2	Pan2	LOC101403403	UBQLN2	Ubqln2	LOC101400146
ESPN	Espn	LOC101402263	PAN3	Pan3	LOC101388954	UBQLN3	Ubqln3	LOC101391470
ESPNL	Espnl	LOC101405864	PANK1	Pank1	LOC101390982	UBQLN4	Ubqln4	LOC101400809
ESR1	Esr1	ESR1	PANK2	Pank2	LOC101403772	UBQLNL	Ubqlnl	LOC101391223
ESR2	Esr2	ESR2	PANK3	Pank3	LOC101398683	UBR1	Ubr1	LOC101405307
ESRP1	Esrp1	LOC101399658	PANK4	Pank4	LOC101396097	UBR2	Ubr2	LOC101390223
ESRP2	Esrp2	LOC101398781	PANX1	Panx1	LOC101397322	UBR3	Ubr3	LOC101400699
ESRRA	Esrra	LOC101403958	PANX2	Panx2	LOC101406335	UBR4	Ubr4	LOC101408020
ESRRB	Esrrb	LOC101391159	PANX3	Panx3	LOC101392464	UBR5	Ubr5	LOC101390149
ESRRG	Esrrg	LOC101393929	PAOX	Paox	LOC101393666	UBR7	Ubr7	LOC101403076
ESS2	Dgcr14	LOC101397381	PAPLN	Papln	LOC101389519	UBTD1	Ubtd1	LOC101391650
ESYT1	Esyt1	LOC101398952	PAPOLA	Papola	LOC101409000	UBTD2	Ubtd2	LOC101392790
ESYT2	Esyt2	LOC101400257	PAPOLB	Papolb	LOC106803621	UBTF	Ubtf	LOC101392368
ESYT3	Esyt3	LOC101404063	PAPOLG	Papolg	LOC101397188	UBXN1	Ubxn1	LOC101400636

ETAA1	Etaa1	LOC101407359	PAPPA	Pappa	LOC101388734	UBXN10	Ubxn10	LOC101390649
ETF1	Etf1	LOC101403104	PAPPA2	Pappa2	LOC101394049	UBXN11	Ubxn11	LOC101395234
ETFA	Etfa	LOC101403199	PAPSS1	Papss1	LOC101404628	UBXN2A	Ubxn2a	LOC101397957
ETFB	Etfb	LOC101401558	PAPSS2	Papss2	LOC101407553	UBXN2B	Ubxn2b	LOC101405268
ETFDH	Etfdh	LOC101393508	PAQR3	Paqr3	LOC101389463	UBXN4	Ubxn4	LOC101408280
ETFRF1	Lyrm5	LOC101390679	PAQR4	Paqr4	LOC101404841	UBXN6	Ubxn6	LOC101392916
ETHE1	Ethe1	LOC101387330	PAQR5	Paqr5	LOC101401802	UBXN7	Ubxn7	LOC101408111
ETNK1	Etnk1	LOC101388599	PAQR6	Paqr6	LOC101403340	UBXN8	Ubxn8	LOC101404292
ETNK2	Etnk2	LOC101403745	PAQR9	Paqr9	LOC101390284	UCHL1	Uchl1	LOC101398560
ETNPPL	Etnppl	LOC101402006	PARD3	Pard3	LOC101390132	UCHL3	Uchl3	LOC101387877
ETS1	Ets1	LOC101402733	PARD3B	Pard3b	LOC101397407	UCHL5	Uchl5	LOC101387934
ETS2	Ets2	LOC101393553	PARD6A	Pard6a	LOC101393313	UCK1	Uck1	LOC101390971
ETV1	Etv1	LOC101390992	PARD6G	Pard6g	LOC101401023	UCK2	Uck2	LOC101401732
ETV2	Etv2	LOC101393846	PARG	Parg	LOC101404901	UCKL1	Uckl1	LOC101390146
ETV3	Etv3	LOC101391757	PARK7	Park7	LOC101404706	UCMA	Ucma	LOC101401395
ETV3L	Etv3l	LOC101391512	PARL	Parl	LOC101397654	UCN	Ucn	LOC101405014
ETV4	Etv4	LOC101409074	PARM1	Parm1	LOC101404331	UCN3	Ucn3	LOC101404626
ETV5	Etv5	LOC101407052	PARN	Parn	LOC101397890	UCP1	Ucp1	LOC101395919
ETV6	Etv6	LOC101391854	PARP1	Parp1	LOC101387174	UCP2	Ucp2	LOC101396298
EVA1A	Eva1a	LOC101404580	PARP10	Parp10	LOC101405138	UCP3	Ucp3	LOC101396557
EVA1B	Eva1b	LOC101392942	PARP11	Parp11	LOC101390180	UEVLD	Uevld	LOC101390012
EVA1C	Eva1c	LOC101404096	PARP12	Parp12	LOC101393978	UFC1	Ufc1	LOC101395483
EVC	Evc	LOC101404565	PARP14	Parp14	LOC101402862	UFD1	Ufd1l	LOC101396155
EVC2	Evc2	LOC101405774	PARP16	Parp16	LOC101390546	UFL1	Ufl1	LOC101400584
EVI2A	Evi2a	LOC101391039	PARP2	Parp2	LOC101407837	UFM1	Ufm1	LOC101387396
EVI2B	Evi2b	LOC101390539	PARP3	Parp3	LOC101396939	UFSP1	Ufsp1	LOC101399178
EVI5	Evi5	LOC101408351	PARP6	Parp6	LOC101407467	UFSP2	Ufsp2	LOC101408034
EVI5L	Evi5l	LOC101407930	PARP8	Parp8	LOC101395740	UGCG	Ugcg	LOC101391718
EVL	Evl	LOC101389473	PARPBP	Parpbp	LOC101402126	UGDH	Ugdh	LOC101394600
EVPL	Evpl	LOC101400622	PARS2	Pars2	LOC101396573	UGGT1	Uggt1	LOC101403126
EVX1	Evx1	LOC101391689	PARVA	Parva	LOC101398313	UGGT2	Uggt2	LOC101396783
EVX2	Evx2	LOC101405407	PARVB	Parvb	LOC101398048	UGP2	Ugp2	LOC101389633
EWSR1	Ewsr1	LOC101406129	PARVG	Parvg	LOC101398453	UGT1A6	Ugt1a6a	LOC101398944
EXD1	Exd1	LOC101396075	PASK	Pask	LOC101388665	UGT1A7	Ugt1a7c	LOC101399726
EXD2	Exd2	LOC101389352	PATE1	Pate1	LOC101396749	UGT2A2	Ugt2a2	LOC101407909
EXO1	Exo1	LOC101392724	PATE2	Pate2	LOC106803132	UGT2B15	Ugt2b1	LOC101404399
EXOC1	Exoc1	LOC101392994	PATE3	Pate3	LOC101398549	UGT3A2	Ugt3a1	LOC101402768
EXOC2	Exoc2	LOC101397618	PATJ	Inadl	LOC101392958	UGT8	Ugt8a	LOC101393207
EXOC3	Exoc3	LOC101387686	PATL1	Patl1	LOC101405896	UHMK1	Uhmk1	LOC101396704
EXOC3L2	Exoc3l2	LOC101407654	PATL2	Patl2	LOC101403634	UHRF1	Uhrf1	LOC101391032
EXOC3L4	Exoc3l4	LOC101395169	PATZ1	Patz1	LOC101390067	UHRF1BP1	Uhrf1bp1	LOC101390641
EXOC4	Exoc4	LOC101401750	PAWR	Pawr	LOC101406417	UHRF1BP1L	Uhrf1bp1l	LOC101397201
EXOC5	Exoc5	LOC101389517	PAX1	Pax1	LOC101400407	UHRF2	Uhrf2	LOC101392383
EXOC6	Exoc6	LOC101396368	PAX2	Pax2	LOC101402090	UIMC1	Uimc1	LOC101404813
EXOC6B	Exoc6b	LOC101391942	PAX3	Pax3	LOC101401139	ULK1	Ulk1	LOC101399995
EXOC7	Exoc7	LOC101402194	PAX4	Pax4	LOC101399949	ULK2	Ulk2	LOC101405428
EXOC8	Exoc8	LOC101388849	PAX5	Pax5	LOC101408971	ULK3	Ulk3	LOC101395124
EXOG	Exog	LOC101404671	PAX6	Pax6	LOC101402047	ULK4	Ulk4	LOC101389932
EXOSC1	Exosc1	LOC101390059	PAX7	Pax7	LOC101407054	UMAD1	Umad1	LOC106801960
EXOSC10	Exosc10	LOC101391155	PAX8	Pax8	LOC101408840	UMODL1	Umodl1	LOC101397414
EXOSC2	Exosc2	LOC101388472	PAX9	Pax9	LOC101409088	UMPS	Umps	LOC101402948
EXOSC3	Exosc3	LOC101406622	PAXBP1	Paxbp1	LOC101405418	UNC119	Unc119	LOC101397429
EXOSC4	Exosc4	LOC101392148	PAXIP1	Paxip1	LOC101406647	UNC119B	Unc119b	LOC101401484
EXOSC5	Exosc5	LOC101402663	PAXX	BC029214	LOC101387531	UNC13A	Unc13a	LOC101394039

EXOSC7	Exosc7	LOC101399434	PBDC1	Pbdc1	LOC101398650	UNC13B	Unc13b	LOC101408452
EXOSC8	Exosc8	LOC101400539	PBK	Pbk	LOC101400975	UNC13C	Unc13c	LOC101408359
EXOSC9	Exosc9	LOC101387974	PBLD	Pbld2	LOC101390651	UNC13D	Unc13d	LOC101398364
EXPH5	Exph5	LOC101400870	PBRM1	Pbrm1	LOC101404404	UNC45A	Unc45a	LOC101408133
EXT1	Ext1	LOC101404977	PBX1	Pbx1	LOC101399021	UNC45B	Unc45b	LOC101401194
EXT2	Ext2	LOC101391096	PBX2	Pbx2	LOC101408281	UNC50	Unc50	LOC106802744
EXTL1	Extl1	LOC101391643	PBX3	Pbx3	LOC101404540	UNC5A	Unc5a	LOC101405501
EXTL2	Extl2	LOC101391793	PBX4	Pbx4	LOC101397740	UNC5B	Unc5b	LOC101400693
EXTL3	Extl3	LOC101398033	PBXIP1	Pbxip1	LOC101406916	UNC5C	Unc5c	LOC101391658
EYA1	Eya1	LOC101397351	PC	Pcx	LOC101402041	UNC5CL	Unc5cl	LOC101396702
EYA2	Eya2	LOC101398956	PCBD1	Pcbd1	LOC101391997	UNC5D	Unc5d	LOC101409159
EYA3	Eya3	LOC101406191	PCBD2	Pcbd2	LOC101393421	UNC79	Unc79	LOC101403597
EYA4	Eya4	LOC101404423	PCBP1	Pcbp1	LOC101400540	UNC80	Unc80	LOC101397575
EZH1	Ezh1	LOC101392705	PCBP2	Pcbp2	LOC101390064	UNC93B1	Unc93b1	LOC101388272
EZH2	Ezh2	LOC101393720	PCBP3	Pcbp3	LOC101387211	UNG	Ung	LOC101395153
EZR	Ezr	LOC101406244	PCBP4	Pcbp4	LOC101397211	UNK	Unk	LOC101398118
F10	F10	LOC101402585	PCCA	Pcca	LOC101402505	UPB1	Upb1	LOC101389085
F11	F11	LOC101391070	PCCB	Pccb	LOC101408687	UPF1	Upf1	LOC101407318
F11R	F11r	LOC101391222	PCDH1	Pcdh1	LOC101406087	UPF2	Upf2	LOC101397241
F12	F12	LOC101401404	PCDH10	Pcdh10	LOC101401663	UPF3A	Upf3a	LOC101404696
F13A1	F13a1	LOC101391176	PCDH12	Pcdh12	LOC101405561	UPF3B	Upf3b	LOC101399702
F13B	F13b	LOC101407981	PCDH15	Pcdh15	LOC101403265	UPK1A	Upk1a	LOC101392909
F2	F2	LOC101399233	PCDH17	Pcdh17	LOC101399139	UPK1B	Upk1b	LOC101407092
F2R	F2r	LOC101389320	PCDH18	Pcdh18	LOC101401134	UPK2	Upk2	LOC101394056
F2RL1	F2rl1	LOC101389070	PCDH19	Pcdh19	LOC101404406	UPK3A	Upk3a	LOC101400108
F2RL2	F2rl2	LOC101389844	PCDH20	Pcdh20	LOC101397265	UPK3B	Upk3b	LOC101406691
F2RL3	F2rl3	LOC101398084	PCDH7	Pcdh7	LOC101388788	UPP1	Upp1	LOC101402905
F3	F3	LOC101401797	PCDH8	Pcdh8	LOC101399664	UPP2	Upp2	LOC101404967
F5	F5	LOC101397752	PCDH9	Pcdh9	LOC101405925	UPRT	Uprt	LOC101397977
F7	F7	LOC101389440	PCDHA3	Pcdha3	LOC101408780	UQCC1	Uqcc1	LOC101394648
F8	F8	LOC101402942	PCDHA4	Pcdha4	LOC101389244	UQCC2	Uqcc2	LOC101394206
F9	F9	LOC101405202	PCDHA5	Pcdha5	LOC101408521	UQCC3	Uqcc3	LOC101400378
FA2H	Fa2h	LOC101408323	PCDHAC1	Pcdhac1	LOC106803529	UQCR10	Uqcr10	LOC101401325
FAAH	Faah	LOC101401985	PCDHAC2	Pcdhac2	LOC101407995	UQCR11	Uqcr11	LOC101387426
FAAP100	Faap100	LOC101395771	PCDHB1	Pcdhb1	LOC101388980	UQCRC1	Uqcrc1	LOC101392653
FAAP24	Faap24	LOC101406345	PCDHB6	Pcdhb13	LOC101387763	UQCRC2	Uqcrc2	LOC101396870
FABP1	Fabp1	LOC101395355	PCDHGA1	Pcdhga1	LOC106803515	UQCRH	Uqcrh	LOC101401110
FABP12	Fabp12	LOC101387800	PCDHGA2	Pcdhga2	LOC101406687	UQCRQ	Uqcrq	LOC101398992
FABP2	Fabp2	LOC101389970	PCDHGA3	Pcdhga3	LOC106803510	URAD	Urad	LOC101388180
FABP3	Fabp3	LOC101393622	PCDHGA4	Pcdhga4	LOC106803182	URB1	Urb1	LOC101394911
FABP4	Fabp4	LOC101387293	PCDHGA5	Pcdhga5	LOC106803179	URB2	Urb2	LOC101390230
FABP5	Fabp5	LOC101408892	PCDHGB4	Pcdhgb4	LOC101409110	URGCP	Urgcp	LOC101408584
FABP6	Fabp6	LOC101404555	PCDHGB5	Pcdhgb5	LOC101408862	URI1	Uri1	LOC101387176
FABP7	Fabp7	LOC101391138	PCDHGC4	Pcdhgc4	LOC101404335	URM1	Urm1	LOC101395999
FABP9	Fabp9	LOC101387545	PCED1A	Pced1a	LOC101390157	UROC1	Uroc1	LOC101404946
FADD	Fadd	LOC101391123	PCF11	Pcf11	LOC101390159	UROD	Urod	LOC101393768
FADS1	Fads1	LOC101394671	PCGF1	Pcgf1	LOC101400544	UROS	Uros	LOC101400470
FADS2	Fads2	LOC101395093	PCGF2	Pcgf2	LOC101387231	USB1	Usb1	LOC101396547
FADS3	Fads3	LOC101395096	PCGF3	Pcgf3	LOC101390833	USE1	Use1	LOC101397395
FADS6	Fads6	LOC101388948	PCGF5	Pcgf5	LOC101392534	USF1	Usf1	LOC101391469
FAF1	Faf1	LOC101402906	PCGF6	Pcgf6	LOC101396185	USF2	Usf2	LOC101391527
FAF2	Faf2	LOC101407607	PCID2	Pcid2	LOC101389955	USF3	Usf3	LOC101390259
FAH	Fah	LOC101390382	PCIF1	Pcif1	LOC101402706	USH1C	Ush1c	LOC101406931
FAHD1	Fahd1	LOC101391768	PCK1	Pck1	LOC101405763	USH1G	Ush1g	LOC101389217

FAIM2	Faim2	LOC101406639	PCK2	Pck2	LOC101398834	USH2A	Ush2a	LOC101393671
FAM102A	Fam102a	LOC101391720	PCLAF	2810417H13I	LOC101408611	USHBP1	Ushbp1	LOC101403472
FAM102B	Fam102b	LOC101409033	PCLO	Pclo	LOC101395074	USO1	Uso1	LOC101404370
FAM104A	Fam104a	LOC101406464	PCM1	Pcm1	LOC101392791	USP1	Usp1	LOC101404373
FAM104B	Tmem29	LOC101402578	PCMT1	Pcmt1	LOC101409109	USP10	Usp10	LOC101392025
FAM107A	Fam107a	LOC101398396	PCMTD1	Pcmtd1	LOC101408839	USP11	Usp11	LOC101397089
FAM107B	Fam107b	LOC101393204	PCMTD2	Pcmtd2	LOC101387814	USP12	Usp12	LOC101406657
FAM114A1	Fam114a1	LOC101391780	PCNA	Pcna	LOC101402540	USP13	Usp13	LOC101402861
FAM114A2	Fam114a2	LOC101392878	PCNP	Pcnp	LOC101388364	USP14	Usp14	LOC101408523
FAM117A	Fam117a	LOC101404038	PCNT	Pcnt	LOC101406644	USP15	Usp15	LOC101404636
FAM117B	Fam117b	LOC101391573	PCNX1	Pcnx	LOC101394900	USP16	Usp16	LOC101396453
FAM118A	Fam118a	LOC101400548	PCNX2	Pcnx2	LOC101390152	USP18	Usp18	LOC101403348
FAM118B	Fam118b	LOC101400820	PCNX3	Pcnx3	LOC101407097	USP19	Usp19	LOC101399084
FAM120A	Fam120a	LOC101398334	PCNX4	Pcnx4	LOC101394051	USP2	Usp2	LOC101409056
FAM120B	Fam120b	LOC101406105	PCOLCE	Pcolce	LOC101395390	USP20	Usp20	LOC101407482
FAM120C	Fam120c	LOC101397642	PCOLCE2	Pcolce2	LOC101395284	USP21	Usp21	LOC101395741
FAM122A	Fam122a	LOC101391785	PCP2	Pcp2	LOC101387437	USP22	Usp22	LOC101388683
FAM122B	Fam122b	LOC101402632	PCP4	Pcp4	LOC101395413	USP24	Usp24	LOC101397611
FAM124A	Fam124a	LOC101395605	PCP4L1	Pcp4l1	LOC106800219	USP25	Usp25	LOC101395632
FAM124B	Fam124b	LOC101405236	PCSK1	Pcsk1	LOC101390533	USP27X	Usp27x	LOC101396385
FAM126A	Fam126a	LOC101401784	PCSK1N	Pcsk1n	LOC101388347	USP28	Usp28	LOC101388747
FAM126B	Fam126b	LOC101388926	PCSK2	Pcsk2	LOC101391503	USP3	Usp3	LOC101405131
FAM131A	Fam131a	LOC101394207	PCSK4	Pcsk4	LOC101408779	USP30	Usp30	LOC101394646
FAM131B	Fam131b	LOC101400172	PCSK5	Pcsk5	LOC101407812	USP31	Usp31	LOC101393111
FAM131C	Fam131c	LOC101387796	PCSK6	Pcsk6	LOC101398526	USP32	Usp32	LOC101395435
FAM133B	Fam133b	LOC101399568	PCSK7	Pcsk7	LOC101404018	USP33	Usp33	LOC101401936
FAM135A	Fam135a	LOC101395314	PCSK9	Pcsk9	LOC101404750	USP34	Usp34	LOC101395610
FAM135B	Fam135b	LOC101387901	PCTP	Pctp	LOC101400461	USP35	Usp35	LOC101387999
FAM136A	Fam136a	LOC101398542	PCYOX1	Pcyox1	LOC101399061	USP36	Usp36	LOC101389471
FAM13A	Fam13a	LOC101407959	PCYOX1L	Pcyox1l	LOC101404092	USP37	Usp37	LOC101408896
FAM13B	Fam13b	LOC101408519	PCYT1A	Pcyt1a	LOC101407231	USP38	Usp38	LOC101393264
FAM13C	Fam13c	LOC101400342	PCYT1B	Pcyt1b	LOC101387916	USP39	Usp39	LOC101390174
FAM149A	Fam149a	LOC101390063	PCYT2	Pcyt2	LOC101399222	USP4	Usp4	LOC101400921
FAM149B1	Fam149b	LOC101393373	PDAP1	Pdap1	LOC101405817	USP40	Usp40	LOC101405157
FAM151A	Fam151a	LOC101395530	PDC	Pdc	LOC101395139	USP42	Usp42	LOC101401540
FAM151B	Fam151b	LOC101404249	PDCD1	Pdcd1	LOC101393044	USP43	Usp43	LOC101408828
FAM155A	Fam155a	LOC101407676	PDCD10	Pdcd10	LOC101398854	USP44	Usp44	LOC101388966
FAM155B	Tmem28	LOC101389558	PDCD11	Pdcd11	LOC101396445	USP45	Usp45	LOC101404158
FAM160A1	Fam160a1	LOC101403016	PDCD1LG2	Pdcd1lg2	LOC101394846	USP46	Usp46	LOC101388885
FAM160A2	Fam160a2	LOC101394941	PDCD2	Pdcd2	LOC101405140	USP47	Usp47	LOC101399428
FAM160B1	Fam160b1	LOC101391166	PDCD2L	Pdcd2l	LOC101388199	USP48	Usp48	LOC101391401
FAM160B2	Fam160b2	LOC101390294	PDCD4	Pdcd4	LOC101406890	USP49	Usp49	LOC101401654
FAM161A	Fam161a	LOC101393828	PDCD5	Pdcd5	LOC101407550	USP5	Usp5	LOC101398708
FAM161B	Fam161b	LOC101402956	PDCD6	Pdcd6	LOC101409102	USP50	Usp50	LOC101405646
FAM162A	Fam162a	LOC101396654	PDCD6IP	Pdcd6ip	LOC101395112	USP53	Usp53	LOC101390482
FAM162B	Fam162b	LOC101407472	PDCD7	Pdcd7	LOC101388304	USP54	Usp54	LOC101391484
FAM163A	Fam163a	LOC101387887	PDCL	Pdcl	LOC101396621	USP6NL	Usp6nl	LOC101398180
FAM163B	Fam163b	LOC101398752	PDCL2	Pdcl2	LOC101392129	USP7	Usp7	LOC101387830
FAM166A	Fam166a	LOC101393793	PDCL3	Pdcl3	LOC101407712	USP8	Usp8	LOC101405044
FAM166B	Fam166b	LOC101408966	PDE10A	Pde10a	LOC101394180	USP9X	Usp9x	LOC101405883
FAM166C	1700001C02F	LOC101389832	PDE11A	Pde11a	LOC101404186	USPL1	Uspl1	LOC101392020
FAM167A	Fam167a	LOC101394155	PDE12	Pde12	LOC101393000	UST	Ust	LOC101389066
FAM167B	Fam167b	LOC101399806	PDE1A	Pde1a	LOC101390399	UTP11	Utp11l	LOC101399898
FAM168A	Fam168a	LOC101393992	PDE1B	Pde1b	LOC101399477	UTP14A	Utp14a	LOC101390515

FAM168B	Fam168b	LOC101404436	PDE1C	Pde1c	LOC101404759	UTP15	Utp15	LOC101393098
FAM169A	Fam169a	LOC101391536	PDE2A	Pde2a	LOC101390506	UTP18	Utp18	LOC101398125
FAM170A	Fam170a	LOC101394264	PDE3A	Pde3a	LOC101405180	UTP20	Utp20	LOC101399850
FAM170B	Fam170b	LOC101407787	PDE3B	Pde3b	LOC101402741	UTP23	Utp23	LOC101406038
FAM171A1	Fam171a1	LOC101399803	PDE4A	Pde4a	LOC101388993	UTP25	Diexf	LOC101405401
FAM171A2	Fam171a2	LOC101406807	PDE4B	Pde4b	LOC101387903	UTP3	Utp3	LOC101388531
FAM171B	Fam171b	LOC101393040	PDE4C	Pde4c	LOC101390552	UTP4	Cirh1a	LOC101402614
FAM172A	Fam172a	LOC101393940	PDE4D	Pde4d	LOC101408011	UTP6	Utp6	LOC101389571
FAM174A	Fam174a	LOC101388372	PDE4DIP	Pde4dip	LOC101392405	UTRN	Utrn	LOC101391788
FAM174B	Fam174b	LOC101400359	PDE5A	Pde5a	LOC101389520	UTS2	Uts2	LOC101404181
FAM174C	1600002K03F	LOC101387691	PDE6A	Pde6a	LOC101402873	UTS2B	Uts2b	LOC101397047
FAM177A1	Fam177a	LOC101391342	PDE6B	Pde6b	LOC101390332	UTS2R	Uts2r	LOC101406466
FAM180A	Fam180a	LOC101396285	PDE6C	Pde6c	LOC101399558	UVRAG	Uvrag	LOC101404383
FAM181A	Fam181a	LOC101404303	PDE6D	Pde6d	LOC101392434	UVSSA	Uvssa	LOC101393641
FAM183A	Fam183b	LOC101403543	PDE6G	Pde6g	LOC101396293	UXS1	Uxs1	LOC101391857
FAM184A	Fam184a	LOC101388641	PDE6H	Pde6h	LOC101399584	UXT	Uxt	LOC101399498
FAM184B	Fam184b	LOC101391751	PDE7A	Pde7a	LOC101398128	VAC14	Vac14	LOC101389988
FAM186B	Fam186b	LOC101387133	PDE7B	Pde7b	LOC101408360	VAMP1	Vamp1	LOC101406481
FAM187A	Fam187a	LOC101407349	PDE8A	Pde8a	LOC101389617	VAMP2	Vamp2	LOC101387737
FAM187B	Fam187b	LOC101399599	PDE8B	Pde8b	LOC101387855	VAMP3	Vamp3	LOC106801106
FAM189A1	Fam189a1	LOC101398281	PDE9A	Pde9a	LOC101399126	VAMP4	Vamp4	LOC101402950
FAM189A2	Fam189a2	LOC101399520	PDF	Pdf	LOC101403504	VAMP5	Vamp5	LOC101400545
FAM189B	Fam189b	LOC101392976	PDGFA	Pdgfa	LOC101400215	VAMP7	Vamp7	LOC101406518
FAM193A	Fam193a	LOC101397424	PDGFB	Pdgfb	LOC101396571	VAMP8	Vamp8	LOC101389142
FAM193B	Fam193b	LOC101391069	PDGFC	Pdgfc	LOC101390029	VANGL1	Vangl1	LOC101400037
FAM199X	Fam199x	LOC101400322	PDGFD	Pdgfd	LOC101406714	VANGL2	Vangl2	LOC101393864
FAM204A	Fam204a	LOC101395240	PDGFRA	Pdgfra	LOC101391201	VAPA	Vapa	LOC101397032
FAM205C	Fam205c	LOC101406026	PDGFRB	Pdgfrb	LOC101401220	VAPB	Vapb	LOC101407255
FAM207A	Fam207a	LOC101408215	PDGFRL	Pdgfrl	LOC101394387	VARS	Vars	LOC101393702
FAM20A	Fam20a	LOC101402887	PDHA1	Pdha1	LOC101401605	VARS2	Vars2	LOC101404176
FAM20B	Fam20b	LOC101391153	PDHA2	Pdha2	LOC101392091	VASH1	Vash1	LOC101391910
FAM20C	Fam20c	LOC101408861	PDHB	Pdhb	LOC101397458	VASH2	Vash2	LOC101391152
FAM210A	Fam210a	LOC101395205	PDHX	Pdhx	LOC101407186	VASN	Vasn	LOC101396662
FAM210B	Fam210b	LOC101408479	PDIA2	Pdia2	LOC101405537	VASP	Vasp	LOC101397713
FAM214A	Fam214a	LOC101407580	PDIA3	Pdia3	LOC101387954	VAT1	Vat1	LOC101388002
FAM214B	Fam214b	LOC101408009	PDIA4	Pdia4	LOC101393460	VAT1L	Vat1l	LOC101401955
FAM216A	Fam216a	LOC101399565	PDIA5	Pdia5	LOC101400427	VAV1	Vav1	LOC101401795
FAM216B	Fam216b	LOC101403418	PDIA6	Pdia6	LOC101408751	VAV2	Vav2	LOC101408723
FAM217A	Fam217a	LOC101393224	PDIK1L	Pdik1l	LOC101392425	VAV3	Vav3	LOC101387429
FAM217B	Fam217b	LOC101406208	PDK1	Pdk1	LOC101394724	VAX1	Vax1	LOC101393546
FAM219A	Fam219a	LOC101387631	PDK2	Pdk2	LOC101406819	VAX2	Vax2	LOC101397269
FAM219B	Fam219b	LOC101396078	PDK3	Pdk3	LOC101387656	VBP1	Vbp1	LOC101404166
FAM220A	Fam220a	LOC101401793	PDK4	Pdk4	LOC101405609	VCAM1	Vcam1	LOC101392051
FAM221A	Fam221a	LOC101399429	PDLIM1	Pdlim1	LOC101405939	VCAN	Vcan	LOC101399527
FAM221B	Fam221b	LOC101390207	PDLIM2	Pdlim2	LOC101389762	VCL	Vcl	LOC101408292
FAM222A	Fam222a	LOC101396803	PDLIM3	Pdlim3	LOC101408812	VCP	Vcp	LOC101393189
FAM222B	Fam222b	LOC101393065	PDLIM4	Pdlim4	LOC101402923	VCPIP1	Vcpip1	LOC101392021
FAM227A	Fam227a	LOC101393919	PDLIM5	Pdlim5	LOC101388942	VCPKMT	Vcpkmt	LOC101395179
FAM228B	Fam228b	LOC101396490	PDLIM7	Pdlim7	LOC101399124	VDAC1	Vdac1	LOC101397023
FAM229A	Fam229a	LOC101401314	PDP1	Pdp1	LOC101398112	VDAC2	Vdac2	LOC101404016
FAM229B	Fam229b	LOC101401994	PDPK1	Pdpk1	LOC101406154	VDAC3	Vdac3	LOC101393262
FAM234A	Fam234a	LOC101405797	PDPN	Pdpn	LOC101407596	VDR	Vdr	LOC101390658
FAM234B	Fam234b	LOC101403246	PDPR	Pdpr	LOC101387529	VEGFA	Vegfa	LOC101397997
FAM237A	Gm39653	LOC106801332	PDRG1	Pdrg1	LOC101390295	VEGFB	Vegfb	LOC101403169

FAM240A	Gm590	LOC106802021	PDS5A	Pds5a	LOC101396057	VEGFC	Vegfc	LOC101400959
FAM241B	2010107G23I	LOC101405329	PDS5B	Pds5b	LOC101395438	VEGFD	Vegfd	LOC101395174
FAM243A	4930563D23I	LOC101408988	PDSS1	Pdss1	LOC101400067	VEPH1	Veph1	LOC101391287
FAM24A	Fam24a	LOC106803236	PDSS2	Pdss2	LOC101389581	VEZF1	Vezf1	LOC101403949
FAM25A	Fam25c	LOC101390249	PDX1	Pdx1	LOC101387657	VEZT	Vezt	LOC101408506
FAM32A	Fam32a	LOC101401208	PDXDC1	Pdxdc1	LOC101396336	VGF	Vgf	LOC101394609
FAM3A	Fam3a	LOC101396426	PDXK	Pdxk	LOC101402014	VGLL2	Vgll2	LOC101408007
FAM3B	Fam3b	LOC101400874	PDXP	Pdxp	LOC101402388	VGLL3	Vgll3	LOC101399947
FAM3C	Fam3c	LOC101406419	PDYN	Pdyn	LOC101394661	VGLL4	Vgll4	LOC101390550
FAM3D	Oit1	LOC101398644	PDZD11	Pdzd11	LOC101387420	VHL	Vhl	LOC101388908
FAM43A	Fam43a	LOC101392937	PDZD2	Pdzd2	LOC101395563	VIL1	Vil1	LOC101408636
FAM43B	Fam43b	LOC101391642	PDZD3	Pdzd3	LOC101388568	VILL	Vill	LOC101400919
FAM47E	Fam47e	LOC101399999	PDZD4	Pdzd4	LOC101388048	VIM	Vim	LOC101388231
FAM49A	Fam49a	LOC101390847	PDZD7	Pdzd7	LOC101404632	VIP	Vip	LOC101405118
FAM49B	Fam49b	LOC101392625	PDZD8	Pdzd8	LOC101394298	VIPAS39	Vipas39	LOC101394983
FAM50A	Fam50a	LOC101395479	PDZD9	Pdzd9	LOC101400222	VIPR1	Vipr1	LOC101391279
FAM50B	Fam50b	LOC101393730	PDZK1IP1	Pdzk1ip1	LOC101398550	VIPR2	Vipr2	LOC101399739
FAM53B	Fam53b	LOC101392124	PDZRN4	Pdzrn4	LOC101403922	VIRMA	1110037F02R	LOC101399395
FAM53C	Fam53c	LOC101404508	PEA15	Pea15a	LOC101390120	VIT	Vit	LOC101408242
FAM71B	Fam71b	LOC101388575	PEAK1	Peak1	LOC101401549	VKORC1	Vkorc1	LOC101404665
FAM71D	Fam71d	LOC101387449	PEAR1	Pear1	LOC101390593	VKORC1L1	Vkorc1l1	LOC101390608
FAM71E1	Fam71e1	LOC101405712	PEBP1	Pebp1	LOC101408388	VLDLR	Vldlr	LOC101394773
FAM71E2	Fam71e2	LOC101391018	PEBP4	Pebp4	LOC101401987	VMA21	Vma21	LOC101394965
FAM71F1	Fam71f1	LOC101396402	PECAM1	Pecam1	LOC101394567	VMAC	Vmac	LOC101408176
FAM71F2	Fam71f2	LOC101396669	PECR	Pecr	LOC101403224	VMO1	Vmo1	LOC101394741
FAM72A	Fam72a	LOC101395229	PEF1	Pef1	LOC101394980	VMP1	Vmp1	LOC101390079
FAM76A	Fam76a	LOC101404182	PEG3	Peg3	LOC101398216	VNN1	Vnn1	LOC101403374
FAM76B	Fam76b	LOC101393709	PELI1	Peli1	LOC101388955	VNN3	Vnn3	LOC101403637
FAM78A	Fam78a	LOC101389776	PELI2	Peli2	LOC101405936	VOPP1	Vopp1	LOC101405538
FAM78B	Fam78b	LOC101402001	PELI3	Peli3	LOC101396309	VPREB3	Vpreb3	LOC101393113
FAM81A	Fam81a	LOC101398331	PELO	Pelo	LOC101396696	VPS11	Vps11	LOC101391486
FAM81B	Fam81b	LOC101392917	PELP1	Pelp1	LOC101393063	VPS13A	Vps13a	LOC101406677
FAM83A	Fam83a	LOC101400173	PEMT	Pemt	LOC101390333	VPS13B	Vps13b	LOC101407164
FAM83B	Fam83b	LOC101402000	PENK	Penk	LOC101393571	VPS13C	Vps13c	LOC101400750
FAM83C	Fam83c	LOC101395248	PEPD	Pepd	LOC101404758	VPS13D	Vps13d	LOC101396627
FAM83D	Fam83d	LOC101399654	PER1	Per1	LOC101394568	VPS16	Vps16	LOC101389724
FAM83E	Fam83e	LOC101392732	PER2	Per2	LOC101408296	VPS18	Vps18	LOC101394876
FAM83F	Fam83f	LOC101399414	PER3	Per3	LOC101402344	VPS25	Vps25	LOC101392190
FAM83G	Fam83g	LOC101395688	PERM1	Perm1	LOC101391995	VPS26A	Vps26a	LOC101407409
FAM83H	Fam83h	LOC101395652	PERP	Perp	LOC101388642	VPS26B	Vps26b	LOC101389151
FAM89A	Fam89a	LOC101398117	PES1	Pes1	LOC101395849	VPS26C	Dscr3	LOC101390491
FAM89B	Fam89b	LOC101406152	PET100	Pet100	LOC101387874	VPS28	Vps28	LOC101388811
FAM8A1	Fam8a1	LOC101401066	PET117	Pet117	LOC101389130	VPS29	Vps29	LOC101398774
FAM91A1	Fam91a1	LOC101397332	PEX1	Pex1	LOC101398605	VPS33A	Vps33a	LOC101408906
FAM92A	Fam92a	LOC101403935	PEX10	Pex10	LOC101398181	VPS33B	Vps33b	LOC101406651
FAM92B	Fam92b	LOC101391760	PEX11A	Pex11a	LOC101402710	VPS35	Vps35	LOC101403897
FAM98A	Fam98a	LOC101387248	PEX11B	Pex11b	LOC101408697	VPS36	Vps36	LOC101398792
FAM98B	Fam98b	LOC101408871	PEX11G	Pex11g	LOC101392764	VPS37A	Vps37a	LOC101395331
FAM98C	Fam98c	LOC101407022	PEX12	Pex12	LOC101404990	VPS37B	Vps37b	LOC101389460
FAN1	Fan1	LOC101387220	PEX13	Pex13	LOC101396387	VPS37C	Vps37c	LOC101388604
FANCA	Fanca	LOC101401954	PEX14	Pex14	LOC101389782	VPS37D	Vps37d	LOC101391541
FANCB	Fancb	LOC101390255	PEX16	Pex16	LOC101394086	VPS39	Vps39	LOC101401199
FANCC	Fancc	LOC101393499	PEX19	Pex19	LOC101392499	VPS41	Vps41	LOC101397462
FANCD2	Fancd2	LOC101395301	PEX26	Pex26	LOC101391610	VPS45	Vps45	LOC101388511

FANCD2OS	Fancd2os	LOC101389182	PEX3	Pex3	LOC101394688	VPS4A	Vps4a	LOC101402885
FANCE	Fance	LOC101408201	PEX5	Pex5	LOC101393332	VPS4B	Vps4b	LOC101388049
FANCG	Fancg	LOC101406869	PEX5L	Pex5l	LOC101403131	VPS50	Vps50	LOC101400879
FANCI	Fanci	LOC101401939	PEX6	Pex6	LOC101387789	VPS51	Vps51	LOC101401257
FANCL	Fancl	LOC101398373	PEX7	Pex7	LOC101387354	VPS52	Vps52	LOC101399372
FANCM	Fancm	LOC101396480	PFAS	Pfas	LOC101392803	VPS53	Vps53	LOC101405878
FANK1	Fank1	LOC101390858	PFDN1	Pfdn1	LOC101396500	VPS54	Vps54	LOC101389223
FAP	Fap	LOC101391167	PFDN2	Pfdn2	LOC101393194	VPS72	Vps72	LOC101400100
FAR1	Far1	LOC101400911	PFDN4	Pfdn4	LOC101387382	VPS8	Vps8	LOC101388228
FAR2	Far2	LOC101407294	PFDN5	Pfdn5	LOC101387209	VPS9D1	Vps9d1	LOC101387925
FARP1	Farp1	LOC101398748	PFDN6	Pfdn6	LOC101398418	VRK1	Vrk1	LOC101388000
FARP2	Farp2	LOC101389277	PFKFB1	Pfkfb1	LOC101400225	VRK2	Vrk2	LOC101398617
FARS2	Fars2	LOC101391663	PFKFB2	Pfkfb2	LOC101390899	VRK3	Vrk3	LOC101389493
FARSA	Farsa	LOC101406366	PFKFB3	Pfkfb3	LOC101401575	VSIG1	Vsig1	LOC101404241
FARSB	Farsb	LOC101402176	PFKFB4	Pfkfb4	LOC101391960	VSIG10	Vsig10	LOC101408644
FAS	Fas	LOC101405980	PFKL	Pfkl	LOC101403229	VSIG10L	Vsig10l	LOC101404268
FASN	Fasn	LOC101402452	PFKM	Pfkm	LOC101391738	VSIG2	Vsig2	LOC101393746
FASTK	Fastk	LOC101408391	PFKP	Pfkp	LOC101389452	VSIG4	Vsig4	LOC101393491
FASTKD1	Fastkd1	LOC101402696	PFN1	Pfn1	LOC101398287	VSIG8	Vsig8	LOC101387103
FASTKD2	Fastkd2	LOC101392612	PFN3	Pfn3	LOC101401668	VSIR	Vsir	LOC101400434
FASTKD3	Fastkd3	LOC101397113	PFN4	Pfn4	LOC101396754	VSNL1	Vsnl1	LOC101403790
FAT1	Fat1	LOC101391737	PGA3	Pga5	LOC101388870	VSTM2A	Vstm2a	LOC101398977
FAT2	Fat2	LOC101395763	PGAM1	Pgam1	LOC101389792	VSTM2B	Vstm2b	LOC101388200
FAT3	Fat3	LOC101400075	PGAM2	Pgam2	LOC101387406	VSTM2L	Vstm2l	LOC101402879
FAT4	Fat4	LOC101405328	PGAM5	Pgam5	LOC101401587	VSTM4	Vstm4	LOC101405424
FAU	Fau	LOC106803008	PGAP1	Pgap1	LOC101403489	VSTM5	Vstm5	LOC101397836
FAXC	Faxc	LOC101403203	PGAP2	Pgap2	LOC101408226	VSX1	Vsx1	LOC101401716
FBF1	Fbf1	LOC101396027	PGAP3	Pgap3	LOC101403077	VSX2	Vsx2	LOC101404709
FBH1	Fbxo18	LOC101402512	PGAP4	Tmem246	LOC101397483	VTA1	Vta1	LOC101394269
FBL	Fbl	LOC101391377	PGAP6	Tmem8	LOC101400733	VTCN1	Vtcn1	LOC101396859
FBLIM1	Fblim1	LOC101400159	PGBD5	Pgbd5	LOC101395665	VTI1A	Vti1a	LOC101387718
FBLN1	Fbln1	LOC101401522	PGC	Pgc	LOC101401909	VTI1B	Vti1b	LOC101408542
FBLN2	Fbln2	LOC101388810	PGD	Pgd	LOC101388740	VTN	Vtn	LOC101398790
FBLN5	Fbln5	LOC101398965	PGF	Pgf	LOC101408116	VWA2	Vwa2	LOC101390653
FBLN7	Fbln7	LOC101391511	PGGT1B	Pggt1b	LOC101399255	VWA3A	Vwa3a	LOC101400753
FBN1	Fbn1	LOC101400577	PGK1	Pgk1	LOC101387600	VWA3B	Vwa3b	LOC101403600
FBN2	Fbn2	LOC101408438	PGK2	Pgk2	LOC101387367	VWA5A	AW551984	LOC101399029
FBP1	Fbp1	LOC101392999	PGLS	Pgls	LOC101394280	VWA5B1	Vwa5b1	LOC101390902
FBP2	Fbp2	LOC101392736	PGLYRP1	Pglyrp1	LOC106803526	VWA5B2	Vwa5b2	LOC101395576
FBRS	Fbrs	LOC101399515	PGLYRP2	Pglyrp2	LOC101396613	VWA7	Vwa7	LOC101389966
FBXL12	Fbxl12	LOC101405310	PGLYRP3	Pglyrp3	LOC101387661	VWA8	Vwa8	LOC101403155
FBXL13	Fbxl13	LOC101399075	PGLYRP4	Pglyrp4	LOC101389911	VWC2	Vwc2	LOC101395362
FBXL15	Fbxl15	LOC101391164	PGM1	Pgm2	LOC101407343	VWC2L	Vwc2l	LOC101399902
FBXL16	Fbxl16	LOC101401260	PGM2	Pgm1	LOC101390361	VWCE	Vwce	LOC101394089
FBXL17	Fbxl17	LOC101406499	PGM2L1	Pgm2l1	LOC101397515	VWF	Vwf	LOC101408498
FBXL18	Fbxl18	LOC101392221	PGM3	Pgm3	LOC101407743	VXN	3110035E14F	LOC101400369
FBXL19	Fbxl19	LOC101402129	PGM5	Pgm5	LOC101392045	WAC	Wac	LOC101395230
FBXL2	Fbxl2	LOC101394014	PGP	Pgp	LOC101394757	WAPL	Wapl	LOC101392963
FBXL20	Fbxl20	LOC101405693	PGPEP1	Pgpep1	LOC101399274	WARS2	Wars2	LOC101394873
FBXL3	Fbxl3	LOC101389187	PGPEP1L	Pgpep1l	LOC101399306	WAS	Was	LOC101389908
FBXL4	Fbxl4	LOC101402939	PGR	Pgr	LOC101390564	WASF1	Wasf1	LOC101396423
FBXL5	Fbxl5	LOC101388946	PGRMC1	Pgrmc1	LOC101395643	WASF2	Wasf2	LOC101402265
FBXL6	Fbxl6	LOC101402248	PGRMC2	Pgrmc2	LOC101403488	WASF3	Wasf3	LOC101407431
FBXL7	Fbxl7	LOC101392243	PGS1	Pgs1	LOC101398872	WASL	Wasl	LOC101401782

FBXL8	Fbxl8	LOC101408397	PHACTR1	Phactr1	LOC101404208	WBP1	Wbp1	LOC101398623
FBXO10	Fbxo10	LOC101407677	PHACTR2	Phactr2	LOC101394017	WBP11	Wbp11	LOC101398295
FBXO11	Fbxo11	LOC101393658	PHACTR3	Phactr3	LOC101401486	WBP1L	Wbp1l	LOC101393889
FBXO15	Fbxo15	LOC101395219	PHACTR4	Phactr4	LOC101408207	WBP2	Wbp2	LOC101398608
FBXO16	Fbxo16	LOC101398535	PHAX	Phax	LOC101388795	WBP2NL	Wbp2nl	LOC101392303
FBXO17	Fbxo17	LOC101407736	PHB	Phb	LOC101402544	WBP4	Wbp4	LOC101401241
FBXO2	Fbxo2	LOC101392607	PHB2	Phb2	LOC101395792	WDFY1	Wdfy1	LOC101404090
FBXO21	Fbxo21	LOC101387984	PHC1	Phc1	LOC101387835	WDFY2	Wdfy2	LOC101396554
FBXO22	Fbxo22	LOC101403889	PHC2	Phc2	LOC101406631	WDFY3	Wdfy3	LOC101398435
FBXO24	Fbxo24	LOC101394791	PHC3	Phc3	LOC101394713	WDFY4	Wdfy4	LOC101408046
FBXO25	Fbxo25	LOC101400395	PHETA2	Fam109b	LOC101387405	WDHD1	Wdhd1	LOC101408376
FBXO27	Fbxo27	LOC101407828	PHEX	Phex	LOC101406656	WDPCP	Wdpcp	LOC101406327
FBXO28	Fbxo28	LOC101404666	PHF1	Phf1	LOC101396522	WDR1	Wdr1	LOC101405084
FBXO3	Fbxo3	LOC101403428	PHF10	Phf10	LOC101403639	WDR11	Wdr11	LOC101393713
FBXO30	Fbxo30	LOC101391282	PHF12	Phf12	LOC101392283	WDR12	Wdr12	LOC101393628
FBXO31	Fbxo31	LOC101390514	PHF13	Phf13	LOC101403483	WDR13	Wdr13	LOC101390166
FBXO32	Fbxo32	LOC101398277	PHF14	Phf14	LOC101389285	WDR17	Wdr17	LOC101399560
FBXO33	Fbxo33	LOC101397524	PHF19	Phf19	LOC101403739	WDR18	Wdr18	LOC101390952
FBXO36	Fbxo36	LOC101388927	PHF2	Phf2	LOC101402497	WDR19	Wdr19	LOC101392835
FBXO38	Fbxo38	LOC101406638	PHF20	Phf20	LOC101389209	WDR20	Wdr20	LOC101392451
FBXO39	Fbxo39	LOC101407424	PHF20L1	Phf20l1	LOC101390242	WDR24	Wdr24	LOC101401523
FBXO4	Fbxo4	LOC101391391	PHF21A	Phf21a	LOC101394580	WDR25	Wdr25	LOC101390422
FBXO40	Fbxo40	LOC101399837	PHF21B	Phf21b	LOC101398215	WDR26	Wdr26	LOC101405974
FBXO41	Fbxo41	LOC101388770	PHF23	Phf23	LOC101388425	WDR27	Wdr27	LOC101405580
FBXO42	Fbxo42	LOC101402780	PHF24	Phf24	LOC101406371	WDR3	Wdr3	LOC101395638
FBXO43	Fbxo43	LOC101407873	PHF3	Phf3	LOC101397150	WDR31	Wdr31	LOC101395830
FBXO44	Fbxo44	LOC101392870	PHF5A	Phf5a	LOC101405358	WDR33	Wdr33	LOC101401907
FBXO45	Fbxo45	LOC101387280	PHF6	Phf6	LOC101403955	WDR34	Wdr34	LOC101399369
FBXO46	Fbxo46	LOC101399858	PHF7	Phf7	LOC101402230	WDR35	Wdr35	LOC101400475
FBXO47	Fbxo47	LOC101407795	PHF8	Phf8	LOC101397390	WDR36	Wdr36	LOC101404769
FBXO48	Fbxo48	LOC101405530	PHGDH	Phgdh	LOC101393855	WDR37	Wdr37	LOC101389698
FBXO5	Fbxo5	LOC101405373	PHIP	Phip	LOC101392503	WDR38	Wdr38	LOC101400150
FBXO6	Fbxo6	LOC101404958	PHKA1	Phka1	LOC101398145	WDR4	Wdr4	LOC101399389
FBXO7	Fbxo7	LOC101390860	PHKA2	Phka2	LOC101401073	WDR41	Wdr41	LOC101387595
FBXO8	Fbxo8	LOC101390760	PHKG1	Phkg1	LOC101388617	WDR43	Wdr43	LOC101395367
FBXO9	Fbxo9	LOC101393617	PHKG2	Phkg2	LOC101400287	WDR44	Wdr44	LOC101394193
FBXW11	Fbxw11	LOC101393299	PHLDB1	Phldb1	LOC101396102	WDR45	Wdr45	LOC101405882
FBXW12	Fbxw21	LOC101407381	PHLDB2	Phldb2	LOC101394495	WDR45B	Wdr45b	LOC101404828
FBXW2	Fbxw2	LOC101402587	PHLDB3	Phldb3	LOC101387083	WDR46	Wdr46	LOC101398668
FBXW4	Fbxw4	LOC101406538	PHLPP1	Phlpp1	LOC101387111	WDR47	Wdr47	LOC101406946
FBXW5	Fbxw5	LOC101408884	PHLPP2	Phlpp2	LOC101387647	WDR48	Wdr48	LOC101406350
FBXW7	Fbxw7	LOC101403542	PHOSPHO1	Phospho1	LOC101401684	WDR49	Wdr49	LOC101399115
FBXW8	Fbxw8	LOC101388842	PHOSPHO2	Phospho2	LOC101401740	WDR5	Wdr5	LOC101399719
FBXW9	Fbxw9	LOC101402319	PHOX2A	Phox2a	LOC101402809	WDR53	Wdr53	LOC101387532
FCER1A	Fcer1a	LOC101402155	PHOX2B	Phox2b	LOC101400119	WDR54	Wdr54	LOC101397002
FCER1G	Fcer1g	LOC101397739	PHPT1	Phpt1	LOC101407853	WDR55	Wdr55	LOC101390788
FCER2	Fcer2a	LOC101408621	PHRF1	Phrf1	LOC101387505	WDR59	Wdr59	LOC101408059
FCF1	Fcf1	LOC101406785	PHTF1	Phtf1	LOC101407386	WDR5B	Wdr5b	LOC101396389
FCGBP	Fcgbp	LOC101387769	PHTF2	Phtf2	LOC101392205	WDR6	Wdr6	LOC101396060
FCGR1A	Fcgr1	LOC101404044	PHYH	Phyh	LOC101394977	WDR60	Wdr60	LOC101399997
FCGR2A	Fcgr3	LOC101393120	PHYHIP	Phyhip	LOC101405726	WDR61	Wdr61	LOC101406693
FCGR2B	Fcgr2b	LOC101393878	PHYHIPL	Phyhipl	LOC101400780	WDR62	Wdr62	LOC101388970
FCGR3B	Fcgr4	LOC101393618	PHYKPL	Phykpl	LOC101390567	WDR63	Wdr63	LOC101400446
FCGRT	Fcgrt	LOC101402054	PI15	Pi15	LOC101403248	WDR64	Wdr64	LOC101392471

FCHO1	Fcho1	LOC101393193	PI16	Pi16	LOC101405855	WDR66	Wdr66	LOC101407252
FCHO2	Fcho2	LOC101400840	PI4K2A	Pi4k2a	LOC101392877	WDR7	Wdr7	LOC101401465
FCHSD1	Fchsd1	LOC101406601	PI4K2B	Pi4k2b	LOC101397336	WDR70	Wdr70	LOC101406957
FCHSD2	Fchsd2	LOC101392374	PI4KA	Pi4ka	LOC101388633	WDR72	Wdr72	LOC101408098
FCMR	Fcmr	LOC101402170	PI4KB	Pi4kb	LOC101401516	WDR73	Wdr73	LOC101402192
FCRL1	Fcrl1	LOC101391261	PIANP	Pianp	LOC101401870	WDR74	Wdr74	LOC101403862
FCRL5	Fcrl5	LOC101390257	PIAS1	Pias1	LOC101399004	WDR75	Wdr75	LOC101395581
FCRL6	Fcrl6	LOC101403201	PIAS2	Pias2	LOC101387613	WDR76	Wdr76	LOC101389853
FCRLA	Fcrla	LOC101408797	PIAS3	Pias3	LOC101387509	WDR77	Wdr77	LOC106800346
FCRLB	Fcrlb	LOC101394458	PIAS4	Pias4	LOC101396066	WDR78	Wdr78	LOC101395250
FDFT1	Fdft1	LOC101393228	PIBF1	Pibf1	LOC101408192	WDR81	Wdr81	LOC101402197
FDPS	Fdps	LOC101396038	PICALM	Picalm	LOC101395345	WDR82	Wdr82	LOC101401007
FDX1	Fdx1	LOC101389355	PICK1	Pick1	LOC101390773	WDR83	Wdr83	LOC101401801
FDX2	Fdx1l	LOC101387778	PID1	Pid1	LOC101387888	WDR83OS	Wdr83os	LOC101401114
FDXACB1	Fdxacb1	LOC101395757	PIDD1	Pidd1	LOC101392655	WDR86	Wdr86	LOC101407958
FDXR	Fdxr	LOC101387992	PIEZO1	Piezo1	LOC101388691	WDR88	Wdr88	LOC101395630
FECH	Fech	LOC101402420	PIEZO2	Piezo2	LOC101396252	WDR90	Wdr90	LOC101399934
FEM1A	Fem1a	LOC101391701	PIF1	Pif1	LOC101388039	WDR91	Wdr91	LOC101397928
FEM1B	Fem1b	LOC101400139	PIFO	Pifo	LOC101391888	WDR92	Wdr92	LOC101406822
FEM1C	Fem1c	LOC101398726	PIGA	Piga	LOC101394749	WDR93	Wdr93	LOC101403152
FER	Fer	LOC101405822	PIGB	Pigb	LOC101391045	WDR97	Gm35339	LOC101403813
FER1L5	Fer1l5	LOC101402546	PIGC	Pigc	LOC101401656	WDSUB1	Wdsub1	LOC101397162
FER1L6	Fer1l6	LOC101397067	PIGF	Pigf	LOC101397785	WDTC1	Wdtc1	LOC101400069
FERMT1	Fermt1	LOC101400266	PIGG	Pigg	LOC101389893	WDYHV1	Wdyhv1	LOC101398868
FERMT2	Fermt2	LOC101404184	PIGH	Pigh	LOC101408021	WEE1	Wee1	LOC101393411
FERMT3	Fermt3	LOC101389144	PIGK	Pigk	LOC101400968	WEE2	Wee2	LOC101406900
FES	Fes	LOC101408913	PIGL	Pigl	LOC101398198	WFDC1	Wfdc1	LOC101392561
FETUB	Fetub	LOC101404439	PIGM	Pigm	LOC101388303	WFDC13	Wfdc13	LOC101391840
FEV	Fev	LOC101399646	PIGN	Pign	LOC101408532	WFDC2	Wfdc2	LOC101393143
FEZ1	Fez1	LOC101396396	PIGO	Pigo	LOC101407314	WFDC5	Wfdc5	LOC101394649
FEZ2	Fez2	LOC101408502	PIGP	Pigp	LOC101390236	WFDC8	Wfdc8	LOC101392623
FEZF1	Fezf1	LOC101405110	PIGQ	Pigq	LOC101400194	WFIKKN1	Wfikkn1	LOC101403345
FEZF2	Fezf2	LOC101399865	PIGR	Pigr	LOC101391820	WFIKKN2	Wfikkn2	LOC101394746
FFAR1	Ffar1	LOC101394338	PIGS	Pigs	LOC101396732	WFS1	Wfs1	LOC101404645
FFAR3	Ffar3	LOC101393587	PIGT	Pigt	LOC101407610	WHAMM	Whamm	LOC101402974
FFAR4	Ffar4	LOC101398354	PIGU	Pigu	LOC101399480	WHRN	Whrn	LOC101399367
FGA	Fga	LOC101392922	PIGV	Pigv	LOC101396969	WIF1	Wif1	LOC101408302
FGB	Fgb	LOC101408262	PIGW	Pigw	LOC101396474	WIPF1	Wipf1	LOC101397835
FGD1	Fgd1	LOC101399185	PIGX	Pigx	LOC101405226	WIPF2	Wipf2	LOC101396121
FGD2	Fgd2	LOC101401571	PIGZ	Pigz	LOC101405746	WIPF3	Wipf3	LOC101407012
FGD3	Fgd3	LOC101401201	PIH1D1	Pih1d1	LOC101403711	WIPI1	Wipi1	LOC101401851
FGD4	Fgd4	LOC101403456	PIH1D2	Pih1d2	LOC101393888	WIPI2	Wipi2	LOC101393001
FGD5	Fgd5	LOC101396257	PIH1D3	Pih1h3b	LOC101388707	WIZ	Wiz	LOC101395565
FGD6	Fgd6	LOC101388275	PIK3AP1	Pik3ap1	LOC101387805	WLS	Wls	LOC101392887
FGF1	Fgf1	LOC101404064	PIK3C2A	Pik3c2a	LOC101405905	WNK1	Wnk1	LOC101394936
FGF10	Fgf10	LOC101395220	PIK3C2B	Pik3c2b	LOC101402511	WNK2	Wnk2	LOC101402246
FGF11	Fgf11	LOC101404468	PIK3C2G	Pik3c2g	LOC101403079	WNK3	Wnk3	LOC101398484
FGF12	Fgf12	LOC101396175	PIK3C3	Pik3c3	LOC101404266	WNK4	Wnk4	LOC101391938
FGF13	Fgf13	LOC101404411	PIK3CA	Pik3ca	LOC101407144	WNT1	Wnt1	LOC101399475
FGF14	Fgf14	LOC101404794	PIK3CB	Pik3cb	LOC101402746	WNT10A	Wnt10a	LOC101390231
FGF16	Fgf16	LOC101398913	PIK3CD	Pik3cd	LOC101408205	WNT10B	Wnt10b	LOC101399210
FGF17	Fgf17	LOC101387100	PIK3CG	Pik3cg	LOC101406342	WNT11	Wnt11	LOC101404652
FGF18	Fgf18	LOC101393547	PIK3IP1	Pik3ip1	LOC101390986	WNT16	Wnt16	LOC101406845
FGF2	Fgf2	LOC101389521	PIK3R1	Pik3r1	LOC101396084	WNT2	Wnt2	LOC101387419

FGF20	Fgf20	LOC101396450	PIK3R2	Pik3r2	LOC101400325	WNT2B	Wnt2b	LOC101389173
FGF21	Fgf21	LOC101395454	PIK3R3	Pik3r3	LOC101398833	WNT3	Wnt3	LOC101402367
FGF22	Fgf22	LOC101392321	PIK3R4	Pik3r4	LOC101396144	WNT3A	Wnt3a	LOC101402490
FGF23	Fgf23	LOC101388518	PIK3R5	Pik3r5	LOC101388424	WNT4	Wnt4	LOC101398095
FGF3	Fgf3	LOC101391620	PIK3R6	Pik3r6	LOC101388853	WNT5A	Wnt5a	LOC101389317
FGF4	Fgf4	LOC101401975	PIKFYVE	Pikfyve	LOC101404088	WNT5B	Wnt5b	LOC101397008
FGF5	Fgf5	LOC101390665	PILRA	Pilra	LOC106803967	WNT6	Wnt6	LOC101389974
FGF6	Fgf6	LOC101389402	PIM1	Pim1	LOC101401306	WNT7A	Wnt7a	LOC101389257
FGF7	Fgf7	LOC101402844	PIM2	Pim2	LOC101408574	WNT7B	Wnt7b	LOC101402215
FGF8	Fgf8	LOC101406790	PIM3	Pim3	LOC101405536	WNT8A	Wnt8a	LOC101408083
FGF9	Fgf9	LOC101402458	PIN1	Pin1	LOC101405995	WNT8B	Wnt8b	LOC101401321
FGFR1	Fgfr1	LOC101393635	PIN4	Pin4	LOC101400480	WRAP53	Wrap53	LOC101399311
FGFR1OP	Fgfr1op	LOC101394686	PINK1	Pink1	LOC101390904	WRAP73	Wrap73	LOC106801115
FGFR1OP2	Fgfr1op2	LOC101392379	PINLYP	Pinlyp	LOC101402652	WRN	Wrn	LOC101405240
FGFR2	Fgfr2	LOC101399728	PINX1	Pinx1	LOC101395429	WRNIP1	Wrnip1	LOC101396731
FGFR3	Fgfr3	LOC101394563	PIP	Pip	LOC101399740	WSB1	Wsb1	LOC101391289
FGFR4	Fgfr4	LOC101404290	PIP4K2A	Pip4k2a	LOC101403747	WSB2	Wsb2	LOC101408905
FGFRL1	Fgfrl1	LOC101391844	PIP4K2B	Pip4k2b	LOC101409083	WSCD1	Wscd1	LOC101404112
FGG	Fgg	LOC101408699	PIP4K2C	Pip4k2c	LOC101396635	WSCD2	Wscd2	LOC101391652
FGGY	Fggy	LOC101400639	PIP5K1A	Pip5k1a	LOC101400370	WT1	Wt1	LOC101403093
FGL1	Fgl1	LOC101393213	PIP5K1B	Pip5k1b	LOC101391370	WTAP	Wtap	LOC101407457
FGL2	Fgl2	LOC101390863	PIP5K1C	Pip5k1c	LOC101400925	WTIP	Wtip	LOC106803417
FGR	Fgr	LOC101403133	PIP5KL1	Pip5kl1	LOC101391477	WWC1	Wwc1	LOC101399207
FH	Fh1	LOC101391952	PIPOX	Pipox	LOC101391423	WWC2	Wwc2	LOC101402600
FHAD1	Fhad1	LOC101408375	PIR	Pir	LOC101395437	WWOX	Wwox	LOC101401167
FHDC1	Fhdc1	LOC101405570	PIRT	Pirt	LOC101407790	WWP1	Wwp1	LOC101393682
FHL1	Fhl1	LOC101400662	PITHD1	Pithd1	LOC101392160	WWP2	Wwp2	LOC101405514
FHL2	Fhl2	LOC101391092	PITPNA	Pitpna	LOC101403771	WWTR1	Wwtr1	LOC101389164
FHL3	Fhl3	LOC101399464	PITPNB	Pitpnb	LOC101387722	XAB2	Xab2	LOC101388136
FHL5	Fhl5	LOC101401018	PITPNC1	Pitpnc1	LOC101397177	XAF1	Xaf1	LOC101391848
FHOD1	Fhod1	LOC101389214	PITPNM1	Pitpnm1	LOC101407979	XBP1	Xbp1	LOC101408303
FHOD3	Fhod3	LOC101401811	PITPNM2	Pitpnm2	LOC101390988	XCL1	Xcl1	LOC101387445
FIBCD1	Fibcd1	LOC101389002	PITPNM3	Pitpnm3	LOC101405777	XDH	Xdh	LOC101394861
FIBIN	Fibin	LOC101397707	PITRM1	Pitrm1	LOC101389200	XIAP	Xiap	LOC101396043
FIBP	Fibp	LOC101388516	PITX1	Pitx1	LOC101392741	XIRP1	Xirp1	LOC101407911
FICD	Ficd	LOC101392173	PITX2	Pitx2	LOC101397834	XIRP2	Xirp2	LOC101406199
FIG4	Fig4	LOC101395904	PITX3	Pitx3	LOC101389793	XK	Xk	LOC101389541
FIGLA	Figla	LOC101397781	PIWIL1	Piwil1	LOC101398518	XKR5	Xkr5	LOC101398226
FIGN	Fign	LOC101389880	PIWIL2	Piwil2	LOC101405041	XKR7	Xkr7	LOC101396340
FILIP1	Filip1	LOC101402067	PIWIL4	Piwil4	LOC101395498	XKR8	Xkr8	LOC101405935
FILIP1L	Filip1l	LOC101406679	PJA1	Pja1	LOC101389834	XKR9	Xkr9	LOC101397096
FIP1L1	Fip1l1	LOC101389654	PJA2	Pja2	LOC101405563	XKRX	Xkrx	LOC101401885
FIS1	Fis1	LOC101388037	PJVK	Dfnb59	LOC101406632	XPA	Xpa	LOC101388652
FITM1	Fitm1	LOC101397631	PKD1	Pkd1	LOC101395529	XPC	Xpc	LOC101389765
FITM2	Fitm2	LOC101393556	PKD1L1	Pkd1l1	LOC101402638	XPNPEP1	Xpnpep1	LOC101403574
FIZ1	Fiz1	LOC101391773	PKD1L2	Pkd1l2	LOC101398972	XPNPEP2	Xpnpep2	LOC101392026
FKBP10	Fkbp10	LOC101404475	PKD1L3	Pkd1l3	LOC101404562	XPNPEP3	Xpnpep3	LOC101402214
FKBP11	Fkbp11	LOC101398684	PKD2	Pkd2	LOC101405766	XPO1	Xpo1	LOC101394491
FKBP14	Fkbp14	LOC101388282	PKD2L1	Pkd2l1	LOC101400607	XPO4	Xpo4	LOC101400725
FKBP15	Fkbp15	LOC101394538	PKD2L2	Pkd2l2	LOC101409112	XPO5	Xpo5	LOC101399720
FKBP1A	Fkbp1a	LOC101395514	PKDCC	Pkdcc	LOC101405892	XPO6	Xpo6	LOC101403525
FKBP1B	Fkbp1b	LOC101397536	PKHD1	Pkhd1	LOC101395400	XPO7	Xpo7	LOC101387775
FKBP2	Fkbp2	LOC101390852	PKHD1L1	Pkhd1l1	LOC101407874	XPOT	Xpot	LOC101407250
FKBP3	Fkbp3	LOC101402988	PKIA	Pkia	LOC101406195	XPR1	Xpr1	LOC101407759

FKBP4	Fkbp4	LOC101392826	PKIB	Pkib	LOC101390883	XRCC1	Xrcc1	LOC101387842
FKBP5	Fkbp5	LOC101408370	PKIG	Pkig	LOC101391744	XRCC2	Xrcc2	LOC101403840
FKBP6	Fkbp6	LOC101392053	PKLR	Pklr	LOC101394494	XRCC3	Xrcc3	LOC101399493
FKBP7	Fkbp7	LOC101407058	PKM	Pkm	LOC101405917	XRCC4	Xrcc4	LOC101400303
FKBP8	Fkbp8	LOC101388052	PKMYT1	Pkmyt1	LOC101404585	XRCC5	Xrcc5	LOC101403758
FKBP9	Fkbp9	LOC101403710	PKN1	Pkn1	LOC101390880	XRCC6	Xrcc6	LOC101406580
FKRP	Fkrp	LOC101394770	PKN2	Pkn2	LOC101404341	XRN1	Xrn1	LOC101396497
FKTN	Fktn	LOC101405319	PKN3	Pkn3	LOC101399888	XRRA1	Xrra1	LOC101399748
FLAD1	Flad1	LOC101408153	PKNOX1	Pknox1	LOC101400081	XXYLT1	Xxylt1	LOC101406279
FLCN	Flcn	LOC101388950	PKNOX2	Pknox2	LOC101396133	XYLB	Xylb	LOC101403799
FLI1	Fli1	LOC101403346	PKP1	Pkp1	LOC101402218	XYLT1	Xylt1	LOC101399533
FLII	Flii	LOC101392889	PKP2	Pkp2	LOC101402143	XYLT2	Xylt2	LOC101391430
FLNA	Flna	LOC101392760	PKP3	Pkp3	LOC101389067	YAE1	Yae1d1	LOC101396769
FLNB	Flnb	LOC101394775	PKP4	Pkp4	LOC101397920	YAF2	Yaf2	LOC101404452
FLNC	Flnc	LOC101394596	PLA1A	Pla1a	LOC101405000	YAP1	Yap1	LOC101388831
FLOT1	Flot1	LOC101405591	PLA2G10	Pla2g10	LOC101400573	YARS2	Yars2	LOC101402402
FLOT2	Flot2	LOC101392549	PLA2G12A	Pla2g12a	LOC101400779	YBEY	Ybey	LOC101406128
FLRT3	Flrt3	LOC101393390	PLA2G12B	Pla2g12b	LOC101394903	YBX1	Ybx1	LOC101407928
FLT1	Flt1	LOC101389222	PLA2G15	Pla2g15	LOC101399041	YBX2	Ybx2	LOC101408660
FLT3	Flt3	LOC101388431	PLA2G1B	Pla2g1b	LOC101404894	YBX3	Ybx3	LOC101389392
FLT4	Flt4	LOC101408553	PLA2G2C	Pla2g2c	LOC101390398	YDJC	Ydjc	LOC101404259
FLVCR1	Mfsd7b	LOC101390897	PLA2G2D	Pla2g2d	LOC101390648	YEATS2	Yeats2	LOC101398345
FLYWCH2	Flywch2	LOC101392716	PLA2G2E	Pla2g2e	LOC101390135	YEATS4	Yeats4	LOC101393140
FMC1	1110001J03R	LOC101389372	PLA2G2F	Pla2g2f	LOC101390137	YES1	Yes1	LOC101406689
FMN1	Fmn1	LOC101404257	PLA2G3	Pla2g3	LOC101392275	YIF1A	Yif1a	LOC101393836
FMN2	Fmn2	LOC101400389	PLA2G4A	Pla2g4a	LOC101394289	YIF1B	Yif1b	LOC101401980
FMNL1	Fmnl1	LOC101407882	PLA2G4B	Pla2g4b	LOC101399960	YIPF1	Yipf1	LOC101391352
FMNL2	Fmnl2	LOC101406625	PLA2G4D	Pla2g4d	LOC101400935	YIPF2	Yipf2	LOC101391895
FMNL3	Fmnl3	LOC101405414	PLA2G4E	Pla2g4e	LOC101400669	YIPF3	Yipf3	LOC101400238
FMO1	Fmo1	LOC101404179	PLA2G4F	Pla2g4f	LOC101401457	YIPF4	Yipf4	LOC101389307
FMO2	Fmo2	LOC101404624	PLA2G5	Pla2g5	LOC101389873	YIPF5	Yipf5	LOC101402567
FMO3	Fmo3	LOC101396436	PLA2G6	Pla2g6	LOC101389231	YIPF6	Yipf6	LOC101391364
FMO4	Fmo4	LOC101403910	PLA2G7	Pla2g7	LOC101390640	YIPF7	Yipf7	LOC101402394
FMO5	Fmo5	LOC101391624	PLA2R1	Pla2r1	LOC101394818	YJEFN3	Yjefn3	LOC101397991
FMOD	Fmod	LOC101407233	PLAA	Plaa	LOC101398766	YJU2	Ccdc94	LOC101394605
FMR1	Fmr1	LOC101398404	PLAC8	Plac8	LOC101401590	YKT6	Ykt6	LOC101388875
FN1	Fn1	LOC101401138	PLAC8L1	Plac8l1	LOC101401010	YLPM1	Ylpm1	LOC101407057
FN3K	Fn3k	LOC101405775	PLAG1	Plag1	LOC101392457	YME1L1	Yme1l1	LOC101396791
FN3KRP	Fn3krp	LOC101405517	PLAGL2	Plagl2	LOC101388901	YPEL1	Ypel1	LOC101403463
FNBP1	Fnbp1	LOC101407752	PLAT	Plat	LOC101391883	YPEL2	Ypel2	LOC101389134
FNBP1L	Fnbp1l	LOC101404150	PLAU	Plau	LOC101408730	YPEL3	Ypel3	LOC101388616
FNBP4	Fnbp4	LOC101408322	PLAUR	Plaur	LOC101389060	YPEL4	Ypel4	LOC101390000
FNDC1	Fndc1	LOC101406939	PLB1	Plb1	LOC101396139	YPEL5	Ypel5	LOC101394095
FNDC3A	Fndc3a	LOC101391424	PLBD1	Plbd1	LOC101397520	YRDC	Yrdc	LOC101398004
FNDC3B	Fndc3b	LOC101390047	PLBD2	Plbd2	LOC101391240	YTHDC1	Ythdc1	LOC101401270
FNDC4	Fndc4	LOC101401178	PLCB1	Plcb1	LOC101399055	YTHDC2	Ythdc2	LOC101400657
FNDC5	Fndc5	LOC101404012	PLCB2	Plcb2	LOC101389327	YTHDF1	Ythdf1	LOC101396540
FNDC7	Fndc7	LOC101408522	PLCB3	Plcb3	LOC101391763	YTHDF2	Ythdf2	LOC101388826
FNDC8	Fndc8	LOC101392748	PLCB4	Plcb4	LOC101398439	YTHDF3	Ythdf3	LOC101396828
FNDC9	Fndc9	LOC101388073	PLCD1	Plcd1	LOC101401189	YWHAB	Ywhab	LOC101390494
FNIP1	Fnip1	LOC101405029	PLCD3	Plcd3	LOC101399308	YWHAE	Ywhae	LOC101405345
FNIP2	Fnip2	LOC101394189	PLCD4	Plcd4	LOC101387297	YWHAG	Ywhag	LOC101405915
FNTA	Fnta	LOC101396330	PLCE1	Plce1	LOC101400955	YWHAH	Ywhah	LOC101408477
FNTB	Fntb	LOC101403045	PLCG1	Plcg1	LOC101398275	YWHAQ	Ywhaq	LOC101405442

FOCAD	Focad	LOC101403163	PLCG2	Plcg2	LOC101397947	YWHAZ	Ywhaz	LOC101387644
FOLR2	Folr2	LOC101389626	PLCH1	Plch1	LOC101405306	YY1	Yy1	LOC101389903
FOPNL	Fopnl	LOC106803765	PLCH2	Plch2	LOC101398422	YY2	Yy2	LOC106802617
FOS	Fos	LOC101388064	PLCL1	Plcl1	LOC101406633	ZADH2	Zadh2	LOC101397306
FOSB	Fosb	LOC101397018	PLCL2	Plcl2	LOC101400918	ZAN	Zan	LOC101403024
FOSL1	Fosl1	LOC101388271	PLCXD1	Plcxd1	LOC101408946	ZAP70	Zap70	LOC101401514
FOSL2	Fosl2	LOC101397104	PLCXD2	Plcxd2	LOC101394932	ZAR1L	Zar1l	LOC101394489
FOXA1	Foxa1	LOC101408416	PLCXD3	Plcxd3	LOC101390639	ZBBX	Zbbx	LOC101399801
FOXA2	Foxa2	LOC101399958	PLCZ1	Plcz1	LOC101405092	ZBED6	Zbed6	LOC106800023
FOXA3	Foxa3	LOC101401358	PLD1	Pld1	LOC101390308	ZBTB1	Zbtb1	LOC101401661
FOXB1	Foxb1	LOC101399358	PLD2	Pld2	LOC101395689	ZBTB10	Zbtb10	LOC101398184
FOXB2	Foxb2	LOC101407283	PLD3	Pld3	LOC101393942	ZBTB11	Zbtb11	LOC101388620
FOXC2	Foxc2	LOC101390597	PLD4	Pld4	LOC101391004	ZBTB12	Zbtb12	LOC101391150
FOXE1	Foxe1	LOC101388915	PLD5	Pld5	LOC101393247	ZBTB14	Zbtb14	LOC101400929
FOXF1	Foxf1	LOC101391263	PLEC	Plec	LOC101392926	ZBTB16	Zbtb16	LOC101387802
FOXF2	Foxf2	LOC101397114	PLEK	Plek	LOC101405789	ZBTB17	Zbtb17	LOC101400865
FOXH1	Foxh1	LOC101387781	PLEK2	Plek2	LOC101407237	ZBTB18	Zbtb18	LOC101395451
FOXI1	Foxi1	LOC101397059	PLEKHA1	Plekha1	LOC101401925	ZBTB2	Zbtb2	LOC101403183
FOXI2	Foxi2	LOC101390092	PLEKHA2	Plekha2	LOC101394640	ZBTB20	Zbtb20	LOC101408321
FOXI3	Foxi3	LOC101401612	PLEKHA3	Plekha3	LOC101407330	ZBTB21	Zbtb21	LOC101396984
FOXJ1	Foxj1	LOC101403678	PLEKHA4	Plekha4	LOC101396143	ZBTB22	Zbtb22	LOC101397748
FOXJ2	Foxj2	LOC101391267	PLEKHA5	Plekha5	LOC101404041	ZBTB24	Zbtb24	LOC101395475
FOXJ3	Foxj3	LOC101403286	PLEKHA6	Plekha6	LOC101402779	ZBTB25	Zbtb25	LOC101401396
FOXK1	Foxk1	LOC101394181	PLEKHA7	Plekha7	LOC101399944	ZBTB26	Zbtb26	LOC106800758
FOXK2	Foxk2	LOC101406993	PLEKHA8	Plekha8	LOC101387684	ZBTB3	Zbtb3	LOC101402121
FOXL1	Foxl1	LOC101390347	PLEKHB1	Plekhb1	LOC101394234	ZBTB32	Zbtb32	LOC106803442
FOXL2	Foxl2	LOC101392584	PLEKHB2	Plekhb2	LOC101403651	ZBTB33	Zbtb33	LOC101400280
FOXM1	Foxm1	LOC101392122	PLEKHD1	Plekhd1	LOC101391062	ZBTB34	Zbtb34	LOC101406440
FOXN1	Foxn1	LOC101397685	PLEKHF1	Plekhf1	LOC101396670	ZBTB37	Zbtb37	LOC101398756
FOXN2	Foxn2	LOC101393398	PLEKHF2	Plekhf2	LOC101401493	ZBTB38	Zbtb38	LOC101399248
FOXN3	Foxn3	LOC101394663	PLEKHG1	Plekhg1	LOC101402654	ZBTB39	Zbtb39	LOC101388157
FOXN4	Foxn4	LOC101395415	PLEKHG2	Plekhg2	LOC101388716	ZBTB4	Zbtb4	LOC101403680
FOXO1	Foxo1	LOC101394452	PLEKHG3	Plekhg3	LOC101402345	ZBTB40	Zbtb40	LOC101398347
FOXO3	Foxo3	LOC101392149	PLEKHG4	Plekhg4	LOC101401676	ZBTB41	Zbtb41	LOC101407278
FOXO4	Foxo4	LOC101407112	PLEKHG5	Plekhg5	LOC101401579	ZBTB42	Zbtb42	LOC101403239
FOXO6	Foxo6	LOC101390978	PLEKHG6	Plekhg6	LOC101407806	ZBTB43	Zbtb43	LOC101406013
FOXP1	Foxp1	LOC101408860	PLEKHH1	Plekhh1	LOC101407765	ZBTB44	Zbtb44	LOC101407644
FOXP2	Foxp2	LOC101391778	PLEKHH2	Plekhh2	LOC101403082	ZBTB45	Zbtb45	LOC101407280
FOXP3	Foxp3	LOC101402373	PLEKHH3	Plekhh3	LOC101405787	ZBTB46	Zbtb46	LOC101391654
FOXP4	Foxp4	LOC101394711	PLEKHJ1	Plekhj1	LOC101406426	ZBTB48	Zbtb48	LOC101402954
FOXR1	Foxr1	LOC101393801	PLEKHM1	Plekhm1	LOC101408917	ZBTB49	Zbtb49	LOC101407348
FOXRED1	Foxred1	LOC101401262	PLEKHM2	Plekhm2	LOC101399379	ZBTB5	Zbtb5	LOC101408195
FOXRED2	Foxred2	LOC101395793	PLEKHM3	Plekhm3	LOC101403047	ZBTB6	Zbtb6	LOC101397147
FOXS1	Foxs1	LOC101391046	PLEKHN1	Plekhn1	LOC101391727	ZBTB7B	Zbtb7b	LOC101408414
FPGS	Fpgs	LOC101389513	PLEKHO1	Plekho1	LOC101388772	ZBTB7C	Zbtb7c	LOC101389947
FPGT	Fpgt	LOC106799934	PLEKHO2	Plekho2	LOC101388301	ZBTB8A	Zbtb8a	LOC101402172
FRA10AC1	Fra10ac1	LOC101399812	PLEKHS1	Plekhs1	LOC101389107	ZBTB8OS	Zbtb8os	LOC101402431
FRAS1	Fras1	LOC101388675	PLGRKT	Plgrkt	LOC101395353	ZBTB9	Zbtb9	LOC101395227
FREM1	Frem1	LOC101409090	PLIN1	Plin1	LOC101401415	ZC2HC1A	Zc2hc1a	LOC101406636
FREM2	Frem2	LOC101387918	PLIN2	Plin2	LOC101404915	ZC2HC1B	Zc2hc1b	LOC101393501
FREM3	Frem3	LOC101391131	PLIN3	Plin3	LOC101391284	ZC2HC1C	Zc2hc1c	LOC101390561
FRG1	Frg1	LOC101392538	PLK1	Plk1	LOC101408267	ZC3H10	Zc3h10	LOC101398685
FRK	Frk	LOC101403636	PLK2	Plk2	LOC101407044	ZC3H11A	Zc3h11a	LOC101404882
FRMD4A	Frmd4a	LOC101393447	PLK3	Plk3	LOC101392492	ZC3H12A	Zc3h12a	LOC101395233

FRMD4B	Frmd4b	LOC101407291	PLK4	Plk4	LOC101387975	ZC3H12B	Zc3h12b	LOC101394598
FRMD5	Frmd5	LOC101390118	PLK5	Plk5	LOC101408258	ZC3H12C	Zc3h12c	LOC101400074
FRMD6	Frmd6	LOC101396039	PLLP	Pllp	LOC101392959	ZC3H12D	Zc3h12d	LOC101395721
FRMD7	Frmd7	LOC101407364	PLOD1	Plod1	LOC101395837	ZC3H13	Zc3h13	LOC101409080
FRMD8	Frmd8	LOC101404486	PLOD2	Plod2	LOC101393755	ZC3H14	Zc3h14	LOC101402783
FRMPD1	Frmpd1	LOC101407139	PLOD3	Plod3	LOC101388805	ZC3H15	Zc3h15	LOC101392349
FRMPD2	Gm626	LOC101387730	PLP1	Plp1	LOC101388648	ZC3H18	Zc3h18	LOC101388010
FRMPD3	Frmpd3	LOC101403448	PLP2	Plp2	LOC101404572	ZC3H3	Zc3h3	LOC101396161
FRMPD4	Frmpd4	LOC101387568	PLPBP	Prosc	LOC101388407	ZC3H4	Zc3h4	LOC101405809
FRRS1	Frrs1	LOC101396072	PLPP1	Plpp1	LOC101401816	ZC3H6	Zc3h6	LOC101391008
FRRS1L	Frrs1l	LOC101408719	PLPP2	Plpp2	LOC101394438	ZC3H7A	Zc3h7a	LOC101406336
FRS2	Frs2	LOC101393636	PLPP3	Plpp3	LOC101397869	ZC3H7B	Zc3h7b	LOC101404227
FRS3	Frs3	LOC101393198	PLPP4	Plpp4	LOC101399472	ZC3H8	Zc3h8	LOC101391259
FRY	Fry	LOC101394080	PLPP5	Plpp5	LOC101391836	ZC3HAV1	Zc3hav1	LOC101394731
FRYL	Fryl	LOC101408250	PLPP6	Plpp6	LOC101397622	ZC3HAV1L	Zc3hav1l	LOC101394992
FRZB	Frzb	LOC101390905	PLPP7	Plpp7	LOC101390044	ZC3HC1	Zc3hc1	LOC101390442
FSCN1	Fscn1	LOC101391371	PLPPR1	Plppr1	LOC101395132	ZC4H2	Zc4h2	LOC101394865
FSCN2	Fscn2	LOC101395342	PLPPR2	Plppr2	LOC101394117	ZCCHC10	Zcchc10	LOC101398067
FSCN3	Fscn3	LOC101399079	PLPPR3	Plppr3	LOC101391885	ZCCHC12	Zcchc12	LOC101394961
FSD1	Fsd1	LOC101393939	PLPPR4	Plppr4	LOC101396598	ZCCHC13	Zcchc13	LOC101400218
FSD1L	Fsd1l	LOC101405056	PLPPR5	Plppr5	LOC101397029	ZCCHC14	Zcchc14	LOC101389823
FSD2	Fsd2	LOC101390997	PLRG1	Plrg1	LOC101407832	ZCCHC17	Zcchc17	LOC101393038
FSHB	Fshb	LOC101400478	PLS1	Pls1	LOC101395977	ZCCHC18	Zcchc18	LOC101400754
FSHR	Fshr	LOC101391012	PLS3	Pls3	LOC101402484	ZCCHC2	Zcchc2	LOC101409131
FSIP1	Fsip1	LOC101387697	PLSCR1	Plscr2	LOC101389494	ZCCHC24	Zcchc24	LOC101396972
FSIP2	Fsip2	LOC101406713	PLSCR3	Plscr3	LOC101406215	ZCCHC4	Zcchc4	LOC101397590
FST	Fst	LOC101390302	PLSCR4	Plscr4	LOC101389753	ZCCHC7	Zcchc7	LOC101408718
FSTL1	Fstl1	LOC101402118	PLSCR5	Plscr5	LOC101393495	ZCCHC8	Zcchc8	LOC101387897
FSTL4	Fstl4	LOC101397545	PLTP	Pltp	LOC101402970	ZCCHC9	Zcchc9	LOC101402488
FSTL5	Fstl5	LOC101391797	PLVAP	Plvap	LOC101402680	ZCRB1	Zcrb1	LOC101405413
FTCD	Ftcd	LOC101388332	PLXDC1	Plxdc1	LOC101407531	ZCWPW1	Zcwpw1	LOC101392501
FTH1	Fth1	LOC101396226	PLXDC2	Plxdc2	LOC101405932	ZCWPW2	Zcwpw2	LOC101387846
FTL	Ftl1	LOC101398223	PLXNA1	Plxna1	LOC101407316	ZDBF2	Zdbf2	LOC101392084
FTMT	Ftmt	LOC101393763	PLXNA2	Plxna2	LOC101402778	ZDHHC1	Zdhhc1	LOC101390667
FTO	Fto	LOC101405771	PLXNA3	Plxna3	LOC101395738	ZDHHC12	Zdhhc12	LOC101400329
FTSJ1	Ftsj1	LOC101392455	PLXNA4	Plxna4	LOC101402446	ZDHHC13	Zdhhc13	LOC101392470
FTSJ3	Ftsj3	LOC101392182	PLXNB1	Plxnb1	LOC101389839	ZDHHC14	Zdhhc14	LOC106803576
FUBP1	Fubp1	LOC101403062	PLXNB2	Plxnb2	LOC101407101	ZDHHC15	Zdhhc15	LOC101400749
FUBP3	Fubp3	LOC101387787	PLXNB3	Plxnb3	LOC101409041	ZDHHC16	Zdhhc16	LOC101390319
FUCA1	Fuca1	LOC101405934	PLXNC1	Plxnc1	LOC101408761	ZDHHC17	Zdhhc17	LOC101392828
FUCA2	Fuca2	LOC101394434	PLXND1	Plxnd1	LOC101401639	ZDHHC18	Zdhhc18	LOC101397404
FUNDC1	Fundc1	LOC101402114	PM20D1	Pm20d1	LOC101402689	ZDHHC2	Zdhhc2	LOC101399814
FUNDC2	Fundc2	LOC101403206	PM20D2	Pm20d2	LOC101395392	ZDHHC20	Zdhhc20	LOC101401766
FUOM	Fuom	LOC101393922	PMAIP1	Pmaip1	LOC101407476	ZDHHC21	Zdhhc21	LOC101387487
FURIN	Furin	LOC101387560	PMCH	Pmch	LOC101402387	ZDHHC22	Zdhhc22	LOC101393127
FUS	Fus	LOC101406235	PMEL	Pmel	LOC101395590	ZDHHC23	Zdhhc23	LOC101388868
FUT10	Fut10	LOC101407507	PMF1	Pmf1	LOC101404119	ZDHHC24	Zdhhc24	LOC101397788
FUT11	Fut11	LOC101389972	PMFBP1	Pmfbp1	LOC101406650	ZDHHC3	Zdhhc3	LOC101398988
FUT4	Fut4	LOC101395758	PML	Pml	LOC101389674	ZDHHC4	Zdhhc4	LOC101389565
FUT7	Fut7	LOC101388055	PMM1	Pmm1	LOC101406334	ZDHHC5	Zdhhc5	LOC101390685
FUT8	Fut8	LOC101404959	PMM2	Pmm2	LOC101388356	ZDHHC6	Zdhhc6	LOC101387455
FUZ	Fuz	LOC101387424	PMP2	Pmp2	LOC101409149	ZDHHC7	Zdhhc7	LOC101392385
FXN	Fxn	LOC101399771	PMP22	Pmp22	LOC101403591	ZDHHC8	Zdhhc8	LOC101392326
FXR1	Fxr1	LOC101401393	PMPCA	Pmpca	LOC101403386	ZDHHC9	Zdhhc9	LOC101391347

FXR2	Fxr2	LOC101400363	PMPCB	Pmpcb	LOC101398553	ZEB1	Zeb1	LOC101393036
FXYD1	Fxyd1	LOC101395278	PMS1	Pms1	LOC101397321	ZEB2	Zeb2	LOC101404004
FXYD2	Fxyd2	LOC101401738	PMS2	Pms2	LOC101387850	ZER1	Zer1	LOC101400592
FXYD3	Fxyd3	LOC101401624	PMVK	Pmvk	LOC101406661	ZFAND1	Zfand1	LOC101388567
FXYD4	Fxyd4	LOC106802175	PNCK	Pnck	LOC101407221	ZFAND2A	Zfand2a	LOC101398645
FXYD5	Fxyd5	LOC101391274	PNISR	Pnisr	LOC101403733	ZFAND2B	Zfand2b	LOC101392433
FXYD6	Fxyd6	LOC101401475	PNKD	Pnkd	LOC106799928	ZFAND3	Zfand3	LOC101405060
FXYD7	Fxyd7	LOC101396401	PNKP	Pnkp	LOC101388110	ZFAND4	Zfand4	LOC101404377
FYB1	Fyb	LOC101387436	PNLDC1	Pnldc1	LOC101408859	ZFAND5	Zfand5	LOC101388279
FYB2	1700024P16F	LOC101405010	PNLIP	Pnlip	LOC106800133	ZFAND6	Zfand6	LOC101388903
FYCO1	Fyco1	LOC101401972	PNMA1	Pnma1	LOC101401397	ZFAT	Zfat	LOC101388415
FYN	Fyn	LOC101400676	PNMA2	Pnma2	LOC101405521	ZFC3H1	Zfc3h1	LOC101397502
FYTTD1	Fyttd1	LOC101404440	PNMT	Pnmt	LOC101403336	ZFHX2	Zfhx2	LOC101401196
FZD1	Fzd1	LOC101396461	PNN	Pnn	LOC101405792	ZFHX3	Zfhx3	LOC101406395
FZD10	Fzd10	LOC101398273	PNO1	Pno1	LOC101406571	ZFHX4	Zfhx4	LOC101405496
FZD2	Fzd2	LOC101395163	PNOC	Pnoc	LOC101399580	ZFP1	Zfp1	LOC101406740
FZD3	Fzd3	LOC101398289	PNPLA1	Pnpla1	LOC101405745	ZFP28	Zfp28	LOC101400386
FZD4	Fzd4	LOC101398612	PNPLA2	Pnpla2	LOC101392132	ZFP3	Zfp3	LOC101400973
FZD5	Fzd5	LOC101402786	PNPLA3	Pnpla3	LOC101397952	ZFP30	Zfp30	LOC101388705
FZD6	Fzd6	LOC101391926	PNPLA6	Pnpla6	LOC101388389	ZFP36	Zfp36	LOC101388458
FZD7	Fzd7	LOC101391065	PNPLA7	Pnpla7	LOC101394805	ZFP36L1	Zfp36l1	LOC101388063
FZR1	Fzr1	LOC101399346	PNPLA8	Pnpla8	LOC101401179	ZFP36L2	Zfp36l2	LOC101403783
G0S2	G0s2	LOC101403566	PNPO	Pnpo	LOC101392707	ZFP37	Zfp37	LOC101408279
G2E3	G2e3	LOC101407303	PNPT1	Pnpt1	LOC101399405	ZFP42	Zfp42	LOC101392004
G3BP1	G3bp1	LOC101394988	PNRC1	Pnrc1	LOC101395128	ZFP64	Zfp64	LOC101389117
G3BP2	G3bp2	LOC101403931	PNRC2	Pnrc2	LOC101406447	ZFP69	Zfp69	LOC101408800
G6PC	G6pc	LOC101389628	POC1A	Poc1a	LOC101399608	ZFP90	Zfp90	LOC101400708
G6PC2	G6pc2	LOC101404289	POC1B	Poc1b	LOC101403088	ZFP91	Zfp91	LOC101399148
G6PC3	G6pc3	LOC101390756	POC5	Poc5	LOC101390371	ZFP92	Zfp92	LOC101397986
G6PD	G6pdx	LOC101397039	PODN	Podn	LOC101388964	ZFPL1	Zfpl1	LOC101400731
GAA	Gaa	LOC101391932	PODNL1	Podnl1	LOC101398238	ZFPM1	Zfpm1	LOC101389734
GAB1	Gab1	LOC101393506	PODXL	Podxl	LOC101399911	ZFPM2	Zfpm2	LOC101394561
GAB2	Gab2	LOC101388258	PODXL2	Podxl2	LOC101403464	ZFR	Zfr	LOC101396517
GAB3	Gab3	LOC101401466	POF1B	Pof1b	LOC101394085	ZFR2	Zfr2	LOC101400402
GABARAP	Gabarap	LOC101388171	POFUT1	Pofut1	LOC101388635	ZFX	Zfx	LOC101387394
GABARAPL1	Gabarapl1	LOC101406338	POFUT2	Pofut2	LOC101405605	ZFYVE1	Zfyve1	LOC101397492
GABARAPL2	Gabarapl2	LOC101404579	POGK	Pogk	LOC101390645	ZFYVE16	Zfyve16	LOC101404512
GABBR1	Gabbr1	LOC101399172	POGLUT1	Poglut1	LOC101406062	ZFYVE19	Zfyve19	LOC101393858
GABBR2	Gabbr2	LOC101390810	POGLUT2	Kdelc1	LOC101406009	ZFYVE21	Zfyve21	LOC101399750
GABPA	Gabpa	LOC101390985	POGLUT3	Kdelc2	LOC101401137	ZFYVE26	Zfyve26	LOC101387542
GABPB1	Gabpb1	LOC101404416	POGZ	Pogz	LOC101403601	ZFYVE27	Zfyve27	LOC101393376
GABPB2	Gabpb2	LOC101397864	POLA1	Pola1	LOC101388348	ZFYVE28	Zfyve28	LOC101396641
GABRA1	Gabra1	LOC101401054	POLA2	Pola2	LOC101403432	ZFYVE9	Zfyve9	LOC101407804
GABRA2	Gabra2	LOC101403615	POLB	Polb	LOC101392742	ZG16	Zg16	LOC101395535
GABRA3	Gabra3	LOC101406004	POLD2	Pold2	LOC101387931	ZGLP1	Zglp1	LOC101387520
GABRA4	Gabra4	LOC101404134	POLD3	Pold3	LOC101398291	ZGPAT	Zgpat	LOC101391922
GABRA5	Gabra5	LOC101393353	POLD4	Pold4	LOC101404487	ZGRF1	Zgrf1	LOC101396795
GABRA6	Gabra6	LOC101401669	POLDIP2	Poldip2	LOC101399578	ZHX1	Zhx1	LOC106799933
GABRB1	Gabrb1	LOC101404760	POLDIP3	Poldip3	LOC101394418	ZHX3	Zhx3	LOC101398020
GABRB2	Gabrb2	LOC101401927	POLE	Pole	LOC101407514	ZIC1	Zic1	LOC101392839
GABRB3	Gabrb3	LOC101393604	POLE2	Pole2	LOC101400893	ZIC2	Zic2	LOC101399717
GABRD	Gabrd	LOC101395057	POLE3	Pole3	LOC101397233	ZIC3	Zic3	LOC101403885
GABRE	Gabre	LOC101400503	POLE4	Pole4	LOC101404047	ZIC4	Zic4	LOC101393094
GABRG1	Gabrg1	LOC101403356	POLG	Polg	LOC101401677	ZIC5	Zic5	LOC101401998

GABRG2	Gabrg2	LOC101400608	POLG2	Polg2	LOC101394833	ZKSCAN1	Zkscan1	LOC106803953
GABRG3	Gabrg3	LOC101393110	POLH	Polh	LOC101399462	ZKSCAN2	Zkscan2	LOC101401967
GABRP	Gabrp	LOC101395330	POLI	Poli	LOC101397643	ZKSCAN3	Zkscan3	LOC101389426
GABRQ	Gabrq	LOC101407042	POLK	Polk	LOC101390620	ZKSCAN5	Zkscan5	LOC101405728
GABRR1	Gabrr1	LOC101395651	POLL	Poll	LOC101388834	ZKSCAN7	Zkscan7	LOC101397805
GABRR2	Gabrr2	LOC101395902	POLM	Polm	LOC101387670	ZKSCAN8	Zkscan8	LOC101391626
GAD1	Gad1	LOC101399729	POLN	Poln	LOC101396114	ZMAT1	Zmat1	LOC101390126
GAD2	Gad2	LOC101400599	POLQ	Polq	LOC101400275	ZMAT2	Zmat2	LOC101389496
GADD45A	Gadd45a	LOC101391748	POLR1A	Polr1a	LOC101391346	ZMAT3	Zmat3	LOC101407405
GADD45B	Gadd45b	LOC101404248	POLR1B	Polr1b	LOC101389817	ZMAT4	Zmat4	LOC101388981
GADD45G	Gadd45g	LOC101403495	POLR1C	Polr1c	LOC101399978	ZMAT5	Zmat5	LOC101401058
GADD45GIP1	Gadd45gip1	LOC101407466	POLR1E	Polr1e	LOC101407934	ZMIZ1	Zmiz1	LOC101397244
GADL1	Gadl1	LOC101389931	POLR2A	Polr2a	LOC101403154	ZMIZ2	Zmiz2	LOC101391193
GAK	Gak	LOC101391080	POLR2B	Polr2b	LOC101396141	ZMPSTE24	Zmpste24	LOC101407687
GAL	Gal	LOC101404407	POLR2C	Polr2c	LOC101394228	ZMYM1	Zmym1	LOC101387538
GAL3ST1	Gal3st1	LOC101396280	POLR2D	Polr2d	LOC101402163	ZMYM2	Zmym2	LOC101398294
GAL3ST3	Gal3st3	LOC101391764	POLR2F	Polr2f	LOC101390266	ZMYM3	Zmym3	LOC101403971
GAL3ST4	Gal3st4	LOC101391463	POLR2G	Polr2g	LOC101402381	ZMYM4	Zmym4	LOC101388061
GALC	Galc	LOC101400778	POLR2H	Polr2h	LOC101395056	ZMYM5	Zmym5	LOC101397860
GALE	Gale	LOC101405230	POLR2I	Polr2i	LOC101394595	ZMYM6	Zmym6	LOC101409143
GALK2	Galk2	LOC101402412	POLR2J	Polr2j	LOC101407573	ZMYND10	Zmynd10	LOC101391451
GALM	Galm	LOC101403175	POLR2K	Polr2k	LOC101408128	ZMYND11	Zmynd11	LOC101390901
GALNS	Galns	LOC101408419	POLR2L	Polr2l	LOC101391372	ZMYND12	Zmynd12	LOC101403814
GALNT1	Galnt1	LOC101399363	POLR2M	Polr2m	LOC106800482	ZMYND15	Zmynd15	LOC101394313
GALNT10	Galnt10	LOC101392352	POLR3A	Polr3a	LOC101398424	ZMYND19	Zmynd19	LOC101395312
GALNT11	Galnt11	LOC101404368	POLR3B	Polr3b	LOC101409017	ZMYND8	Zmynd8	LOC101397670
GALNT12	Galnt12	LOC101398848	POLR3C	Polr3c	LOC101388030	ZNF407	Zfp407	LOC101397040
GALNT13	Galnt13	LOC101397236	POLR3D	Polr3d	LOC101405468	ZNF408	Zfp408	LOC101395963
GALNT14	Galnt14	LOC101392830	POLR3E	Polr3e	LOC101397557	ZNF410	Zfp410	LOC101402173
GALNT15	Galnt15	LOC101399692	POLR3F	Polr3f	LOC101387739	ZNF414	Zfp414	LOC101401903
GALNT16	Galnt16	LOC101389785	POLR3G	Polr3g	LOC101395551	ZNF419	Zfp954	LOC101405281
GALNT18	Galnt18	LOC101397014	POLR3GL	Polr3gl	LOC101407919	ZNF423	Zfp423	LOC101401937
GALNT2	Galnt2	LOC101395407	POLR3H	Polr3h	LOC101405899	ZNF428	Zfp428	LOC101388622
GALNT3	Galnt3	LOC101408900	POLR3K	Polr3k	LOC101408676	ZNF436	Zfp46	LOC101401916
GALNT4	Galnt4	LOC106799936	POLRMT	Polrmt	LOC101392586	ZNF438	Zfp438	LOC101393621
GALNT5	Galnt5	LOC101400347	POMC	Pomc	LOC101393749	ZNF444	Zfp444	LOC101396930
GALNT6	Galnt6	LOC101395149	POMGNT1	Pomgnt1	LOC101399700	ZNF445	Zfp445	LOC101397371
GALNT7	Galnt7	LOC101389536	POMP	Pomp	LOC101389476	ZNF446	Zfp446	LOC101399423
GALNT9	Galnt9	LOC101401059	POMT1	Pomt1	LOC101390556	ZNF449	Zfp449	LOC101398998
GALNTL5	Galntl5	LOC101407419	POMT2	Pomt2	LOC101393625	ZNF451	Zfp451	LOC101391817
GALNTL6	Galntl6	LOC101389289	PON1	Pon1	LOC101387277	ZNF462	Zfp462	LOC101406274
GALP	Galp	LOC101397202	PON2	Pon2	LOC101405080	ZNF467	Zfp467	LOC101392439
GALR1	Galr1	LOC101398843	PON3	Pon3	LOC101404821	ZNF473	Zfp473	LOC101404401
GALR2	Galr2	LOC101401680	POP1	Pop1	LOC101405511	ZNF48	Zfp553	LOC101396840
GALR3	Galr3	LOC101389146	POP4	Pop4	LOC101387941	ZNF483	Zkscan16	LOC101407227
GALT	Galt	LOC101406200	POP5	Pop5	LOC101402442	ZNF496	Zkscan17	LOC101400130
GAMT	Gamt	LOC106803685	POP7	Pop7	LOC101397303	ZNF605	Zfp605	LOC101408558
GAN	Gan	LOC101398448	POPDC2	Popdc2	LOC101404387	ZNF606	Zfp606	LOC101404232
GANAB	Ganab	LOC101399412	POPDC3	Popdc3	LOC101408187	ZNF608	Zfp608	LOC101390107
GANC	Ganc	LOC101402151	POR	Por	LOC101404609	ZNF609	Zfp609	LOC101409124
GAP43	Gap43	LOC101408057	PORCN	Porcn	LOC101391508	ZNF618	Zfp618	LOC101398584
GAPDH	Gapdh	LOC101405704	POSTN	Postn	LOC101400807	ZNF622	Zfp622	LOC101392508
GAPDHS	Gapdhs	LOC101391443	POT1	Pot1a	LOC101401000	ZNF629	Zfp629	LOC101401439
GAPT	Gapt	LOC101407478	POU1F1	Pou1f1	LOC101398819	ZNF638	Zfp638	LOC101395348

GAPVD1	Gapvd1	LOC101402945	POU2AF1	Pou2af1	LOC101397918	ZNF639	Zfp639	LOC106800953
GAR1	Gar1	LOC101400243	POU2F1	Pou2f1	LOC101388922	ZNF641	Zfp641	LOC101393301
GARNL3	Garnl3	LOC101407400	POU2F2	Pou2f2	LOC101405551	ZNF644	Zfp644	LOC101389938
GART	Gart	LOC101406981	POU2F3	Pou2f3	LOC101407601	ZNF646	Zfp646	LOC101404395
GAS2	Gas2	LOC101396319	POU3F2	Pou3f2	LOC101402676	ZNF652	Zfp652	LOC101402295
GAS2L1	Gas2l1	LOC101405337	POU3F4	Pou3f4	LOC101395870	ZNF653	Zfp653	LOC101396777
GAS2L2	Gas2l2	LOC101403512	POU4F1	Pou4f1	LOC101390966	ZNF654	Zfp654	LOC101397878
GAS2L3	Gas2l3	LOC101387417	POU4F2	Pou4f2	LOC101397974	ZNF655	Zfp655	LOC101406258
GAS6	Gas6	LOC101391985	POU4F3	Pou4f3	LOC101399437	ZNF668	Zfp668	LOC101404130
GAS7	Gas7	LOC101408222	POU5F1	Pou5f1	LOC101402859	ZNF687	Zfp687	LOC101401254
GAS8	Gas8	LOC101403083	POU5F2	Pou5f2	LOC106803170	ZNF688	Zfp688	LOC101398312
GASK1A	Fam198a	LOC101401631	POU6F1	Pou6f1	LOC101392950	ZNF689	Zfp689	LOC101398813
GASK1B	Fam198b	LOC101391457	POU6F2	Pou6f2	LOC101397026	ZNF691	Zfp691	LOC101406262
GAST	Gast	LOC101405786	PP2D1	Pp2d1	LOC101395375	ZNF692	Zfp692	LOC101399012
GATA1	Gata1	LOC101388863	PPA1	Ppa1	LOC101403303	ZNF697	Zfp697	LOC101394109
GATA2	Gata2	LOC101399357	PPA2	Ppa2	LOC101393627	ZNFX1	Znfx1	LOC101395678
GATA3	Gata3	LOC101398935	PPAN	Ppan	LOC101407308	ZNHIT1	Znhit1	LOC101388547
GATA4	Gata4	LOC101393148	PPARA	Ppara	LOC101402636	ZNHIT2	Znhit2	LOC101401957
GATA5	Gata5	LOC101398274	PPARD	Ppard	LOC101387626	ZNHIT3	Znhit3	LOC101395956
GATA6	Gata6	LOC101396254	PPARG	Pparg	LOC101388996	ZNHIT6	Znhit6	LOC101390575
GATAD1	Gatad1	LOC101398361	PPARGC1A	Ppargc1a	LOC101396025	ZNRD1	Znrd1	LOC101402406
GATAD2A	Gatad2a	LOC101402254	PPARGC1B	Ppargc1b	LOC101403141	ZNRD2	Sssca1	LOC101406829
GATAD2B	Gatad2b	LOC101396305	PPAT	Ppat	LOC101393848	ZNRF1	Znrf1	LOC101407441
GATB	Gatb	LOC101403274	PPCDC	Ppcdc	LOC101396868	ZNRF2	Znrf2	LOC101406750
GATC	Gatc	LOC101403926	PPCS	Ppcs	LOC101404265	ZNRF3	Znrf3	LOC101408037
GATD1	Pddc1	LOC101393342	PPDPF	Ppdpf	LOC101394144	ZP1	Zp1	LOC101393329
GATD3A	D10Jhu81e	LOC101404559	PPEF1	Ppef1	LOC101392809	ZP2	Zp2	LOC101396080
GATM	Gatm	LOC101395211	PPEF2	Ppef2	LOC101404643	ZP3	Zp3	LOC101406171
GBA2	Gba2	LOC101388907	PPFIA1	Ppfia1	LOC101401542	ZPBP	Zpbp	LOC101403700
GBE1	Gbe1	LOC101400828	PPFIA2	Ppfia2	LOC101396927	ZPBP2	Zpbp2	LOC101401071
GBF1	Gbf1	LOC101390060	PPFIA3	Ppfia3	LOC101400559	ZPLD1	Zpld1	LOC101389927
GBGT1	Gbgt1	LOC101395909	PPFIA4	Ppfia4	LOC101395015	ZPR1	Zpr1	LOC101406124
GBP4	Gbp7	LOC101390877	PPFIBP1	Ppfibp1	LOC101394081	ZRANB1	Zranb1	LOC101401962
GBP5	Gbp5	LOC101398413	PPFIBP2	Ppfibp2	LOC101404593	ZRANB2	Zranb2	LOC101397260
GBP6	Gbp4	LOC101392488	PPHLN1	Pphln1	LOC101405943	ZRANB3	Zranb3	LOC101387442
GBX1	Gbx1	LOC101407611	PPIA	Ppia	LOC101391607	ZRSR2	Zrsr2	LOC101396738
GBX2	Gbx2	LOC101401923	PPIB	Ppib	LOC101408356	ZSCAN10	Zscan10	LOC101402471
GC	Gc	LOC101391117	PPIC	Ppic	LOC101391453	ZSCAN12	Zscan12	LOC101388543
GCA	Gca	LOC101390405	PPID	Ppid	LOC101393946	ZSCAN2	Zscan2	LOC101388584
GCAT	Gcat	LOC101388871	PPIE	Ppie	LOC101405064	ZSCAN20	Zscan20	LOC101407055
GCC1	Gcc1	LOC101399600	PPIF	Ppif	LOC101391236	ZSCAN21	Zscan21	LOC101405996
GCC2	Gcc2	LOC101406739	PPIG	Ppig	LOC101402436	ZSCAN22	Zscan22	LOC101404494
GCDH	Gcdh	LOC101406101	PPIH	Ppih	LOC101404685	ZSCAN25	Zscan25	LOC106803954
GCFC2	Gcfc2	LOC101405095	PPIL1	Ppil1	LOC101402686	ZSCAN26	Zscan26	LOC106803756
GCG	Gcg	LOC101391734	PPIL2	Ppil2	LOC101403727	ZSCAN29	Zscan29	LOC101407214
GCGR	Gcgr	LOC101397512	PPIL3	Ppil3	LOC101387976	ZSCAN30	Zscan30	LOC101397478
GCH1	Gch1	LOC101408115	PPIL4	Ppil4	LOC101388536	ZSWIM1	Zswim1	LOC101404639
GCHFR	Gchfr	LOC101393106	PPIL6	Ppil6	LOC101394120	ZSWIM2	Zswim2	LOC101393294
GCK	Gck	LOC101388191	PPIP5K1	Ppip5k1	LOC101408445	ZSWIM3	Zswim3	LOC101405078
GCKR	Gckr	LOC101400908	PPIP5K2	Ppip5k2	LOC101408177	ZSWIM4	Zswim4	LOC101388126
GCLC	Gclc	LOC101403040	PPL	Ppl	LOC101390854	ZSWIM5	Zswim5	LOC101394024
GCLM	Gclm	LOC101403192	PPM1A	Ppm1a	LOC101394548	ZSWIM6	Zswim6	LOC101391303
GCM1	Gcm1	LOC101403906	PPM1B	Ppm1b	LOC101401077	ZSWIM7	Zswim7	LOC101399310
GCM2	Gcm2	LOC101407786	PPM1D	Ppm1d	LOC101394747	ZSWIM8	Zswim8	LOC101389101

GCNT1	Gcnt1	LOC101407548	PPM1E	Ppm1e	LOC101388089	ZSWIM9	6330408A02F	LOC101397370
GCNT2	Gcnt2	LOC106802113	PPM1F	Ppm1f	LOC101403195	ZUP1	Zufsp	LOC101406769
GCSAM	Gcsam	LOC101404045	PPM1G	Ppm1g	LOC101402553	ZW10	Zw10	LOC101389276
GCSH	Gcsh	LOC106802928	PPM1H	Ppm1h	LOC101406029	ZWILCH	Zwilch	LOC101396077
GDA	Gda	LOC101388882	PPM1J	Ppm1j	LOC101387342	ZWINT	Zwint	LOC101404923
GDAP1	Gdap1	LOC101402987	PPM1K	Ppm1k	LOC101406301	ZXDC	Zxdc	LOC101405738
GDAP1L1	Gdap1l1	LOC101393810	PPM1L	Ppm1l	LOC101402340	ZYG11A	Zyg11a	LOC101403173
GDAP2	Gdap2	LOC101396069	PPM1M	Ppm1m	LOC101400745	ZYG11B	Zyg11b	LOC101387671
GDE1	Gde1	LOC101391130	PPM1N	Ppm1n	LOC106803518	ZYX	Zyx	LOC101399910
GDF1	Gdf1	LOC101407045	PPME1	Ppme1	LOC101397082	ZZEF1	Zzef1	LOC101387995
GDF10	Gdf10	LOC101387991	PPOX	Ppox	LOC101396350	ZZZ3	Zzz3	LOC101401494

Supplementary movie 1. Live-imaging with tracking of SWR-PGCLCs in culture

Supplementary movie 2. Live-imaging with tracking of SWR-ESCs in culture
